# Proteomic footprint of myocardial ischemia/reperfusion injury: *Longitudinal study of the at-risk and remote regions in the pig model*

**DOI:** 10.1038/s41598-017-11985-5

**Published:** 2017-09-27

**Authors:** Aleksandra Binek, Rodrigo Fernández-Jiménez, Inmaculada Jorge, Emilio Camafeita, Juan Antonio López, Navratan Bagwan, Carlos Galán-Arriola, Andres Pun, Jaume Agüero, Valentin Fuster, Borja Ibanez, Jesús Vázquez

**Affiliations:** 1 Fundación Centro Nacional de Investigaciones Cardiovasculares Carlos III (CNIC), Madrid, Spain; 2 The Zena and Michael A. Wiener CVI, Icahn School of Medicine at Mount Sinai, New York, USA; 3 CIBER de Enfermedades Cardiovasculares (CIBERCV), Madrid, Spain; 4 IIS-Fundación Jiménez Díaz Hospital, Madrid, Spain

## Abstract

Reperfusion alters post-myocardial infarction (MI) healing; however, very few systematic studies report the early molecular changes following ischemia/reperfusion (I/R). Alterations in the remote myocardium have also been neglected, disregarding its contribution to post-MI heart failure (HF) development. This study characterizes protein dynamics and contractile abnormalities in the ischemic and remote myocardium during one week after MI. Closed-chest 40 min I/R was performed in 20 pigs sacrificed at 120 min, 24 hours, 4days, and 7days after reperfusion (n = 5 per group). Myocardial contractility was followed up by cardiac magnetic resonance (CMR) and tissue samples were analyzed by multiplexed quantitative proteomics. At early reperfusion (120 min), the ischemic area showed a coordinated upregulation of inflammatory processes, whereas interstitial proteins, angiogenesis and cardio-renal signaling processes increased at later reperfusion (day 4 and 7). Remote myocardium showed decreased contractility at 120 min- and 24 h-CMR accompanied by transient alterations in contractile and mitochondrial proteins. Subsequent recovery of regional contractility was associated with edema formation on CMR and increases in inflammation and wound healing proteins on post-MI day 7. Our results establish for the first time the altered protein signatures in the ischemic and remote myocardium early after I/R and might have implications for new therapeutic targets to improve early post-MI remodeling.

## Introduction

Widespread implementation of reperfusion strategies has dramatically reduced mortality associated with myocardial infarction (MI). One consequence of increased survival is an increased prevalence of chronic heart failure (HF), because patients surviving the acute episode then live with a significantly damaged heart^1,2^. Treatments for post-MI HF have primarily focused on symptom management after the occurrence of irreversible remodeling and functional impairment of the left ventricle (LV)^3^. A better understanding of the molecular mechanisms driving post-ischemia/reperfusion (I/R) cardiac dysfunction at earlier stages might enable the development of therapeutic interventions to prevent the onset of HF^4,5^.

Much of the prevailing knowledge about post-infarcted tissue and the molecular modifications involved comes from studies performed in non-reperfused conditions^6–8^. Surprisingly, although reperfusion is known to alter post-MI myocardial healing^9,10^, to date there have been no systematic studies on the molecular changes occurring in the post-reperfused myocardium at early stages after I/R. Moreover, previous proteomic characterizations of post-I/R myocardial tissue were limited to mitochondrial proteins^11^ or extracellular matrix proteins^12^ or were performed at single time points, mostly at late remodeling stages (weeks after MI)^12–15^. There has also been a notable lack of attention paid to the remote myocardium, which has been used in most studies as a control tissue for the analysis of changes in ischemic areas, thus carrying the implicit assumption that no important changes take place in this area. Unsurprisingly therefore, the molecular alterations taking place in the remote myocardium at early post-MI stages are poorly understood. This lack of knowledge is particularly important because the remote myocardium plays an important role in the development of maladaptive LV remodeling and subsequent HF after MI^16,17^.

In a pig model of myocardial I/R, serial cardiac magnetic resonance (CMR) imaging shows that tissue composition changes dynamically during the first week after infarction^18^. After a transient hyperacute, reperfusion-related edematous reaction, there is a longer, healing-related deferred phase of edema^10^. The precise molecular and cellular events taking place during these highly dynamic early post-I/R phases remain largely unknown.

In this study, we characterize the dynamic changes that take place in both the ischemic and remote myocardium during the first week after infarction in a clinically relevant animal model. This was undertaken by high-throughput multiplexed quantitative proteomics of pig myocardium at different times during the first week after I/R, together with serial *in-vivo* tissue characterization by CMR at each time point. In a first screening approach, we provide a detailed pattern of the time-course behavior of more than 5.000 proteins in both the ischemic and remote areas. This was followed by a further validation step in which we focused on most relevant protein changes. Our results reveal a highly coordinated, multimodal pattern of functional protein alterations in both myocardium regions in the early stages post infarction. Most notably, the remote myocardium undergoes transitory alterations in the contractile machinery that result in a stunned myocardium within the first 24 h. Addressing the need for unbiased “omics” approaches, our data constitute a rich resource of information about the precise molecular signatures and pathways that characterize early remodeling stages, with potential utility for molecular researchers looking for novel drug targets for cardioprotection and HF prevention^5^.

## Results

### Time profile of regional myocardial contractility during early post-I/R remodeling

**Figure 1:**
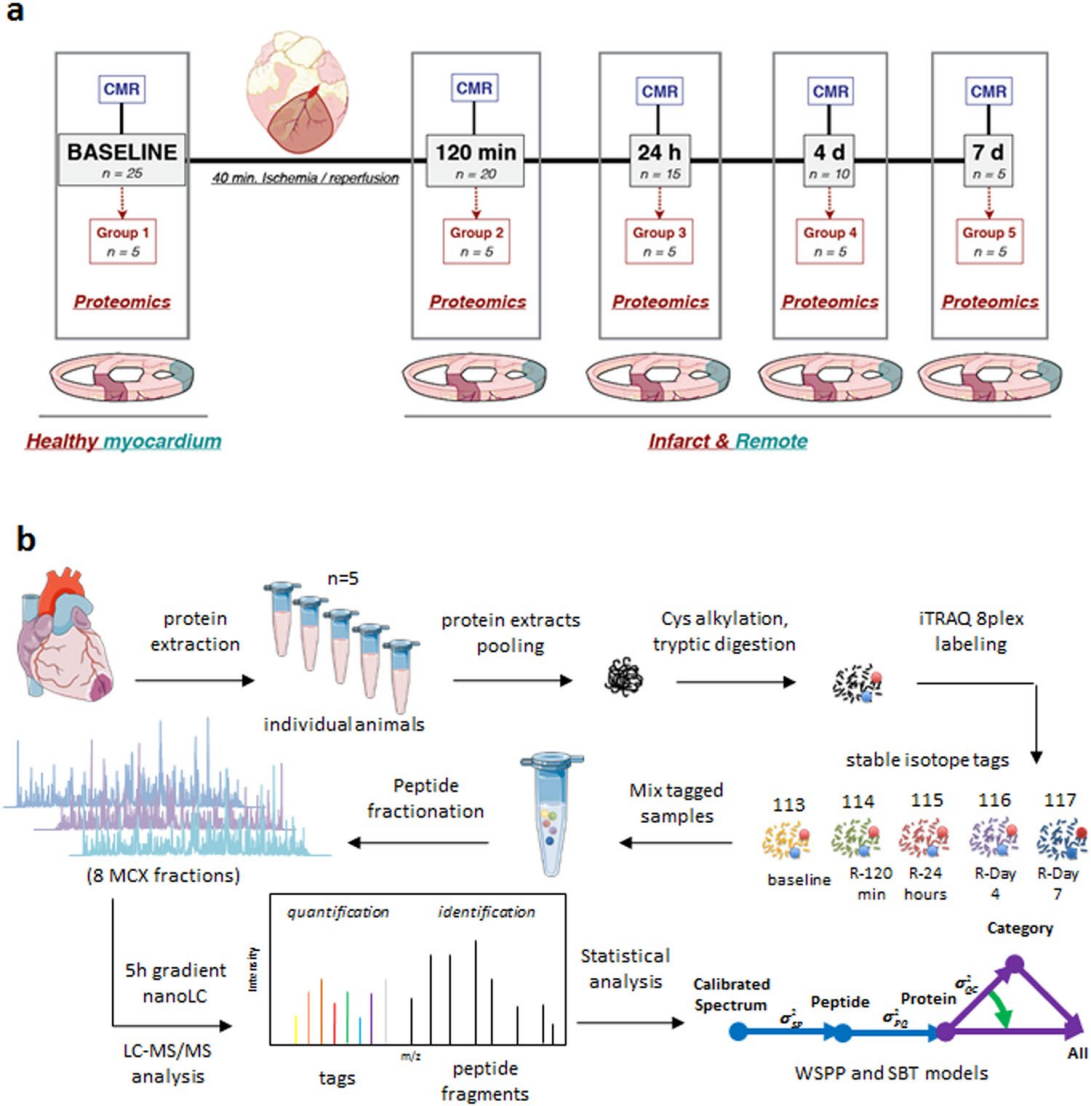
Study design. (**a**) The study population comprised 5 groups of pigs (n = 5/group). Groups 1 to 5 were used for the histopathological characterization of myocardial tissue changes during the first week after ischemia/reperfusion (I/R). CMR (cardiac magnetic resonance) scans included cine steady-state free-precession (SSFP) sequences to provide high quality anatomical references and functional information. CMR scans were performed at all follow-up stages until sacrifice, so that animals sacrificed at day 7 underwent baseline, 120 min, 24 h, day 4, and day 7 CMR. CMR and myocardial water content data from animals in groups 1 to 5 have been reported previously^18^. (**b**) For proteomic analysis, remote and ischemic tissue samples from these animals were processed for protein extraction, tryptic digestion, multiplexed stable isotope labeling, and fractionation followed by nano-liquid chromatography-tandem mass spectrometry (nanoLC-MS/MS) and systems biology analysis. Graphical elements in panel b were adapted from the Servier Medical Art Powerpoint image bank. Servier Medical Art by Servier (http://www.servier.com/Powerpoint-image-bank) is licensed under a Creative Commons Attribution 3.0 Unported License (https://creativecommons.org/licenses/by/3.0/).

**Figure 2:**
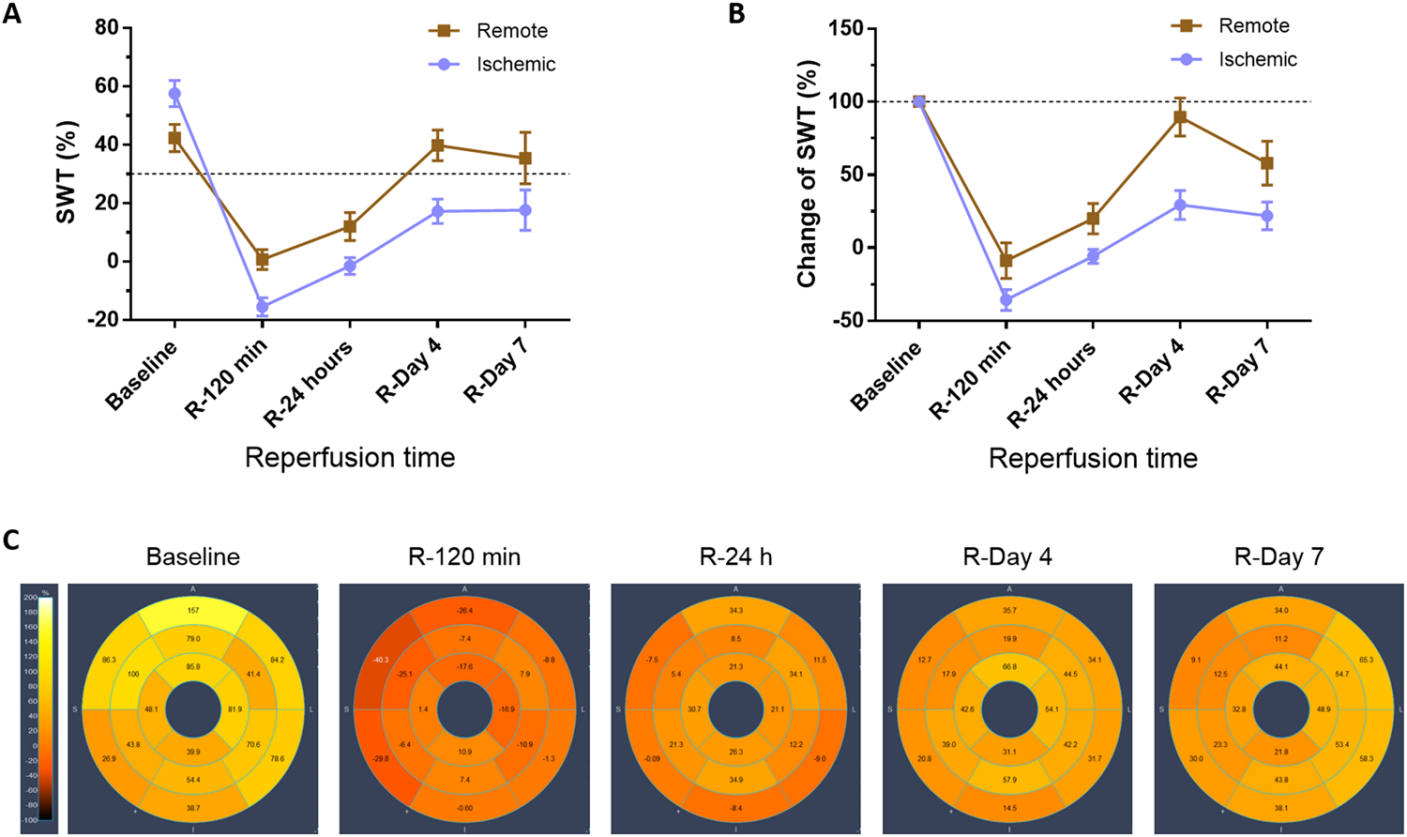
Functional cardiac magnetic resonance during early post-I/R remodeling. Time course of regional contractility in the ischemic and remote myocardium. Systolic wall thickening (SWT) in the ischemic and remote myocardium: (**A**) Absolute values. (**B**) Percentage SWT change relative to baseline. Symbols and bars denote mean and standard error of the mean. Dashed lines indicate reference values from healthy pigs, before induction of myocardial infarction. Myocardial segments were considered dysfunctional if SWT is 30% or less. (**C**) Representative bullseye display of SWT in one pig at baseline and over the first week post I/R after 40-minute mid left anterior descending coronary artery occlusion. Transient contractile dysfunction was observed in the remote myocardium at early post-I/R phases.

Five groups of animals (n = 5 per group) were used in this study (Fig. 1a): (1) control pigs (not undergoing I/R), (2) I/R pigs sacrificed 120 min after reperfusion, (3) I/R pigs sacrificed 1 day after reperfusion, (4) I/R pig sacrificed 4 days after reperfusion, and (5) I/R pigs sacrificed 7 days after reperfusion. Table 1 summarizes functional cardiac CMR data obtained during early remodeling after I/R. As expected, regional contractility in the post-ischemic myocardium, as measured by systolic wall thickening (SWT), was impaired at all time-points evaluated. The remote myocardium also showed a significant transitory alteration in regional myocardial function, with SWT significantly reduced at 120 min and 24 h, and then recovering to baseline values at day 7 post-reperfusion. The SWT time course in the ischemic and remote myocardium is shown in Fig. 2, together with a representative bullseye display of SWT of one pig at all time-points evaluated.

**Table 1:** Left ventricular regional systolic wall thickening analysis in the ischemic and remote pig myocardium during the first week after I/R.

		Baseline	R-120 min	R-24 hours	R-Day 4	R-Day 7
Group 1 (Control)	**I**	75.7 (16.7)				
	**R**	61.8 (20.1)				
Group 2 (I/R-120min)	**I**	38.4 (10.2)	−21.4 (15.1)			
	**R**	18.3 (7.0)	−12.3 (9.3)			
Group 3 (I/R-24hour)	**I**	47.5 (11.6)	−15.8 (12.4)	−4.3 (9.7)		
	**R**	30.6 (14.0)	6.7 (16.1)	9.7 (13.9)		
Group 4 (I/R-4days)	**I**	45.4 (17.7)	−14.6 (9.7)	−3.7 (7.9)	12.9 (16.9)	
	**R**	35.3 (16.3)	−5.5 (9.4)	7.9 (21.5)	39.7 (21.7)	
Group 5 (I/R-7days)	**I**	80.6 (16.2)	−10.2 (18.6)	3.6 (15.3)	21.6 (7.8)	17.6 (15.5)
	**R**	65.6 (14.9)	14.1 (11.3)	18.6 (21.3)	40.0 (12.7)	35.4 (19.7)
Pooled	**I**	**57**.**5 (22**.**1)**	**−15**.**5 (13**.**8)** ^*^	**−1**.**5 (11**.**2)** ^*^	**17**.**2 (13**.**2)** ^*^	**17**.**6 (15**.**5)** ^*^
	**R**	**42**.**3 (23**.**3)**	**0**.**7 (15**.**2)** ^*^	**12**.**0 (18**.**4)** ^*^	**39**.**8 (16**.**7)**	**35**.**4 (19**.**7)**

Left ventricular regional systolic wall thickening in the ischemic and remote myocardium, reported as mean % (standard deviation). A priori pairwise comparisons between baseline and pooled data for each time point were performed using generalized linear mixed models. Multiple comparison adjustment of p-value was performed according to the sequential Holm-Bonferroni procedure. *Statistically significant differences (p < 0.05) between baseline and corresponding pooled time-point data. The different myocardial states (ischemic vs. remote) were initially defined by the localization relative to late gadolinium enhanced defined infarction^41,42^. In the case of baseline imaging, i.e. before ischemia/reperfusion, such a definition was retrospectively performed based on follow-up imaging; while matched areas were selected in the case of controls. I: Ischemic; R: Remote

### Proteome changes in the ischemic myocardium during early stage remodeling after I/R

In a first screening approach, we subjected pooled heart extracts from five animals per group to high-throughput multiplexed quantitative proteomics in three technical replicates. This allowed very precise relative quantification of myocardial proteins with very high proteome coverage. We were able to quantify more than 5000 proteins from the ischemic and remote myocardium. Functional annotation was followed by a novel systems biology analysis developed by our group^19^. This analysis detected statistically significant biological changes (FDR < 0.05), specifically driven by the coordinated action of protein subsets that accurately depict phenotypic alterations in myocardial tissue.

**Figure 3:**
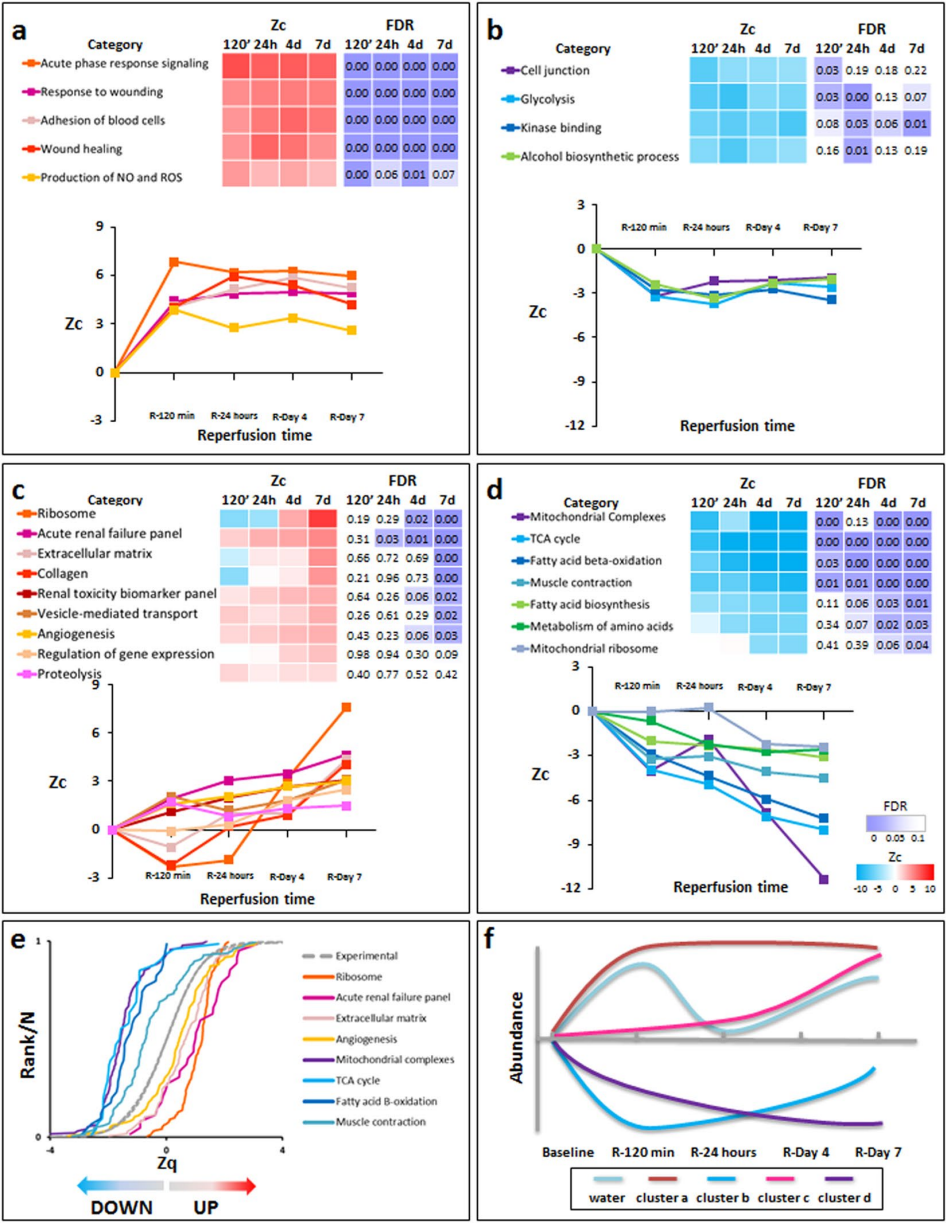
Quantitative proteomics time course analysis of early reperfusion after acute infarction in the ischemic myocardium. Quantitative proteomics screening approach results were analyzed using the Systems Biology Triangle (SBT) model to detect coordinated protein changes in functional categories over time. Functional categories significantly altered (False Discovery Rate, FDR < 0.05) at least one time point were classified into four clusters (**a**–**d**) Cluster a, increasing after 120 min with sustained upregulation for 7 days after ischemia onset; Cluster b, decreasing after 120 min and recovering to baseline values at day 7, except for glycolysis and kinase-binding proteins. Cluster c, increasing at days 4 and 7, with maximum change at day 7; and Cluster d, decreasing over time, with a tendency to reach minimum values at day 7. Protein values (Zq) and functional category values (Zc) are reported as log_2_ fold changes with respect to baseline, in units of standard deviation. The complete set of proteins changing in each category is listed in Supplementary Tables S1–S4. Panel (e) displays the cumulative distributions of Zq from proteins belonging to a set of representative categories from clusters **c** and **d**, showing the high coordination of protein responses. (**f**) Schematic chart of changes in water content and protein clusters. The proteomics results presented here are representative of three technical replicate experiments using five animal sample pools.

Early after reperfusion (120 min), the ischemic myocardium showed a coordinated increase in the expression of proteins involved in acute-phase response signaling, response to wounding, wound healing, blood cell adhesion, and production of nitric oxide and reactive oxygen species (Fig. 3a), and this was accompanied by decreases in proteins involved in glycolysis, alcohol biosynthesis, and kinase binding (Fig. 3b). These processes reflect an early activation of immunological/inflammatory responses in the post-reperfused myocardium. In addition, there was also a coordinated decrease in cell junction proteins (Fig. 3b **)**, which is consistent with the initial wave of edema formation described in this model^18^.

At a later stage after reperfusion (day 4 to day 7), the ischemic myocardium showed a coordinated increase in protein biosynthesis processes, manifested by increased abundance of proteins related to gene expression, ribosomes, and vesicle-mediated protein transport (Fig. 3c). There was also significant increase of collagen and extracellular matrix proteins, peaking at day 7 post-reperfusion, and of angiogenesis-associated proteins, starting at day 4 and peaking at day 7 (Fig. 3c). The category of proteolysis also increased; although this change was less statistically significant, it was consistently increased in the four time points and included a considerable number of proteins (183) (Fig. 3c). These changes correlated with significant fibrosis^10^ and are consistent with a late and gradual activation of healing processes. Conversely, between day 4 and day 7 post I/R the ischemic myocardium showed a generalized decrease of proteins involved in the mitochondrial electron transport chain (ETC) (mitochondrial complexes I-V) (Supplementary Figure S1), fatty acid biosynthesis, amino acid metabolism, fatty acid beta-oxidation, tricarboxylic acid (TCA) cycle, and mitochondrial ribosomes (Fig. 3d), suggesting a diminished mitochondrial content. A coordinated decrease in muscle contraction was also detected (Fig. 3d). These changes seem to reflect the removal of necrotic myocytes (rich in mitochondria) and their replacement by fibroblasts (less mitochondrial density) during healing. Interestingly, this stage was also characterized by a coordinated increase in proteins implicated in acute renal failure and in renal toxicity biomarkers (Fig. 3c), indicating concomitant gradual activation of cardio-renal signaling processes. The coordinated nature of the protein responses detected in this analysis is illustrated in Fig. 3e. A representational diagram that relates the changes in water content with the protein clusters in the ischemic myocardium is shown in Fig. 3f.

**Figure 4:**
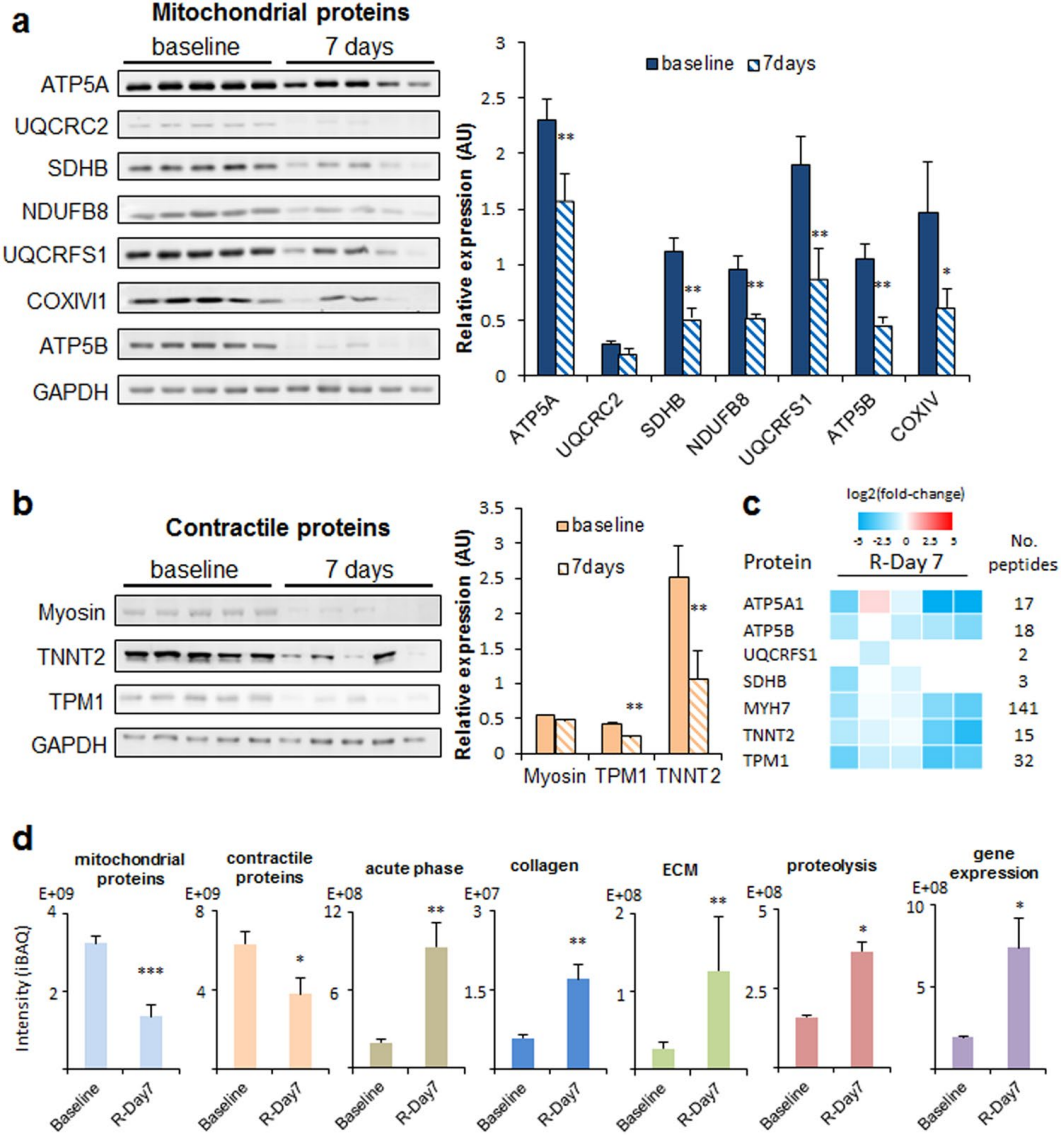
Validation of the most representatitve protein changes detected in ischemic myocardium after acute infarction. (**a**) Western blot analysis and corresponding quantitative densitometry of ATP5A, UQCRC2, SDHB, NDUFB8, UQCRFS1, COXIV and ATP5B confirming the decrease in mitochondrial protein content 7 days after reperfusion with respect to baseline. (**b**) Western blot analysis and quantitative densitometry of cardiac Myosin heavy chain, TNNT2 and TPM1 confirming the decrease in sarcomeric contractile proteins 7 days after reperfusion. Each lane of western blot corresponds to a protein extract obtained from one animal. Protein values are normalized relative to GAPDH. In the bar plots, results are shown as the mean ± s.e.m. of five determinations. *Indicates p value < 0.05; **indicates p value < 0.01 versus baseline by Mann-Whitney test. Full-length blots from panels a and b are presented in Supplementary Figure S8 as well as their raw integrated densities before and after normalization can be consulted in Supplementary Figures S9 and S10. iTRAQ quantifications of the proteins from panels a and b are shown in the Supplementary Figure S11a for comparison. (**c**) Label free individual protein quantifications of ATP5A, ATP5B, UQCRFS1, SDHB, MYH7, TNNT2 and TPM1 confirming the decrease in mitochondrial and contractile protein content. Protein quantifications are represented as log2(fold-change) of iBAQ intensities of individual animals from R-Day 7 compared with pooled average of iBAQ intensities from all Baseline group animals. (**d**) Label free quantitative proteomics confirming the decrease in mitochondrial and contractile proteins and the increase in acute-phase related proteins, collagens, extracellular matrix proteins (ECM), proteolysis related proteins and gene expression related protein contents, 7 days after reperfusion with respect to the baseline. Protein abundances were expressed as the iBAQ values. Results are shown as the mean ± s.e.m of five determinations performed in individual animals, *indicates FDR < 0.05; **indicates FDR < 0.01, ***indicates FDR < 0.001 versus baseline by Mann-Whitney test after Benjamini–Hochberg correction. Label free individual protein quantifications included in the mitochondrial, contractile, acute-phase, collagens, ECM, proteolysis and gene expression protein groups are shown in Supplementary Figure S13.

In the next step of this study, we used alternative approaches to validate the main phenotypic changes. In this step we wanted not only to validate the previous results, but also to determine whether the protein alterations are consistently reproduced in different individuals. Western blotting analysis of a set of representative proteins from complex I (NDUFB8), complex II (SDHB), complex III (UQCRC2, UQCRFS1), complex IV (COXIVI1) and complex V (ATP5A, ATP5B) from ETC confirmed that the decrease in mitochondrial proteins at 7 days after reperfusion was a generalized event suffered by practically all animals analyzed (n = 5) (Fig. 4a). Similar results were obtained when representative contractile proteins from thick filaments (myosin) and from thin filaments (TNNT2, TPM1) were analyzed by western blot (Fig. 4b). Furthermore, label-free mass spectrometry analysis in individual animals (n = 5) also confirmed that the absolute amounts of proteins, expressed as iBAQ intensities^20^, belonging to mitochondria or implicated in contractile processes were decreased (Figs 4c,d, S2 and S13a,b), while acute phase response proteins were clearly increased at 7 days after reperfusion (Figs 4d, S2 and S13c). The total amount of collagens or extracellular matrix proteins were also confirmed to increase (Fig. 4d, S2 and S13d,e). In addition, both the total amount of proteolytic and gene expression proteins were consistently found increased in the animals after 7 days of reperfusion (Figs 4d, S2 and S13f,g). These results suggested that the increase in collagen and extracellular matrix proteins was the result of a net balance between increased mRNA expression and increased proteolytic activity, as proposed by other authors^21^.

### Proteome changes in the remote myocardium during early stage remodeling after I/R

**Figure 5:**
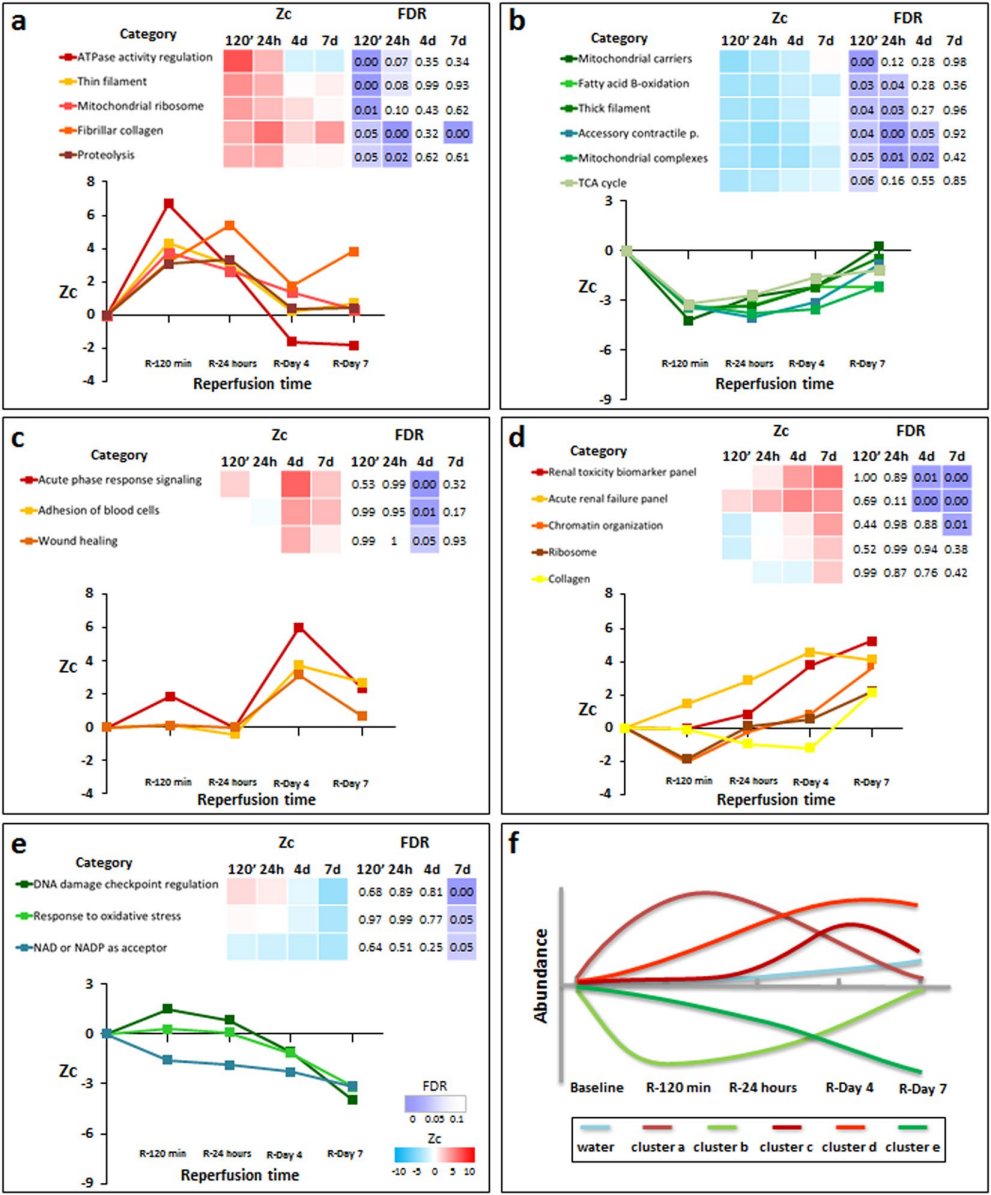
Quantitative proteomics time course analysis of early reperfusion after acute infarction in the remote myocardium. Quantitative proteomics screening approach results are presented as in Fig. 3. Significantly altered functional categories were classified into five clusters (**a**–**e**): Cluster a, showing a transient increase at 120 min or 24 h; Cluster b, decreasing at 120 min and 24 h and reverting to almost normal values at day 7; Cluster c, reaching maximum upregulation at day 4, Cluster d, increasing gradually over time (this cluster included ribosome and collagen categories for comparison of results with the infarcted area); and Cluster e, decreasing in the late phase of reperfusion, reaching minimal values at day 7. Panel (f) shows a schematic chart of changes in water content and protein clusters. The complete set of proteins changing in each category is listed in Supplementary Tables S5–S9. The proteomics results presented here are representative of three technical replicate experiments using five animal sample pools.

At early post-reperfusion stages (120 min to 24 h) the remote myocardium showed a transient coordinated upregulation of proteins related to ATPase activity, muscle thin filament (mainly troponins, tropomyosins, and tropomodulin), proteolysis, fibrillar collagen deposition, and mitochondrial ribosomes (Fig. 5a). Conversely, there was a transient downregulation of muscle thick filament proteins, accessory contractile proteins, and proteins related to several mitochondrial processes (including carriers, tricarboxylic acid cycle, fatty acid beta-oxidation, and ETC complexes, mainly affecting complexes III and IV) (Figs 5b, S3 and S4). With the exception of fibrillar collagen, values for these coordinated processes returned to normal by day 7 after reperfusion (Figs 5a,b and S3). The imbalance in the proportion of sarcomere components strongly suggests uncoupling of the finely-tuned contractile machinery in the remote myocardium early after myocardial infarction. Notably, unlike the pattern seen in the infarcted area (Figs 3d, 4a,c and S1), mitochondrial proteins in the remote myocardium did not all follow the same trend (Figs 5a,b and S4). This finding suggests that mitochondrial protein alterations do not reflect a decrease in mitochondrial number, but rather a progressive dysfunctional imbalance. Furthermore, this transient uncoupling of the mitochondrial and contractile protein machinery correlated with the temporary contractile dysfunction (SWT) observed by CMR during the first 24 h of reperfusion (Fig. 2). These findings clearly indicate that the molecular alterations triggered by I/R have a transient impact on remote myocardium function early during reperfusion.

Functional analysis in the remote myocardium in late reperfusion phases (4 and 7 days post I/R) revealed an increase in proteins related to acute phase response signaling, wound healing, and blood cell adhesion (Fig. 5c and d). These functional alterations correlated with the linear edema in this area, revealed by progressively increasing CMR T2 relaxation times during the first week after reperfusion^18,22^. Furthermore, there were also increases in proteins related to chromatin organization and cardio-renal signaling processes (acute renal failure and renal toxicity biomarkers), reaching peak values on days 4 and 7 post-I/R (Fig. 5d). Collagen and ribosome-related proteins showed a similar trend, although with lower statistical significance. Downregulated proteins included NAD and NADP acceptor proteins and proteins implicated in oxidative stress and DNA damage (Fig. 5e). Finally, the biological processes grouped in Fig. 5c and d show intriguing time offset/timeline discrepancies compared with the observations in the ischemic myocardium. A schematic diagram of changes in water content and protein categories clusters which occur in the remote myocardium is represented in Fig. 5f.

**Figure 6:**
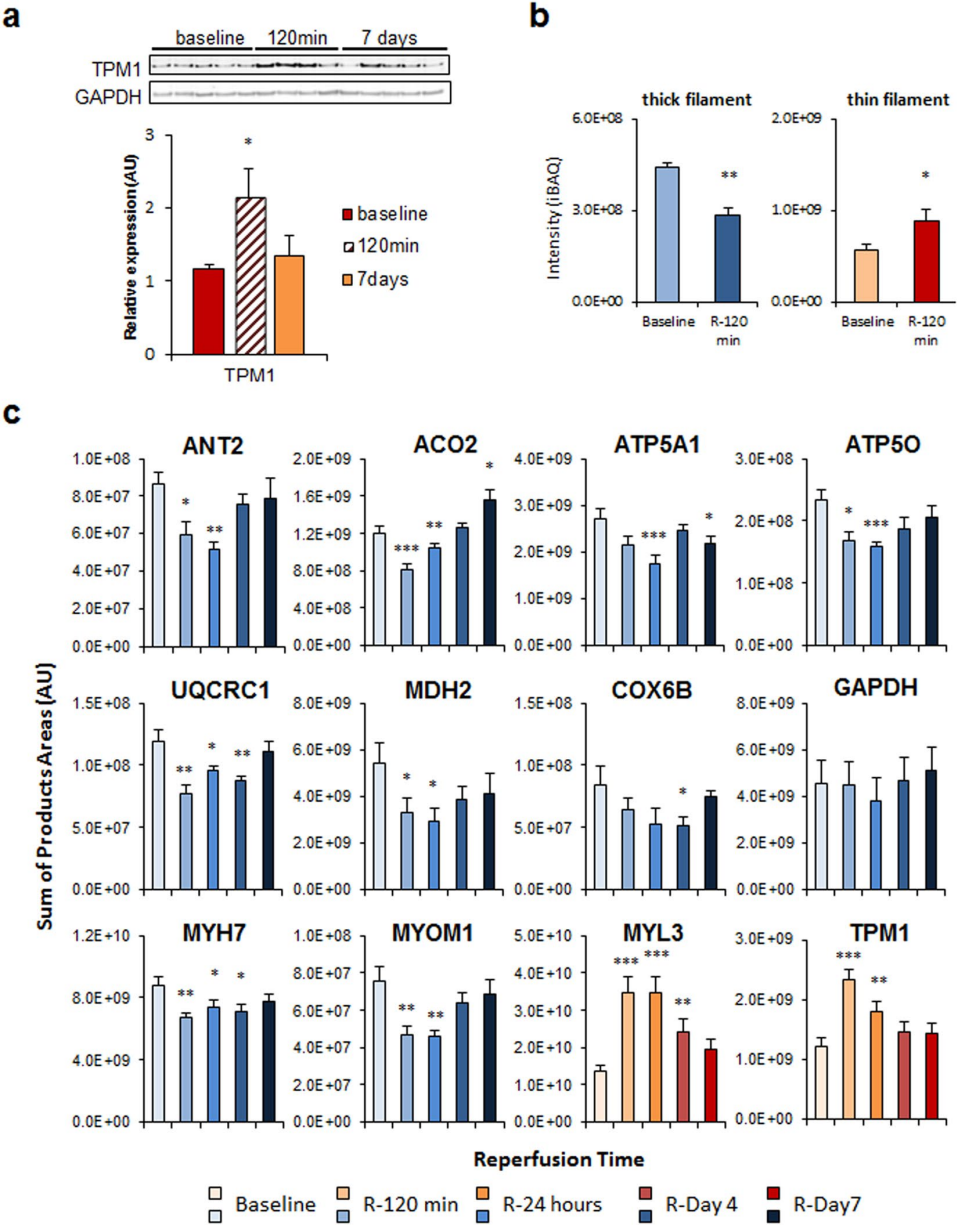
Validation of the most representatitve protein changes detected in remote myocardium after acute infarction. (**a**) Western blot analysis and quantitative densitometry of TPM1 confirming the transient increase in thin filament sarcomeric proteins early after reperfusion. Full-length blots are presented in Supplementary Figure S8 as well as their raw integrated densities before and after normalization can be consulted in Supplementary Figure S12. Protein values are normalized relative to GAPDH. Results are shown as the mean ± s.e.m. of values obtained in at least four animals. *Indicates p value < 0.05 versus baseline by Mann-Whitney test. (**b**) Label free proteomics analysis confirming the decrease in thick filament proteins and an increase in thin filament proteins early after reperfusion (in at least four animals). Results are expressed as in Fig. 4d. Label free individual protein quantifications included in the thick filament and thin filament protein groups are shown in Supplementary Figure S14. (**c**) Quantitative analysis by PRM targeted proteomics of proteins ANT2, ACO2, ATP5A1, ATP5O, UQCRC1, MDH2, COX6B, GAPDH, MYH7, MYOM1, MYL3 and TPM1, confirming the transient changes in abundance of mitochondrial and contractile sarcomeric proteins after early reperfusion. Protein values are normalized by the total base peak area of the chromatograms. Results are shown as the mean ± s.e.m. of at least four animals at each time point in two technical replicates. *Indicates *p value* < 0.05; **indicates *p value* < 0.01, ***indicates *p value* < 0.001 versus baseline by Mann-Whitney test. iTRAQ quantifications of the proteins from panels a and c are shown in the Supplementary Figure S11b for comparison.

**Figure 7:**
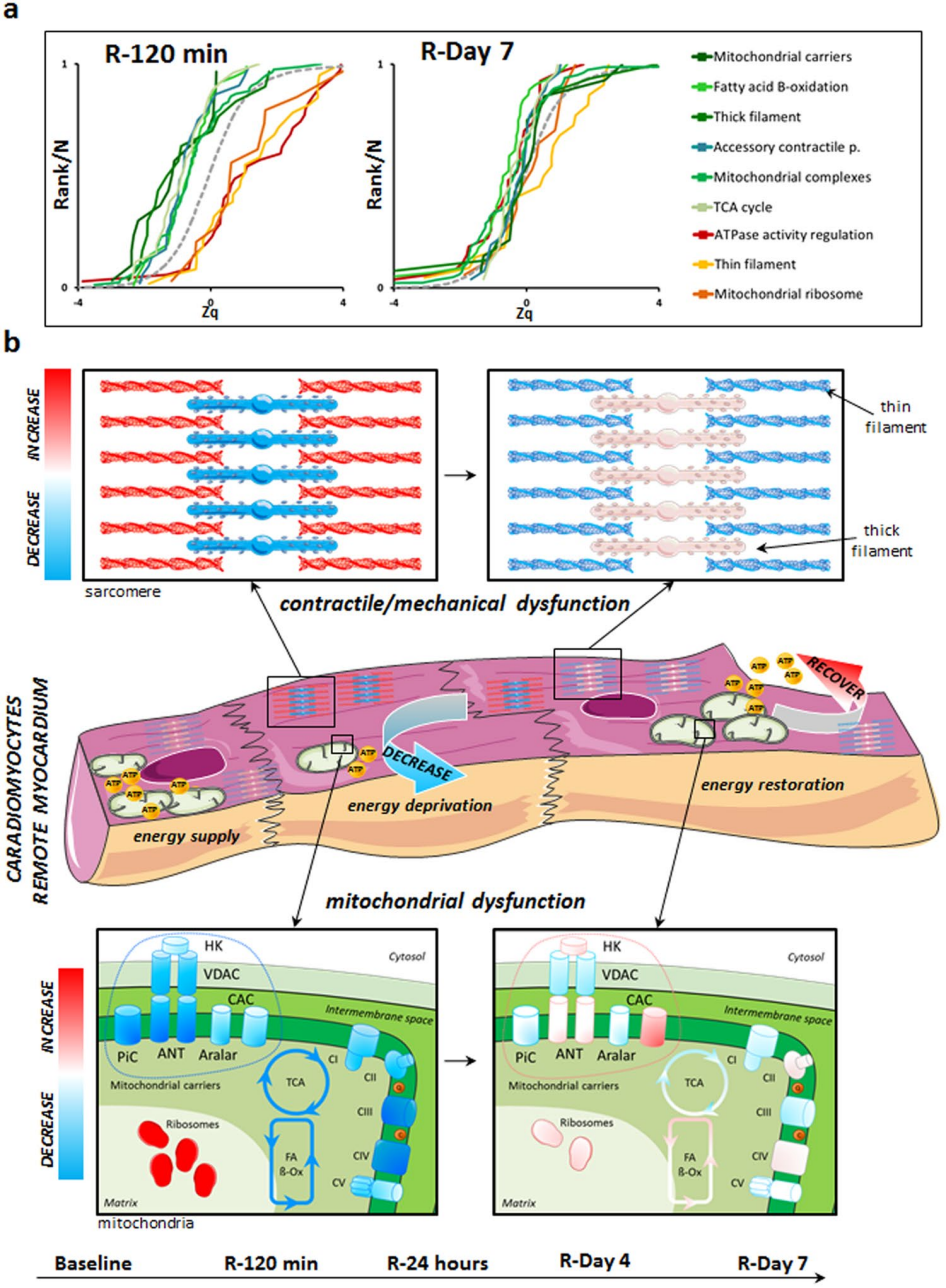
Affected functional processes in sarcomeres and mitochondria of the remote myocardium. (**a**) Cumulative distributions of all Zq values from proteins in the categories depicted in (Supplementary Figure S3) after 120 min (left) or 7 days (right) of reperfusion; for clarity, all proteins belonging to mitochondrial electron transport chain (ETC) complexes are grouped into one category. (**b**) Outline of the mechanical uncoupling within the contractile machinery and the transient decrease in processes crucial for energy production (tricarboxylic acid (TCA) cycle, fatty-acid beta-oxidation, mitochondrial complexes III and IV) and energy transfer and supply (mitochondrial substrate/solute carrier proteins). For clarity, mitochondrial complexes are drawn as simple entities, neglecting their complex structures. Graphical elements in Fig. 7 were adapted from the Servier Medical Art Powerpoint image bank. Servier Medical Art by Servier (http://www.servier.com/Powerpoint-image-bank) is licensed under a Creative Commons Attribution 3.0 Unported License (https://creativecommons.org/licenses/by/3.0/).

The main phenotypic changes in remote myocardium were also validated by alternative analysis. Western blot results obtained in at least four animals confirmed the generalized transitory increase at 120 min after reperfusion of a contractile protein belonging to thin filaments (TPM1) (Fig. 6a). Label-free mass spectrometry of individual animal samples (at least n = 4 per each condition) also confirmed that the absolute amount of proteins belonging to thick filaments was decreased at 120 min, while those belonging to thin filaments were increased (Figs 6b, S5 and S14). A more accurate quantification of specific groups of proteins was also performed in individual animal samples (at least n = 4 per each condition) by targeted proteomics, performing two technical replicates per sample. These analysis confirmed the transient decrease at early times (120 min to 1 day) followed by subsequent recovery (7 days after reperfusion) of proteins belonging to mitochondrial carriers (ANT2), TCA cycle (ACO2, MDH), complex V (ATP5A1, ATP5O), complex III (UQCRC1), complex IV (COX6B), thick filaments (MYH7) or the accessory contractile proteins category (MYOM1). Similarly, we could also confirm the transient increase at early times of proteins implicated in ATPase activity regulation (MYL3) or belonging to thin filaments (TPM1). The protein used as internal control (GAPDH) was not significantly altered (Figs 6c and S6). Finally, as stated in the previous paragraphs, in our exploratory iTRAQ experiments of the remote myocardium tissue, we detected a coordinated initial disengagement (at 120 min) of the values of mitochondrial and sarcomeric proteins followed by the latter coordinated restoration to normal (at day 7), which is shown in Fig. 7a-left and Fig. 7a-right, respectively.

## Discussion

In the present study, advanced proteomic analysis in a pig model of I/R provides quantitative time-course information on thousands of proteins and unravels an array of coordinated early changes in the heart proteome after MI. We have assessed not only the particular altered protein signatures that characterize the post-reperfused ischemic and remote myocardium, but also their relation to functional changes detected by CMR. This is the first study reporting a comprehensive proteomics analysis of molecular changes during early reperfusion in the post-MI remote myocardium.

Early reperfusion is characterized by an abrupt edematous reaction^10,18^ and a rapid and profound acute inflammatory response^23,24^. Upon reperfusion, intracellular, mitochondrial and sarcolemma Ca^2+^ concentrations increase abruptly, inducing cardiomyocyte hypercontraction and opening of the mitochondrial permeability transition pore (MPTP), the hallmark of reperfusion-induced cardiomyocyte death^25^. MPTP opening collapses the mitochondrial membrane potential and disconnects the electron transport chain, resulting in ATP depletion and subsequent cell death^26^. Our deep proteomics analysis of ischemic myocardium fully supports and extends these observations by establishing the time-course of hundreds of protein changes and by demonstrating a coordinated activation of proteins implicated in inflammation, wound healing, and production of reactive oxygen species as soon as 120 min after reperfusion. Furthermore, at these early time-points, we identified downregulation of cell junction and glycolytic metabolism proteins, consistent with the reperfusion-induced interstitial edema^10^ and the disruption of the normal mitochondria function^11,27^. Our study identifies a rich repository of early-response proteins directly related to reperfusion injury that could contribute to the development of therapies and diagnostic tools^5^.

At later reperfusion phases (4 and 7 days post reperfusion), coordinated protein changes in the ischemic myocardium indicated activation of protein biosynthesis, synthesis of collagen and interstitial proteins, and formation of new vasculature, together with decreases in mitochondrial and muscle proteins. These processes reflect a phenotypic change in the infarcted myocardial tissue caused by the replacement of necrotic myocytes (rich in mitochondria and with high metabolic activity) by collagen, extracellular matrix proteins, and fibroblasts (lower mitochondrial content and metabolic activity). Notably, in the ischemic myocardium we also identified a progressive increase in proteins related to cardio-renal signaling, suggesting active cross-talk between the acutely injured heart and the kidney.

The remote myocardium plays an important role in ventricular function after AMI, and acute post-AMI HF can be exacerbated by transitory stunning of the non-ischemic region^28^. Similarly, the long-term inflammatory response in the remote region can lead to progressive adverse remodeling, chronic ventricular dilatation, and HF^17^. Therefore, understanding the molecular changes occurring in the remote region is of the utmost importance for the development of therapeutic strategies targeting this region. However, the early post-infarct behavior of the remote tissue, particularly at the molecular level, has remained unexplored until now. In this study, we contribute to fill this gap of knowledge by providing a first insight into the transiently stunned myocardium and a highly detailed characterization of phenotypic events taking place in the remote area during the first week after I/R.

We demonstrate that I/R significantly impacts the remote myocardium, inducing molecular changes that correlate precisely with CMR-evaluated alterations in contractile function (transient stunned myocardium) and tissue composition (late progressive edema). In the period from 120 min to 24 h after reperfusion, transient molecular alterations of sarcomere components are detected in the remote myocardium, associated with alterations in mitochondrial components (Figs 7a and S3), coinciding with CMR-detected temporal alterations in systolic wall thickness. At later phases (one week post I/R), these changes reverted to the levels detected in the control sample, coinciding with restored systolic contractile function in the remote myocardium.

Acute loss of myocardial function after MI has classically been considered to trigger rapid increases in loading conditions and adaptive responses to preserve stroke volume; these responses include deformation of the remote myocardium, which alters Frank/Starling relations, and activation of the sympathetic adrenergic and renin-angiotensin-aldosterone systems^29^. Our results suggest that these responses might be temporarily maladaptive, because the decrease in regional contractility in the remote myocardium early after reperfusion is almost fully recovered some days later.

Our findings indicate that abrupt post-MI overload of the heart’s contractile apparatus and the temporary energy-supply cut-off during early reperfusion induces a compensatory response in the remote myocardium involving transient molecular changes in thin filament components, mitochondrial biosynthesis machinery, and ATPase regulators. However, these adaptive changes are accompanied by a temporary mitochondrial dysfunction that affects energy-producing components (ETC, TCA cycle, fatty acid beta-oxidation, and mitochondrial energy carriers), while there are transient alterations in other sarcomere components (thick filaments and accessory contractile proteins) (Fig. 7b). This view is supported by recent evidence that thin and thick filaments are independently and separately regulated^30^. Likewise, transcriptional analysis of the failing mammalian myocardium suggests an orchestrated dysregulation of energy supply (metabolism) and expenditure (muscle contraction and ion homeostasis)^31^. In addition, the strong alteration of mitochondrial complexes III and IV (at 120 min and 24 h, Supplementary Figure S2) is in good agreement with a recent report that succinate accumulating during ischemia in mice is rapidly metabolized after reperfusion, producing a large proton motive force through ETC complexes III and IV^32^.

State-of-the-art T2 mapping CMR sequences show a moderate edematous reaction appearing progressively several days after I/R, evidenced by the progressive increase in T2 relaxation times during the first week after reperfusion^18,22^. These results also correlate precisely with the proteomics analysis in the remote myocardium, which shows changes in proteins involved in inflammation, wound healing, and fibrotic processes. Notably, edema in the remote region follows a different pattern to that found in the ischemic region^18,22^. The at-risk region shows a bimodal pattern of edema, with the initial wave secondary to reperfusion and the deferred associated with tissue repair; in contrast, in the remote region edema formation is unimodal and progressive. In the infarcted region, activation of inflammatory response proteins coincides with the initial edema wave, whereas in the remote region it correlates with the later progressive edema formation. This observation reinforces the idea that these edematous processes are closely related to inflammation and indicate that the inflammatory response in the remote region is delayed by some days relative to the reaction in the infarcted region. Inflammation also coincided with increased collagen levels that were also observed in the remote myocardium at these later reperfusion phases^10^. A subtle post-MI inflammation in the remote myocardium is also supported by the accumulation in this region of monocytes and macrophages, albeit more slowly and in lower amounts than the ischemic myocardium^33^.

The different timing of inflammatory responses in the remote and ischemic post-MI myocardium might play an important role in modulating extracellular matrix protein composition, which contributes to adverse remodeling of the remote region and heart failure^33–37^. Well-healed infarcts contain large amounts of extracellular matrix, occupying up to 80% of the infarcted area. Collagen deposition in the non-infarcted remote myocardium occurs predominantly in the interstitium, where it contributes to ventricular stiffness and dysfunction^16^. Although collagen deposition initially helps to maintain ventricular morphology after MI, over time it can contribute to geometric changes and functional deterioration^35^. Phenotypically transformed myofibroblasts and inflammatory cells, including those located in the remote myocardium, seem to be central to fibrosis at remote sites after MI due to their possible contribution to the upregulation of the renin-angiotensin system, which is strongly implicated in ventricular remodeling^16,17,38^.

### Limitations

The results presented are from a juvenile healthy animal model in the absence of comorbidities and classical risk factors for cardiovascular disease; therefore, care should be taken in extrapolating to the clinic^39^. Nonetheless, the pig is one of the most clinically translatable large animal models for the study of I/R issues, because unlike other mammals its coronary artery anatomy and distribution resembles that of humans, including a minimal pre-existing coronary collateral flow^40^. The use of a large animal model is of great translational value, especially considering the difficulty of performing such a comprehensive myocardial tissue analysis at the molecular level in patients. The time course of tissue changes in the post-I/R myocardium in pigs, albeit slightly briefer, is similar to that observed in humans^10,41^. In general, cardiac contractility and performance, and therefore cardiac tolerance against ischemia/reperfusion injury might be influenced by anesthesia with additional potential inter- and intra-individual variations in experimental models. We tried to overcome this limitation by performing different but complimentary analysis as follows. Firstly, we analyzed the individual percentage SWT change relative to baseline; therefore taking account for inter-individual variations. Secondly, we analyzed SWT results by using generalized linear mixed models for ischemic and remote myocardium separately; therefore taking account for repeated measurements, inter- and intra-individual variations. Finally, we demonstrated that infarct transmurality and final tissue damage was very similar among pigs. All these data together reinforce the notion that the time profile of regional contractility and final injury inflicted after I/R was quite homogeneous in our experimental model with little influence (if any) of anesthesia in our results, though we cannot definitely exclude any potential interaction.

Another limitation is that the proteomics results in the present study come from homogenized myocardial tissue; therefore it is not possible to differentiate protein expression in different cell types or myocardial layers. Similarly, our data lacks information on subcellular location, on post-translational modifications and on the activity of the proteins quantified. A related problem is the possibility that during reperfusion, blood may occasionally enter the interstitial area through damaged vessels, influencing the proteomics results. The total amount of proteins potentially originating from plasma is, however, very low (Supplementary Figure S7), suggesting that if present, this kind of contamination is insufficient to significantly alter our results.

Finally, the incomplete annotation and functional classification of *Sus scrofa* proteins is also a problem that difficulties biological interpretation. Although we have managed this issue by assigning pig proteins to their human counterparts on the basis of shared peptide sequences, extrapolation of pig protein results to humans should be done with caution.

We present the first comprehensive proteomics analysis of the post-I/R myocardium, covering the first week after infarction. Our results highlight a dynamic pattern of molecular responses that take place in the ischemic and remote myocardium, linked to functional changes revealed by CMR. The results provide evidence of intriguing transformations in the remote non-infarcted myocardium during the first week after I/R, as demonstrated by striking local proteome changes, temporarily reduced contractility function, and the presence of edema in this region. These findings provide insight into the precise molecular signatures underlying early remodeling stages in both the ischemic and the remote myocardium, with potential for the development of new therapeutic approaches to preventing post-MI HF.

## Methods

### Study design and myocardial infarction procedure

The study population was formed by 25 castrated male 3 to 4 month old Large-White pigs weighing 30 to 40 kg. The studies on swine were conducted in accordance with the *Guide for the Care and Use of Laboratory Animals* (the Institute of Laboratory Animal Resources, 1996), and with the approval of Centro Nacional de Investigaciones Cardiovasculares Carlos III (CNIC) Institutional Animal Research Committee (CNIC 05/13) and the Regional Animal Research Committee (PROEX 51/13). We previously reported the dynamic changes in edema formation (assessed by T2 mapping CMR) and tissue composition (assessed by histology) in the ischemic area in same cohort of pigs^18^. The study design is summarized in Fig. 1a. Five pigs (Group 1) were sacrificed with no intervention other than baseline CMR, and served as controls. In 20 pigs, reperfused transmural acute myocardial infarction (I/R) was induced experimentally by closed-chest 40-minute mid left anterior descending coronary artery occlusion followed by balloon deflation and reestablishment of blood flow^18,41,42^. These pigs were sacrificed at 120 min (n = 5, Group 2), 24 h (n = 5, Group 3), 4 days (n = 5, Group 4), and 7 days (n = 5, Group 5). CMR scans were performed at every follow-up stage until sacrifice (i.e. animals sacrificed on day 7 underwent baseline, 120 min, 24 h, day 4, and day 7 CMR exams). The animals were euthanized immediately after the last follow-up CMR scan, and transmural myocardial tissue samples from ischemic and remote areas were rapidly collected for proteomic evaluations. Based on anatomical correlates and standard left ventricle segmentation, those areas from mid-apical ventricular short axis slices matching regional contractility analysis were selected for sampling collection. Detailed information about the study design and myocardial infarction procedure can be found in a previous publication^18^.

### CMR protocol and analysis

CMR exams were performed immediately before MI induction and at subsequent post-MI follow-up time points until sacrifice. All CMR studies were conducted with a Philips 3-Tesla Achieva Tx whole body scanner (Philips Healthcare, Best, the Netherlands) equipped with a 32-element phased-array cardiac coil. The imaging protocol included a standard segmented cine steady-state free-precession (SSFP) sequence to provide high quality anatomical references and segmental regional contractility^18,41,42^. CMR images were analyzed using dedicated software for regional contractility (QMass MR 7.6; Medis, Leiden, the Netherlands) by two observers experienced in CMR analysis and blinded to group allocation. To quantify end-diastolic wall thickness (EDWT), end-systolic wall thickness (ESWT), LV volumes, and LV mass, epicardial and endocardial contours were detected automatically, and corrected manually on short-axis cine SSFP if needed. Systolic wall thickening (SWT) was calculated according to the formula (ESWT − EDWT)/EDWT ×100. SWT was summed and then averaged from mid-apical anteroseptal and anterior segments, for the ischemic area, and mid-apical inferolateral and inferior segments, for the remote area. Myocardial segments were considered dysfunctional if SWT was 30% or lower^43^. Detailed information about imaging parameters and CMR analysis can be found in the Supplementary Information file.

### Mass spectrometry analysis, and protein identification and quantification

Pigs were sacrificed at different time-points after CMR (120 min, 1 day, 4 days, and 7 days post-reperfusion). Samples from the ischemic and remote myocardium of all pigs were collected within minutes of euthanasia and processed for proteomic analysis (Fig. 1b). The 40 minutes-I/R protocol applied in our experimental setting is able to induce a transmural necrosis with virtually no viable myocardium within the ischemic area^10,18,41,42^; therefore ensuring analysis at pure infarcted and remote myocardium levels. Protein extracts were obtained by tissue homogenization with ceramic beads (MagNa Lyser Green Beads apparatus, Roche, Germany) in extraction buffer (50 mM Tris-HCl, 1 mM EDTA, 1.5% SDS, pH 8.5). Free Cys residues were blocked with 50 mM iodoacetamide at the time of protein extraction. For the screening proteomics approach, the protein extracts for each time-point from five biological replicate samples were pooled according to their concentration. Samples were subjected to tryptic digestion and the resulting peptides were labeled with 8-plex isobaric tags for relative and absolute quantification (iTRAQ) and separated by cation exchange chromatography. The fractionated peptides were analyzed by nano-liquid chromatography-tandem mass spectrometry (nanoLC-MS/MS) using a Q-Exactive hybrid quadrupole orbitrap mass spectrometer (Thermo Scientific). The proteomics results presented in Figs 3 and 5 are representative of three technical replicate experiments.

Protein identification was performed using the SEQUEST HT algorithm integrated in Proteome Discoverer 1.4 (Thermo Scientific). Since *Sus scrofa* gene and protein annotation is not complete, MS/MS scans were searched against a combined pig and human database (UniProtKB/Swiss-Prot 2014_02 Release); pig proteins are given priority when they share peptides with human proteins. Peptides were identified from MS/MS data using the probability ratio method^44^. False discovery rate (FDR) of peptide identifications was calculated by the refined method^45,46^. Quantitative information was extracted from the MS/MS spectra of iTRAQ-labeled peptides using the in-house program QuiXoT, as described^47^. Differential protein expression was analyzed using the WSPP model^47,48^, which uses raw quantifications as input data and computes the protein log_2_-fold changes (FC) expressed in units of standard deviation around the averages (Zq) for each condition (Groups 2–5), with respect to the control (Group 1). For protein quantification, no limit was imposed on the minimum number of peptides per protein^47,48^. A quantitative proteomics result of total protein changes globally between remote and ischemic myocardium during the first week of reperfusion is summarized in Supplementary Table S10. Alterations in biological functions as a consequence of the coordinated behavior of proteins, was analyzed by estimating functional category averages (Zc) according to the Systems Biology Triangle (SBT) model^19^. The quantified proteins were functionally annotated using the Ingenuity Knowledge Database (IPA)^49,50^ and DAVID^51^. The DAVID repository includes 13 functional databases, including Gene Ontology, KEGG, and Panther. Further details about proteomics and statistical analysis can be found in the Supplementary Information file.

### Parallel Reaction Monitoring Analysis

Samples from individual pigs used in this analysis were obtained from five animals for each time-point (baseline, R-24 hours, R-Day 4 and R-Day 7) and four animals for time-point R-120 min. For the targeted monitoring, we selected the peptides which were identified with the highest SEQUEST score (Xcorr) and that belonged to representative proteins from the functional categories (Supplementary Table S11). Quantitative targeted protein analyses for each biological replicates were performed by parallel reaction monitoring (PRM)^52^ by nano-liquid chromatography-tandem mass spectrometry (nanoLC-MS/MS) using a QExactive HF Hybrid Quadrupole-Orbitrap Mass Spectrometer (Thermo Scientific) in two technical replicates. Raw mass spectrometry files (Thermo) and spectral libraries (msf files obtained with Proteome Discoverer 2.1 search engine) were imported into Skyline version 3.6.0.10162 ((https://skyline.gs.washington.edu) for identification of transitions and peak area integration according to the software instructions^53^. Only b- or y- fragment ions were selected to build the elution profile for peptide quantitation. All extracted ion chromatograms (XICs) of selected fragments were manually inspected and adjusted to ensure proper peak picking and peak integration.

### Label Free Proteomics Analysis

Samples used in this analysis obtained from 4 individual pigs (time-point R-120 min) or 5 individual pigs (time-points baseline, R-24 hours, R-Day 4 and R-Day 7) were processed as described above. Label free experiments for all biological replicates were acquired using nano-liquid chromatography-tandem mass spectrometry (nanoLC-MS/MS) in a QExactive HF Hybrid Quadrupole-Orbitrap Mass Spectrometer (Thermo Scientific). MS data were acquired with a Top10 data‐dependent MS/MS scan method (topN method). Mass spectrometry raw files were analyzed by MaxQuant software (version 1.5.6.5)^54^. Label‐free protein quantitation (LFQ) was performed with a minimum ratio count of 1^55^. Intensity-based absolute quantification (iBAQ)^20^ implemented in MaxQuant was used for the analysis of the label-free proteomics experiments. We express the protein abundances as percentage of the identified proteome, obtained by normalizing the iBAQ intensities to the sum of all intensities.

### Western Immunoblotting Analysis

Validation by western blot was performed on selected representative proteins from each functional category according to antibody availability, giving priority to the proteins identified with the highest number of peptides by mass spectrometry. Immunoblotting was performed according to standard protocols. Briefly, 10 μg of heart tissue extracts were loaded per lane separated on the 4–10% gel and transferred to polyvinylidene fluoride (PVDF) (Immobilon-FL, Milipore) membranes for fluorescence applications. Validation of proteomics results was performed using the following antibodies: ATP5A (Abcam, ab14748), UQCRC2 (Abcam, ab14745), SDHB (Abcam, ab14714), NDUFB8 (Abcam, ab110242), UQCRFS1 (Abcam, ab14746), COXIV (Abcam, ab14744), ATP5B (Abcam, ab14730), cardiac Myosin (Abcam, ab50967), TPM1 (Abcam, ab133292), TNNT2 (Abcam, ab10214) and GAPDH (Santa Cruz Biotechnology, sc-32233). Secondary antibodies goat anti-mouse IgG DyLight 800 (Rockland, 610-145-121) and goat anti-rabbit IgG Alexa Fluor 680 (Thermo Fisher Scientific, A-21076) were used against the corresponding primary antibodies and the images were acquired with the ODYSSEY Infrared Imaging System (LI-COR).

### Statistical analysis

Normal distribution of each data subset was checked using graphical methods and a Shapiro–Wilk test. For quantitative CMR variables, data are expressed as mean and standard deviation. To take into account repeated measures, the time course of regional contractility was analyzed with generalized linear mixed models. Pairwise comparisons were made between baseline and all other CMR time points, and p-values were adjusted for multiple comparisons by the sequential Holm-Bonferroni method. Statistical analyses were performed with Stata 12.0 (StataCorp, College Station, Texas). Western blot analysis to determine significant differences between groups after densitometry analysis and statistical analysis of absolute peptide quantitation by PRM were performed using the Mann-Whitney test, using the GraphPad Prism 7.02 software. Results of intensity-based absolute quantification (iBAQ) of label free proteomics experiments were tested for significance using the Mann-Whitney test with a false discovery rate of <0.05 after Benjamini–Hochberg correction.

### Data availability

The dataset from the analysis of the pig myocardium proteome (raw files and excel tables with peptide and protein quantification data), the label-free and the PRM analysis raw data as well as the raw western blot data from the ODISSEY scanner are available in the PeptideAtlas repository http://www.peptideatlas.org/PASS/PASS00891), which can be downloaded via ftp.peptideatlas.org.

## Electronic supplementary material

Supplemental Material

## References

[1] Menees DS (2013). Door-to-balloon time and mortality among patients undergoing primary PCI. The New England journal of medicine.

[2] Eapen ZJ (2012). Defining heart failure end points in ST-segment elevation myocardial infarction trials: integrating past experiences to chart a path forward. Circulation. Cardiovascular quality and outcomes.

[3] de Couto G, Ouzounian M, Liu PP (2010). Early detection of myocardial dysfunction and heart failure. Nat Rev Cardiol.

[4] Lindsey ML (2015). Transformative Impact of Proteomics on Cardiovascular Health and Disease: A Scientific Statement From the American Heart Association. Circulation.

[5] Varga ZV (2015). Functional Genomics of Cardioprotection by Ischemic Conditioning and the Influence of Comorbid Conditions: Implications in Target Identification. Curr Drug Targets.

[6] Chen W (2016). Endothelial Actions of ANP Enhance Myocardial Inflammatory Infiltration in the Early Phase After Acute Infarction. Circulation research.

[7] Kain V, Prabhu SD, Halade GV (2014). Inflammation revisited: inflammation versus resolution of inflammation following myocardial infarction. Basic Res Cardiol.

[8] Peng Y (2014). Top-down proteomics reveals concerted reductions in myofilament and Z-disc protein phosphorylation after acute myocardial infarction. Mol Cell Proteomics.

[9] Basso C, Rizzo S, Thiene G (2010). The metamorphosis of myocardial infarction following coronary recanalization. Cardiovascular pathology: the official journal of the Society for Cardiovascular Pathology.

[10] Fernandez-Jimenez R (2015). Pathophysiology Underlying the Bimodal Edema Phenomenon After Myocardial Ischemia/Reperfusion. J Am Coll Cardiol.

[11] Fernandez-Caggiano M (2016). Analysis of Mitochondrial Proteins in the Surviving Myocardium after Ischemia Identifies Mitochondrial Pyruvate Carrier Expression as Possible Mediator of Tissue Viability. Molecular & cellular proteomics: MCP.

[12] Barallobre-Barreiro J (2012). Proteomics analysis of cardiac extracellular matrix remodeling in a porcine model of ischemia/reperfusion injury. Circulation.

[13] Chang YH (2015). Quantitative proteomics reveals differential regulation of protein expression in recipient myocardium after trilineage cardiovascular cell transplantation. Proteomics.

[14] Cieniewski-Bernard C (2008). Proteomic analysis of left ventricular remodeling in an experimental model of heart failure. J Proteome Res.

[15] Page BJ (2015). Revascularization of chronic hibernating myocardium stimulates myocyte proliferation and partially reverses chronic adaptations to ischemia. J Am Coll Cardiol.

[16] van den Borne SW (2010). Myocardial remodeling after infarction: the role of myofibroblasts. Nat Rev Cardiol.

[17] Heusch G (2014). Cardiovascular remodelling in coronary artery disease and heart failure. The Lancet.

[18] Fernandez-Jimenez R (2015). Myocardial edema after ischemia/reperfusion is not stable and follows a bimodal pattern: imaging and histological tissue characterization. J Am Coll Cardiol.

[19] Garcia-Marques F (2016). A Novel Systems-Biology Algorithm for the Analysis of Coordinated Protein Responses Using Quantitative Proteomics. Mol Cell Proteomics.

[20] Schwanhausser B (2011). Global quantification of mammalian gene expression control. Nature.

[21] Zamilpa R, Lindsey ML (2010). Extracellular matrix turnover and signaling during cardiac remodeling following MI: causes and consequences. J Mol Cell Cardiol.

[22] Fernandez-Jimenez R (2015). Fast T2 gradient-spin-echo (T2-GraSE) mapping for myocardial edema quantification: first *in vivo* validation in a porcine model of ischemia/reperfusion. J Cardiovasc Magn Reson.

[23] Ibanez B, Heusch G, Ovize M (2015). & Van de Werf, F. Evolving therapies for myocardial ischemia/reperfusion injury. J Am Coll Cardiol.

[24] Vogel B, Shinagawa H, Hofmann U, Ertl G, Frantz S (2015). Acute DNase1 treatment improves left ventricular remodeling after myocardial infarction by disruption of free chromatin. Basic Res Cardiol.

[25] Ong SB, Samangouei P, Kalkhoran SB, Hausenloy DJ (2015). The mitochondrial permeability transition pore and its role in myocardial ischemia reperfusion injury. Journal of molecular and cellular cardiology.

[26] Inserte J (2016). Studies on the role of apoptosis after transient myocardial ischemia: genetic deletion of the executioner caspases-3 and -7 does not limit infarct size and ventricular remodeling. Basic Res Cardiol.

[27] Smeele KM (2011). Disruption of hexokinase II-mitochondrial binding blocks ischemic preconditioning and causes rapid cardiac necrosis. Circulation research.

[28] Chan W (2012). Acute left ventricular remodeling following myocardial infarction: coupling of regional healing with remote extracellular matrix expansion. JACC Cardiovasc Imaging.

[29] Sutton MG, Sharpe N (2000). Left ventricular remodeling after myocardial infarction: pathophysiology and therapy. Circulation.

[30] Kampourakis T, Sun YB, Irving M (2016). Myosin light chain phosphorylation enhances contraction of heart muscle via structural changes in both thick and thin filaments. Proc Natl Acad Sci USA.

[31] Barth AS, Kumordzie A, Tomaselli GF (2016). Orchestrated regulation of energy supply and energy expenditure: Transcriptional coexpression of metabolism, ion homeostasis, and sarcomeric genes in mammalian myocardium. Heart Rhythm.

[32] Chouchani ET (2014). Ischaemic accumulation of succinate controls reperfusion injury through mitochondrial ROS. Nature.

[33] Frantz S, Nahrendorf M (2014). Cardiac macrophages and their role in ischaemic heart disease. Cardiovasc Res.

[34] Hori M, Nishida K (2009). Oxidative stress and left ventricular remodelling after myocardial infarction. Cardiovasc Res.

[35] Baysa A (2015). The p66ShcA adaptor protein regulates healing after myocardial infarction. Basic Res Cardiol.

[36] Martire A (2016). Mesenchymal stem cells attenuate inflammatory processes in the heart and lung via inhibition of TNF signaling. Basic Res Cardiol.

[37] Robinson E (2015). Exendin-4 protects against post-myocardial infarction remodelling via specific actions on inflammation and the extracellular matrix. Basic Res Cardiol.

[38] Sun Y (2009). Myocardial repair/remodelling following infarction: roles of local factors. Cardiovasc Res.

[39] Ferdinandy P, Hausenloy DJ, Heusch G, Baxter GF, Schulz R (2014). Interaction of risk factors, comorbidities, and comedications with ischemia/reperfusion injury and cardioprotection by preconditioning, postconditioning, and remote conditioning. Pharmacol Rev.

[40] Fernández-Jiménez R, Fernández-Friera L, Sánchez-González J, Ibáñez B (2014). Animal Models of Tissue Characterization of Area at Risk, Edema and Fibrosis. Curr Cardiovasc Imaging Rep.

[41] Fernández-Jiménez, R. *et al*. Dynamic Edematous Response of the Human Heart to Myocardial Infarction: Implications for Assessing Myocardial Area at Risk and Salvage. *Circulation*, doi:10.1161/circulationaha.116.025582 (2017). 10.1161/CIRCULATIONAHA.116.025582 PMC5625960 28687712

[42] Fernández-Jiménez, R. *et al*. Effect of Ischemia Duration and Protective Interventions on the Temporal Dynamics of Tissue Composition After Myocardial Infarction. *Circ Res* **121**, 439–450, 10.1161/CIRCRESAHA.117.310901 (2017). 10.1161/CIRCRESAHA.117.310901 PMC5542781 28596216

[43] Nowosielski M (2009). Comparison of wall thickening and ejection fraction by cardiovascular magnetic resonance and echocardiography in acute myocardial infarction. Journal of cardiovascular magnetic resonance: official journal of the Society for Cardiovascular Magnetic Resonance.

[44] Martinez-Bartolome S (2008). Properties of average score distributions of SEQUEST: the probability ratio method. Mol Cell Proteomics.

[45] Navarro P, Vazquez J (2009). A refined method to calculate false discovery rates for peptide identification using decoy databases. J Proteome Res.

[46] Bonzon-Kulichenko E, Garcia-Marques F, Trevisan-Herraz M, Vazquez J (2015). Revisiting peptide identification by high-accuracy mass spectrometry: problems associated with the use of narrow mass precursor windows. J Proteome Res.

[47] Navarro P (2014). General statistical framework for quantitative proteomics by stable isotope labeling. J Proteome Res.

[48] Martinez-Acedo P (2012). A novel strategy for global analysis of the dynamic thiol redox proteome. Molecular & cellular proteomics: MCP.

[49] Calvano SE (2005). A network-based analysis of systemic inflammation in humans. Nature.

[50] Ficenec D (2003). Computational knowledge integration in biopharmaceutical research. Brief Bioinform.

[51] Huang da, W. *et al*. Extracting biological meaning from large gene lists with DAVID. *Curr Protoc Bioinformatics* Chapter 13, Unit 13 11, doi:10.1002/0471250953.bi1311s27 (2009). 10.1002/0471250953.bi1311s27 19728287

[52] Peterson AC, Russell JD, Bailey DJ, Westphall MS, Coon JJ (2012). Parallel reaction monitoring for high resolution and high mass accuracy quantitative, targeted proteomics. Mol Cell Proteomics.

[53] MacLean B (2010). Skyline: an open source document editor for creating and analyzing targeted proteomics experiments. Bioinformatics.

[54] Cox, J. & Mann, M. In *Nat Biotechnol* Vol. 26 1367–1372 (2008). 10.1038/nbt.1511 19029910

[55] Cox J (2014). Accurate proteome-wide label-free quantification by delayed normalization and maximal peptide ratio extraction, termed MaxLFQ. Mol Cell Proteomics.

