# Supplementary material for: Proteomic footprint of myocardial ischemia/reperfusion injury: Longitudinal study of the at-risk and remote regions in the pig model
Source: Sci Rep. 2017 Sep 27;7:12343. doi: 10.1038/s41598-017-11985-5 (PMC5617837; doi:10.1038/s41598-017-11985-5)
Supplement: Supplementary file 1 — Supplemental Material [file 41598_2017_11985_MOESM1_ESM.pdf]

SUPPLEMENTARY INFORMATION FILE

Proteomic footprint of myocardial ischemia/reperfusion injury: *Longitudinal study of the at-risk and remote regions in the pig model*

Aleksandra Binek[†1], MSc; Rodrigo Fernández-Jiménez[†1,2,3], MD; Inmaculada Jorge[1,3], PhD; Emilio Camafeita[1,3], PhD; Juan Antonio López[1,3], PhD; Navratan Bagwan[1], MSc; Carlos Galán-Arriola[1,3], DVM; Andres Pun[1,3], DVM; Jaume Agüero[1,3], MD; Valentin Fuster[1,2], MD PhD; Borja Ibanez*[1,3,4], MD PhD; Jesús Vázquez*[1,3], PhD

[1]Fundación Centro Nacional de Investigaciones Cardiovasculares Carlos III (CNIC), Madrid, Spain
[2]The Zena and Michael A. Wiener CVI, Icahn School of Medicine at Mount Sinai, New York, USA
[3]CIBER de Enfermedades Cardiovasculares (CIBERCV), Madrid, Spain
[4]IIS-Fundación Jiménez Díaz Hospital, Madrid, Spain
[†]these authors contributed equally to this work
*corresponding authors

SUPPLEMENTAL METHODS

Study design

Experiments were performed on castrated male Large-White pigs weighing 30 to 40 kg. The study population comprised a total of 25 pigs that completed the protocol. The study was approved by the institutional animal research committee. The study design is summarized in main text **Fig. 1**. Five pigs (Group 1) were sacrificed with no intervention other than baseline CMR, and served as controls. In the other 20 pigs, reperfused acute myocardial infarction (I/R) was induced experimentally by closed-chest 40-minute mid left anterior descending coronary artery occlusion. These pigs were sacrificed at 120 minutes (n=5, Group 2), 24 hours (n=5, Group 3), and 7 days (n=5, Group 4) after reperfusion. CMR scans were performed at every follow-up stage until sacrifice (i.e. animals sacrificed on day 7 underwent baseline, 120min, 24h, day4, and day7 CMR exams). Animals were immediately euthanized after the last follow-up CMR scan, and transmural myocardial tissue samples from ischemic and remote areas were rapidly collected for proteomics evaluation. Based on anatomical correlates and standard left ventricle segmentation, those areas from mid-apical ventricular short axis slices matching regional contractility analysis were selected for sampling collection.

Myocardial infarction procedure

The I/R protocol has been detailed elsewhere.[1] Anesthesia was induced by intramuscular injection of ketamine (20 mg/kg), xylazine (2 mg/kg), and midazolam (0.5 mg/kg) and maintained by continuous intravenous infusion of ketamine (2 mg/kg/h), xylazine (0.2 mg/kg/h), and midazolam (0.2 mg/kg/h). Animals were intubated and mechanically ventilated with oxygen (fraction of inspired O_2: 28%). Central venous and arterial lines were inserted, and a single bolus of unfractioned heparin (300 IU/kg) was administered at the onset of instrumentation. The left anterior descending coronary artery, immediately distal to the origin of the first diagonal branch, was occluded for 40 minutes with an angioplasty balloon introduced via the percutaneous femoral route using the Seldinger technique. Balloon location and

maintenance of inflation were monitored angiographically. After balloon deflation, a coronary angiogram was recorded to confirm patency of the coronary artery. A continuous infusion of amiodarone (300 mg/h) was maintained during the procedure in all pigs to prevent malignant ventricular arrhythmias. In cases of ventricular fibrillation, a biphasic defibrillator was used to deliver non-synchronized shocks.

CMR protocol

A baseline CMR scan was performed immediately before myocardial infarction and subsequently repeated at corresponding post-infarction follow-up time points until sacrifice. All studies were performed in a Philips 3-Tesla Achieva Tx whole body scanner (Philips Healthcare, Best, the Netherlands) equipped with a 32-element phased-array cardiac coil. The imaging protocol included a standard segmented cine steady-state free-precession (SSFP) sequence to provide high quality anatomical references and functional information.[1-3] The imaging parameters for the SSFP sequence were FOV 280 x 280 mm, slice thickness 6 mm with no gap, TR 2.8 ms, TE 1.4 ms, flip angle 45°, cardiac phases 30, voxel size 1.8 x 1.8 mm, and 3 NEX. SSFP sequences were performed to acquire 13-15 contiguous short axis slices covering the heart from the base to the apex.

CMR data analysis

CMR images were analyzed using dedicated software (QMass MR 7.6, Medis, The Netherlands) by two observers experienced in CMR analysis and blinded to group allocation. To quantify end-diastolic wall thickness (EDWT), end-systolic wall thickness (ESWT), LV volume, and LV mass, epicardial and endocardial contours were detected automatically and corrected manually on short-axis cine SSFP if needed. Segmental wall thickening (SWT) was calculated as (ESWT − EDWT)/EDWT × 100. SWT was summed and then averaged from mid-apical anteroseptal and anterior segments, for the ischemic area, and mid-apical inferolateral and inferior segments, for the remote area. Myocardial segments were considered dysfunctional if SWT was 30% or less.[4]

Proteomic analysis

Tissue sample preparation for mass spectrometry analysis

To compare the pattern of protein alterations produced by ischemia-reperfusion, myocardial tissue samples (from the ischemic and remote regions) were collected from the control group (no intervention) and at 120 min, 1 day, 4 days, and 7 days after ischemia and reperfusion. Protein extracts were prepared from homogenized tissue using ceramic beads (MagNa Lyser Green Beads apparatus, Roche, Germany) in extraction buffer (50mM Tris-HCl, 1mM EDTA, 1.5% SDS, pH 8.5). Protein digestion was performed as previously described[5]. Briefly, Cys residues were blocked with iodoacetamide (Sigma) at the time of protein extraction. Extracts were quantified by RC/DC Protein Assay (Bio Rad) and stored at -80ºC until tryptic digestion. Before digestion, proteins were reduced with DTT (GE Healthcare) and subsequently alkylated with MMTS (Thermo Scientific). For shotgun proteomics, protein extracts from each time point were pooled, ensuring equal contributions from each replicate. Proteins from pooled samples were digested using sequencing grade trypsin (Promega, Madison, WI, USA) and filter-assisted sample preparation technology (FASP, Expedeon, San Diego, CA, USA), and the resulting peptides were subjected to iTRAQ 8-plex labeling (AB Sciex, Framingham, MA, USA), joined, and desalted. For parallel reaction, monitoring (PRM) and label-free quantitative (iBAQ) analysis protein extract from individual pig samples (five pigs per each time point) were trypsin digested separately and desalted before LC-MS/MS analysis. In PRM experiments each sample was acquired in two technical replicates.

Peptide fractionation

Labeled peptides were separated into 8 fractions using Waters Oasis MCX cartridges (Waters Corp, Milford, MA, USA) and graded concentrations of ammonium formate, pH 3.0 (AF3) in acetonitrile (ACN). Desalted and dried peptides were taken up in 1 mL 5 mM AF3. The MCX cartridge was equilibrated by slowly passing 1 mL of 1:1 methanol:water across the cartridge, followed by 3 mL of AF3 containing 25% ACN (v/v). Samples were applied at a flow rate of 1 drop per second and the cartridge was washed with 1 mL AF3-25% ACN. Bound peptides were

eluted into 8 fractions with 300 uL of freshly prepared buffer: (1) 200 mM AF3, 25% ACN; (2) 350 mM AF3, 25% ACN; (3) 500 mM AF3, 25% ACN; (4) 1 M AF3; (5) 1.5 M AF3, 37,5% ACN; (6) 500 mM AF3, 25% ACN, 1 M potassium chloride (KCl); (7) 500mM AF3, 25% ACN, 1.5 M KCl; and (8) 1M AF3, 50% ACN. Obtained fractions were purified and desalted with the MiniSpin Column Kit (The Nest Group, Inc., Southborough, MA, USA), dried, and stored at −20°C until MS analysis.

Liquid chromatography tandem mass spectrometry (nanoLC-MS/MS)

For shotgun proteomics analysis, the tryptic peptide mixtures were subjected to nanoLC-MS/MS. High-resolution analysis was performed on a nano-HPLC Easy nLC 1000 liquid chromatograph (Thermo Scientific, San Jose, CA, USA) coupled to a QExactive Hybrid Quadrupole-Orbitrap mass spectrometer (Thermo Scientific). Peptides were suspended in 0.1% FA, loaded onto a C18 RP nano-precolumn (Acclaim PepMap100, 75-um internal diameter, 3-um particle size and 2-cm length, Thermo Scientific), and separated on an analytical C18 nano-column (EASY-Spray column PepMap RSLC C18, 75-um internal diameter, 3-um particle size and 50-cm length, Thermo Scientific) in a continuous gradient (8–31%B for 240 min, 31–90%B for 2 min, 90%B for 7 min, 90-5%B for 3 min and 2%B for 30 min, where A is 0.1% formic acid in HPLC H_2O and B is 90% ACN, 0.1% formic acid in HPLC grade H_2O). Spectra were acquired using full ion-scan mode over the mass-to-charge (m/z) range 390–1500 and 70,000 FT-resolution. MS/MS was performed on the top twenty ions in each full MS scan in data-dependent acquisition mode with 45s dynamic exclusion enabled. For PRM and label-free quantitative (iBAQ) analysis, peptide samples were subjected to a nano-HPLC nLC 1200 liquid chromatography (Thermo Scientific) coupled to a QExactive HF Hybrid Quadrupole-Orbitrap mass spectrometer (Thermo Scientific). Peptides were suspended in 0.1% FA, loaded onto a C18 RP nano-precolumn (Acclaim PepMap100, 75-um internal diameter, 3-um particle size and 2-cm length, Thermo Scientific), and separated on an analytical C18 nano-column (Acclaim PepMap100, 75-um internal diameter, 3-um particle size and 50-cm length, Thermo Scientific) in a continuous gradient (10-30%B for 60 min, 30-90%B form 2 min, 90%B for 5 min, 90-5%B

for 3 min and 5%B for 25 min, where A is 0.1% formic acid in HPLC H_2O and B is 80% ACN, 0.1% formic acid in HPLC grade H_2O). A Picotip emitter nanospray needle (New Objective, Woburn, MA, USA) was used for peptide ionization. Spectra were acquired using full ion-scan mode over the mass-to-charge (m/z) range 390–1500 and 70,000 FT-resolution. In label-free experiments MS data were acquired with a Top10 data-dependent MS/MS scan method (topN method).

Protein identification and quantification

Proteins were identified in the raw files using the SEQUEST HT algorithm integrated in Proteome Discoverer 1.4 (Thermo Finnigan). MS/MS scans were matched against a combined pig and human database (UniProtKB/Swiss-Prot 2014_02 Release). For database searching, parameters were selected as follows: trypsin digestion with 2 maximum missed cleavage allowed, precursor mass tolerance of 800 ppm, and a fragment mass tolerance of 0.02 ppm. The N-terminal and lysine iTRAQ-8plex modifications were chosen as fixed modifications, whereas methionine oxidation, cysteine carbamidomethylation, and cysteine methylthiolation were chosen as variable modifications. The same MS/MS spectra collections were searched against inverted databases constructed from the same target databases. SEQUEST results were analyzed by the probability ratio method[6]. False discovery rate (FDR) was calculated for peptides identified in the inverted database search results using the refined method[7,8]. Dynamic protein expression profiles after myocardial ischemia/reperfusion were characterized using the relative quantification approach by iTRAQ8-plex stable isotope labeling (SIL) with tandem mass spectrometry (MS/MS). Quantitative information was extracted from MS/MS spectra of iTRAQ-labeled samples using the in-house-developed QuiXoT program, as described[9].

For label-free proteomics analysis mass spectrometry raw files were processed by MaxQuant software (version 1.5.6.5)[10], and peptide lists were searched against the human Uniprot FASTA database (version July 2016). A contaminants database by the Andromeda search engine[11] with cysteine carbamidomethylation, cysteine methylthiolation and methionine oxidations as variable modifications was used. We set the false discovery rate (FDR) to 0.01 for

protein and peptide levels and the FDR was determined by searching a reverse database. A maximum of two missed cleavages were allowed. All proteins and peptides matching to the reversed database were filtered out. Label-free protein quantitation (LFQ) was performed with a minimum ratio count of 1[12]. Intensity-based absolute quantification (iBAQ)[13] method implemented in MaxQuant proteomics identification and quantitation software was used for the analysis of the label-free proteomics experiments. We express the protein abundances as percentage of the identified proteome, obtained by normalizing the iBAQ intensities to the sum of all intensities.

In the PRM experiments the peptides that were selected for assay development were identified in our previous shotgun proteomics analysis [Supplemental Table 10]. Once MS^2 data is generated by the PRM method, raw mass spectrometry files (Thermo) and spectral libraries (msf files obtained with Proteome Discoverer 2.1 search engine) were imported into Skyline version 3.6.0.10162 ((https://skyline.gs.washington.edu) for identification of transitions and peak area integration according to the software instructions[14]. A spectral library was created using data from the original shotgun proteomics assay as well as the FASTA sequences of the monitored proteins were imported into Skyline for *in silico* tryptic digestion to generate precursor ion list. Only *b*- or *y*- fragment ions were selected to build the elution profile for peptide quantitation. All extracted ion chromatograms (XICs) of selected fragments were manually inspected and adjusted to ensure proper peak picking and peak integration.

Statistical analysis

For comparative analysis of protein abundance changes, we applied the Weighted Scan-Peptide-Protein (WSPP) statistical workflow. [5] As input, WSPP uses a list of quantifications in the form of log2-ratios (for example a condition versus control sample) with their statistical weights. From these, WSPP generates the standardized forms of the original variables by computing the quantitative values expressed in units of standard deviation around the means (Zq). For the protein functional analysis we used the Systems Biology Triangle (SBT) model developed in our group, which estimates functional category averages (Zc) from protein values by performing

the protein-to-category integration. To facilitate detection of similar categories (categories sharing many proteins), a clustering algorithm was applied, as described.[15]

Protein functional annotation

Quantified proteins were functionally annotated using the Ingenuity Knowledge Database (IPA)[16,17] and DAVID[18]. The DAVID repository included 13 functional databases, including Gene Ontology, KEGG, and Panther.

SUPPLEMENTAL DATA

Supplementary Figure S1. Functional categories representing mitochondrial ETC complexes that were significantly altered (FDR < 0.05) at least one time point within the ischemic myocardium.

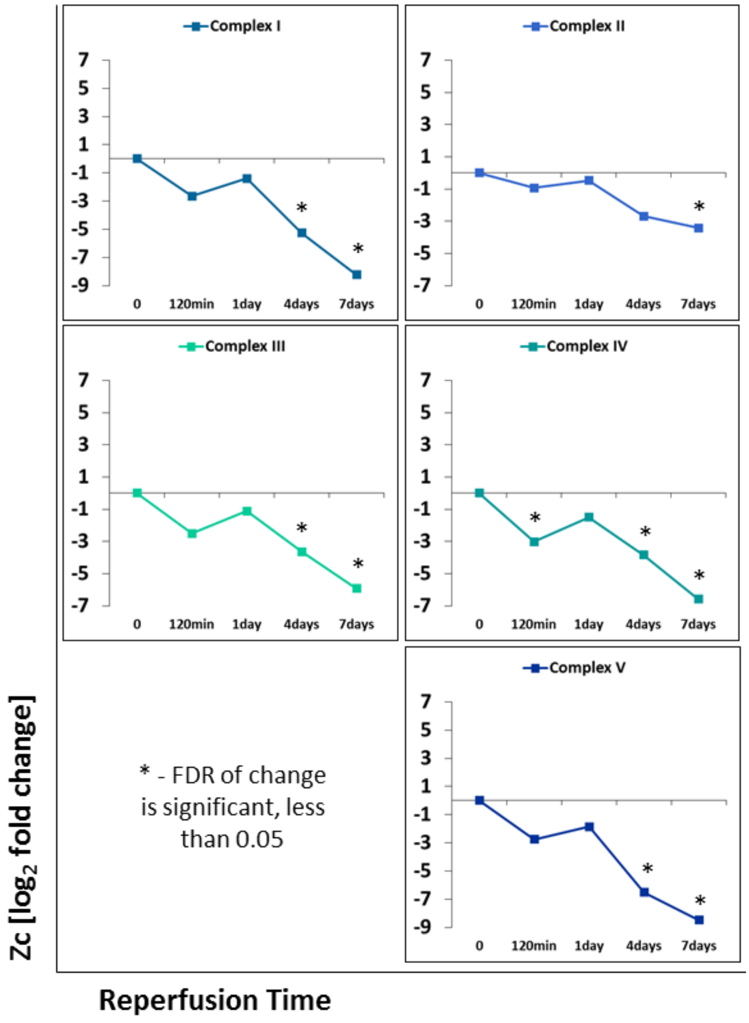

Supplementary Figure S2. Label-free experiments in the ischemic myocardium tissue. In iBAQ analysis the normalized sum of protein values in a biological processes (proteolysis, gene expression, acute phase) and other structural proteins (contractile proteins, extracellular matrix, collagen and mitochondrial proteins) is indicated for each biological individual (protein values are normalized by sum off all intensities).

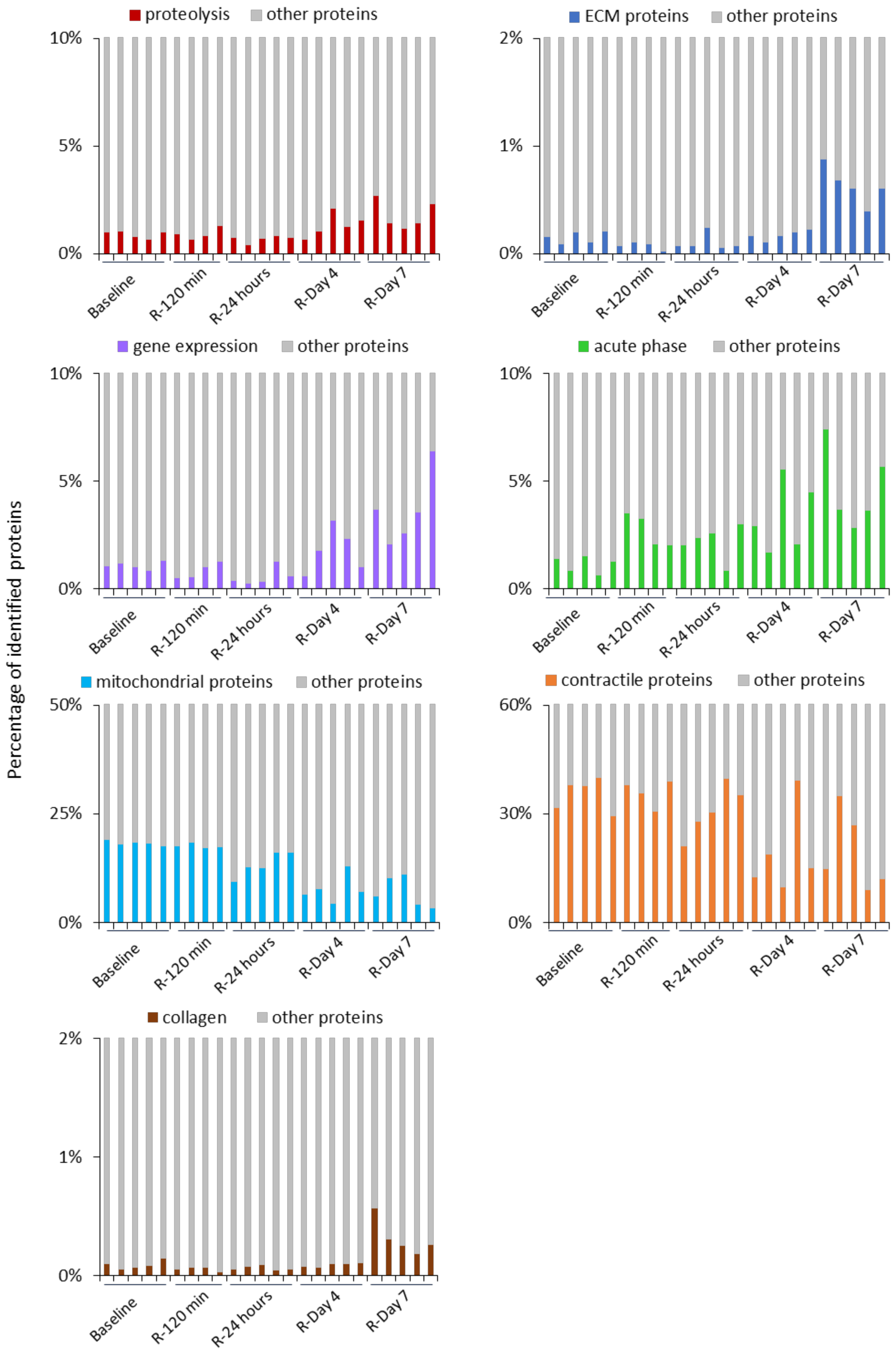

Percentage of identified proteins
proteolysis
other proteins
10%
5%
0%
ECM proteins
other proteins
2%
1%
0%
gene expression
other proteins
10%
5%
0%
acute phase
other proteins
10%
5%
0%
mitochondrial proteins
other proteins
50%
25%
0%
contractile proteins
other proteins
60%
30%
0%
collagen
other proteins
2%
1%
0%
Baseline
R-120 min
R-24 hours
R-Day 4
R-Day 7

Supplementary Figure S3. Functional categories representing sarcomere components, mitochondrial ribosomes, bioenergetics pathways, mitochondrial carriers and ETC complexes that were significantly altered (FDR < 0.05) in at least one time point within the remote myocardium.

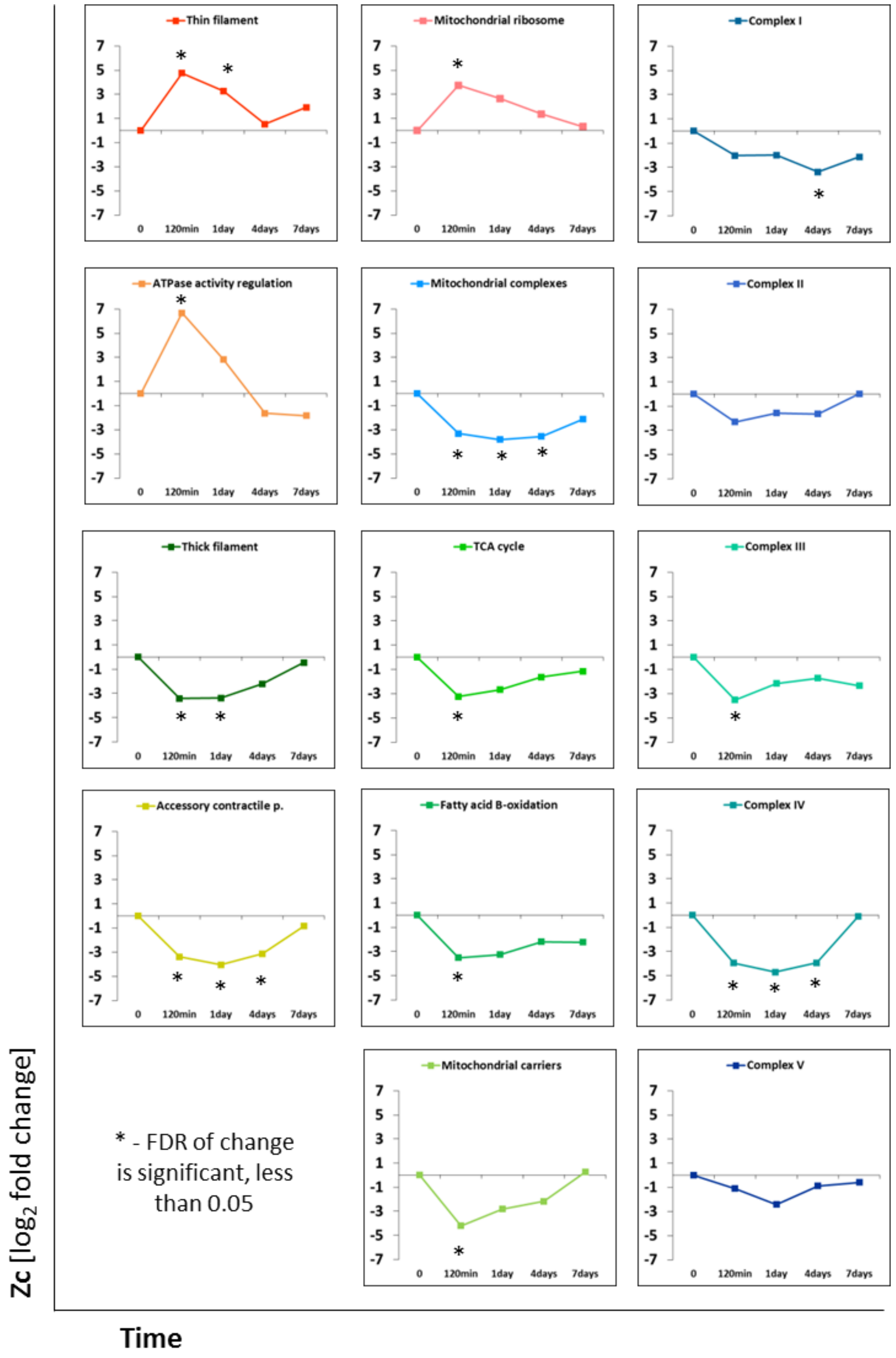

Supplementary Figure S4. Temporary contractile and mitochondrial dysfunction in the remote myocardium. Time course of protein expression changes (Zq) for the eight proteins quantified with the highest number of peptides (at least two) and belonging to altered categories related to sarcomere structure (left) and mitochondria (middle and right panels).

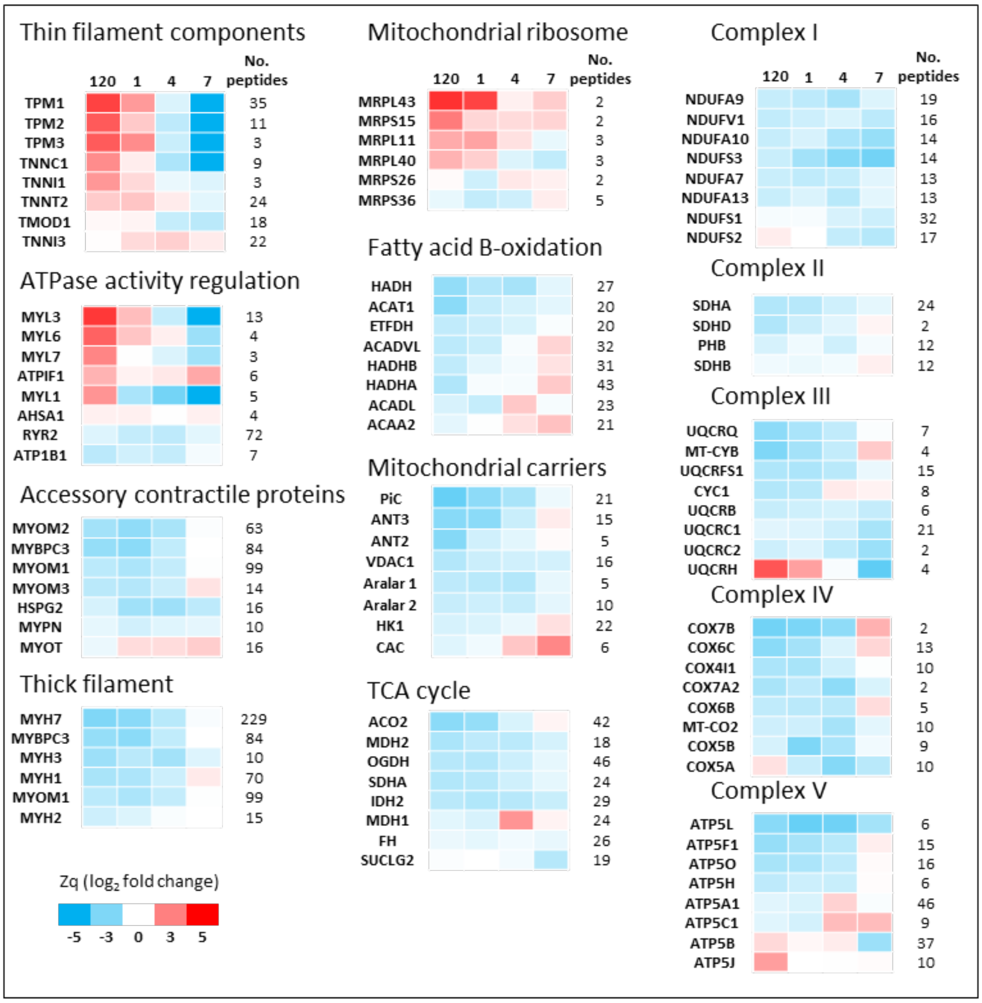

Supplementary Figure S5. Label-free experiments in the remote myocardium tissue. In iBAQ analysis, the normalized sum of protein values in sarcomeric structural proteins (thin filament and thick filament) is indicated for each biological individual (protein values are normalized by the sum of all intensities).

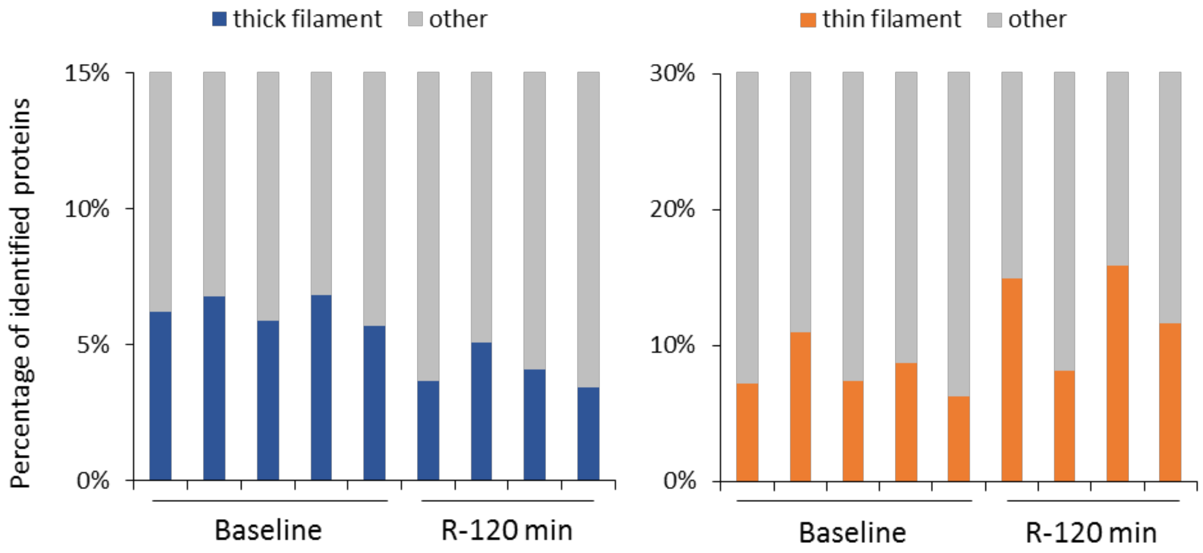

Supplementary Figure S6. Parallel reaction monitoring (PRM) validation experiments in remote myocardial tissue. Extracted ion chromatograms (XICs) of *b*- or *y*- fragment ions in a peptide sequence ALGQNPTQAEVLR from protein MYL3 fragments as an example of fragment peak area integration used for protein quantification generated by Skyline software. Fragments used for the quantification of this peptide: y11 - 1212.6331+; y9 - 1027.5531+; y8 - 913.5102+; y5 - 587.3511+; b2 - 185.1285+.

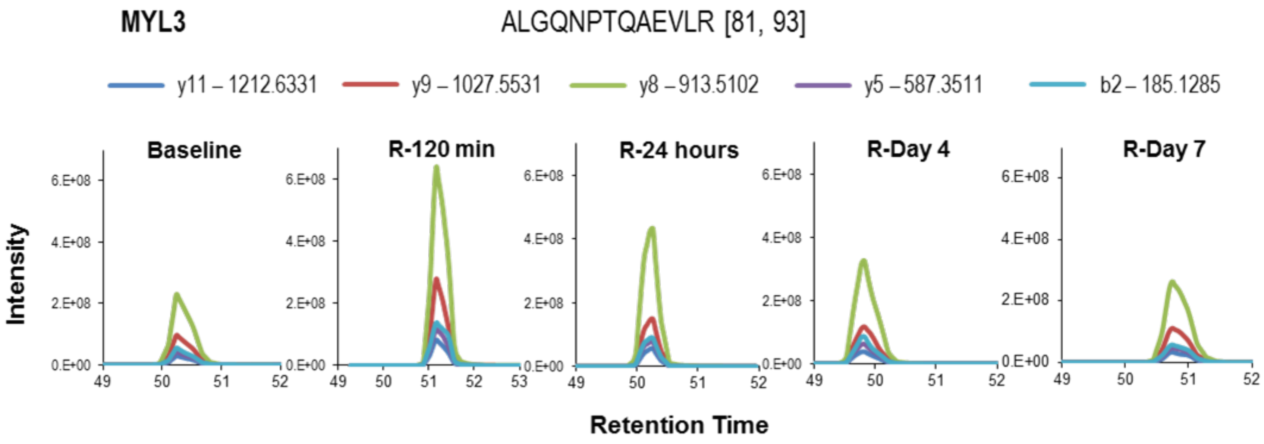

Supplementary Figure S7. Label-free experiments in the ischemic myocardium tissue. In iBAQ analysis the normalized sum of protein values in blood plasma proteins is indicated for each biological individual (protein values are normalized by the sum of all intensities).

	Baseline	R-120 min	R-24 hours	R-Day4	R-Day 7
% of plasma proteins	0.73%	2.11%	2.41%	3.30%	2.87%

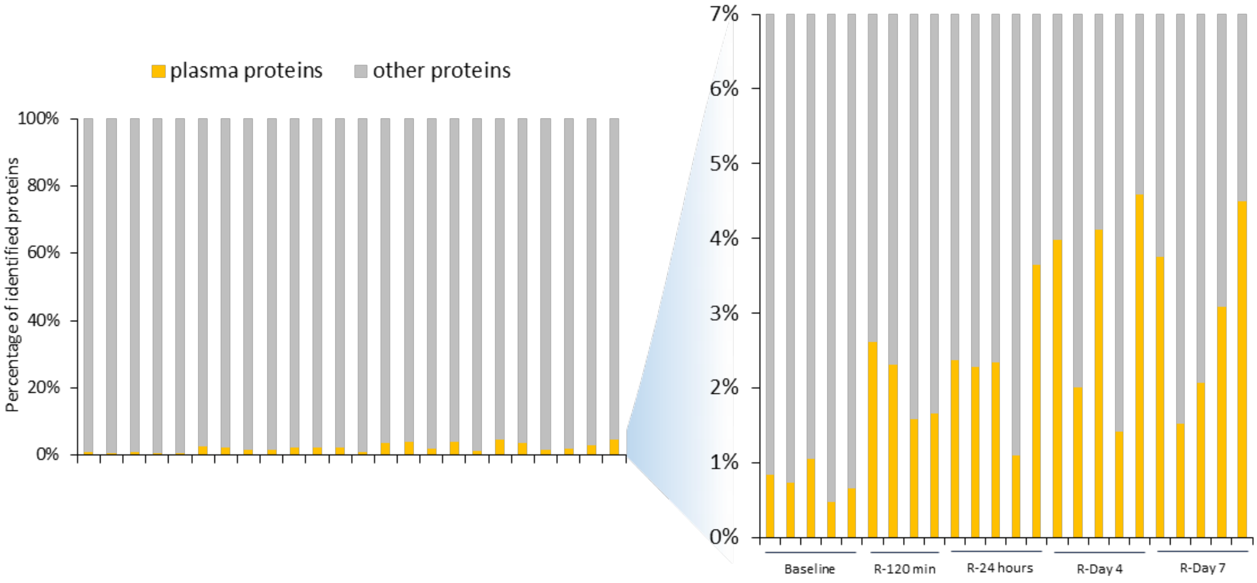

Supplementary Figure S8. Full-length blots corresponding to Fig.4a-b and Fig.6a in the main text. (a) Mitochondrial proteins (ATP5A, UQCRC2, SDHB, NDUFB8, UQCRFS1, COXIV and ATP5B) in the ischemic myocardium. (b) Contractile proteins (Myosin heavy chain, TNNT2 and TPM1) in the ischemic myocardium. (c) TPM1 in the remote myocardium.

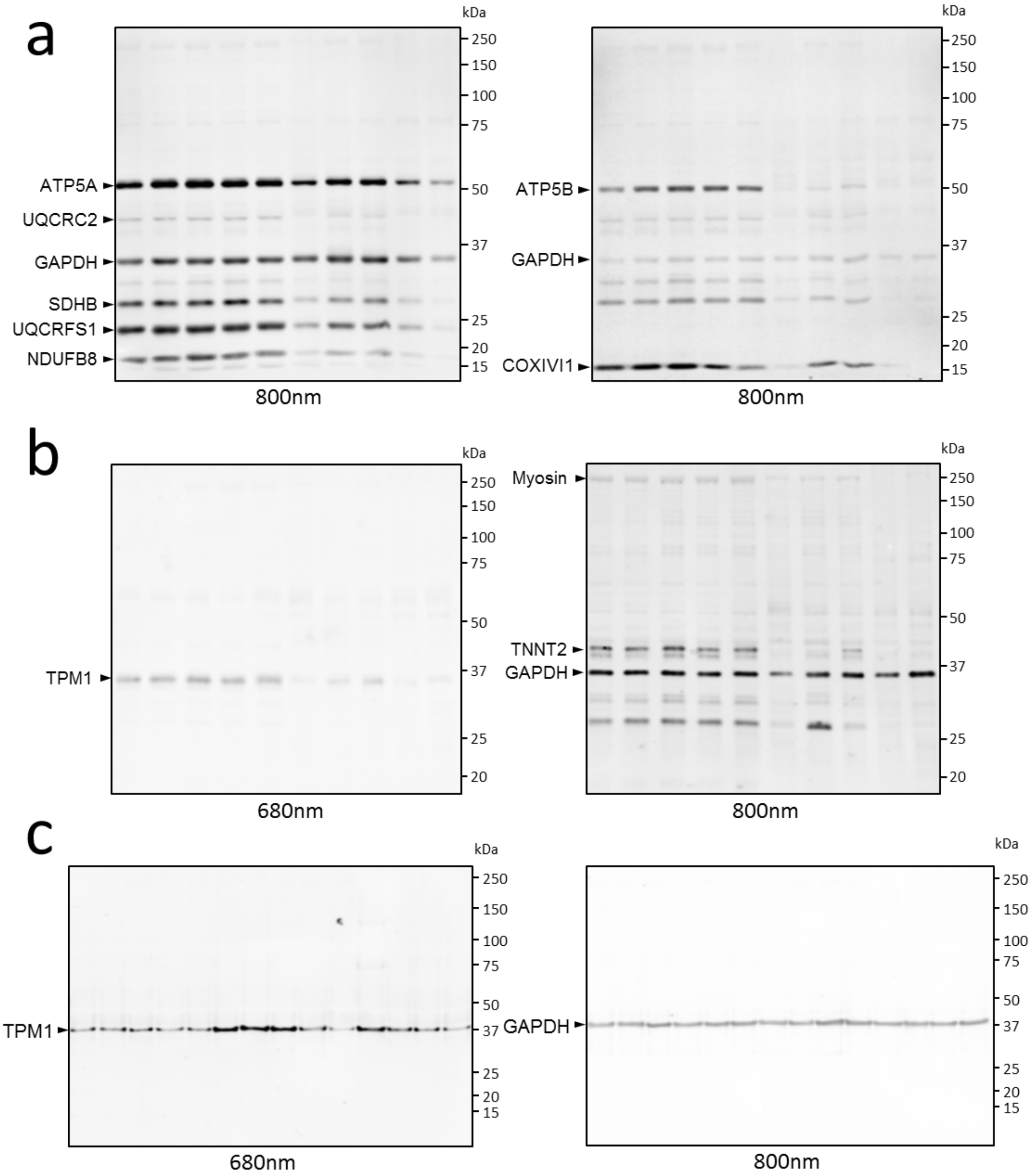

Supplementary Figure S9. Raw integrated density data before and after normalization by signal of the protein load GAPDH for each protein corresponding to WB from Figure 4a.

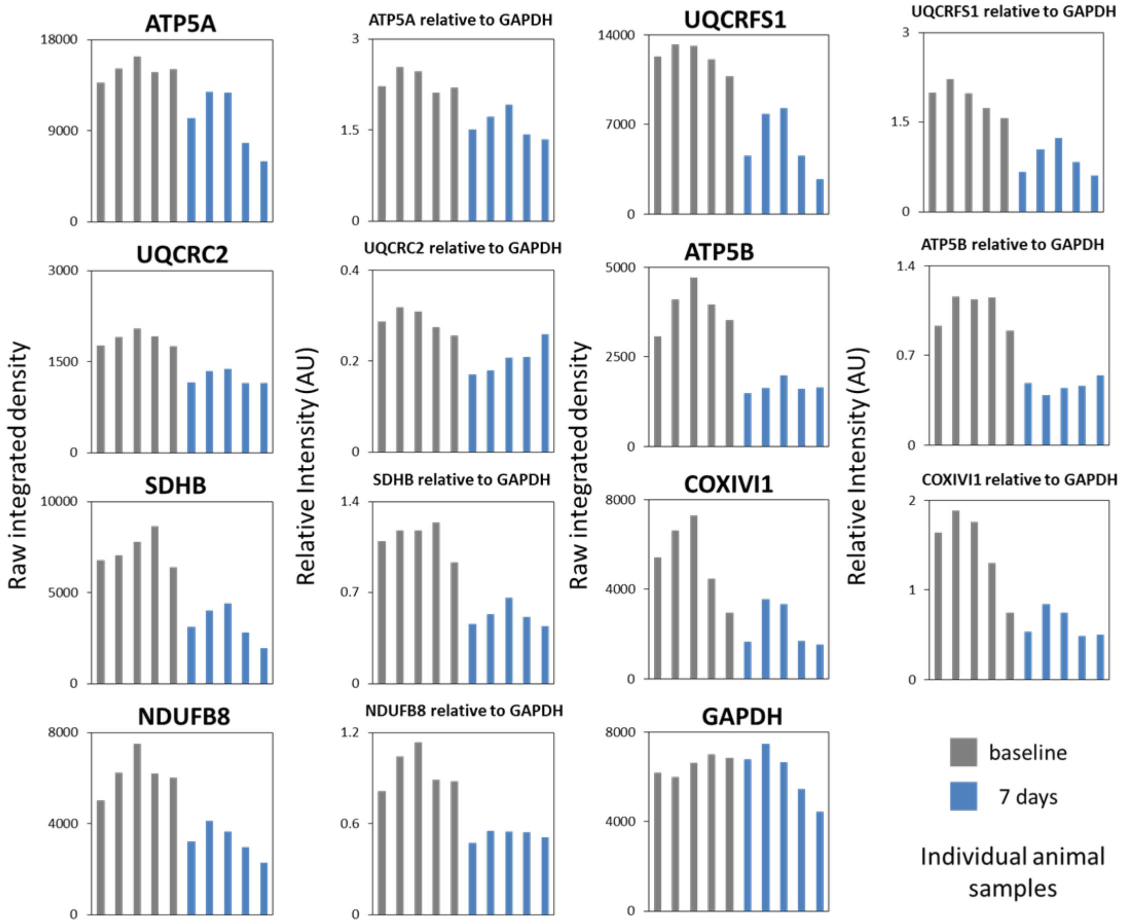

Supplementary Figure S10. Raw integrated density data before and after normalization by signal of the protein load GAPDH for each protein corresponding to WB from Figure 4b.

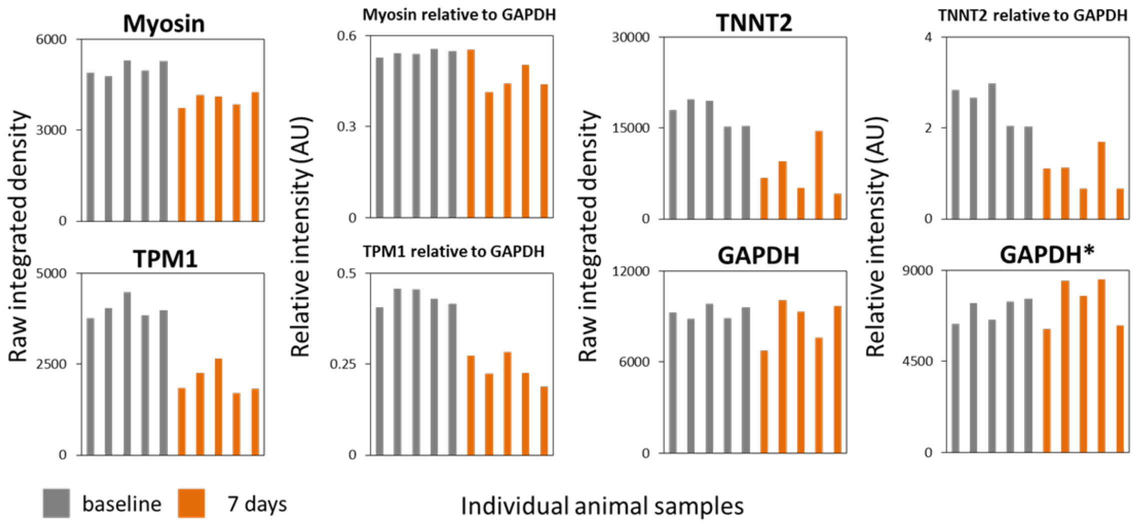

Supplementary Figure S11. Changes determined by isobaric labeling (iTRAQ) which contains (a) the same proteins validated by WB or label-free analysis presented in Figure 4 and (b) the same proteins validated by WB or PRM analysis presented in Figure 6 (b).

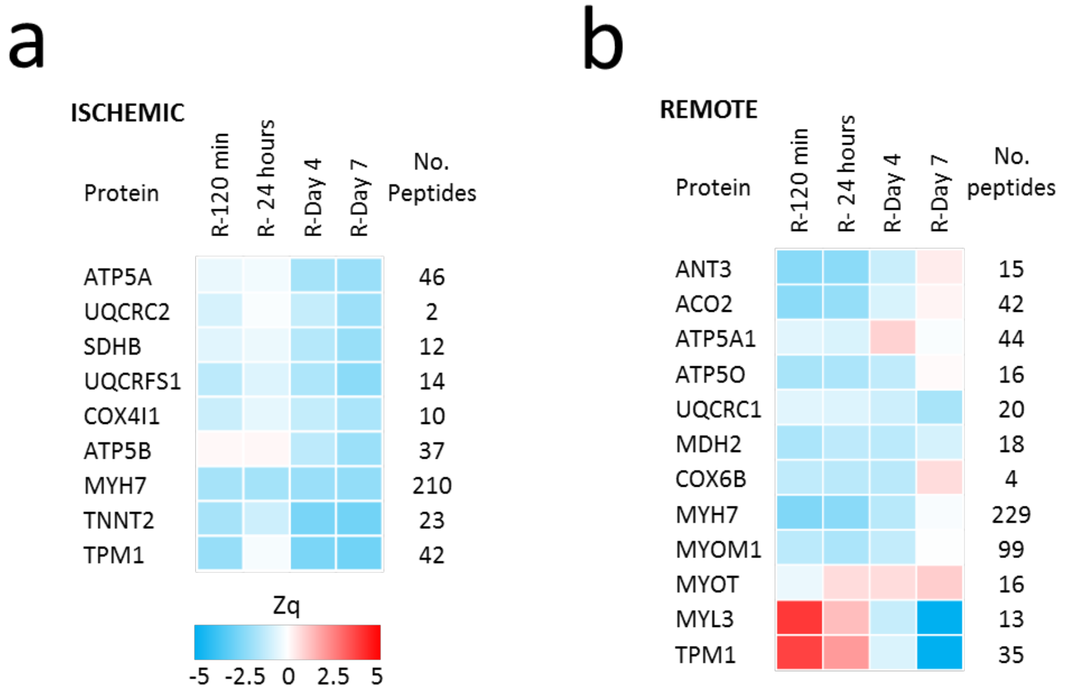

Supplementary Figure S12. Raw integrated density data before and after normalization by signal of the protein load GAPDH for each protein corresponding to WB from Figure 6a.

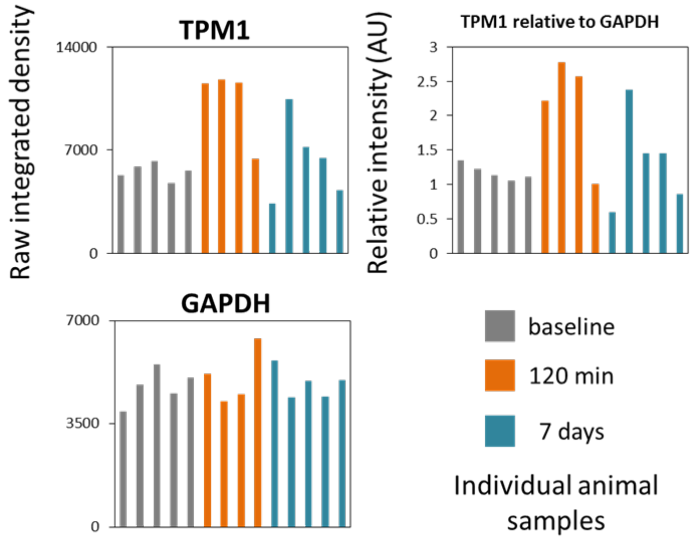

Supplementary Figure S13. Label-free analysis of individual proteins corresponding to (a) mitochondrial proteins, (b) contractile proteins, (c) acute phase, (d) collagen, (e) ECM, (f) proteolysis and (g) gene expression categories, represented in Figure 4d. Protein abundances are expressed as log2 (fold-change) of the iBAQ values of 7 days after reperfusion with respect to the baseline.

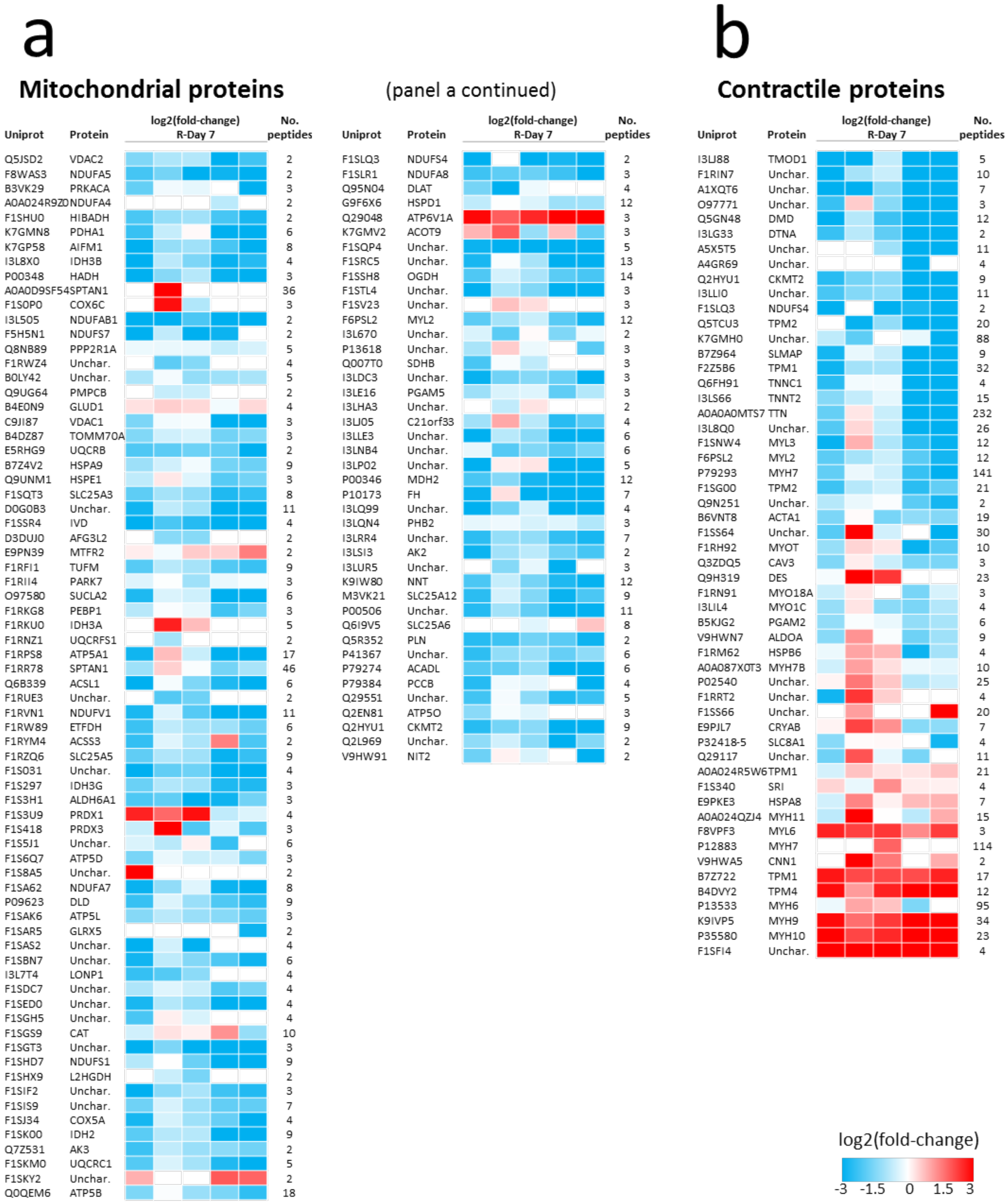

Supplementary Figure S13. (continued)

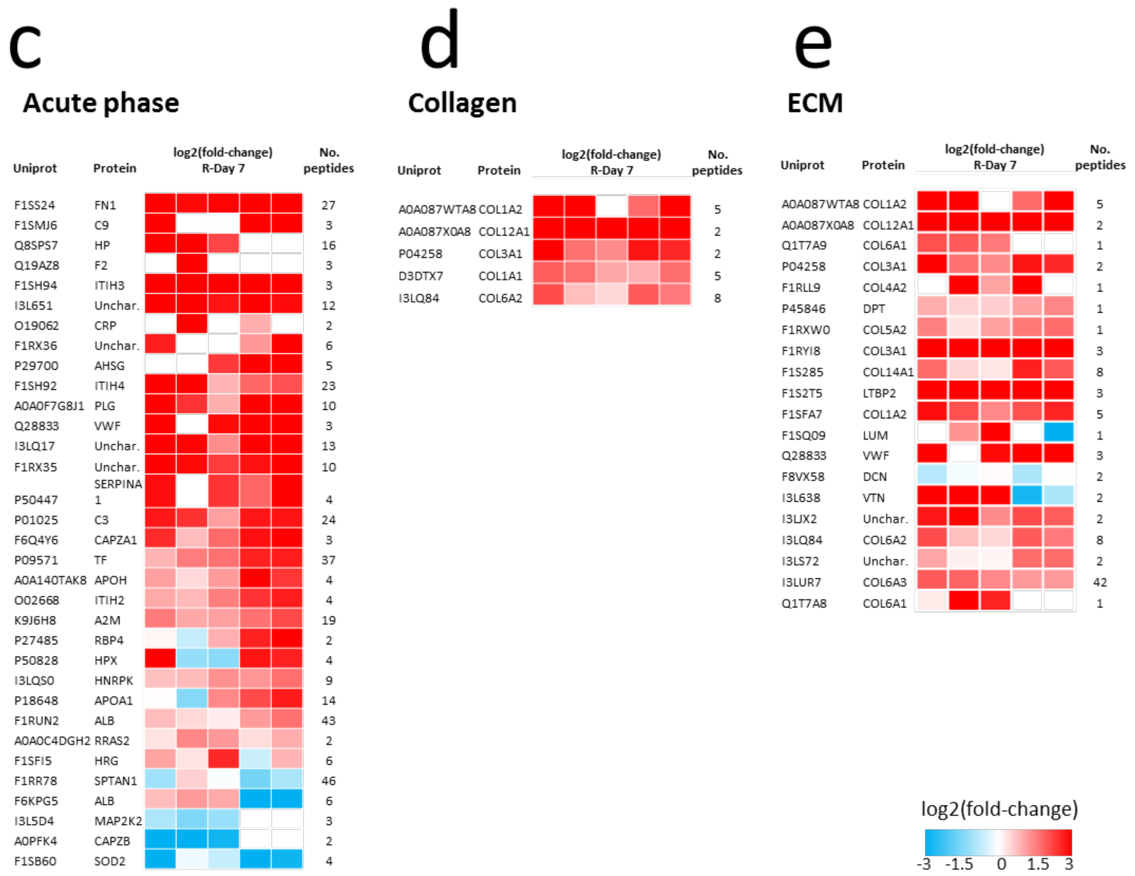

Supplementary Figure S13. (continued)

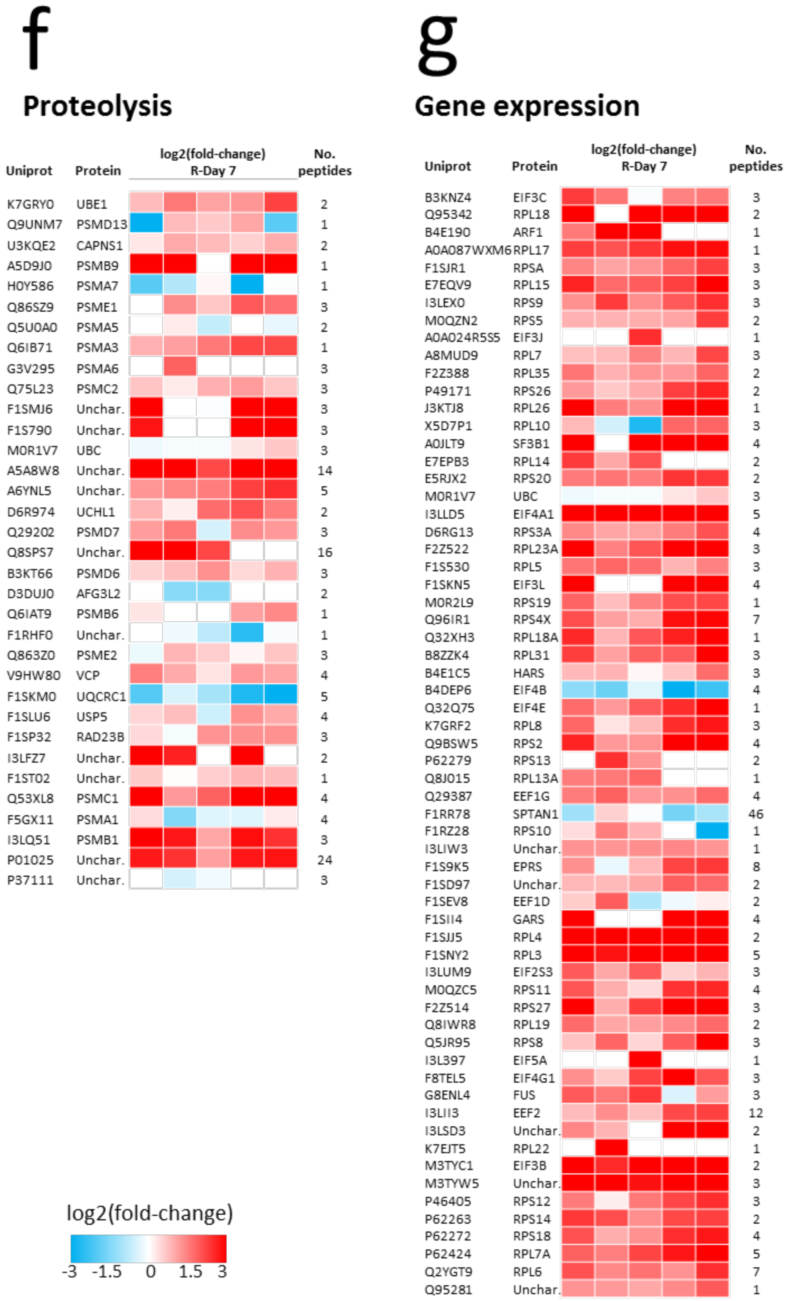

Supplementary Figure S14. Label-free analysis of individual proteins corresponding to (a) thin filament and (b) thick filament categories represented in Figure 6b. Protein abundances are expressed as log2 (fold-change) of the iBAQ values of 120 min after reperfusion with respect to the baseline.

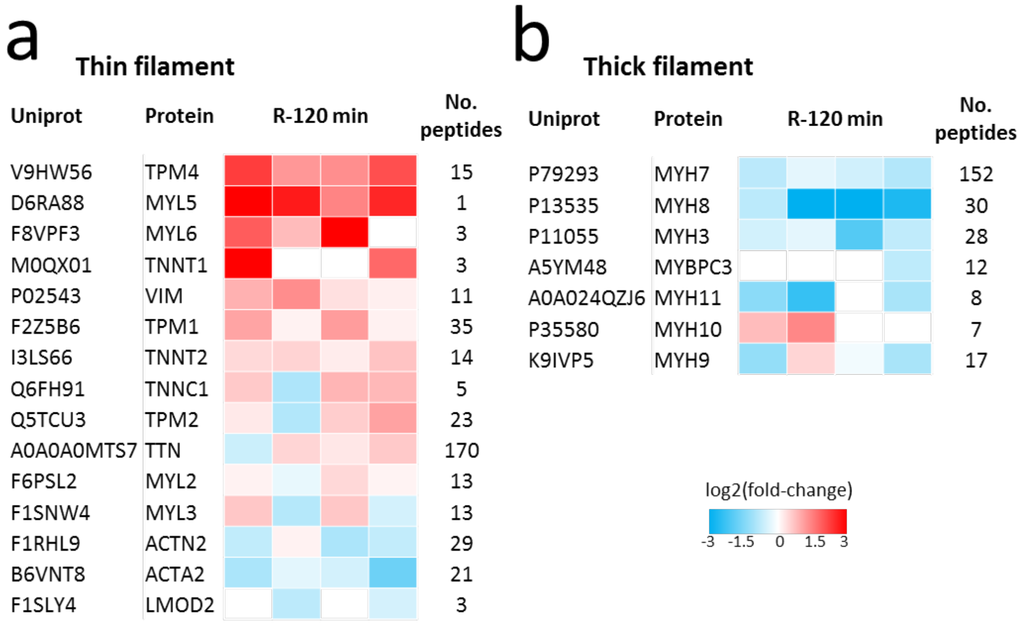

SUPPLEMENTARY TABLES

Supplementary Table S1. Protein expression changes in the functionally annotated categories within cluster a.

Human equivalent Ac. No.	Accession No.	Acute phase response signaling<br>Protein	Zq (log2 FC) 120	1	4	7	FDR 120	1	4	7	No. peptides
Q13233	F1SLK9	PIG Uncharacterized protein MAP3K1	3.134	7.697	6.058	2.267	0.09	0.00	0.00	0.55	1
-	I3LQR9	PIG Fibrinogen alpha chain	1.343	2.49	3.618	3.539	0.89	0.23	0.03	0.11	11
-	F1RX36	PIG Fibrinogen alpha chain	0.735	2.968	4.25	3.62	0.97	0.09	0.00	0.09	4
P02675	I3L651	PIG Uncharacterized protein	1.292	2.457	3.366	3.096	0.91	0.24	0.05	0.23	27
-	Q8SPS7	PIG Haptoglobin	3.055	3.505	4.406	2.82	0.11	0.02	0.00	0.36	14
P45985	F1SS51	PIG Uncharacterized protein (Fragment) MAP2K4	2.622	3.106	3.149	2.857	0.26	0.06	0.09	0.33	1
-	P00747	HUMAN Plasminogen	1.196	4.368	3.759	1.98	0.93	0.00	0.02	0.67	1
-	P18648	PIG Apolipoprotein A-I	2.421	2.571	1.779	1.218	0.36	0.19	0.64	0.86	34
P02679	F1RX35	PIG Uncharacterized protein LOC100627396	0.533	2.086	3.775	3.548	0.99	0.40	0.02	0.11	23
P04196	F1SFI5	PIG Uncharacterized protein HRG	2.263	3.28	1.95	1.42	0.44	0.04	0.55	0.82	12
-	P27485	PIG Retinol-binding protein 4	2.456	2.319	1.927	1.476	0.34	0.30	0.55	0.81	2
-	F1S0J2	PIG Uncharacterized protein C4BPA	2.001	2.623	2.88	2.022	0.58	0.17	0.15	0.65	4
-	P08835	PIG Serum albumin	3.428	2.943	2.28	1.742	0.04	0.09	0.39	0.75	27
-	P06867	PIG Plasminogen	1.073	2.63	3.175	1.432	0.94	0.17	0.08	0.82	13
-	O19063	PIG Serum amyloid P-component	1.188	2.679	3.137	1.279	0.93	0.16	0.09	0.85	6
-	O19062	PIG C-reactive protein	1.116	3.654	4.381	1.655	0.93	0.01	0.00	0.78	7
-	P00450	HUMAN Ceruloplasmin	2.511	1.725	2.332	2.631	0.32	0.61	0.36	0.41	1
-	F1SB81	PIG Plasminogen	0.204	2.644	2.932	2.36	0.99	0.17	0.14	0.52	2

-	Q19AZ8	PIG Prothrombin	2.411	2.994	1.957	1.057	0.36	0.08	0.54	0.90	6
-	I3L818	PIG Uncharacterized protein (Fragment) SERPINF2	1.263	1.742	2.977	2.046	0.91	0.59	0.12	0.65	4
P05546	F1RKY2	PIG Uncharacterized protein SERPIND1	2.37	2.011	1.585	1.229	0.38	0.44	0.72	0.86	9
P02749	I3LGN5	PIG Uncharacterized protein (Fragment)	1.686	2.42	2.192	1.502	0.75	0.25	0.44	0.81	1
-	P02768	HUMAN Serum albumin	1.769	2.264	1.765	0.632	0.71	0.32	0.65	0.96	3
Q06033	F1SH94	PIG Uncharacterized protein ITIH3	1.577	2.157	2.131	1.644	0.79	0.37	0.46	0.78	14
-	P50390	PIG Transthyretin	2.233	1.786	1.388	0.951	0.45	0.57	0.80	0.92	7
-	F1SFI7	PIG Alpha-2-HS-glycoprotein (Fragment)	1.864	1.838	1.492	1.673	0.65	0.54	0.75	0.77	1
-	P01025	PIG Complement C3	2.501	1.593	1.876	1.685	0.32	0.67	0.58	0.77	83
-	P09571	PIG Serotransferrin	3.225	2.269	1.463	1.179	0.07	0.32	0.76	0.87	33
P01023	I3LQ17	PIG Uncharacterized protein	2.016	1.788	2.079	1.597	0.57	0.57	0.48	0.80	50
P02748	F1SMJ6	PIG Uncharacterized protein (Fragment) C9	0.764	1.829	2.012	1.352	0.97	0.55	0.51	0.83	7
-	F1S1A9	PIG Uncharacterized protein APOA2	1.175	1.666	1.865	1.51	0.92	0.64	0.59	0.81	3
P01024	I3LTB8	PIG Uncharacterized protein	1.888	1.799	1.664	1.131	0.63	0.56	0.69	0.88	2
-	P79263	PIG Inter-alpha-trypsin inhibitor heavy chain H4	1.126	2.505	1.926	3.001	0.93	0.22	0.55	0.26	39
P02751	F1SS24	PIG Uncharacterized protein FN1	0.527	0.684	1.731	2.499	0.99	0.99	0.67	0.46	63
-	Q03710	PIG Complement factor B (Fragment)	0.958	1.507	1.723	2.054	0.95	0.72	0.67	0.64	1
-	P50447	PIG Alpha-1-antitrypsin	2.605	2.139	1.561	1.19	0.27	0.38	0.73	0.87	12
-	O02668	PIG Inter-alpha-trypsin inhibitor heavy chain H2	1.779	1.37	1.427	1.103	0.70	0.80	0.78	0.89	23
-	Q0PM28	PIG Pigment epithelium-derived factor	1.395	1.762	1.394	1.491	0.87	0.58	0.80	0.81	13
-	P29700	PIG Alpha-2-HS-glycoprotein (Fragment)	2.106	1.589	1.142	1.059	0.51	0.68	0.90	0.90	4
-	P50828	PIG Hemopexin	2.261	2.028	1.929	1.59	0.44	0.44	0.55	0.80	11
-	A0PFK4	PIG F-actin capping protein subunit beta 2	0.393	0.516	1.712	2.277	0.99	1.00	0.67	0.54	1
-	F1SH92	PIG Inter-alpha-trypsin inhibitor heavy chain H4	1.076	2.146	1.711	2.697	0.94	0.38	0.67	0.40	6
-	P14477	PIG Fibrinogen beta chain (Fragment)	-0.18	1.518	1.03	1.911	0.99	0.72	0.92	0.69	1
-	Q6VPV1	PIG Complement component C5	1.127	1.588	1.7	1.062	0.93	0.68	0.67	0.90	20
-	H0YA55	HUMAN Serum albumin (Fragment)	2.347	2.004	1.349	1.456	0.39	0.45	0.82	0.82	1

-	A0PFK5	PIG Capping protein (Actin filament) muscle Z-line, alpha 1	0.62	0.74	1.517	1.459	0.98	0.99	0.75	0.82	6
-	K9J6H8	PIG Alpha-2-macroglobulin	1.728	1.975	1.262	-0.11	0.73	0.47	0.85	1.00	3
-	I3LPQ9	PIG Uncharacterized protein (Fragment) TRADD	1.369	1.023	0.517	-0.19	0.88	0.93	0.99	0.99	1
-	G3V387	HUMAN Short peptide from AAT (Fragment)	1.437	1.245	0.788	0.556	0.85	0.86	0.96	0.98	1
-	F1S0J3	PIG Uncharacterized protein C4BPB	-0.28	1.227	2.281	1.471	0.99	0.86	0.39	0.81	1
-	H9LBP0	PIG Serpin peptidase inhibitor clade D member 1 (Fragment)	0.678	1.787	1.933	0.65	0.98	0.57	0.55	0.96	2
-	F1S8T1	PIG Uncharacterized protein NOLC1	-0.16	0.42	0.606	0.905	0.99	1.01	0.98	0.92	1
-	P32394	PIG Heme oxygenase 1	0.774	0.663	1.096	1.001	0.97	0.99	0.90	0.92	7
-	P19133	PIG Ferritin light chain (Fragment)	0.082	0.413	0.555	1.553	1.00	1.01	0.98	0.80	3
-	Q28833	PIG von Willebrand factor (Fragment)	0.779	1.131	0.987	0.846	0.97	0.90	0.92	0.94	7
-	P02751	HUMAN Fibronectin	0.889	0.355	0.789	0.961	0.96	1.01	0.96	0.92	5
-	P02675	HUMAN Fibrinogen beta chain	3.779	0.632	-0.02	-0.17	0.02	0.99	1.00	0.99	2
-	Q07890	HUMAN Son of sevenless homolog 2	0.75	0.816	1.022	0.593	0.97	0.98	0.92	0.97	2
-	Q2MJK3	PIG GTPase NRas	2.217	2.09	0.313	0.064	0.46	0.40	1.00	1.00	1
-	P79335	PIG Plasminogen activator inhibitor 1	1.745	1.24	0.6	0.308	0.72	0.86	0.98	0.99	2
-	P14460	PIG Fibrinogen alpha chain (Fragment)	0.285	0.985	0.488	0.584	0.99	0.94	0.99	0.97	2
Q16539	F1RYA1	PIG Uncharacterized protein MAPK14	0.409	0.406	1.014	0.657	0.99	1.01	0.92	0.96	2
-	P01100	HUMAN Proto-oncogene c-Fos	0.723	0.362	0.213	0.692	0.97	1.01	1.00	0.96	1
-	F1RUN2	PIG Serum albumin	1.327	0.551	0.491	0.512	0.90	0.99	0.99	0.98	2
-	Q19S50	PIG Signal transducer and activator of transcription 3	0.291	-0.11	0.77	1.196	1.00	1.00	0.97	0.87	3
-	F1SA28	PIG Uncharacterized protein ECSIT	0.98	0.714	0.955	0.895	0.96	0.99	0.93	0.93	1
P27361	I3LVK2	PIG Uncharacterized protein (Fragment) MAPK3	0.585	0.463	0.676	0.198	0.98	1.00	0.97	0.99	2
-	P00736	HUMAN Complement C1r subcomponent	-0.56	0.502	0.99	0.49	0.98	1.00	0.92	0.98	1
Q15750	K7GLB8	PIG Uncharacterized protein TAB1	1.986	0.997	-0.01	0.181	0.59	0.94	1.00	0.99	1
-	P40189	HUMAN Interleukin-6 receptor subunit beta	1.899	0.249	-0.9	-1.82	0.63	1.00	0.94	0.73	1
-	A7XNS1	PIG Nuclear factor of kappa light polypeptide enhancer in B-cells 1	1.576	1.025	0.599	0.161	0.79	0.93	0.98	1.00	2
Q9Y243	K7GNL2	PIG Uncharacterized protein (Fragment) AKT3	0.696	0.681	-0.28	-0.15	0.98	0.99	1.00	1.00	1

-	A7XUJ6	PIG TNF receptor-associated factor 6	0.197	-0.65	0.55	0.873	0.99	0.99	0.98	0.93	1
P10301	F1RHW4	PIG Uncharacterized protein RRAS	0.288	0.066	0.065	0.071	1.00	1.00	1.00	1.00	5
-	P62993	HUMAN Growth factor receptor-bound protein 2	-0.41	-0.57	0.253	0.693	0.99	0.99	1.00	0.96	3
-	O97506	PIG Kallikrein	0.582	-0.7	-0.27	0.079	0.98	0.99	1.00	1.00	4
-	P04366	PIG Protein AMBP (Fragment)	0.727	0.555	0.094	-0.52	0.97	0.99	1.00	0.98	3
-	M3TYR4	PIG Mitogen-activated protein kinase kinase 3	0.085	-0.57	-0.24	-0.16	1.00	0.99	1.00	1.00	2
P61978	I3LQS0	PIG Uncharacterized protein HNRNPK	-0.99	-0.61	0.283	0.793	0.95	0.99	1.00	0.95	11
P36507	I3L5D4	PIG Uncharacterized protein MAP2K2	-0.24	-0.44	-0.05	0.155	0.99	1.00	1.00	1.00	4
-	P19823	HUMAN Inter-alpha-trypsin inhibitor heavy chain H2	-0.13	-1.14	-0.45	-0.65	0.99	0.89	0.99	0.96	1
-	Q7YRV6	PIG Suppressor of cytokine signaling 2	-0.37	-0.75	-0.28	0.012	1.00	0.99	1.00	1.00	1
-	M3V839	PIG Mitogen-activated protein kinase 3	-0.25	-0.92	-0.64	0.221	0.99	0.96	0.98	0.99	3
-	P61978	HUMAN Heterogeneous nuclear ribonucleoprotein K	-0.63	-0.69	-0.57	-0.03	0.98	0.99	0.98	1.00	4
Q02750	I3LQS6	PIG Uncharacterized protein (Fragment) MAP2K1	-0.74	-0.93	-0.17	0.036	0.97	0.96	1.00	1.00	3
-	P04150	HUMAN Glucocorticoid receptor	-0.14	-0.51	-0.55	-0.64	0.99	1.00	0.98	0.96	1
-	A9QT41	PIG Inhibitor of kappa light polypeptide gene enhancer in B-cells kinase gamma	0.087	0.011	-0.2	-0.54	1.00	1.00	1.00	0.97	2
Q07890	F1SHY0	PIG Uncharacterized protein (Fragment) SOS2	-1.17	-0.22	-0.51	-0.48	0.93	1.00	0.99	0.98	1
P02549	F1RR78	PIG Uncharacterized protein LOC100049693	-0.89	-1.12	-0.83	-0.49	0.96	0.90	0.95	0.98	148
-	P04179	HUMAN Superoxide dismutase [Mn], mitochondrial	-1.71	-0.66	-1.12	-0.92	0.74	0.99	0.90	0.92	1
-	P02792	HUMAN Ferritin light chain	-0.7	-0.91	-1.29	-1.74	0.98	0.96	0.84	0.75	1
-	P62070	HUMAN Ras-related protein R-Ras2	-0.49	0.124	-0.52	-0.82	0.99	1.00	0.99	0.94	1
-	G9BWQ1	PIG V-akt murine thymoma viral oncogene-like 1	-0.4	-0.74	-1.18	-1.34	0.99	0.99	0.88	0.83	1
-	F1RWY6	PIG Uncharacterized protein RIPK1	-1.12	-1.4	-1.69	-1.17	0.93	0.78	0.68	0.87	1
-	Q9NPH3	HUMAN Interleukin-1 receptor accessory protein	-1.28	-1.31	-1.2	-1.35	0.91	0.83	0.87	0.83	1
-	P15884	HUMAN Transcription factor 4	-1.01	-1.18	-1.46	-1.42	0.96	0.88	0.76	0.82	1
P30519	F1RK58	PIG Uncharacterized protein HMOX2	-1.18	-1.39	-1.59	-1.24	0.93	0.79	0.71	0.85	2
-	Q07889	HUMAN Son of sevenless homolog 1	-0.57	-1.03	-1.02	-2.04	0.98	0.94	0.92	0.65	1
-	Q2TAM5	HUMAN RELA protein	-1.14	-1.48	-1.18	-0.46	0.93	0.73	0.88	0.99	1

-	H0YCB4	HUMAN Transcription factor p65 (Fragment)	-1.13	-1.83	-2.34	-1.88	0.93	0.54	0.35	0.70	1
-	P28768	PIG Superoxide dismutase [Mn], mitochondrial (Fragment)	-1.42	-0.19	-1.84	-2.26	0.86	1.00	0.60	0.55	7

Response to wounding

Human equivalent Ac. No.	Accession No.	Protein	Zq (log2 FC) 120	1	4	7	FDR 120	1	4	7	No. peptides
-	A5D9N3	PIG Allograft inflammatory factor 1	0.716	2.363	4.097	4.459	0.97	0.27	0.01	0.01	1
-	P19619	PIG Annexin A1	0.759	3.181	3.067	2.43	0.97	0.05	0.11	0.49	16
-	Q29549	PIG Clusterin	2.275	3.807	3.763	1.741	0.43	0.01	0.02	0.75	28
-	F1S0J2	PIG Uncharacterized protein C4BPA	2.001	2.623	2.88	2.022	0.58	0.17	0.15	0.65	4
-	O19063	PIG Serum amyloid P-component	1.188	2.679	3.137	1.279	0.93	0.16	0.09	0.85	6
-	O19062	PIG C-reactive protein	1.116	3.654	4.381	1.655	0.93	0.01	0.00	0.78	7
-	F1STZ3	PIG Uncharacterized protein C1QC	2.553	2.481	2.407	1.545	0.30	0.23	0.32	0.80	1
-	Q69DL0	PIG Complement C1q subcomponent subunit A	0.889	2.606	3.095	1.688	0.96	0.18	0.10	0.76	4
-	F1SFI4	PIG Uncharacterized protein KNG1	2.032	1.954	2.013	1.727	0.57	0.48	0.51	0.75	12
-	F1SFI7	PIG Alpha-2-HS-glycoprotein (Fragment)	1.864	1.838	1.492	1.673	0.65	0.54	0.75	0.77	1
-	P01025	PIG Complement C3	2.501	1.593	1.876	1.685	0.32	0.67	0.58	0.77	83
P01023	I3LQ17	PIG Uncharacterized protein	2.016	1.788	2.079	1.597	0.57	0.57	0.48	0.80	50
P02748	F1SMJ6	PIG Uncharacterized protein (Fragment) C9	0.764	1.829	2.012	1.352	0.97	0.55	0.51	0.83	7
-	F1SGT4	PIG Uncharacterized protein CD44	1.007	2.072	2.186	1.712	0.96	0.41	0.44	0.75	3
-	F1S1A9	PIG Uncharacterized protein APOA2	1.175	1.666	1.865	1.51	0.92	0.64	0.59	0.81	3
P01024	I3LTB8	PIG Uncharacterized protein	1.888	1.799	1.664	1.131	0.63	0.56	0.69	0.88	2
-	P79263	PIG Inter-alpha-trypsin inhibitor heavy chain H4	1.126	2.505	1.926	3.001	0.93	0.22	0.55	0.26	39
-	A2SW51	PIG Monocyte differentiation antigen CD14	1.601	2.47	1.714	1.165	0.78	0.23	0.67	0.87	2
-	Q03710	PIG Complement factor B (Fragment)	0.958	1.507	1.723	2.054	0.95	0.72	0.67	0.64	1
-	P29700	PIG Alpha-2-HS-glycoprotein (Fragment)	2.106	1.589	1.142	1.059	0.51	0.68	0.90	0.90	4
-	Q9TUQ3	PIG Complement component C7	1.658	1.551	1.261	0.905	0.76	0.71	0.85	0.92	7

-	F1S790	PIG Uncharacterized protein C8B	1.473	1.677	1.526	0.922	0.84	0.64	0.74	0.92	3
-	A5A767	PIG Adaptor-related protein complex 3, beta-1 subunit	1.363	1.44	0.671	0.057	0.89	0.76	0.97	1.00	1
-	F1S643	PIG Uncharacterized protein CFH	1.275	1.343	1.466	1.189	0.91	0.81	0.76	0.87	9
-	P16050	HUMAN Arachidonate 15-lipoxygenase	1.443	1.154	1.548	1.104	0.85	0.89	0.73	0.89	1
-	F1SH92	PIG Inter-alpha-trypsin inhibitor heavy chain H4	1.076	2.146	1.711	2.697	0.94	0.38	0.67	0.40	6
-	B0LFE9	PIG Complement component 4	1.503	1.43	1.345	1.384	0.83	0.76	0.82	0.83	38
-	P01042	HUMAN Kininogen-1	3.166	2.613	0.966	0.818	0.09	0.18	0.93	0.94	1
-	Q8WMQ3	PIG CD9 antigen	0.139	1.238	2.075	1.81	0.99	0.86	0.48	0.73	1
-	Q6VPV1	PIG Complement component C5	1.127	1.588	1.7	1.062	0.93	0.68	0.67	0.90	20
-	A0SEH3	PIG Complement component C8G	1.471	1.551	1.249	0.718	0.84	0.71	0.85	0.96	5
-	Q69DK8	PIG Complement C1s subcomponent	1.744	1.63	0.966	0.578	0.72	0.66	0.93	0.97	4
-	P53714	PIG Integrin beta-2	1.232	0.766	1.018	1.091	0.91	0.99	0.92	0.89	6
-	F1SMJ1	PIG Complement component C7 (Fragment)	0.624	1.35	1.158	1.129	0.99	0.81	0.89	0.88	1
-	P51779	PIG Complement factor D	1.691	1.866	1.327	0.896	0.75	0.52	0.83	0.93	1
-	K9J6H8	PIG Alpha-2-macroglobulin	1.728	1.975	1.262	-0.11	0.73	0.47	0.85	1.00	3
-	F1SMI8	PIG Uncharacterized protein C6	0.829	1.231	1.636	1.397	0.96	0.86	0.70	0.82	2
-	A8DSD5	PIG Integrin beta	0.901	0.853	1.091	1.131	0.96	0.98	0.90	0.88	1
-	A1E295	PIG Cathepsin B	0.227	0.213	1.518	2.323	1.00	1.00	0.75	0.52	6
-	F1S0J3	PIG Uncharacterized protein C4BPB	-0.28	1.227	2.281	1.471	0.99	0.86	0.39	0.81	1
-	F1SCY4	PIG Lipase	0.648	1.198	1.039	1.48	0.98	0.87	0.92	0.81	2
-	P08758	HUMAN Annexin A5	-0.43	0.913	1.156	1.574	0.99	0.96	0.89	0.80	3
-	P0C0L4	HUMAN Complement C4-A	-0.26	0.046	0.268	1.351	0.99	1.00	1.00	0.83	1
-	P52649	PIG Cytochrome b-245 heavy chain (Fragment)	0.899	1.093	0.834	0.424	0.96	0.91	0.95	0.99	3
-	A8DNU5	PIG Integrin alpha-L	1.129	1.135	0.806	0.609	0.93	0.90	0.96	0.97	2
-	F1STZ1	PIG Uncharacterized protein C1QB	-0.17	0.685	1.641	0.911	0.99	0.99	0.69	0.92	3
P02461	F1RYI8	PIG Uncharacterized protein COL3A1	-0	0.042	1.021	1.81	1.00	1.00	0.92	0.73	7
P20908	F1S021	PIG Uncharacterized protein (Fragment) COL5A1	-0.29	0.554	1.014	1.412	1.00	0.99	0.91	0.82	5

-	P04264	HUMAN Keratin, type II cytoskeletal 1	0.218	0.721	-0.2	-0.19	0.99	0.99	1.00	0.99	20
-	P33076	HUMAN MHC class II transactivator	-0.67	0.343	1.249	1.991	0.98	1.01	0.85	0.67	1
-	F2Z5C1	PIG Annexin	0.034	0.464	0.801	1.337	1.00	1.00	0.96	0.83	19
-	F1S1G8	PIG Amine oxidase	-0	0.502	0.547	0.493	1.00	1.00	0.98	0.98	9
-	P02461	HUMAN Collagen alpha-1(III) chain	-1.32	0.97	0.332	1.023	0.90	0.95	0.99	0.91	2
P04264	F1SGG3	PIG Uncharacterized protein KRT1	0.476	0.536	-0.37	-0.11	0.99	0.99	0.99	1.00	7
-	F1SBN9	PIG Uncharacterized protein ADORA3	0.671	0.235	0.997	0.764	0.98	1.00	0.92	0.95	1
-	A0SEH0	PIG Complement component C6	0.592	0.5	0.728	0.5	0.99	1.00	0.97	0.98	7
-	P00736	HUMAN Complement C1r subcomponent	-0.56	0.502	0.99	0.49	0.98	1.00	0.92	0.98	1
P49257	F1SMS8	PIG Uncharacterized protein LMAN1	0.099	0.197	0.561	0.644	1.00	1.00	0.98	0.96	7
-	P16469	PIG Arachidonate 15-lipoxygenase	1.269	1.138	0.615	-0.18	0.91	0.89	0.98	0.99	4
-	Q767L0	PIG ATP-binding cassette sub-family F member 1	-0.18	-0.33	0.205	0.495	0.99	1.00	1.00	0.98	3
-	F1RW73	PIG Uncharacterized protein BMP6	0.796	-0.07	0.367	0.204	0.96	1.00	1.00	0.99	1
-	P81076	PIG Allograft inflammatory factor 1	0.136	-0.1	0.345	0.919	0.99	1.00	0.99	0.92	2
-	Q2VL90	PIG Scavenger receptor cysteine-rich type 1 protein M130	-0.23	-0.35	0.27	0.563	1.00	1.01	1.00	0.97	4
P07357	F1S788	PIG Uncharacterized protein (Fragment) C8A	0.717	0.082	0.21	0.226	0.97	1.00	1.00	0.99	5
-	O75882	HUMAN Attractin	0.276	-0.36	-0.27	-0.75	0.99	1.01	1.00	0.96	1
-	O97506	PIG Kallikrein	0.582	-0.7	-0.27	0.079	0.98	0.99	1.00	1.00	4
-	Q95N02	PIG Cysteinyl leukotriene receptor 1	0.124	0.323	-0.54	-0.79	0.99	1.00	0.98	0.95	1
-	A5A774	PIG Hermansky-Pudlak syndrome 5 protein	0.082	0.574	-1.03	-0.74	1.00	0.99	0.92	0.96	1
-	B3STX8	PIG Thrombomodulin	-0.98	0.127	0.581	0.711	0.96	1.00	0.98	0.96	1
P55268	F1SPT5	PIG Uncharacterized protein LAMB2	-0.01	-0.19	-0.3	-0.37	1.00	1.00	1.00	0.99	25
-	P09917	HUMAN Arachidonate 5-lipoxygenase	0.322	0.326	-0.37	-0.91	1.00	1.00	0.99	0.92	1
-	P15924	HUMAN Desmoplakin	1.216	-0.12	-0.84	-0.82	0.92	1.00	0.95	0.94	2
-	K7GL02	PIG ATP-binding cassette sub-family F member 1	-0.99	-1.19	-0.74	0.742	0.96	0.88	0.97	0.96	1
Q9C0E8	F1RZH8	PIG Uncharacterized protein KIAA1715	0.099	-0.9	-0.49	-0.03	1.00	0.97	0.99	1.00	1
-	Q197W4	PIG Cyclin-dependent kinase 5	-0.85	-1.02	-0.62	-0.04	0.96	0.94	0.98	1.00	1

-	P20908	HUMAN Collagen alpha-1(V) chain	-1.42	-0.7	-0.56	0.256	0.86	0.99	0.98	0.99	1
-	P55268	HUMAN Laminin subunit beta-2	-0.84	-0.83	-0.69	-0.61	0.96	0.98	0.97	0.97	5
Q16352	F1S847	PIG Uncharacterized protein INA	-0.85	-1.24	-0.46	-0.48	0.96	0.86	0.99	0.98	1
-	Q3HUX1	PIG Fatty acid translocase/CD36	-0.69	-0.18	-0.77	-0.95	0.98	1.00	0.97	0.92	8
-	I3L6A5	PIG Uncharacterized protein CHST2	1.425	-1.27	-1.07	-0.75	0.86	0.85	0.91	0.96	1
-	G9BWQ1	PIG V-akt murine thymoma viral oncogene-like 1	-0.4	-0.74	-1.18	-1.34	0.99	0.99	0.88	0.83	1
P15924	F1RW75	PIG Uncharacterized protein DSP	-1.55	-2.08	-1.37	-1.1	0.81	0.40	0.81	0.89	75
-	Q9NPH3	HUMAN Interleukin-1 receptor accessory protein	-1.28	-1.31	-1.2	-1.35	0.91	0.83	0.87	0.83	1
-	I3LDH5	PIG Uncharacterized protein AOX1	-1.34	-1.73	-1.47	-1.82	0.89	0.61	0.76	0.73	1
-	F1SJM8	PIG Uncharacterized protein IGSF10	-1.24	-1.07	-1.68	-1.81	0.91	0.92	0.69	0.73	1
P35367	F1SQA5	PIG Uncharacterized protein HRH1	-2.84	-2.92	-2.12	-1.43	0.17	0.09	0.46	0.82	1
-	P22301	HUMAN Interleukin-10	-2.96	-3.56	-3.03	-2.36	0.13	0.02	0.11	0.52	1

Adhesion of blood cells

Human equivalent Ac. No.	Accession No.	Protein	Zq (log2 FC)				FDR				No. peptides
			120	1	4	7	120	1	4	7	
-	P27917	PIG Apolipoprotein C-III	2.024	6.07	4.55	1.409	0.57	0.00	0.00	0.82	7
-	I3LQR9	PIG Fibrinogen alpha chain	1.343	2.49	3.618	3.539	0.89	0.23	0.03	0.11	11
-	F1RX36	PIG Fibrinogen alpha chain	0.735	2.968	4.25	3.62	0.97	0.09	0.00	0.09	4
-	P19619	PIG Annexin A1	0.759	3.181	3.067	2.43	0.97	0.05	0.11	0.49	16
-	Q06AU2	PIG Ras-related protein Rap-2a	2.086	2.385	2.384	1.985	0.53	0.27	0.33	0.67	2
-	O19063	PIG Serum amyloid P-component	1.188	2.679	3.137	1.279	0.93	0.16	0.09	0.85	6
-	O19062	PIG C-reactive protein	1.116	3.654	4.381	1.655	0.93	0.01	0.00	0.78	7
-	A7WPA5	PIG P-selectin glycoprotein ligand 1 propeptide	1.192	5.124	4.126	1.264	0.93	0.00	0.01	0.85	1
-	Q19AZ8	PIG Prothrombin	2.411	2.994	1.957	1.057	0.36	0.08	0.54	0.90	6
-	K7ZJP7	PIG IgM heavy chain constant region (Fragment)	1.687	2.275	3.188	1.567	0.75	0.32	0.08	0.80	12
-	C3S7K6	PIG Calcium-binding protein A9	0.956	4.087	2.718	0.47	0.95	0.00	0.21	0.98	7

-	P14632	PIG Lactotransferrin	2.049	4.138	2.116	-0.21	0.55	0.00	0.46	0.99	16
P02749	I3LGN5	PIG Uncharacterized protein (Fragment)	1.686	2.42	2.192	1.502	0.75	0.25	0.44	0.81	1
-	P18650	PIG Apolipoprotein E	0.934	3.405	3.194	1.376	0.96	0.03	0.08	0.83	25
-	P01025	PIG Complement C3	2.501	1.593	1.876	1.685	0.32	0.67	0.58	0.77	83
P01023	I3LQ17	PIG Uncharacterized protein	2.016	1.788	2.079	1.597	0.57	0.57	0.48	0.80	50
-	K7GR72	PIG Uncharacterized protein SLC4A1	0.625	3.504	3.243	0.672	0.98	0.02	0.07	0.96	14
-	F1SGT4	PIG Uncharacterized protein CD44	1.007	2.072	2.186	1.712	0.96	0.41	0.44	0.75	3
-	Q9GLP2	PIG Vitamin K-dependent protein C	1.425	1.189	1.521	1.181	0.86	0.88	0.74	0.87	6
P01024	I3LTB8	PIG Uncharacterized protein	1.888	1.799	1.664	1.131	0.63	0.56	0.69	0.88	2
-	K9IVW2	PIG Integrin alpha-X	1.852	1.931	1.32	0.69	0.66	0.49	0.83	0.96	1
-	A2SW51	PIG Monocyte differentiation antigen CD14	1.601	2.47	1.714	1.165	0.78	0.23	0.67	0.87	2
P02751	F1SS24	PIG Uncharacterized protein FN1	0.527	0.684	1.731	2.499	0.99	0.99	0.67	0.46	63
-	P05109	HUMAN Protein S100-A8	-0.72	-0.47	0.445	1.595	0.97	1.00	0.99	0.80	1
-	I3LK80	PIG Uncharacterized protein ELANE	1.15	1.925	1.315	0.759	0.93	0.49	0.83	0.96	2
-	F1RM45	PIG Apolipoprotein E	0.448	2.486	2.694	0.54	0.99	0.22	0.21	0.97	2
-	P06702	HUMAN Protein S100-A9	0.489	1.245	0.759	0.297	0.98	0.86	0.97	0.99	2
-	Q15848	HUMAN Adiponectin	0.918	0.866	1.13	2.405	0.96	0.97	0.90	0.51	1
-	Q6VPV1	PIG Complement component C5	1.127	1.588	1.7	1.062	0.93	0.68	0.67	0.90	20
-	P09326	HUMAN CD48 antigen	1.59	1.68	0.139	0.332	0.78	0.64	1.00	0.99	1
-	P02788	HUMAN Lactotransferrin	1.665	1.629	1.164	0.709	0.76	0.66	0.88	0.96	1
-	P53714	PIG Integrin beta-2	1.232	0.766	1.018	1.091	0.91	0.99	0.92	0.89	6
P23284	I3LUC8	PIG Uncharacterized protein LOC100154783	0.172	0.475	1.321	2.136	0.99	1.00	0.83	0.59	10
-	K9J6H8	PIG Alpha-2-macroglobulin	1.728	1.975	1.262	-0.11	0.73	0.47	0.85	1.00	3
-	A8DSD5	PIG Integrin beta	0.901	0.853	1.091	1.131	0.96	0.98	0.90	0.88	1
-	F1SIW0	PIG Uncharacterized protein STAB1	1.787	1.642	1.45	0.314	0.70	0.65	0.77	0.99	2
-	O46409	PIG Apolipoprotein A-IV	1.573	1.955	0.882	0.361	0.80	0.47	0.94	0.99	30
P00742	F1RN41	PIG Uncharacterized protein F10	0.768	1.64	1.585	0.785	0.97	0.65	0.72	0.95	5

-	F1RMJ0	PIG Tyrosine-protein kinase	2.761	2.319	0.721	0.688	0.20	0.29	0.97	0.96	2
-	A7WLI1	PIG CD81	1.053	0.875	0.751	0.913	0.95	0.97	0.97	0.92	1
Q86UX7	F1RQ01	PIG Uncharacterized protein FERMT3	0.785	1.243	1.309	0.999	0.96	0.86	0.83	0.92	8
-	Q8WMN8	PIG Lactoferrin (Fragment)	1.302	1.583	0.573	0.107	0.90	0.68	0.98	1.00	2
-	F1SJT7	PIG Apolipoprotein A-IV	1.4	1.571	1.016	0.37	0.87	0.69	0.91	0.99	1
-	A3EX84	PIG Galectin	-0.59	0.06	1.552	2.42	0.99	1.00	0.73	0.50	4
-	O14746	HUMAN Telomerase reverse transcriptase	1.55	1.253	1.069	0.831	0.81	0.85	0.91	0.94	3
-	Q28833	PIG von Willebrand factor (Fragment)	0.779	1.131	0.987	0.846	0.97	0.90	0.92	0.94	7
-	P02751	HUMAN Fibronectin	0.889	0.355	0.789	0.961	0.96	1.01	0.96	0.92	5
-	A8DNU5	PIG Integrin alpha-L	1.129	1.135	0.806	0.609	0.93	0.90	0.96	0.97	2
-	F1S0A2	PIG Peptidyl-prolyl cis-trans isomerase	0.431	0.594	1.111	1.337	0.99	0.99	0.91	0.83	4
-	F1RJ33	PIG Galectin	0.297	0.696	1.08	0.778	1.00	0.99	0.91	0.95	2
-	P14460	PIG Fibrinogen alpha chain (Fragment)	0.285	0.985	0.488	0.584	0.99	0.94	0.99	0.97	2
-	Q9GKE8	PIG Leukocyte surface antigen CD47	0.301	0.591	0.921	0.421	1.00	0.99	0.93	0.99	1
Q96S97	F1RNK3	PIG Uncharacterized protein LOC100519316	0.722	0.671	0.455	0.593	0.97	0.99	0.99	0.97	3
-	P14287	PIG Osteopontin	0.038	0.042	0.493	1.487	1.00	1.00	0.99	0.81	6
-	A0AVL1	HUMAN ADAM9 protein	0.083	0.02	0.376	1.285	1.00	1.00	0.99	0.85	1
-	P31146	HUMAN Coronin-1A	-0.49	0.502	0.76	0.4	0.99	1.00	0.97	0.99	1
-	P52552	PIG Peroxiredoxin-2 (Fragment)	-0.79	1.34	1.782	0.596	0.96	0.81	0.64	0.97	8
-	P26042	PIG Moesin	-0.49	-0.18	0.731	1.353	0.99	1.00	0.97	0.83	46
-	F1S1G8	PIG Amine oxidase	-0	0.502	0.547	0.493	1.00	1.00	0.98	0.98	9
-	F1SCN0	PIG Uncharacterized protein (Fragment) VAV1	0.476	0.675	0.851	0.645	0.99	0.99	0.95	0.96	1
O95267	F1SS17	PIG Uncharacterized protein RASGRP1	0.988	0.173	0.75	0.798	0.95	1.00	0.97	0.95	2
-	F1RTN3	PIG Moesin	-0.33	-0.35	0.604	1.164	1.00	1.01	0.98	0.87	3
-	P40189	HUMAN Interleukin-6 receptor subunit beta	1.899	0.249	-0.9	-1.82	0.63	1.00	0.94	0.73	1
-	F1SAX5	PIG Uncharacterized protein (Fragment) CD58	2.079	0.677	-0.02	-0.16	0.53	0.99	1.00	1.00	1
-	Q9GLP0	PIG Integrin beta-1	0.19	0.551	0.363	0.497	0.99	0.99	1.00	0.98	12

-	Q9NNX6	HUMAN CD209 antigen	0.222	-0.08	0.229	0.304	0.99	1.00	1.00	0.99	1
-	Q8WMN7	PIG Plasma phospholipid transfer protein	1.009	1.557	0.049	-0.38	0.96	0.70	1.00	0.99	1
-	Q8SQC1	PIG Scavenger receptor class B member 1	-0.01	0.204	0.256	0.59	1.00	1.00	1.00	0.97	1
-	Q9UEW3	HUMAN Macrophage receptor MARCO	0.1	-0.04	0.268	0.499	1.00	1.00	1.00	0.98	1
P63000	I3LFI0	PIG Uncharacterized protein (Fragment) RAC1	0.262	-0.24	0.024	0.243	0.99	1.00	1.00	0.99	3
-	F1STD6	PIG Tyrosine-protein kinase	-0.56	-0.03	0.106	1.004	0.98	1.00	1.00	0.92	1
-	O75882	HUMAN Attractin	0.276	-0.36	-0.27	-0.75	0.99	1.01	1.00	0.96	1
-	K7GS51	PIG Uncharacterized protein (Fragment) ITGA1	0.839	0.124	-0.4	-0.61	0.96	1.00	1.00	0.97	2
Q12846	F1RIR2	PIG Uncharacterized protein STX4	-0.8	-0.24	-0.22	-0.07	0.96	1.00	1.00	1.00	2
-	P82460	PIG Thioredoxin	-1.19	-0.21	0.568	0.916	0.93	1.00	0.98	0.92	4
-	Q95242	PIG Platelet endothelial cell adhesion molecule	0.074	-0.02	-0.33	0.095	1.00	1.00	1.00	1.00	4
-	F1RYZ1	PIG Uncharacterized protein CD151	-1.39	-0.49	-0.1	0.537	0.87	1.00	1.00	0.97	1
-	F1RIS5	PIG Uncharacterized protein (Fragment) ITGAM	-1.35	-0.53	-0.1	0.314	0.89	1.00	1.00	0.99	1
-	I3LR17	PIG Uncharacterized protein CORO1A	-0.46	-1.08	-0.95	0.639	0.99	0.91	0.93	0.96	1
-	P21980	HUMAN Protein-glutamine gamma-glutamyltransferase 2	-1.81	-1.81	-0.37	0.689	0.69	0.56	0.99	0.96	1
P15311	F1SB42	PIG Uncharacterized protein (Fragment) EZR	-1.1	-0.83	-0.25	-0.06	0.94	0.98	1.00	1.00	13
Q02750	I3LQS6	PIG Uncharacterized protein (Fragment) MAP2K1	-0.74	-0.93	-0.17	0.036	0.97	0.96	1.00	1.00	3
-	P55058	HUMAN Phospholipid transfer protein	-0.32	-0.85	-0.97	-0.57	1.00	0.98	0.93	0.97	1
P02549	F1RR78	PIG Uncharacterized protein LOC100049693	-0.89	-1.12	-0.83	-0.49	0.96	0.90	0.95	0.98	148
P21980	F1SDX6	PIG Uncharacterized protein TGM2	-1.63	-1.82	-0.77	-0.02	0.77	0.55	0.97	1.00	26
-	P15311	HUMAN Ezrin	-1.58	-1.68	-0.19	0.474	0.79	0.64	1.00	0.98	4
-	O15230	HUMAN Laminin subunit alpha-5	-1.81	-1.37	-0.28	0.013	0.69	0.80	1.00	1.00	3
-	Q3HUX1	PIG Fatty acid translocase/CD36	-0.69	-0.18	-0.77	-0.95	0.98	1.00	0.97	0.92	8
-	P56524	HUMAN Histone deacetylase 4	-0.33	-0.9	-1.41	-1.37	1.00	0.97	0.79	0.83	1
-	F1S430	PIG Uncharacterized protein (Fragment) LOC100525578	-0.4	-0.9	-1.67	-1.27	0.99	0.97	0.69	0.85	1
-	Q9N1X4	PIG Pulmonary surfactant-associated protein D	-1.81	-1.04	-1.32	-1.06	0.69	0.93	0.83	0.90	1
-	Q2TAM5	HUMAN RELA protein	-1.14	-1.48	-1.18	-0.46	0.93	0.73	0.88	0.99	1

-	F1S2E3	PIG Peptidyl-prolyl cis-trans isomerase	-0.98	-1.2	-2.11	-1.65	0.96	0.87	0.47	0.77	7
-	H0YCB4	HUMAN Transcription factor p65 (Fragment)	-1.13	-1.83	-2.34	-1.88	0.93	0.54	0.35	0.70	1
-	P22301	HUMAN Interleukin-10	-2.96	-3.56	-3.03	-2.36	0.13	0.02	0.11	0.52	1

Human equivalent Ac. No.	Accession No.	Wound healing<br>Protein	Zq (log2 FC) 120	1	4	7	FDR 120	1	4	7	No. peptides
-	I3LQR9	PIG Fibrinogen alpha chain	1.343	2.49	3.618	3.539	0.89	0.23	0.03	0.11	11
-	F1RX36	PIG Fibrinogen alpha chain	0.735	2.968	4.25	3.62	0.97	0.09	0.00	0.09	4
P02675	I3L651	PIG Uncharacterized protein	1.292	2.457	3.366	3.096	0.91	0.24	0.05	0.23	27
-	P00747	HUMAN Plasminogen	1.196	4.368	3.759	1.98	0.93	0.00	0.02	0.67	1
P11940	F1RPL9	PIG Uncharacterized protein LOC100525442	3.048	2.503	2.287	2.18	0.11	0.22	0.38	0.59	1
-	P06867	PIG Plasminogen	1.073	2.63	3.175	1.432	0.94	0.17	0.08	0.82	13
-	F1SB81	PIG Plasminogen	0.204	2.644	2.932	2.36	0.99	0.17	0.14	0.52	2
-	Q19AZ8	PIG Prothrombin	2.411	2.994	1.957	1.057	0.36	0.08	0.54	0.90	6
-	P06396	HUMAN Gelsolin	1.931	3.018	2.411	1.575	0.61	0.08	0.32	0.81	1
-	P20305	PIG Gelsolin (Fragment)	0.639	3.032	2.689	1.409	0.98	0.07	0.21	0.82	25
-	Q9GLP2	PIG Vitamin K-dependent protein C	1.425	1.189	1.521	1.181	0.86	0.88	0.74	0.87	6
-	Q99075	HUMAN Proheparin-binding EGF-like growth factor	1.717	2.162	1.556	0.042	0.73	0.37	0.73	1.00	1
P02751	F1SS24	PIG Uncharacterized protein FN1	0.527	0.684	1.731	2.499	0.99	0.99	0.67	0.46	63
-	K7GQL2	PIG Coagulation factor XIII, A1 polypeptide	1.041	1.609	2.174	1.22	0.95	0.67	0.44	0.86	3
-	P14477	PIG Fibrinogen beta chain (Fragment)	-0.18	1.518	1.03	1.911	0.99	0.72	0.92	0.69	1
-	Q02297	HUMAN Pro-neuregulin-1, membrane-bound isoform	0.258	2.377	1.5	0.9	0.99	0.27	0.75	0.92	1
-	Q9GLP1	PIG Coagulation factor V	1.122	1.457	1.776	0.899	0.93	0.74	0.64	0.92	9
-	P16293	PIG Coagulation factor IX (Fragment)	1.951	1.716	1.332	1.102	0.61	0.61	0.83	0.89	2
-	P50440	HUMAN Glycine amidinotransferase, mitochondrial	1.325	0.561	0.836	0.736	0.90	0.99	0.95	0.96	1
P00742	F1RN41	PIG Uncharacterized protein F10	0.768	1.64	1.585	0.785	0.97	0.65	0.72	0.95	5

-	P32394	PIG Heme oxygenase 1	0.774	0.663	1.096	1.001	0.97	0.99	0.90	0.92	7
-	P02751	HUMAN Fibronectin	0.889	0.355	0.789	0.961	0.96	1.01	0.96	0.92	5
-	P02675	HUMAN Fibrinogen beta chain	3.779	0.632	-0.02	-0.17	0.02	0.99	1.00	0.99	2
P07225	I3LQM5	PIG Uncharacterized protein PROS1	0.885	1.705	1.293	0.299	0.96	0.62	0.84	0.99	6
-	P14460	PIG Fibrinogen alpha chain (Fragment)	0.285	0.985	0.488	0.584	0.99	0.94	0.99	0.97	2
-	P37176	PIG Endoglin	1.135	1.103	0.629	0.132	0.93	0.90	0.98	1.00	3
-	A5A770	PIG Dystrobrevin-binding protein 1	1.105	1.724	0.695	-0.58	0.94	0.61	0.97	0.97	1
P17677	I3LTW2	PIG Uncharacterized protein (Fragment) GAP43	0.044	-0.37	0.179	0.711	1.00	1.01	1.00	0.96	1
-	Q8MJ14	PIG Glutathione peroxidase 1	0.36	0.465	0.562	0.734	1.00	1.00	0.98	0.96	8
Q9UBX5	F1SD87	PIG Uncharacterized protein (Fragment) FBLN5	0.313	0.297	0.011	0.041	1.00	1.00	1.00	1.00	6
-	O97506	PIG Kallikrein	0.582	-0.7	-0.27	0.079	0.98	0.99	1.00	1.00	4
-	J9JIL1	PIG Guanine nucleotide-binding protein G(q) subunit alpha (Fragment)	0.941	-0.17	-0.22	-0.16	0.96	1.00	1.00	1.00	2
-	B3STX8	PIG Thrombomodulin	-0.98	0.127	0.581	0.711	0.96	1.00	0.98	0.96	1
O75923	F1SLE7	PIG Uncharacterized protein LOC100522732	0.158	0.306	-0.09	-0.24	0.99	1.00	1.00	0.99	7
P0CB38	F1SV06	PIG Uncharacterized protein PABPC4	-0.69	-0.99	-0.12	0.081	0.98	0.94	1.00	1.00	10
Q14344	F1RV20	PIG Uncharacterized protein GNA13	0.845	0.073	-0.51	-0.72	0.96	1.00	0.99	0.96	1
-	I3L6Q6	PIG Uncharacterized protein (Fragment) F13B	-0.18	-0.5	-0.69	-1.08	0.99	1.00	0.97	0.89	1
-	E5RI09	HUMAN Beta-enolase (Fragment)	-1.49	-0.5	-0.78	-0.49	0.83	1.00	0.96	0.98	1
-	Q1KYT0	PIG Beta-enolase	-2.2	-3.1	-2.23	-1.85	0.46	0.07	0.41	0.72	30
-	P55085	HUMAN Proteinase-activated receptor 2	-4.54	-4.45	-2.73	-2.22	0.00	0.00	0.20	0.57	1

Production of NO and ROS

Human equivalent Ac. No.	Accession No.	Protein	Zq (log2 FC)				FDR				No. peptides
			120	1	4	7	120	1	4	7	
Q13233	F1SLK9	PIG Uncharacterized protein MAP3K1	3.134	7.697	6.058	2.267	0.09	0.00	0.00	0.55	1
-	P27917	PIG Apolipoprotein C-III	2.024	6.07	4.55	1.409	0.57	0.00	0.00	0.82	7
P45985	F1SS51	PIG Uncharacterized protein (Fragment) MAP2K4	2.622	3.106	3.149	2.857	0.26	0.06	0.09	0.33	1

-	P18648	PIG Apolipoprotein A-I	2.421	2.571	1.779	1.218	0.36	0.19	0.64	0.86	34
-	P27485	PIG Retinol-binding protein 4	2.456	2.319	1.927	1.476	0.34	0.30	0.55	0.81	2
-	Q29549	PIG Clusterin	2.275	3.807	3.763	1.741	0.43	0.01	0.02	0.75	28
-	P08835	PIG Serum albumin	3.428	2.943	2.28	1.742	0.04	0.09	0.39	0.75	27
-	P18650	PIG Apolipoprotein E	0.934	3.405	3.194	1.376	0.96	0.03	0.08	0.83	25
-	P02768	HUMAN Serum albumin	1.769	2.264	1.765	0.632	0.71	0.32	0.65	0.96	3
-	F1SLB7	PIG Uncharacterized protein (Fragment) APOF	1.618	3.517	2.547	0.787	0.77	0.02	0.26	0.95	3
P05090	F1SQX9	PIG Uncharacterized protein APOD	1.326	2.504	2.021	0.443	0.90	0.22	0.51	0.99	3
-	F1S1A9	PIG Uncharacterized protein APOA2	1.175	1.666	1.865	1.51	0.92	0.64	0.59	0.81	3
-	P05109	HUMAN Protein S100-A8	-0.72	-0.47	0.445	1.595	0.97	1.00	0.99	0.80	1
-	P50447	PIG Alpha-1-antitrypsin	2.605	2.139	1.561	1.19	0.27	0.38	0.73	0.87	12
P05164	K7GRV6	PIG Uncharacterized protein MPO	0.927	2.497	1.746	0.327	0.96	0.22	0.66	0.99	10
-	F1RM45	PIG Apolipoprotein E	0.448	2.486	2.694	0.54	0.99	0.22	0.21	0.97	2
-	I3LKA3	PIG Tyrosine-protein phosphatase non-receptor type	1.015	1.107	1.491	1.248	0.96	0.90	0.75	0.86	7
-	Q06190	HUMAN Serine/threonine-protein phosphatase 2A regulatory subunit B	0.307	0.171	0.421	-0.15	1.00	1.00	1.00	1.00	1
P04114	I3LKZ1	PIG Uncharacterized protein	0.847	1.018	1.486	1.379	0.96	0.93	0.75	0.83	16
P04114	F1SCV8	PIG Uncharacterized protein (Fragment)	1.058	1.399	1.319	1.01	0.95	0.78	0.83	0.92	25
-	Q02779	HUMAN Mitogen-activated protein kinase kinase kinase 10	1.335	1.343	1.513	1.259	0.90	0.81	0.75	0.85	1
-	H0YA55	HUMAN Serum albumin (Fragment)	2.347	2.004	1.349	1.456	0.39	0.45	0.82	0.82	1
-	Q06AT6	PIG RHOG	0.201	0.185	1.47	1.842	0.99	1.00	0.76	0.72	5
P84095	I3L725	PIG Uncharacterized protein RHOG	1.999	1.423	1.208	0.98	0.58	0.77	0.86	0.92	2
-	P08134	HUMAN Rho-related GTP-binding protein RhoC	-0.05	0.073	1.388	2.004	1.00	1.00	0.80	0.66	3
-	A4US67	PIG Paraoxonase	1.488	1.378	0.988	0.053	0.83	0.79	0.92	1.00	6
-	Q764M5	PIG Signal transducer and activator of transcription 1	1.111	0.314	0.849	0.971	0.94	1.00	0.95	0.92	14
Q06190	F1SL86	PIG Uncharacterized protein (Fragment)	0.899	0.742	0.986	1.021	0.96	0.99	0.92	0.91	1
-	O46409	PIG Apolipoprotein A-IV	1.573	1.955	0.882	0.361	0.80	0.47	0.94	0.99	30
-	G3V387	HUMAN Short peptide from AAT (Fragment)	1.437	1.245	0.788	0.556	0.85	0.86	0.96	0.98	1

-	B1PK10	PIG Neutrophil cytosolic factor 2	0.655	0.294	0.982	0.781	0.98	1.00	0.93	0.95	1
-	Q2LE37	PIG Apolipoprotein M	0.955	0.951	1.121	0.566	0.95	0.95	0.91	0.97	2
-	F1SJT7	PIG Apolipoprotein A-IV	1.4	1.571	1.016	0.37	0.87	0.69	0.91	0.99	1
-	P52649	PIG Cytochrome b-245 heavy chain (Fragment)	0.899	1.093	0.834	0.424	0.96	0.91	0.95	0.99	3
P35580	F1SSA6	PIG Uncharacterized protein (Fragment) LOC396903	0.245	0.275	0.559	1.388	0.99	1.00	0.98	0.83	70
-	Q29021	PIG Apolipoprotein B (Fragment)	1.063	0.566	0.926	0.508	0.95	0.99	0.93	0.98	12
Q15257	F1RR44	PIG Uncharacterized protein PPP2R4	1.322	0.353	0.595	0.763	0.90	1.01	0.98	0.96	3
-	I3LR85	PIG Protein kinase C delta type	0.06	0.027	0.998	1.286	1.00	1.00	0.92	0.85	1
Q16539	F1RYA1	PIG Uncharacterized protein MAPK14	0.409	0.406	1.014	0.657	0.99	1.01	0.92	0.96	2
-	P01100	HUMAN Proto-oncogene c-Fos	0.723	0.362	0.213	0.692	0.97	1.01	1.00	0.96	1
-	F1RUN2	PIG Serum albumin	1.327	0.551	0.491	0.512	0.90	0.99	0.99	0.98	2
Q14204	F1S9Y5	PIG Uncharacterized protein (Fragment) PPP2R5C	0.452	0.862	0.666	0.665	0.99	0.97	0.97	0.96	1
-	F1S659	PIG Uncharacterized protein NCF2	1.325	0.85	0.125	0.065	0.90	0.98	1.00	1.00	1
P27361	I3LVK2	PIG Uncharacterized protein (Fragment) MAPK3	0.585	0.463	0.676	0.198	0.98	1.00	0.97	0.99	2
-	P79275	PIG Rho A (Fragment)	0.67	0.219	0.385	0.604	0.98	1.00	0.99	0.97	1
-	A5GFQ9	PIG Protein phosphatase 2, regulatory subunit B (B56), delta isoform	0.891	0.816	0.505	0.344	0.96	0.98	0.99	0.99	2
-	P61224	HUMAN Ras-related protein Rap-1b	0.293	0.069	0.337	0.642	1.00	1.00	0.99	0.96	2
-	I3L9F5	PIG Uncharacterized protein (Fragment) PCYOX1	1.002	0.955	0.314	-0.23	0.96	0.95	1.00	0.99	7
-	A6XAC5	PIG Protein phosphatase 2A regulatory subunit B B56 alpha isoform	1.561	0.772	0.778	0.631	0.80	0.98	0.97	0.96	1
-	H7C118	HUMAN Protein phosphatase 1 regulatory subunit 7 (Fragment)	0.318	0.265	0.709	1.439	1.00	1.00	0.97	0.82	1
-	F1SGS9	PIG Catalase	0.558	1.08	0.893	0.246	0.98	0.91	0.94	0.99	3
-	K7GLE0	PIG Uncharacterized protein NCF4	1.177	0.096	-0.13	-0.58	0.92	1.00	1.00	0.97	2
-	A7XNS1	PIG Nuclear factor of kappa light polypeptide enhancer in B-cells 1	1.576	1.025	0.599	0.161	0.79	0.93	0.98	1.00	2
Q9Y243	K7GNL2	PIG Uncharacterized protein (Fragment) AKT3	0.696	0.681	-0.28	-0.15	0.98	0.99	1.00	1.00	1
-	O62839	PIG Catalase	-0.09	1.227	0.904	-0.01	1.00	0.86	0.93	1.00	18
-	Q15435	HUMAN Protein phosphatase 1 regulatory subunit 7	-0.48	0.039	-0.12	0.166	0.99	1.00	1.00	1.00	1
P62834	I3LF84	PIG Uncharacterized protein RAP1A	-0.64	-0.46	0.321	0.797	0.98	1.00	1.00	0.95	6

-	Q9Y2U5	HUMAN Mitogen-activated protein kinase kinase kinase 2	-1.41	-1.47	0.149	0.898	0.86	0.73	1.00	0.92	1
-	P62136	HUMAN Serine/threonine-protein phosphatase PP1-alpha catalytic subunit	-0.21	-0.21	0.426	0.565	0.99	1.00	1.00	0.97	2
P63000	I3LFI0	PIG Uncharacterized protein (Fragment) RAC1	0.262	-0.24	0.024	0.243	0.99	1.00	1.00	0.99	3
-	P04114	HUMAN Apolipoprotein B-100	0.647	-0.06	-0.2	0.209	0.98	1.00	1.00	0.99	6
Q16537	F1SRR2	PIG Uncharacterized protein PPP2R5E	0.493	-0.4	-0.3	0.057	0.99	1.01	1.00	1.00	1
P27169	F1SFA2	PIG Uncharacterized protein LOC100620687	1.402	0.231	-0.16	-0.48	0.87	1.00	1.00	0.98	1
-	Q29090	PIG Serine/threonine-protein phosphatase 2A 55 kDa regulatory subunit B	-0.18	-0.46	-0.25	0.079	0.99	1.00	1.00	1.00	8
-	P05164	HUMAN Myeloperoxidase	-0.08	0.235	-0.27	-0.38	1.00	1.00	1.00	0.99	1
-	Q864R5	PIG Mitochondrial Rho GTPase 2	-0.22	-0.27	-0.22	0.074	0.99	1.00	1.00	1.00	1
-	P67776	PIG Serine/threonine-protein phosphatase 2A catalytic subunit alpha isoform	0.2	-0.34	-0.05	0.129	0.99	1.01	1.00	1.00	6
-	P54612	PIG Serine/threonine-protein phosphatase 2A 65 kDa regulatory subunit A	-0.39	-0.88	-0.3	-0.02	0.99	0.97	1.00	1.00	17
-	M3V839	PIG Mitogen-activated protein kinase 3	-0.25	-0.92	-0.64	0.221	0.99	0.96	0.98	0.99	3
Q02750	I3LQS6	PIG Uncharacterized protein (Fragment) MAP2K1	-0.74	-0.93	-0.17	0.036	0.97	0.96	1.00	1.00	3
P62834	F2Z5K3	PIG Uncharacterized protein RAP1A	-0.66	-0.46	-0.52	-0.56	0.98	1.00	0.99	0.98	1
-	P62745	HUMAN Rho-related GTP-binding protein RhoB	-0.07	-0.52	-0.56	-0.28	1.00	1.00	0.98	0.99	1
-	A9QT41	PIG Inhibitor of kappa light polypeptide gene enhancer in B-cells kinase gamma	0.087	0.011	-0.2	-0.54	1.00	1.00	1.00	0.97	2
Q15435	F1SI09	PIG Uncharacterized protein PPP1R7	-0.01	-0.62	-0.69	-0.43	1.00	0.99	0.97	0.99	9
-	F1SJC2	PIG Uncharacterized protein PPP1R3A	-0.71	-1.13	-0.31	-0.35	0.98	0.90	1.00	0.99	1
-	Q9TV77	PIG 130 kDa regulatory subunit of myosin phosphatase (Fragment)	-0.62	-1.28	-0.6	-0.01	0.98	0.84	0.98	1.00	3
P02549	F1RR78	PIG Uncharacterized protein LOC100049693	-0.89	-1.12	-0.83	-0.49	0.96	0.90	0.95	0.98	148
O14974	I3LVP5	PIG Uncharacterized protein PPP1R12A	-0.93	-1.38	-0.44	0.351	0.96	0.79	0.99	0.99	4
-	F1S7V6	PIG Uncharacterized protein PPP1R14C	-0.03	-1.32	-0.93	-0.63	1.00	0.82	0.93	0.96	1
-	P08519	HUMAN Apolipoprotein(a)	-1.14	-1.11	-0.99	-0.43	0.93	0.90	0.92	0.99	1
P24723	I3LEA7	PIG Uncharacterized protein	-1	-1.03	-0.15	-0.1	0.96	0.93	1.00	1.00	1
-	P61586	HUMAN Transforming protein RhoA	-3	-1.81	-0.34	1.034	0.12	0.56	1.00	0.91	2
-	O14974	HUMAN Protein phosphatase 1 regulatory subunit 12A	0.879	0.709	-1.42	-1.86	0.96	0.99	0.78	0.71	1
-	Q2EHH7	PIG Serine/threonine-protein phosphatase	-0.85	-1.26	-0.5	-0.02	0.96	0.85	0.99	1.00	3

Q9Y6R4	K7GP00	PIG Uncharacterized protein (Fragment) LOC100518100	-1.09	-0.31	-0.97	-0.94	0.94	1.00	0.93	0.92	2
-	P63151	HUMAN Serine/threonine-protein phosphatase 2A 55 kDa regulatory subunit B	-1.03	-1.4	-0.78	-0.34	0.95	0.78	0.96	0.99	1
-	G9BWQ1	PIG V-akt murine thymoma viral oncogene-like 1	-0.4	-0.74	-1.18	-1.34	0.99	0.99	0.88	0.83	1
Q5JR12	F1SBQ0	PIG Uncharacterized protein PPM1J	-1.9	-1.66	-1.3	-0.98	0.63	0.64	0.84	0.92	1
-	Q2TAM5	HUMAN RELA protein	-1.14	-1.48	-1.18	-0.46	0.93	0.73	0.88	0.99	1
-	H0YCB4	HUMAN Transcription factor p65 (Fragment)	-1.13	-1.83	-2.34	-1.88	0.93	0.54	0.35	0.70	1
-	Q9Y6R4	HUMAN Mitogen-activated protein kinase kinase kinase 4	-3.51	-3.04	-3.92	-3.1	0.03	0.07	0.01	0.23	1

Supplementary Table S2. Protein expression changes in the functionally annotated categories within cluster b.

Human equivalent Ac. No.	Accession No.	Cell junction	Zq (log2 FC)				FDR				No. peptides
		Protein	120	1	4	7	120	1	4	7	
Q14192	F1SU18	PIG Uncharacterized protein (Fragment) FHL2	-3.93	-4.76	-3.23	-2.24	0.01	0.00	0.07	0.55	5
Q5VT25	F1S8P5	PIG Uncharacterized protein (Fragment) LOC100511216	-2.54	-2.8	-2.05	-1.71	0.30	0.12	0.49	0.75	1
-	Q14192	HUMAN Four and a half LIM domains protein 2	-2.04	-3.35	-2.56	-2.06	0.57	0.03	0.25	0.64	1
-	F1SUE9	PIG Uncharacterized protein (Fragment) COL13A1	-1.84	-1.99	-2.15	-1.98	0.68	0.46	0.45	0.67	1
-	I3LRH7	PIG Uncharacterized protein DSC3	-0.87	-2.01	-1.52	-1.1	0.96	0.44	0.74	0.89	1
-	Q8WNW4	PIG Beta-catenin	-1.48	-1.78	-1.42	-1.11	0.84	0.57	0.79	0.88	19
Q9UI47	F1SUJ6	PIG Uncharacterized protein CTNNA3	-1.25	-1.75	-1.92	-1.55	0.91	0.59	0.55	0.80	1
-	Q03135	HUMAN Caveolin-1	-0.66	-1.44	-1.89	-1.92	0.98	0.75	0.57	0.69	1
P15924	F1RW75	PIG Uncharacterized protein DSP	-1.55	-2.08	-1.37	-1.1	0.81	0.40	0.81	0.89	75
-	P48169	HUMAN Gamma-aminobutyric acid receptor subunit alpha-4	-1.76	-1.84	-1.32	-0.87	0.71	0.54	0.83	0.93	1
Q9UI47	I3LUC9	PIG Uncharacterized protein	-0.54	-0.94	-1.49	-1.21	0.98	0.96	0.75	0.87	1
Q9Y4J8	I3LG33	PIG Uncharacterized protein (Fragment) LOC100627907	-0.78	-1.35	-1.3	-1.44	0.97	0.81	0.84	0.82	4
-	I3LVH8	PIG Uncharacterized protein (Fragment) DSC2	-1.15	-1.06	-1.18	-1.15	0.93	0.92	0.88	0.87	3
-	Q96F07	HUMAN Cytoplasmic FMR1-interacting protein 2	-1.53	-0.76	-1.22	-1.47	0.82	0.99	0.86	0.81	1
P19022	F1SAM3	PIG Uncharacterized protein (Fragment) CDH2	-0.63	-0.58	-1.06	-1.2	0.98	0.99	0.91	0.87	14
-	Q9UQB3	HUMAN Catenin delta-2	-0.6	-0.82	-1.2	-0.63	0.99	0.98	0.87	0.96	1
O43491	F1SM86	PIG Uncharacterized protein EPB41L3	-0.96	-1.36	-1.08	-0.87	0.95	0.80	0.91	0.93	11
-	Q9UI47	HUMAN Catenin alpha-3	-0.93	-0.81	-0.63	-0.7	0.96	0.98	0.98	0.96	3
Q03001	F1RZU8	PIG Uncharacterized protein (Fragment) DST	-0.8	0.327	-1.77	-1.09	0.96	1.00	0.65	0.89	4
-	Q6RVA9	PIG Caveolin-1	-1.6	-1.09	-1.11	-0.81	0.78	0.91	0.91	0.95	7
-	Q5GN48	PIG Dystrophin	-0.99	-1.29	-1	-0.84	0.96	0.84	0.92	0.94	59

-	Q29101	PIG Gap junction alpha-1 protein	-0.75	-0.8	-0.95	-0.78	0.97	0.99	0.93	0.95	2
Q9Y2X7	F1RN87	PIG Uncharacterized protein (Fragment) GIT1	-1.23	-0.58	-0.88	-0.53	0.91	0.99	0.94	0.98	1
-	Q96RT1	HUMAN Protein LAP2	-0.66	-0.31	-0.65	-0.71	0.98	1.00	0.98	0.96	1
-	C3VML1	PIG Claudin	-1	-0.25	-0.48	-0.07	0.96	1.00	0.99	1.00	1
Q9NQX3	F1SA44	PIG Uncharacterized protein (Fragment) GPHN	-0.09	-0.6	-1.06	-0.36	1.00	0.99	0.91	0.99	1
-	Q5TAT6	HUMAN Collagen alpha-1(XIII) chain	-0.69	-0.22	0.023	-0.34	0.98	1.00	1.00	0.99	1
-	O02840	PIG Cadherin-5	-0.47	-0.08	-0.33	-0.24	0.99	1.00	1.00	0.99	4
P26232	F1SNX3	PIG Uncharacterized protein LOC100525516	-0.14	-0.21	-0.37	0.245	0.99	1.00	0.99	0.99	1
P35221	F1RGJ2	PIG Uncharacterized protein CTNNA1	-0.55	-0.77	-0.13	0.185	0.98	0.98	1.00	0.99	20
-	Q9Y2J2	HUMAN Band 4.1-like protein 3	-0.69	-0.61	-0.25	-0.98	0.98	0.99	1.00	0.92	3
-	Q9P2M7	HUMAN Cingulin	0.194	-0.09	-0.53	-0.99	0.99	1.00	0.98	0.92	1
-	P15924	HUMAN Desmoplakin	1.216	-0.12	-0.84	-0.82	0.92	1.00	0.95	0.94	2
-	A4GVD1	PIG Gap junction gamma-1 protein	-0.58	-0.32	-1	-0.88	0.98	1.00	0.92	0.93	1
Q9Y4J8	I3LB94	PIG Uncharacterized protein DTNA	0.324	-0.38	-0.59	-1.07	1.00	1.01	0.98	0.90	1
-	Q96KN9	HUMAN Gap junction delta-4 protein	-0.18	-0.41	-0.55	-0.27	0.99	1.01	0.98	0.99	1
Q12959	K7GN86	PIG Uncharacterized protein DLG1	-0.93	-0.19	0.559	0.106	0.96	1.00	0.98	1.00	1
-	F1SAM8	PIG Uncharacterized protein (Fragment) CDC42BPB	0.182	-0.22	-0.51	-0.46	0.99	1.00	0.99	0.99	1
-	C3VMW6	PIG Claudin	-0.86	-0.04	-0.18	0.323	0.96	1.00	1.00	0.99	1
Q8N8S7	F1S8S4	PIG Uncharacterized protein (Fragment) ENAH	-1.67	-1.05	0.284	1.181	0.76	0.93	1.00	0.87	3
-	C3VMK5	PIG Claudin	-0.02	-0.41	-0.58	-0.61	1.00	1.01	0.98	0.97	1
-	Q9UMD9	HUMAN Collagen alpha-1(XVII) chain	-1.29	-0.25	0.153	-1.01	0.91	1.00	1.00	0.91	1
P50570	F1S593	PIG Uncharacterized protein (Fragment) DNM2	-0.11	-0.16	0.117	0.127	1.00	1.00	1.00	1.00	3
-	Q14126	HUMAN Desmoglein-2	-1.21	-0.93	0.541	1.265	0.92	0.96	0.98	0.85	1
-	F8WDM7	HUMAN Caveolin-1	0.136	-0.19	0.03	0.123	0.99	1.00	1.00	1.00	1
-	I3LPQ6	PIG Uncharacterized protein (Fragment) CHRNA9	0.651	-0.11	-0.31	-0.33	0.98	1.00	1.00	0.99	1
Q9P2M7	F1ST04	PIG Uncharacterized protein CGN	-0.24	-0.07	0.467	0.563	0.99	1.00	0.99	0.97	1
-	Q03001	HUMAN Dystonin	0.408	0.263	0.287	0.447	0.99	1.00	1.00	0.99	3

P11230	I3L9G3	PIG Uncharacterized protein CHRNB1	0.777	0.492	-0.11	-0.23	0.97	1.00	1.00	0.99	1
-	M3V7Z1	PIG Cytoplasmic FMR1 interacting protein 1 tv1	-0.47	-0.72	0.22	0.441	0.99	0.99	1.00	0.99	1
Q16099	F1S9S0	PIG Uncharacterized protein (Fragment) GRIK4	0.561	0.488	0.015	0.525	0.98	1.00	1.00	0.98	1
O75899	I3L6R6	PIG Uncharacterized protein GABBR2	1.334	0.978	0.702	0.135	0.90	0.95	0.97	1.00	1
P17677	I3LTW2	PIG Uncharacterized protein (Fragment) GAP43	0.044	-0.37	0.179	0.711	1.00	1.01	1.00	0.96	1
-	I3LNL5	PIG Uncharacterized protein DIXDC1	-0.44	-0.09	0.636	1.123	0.99	1.00	0.98	0.88	1
P47869	I3L720	PIG Uncharacterized protein GABRA2	0.301	0.663	0.074	-1.54	1.00	0.99	1.00	0.80	1
-	P50570	HUMAN Dynamin-2	0.309	0.59	0.849	1.127	1.00	0.99	0.95	0.88	1
-	O14490	HUMAN Disks large-associated protein 1	-0.18	-0.36	0.568	0.677	0.99	1.01	0.98	0.96	1
Q969H4	I3LNS0	PIG Uncharacterized protein (Fragment) CNKSR1	-0.32	0.493	0.88	1.205	1.00	1.00	0.94	0.87	1
Q8WUP2	F1SUU4	PIG Uncharacterized protein FBLIM1	-0.54	-0.36	0.949	1.715	0.98	1.01	0.93	0.75	1
-	O15083	HUMAN ERC protein 2	0.884	0.661	1.135	1.185	0.96	0.99	0.90	0.87	1
-	F1S0Y1	PIG Uncharacterized protein (Fragment) HMCN2	1.049	0.699	0.657	0.971	0.95	0.99	0.98	0.92	2
-	Q6P9H4	HUMAN Connector enhancer of kinase suppressor of ras 3	0.567	1.552	1.445	0.445	0.98	0.71	0.78	0.99	1
-	Q9Y5S2	HUMAN Serine/threonine-protein kinase MRCK beta	-0.86	2.72	3.344	1.112	0.96	0.15	0.06	0.88	1
Q9Y3R0	K7GMX8	PIG Uncharacterized protein (Fragment) GRIP1	0.334	0.317	1.613	2.504	1.00	1.00	0.70	0.47	1
-	B0LFE9	PIG Complement component 4	1.503	1.43	1.345	1.384	0.83	0.76	0.82	0.83	38
-	Q08094	PIG Calponin-2 (Fragment)	0.23	0.635	1.793	2.466	1.00	0.99	0.64	0.48	2
Q7L576	F1RQE9	PIG Uncharacterized protein CYFIP2	2.245	2.004	1.618	1.196	0.44	0.45	0.70	0.87	2
-	F1SGT4	PIG Uncharacterized protein CD44	1.007	2.072	2.186	1.712	0.96	0.41	0.44	0.75	3

Human equivalent Ac. No.	Accession No.	Glycolysis<br>Protein	Zq (log2 FC)<br>120	1	4	7	FDR<br>120	1	4	7	No. peptides
-	Q1KYT0	PIG Beta-enolase	-2.2	-3.1	-2.23	-1.85	0.46	0.07	0.41	0.72	30
-	P29804	PIG Pyruvate dehydrogenase E1 component subunit alpha	-1.75	-3.05	-2.6	-2.13	0.72	0.07	0.24	0.59	22
P09104	I3LCN1	PIG Uncharacterized protein ENO2	-2.06	-2.89	-2.14	-1.72	0.54	0.10	0.45	0.75	3

-	B5KJG2	PIG Phosphoglycerate mutase 2	-2.09	-2.84	-1.87	-1.35	0.52	0.11	0.59	0.83	13
-	Q7SIB7	PIG Phosphoglycerate kinase 1	-2.58	-2.7	-1.17	-0.59	0.28	0.15	0.88	0.97	28
-	P11177	HUMAN Pyruvate dehydrogenase E1 component subunit beta, mitochondrial	-1.66	-1.82	-1.46	-1.15	0.76	0.55	0.76	0.87	1
-	Q9TQR6	PIG Phosphoglucomutase 1 (Fragment)	-0.98	-1.93	-1.33	-1.11	0.96	0.49	0.83	0.88	9
-	P08059	PIG Glucose-6-phosphate isomerase	-1.6	-2.26	-0.87	-0.44	0.78	0.32	0.94	0.99	19
-	K9IVI1	PIG 2-oxoglutarate dehydrogenase, mitochondrial	-0.81	-0.68	-1.26	-1.29	0.96	0.99	0.85	0.84	39
-	E5RI09	HUMAN Beta-enolase (Fragment)	-1.49	-0.5	-0.78	-0.49	0.83	1.00	0.96	0.98	1
-	P19367	HUMAN Hexokinase-1	-0.61	-0.62	-0.91	-1.06	0.99	0.99	0.94	0.90	1
P18669	F1S8Y5	PIG Uncharacterized protein PGAM1	-1.03	-1.63	-0.72	-0.32	0.95	0.66	0.97	0.99	7
-	P00355	PIG Glyceraldehyde-3-phosphate dehydrogenase	-1.37	-2.07	-0.81	-0.57	0.88	0.41	0.96	0.97	30
-	H9TUB4	PIG UME3A	-1.35	-0.79	-0.55	-0.5	0.89	0.98	0.98	0.98	1
-	C6KE31	PIG Transporter	-0.06	-0.32	-0.97	-1.02	1.00	1.00	0.93	0.91	1
-	B8XSK3	PIG 6-phosphofructo-2-kinase/fructose-2, 6-biphosphatase 1	-0.11	-0.71	-0.56	-0.48	1.00	0.99	0.98	0.98	1
-	A6NI74	HUMAN Enolase-like protein ENO4	-0.99	-1.28	0.031	0.449	0.96	0.85	1.00	0.99	1
-	Q6RI85	PIG Phosphoglycerate kinase 2	-0.72	0.613	0.245	-0.54	0.97	0.99	1.00	0.97	1
P19367	F1SUF2	PIG Uncharacterized protein (Fragment) HK1	-0.48	-0.32	-0.6	-0.72	0.99	1.00	0.98	0.96	23
-	G3CKJ2	PIG Glyceraldehyde-3-phosphate dehydrogenase (Fragment)	-0.25	-0.83	-0.48	-0.43	0.99	0.98	0.99	0.99	5
-	P06733	HUMAN Alpha-enolase	-1.53	-0.46	0.666	1.345	0.82	1.00	0.97	0.83	2
-	I3LCA1	PIG 6-phosphofructokinase, muscle type	0.192	-0.01	0.439	0.023	0.99	1.00	0.99	1.00	1
-	Q1W674	PIG Hexokinase-2	0.565	0.457	0.076	-0.57	0.98	1.00	1.00	0.97	6
P19367	I3LDQ9	PIG Uncharacterized protein LOC100737818	0.373	0.508	0.223	-0.37	1.00	1.00	1.00	0.99	3
-	I3LAK4	PIG Pyruvate kinase	0.69	0.297	0.091	0.193	0.98	1.00	1.00	0.99	1
Q01813	I3LK68	PIG Uncharacterized protein (Fragment) PFKP	0.045	-0.28	0.349	0.299	1.00	1.00	0.99	0.99	6
-	F1SUF3	PIG Uncharacterized protein (Fragment) HKDC1	0.637	0.327	-0.22	-0.59	0.98	1.00	1.00	0.97	1
-	P16118	HUMAN 6-phosphofructo-2-kinase/fructose-2,6-bisphosphatase 1	0.85	0.251	0.099	0.209	0.96	1.00	1.00	0.99	1
P17858	I3LFQ5	PIG Uncharacterized protein PFKL	0.365	0.155	0.493	0.504	1.00	1.00	0.99	0.98	10
-	P30613	HUMAN Pyruvate kinase PKLR	1.83	0.728	0.215	0.094	0.68	0.99	1.00	1.00	1

-	I3LK59	PIG Enolase	-0.15	0.689	1.599	1.865	0.99	0.99	0.71	0.71	15
-	P52789	HUMAN Hexokinase-2	0.373	1.98	2.064	0.932	1.00	0.46	0.49	0.92	1
-	P09104	HUMAN Gamma-enolase	1.483	0.928	0.881	-0.19	0.84	0.96	0.94	0.99	1

Kinase binding

Human equivalent Ac. No.	Accession No.	Protein	Zq (log2 FC)				FDR				No. peptides
			120	1	4	7	120	1	4	7	
-	P42771	HUMAN Cyclin-dependent kinase inhibitor 2A, isoforms 1/2/3	-2.45	-2.67	-1.57	-1.95	0.34	0.16	0.72	0.68	1
-	I3LNM6	PIG Uncharacterized protein CEP250	-2.61	-2.13	-2.01	-1.37	0.27	0.38	0.52	0.83	2
P25054	F1RLG3	PIG Uncharacterized protein (Fragment) APC	-0.6	-0.32	-1.64	-2.52	0.98	1.00	0.70	0.46	1
P30048	F1S418	PIG Uncharacterized protein PRDX3	-1.32	-0.61	-1.89	-2.15	0.90	0.99	0.57	0.58	8
-	Q96HC4	HUMAN PDZ and LIM domain protein 5	-1.48	-1.3	-1.8	-1.75	0.84	0.84	0.63	0.74	2
P30086	F1RKG8	PIG Uncharacterized protein PEBP1	-1.96	-2.17	-1.41	-1.13	0.60	0.37	0.79	0.88	9
-	B0FSM8	PIG Nucleotide-binding oligomerization domain containing 2	-1.48	-1.96	-1.14	-0.87	0.84	0.47	0.90	0.93	1
-	Q8WNW4	PIG Beta-catenin	-1.48	-1.78	-1.42	-1.11	0.84	0.57	0.79	0.88	19
-	Q03135	HUMAN Caveolin-1	-0.66	-1.44	-1.89	-1.92	0.98	0.75	0.57	0.69	1
-	P16066	HUMAN Atrial natriuretic peptide receptor 1	-0.8	-1.03	-1.39	-2.04	0.96	0.93	0.80	0.65	1
P19022	F1SAM3	PIG Uncharacterized protein (Fragment) CDH2	-0.63	-0.58	-1.06	-1.2	0.98	0.99	0.91	0.87	14
-	P36887	PIG cAMP-dependent protein kinase catalytic subunit alpha	-1.38	-2.15	-1.31	-0.59	0.87	0.38	0.83	0.97	10
Q96HC4	F1RWW4	PIG Uncharacterized protein PDLIM5	-1.07	-1.27	-1.22	-0.77	0.94	0.85	0.86	0.95	13
-	Q02224	HUMAN Centromere-associated protein E	-1.69	-2.22	-1.44	-0.72	0.75	0.35	0.78	0.96	1
-	Q6RVA9	PIG Caveolin-1	-1.6	-1.09	-1.11	-0.81	0.78	0.91	0.91	0.95	7
-	Q2EHH7	PIG Serine/threonine-protein phosphatase	-0.85	-1.26	-0.5	-0.02	0.96	0.85	0.99	1.00	3
-	F1S492	PIG Uncharacterized protein CDKN2D	-1.13	-1.1	-0.4	-0.63	0.93	0.90	0.99	0.96	1
P18669	F1S8Y5	PIG Uncharacterized protein PGAM1	-1.03	-1.63	-0.72	-0.32	0.95	0.66	0.97	0.99	7
-	Q969G5	HUMAN Protein kinase C delta-binding protein	-1.26	-0.44	-0.82	-0.81	0.91	1.00	0.96	0.95	1
-	P30048	HUMAN Thioredoxin-dependent peroxide reductase, mitochondrial	0.539	-0.66	-1.16	-1.13	0.98	0.99	0.88	0.88	1

-	Q3V6T2	HUMAN Girdin	-1.13	-0.27	-0.43	-0.96	0.93	1.00	1.00	0.92	1
-	Q2QLE2	PIG Caveolin-2	-1.4	-1.04	-0.93	-0.49	0.87	0.93	0.93	0.98	2
-	Q09138	PIG 5'-AMP-activated protein kinase subunit gamma-1	-0.22	-0.77	-0.85	-0.82	0.99	0.99	0.95	0.94	3
-	B8XSK3	PIG 6-phosphofructo-2-kinase/fructose-2, 6-biphosphatase 1	-0.11	-0.71	-0.56	-0.48	1.00	0.99	0.98	0.98	1
-	E7FLX9	PIG A kinase anchor protein 1	-0.99	-1.38	-1	-0.33	0.96	0.79	0.92	0.99	1
-	Q9BV73	HUMAN Centrosome-associated protein CEP250	1.09	-0.53	-0.58	-0.75	0.94	1.00	0.98	0.96	2
-	K7ELL7	HUMAN Glucosidase 2 subunit beta	-0.52	0.327	-0.14	-0.23	0.99	1.00	1.00	0.99	1
Q9UEY8	F1S5M9	PIG Uncharacterized protein ADD3	0.108	-0.69	-0.71	-0.2	1.00	0.99	0.97	0.99	6
Q12959	K7GN86	PIG Uncharacterized protein DLG1	-0.93	-0.19	0.559	0.106	0.96	1.00	0.98	1.00	1
-	I3LR17	PIG Uncharacterized protein CORO1A	-0.46	-1.08	-0.95	0.639	0.99	0.91	0.93	0.96	1
Q96EY1	I3LLG4	PIG Uncharacterized protein DNAJA3	0.51	0.587	-0.07	-0.87	0.99	0.99	1.00	0.93	3
P42773	F1S6F7	PIG Uncharacterized protein CDKN2C	0.356	-0.46	-0.16	0.267	0.99	1.00	1.00	0.99	1
-	F8WDM7	HUMAN Caveolin-1	0.136	-0.19	0.03	0.123	0.99	1.00	1.00	1.00	1
-	I3LCA1	PIG 6-phosphofructokinase, muscle type	0.192	-0.01	0.439	0.023	0.99	1.00	0.99	1.00	1
-	F1SFW3	PIG Guanylate cyclase (Fragment)	0.397	0.082	0.089	-0.18	0.99	1.00	1.00	0.99	1
-	P16118	HUMAN 6-phosphofructo-2-kinase/fructose-2,6-bisphosphatase 1	0.85	0.251	0.099	0.209	0.96	1.00	1.00	0.99	1
P17858	I3LFQ5	PIG Uncharacterized protein PFKL	0.365	0.155	0.493	0.504	1.00	1.00	0.99	0.98	10
-	Q13370	HUMAN cGMP-inhibited 3',5'-cyclic phosphodiesterase B	0.39	0.343	-0.33	-0.26	0.99	1.01	0.99	0.99	2
Q969G5	F1RMM0	PIG Uncharacterized protein PRKCDBP	-0.26	-0.2	0.019	0.703	0.99	1.00	1.00	0.96	3
P14314	F1S596	PIG Uncharacterized protein PRKCSH	-0.14	0.7	0.824	1.048	0.99	0.99	0.95	0.90	10
O43741	F1SDB6	PIG Uncharacterized protein PRKAB2	1.995	0.459	0.195	0.068	0.58	1.00	1.00	1.00	1
-	Q95319	PIG Beta-actin, cytoplasmic (Fragment)	0.124	0.107	0.153	0.997	0.99	1.00	1.00	0.92	5
-	B5LX40	PIG Adaptor protein phosphotyrosine interaction PH domain and leucine zipper	1.024	0.083	0.162	0.266	0.95	1.00	1.00	0.99	3
-	F1RWH6	PIG Uncharacterized protein CDK5RAP3	1.19	1.244	0.003	-0.02	0.93	0.86	1.00	1.00	1
-	P31146	HUMAN Coronin-1A	-0.49	0.502	0.76	0.4	0.99	1.00	0.97	0.99	1
-	P56945	HUMAN Breast cancer anti-estrogen resistance protein 1	1.247	0.416	0.58	0.343	0.91	1.01	0.98	0.99	1
-	Q7M3B0	PIG Actin beta (Fragments)	0.213	0.349	0.823	0.926	0.99	1.01	0.96	0.92	1

-	Q2M2Z5	HUMAN Centrosomal protein kizuna	0.935	3.757	3.059	-0.27	0.96	0.01	0.11	0.99	1
-	P60709	HUMAN Actin, cytoplasmic 1	0.706	1.257	2.826	4.524	0.98	0.85	0.17	0.01	1

Human equivalent Ac. No.	Accession No.	Alcohol biosynthetic process<br><br>Protein	Zq (log2 FC)<br>120	 1	 4	 7	FDR<br>120	 1	 4	 7	No. peptides
-	P00503	PIG Aspartate aminotransferase, cytoplasmic	-2.06	-3.05	-2.38	-1.86	0.55	0.07	0.33	0.71	24
Q8TBE9	F1SAS1	PIG Uncharacterized protein NANP	-2.61	-1.98	-1.94	-1.52	0.27	0.46	0.55	0.81	1
P24298	F1RSP5	PIG Uncharacterized protein LOC100524618	-1.46	-1.28	-1.73	-1.91	0.84	0.84	0.66	0.69	8
Q8N335	F1SHA2	PIG Uncharacterized protein (Fragment) GPD1	-1.47	-2.34	-2.11	-1.61	0.84	0.28	0.47	0.80	3
P09104	I3LCN1	PIG Uncharacterized protein ENO2	-2.06	-2.89	-2.14	-1.72	0.54	0.10	0.45	0.75	3
-	B5KJG2	PIG Phosphoglycerate mutase 2	-2.09	-2.84	-1.87	-1.35	0.52	0.11	0.59	0.83	13
-	Q9TQR6	PIG Phosphoglucomutase 1 (Fragment)	-0.98	-1.93	-1.33	-1.11	0.96	0.49	0.83	0.88	9
-	P08059	PIG Glucose-6-phosphate isomerase	-1.6	-2.26	-0.87	-0.44	0.78	0.32	0.94	0.99	19
-	B8XSK3	PIG 6-phosphofructo-2-kinase/fructose-2, 6-biphosphatase 1	-0.11	-0.71	-0.56	-0.48	1.00	0.99	0.98	0.98	1
-	P00636	PIG Fructose-1,6-bisphosphatase 1	0.807	-0.06	-0.5	-0.48	0.96	1.00	0.99	0.98	2
-	F1RQM2	PIG Phosphoacetylglucosamine mutase	0.842	-0.01	0.122	0.121	0.96	1.00	1.00	1.00	4
O15305	I3LCY2	PIG Uncharacterized protein	0.415	0.018	0.635	0.843	0.99	1.00	0.98	0.94	2
-	P16118	HUMAN 6-phosphofructo-2-kinase/fructose-2,6-bisphosphatase 1	0.85	0.251	0.099	0.209	0.96	1.00	1.00	0.99	1
-	P14332	PIG 6-phosphogluconate dehydrogenase, decarboxylating (Fragment)	0.434	0.709	1.076	1.149	0.99	0.99	0.91	0.87	7
-	I3L677	PIG Glucose-6-phosphate 1-dehydrogenase (Fragment)	0.214	0.979	1.279	1.101	0.99	0.95	0.85	0.89	12
-	F1RIF8	PIG 6-phosphogluconate dehydrogenase, decarboxylating (Fragment)	-0.03	1.046	1.196	1.371	1.00	0.93	0.87	0.83	7
-	P09104	HUMAN Gamma-enolase	1.483	0.928	0.881	-0.19	0.84	0.96	0.94	0.99	1

Supplementary Table S3. Protein expression changes in the functionally annotated categories within cluster c.

Human equivalent Ac. No.	Accession No.	Ribosome<br>Protein	Zq (log2 FC) 120	1	4	7	FDR 120	1	4	7	No. peptides
-	P67985	PIG 60S ribosomal protein L22	0.962	1.526	1.667	1.23	0.95	0.71	0.69	0.86	3
-	Q29293	PIG 60S ribosomal protein L3 (Fragment)	-0.92	0.798	1.9	1.557	0.96	0.99	0.57	0.80	1
-	P63173	HUMAN 60S ribosomal protein L38	-0.02	0.314	1.247	2.006	1.00	1.00	0.85	0.66	5
-	P23396	HUMAN 40S ribosomal protein S3	0.775	0.477	1.526	1.892	0.97	1.00	0.74	0.69	6
-	Q29308	PIG 40S ribosomal protein S19 (Fragment)	0.15	0.615	1.669	1.796	0.99	0.99	0.69	0.73	9
-	P62244	HUMAN 40S ribosomal protein S15a	0.611	0.263	1.39	1.266	0.99	1.00	0.80	0.85	4
-	P62910	HUMAN 60S ribosomal protein L32	-0.14	1.263	2.247	1.359	0.99	0.85	0.41	0.83	2
-	P62913	HUMAN 60S ribosomal protein L11	0.825	-0.16	0.625	2.031	0.96	1.00	0.98	0.65	1
-	Q9Y3U8	HUMAN 60S ribosomal protein L36	1.171	0.71	1.123	0.946	0.93	0.99	0.90	0.92	3
-	P53027	PIG 60S ribosomal protein L10a (Fragment)	-0.25	-0.25	1.334	1.878	0.99	1.00	0.83	0.70	7
P46776	I3LK51	PIG Uncharacterized protein (Fragment)	0.649	0.209	1.028	1.475	0.98	1.00	0.92	0.81	3
-	I3LEX0	PIG 40S ribosomal protein S9 (Fragment)	-0.3	-0.64	1.234	1.835	1.00	0.99	0.86	0.72	5
-	P62280	HUMAN 40S ribosomal protein S11	-0.57	-0.38	1.055	1.885	0.98	1.01	0.91	0.70	7
-	A1XQU7	PIG 60S acidic ribosomal protein P1	-0.05	-0.02	1.631	1.751	1.00	1.00	0.70	0.74	1
P62847	F1SNB6	PIG Uncharacterized protein LOC100515348	1.511	0.812	0.711	1.036	0.83	0.98	0.97	0.91	1
-	F2Z5F5	PIG 40S ribosomal protein S8 (Fragment)	-0.1	0.113	0.989	1.323	1.00	1.00	0.92	0.83	8
-	Q29197	PIG 40S ribosomal protein S9 (Fragment)	-0.18	-0.58	1.051	1.469	0.99	0.99	0.91	0.81	10
-	P62279	PIG 40S ribosomal protein S13 (Fragment)	0.043	-0.05	1.121	1.42	1.00	1.00	0.90	0.82	6
-	Q2YGT9	PIG 60S ribosomal protein L6	-0.05	-0.13	0.953	1.573	1.00	1.00	0.93	0.80	14
-	F6Q5P0	PIG 40S ribosomal protein S13	0.112	0.227	0.795	0.564	1.00	1.00	0.96	0.98	3
-	I3LVI8	PIG 60S ribosomal protein L7a	-0.09	0.592	0.87	1.42	1.00	0.99	0.94	0.82	3

-	Q29205	PIG 60S ribosomal protein L11	-0.33	-0.52	0.755	1.307	1.00	1.00	0.97	0.84	4
-	Q4GWZ2	PIG 40S ribosomal protein SA	0.322	0.154	1.18	1.259	1.00	1.00	0.88	0.85	8
-	F1SUM7	PIG 40S ribosomal protein S3 (Fragment)	-0.12	-0.39	0.832	1.409	0.99	1.00	0.95	0.82	10
-	P32969	HUMAN 60S ribosomal protein L9	-0.4	-0.19	0.873	1.39	0.99	1.00	0.94	0.83	7
-	F1SJJ5	PIG 60S ribosomal protein L4	-0.13	0.181	1.224	1.646	0.99	1.00	0.86	0.78	11
-	I3LS62	PIG Ribosomal protein L15	-0.73	-0.97	0.77	1.557	0.97	0.94	0.97	0.80	3
P62910	F1SPL8	PIG Uncharacterized protein (Fragment)	0.885	0.348	0.578	0.666	0.96	1.01	0.98	0.96	1
-	P61353	HUMAN 60S ribosomal protein L27	0.599	0.352	0.676	0.789	0.99	1.01	0.97	0.95	1
P62899	F1SH66	PIG Uncharacterized protein LOC100625352	0.135	-0.11	0.716	1.657	0.99	1.00	0.97	0.78	1
-	P62424	HUMAN 60S ribosomal protein L7a	-0.41	-0.25	1.278	1.765	0.99	1.00	0.85	0.74	5
-	B7TJ03	PIG Ribosomal protein L26-like 1	-0.59	-0.46	0.657	1.247	0.99	1.00	0.98	0.86	5
-	Q29375	PIG 60S ribosomal protein L7a (Fragment)	-0.1	-0.52	1.109	1.767	1.00	1.00	0.90	0.74	4
-	F8W7C6	HUMAN 60S ribosomal protein L10	1.102	1.301	1.111	0.104	0.94	0.83	0.91	1.00	1
P62249	K7GLK5	PIG Uncharacterized protein	0.107	0.336	0.647	1.02	1.00	1.01	0.98	0.91	4
-	Q29198	PIG Ribosomal protein S6 (Fragment)	0.738	0.065	0.161	0.627	0.97	1.00	1.00	0.96	3
-	Q95307	PIG 60S ribosomal protein L13a (Fragment)	0.113	-0.68	0.619	1.092	0.99	0.99	0.98	0.89	7
-	Q29214	PIG 60S acidic ribosomal protein P0	-0.23	-0.1	1.022	1.459	0.99	1.00	0.92	0.82	12
-	F1S4M2	PIG Uncharacterized protein (Fragment) LOC100738304	-0.24	0.085	0.679	1.202	0.99	1.00	0.97	0.87	6
P18124	F1RWI5	PIG Uncharacterized protein	-0.28	-0.73	0.889	1.336	0.99	0.99	0.94	0.83	13
-	P62899	HUMAN 60S ribosomal protein L31	0.208	0.421	0.582	1.187	0.99	1.01	0.98	0.87	1
-	Q6QAP7	PIG 40S ribosomal protein S17	-0.42	-0.53	0.662	1.114	0.99	1.00	0.98	0.88	6
-	P62750	HUMAN 60S ribosomal protein L23a	-0.15	-0.13	0.656	1.437	0.99	1.00	0.98	0.82	9
-	P62081	HUMAN 40S ribosomal protein S7	-0.17	-0.33	0.606	1.215	0.99	1.01	0.98	0.87	2
-	Q6QAS5	PIG 60S ribosomal protein L12 (Fragment)	-0.13	-0.3	0.868	1.315	0.99	1.00	0.95	0.84	5
P46783	F1RZ28	PIG Uncharacterized protein RPS10	-0.96	-0.03	0.658	1.24	0.95	1.00	0.98	0.85	5
-	P62829	HUMAN 60S ribosomal protein L23	-0.33	-0.71	0.657	1.534	1.00	0.99	0.98	0.80	5
-	P39019	HUMAN 40S ribosomal protein S19	0.092	0.639	0.975	0.75	1.00	0.99	0.93	0.96	1

-	P62249	HUMAN 40S ribosomal protein S16	0.02	-0.5	0.477	1.012	1.00	1.00	0.99	0.91	2
-	P61254	HUMAN 60S ribosomal protein L26	-1.08	-0.78	0.828	1.855	0.94	0.98	0.95	0.72	1
P83731	F2Z5Q2	PIG Uncharacterized protein (Fragment) RPL24	0.379	-0.02	0.708	1.725	1.00	1.00	0.97	0.75	6
-	P79324	PIG 60S ribosomal protein L15 (Fragment)	-0.73	-0.47	0.67	1.409	0.97	1.00	0.97	0.82	6
-	P49666	PIG 60S ribosomal protein L21 (Fragment)	-0.7	-0.58	0.726	1.28	0.98	0.99	0.97	0.85	3
-	I3LSD3	PIG 60S ribosomal protein L13	-0.72	-0.59	0.645	1.304	0.97	0.99	0.98	0.84	9
-	P62269	HUMAN 40S ribosomal protein S18	-0.64	-0.21	0.95	1.355	0.98	1.00	0.93	0.83	7
-	Q5T7N0	HUMAN 60S ribosomal protein L5 (Fragment)	-1.03	-0.29	0.733	1.486	0.95	1.00	0.97	0.81	1
-	I3LFL4	PIG 60S ribosomal protein L18 (Fragment)	-0.12	-0.51	0.308	1.141	0.99	1.00	1.00	0.88	1
-	P62841	HUMAN 40S ribosomal protein S15	-0.98	-0.04	0.834	1.37	0.96	1.00	0.96	0.83	3
P62899	F1SRB7	PIG Uncharacterized protein (Fragment) LOC100624537	-0.25	0.198	0.665	0.883	0.99	1.00	0.97	0.93	1
-	F1RI01	PIG 60S ribosomal protein L13a	0.017	-0.58	0.515	0.995	1.00	0.99	0.99	0.92	4
-	B6V8C8	PIG 40S ribosomal protein S3a	-0.83	-0.65	0.733	1.243	0.96	0.99	0.97	0.86	11
P62910	I3LV37	PIG Uncharacterized protein (Fragment)	-0.52	-0.64	0.576	0.974	0.99	0.99	0.98	0.92	1
-	Q29195	PIG 60S ribosomal protein L10	-0.34	-0.38	0.817	1.437	1.00	1.01	0.96	0.82	5
-	P46778	HUMAN 60S ribosomal protein L21	-0.1	-0.35	0.313	0.864	1.00	1.01	1.00	0.94	1
-	Q29223	PIG 60S ribosomal protein L34	0.276	-0.44	0.426	0.957	0.99	1.01	1.00	0.92	3
-	Q6QAQ3	PIG 60S ribosomal protein L23 (Fragment)	0.23	0.382	0.391	0.388	0.99	1.01	1.00	0.99	1
-	Q6QAP6	PIG 40S ribosomal protein S29	-0.97	-1.34	0.407	1.252	0.96	0.81	1.00	0.85	2
-	F1SJA1	PIG 40S ribosomal protein S6	-0.4	-0.69	0.005	0.573	0.99	0.99	1.00	0.97	1
-	Q29190	PIG Ribosomal protein S7 (Fragment)	-0.27	-0.64	0.248	0.952	0.99	0.99	1.00	0.92	2
-	P25398	HUMAN 40S ribosomal protein S12	0.013	-0.18	0.535	0.668	1.00	1.00	0.98	0.96	2
-	Q95L19	PIG Ribosomal protein L19 (Fragment)	-0.38	-0.6	0.463	0.702	0.99	0.99	0.99	0.96	5
-	Q95342	PIG 60S ribosomal protein L18 (Fragment)	-0.18	-0.59	0.261	0.799	0.99	0.99	1.00	0.95	4
-	P46782	HUMAN 40S ribosomal protein S5	-0.28	-0.28	0.402	0.676	0.99	1.00	1.00	0.96	6
-	Q6QAS9	PIG 60S ribosomal protein L7 (Fragment)	-1.26	-1.31	0.543	1.409	0.91	0.83	0.98	0.82	3
-	Q29361	PIG 60S ribosomal protein L35	-1.21	-0.84	0.638	1.505	0.92	0.98	0.98	0.81	6

-	Q95281	PIG 60S ribosomal protein L29	-1.09	-0.87	0.313	1.402	0.94	0.97	1.00	0.82	3
-	K7ELC7	HUMAN 60S ribosomal protein L27 (Fragment)	0.08	-0.92	-0.42	-0.09	1.00	0.96	1.00	1.00	1
P05386	F1SIT7	PIG Uncharacterized protein LOC100523874	-0.16	0.073	1.486	1.427	0.99	1.00	0.75	0.82	1
-	F1S530	PIG 60S ribosomal protein L5	-1.05	-0.55	0.586	0.874	0.95	0.99	0.98	0.93	7
P62851	F2Z5G8	PIG Uncharacterized protein (Fragment) RPS25	-0.52	-0.67	0.335	0.988	0.99	0.99	0.99	0.92	7
-	P49171	PIG 40S ribosomal protein S26	-0.25	-0.46	0.175	0.729	0.99	1.00	1.00	0.96	2
P46779	I3LSZ6	PIG Uncharacterized protein RPL28	-0.73	-0.24	0.264	0.615	0.97	1.00	1.00	0.97	1
-	F1RYZ0	PIG 60S acidic ribosomal protein P2	0.086	0.249	0.226	0.305	1.00	1.00	1.00	0.99	4
-	Q29323	PIG Ribosomal protein S5 (Fragment)	-1.08	-1.07	0.244	0.669	0.94	0.92	1.00	0.96	1
-	Q6SA96	PIG 40S ribosomal protein S23	-1.29	-1.05	0.177	1.264	0.91	0.93	1.00	0.85	6
P83881	F1RT21	PIG Uncharacterized protein	-0.35	-0.03	-0.41	0.551	1.00	1.00	1.00	0.98	2
P83881	I3LHT5	PIG Uncharacterized protein	-0.86	-0.92	-0.44	0.296	0.96	0.96	0.99	0.99	1
P62249	F1SP46	PIG Uncharacterized protein	-1.15	-1.11	0.116	0.901	0.93	0.90	1.00	0.92	5
-	Q29187	PIG 60S ribosomal protein L4 (Fragment)	-2.22	-1.49	0.317	2.127	0.46	0.73	1.00	0.60	1
-	A1XQU9	PIG 40S ribosomal protein S20	-0.56	-0.91	-0.06	0.385	0.98	0.96	1.00	0.99	3
P62081	I3L7B0	PIG Uncharacterized protein (Fragment)	-1.04	-0.64	0.196	0.847	0.95	0.99	1.00	0.94	2
P62244	F1RZH4	PIG Uncharacterized protein	-0.64	-0.32	-0.2	-0.52	0.98	1.00	1.00	0.98	1
P18621	I3LT81	PIG Uncharacterized protein RPL17	-1.2	-1.11	0.246	1.44	0.93	0.90	1.00	0.82	6
-	P83881	HUMAN 60S ribosomal protein L36a	-1.8	-2.08	-0.31	1.97	0.69	0.40	1.00	0.67	1
-	I3L6F1	PIG 60S ribosomal protein L18	-1.48	-1.88	-0.13	1.091	0.84	0.52	1.00	0.89	1
-	P63220	HUMAN 40S ribosomal protein S21	0.085	0.03	-0.43	-0.66	1.00	1.00	0.99	0.96	1
-	A1XQU3	PIG 60S ribosomal protein L14	-1.62	-1.08	-0.02	0.618	0.77	0.91	1.00	0.97	4
-	P61513	HUMAN 60S ribosomal protein L37a	-0.94	-1.1	-0.35	-0.15	0.96	0.90	0.99	1.00	1
-	Q29315	PIG 60S acidic ribosomal protein P2	0.368	0.459	-0.04	-0.14	0.99	1.00	1.00	1.00	3
-	P62888	HUMAN 60S ribosomal protein L30	-0.82	-1.05	-0.33	0.102	0.96	0.93	1.00	1.00	4
-	P62857	HUMAN 40S ribosomal protein S28	-0.46	-0.86	-0.25	0.024	0.99	0.97	1.00	1.00	2
-	F1SML8	PIG 40S ribosomal protein S6	-1.76	-2.8	-0.71	1.265	0.71	0.12	0.97	0.85	2

P62899	F1SLI2	PIG Uncharacterized protein (Fragment)	-1.13	-0.99	-0.09	0.639	0.93	0.94	1.00	0.96	1
-	P46779	HUMAN 60S ribosomal protein L28	-1.29	-1.89	-0.38	0.557	0.91	0.51	0.99	0.98	3
P02549	F1RR78	PIG Uncharacterized protein LOC100049693	-0.89	-1.12	-0.83	-0.49	0.96	0.90	0.95	0.98	148
-	F2Z514	PIG 40S ribosomal protein S27 (Fragment)	-1	-0.87	-0.7	-0.43	0.96	0.97	0.97	0.99	3

Human equivalent Ac. No.	Accession No.	Acute renal failure panel<br>Protein	Zq (log2 FC)<br>120	1	4	7	FDR<br>120	1	4	7	No. peptides
P02675	I3L651	PIG Uncharacterized protein	1.292	2.457	3.366	3.096	0.91	0.24	0.05	0.23	27
-	P19619	PIG Annexin A1	0.759	3.181	3.067	2.43	0.97	0.05	0.11	0.49	16
-	F1RRX1	PIG Uncharacterized protein LCN2	1.672	3.573	2.143	0.034	0.76	0.02	0.45	1.00	5
P43652	F1RUM1	PIG Uncharacterized protein AFM	3.394	2.507	1.493	0.942	0.05	0.22	0.75	0.92	8
P01023	I3LQ17	PIG Uncharacterized protein	2.016	1.788	2.079	1.597	0.57	0.57	0.48	0.80	50
-	F1SGT4	PIG Uncharacterized protein CD44	1.007	2.072	2.186	1.712	0.96	0.41	0.44	0.75	3
-	A2SW51	PIG Monocyte differentiation antigen CD14	1.601	2.47	1.714	1.165	0.78	0.23	0.67	0.87	2
-	P14477	PIG Fibrinogen beta chain (Fragment)	-0.18	1.518	1.03	1.911	0.99	0.72	0.92	0.69	1
-	P00339	PIG L-lactate dehydrogenase A chain	-0.15	-0.05	1.977	2.197	0.99	1.00	0.53	0.58	19
P02452	I3LUM2	PIG Uncharacterized protein	-1.39	0.987	0.66	2.153	0.87	0.94	0.98	0.58	2
-	K9J6H8	PIG Alpha-2-macroglobulin	1.728	1.975	1.262	-0.11	0.73	0.47	0.85	1.00	3
-	P19620	PIG Annexin A2	-0.12	0.419	1.155	1.75	0.99	1.00	0.89	0.74	32
-	A3EX84	PIG Galectin	-0.59	0.06	1.552	2.42	0.99	1.00	0.73	0.50	4
-	P32394	PIG Heme oxygenase 1	0.774	0.663	1.096	1.001	0.97	0.99	0.90	0.92	7
-	P02675	HUMAN Fibrinogen beta chain	3.779	0.632	-0.02	-0.17	0.02	0.99	1.00	0.99	2
P02461	F1RYI8	PIG Uncharacterized protein COL3A1	-0	0.042	1.021	1.81	1.00	1.00	0.92	0.73	7
-	P01100	HUMAN Proto-oncogene c-Fos	0.723	0.362	0.213	0.692	0.97	1.01	1.00	0.96	1
-	P02452	HUMAN Collagen alpha-1(I) chain	-0.75	0.326	0.6	1.108	0.97	1.00	0.98	0.88	8
Q01995	F1SJS8	PIG Uncharacterized protein TAGLN	-0.71	-0.4	0.822	2.502	0.98	1.01	0.96	0.47	23

-	P02461	HUMAN Collagen alpha-1(III) chain	-1.32	0.97	0.332	1.023	0.90	0.95	0.99	0.91	2
-	P07437	HUMAN Tubulin beta chain	0.055	-0.02	0.858	1.619	1.00	1.00	0.95	0.79	4
-	Q99988	HUMAN Growth/differentiation factor 15	1.457	0.62	-0.38	-0.32	0.85	0.99	0.99	0.99	1
Q02952	F1S7W0	PIG Uncharacterized protein (Fragment) AKAP12	0.815	0.327	0.062	0.743	0.96	1.00	1.00	0.96	12
-	F1SKD6	PIG Uncharacterized protein SLC16A7	-1.88	-1.62	-0.93	0.443	0.64	0.66	0.93	0.99	1
-	Q6PQZ1	PIG Aquaporin-1	0.229	0.108	-0.12	-0.02	0.99	1.00	1.00	1.00	2
-	Q02952	HUMAN A-kinase anchor protein 12	0.11	-0.31	0.073	0.937	1.00	1.00	1.00	0.92	1
P55042	I3L820	PIG Uncharacterized protein (Fragment) RRAD	-0.32	-0.77	-0.13	-0.9	1.00	0.98	1.00	0.93	2
P02452	I3LJX2	PIG Uncharacterized protein	-0.76	-1.23	-0.48	0.464	0.97	0.86	0.99	0.98	2
-	Q2VTP6	PIG Peptidyl-prolyl cis-trans isomerase	-0.73	-0.74	-0.51	-0.38	0.97	0.99	0.99	0.99	2
P07305	I3LNZ2	PIG Uncharacterized protein LOC100510904	-0.84	-0.58	-1.28	-0.9	0.96	0.99	0.85	0.92	4
-	P12429	HUMAN Annexin A3	-1.06	-0.99	-1.35	-1.25	0.95	0.94	0.82	0.86	2

Human equivalent Ac. No.	Accession No.	Extracellular matrix<br>Protein	Zq (log2 FC) 120	1	4	7	FDR 120	1	4	7	No. peptides
-	P48819	PIG Vitronectin	1.499	2.251	3.536	2.449	0.83	0.33	0.03	0.48	11
-	O19112	PIG Cartilage intermediate layer protein 1 (Fragment)	2.161	1.223	1.266	2.143	0.49	0.86	0.85	0.58	2
-	F1SGT4	PIG Uncharacterized protein CD44	1.007	2.072	2.186	1.712	0.96	0.41	0.44	0.75	3
P49747	F1S902	PIG Uncharacterized protein COMP	-0.2	0.34	1.825	3.091	0.99	1.01	0.61	0.23	1
-	F1RQI2	PIG Uncharacterized protein COL12A1	0.621	1.007	1.711	3.067	0.98	0.93	0.67	0.24	1
-	Q95285	PIG Collagen alpha 1 (XV) chain (Fragment)	0.803	1.541	1.506	1.639	0.96	0.71	0.75	0.78	1
-	P08123	HUMAN Collagen alpha-2(I) chain	-1.13	1.075	0.496	1.511	0.93	0.92	0.99	0.81	3
P02452	I3LUM2	PIG Uncharacterized protein	-1.39	0.987	0.66	2.153	0.87	0.94	0.98	0.58	2
-	F1SKM1	PIG Uncharacterized protein COL7A1	0.651	1.58	1.918	1.192	0.98	0.68	0.56	0.87	3
-	O00339	HUMAN Matrilin-2	1.314	0.856	0.444	-0.18	0.90	0.97	0.99	0.99	1
Q9H306	F1SV70	PIG Uncharacterized protein MMP27	1.117	1.5	1.747	0.825	0.93	0.72	0.65	0.94	1

P08572	F1RLL9	PIG Uncharacterized protein (Fragment) COL4A2	0.287	0.465	1.047	1.407	1.00	1.00	0.91	0.82	4
-	P08572	HUMAN Collagen alpha-2(IV) chain	2.153	0.896	0.05	1.243	0.49	0.97	1.00	0.86	1
-	P45846	PIG Dermatopontin	1.028	1.087	0.624	1.418	0.95	0.91	0.98	0.82	2
-	Q28833	PIG von Willebrand factor (Fragment)	0.779	1.131	0.987	0.846	0.97	0.90	0.92	0.94	7
-	A5D9K7	PIG Collagen type XI alpha 2	1.002	1.471	0.935	0.465	0.96	0.74	0.93	0.98	2
P02461	F1RYI8	PIG Uncharacterized protein COL3A1	-0	0.042	1.021	1.81	1.00	1.00	0.92	0.73	7
-	Q99542	HUMAN Matrix metalloproteinase-19	3.655	1.055	-0.02	0.373	0.02	0.93	1.00	0.99	1
P20908	F1S021	PIG Uncharacterized protein (Fragment) COL5A1	-0.29	0.554	1.014	1.412	1.00	0.99	0.91	0.82	5
-	F1SLT8	PIG Uncharacterized protein CCDC80	1.632	0.932	0.425	0.42	0.77	0.96	1.00	0.99	1
P13611	F1SX62	PIG Uncharacterized protein VCAN	0.219	0.314	0.867	1.393	0.99	1.00	0.94	0.83	10
-	Q99715	HUMAN Collagen alpha-1(XII) chain	0.605	0.266	0.62	1.264	0.99	1.00	0.98	0.85	17
-	Q59IP1	PIG Procollagen alpha 3(V)	-0.11	0.704	1.016	1.68	1.00	0.99	0.91	0.77	3
-	F1SV48	PIG Uncharacterized protein COL8A2	0.82	0.434	0.522	0.767	0.96	1.01	0.99	0.95	1
-	P02452	HUMAN Collagen alpha-1(I) chain	-0.75	0.326	0.6	1.108	0.97	1.00	0.98	0.88	8
-	I3L8B2	PIG Uncharacterized protein (Fragment) COL9A2	-0.73	3.018	3.035	1.083	0.97	0.08	0.11	0.89	1
-	P02461	HUMAN Collagen alpha-1(III) chain	-1.32	0.97	0.332	1.023	0.90	0.95	0.99	0.91	2
-	P12107	HUMAN Collagen alpha-1(XI) chain	0.491	-0.12	0.759	1.185	0.99	1.00	0.97	0.87	2
P51884	F1SQ09	PIG Uncharacterized protein LUM	0.069	0.596	0.417	1.329	1.00	0.99	1.00	0.83	12
O75718	I3L6R1	PIG Uncharacterized protein CRTAP	0.822	0.673	0.548	1.056	0.96	0.99	0.98	0.90	2
-	F1SSE7	PIG Uncharacterized protein (Fragment) COL15A1	0.657	0.687	-0.22	0.311	0.98	0.99	1.00	0.99	3
-	Q1T7A9	PIG Type VI collagen alpha-1 chain (Fragment)	0.335	0.209	0.108	0.745	1.00	1.00	1.00	0.96	2
P08123	F1SFA7	PIG Uncharacterized protein COL1A2	-0.82	0.537	-0.03	1.099	0.96	0.99	1.00	0.89	11
Q99715	F1RQI0	PIG Uncharacterized protein COL12A1	-0.13	-0.03	0.516	1.35	0.99	1.00	0.99	0.83	6
-	Q1T7A5	PIG Type VI collagen alpha-3 chain (Fragment)	-0.81	-0.48	0.112	1.221	0.96	1.00	1.00	0.86	16
-	P20849	HUMAN Collagen alpha-1(IX) chain	0.445	0.23	0.25	0.101	0.99	1.00	1.00	1.00	1
-	P02458	HUMAN Collagen alpha-1(II) chain	0.283	0.629	-0.17	0.104	0.99	0.99	1.00	1.00	2
P02458	I3LSV6	PIG Uncharacterized protein (Fragment) COL2A1	-0.72	-0.21	-0.1	0.595	0.97	1.00	1.00	0.97	1

Q05707	K7GT00	PIG Uncharacterized protein (Fragment) COL14A1	0.074	0.045	-0.1	0.418	1.00	1.00	1.00	0.99	20
-	P25940	HUMAN Collagen alpha-3(V) chain	-0.21	0.189	-0.24	0.891	0.99	1.00	1.00	0.93	1
P39059	I3LIL7	PIG Uncharacterized protein LOC100620394	0.011	0.088	0.115	0.719	1.00	1.00	1.00	0.96	6
-	P42099	PIG Zona pellucida sperm-binding protein 2	0.39	0.432	-0.15	-0.05	0.99	1.01	1.00	1.00	1
-	F1S405	PIG Uncharacterized protein LTBP1	0.009	0.035	0.077	0.316	1.00	1.00	1.00	0.99	1
-	Q03001	HUMAN Dystonin	0.408	0.263	0.287	0.447	0.99	1.00	1.00	0.99	3
-	F1S2T5	PIG Uncharacterized protein LTBP2	-1.01	-0.77	0.214	1.022	0.96	0.98	1.00	0.91	1
-	F1SFM3	PIG Uncharacterized protein (Fragment) CHL1	1.209	-0.11	0.079	-0.14	0.92	1.00	1.00	1.00	1
-	O75718	HUMAN Cartilage-associated protein	0.178	1.32	0.189	0.541	0.99	0.83	1.00	0.97	1
-	Q9NQ76	HUMAN Matrix extracellular phosphoglycoprotein	-1.05	0.555	0.883	1.355	0.94	0.99	0.94	0.83	1
-	P12109	HUMAN Collagen alpha-1(VI) chain	-1.9	-1.16	-0.27	1.41	0.63	0.89	1.00	0.82	6
-	Q02388	HUMAN Collagen alpha-1(VII) chain	0.482	0.13	0.039	-0.14	0.98	1.00	1.00	1.00	4
-	F1SEN6	PIG Uncharacterized protein MMRN2	1.369	0.263	-0.35	-0.26	0.88	1.00	0.99	0.99	6
P12109	I3LS72	PIG Uncharacterized protein COL6A1	-1.53	-1.43	-0.36	1.474	0.81	0.76	1.00	0.81	10
-	Q59IP2	PIG Procollagen alpha 2(V)	-0.07	-0.44	0.053	0.596	1.00	1.00	1.00	0.97	3
P12110	I3LQ84	PIG Uncharacterized protein	-1.43	-1.24	-0.48	1.033	0.86	0.86	0.99	0.91	21
-	P51884	HUMAN Lumican	0.942	-0.23	-0.36	-0.38	0.96	1.00	0.99	0.99	1
-	Q9UMD9	HUMAN Collagen alpha-1(XVII) chain	-1.29	-0.25	0.153	-1.01	0.91	1.00	1.00	0.91	1
-	P49923	PIG Lipoprotein lipase	0.312	0.327	-0.4	-0.77	1.00	1.00	1.00	0.95	1
-	Q29243	PIG Dystroglycan (Fragment)	-0.36	0.64	-0.11	-0.17	1.00	0.99	1.00	1.00	6
Q13361	F1SLW6	PIG Uncharacterized protein MFAP5	-1.29	-0.99	-0.71	0.227	0.91	0.94	0.97	0.99	1
-	P05997	HUMAN Collagen alpha-2(V) chain	0.608	0.182	0.109	-0.31	0.99	1.00	1.00	0.99	1
P02462	F1RLM1	PIG Uncharacterized protein (Fragment) COL4A1	-0.49	-0.37	-0.18	0.047	0.99	1.01	1.00	1.00	1
P02452	I3LJX2	PIG Uncharacterized protein	-0.76	-1.23	-0.48	0.464	0.97	0.86	0.99	0.98	2
Q05707	F1S285	PIG Uncharacterized protein COL14A1	-1.33	-1.08	-0.81	0.683	0.90	0.91	0.96	0.96	3
-	A6H560	PIG Mmp12 protein	-0.23	-0.16	-0.48	-0.9	1.00	1.00	0.99	0.92	1
-	Q5TAT6	HUMAN Collagen alpha-1(XIII) chain	-0.69	-0.22	0.023	-0.34	0.98	1.00	1.00	0.99	1

-	Q1T7A8	PIG Type VI collagen alpha-1 chain (Fragment)	-0.9	-1.62	-0.95	-0.13	0.96	0.66	0.93	1.00	2
-	Q9XSD9	PIG Decorin	-1.7	-0.55	-0.51	0.501	0.75	0.99	0.99	0.98	13
-	Q14055	HUMAN Collagen alpha-2(IX) chain	-3.61	-3.09	-0.76	1.596	0.03	0.07	0.97	0.80	1
Q8IZC6	I3LDG8	PIG Uncharacterized protein	-2.13	-1.12	-0.55	0.659	0.50	0.90	0.98	0.96	2
-	Q01955	HUMAN Collagen alpha-3(IV) chain	-0.5	0.501	-0.51	-0.37	0.99	1.00	0.99	0.99	1
-	P20908	HUMAN Collagen alpha-1(V) chain	-1.42	-0.7	-0.56	0.256	0.86	0.99	0.98	0.99	1
-	P79311	PIG Collagen alpha 2(I) chain (Fragment)	-1.55	-0.72	-1.68	0.123	0.81	0.99	0.68	1.00	2
-	Q76M96	HUMAN Coiled-coil domain-containing protein 80	-0.9	-1.18	-0.63	-0.29	0.96	0.88	0.98	0.99	2
-	F1RT93	PIG Uncharacterized protein CHAD	-0.66	-0.47	-1.18	-1.34	0.98	1.00	0.88	0.83	1
-	Q05707	HUMAN Collagen alpha-1(XIV) chain	0.118	0.341	-1.16	-1.18	0.99	1.01	0.88	0.87	1
-	P12111	HUMAN Collagen alpha-3(VI) chain	-0.85	-1.36	-1.2	-0.19	0.96	0.80	0.87	0.99	1
Q03001	F1RZU8	PIG Uncharacterized protein (Fragment) DST	-0.8	0.327	-1.77	-1.09	0.96	1.00	0.65	0.89	4
P20849	F1RTT3	PIG Uncharacterized protein COL9A1	-0.03	-0.45	-1.25	-1.35	1.00	1.00	0.85	0.83	2
-	I3LD20	PIG Dystroglycan	-0.97	-0.44	-0.88	-0.76	0.96	1.00	0.94	0.96	8
-	F1SUE9	PIG Uncharacterized protein (Fragment) COL13A1	-1.84	-1.99	-2.15	-1.98	0.68	0.46	0.45	0.67	1

Human equivalent Ac. No.	Accession No.	Collagen<br>Protein	Zq (log2 FC) 120	1	4	7	FDR 120	1	4	7	No. peptides
-	F1STZ3	PIG Uncharacterized protein C1QC	2.553	2.481	2.407	1.545	0.30	0.23	0.32	0.80	1
-	Q69DL0	PIG Complement C1q subcomponent subunit A	0.889	2.606	3.095	1.688	0.96	0.18	0.10	0.76	4
-	F1RQI2	PIG Uncharacterized protein COL12A1	0.621	1.007	1.711	3.067	0.98	0.93	0.67	0.24	1
-	Q95285	PIG Collagen alpha 1 (XV) chain (Fragment)	0.803	1.541	1.506	1.639	0.96	0.71	0.75	0.78	1
-	P08123	HUMAN Collagen alpha-2(I) chain	-1.13	1.075	0.496	1.511	0.93	0.92	0.99	0.81	3
-	Q15848	HUMAN Adiponectin	0.918	0.866	1.13	2.405	0.96	0.97	0.90	0.51	1
P02452	I3LUM2	PIG Uncharacterized protein	-1.39	0.987	0.66	2.153	0.87	0.94	0.98	0.58	2
-	F1SKM1	PIG Uncharacterized protein COL7A1	0.651	1.58	1.918	1.192	0.98	0.68	0.56	0.87	3

P08572	F1RLL9	PIG Uncharacterized protein (Fragment) COL4A2	0.287	0.465	1.047	1.407	1.00	1.00	0.91	0.82	4
-	P08572	HUMAN Collagen alpha-2(IV) chain	2.153	0.896	0.05	1.243	0.49	0.97	1.00	0.86	1
-	Q86Y22	HUMAN Collagen alpha-1(XXIII) chain	0.726	0.475	0.946	1.15	0.97	1.00	0.93	0.87	1
-	F1STZ1	PIG Uncharacterized protein C1QB	-0.17	0.685	1.641	0.911	0.99	0.99	0.69	0.92	3
-	A5D9K7	PIG Collagen type XI alpha 2	1.002	1.471	0.935	0.465	0.96	0.74	0.93	0.98	2
P02461	F1RYI8	PIG Uncharacterized protein COL3A1	-0	0.042	1.021	1.81	1.00	1.00	0.92	0.73	7
P20908	F1S021	PIG Uncharacterized protein (Fragment) COL5A1	-0.29	0.554	1.014	1.412	1.00	0.99	0.91	0.82	5
-	Q99715	HUMAN Collagen alpha-1(XII) chain	0.605	0.266	0.62	1.264	0.99	1.00	0.98	0.85	17
-	Q59IP1	PIG Procollagen alpha 3(V)	-0.11	0.704	1.016	1.68	1.00	0.99	0.91	0.77	3
-	F1SV48	PIG Uncharacterized protein COL8A2	0.82	0.434	0.522	0.767	0.96	1.01	0.99	0.95	1
-	P02452	HUMAN Collagen alpha-1(I) chain	-0.75	0.326	0.6	1.108	0.97	1.00	0.98	0.88	8
-	I3L8B2	PIG Uncharacterized protein (Fragment) COL9A2	-0.73	3.018	3.035	1.083	0.97	0.08	0.11	0.89	1
-	P02461	HUMAN Collagen alpha-1(III) chain	-1.32	0.97	0.332	1.023	0.90	0.95	0.99	0.91	2
-	P12107	HUMAN Collagen alpha-1(XI) chain	0.491	-0.12	0.759	1.185	0.99	1.00	0.97	0.87	2
-	F1SSE7	PIG Uncharacterized protein (Fragment) COL15A1	0.657	0.687	-0.22	0.311	0.98	0.99	1.00	0.99	3
-	Q1T7A9	PIG Type VI collagen alpha-1 chain (Fragment)	0.335	0.209	0.108	0.745	1.00	1.00	1.00	0.96	2
P08123	F1SFA7	PIG Uncharacterized protein COL1A2	-0.82	0.537	-0.03	1.099	0.96	0.99	1.00	0.89	11
Q99715	F1RQI0	PIG Uncharacterized protein COL12A1	-0.13	-0.03	0.516	1.35	0.99	1.00	0.99	0.83	6
-	Q1T7A5	PIG Type VI collagen alpha-3 chain (Fragment)	-0.81	-0.48	0.112	1.221	0.96	1.00	1.00	0.86	16
-	P20849	HUMAN Collagen alpha-1(IX) chain	0.445	0.23	0.25	0.101	0.99	1.00	1.00	1.00	1
-	P02458	HUMAN Collagen alpha-1(II) chain	0.283	0.629	-0.17	0.104	0.99	0.99	1.00	1.00	2
P02458	I3LSV6	PIG Uncharacterized protein (Fragment) COL2A1	-0.72	-0.21	-0.1	0.595	0.97	1.00	1.00	0.97	1
Q05707	K7GT00	PIG Uncharacterized protein (Fragment) COL14A1	0.074	0.045	-0.1	0.418	1.00	1.00	1.00	0.99	20
-	P25940	HUMAN Collagen alpha-3(V) chain	-0.21	0.189	-0.24	0.891	0.99	1.00	1.00	0.93	1
P39059	I3LIL7	PIG Uncharacterized protein LOC100620394	0.011	0.088	0.115	0.719	1.00	1.00	1.00	0.96	6
-	F1S422	PIG Uncharacterized protein (Fragment) COL23A1	-1.34	-1.04	0.046	1.754	0.89	0.93	1.00	0.74	1
-	P12109	HUMAN Collagen alpha-1(VI) chain	-1.9	-1.16	-0.27	1.41	0.63	0.89	1.00	0.82	6

-	Q02388	HUMAN Collagen alpha-1(VII) chain	0.482	0.13	0.039	-0.14	0.98	1.00	1.00	1.00	4
P12109	I3LS72	PIG Uncharacterized protein COL6A1	-1.53	-1.43	-0.36	1.474	0.81	0.76	1.00	0.81	10
-	Q59IP2	PIG Procollagen alpha 2(V)	-0.07	-0.44	0.053	0.596	1.00	1.00	1.00	0.97	3
P12110	I3LQ84	PIG Uncharacterized protein	-1.43	-1.24	-0.48	1.033	0.86	0.86	0.99	0.91	21
-	Q9UMD9	HUMAN Collagen alpha-1(XVII) chain	-1.29	-0.25	0.153	-1.01	0.91	1.00	1.00	0.91	1
-	P05997	HUMAN Collagen alpha-2(V) chain	0.608	0.182	0.109	-0.31	0.99	1.00	1.00	0.99	1
P02462	F1RLM1	PIG Uncharacterized protein (Fragment) COL4A1	-0.49	-0.37	-0.18	0.047	0.99	1.01	1.00	1.00	1
-	B9UJD6	PIG C1q and tumor necrosis factor related protein 3 isoform b	-0.91	-0.56	-0.15	-0.18	0.96	0.99	1.00	0.99	1
P02452	I3LJX2	PIG Uncharacterized protein	-0.76	-1.23	-0.48	0.464	0.97	0.86	0.99	0.98	2
-	I3LGX5	PIG Collagen, type IV, alpha 3 (Goodpasture antigen) binding protein tv2	-1.39	-1.17	-0.22	0.448	0.87	0.88	1.00	0.99	1
Q05707	F1S285	PIG Uncharacterized protein COL14A1	-1.33	-1.08	-0.81	0.683	0.90	0.91	0.96	0.96	3
-	Q5TAT6	HUMAN Collagen alpha-1(XIII) chain	-0.69	-0.22	0.023	-0.34	0.98	1.00	1.00	0.99	1
-	Q1T7A8	PIG Type VI collagen alpha-1 chain (Fragment)	-0.9	-1.62	-0.95	-0.13	0.96	0.66	0.93	1.00	2
-	Q14055	HUMAN Collagen alpha-2(IX) chain	-3.61	-3.09	-0.76	1.596	0.03	0.07	0.97	0.80	1
Q8IZC6	I3LDG8	PIG Uncharacterized protein	-2.13	-1.12	-0.55	0.659	0.50	0.90	0.98	0.96	2
-	Q01955	HUMAN Collagen alpha-3(IV) chain	-0.5	0.501	-0.51	-0.37	0.99	1.00	0.99	0.99	1
-	P20908	HUMAN Collagen alpha-1(V) chain	-1.42	-0.7	-0.56	0.256	0.86	0.99	0.98	0.99	1
-	P79311	PIG Collagen alpha 2(I) chain (Fragment)	-1.55	-0.72	-1.68	0.123	0.81	0.99	0.68	1.00	2
-	Q3HUX1	PIG Fatty acid translocase/CD36	-0.69	-0.18	-0.77	-0.95	0.98	1.00	0.97	0.92	8
-	Q05707	HUMAN Collagen alpha-1(XIV) chain	0.118	0.341	-1.16	-1.18	0.99	1.01	0.88	0.87	1
-	P12111	HUMAN Collagen alpha-3(VI) chain	-0.85	-1.36	-1.2	-0.19	0.96	0.80	0.87	0.99	1
P20849	F1RTT3	PIG Uncharacterized protein COL9A1	-0.03	-0.45	-1.25	-1.35	1.00	1.00	0.85	0.83	2
-	K9J4M9	PIG Collectin sub-family member 12	-1.22	-1.36	-0.52	-1.12	0.92	0.80	0.99	0.88	1
-	F1SP14	PIG Uncharacterized protein AMACR	-1.27	-1.48	-1.49	-1.01	0.91	0.73	0.75	0.92	2
-	F1SUE9	PIG Uncharacterized protein (Fragment) COL13A1	-1.84	-1.99	-2.15	-1.98	0.68	0.46	0.45	0.67	1
**Human**		**Renal toxicity biomarker panel**									

equivalent Ac. No.	Accession No.	Protein	Zq (log2 FC) 120	1	4	7	FDR 120	1	4	7	No. peptides
-	I3LQR9	PIG Fibrinogen alpha chain	1.343	2.49	3.618	3.539	0.89	0.23	0.03	0.11	11
-	F1RX36	PIG Fibrinogen alpha chain	0.735	2.968	4.25	3.62	0.97	0.09	0.00	0.09	4
-	F1SQZ6	PIG Uncharacterized protein SLCO1A2	1.718	1.769	0.349	0.643	0.73	0.58	0.99	0.96	1
-	P35624	PIG Metalloproteinase inhibitor 1	0.697	0.958	1.523	0.204	0.98	0.95	0.74	0.99	1
-	P14460	PIG Fibrinogen alpha chain (Fragment)	0.285	0.985	0.488	0.584	0.99	0.94	0.99	0.97	2
-	P14287	PIG Osteopontin	0.038	0.042	0.493	1.487	1.00	1.00	0.99	0.81	6
-	O02705	PIG Heat shock protein HSP 90-alpha	-0.49	-0.34	0.936	1.245	0.99	1.01	0.93	0.86	44
-	Q06AA3	PIG Regucalcin	2.663	0.882	0.006	-0.31	0.24	0.97	1.00	0.99	1
-	F1SKD6	PIG Uncharacterized protein SLC16A7	-1.88	-1.62	-0.93	0.443	0.64	0.66	0.93	0.99	1
-	P04792	HUMAN Heat shock protein beta-1	0.135	-0	0.099	0.358	0.99	1.00	1.00	0.99	1
-	P09211	HUMAN Glutathione S-transferase P	0.826	0.113	-0.36	-0.54	0.96	1.00	0.99	0.97	1
-	P80031	PIG Glutathione S-transferase P	0.268	0.17	-0.19	-0.08	0.99	1.00	1.00	1.00	6
-	Q5S1U1	PIG Heat shock protein beta-1	-0.97	-0.29	0.266	0.559	0.96	1.00	1.00	0.98	11

Vesicle-mediated transport

Human equivalent Ac. No.	Accession No.	Protein	Zq (log2 FC) 120	1	4	7	FDR 120	1	4	7	No. peptides
-	P18648	PIG Apolipoprotein A-I	2.421	2.571	1.779	1.218	0.36	0.19	0.64	0.86	34
-	P08835	PIG Serum albumin	3.428	2.943	2.28	1.742	0.04	0.09	0.39	0.75	27
-	I3L818	PIG Uncharacterized protein (Fragment) SERPINF2	1.263	1.742	2.977	2.046	0.91	0.59	0.12	0.65	4
-	P42768	HUMAN Wiskott-Aldrich syndrome protein	2.094	1.578	1.393	1.428	0.52	0.69	0.80	0.82	1
-	Q9GKQ6	PIG Biglycan (Fragments)	1.016	0.999	1.649	3.165	0.96	0.94	0.69	0.22	8
-	P02768	HUMAN Serum albumin	1.769	2.264	1.765	0.632	0.71	0.32	0.65	0.96	3
-	P09571	PIG Serotransferrin	3.225	2.269	1.463	1.179	0.07	0.32	0.76	0.87	33
P01023	I3LQ17	PIG Uncharacterized protein	2.016	1.788	2.079	1.597	0.57	0.57	0.48	0.80	50

P52565	F1SQW8	PIG Uncharacterized protein ARHGDIB	0.663	1.135	1.733	1.67	0.98	0.89	0.67	0.77	10
P40121	F1SVB0	PIG Uncharacterized protein CAPG	0.855	1.012	2.202	2.241	0.96	0.94	0.43	0.55	9
-	P50447	PIG Alpha-1-antitrypsin	2.605	2.139	1.561	1.19	0.27	0.38	0.73	0.87	12
-	Q9H0H5	HUMAN Rac GTPase-activating protein 1	1.535	2.025	1.57	1.573	0.81	0.44	0.72	0.80	1
-	Q0PM28	PIG Pigment epithelium-derived factor	1.395	1.762	1.394	1.491	0.87	0.58	0.80	0.81	13
Q5T5C0	I3LTV2	PIG Uncharacterized protein	0.542	1.49	1.002	1.257	0.98	0.73	0.92	0.85	1
-	Q29268	PIG Syntaxin-binding protein 2 (Fragment)	1.659	1.992	1.646	0.824	0.76	0.46	0.69	0.94	1
-	P61421	HUMAN V-type proton ATPase subunit d 1	1.043	1.46	1.179	1.319	0.95	0.74	0.88	0.84	3
-	H0Y9H1	HUMAN Calnexin (Fragment)	1.048	2.633	3.115	0.64	0.95	0.17	0.09	0.96	1
O43752	F1S678	PIG Uncharacterized protein STX6	0.804	1.686	2.414	1.817	0.96	0.63	0.31	0.73	1
P04114	I3LKZ1	PIG Uncharacterized protein	0.847	1.018	1.486	1.379	0.96	0.93	0.75	0.83	16
P04114	F1SCV8	PIG Uncharacterized protein (Fragment)	1.058	1.399	1.319	1.01	0.95	0.78	0.83	0.92	25
Q7KZF4	F1SML4	PIG Uncharacterized protein SND1	1.231	0.699	1.266	1.425	0.91	0.99	0.85	0.82	10
-	H0YA55	HUMAN Serum albumin (Fragment)	2.347	2.004	1.349	1.456	0.39	0.45	0.82	0.82	1
P31946	F1SDR7	PIG Uncharacterized protein YWHAB	0.492	0.396	0.958	1.315	0.99	1.00	0.93	0.84	8
-	O77735	PIG Secretory carrier-associated membrane protein 1	0.736	0.815	1.384	0.678	0.97	0.98	0.80	0.96	1
-	I3LKI1	PIG Uncharacterized protein SRGN	1.718	1.809	1.266	0.4	0.73	0.56	0.85	0.99	2
-	K9J6H8	PIG Alpha-2-macroglobulin	1.728	1.975	1.262	-0.11	0.73	0.47	0.85	1.00	3
-	F1SS42	PIG Uncharacterized protein AP1G2	0.919	0.759	1.472	1.661	0.96	0.99	0.76	0.77	1
-	F1SG75	PIG Uncharacterized protein SSPN	1.227	1.728	1.329	2.361	0.91	0.61	0.83	0.52	1
P15313	F1RMZ8	PIG Uncharacterized protein ATP6V1B2	0.741	1.146	1.276	0.687	0.97	0.89	0.85	0.96	6
P35443	F1RF28	PIG Uncharacterized protein	0.181	0.143	0.961	1.717	0.99	1.00	0.93	0.75	9
P51149	F1SPG0	PIG Uncharacterized protein (Fragment) RAB7A	0.737	0.657	1.126	0.947	0.97	0.99	0.90	0.92	7
-	G3V387	HUMAN Short peptide from AAT (Fragment)	1.437	1.245	0.788	0.556	0.85	0.86	0.96	0.98	1
Q9BTT0	F1SDG7	PIG Uncharacterized protein ANP32E	1.081	1.466	0.959	0.246	0.94	0.74	0.93	0.99	1
-	A1E295	PIG Cathepsin B	0.227	0.213	1.518	2.323	1.00	1.00	0.75	0.52	6
-	P19620	PIG Annexin A2	-0.12	0.419	1.155	1.75	0.99	1.00	0.89	0.74	32

O95782	I3LK24	PIG Uncharacterized protein AP2A1	1.336	1.124	1.066	1.158	0.89	0.90	0.91	0.87	8
O94855	F1S155	PIG Uncharacterized protein SEC24D	1.647	0.784	0.626	0.606	0.76	0.98	0.98	0.97	3
P49755	F1S2Q3	PIG Uncharacterized protein TMED10	0.791	0.745	0.848	0.841	0.96	0.99	0.95	0.94	6
P63010	I3L6Y6	PIG Uncharacterized protein (Fragment) AP2B1	0.682	0.119	0.767	1.037	0.98	1.00	0.97	0.91	13
-	Q29021	PIG Apolipoprotein B (Fragment)	1.063	0.566	0.926	0.508	0.95	0.99	0.93	0.98	12
-	Q28833	PIG von Willebrand factor (Fragment)	0.779	1.131	0.987	0.846	0.97	0.90	0.92	0.94	7
-	P15145	PIG Aminopeptidase N	5.262	2.949	-0.21	-0.64	0.00	0.09	1.00	0.96	1
P12814	I3LS04	PIG Uncharacterized protein (Fragment)	-0.39	-0.3	0.908	2.173	0.99	1.00	0.93	0.60	18
-	Q15907	HUMAN Ras-related protein Rab-11B	0.973	0.714	0.354	0.264	0.96	0.99	0.99	0.99	1
O43242	F1RXA7	PIG Uncharacterized protein LOC100512253	0.293	-0.02	0.434	0.77	1.00	1.00	0.99	0.95	13
-	P35624	PIG Metalloproteinase inhibitor 1	0.697	0.958	1.523	0.204	0.98	0.95	0.74	0.99	1
-	Q9GMB0	PIG Dolichyl-diphosphooligosaccharide--protein glycosyltransferase subunit 1	-0.15	0.209	0.794	1.095	0.99	1.00	0.96	0.89	19
-	P20112	PIG SPARC	-0.07	0.244	1.339	1.478	1.00	1.00	0.82	0.81	5
P12814	I3LIK6	PIG Uncharacterized protein	-0.26	-0.39	0.658	1.7	0.99	1.01	0.98	0.75	9
-	Q06AU3	PIG Ras-related protein Rab-3A	0.219	0.181	0.706	1.309	0.99	1.00	0.97	0.84	1
-	Q7L1I2	HUMAN Synaptic vesicle glycoprotein 2B	1.052	0.63	0.688	0.845	0.95	0.99	0.97	0.94	1
-	F1SVB3	PIG Uncharacterized protein (Fragment) TGOLN2	0.717	0.321	0.59	0.614	0.97	1.00	0.98	0.97	1
-	Q8MJ37	PIG Ran (Fragment)	-0.73	-0.48	0.884	1.357	0.97	1.00	0.94	0.83	3
P48444	I3LBY0	PIG Uncharacterized protein ARCN1	-0.09	0.069	0.605	1.333	1.00	1.00	0.98	0.83	9
-	P14287	PIG Osteopontin	0.038	0.042	0.493	1.487	1.00	1.00	0.99	0.81	6
-	F1RUN2	PIG Serum albumin	1.327	0.551	0.491	0.512	0.90	0.99	0.99	0.98	2
-	P51149	HUMAN Ras-related protein Rab-7a	-0.06	0.407	0.918	1.113	1.00	1.01	0.93	0.88	3
O94979	F1RVD4	PIG Uncharacterized protein SEC31A	0.072	0.034	0.689	1.377	1.00	1.00	0.97	0.83	14
-	I3LB80	PIG Uncharacterized protein (Fragment) SLC3A2	0.615	1.022	0.619	0.623	0.98	0.93	0.98	0.96	3
-	Q06AU6	PIG Ras-related protein Rab-5A	1.162	0.505	0.57	0.648	0.93	1.00	0.98	0.96	5
Q15437	F1SBH2	PIG Uncharacterized protein SEC23B	0.493	1.077	1.156	0.622	0.99	0.91	0.89	0.96	2
-	K7EM20	HUMAN 14-3-3 protein epsilon (Fragment)	0.556	0.167	0.379	-0.04	0.98	1.00	0.99	1.00	1

-	P31146	HUMAN Coronin-1A	-0.49	0.502	0.76	0.4	0.99	1.00	0.97	0.99	1
Q8WVM8	F1SHF4	PIG Uncharacterized protein SCFD1	1.016	0.525	0.344	0.443	0.96	1.00	0.99	0.99	6
-	P54709	HUMAN Sodium/potassium-transporting ATPase subunit beta-3	0.977	1.346	0.23	0.245	0.96	0.81	1.00	0.99	2
P53992	F1SU53	PIG Uncharacterized protein SEC24C	0.192	0.117	0.305	0.607	0.99	1.00	1.00	0.97	2
-	B5LX40	PIG Adaptor protein phosphotyrosine interaction PH domain and leucine zipper	1.024	0.083	0.162	0.266	0.95	1.00	1.00	0.99	3
-	F1S0V3	PIG Annexin (Fragment)	0.339	0.395	0.242	0.585	1.00	1.00	1.00	0.97	31
-	F1SI24	PIG Synaptosomal-associated protein	0.106	-0.22	0.418	0.972	1.00	1.00	1.00	0.92	3
-	J3KP28	HUMAN Synaptotagmin-like protein 2	0.751	0.582	0.252	0.748	0.97	0.99	1.00	0.96	1
-	Q52NJ0	PIG Prenylated Rab acceptor protein 1	1.173	0.283	0.379	0.476	0.93	1.00	0.99	0.98	1
Q15436	F1SHL3	PIG Uncharacterized protein SEC23A	-0.51	-0.09	0.687	1.236	0.99	1.00	0.97	0.86	3
P27105	F1SMD7	PIG Uncharacterized protein STOM	0.206	0.689	0.466	0.347	0.99	0.99	0.99	0.99	5
O95486	F1S129	PIG Uncharacterized protein (Fragment) SEC24B	0.926	0.662	0.18	-0.15	0.96	0.99	1.00	1.00	2
-	Q52NJ1	PIG Ras-related protein Rab-11A	-0.13	0.323	0.347	0.312	0.99	1.00	0.99	0.99	9
Q7KZF4	I3LDE1	PIG Uncharacterized protein LOC100521234	0.514	-0.25	0.333	0.902	0.99	1.00	1.00	0.92	3
O43707	F1RI39	PIG Uncharacterized protein LOC100517284	0.113	-0.14	0.346	0.844	1.00	1.00	0.99	0.94	37
O43852	F1SMN1	PIG Uncharacterized protein (Fragment) CALU	0.836	1.341	0.57	0.984	0.96	0.81	0.98	0.92	11
P57088	F1S4G6	PIG Uncharacterized protein TMEM33	0.016	0.096	0.27	0.216	1.00	1.00	1.00	0.99	1
Q9P2U7	F1RHZ7	PIG Uncharacterized protein SLC17A7	0.817	0.155	0.169	0.222	0.96	1.00	1.00	0.99	1
P61020	I3LFZ8	PIG Uncharacterized protein (Fragment) RAB5B	0.588	0.421	-0.34	-0.68	0.98	1.01	0.99	0.96	3
-	Q9MZ46	PIG Crocalbin-like protein (Fragment)	-0.78	0.469	0.446	1.227	0.97	1.00	0.99	0.86	9
-	F1RP38	PIG Uncharacterized protein BCAN	0.108	0.664	0.161	0.07	1.00	0.99	1.00	1.00	1
Q9NRW1	F2Z5S1	PIG Uncharacterized protein (Fragment) RAB6B	-0.03	0.126	-0	0.22	1.00	1.00	1.00	0.99	4
H7C4W3	K7GNZ1	PIG Uncharacterized protein (Fragment) SYP	0.975	0.942	-0.05	-1.02	0.96	0.96	1.00	0.91	1
-	F1S0V2	PIG Annexin	-0.31	-0.33	-0.16	0.543	1.00	1.01	1.00	0.97	5
-	P08133	HUMAN Annexin A6	0.779	0.511	0.159	-0.57	0.97	1.00	1.00	0.97	1
-	D6RB85	HUMAN Calnexin (Fragment)	-0.04	-0	0.714	1.048	1.00	1.00	0.97	0.91	1
Q9UL25	F1SH25	PIG Uncharacterized protein RAB21	0.282	-0.46	-0.21	0.005	0.99	1.00	1.00	1.00	2

-	I3LBF3	PIG Uncharacterized protein RILP	0.31	0.119	-0.21	0.192	1.00	1.00	1.00	0.99	1
-	I3LBS8	PIG Uncharacterized protein SYNRG	-0.05	-0.41	-0.29	0.225	1.00	1.01	1.00	0.99	1
P63000	I3LFI0	PIG Uncharacterized protein (Fragment) RAC1	0.262	-0.24	0.024	0.243	0.99	1.00	1.00	0.99	3
-	Q96CW1	HUMAN AP-2 complex subunit mu	-0.22	-0.35	0.111	0.525	0.99	1.01	1.00	0.98	4
-	P04114	HUMAN Apolipoprotein B-100	0.647	-0.06	-0.2	0.209	0.98	1.00	1.00	0.99	6
-	P62258	HUMAN 14-3-3 protein epsilon	0.205	-0.79	-0.49	-0.06	0.99	0.99	0.99	1.00	5
-	Q710C4	PIG Adenosylhomocysteinase	-0.55	-1.01	0.101	0.583	0.99	0.94	1.00	0.97	10
-	Q13554	HUMAN Calcium/calmodulin-dependent protein kinase type II subunit beta	0.052	-0.54	-0.25	-0.67	1.00	0.99	1.00	0.96	2
-	F8WCP1	HUMAN ALS2 C-terminal-like protein	0.274	-0.15	0.065	-0.14	0.99	1.00	1.00	1.00	1
Q12846	F1RIR2	PIG Uncharacterized protein STX4	-0.8	-0.24	-0.22	-0.07	0.96	1.00	1.00	1.00	2
-	A0SNV3	PIG Tyrosine 3-monooxygenase/tryptophan 5-monooxygenase activation protein	-0.86	-0.88	0.181	0.714	0.96	0.97	1.00	0.96	7
-	F1S2E2	PIG Annexin	-0.14	0.054	-0.1	-0.2	0.99	1.00	1.00	0.99	14
-	Q8HZV3	PIG Transferrin receptor protein 1	0.137	-0.12	-0.31	-0.37	0.99	1.00	1.00	0.99	15
-	P56377	HUMAN AP-1 complex subunit sigma-2	1.459	1.474	-0.53	-1.05	0.84	0.73	0.98	0.91	1
-	Q9BTT0	HUMAN Acidic leucine-rich nuclear phosphoprotein 32 family member E	-1.95	-1.17	0.096	0.862	0.61	0.88	1.00	0.94	2
-	Q52NJ6	PIG Ras-related protein Rab-14	0.116	0.082	-0.13	-0.07	0.99	1.00	1.00	1.00	5
-	M3VH45	PIG Annexin	-0.03	0.459	-0.36	-0.07	1.00	1.00	1.00	1.00	4
-	P05024	PIG Sodium/potassium-transporting ATPase subunit alpha-1	-0.65	-0.35	-0.48	-0.52	0.98	1.01	0.99	0.98	39
-	P12814	HUMAN Alpha-actinin-1	-1	-1.06	-0.14	2.457	0.96	0.92	1.00	0.48	4
-	P02818	HUMAN Osteocalcin	1.316	2.546	0.353	-0.78	0.90	0.20	0.99	0.95	1
-	P07101	HUMAN Tyrosine 3-monooxygenase	-0.44	1.034	-0.37	-0.29	0.99	0.93	0.99	0.99	1
-	P62826	HUMAN GTP-binding nuclear protein Ran	-1.42	-0.65	0.072	0.237	0.86	0.99	1.00	0.99	3
-	I3LR17	PIG Uncharacterized protein CORO1A	-0.46	-1.08	-0.95	0.639	0.99	0.91	0.93	0.96	1
-	Q29245	PIG Clathrin coat assembly protein AP50 (Fragment)	-0.59	-0.69	-0.61	-0.04	0.98	0.99	0.98	1.00	1
-	H3BPJ5	HUMAN Synaptogyrin-3 (Fragment)	-0.55	-0.84	-0.34	0.16	0.98	0.98	0.99	1.00	1
-	I3L812	PIG Uncharacterized protein TPP1	-0.17	-0.43	-0.15	0.228	0.99	1.01	1.00	0.99	1
-	K9IVU3	PIG AP-1 complex subunit gamma-1	-0.59	-0.46	-0.58	-0.01	0.99	1.00	0.98	1.00	1

-	F1RZJ0	PIG Uncharacterized protein (Fragment) RAB11FIP1	-0.99	-0.64	0.354	0.035	0.96	0.99	0.99	1.00	1
P62258	K7GP62	PIG Uncharacterized protein	-0.39	-1.02	-0.58	-0.18	0.99	0.93	0.98	0.99	5
Q9BT88	I3LSH0	PIG Uncharacterized protein	-1.17	-0.96	0.195	0.352	0.93	0.95	1.00	0.99	1
O94973	F1RZ02	PIG Uncharacterized protein (Fragment) AP2A2	0.005	-0.57	-0.52	-0.27	1.00	0.99	0.99	0.99	3
Q86Y82	I3LHT9	PIG Uncharacterized protein STX12	-0.2	-0.04	-0.37	-0.44	0.99	1.00	0.99	0.99	3
Q96KG9	F1RRK0	PIG Uncharacterized protein SCYL1	-0.74	-0.81	-0.49	0.006	0.97	0.98	0.99	1.00	2
-	P20303	PIG Solute carrier family 2, facilitated glucose transporter member 1 (Fragment)	0.019	-0.1	-0.9	-0.87	1.00	1.00	0.93	0.93	1
Q9HCH5	F1STT2	PIG Uncharacterized protein SYTL2	-1.2	-0.72	-0.22	-0.32	0.92	0.99	1.00	0.99	1
-	F1STQ1	PIG Uncharacterized protein (Fragment) SYTL1	1.255	-0.51	-0.68	-0.39	0.91	1.00	0.97	0.99	2
-	I3L9F1	PIG Uncharacterized protein (Fragment) BSG	-0.57	0.312	-0.62	-1.26	0.98	1.00	0.98	0.85	6
-	Q2QLE2	PIG Caveolin-2	-1.4	-1.04	-0.93	-0.49	0.87	0.93	0.93	0.98	2
P21579	F1RYK1	PIG Uncharacterized protein SYT1	-0.86	-1.35	-1.21	-0.78	0.96	0.81	0.86	0.95	2
-	Q7JFN4	PIG Calcium/calmodulin-dependent protein kinase II isoform gamma-B	-0.72	-0.84	-0.92	-0.55	0.97	0.98	0.93	0.97	3
-	F1RVY9	PIG Uncharacterized protein UNC13D	1.892	-1.65	-1.3	-1.12	0.63	0.65	0.83	0.88	1
Q7L0J3	F1SDF9	PIG Uncharacterized protein SV2A	-1.21	-0.63	-1.49	-1.25	0.92	0.99	0.75	0.85	1
-	P10323	HUMAN Acrosin	-0.87	-0.24	-0.98	-0.41	0.96	1.00	0.92	0.99	1
-	O75843	HUMAN AP-1 complex subunit gamma-like 2	-0.94	-0.91	-1.05	-1.21	0.96	0.96	0.91	0.87	1
-	P15203	PIG Transforming growth factor beta-3	-1.66	-0.54	-1.4	-2.16	0.76	0.99	0.80	0.59	1
Q13554	F1SSH3	PIG Uncharacterized protein	-0.19	-1.04	-1.03	-1.08	0.99	0.93	0.92	0.89	1
O00186	F1S5Y5	PIG Uncharacterized protein STXBP3	-1.65	-1.84	-1.55	-0.82	0.76	0.54	0.73	0.94	4
-	Q9N1X4	PIG Pulmonary surfactant-associated protein D	-1.81	-1.04	-1.32	-1.06	0.69	0.93	0.83	0.90	1
P12814	I3LCP0	PIG Uncharacterized protein (Fragment) LOC100519531	-1.57	-2.46	-1.73	-1.53	0.80	0.23	0.66	0.80	11
-	Q6UV40	PIG Fructose-bisphosphate aldolase A (Fragment)	-1.82	-2.69	-1.62	-0.99	0.68	0.15	0.70	0.92	5
-	P04075	HUMAN Fructose-bisphosphate aldolase A	-1.91	-2.87	-1.65	-1.09	0.62	0.11	0.69	0.89	16
-	Q95266	PIG Calcium/calmodulin-dependent protein kinase type II subunit delta	-2.12	-2.99	-1.67	-1.05	0.51	0.08	0.69	0.91	16
-	Q9Y296	HUMAN Trafficking protein particle complex subunit 4	-1.51	-2	-2.25	-1.88	0.83	0.45	0.40	0.70	1
-	Q96AX2	HUMAN Ras-related protein Rab-37	-1.89	-3.3	-2.5	-1.7	0.63	0.04	0.28	0.75	1

P12814	F1RHL9	PIG Uncharacterized protein ACTN2	-2.02	-3.39	-2.64	-2.17	0.57	0.03	0.23	0.59	55

Human equivalent Ac. No.	Accession No.	Angiogenesis<br>Protein	Zq (log2 FC)<br>120	1	4	7	FDR<br>120	1	4	7	No. peptides
-	P35054	PIG Transforming growth factor beta receptor type 3	4.203	5.916	2.7	0.092	0.00	0.00	0.21	1.00	1
-	P00747	HUMAN Plasminogen	1.196	4.368	3.759	1.98	0.93	0.00	0.02	0.67	1
P04196	F1SFI5	PIG Uncharacterized protein HRG	2.263	3.28	1.95	1.42	0.44	0.04	0.55	0.82	12
-	P06867	PIG Plasminogen	1.073	2.63	3.175	1.432	0.94	0.17	0.08	0.82	13
-	F1SB81	PIG Plasminogen	0.204	2.644	2.932	2.36	0.99	0.17	0.14	0.52	2
-	P08670	HUMAN Vimentin	1.094	1.74	2.384	3.078	0.94	0.60	0.33	0.24	1
P98095	F1SPG5	PIG Uncharacterized protein LOC100738595	1.026	0.998	1.976	3.221	0.95	0.94	0.53	0.20	4
P05546	F1RKY2	PIG Uncharacterized protein SERPIND1	2.37	2.011	1.585	1.229	0.38	0.44	0.72	0.86	9
P02749	I3LGN5	PIG Uncharacterized protein (Fragment)	1.686	2.42	2.192	1.502	0.75	0.25	0.44	0.81	1
-	Q9H8V3	HUMAN Protein ECT2	2.675	2.623	1.772	1.08	0.24	0.17	0.64	0.89	1
-	P02543	PIG Vimentin	0.055	0.626	1.889	3.031	1.00	0.99	0.57	0.25	50
P02751	F1SS24	PIG Uncharacterized protein FN1	0.527	0.684	1.731	2.499	0.99	0.99	0.67	0.46	63
-	P29590	HUMAN Protein PML	1.274	2.803	2.666	0.78	0.91	0.12	0.22	0.95	1
-	Q0PM28	PIG Pigment epithelium-derived factor	1.395	1.762	1.394	1.491	0.87	0.58	0.80	0.81	13
-	Q9BY76	HUMAN Angiopoietin-related protein 4	1.476	1.112	1.427	1.342	0.84	0.90	0.78	0.83	1
Q13214	F1SPP4	PIG Uncharacterized protein SEMA3B	-0.02	2.759	3.172	0.88	1.00	0.14	0.08	0.93	1
-	Q03167	HUMAN Transforming growth factor beta receptor type 3	1.054	2.843	2.575	0.73	0.95	0.11	0.25	0.96	1
-	Q02297	HUMAN Pro-neuregulin-1, membrane-bound isoform	0.258	2.377	1.5	0.9	0.99	0.27	0.75	0.92	1
-	Q15848	HUMAN Adiponectin	0.918	0.866	1.13	2.405	0.96	0.97	0.90	0.51	1
-	Q19AZ5	PIG Antithrombin protein	1.401	1.523	1.301	1.017	0.87	0.72	0.84	0.91	17
-	Q8WMQ3	PIG CD9 antigen	0.139	1.238	2.075	1.81	0.99	0.86	0.48	0.73	1
-	Q8WWQ8	HUMAN Stabilin-2	0.536	0.792	1.622	1.83	0.98	0.98	0.70	0.73	1

-	Q06AT6	PIG RHOG	0.201	0.185	1.47	1.842	0.99	1.00	0.76	0.72	5
P84095	I3L725	PIG Uncharacterized protein RHOG	1.999	1.423	1.208	0.98	0.58	0.77	0.86	0.92	2
P35968	K7GNY2	PIG Uncharacterized protein (Fragment) KDR	1.298	0.004	0.28	0.158	0.91	1.00	1.00	1.00	1
-	P24821	HUMAN Tenascin	0.849	1.05	0.949	0.978	0.96	0.93	0.93	0.92	2
-	Q764M5	PIG Signal transducer and activator of transcription 1	1.111	0.314	0.849	0.971	0.94	1.00	0.95	0.92	14
-	E9PNH7	HUMAN Alpha-crystallin B chain (Fragment)	0.091	0.38	1.155	0.679	1.00	1.01	0.89	0.96	1
-	K9IVP5	PIG N-myosin-9	0.302	0.419	1.155	2.007	1.00	1.00	0.89	0.66	14
-	P21333	HUMAN Filamin-A	-0.08	0.1	1.221	2.285	1.00	1.00	0.86	0.54	60
P08572	F1RLL9	PIG Uncharacterized protein (Fragment) COL4A2	0.287	0.465	1.047	1.407	1.00	1.00	0.91	0.82	4
-	P08572	HUMAN Collagen alpha-2(IV) chain	2.153	0.896	0.05	1.243	0.49	0.97	1.00	0.86	1
-	Q9BX79	HUMAN Stimulated by retinoic acid gene 6 protein homolog	0.277	0.459	1.807	2.327	0.99	1.00	0.63	0.53	1
-	F1SIW0	PIG Uncharacterized protein STAB1	1.787	1.642	1.45	0.314	0.70	0.65	0.77	0.99	2
-	A1E295	PIG Cathepsin B	0.227	0.213	1.518	2.323	1.00	1.00	0.75	0.52	6
-	G9FSR3	PIG Non-muscle myosin heavy chain II A (Fragment)	0.202	0.322	0.959	1.505	0.99	1.00	0.93	0.81	27
-	F2Z5E2	PIG Uncharacterized protein SERPINC1	0.492	1.09	0.925	0.744	0.99	0.91	0.93	0.96	3
-	K7GLU6	PIG Uncharacterized protein ERAP1	1.378	1.339	0.939	0.43	0.88	0.81	0.93	0.99	2
-	H9LBP0	PIG Serpin peptidase inhibitor clade D member 1 (Fragment)	0.678	1.787	1.933	0.65	0.98	0.57	0.55	0.96	2
Q86UX7	F1RQ01	PIG Uncharacterized protein FERMT3	0.785	1.243	1.309	0.999	0.96	0.86	0.83	0.92	8
-	F1S4D7	PIG Uncharacterized protein GBP1	0.767	0.923	0.748	0.547	0.97	0.96	0.97	0.97	4
P54577	I3L5T8	PIG Uncharacterized protein YARS	1.163	0.515	0.827	0.79	0.92	1.00	0.95	0.95	6
-	A3EX84	PIG Galectin	-0.59	0.06	1.552	2.42	0.99	1.00	0.73	0.50	4
-	P32394	PIG Heme oxygenase 1	0.774	0.663	1.096	1.001	0.97	0.99	0.90	0.92	7
-	O14746	HUMAN Telomerase reverse transcriptase	1.55	1.253	1.069	0.831	0.81	0.85	0.91	0.94	3
-	Q9UP79	HUMAN A disintegrin and metalloproteinase with thrombospondin motifs 8	0.998	3.322	0.015	-0.82	0.96	0.04	1.00	0.94	1
-	P15145	PIG Aminopeptidase N	5.262	2.949	-0.21	-0.64	0.00	0.09	1.00	0.96	1
-	P02751	HUMAN Fibronectin	0.889	0.355	0.789	0.961	0.96	1.01	0.96	0.92	5
-	P79335	PIG Plasminogen activator inhibitor 1	1.745	1.24	0.6	0.308	0.72	0.86	0.98	0.99	2

-	P54577	HUMAN Tyrosine--tRNA ligase, cytoplasmic	0.757	1.253	1.555	0.269	0.97	0.85	0.73	0.99	1
-	Q29116	PIG Tenascin	0.059	-0.29	0.598	1.922	1.00	1.00	0.98	0.68	30
-	Q4GWZ2	PIG 40S ribosomal protein SA	0.322	0.154	1.18	1.259	1.00	1.00	0.88	0.85	8
-	P37176	PIG Endoglin	1.135	1.103	0.629	0.132	0.93	0.90	0.98	1.00	3
-	F1SPK8	PIG Uncharacterized protein CD63	0.774	0.457	1.041	1.036	0.97	1.00	0.91	0.91	3
-	P20112	PIG SPARC	-0.07	0.244	1.339	1.478	1.00	1.00	0.82	0.81	5
Q16270	F1RVH7	PIG Uncharacterized protein IGFBP7	0.645	0.393	0.616	1.113	0.98	1.00	0.98	0.88	1
-	Q5SZJ2	HUMAN Endorepellin (Fragment)	1.005	0.285	0.387	1.002	0.96	1.00	0.99	0.92	1
-	K7ENN9	HUMAN Arachidonate 12-lipoxygenase, 12S-type (Fragment)	1.849	1.505	-0.17	0.266	0.66	0.72	1.00	0.99	1
P49591	F1S5Z3	PIG Uncharacterized protein SARS	-0.38	0.37	0.752	1.188	0.99	1.01	0.97	0.87	7
-	Q95295	PIG Tryptophanyl-tRNA synthase (Fragment)	0.423	-0.07	0.068	1.278	0.99	1.00	1.00	0.85	1
-	O75628	HUMAN GTP-binding protein REM 1	0.972	0.175	0.617	0.389	0.96	1.00	0.98	0.99	1
-	P18054	HUMAN Arachidonate 12-lipoxygenase, 12S-type	0.231	1.471	0.483	0.48	0.99	0.74	0.99	0.98	1
-	Q000H8	PIG Glutathione S-transferase mu 2 (Fragment)	1.946	1.781	0.072	0.231	0.61	0.57	1.00	0.99	2
-	P79275	PIG Rho A (Fragment)	0.67	0.219	0.385	0.604	0.98	1.00	0.99	0.97	1
-	Q9UII2	HUMAN ATPase inhibitor, mitochondrial	6.597	3.521	-1.98	-2.31	0.00	0.02	0.53	0.53	1
-	O75369	HUMAN Filamin-B	0.497	0.223	0.379	0.756	0.99	1.00	0.99	0.96	20
-	I3LC64	PIG Uncharacterized protein ECM1	0.462	0.779	0.586	0.406	0.99	0.98	0.98	0.99	5
-	P61224	HUMAN Ras-related protein Rap-1b	0.293	0.069	0.337	0.642	1.00	1.00	0.99	0.96	2
-	K9IVV5	PIG Tryptophan--tRNA ligase, cytoplasmic isoform a	0.216	0.238	0.374	0.469	0.99	1.00	0.99	0.98	5
-	K9IVM8	PIG Ubiquitin carboxyl-terminal hydrolase CYLD isoform 2	-0.24	1.365	1.671	0.288	0.99	0.80	0.68	0.99	1
-	F1SJM0	PIG Uncharacterized protein PTX3	0.746	0.777	0.278	0.354	0.97	0.98	1.00	0.99	2
O75369	F1SGJ3	PIG Uncharacterized protein FLNB	0.994	0.164	-0.01	0.447	0.96	1.00	1.00	0.99	6
P19338	I3LRH2	PIG Uncharacterized protein LOC100519984	-0.44	0.21	0.589	0.441	0.99	1.00	0.98	0.99	19
P29317	F1SUT8	PIG Uncharacterized protein EPHA2	0.223	0.748	1.057	0.298	0.99	0.99	0.91	0.99	1
-	O18866	PIG Calcium-activated potassium channel subunit alpha-1 (Fragment)	0.86	0.128	-0.19	-0.33	0.96	1.00	1.00	0.99	1
-	P55290	HUMAN Cadherin-13	-0.01	0.582	0.145	0.507	1.00	0.99	1.00	0.98	2

-	H0YI37	HUMAN ATP synthase subunit beta, mitochondrial (Fragment)	0.52	0.072	0.166	-0.07	0.99	1.00	1.00	1.00	1
Q86UE4	F1S0L8	PIG Uncharacterized protein MTDH	0.061	0.11	0.327	0.766	1.00	1.00	1.00	0.95	6
Q9Y696	I3L8R1	PIG Uncharacterized protein	-0.06	-0.07	0.339	0.295	1.00	1.00	0.99	0.99	1
-	Q2YHQ3	PIG Filamin-A (Fragment)	-0.85	-0.14	0.589	1.154	0.96	1.00	0.98	0.87	9
-	F1SFW3	PIG Guanylate cyclase (Fragment)	0.397	0.082	0.089	-0.18	0.99	1.00	1.00	0.99	1
-	P24259	PIG Natriuretic peptides A	0.098	0.088	0.114	-0.09	1.00	1.00	1.00	1.00	1
P62834	I3LF84	PIG Uncharacterized protein RAP1A	-0.64	-0.46	0.321	0.797	0.98	1.00	1.00	0.95	6
-	F1SK64	PIG Uncharacterized protein ROBO1	-0.17	-0.2	-0.18	-0.13	0.99	1.00	1.00	1.00	1
-	Q6PQZ1	PIG Aquaporin-1	0.229	0.108	-0.12	-0.02	0.99	1.00	1.00	1.00	2
-	Q7M2W6	PIG Alpha-crystallin B chain	-0.8	0.114	0.825	0.744	0.96	1.00	0.96	0.96	13
P63000	I3LFI0	PIG Uncharacterized protein (Fragment) RAC1	0.262	-0.24	0.024	0.243	0.99	1.00	1.00	0.99	3
-	P04792	HUMAN Heat shock protein beta-1	0.135	-0	0.099	0.358	0.99	1.00	1.00	0.99	1
Q8NHM5	I3LL74	PIG Uncharacterized protein (Fragment)	0.387	-0.28	-0.24	-0.31	0.99	1.00	1.00	0.99	3
-	P10775	PIG Ribonuclease inhibitor	0.05	-0.75	0.282	0.658	1.00	0.99	1.00	0.96	4
-	P13489	HUMAN Ribonuclease inhibitor	-0.27	-0.65	-0.1	0.054	0.99	0.99	1.00	1.00	1
-	F8WDM7	HUMAN Caveolin-1	0.136	-0.19	0.03	0.123	0.99	1.00	1.00	1.00	1
-	K7GS51	PIG Uncharacterized protein (Fragment) ITGA1	0.839	0.124	-0.4	-0.61	0.96	1.00	1.00	0.97	2
P98160	F1SU00	PIG Uncharacterized protein (Fragment)	-0.28	-0.8	-0.31	0.608	0.99	0.99	1.00	0.97	2
-	P23471	HUMAN Receptor-type tyrosine-protein phosphatase zeta	-0.01	-0.06	-1.04	-1.14	1.00	1.00	0.92	0.88	2
-	A0SNV3	PIG Tyrosine 3-monooxygenase/tryptophan 5-monooxygenase activation protein	-0.86	-0.88	0.181	0.714	0.96	0.97	1.00	0.96	7
-	F1RYZ1	PIG Uncharacterized protein CD151	-1.39	-0.49	-0.1	0.537	0.87	1.00	1.00	0.97	1
P98160	I3LLD8	PIG Uncharacterized protein (Fragment) LOC100626701	0.104	-0.1	-0.33	-0.29	1.00	1.00	0.99	0.99	9
-	Q9Y696	HUMAN Chloride intracellular channel protein 4	-0.13	-0.53	-0.43	0.276	0.99	1.00	1.00	0.99	4
-	Q5S1U1	PIG Heat shock protein beta-1	-0.97	-0.29	0.266	0.559	0.96	1.00	1.00	0.98	11
-	P98160	HUMAN Basement membrane-specific heparan sulfate proteoglycan core protein	-0.56	-0.67	-0.28	0.217	0.98	0.99	1.00	0.99	12
-	Q99466	HUMAN Neurogenic locus notch homolog protein 4	-0.42	-1.07	-0.81	-0.71	0.99	0.92	0.96	0.96	1
P98160	F1SU03	PIG Uncharacterized protein HSPG2	-0.15	-0.58	-0.34	0.021	0.99	0.99	1.00	1.00	19

-	H0YCW8	HUMAN Alpha-crystallin B chain (Fragment)	-0.75	-0.71	-0.65	-0.26	0.97	0.99	0.98	0.99	1
P98160	I3LAA4	PIG Uncharacterized protein	-0.78	-1.16	-0.29	0.207	0.97	0.89	1.00	0.99	5
Q13740	K7GLE8	PIG Uncharacterized protein (Fragment) ALCAM	0.904	0.38	-0.45	-0.93	0.96	1.01	0.99	0.92	5
P55290	I3LRQ0	PIG Uncharacterized protein	0.722	0.861	-0.74	-1.1	0.97	0.98	0.97	0.89	4
Q13191	F1SAH0	PIG Uncharacterized protein CBL	-1.12	-0.99	-0.4	-0.07	0.93	0.94	1.00	1.00	1
P62834	F2Z5K3	PIG Uncharacterized protein RAP1A	-0.66	-0.46	-0.52	-0.56	0.98	1.00	0.99	0.98	1
-	O02840	PIG Cadherin-5	-0.47	-0.08	-0.33	-0.24	0.99	1.00	1.00	0.99	4
-	Q9BDE7	PIG Activated leukocyte cell adhesion molecule (Fragment)	-0.85	-0.51	-0.56	-0.67	0.96	1.00	0.98	0.96	3
-	A6H560	PIG Mmp12 protein	-0.23	-0.16	-0.48	-0.9	1.00	1.00	0.99	0.92	1
-	P62745	HUMAN Rho-related GTP-binding protein RhoB	-0.07	-0.52	-0.56	-0.28	1.00	1.00	0.98	0.99	1
-	Q9XSD9	PIG Decorin	-1.7	-0.55	-0.51	0.501	0.75	0.99	0.99	0.98	13
-	A8D737	PIG T-cadherin	-0.27	0.171	-0.58	-0.7	0.99	1.00	0.98	0.96	13
P02549	F1RR78	PIG Uncharacterized protein LOC100049693	-0.89	-1.12	-0.83	-0.49	0.96	0.90	0.95	0.98	148
-	F1RSV5	PIG Forkhead box O4	-0.75	-0.56	-0.81	-0.25	0.97	0.99	0.96	0.99	1
-	Q01955	HUMAN Collagen alpha-3(IV) chain	-0.5	0.501	-0.51	-0.37	0.99	1.00	0.99	0.99	1
-	P01160	HUMAN Natriuretic peptides A	-1.37	-1.54	-0.79	-0.51	0.88	0.71	0.96	0.98	1
-	Q0QEM6	PIG ATP synthase subunit beta (Fragment)	0.153	0.159	-1.28	-1.95	0.99	1.00	0.85	0.67	37
-	Q6RVA9	PIG Caveolin-1	-1.6	-1.09	-1.11	-0.81	0.78	0.91	0.91	0.95	7
-	A4GV94	PIG Bactericidal permeability increasing protein	-1.28	-1.07	-0.95	-0.64	0.91	0.92	0.93	0.96	1
-	Q96DR7	HUMAN Rho guanine nucleotide exchange factor 26	-0.87	0.121	-1.02	-1.86	0.96	1.00	0.92	0.71	1
-	P16066	HUMAN Atrial natriuretic peptide receptor 1	-0.8	-1.03	-1.39	-2.04	0.96	0.93	0.80	0.65	1
-	P08059	PIG Glucose-6-phosphate isomerase	-1.6	-2.26	-0.87	-0.44	0.78	0.32	0.94	0.99	19
-	P12429	HUMAN Annexin A3	-1.06	-0.99	-1.35	-1.25	0.95	0.94	0.82	0.86	2
-	I3LS68	PIG Uncharacterized protein ARHGEF26	-1.77	-1.65	-1.74	-1.61	0.71	0.65	0.66	0.80	1
-	Q03135	HUMAN Caveolin-1	-0.66	-1.44	-1.89	-1.92	0.98	0.75	0.57	0.69	1
-	Q8WNW4	PIG Beta-catenin	-1.48	-1.78	-1.42	-1.11	0.84	0.57	0.79	0.88	19
-	P06576	HUMAN ATP synthase subunit beta, mitochondrial	-0.71	-0.81	-1.77	-2.75	0.98	0.98	0.65	0.38	1

-	Q9BEA8	PIG Tumor necrosis factor ligand superfamily member 6	-1.93	-2.14	-1.74	-1.38	0.62	0.38	0.66	0.83	1
-	P42771	HUMAN Cyclin-dependent kinase inhibitor 2A, isoforms 1/2/3	-2.45	-2.67	-1.57	-1.95	0.34	0.16	0.72	0.68	1
-	P28827	HUMAN Receptor-type tyrosine-protein phosphatase mu	-2.84	-2.43	-2.72	-3.45	0.17	0.25	0.21	0.14	1
-	Q29307	PIG ATPase inhibitor, mitochondrial	-3.77	-3.11	-3.79	-3.16	0.02	0.06	0.02	0.22	7

Regulation of gene expression

Human equivalent Ac. No.	Accession No.	Protein	Zq (log2 FC) 120	1	4	7	FDR 120	1	4	7	No. peptides
-	P62495	HUMAN Eukaryotic peptide chain release factor subunit 1	1.202	0.88	1.131	1.201	0.92	0.97	0.90	0.87	4
-	K9IW91	PIG GCN1 general control of amino-acid synthesis 1-like 1	0.123	1.455	2.566	1.199	0.99	0.74	0.25	0.87	2
-	P21333	HUMAN Filamin-A	-0.08	0.1	1.221	2.285	1.00	1.00	0.86	0.54	60
-	A6M931	PIG Eukaryotic initiation factor 4A-III	-0.78	-0.39	1.27	1.894	0.97	1.01	0.85	0.69	6
-	D3K5N7	PIG ELAV (Embryonic lethal, abnormal vision, Drosophila)-like 1	1.296	0.864	0.741	0.884	0.90	0.97	0.97	0.93	4
-	Q13144	HUMAN Translation initiation factor eIF-2B subunit epsilon	1.552	1.443	0.786	0.019	0.81	0.75	0.96	1.00	1
-	F1RFM6	PIG Uncharacterized protein (Fragment) EIF2AK1	0.467	0.957	0.971	1.24	0.99	0.95	0.93	0.86	1
-	F1RI41	PIG Eukaryotic translation initiation factor 3 subunit K	0.95	0.482	0.492	0.621	0.96	1.00	0.99	0.96	2
Q04637	I3LMH4	PIG Uncharacterized protein EIF4G1	0.27	0.154	1.092	1.275	0.99	1.00	0.90	0.85	16
-	Q9Y3I1	HUMAN F-box only protein 7	-0.99	0.645	-0.2	1.517	0.96	0.99	1.00	0.81	1
-	M3TYC1	PIG Eukaryotic translation initiation factor 3 subunit B	0.217	0.38	0.771	0.752	0.99	1.01	0.97	0.96	10
-	F2Z5J8	PIG Eukaryotic translation initiation factor 2 subunit 1	0.501	-0.02	0.384	0.66	0.99	1.00	0.99	0.96	11
Q92616	F1RJK5	PIG Uncharacterized protein (Fragment) GCN1L1	0.695	1.452	1.205	-0.55	0.98	0.75	0.87	0.97	2
-	F1S1J9	PIG Eukaryotic translation initiation factor 3 subunit H	0.132	-0.24	0.371	0.69	0.99	1.00	0.99	0.96	3
-	Q2YHQ3	PIG Filamin-A (Fragment)	-0.85	-0.14	0.589	1.154	0.96	1.00	0.98	0.87	9
P23588	I3LFM8	PIG Uncharacterized protein EIF4B	-0.71	-0.37	0.231	0.215	0.98	1.01	1.00	0.99	1
-	P60228	HUMAN Eukaryotic translation initiation factor 3 subunit E	0.303	0.348	0.218	0.389	1.00	1.01	1.00	0.99	1
Q9NZJ5	K7GP80	PIG Uncharacterized protein EIF2AK3	0.228	-0.38	-0.02	0.094	1.00	1.01	1.00	1.00	1
-	H0YEI7	HUMAN Eukaryotic translation initiation factor 4 gamma 2 (Fragment)	0.102	-1.19	-0.82	-0.64	1.00	0.88	0.96	0.96	1

P06730	F1S0B9	PIG Uncharacterized protein EIF4E	-1.25	-0.87	0.616	0.877	0.91	0.97	0.98	0.93	3
-	M3VK13	PIG Eukaryotic translation initiation factor 5 tv1	-0.44	-0.32	0.095	0.297	0.99	1.00	1.00	0.99	5
-	P23588	HUMAN Eukaryotic translation initiation factor 4B	-0.99	-0.33	0.013	0.22	0.96	1.00	1.00	0.99	3
-	I3LN50	PIG Uncharacterized protein EIF2B3	-1.09	-1.5	-0.99	-0.04	0.94	0.72	0.92	1.00	1
-	D2KMN4	PIG Glial cell-line derived neurotrophic factor (Fragment)	-1.47	-1.63	-1.22	-0.94	0.84	0.66	0.86	0.92	1
-	Q9P2K8	HUMAN Eukaryotic translation initiation factor 2-alpha kinase 4	-0.92	-1.05	-1.63	-1.73	0.96	0.93	0.70	0.75	1

Proteolysis

Human equivalent Ac. No.	Accession No.	Protein	Zq (log2 FC)				FDR				No. peptides
			120	1	4	7	120	1	4	7	
	O15072	HUMAN A disintegrin and metalloproteinase with thrombospondin motifs 3	0.919	6.026	6.044	2.485	0.957	5E-07	1E-06	0.47	1
	Q8SPS7	PIG Haptoglobin	3.055	3.505	4.406	2.82	0.11	0.022	0.002	0.361	14
F1S0J2	F1S0J2	PIG Uncharacterized protein C4BPA	2.001	2.623	2.88	2.022	0.578	0.175	0.152	0.654	4
F1STZ3	F1STZ3	PIG Uncharacterized protein C1QC	2.553	2.481	2.407	1.545	0.298	0.226	0.317	0.799	1
	Q69DL0	PIG Complement C1q subcomponent subunit A	0.889	2.606	3.095	1.688	0.957	0.179	0.099	0.762	4
	P01025	PIG Complement C3	2.501	1.593	1.876	1.685	0.32	0.675	0.582	0.766	83
P02748	F1SMJ6	PIG Uncharacterized protein (Fragment) C9	0.764	1.829	2.012	1.352	0.968	0.546	0.513	0.829	7
	A5D9P0	PIG Ring finger protein 1	1.541	1.903	1.613	2.745	0.81	0.505	0.7	0.376	1
P01024	I3LTB8	PIG Uncharacterized protein	1.888	1.799	1.664	1.131	0.632	0.562	0.687	0.881	2
	Q9TUQ3	PIG Complement component C7	1.658	1.551	1.261	0.905	0.757	0.705	0.851	0.925	7
F1S790	F1S790	PIG Uncharacterized protein C8B	1.473	1.677	1.526	0.922	0.842	0.638	0.743	0.921	3
	I3L8K6	PIG E3 ubiquitin-protein ligase	1.44	2.889	2.067	0.225	0.852	0.102	0.485	0.991	1
	B0LFE9	PIG Complement component 4	1.503	1.43	1.345	1.384	0.828	0.763	0.821	0.826	38
	F1SPT0	PIG Ubiquitin carboxyl-terminal hydrolase	2.268	1.108	0.843	0.346	0.43	0.9	0.953	0.99	1
K7GQI7	K7GQI7	PIG Uncharacterized protein PYCARD	1.114	1.225	1.766	1.394	0.934	0.861	0.649	0.825	4
	Q6VPV1	PIG Complement component C5	1.127	1.588	1.7	1.062	0.934	0.678	0.673	0.902	20

	A0SEH3	PIG Complement component C8G	1.471	1.551	1.249	0.718	0.841	0.706	0.854	0.963	5
	Q9N2I1	PIG Caspase-1	0.133	1.248	1.213	1.396	0.993	0.856	0.863	0.824	1
E7EMW7	I3LN69	PIG Uncharacterized protein (Fragment)	0.889	1.612	1.847	1.31	0.957	0.664	0.602	0.84	1
	Q69DK8	PIG Complement C1s subcomponent	1.744	1.63	0.966	0.578	0.72	0.658	0.928	0.972	4
	F1SMJ1	PIG Complement component C7 (Fragment)	0.624	1.35	1.158	1.129	0.985	0.807	0.887	0.88	1
F1SUE6	F1SUE6	PIG Uncharacterized protein TYSND1	1.556	0.895	1.198	1.283	0.804	0.967	0.869	0.848	1
	K9J4S2	PIG E3 ubiquitin-protein ligase TRIM11	0.881	1.476	1.577	1.615	0.957	0.732	0.722	0.791	1
F1SMI8	F1SMI8	PIG Uncharacterized protein C6	0.829	1.231	1.636	1.397	0.963	0.863	0.697	0.823	2
	Q9UPU5	HUMAN Ubiquitin carboxyl-terminal hydrolase 24	1.66	1.187	1.224	1.536	0.759	0.879	0.861	0.804	1
	F1SBA5	PIG Proteasome subunit alpha type	0.333	0.715	1.508	1.06	0.997	0.992	0.749	0.903	2
Q9UBT2	F1RNU6	PIG Uncharacterized protein UBA2	1.14	0.983	0.734	0.693	0.933	0.943	0.969	0.963	1
	A1E295	PIG Cathepsin B	0.227	0.213	1.518	2.323	0.996	1.001	0.747	0.522	6
	Q96T88	HUMAN E3 ubiquitin-protein ligase UHRF1	-0.16	0.136	1.062	1.923	0.99	1.004	0.91	0.684	1
F1S0J3	F1S0J3	PIG Uncharacterized protein C4BPB	-0.28	1.227	2.281	1.471	0.995	0.863	0.389	0.811	1
	P20618	HUMAN Proteasome subunit beta type-1	4.728	4.26	-0.39	-0.22	5E-04	0.002	0.994	0.991	1
F1RSC3	F1RSC3	PIG Uncharacterized protein SCPEP1	0.989	0.406	0.882	1.552	0.955	1.006	0.942	0.799	6
F1SU29	F1SU29	PIG Uncharacterized protein (Fragment) RANBP2	0.419	0.211	0.599	1.047	0.99	1.001	0.984	0.904	2
	K9J6J4	PIG Caspase-7 isoform alpha	1.101	0.318	0.463	0.811	0.937	0.999	0.988	0.947	3
	P0C0L4	HUMAN Complement C4-A	-0.26	0.046	0.268	1.351	0.993	1.001	1.001	0.829	1
Q99460	F1SMW9	PIG Uncharacterized protein PSMD1	1.099	0.749	0.758	0.83	0.939	0.989	0.966	0.942	6
Q86VP6	F1RYI3	PIG Uncharacterized protein CAND1	0.815	0.456	0.667	0.797	0.961	1.001	0.974	0.949	11
	F1SKI2	PIG Ubiquitin carboxyl-terminal hydrolase (Fragment)	1.268	0.898	0.643	0.477	0.908	0.968	0.98	0.984	1
	Q9UP79	HUMAN A disintegrin and metalloproteinase with thrombospondin motifs 8	0.998	3.322	0.015	-0.82	0.957	0.037	1.001	0.943	1
	P15145	PIG Aminopeptidase N	5.262	2.949	-0.21	-0.64	5E-05	0.09	0.999	0.964	1
	B6DT15	PIG Ubiquitin carboxyl-terminal hydrolase	0.592	-0.41	0.826	1.094	0.987	1.006	0.954	0.89	2
F1S6S9	F1S6S9	PIG Uncharacterized protein PRTN3	1.217	1.718	0.811	-0.17	0.919	0.612	0.958	0.996	1

	P49720	HUMAN Proteasome subunit beta type-3	0.515	1.23	1.61	0.449	0.989	0.862	0.701	0.986	2
F1STZ1	F1STZ1	PIG Uncharacterized protein C1QB	-0.17	0.685	1.641	0.911	0.989	0.992	0.695	0.923	3
	Q29384	PIG Proteasome subunit beta type-4 (Fragment)	1.28	0.45	0.829	0.79	0.91	1.003	0.954	0.95	2
I3LCC6	I3LCC6	PIG Uncharacterized protein (Fragment) PSMB3	1.337	0.941	1.05	0.587	0.895	0.955	0.91	0.971	1
O75155	I3LPW6	PIG Uncharacterized protein (Fragment)	1.257	1.437	0.684	-0.04	0.908	0.758	0.971	1	2
Q9UKU6	F1SH20	PIG Uncharacterized protein TRHDE	0.536	0.018	0.634	0.985	0.984	0.999	0.981	0.919	1
O75155	I3LES1	PIG Uncharacterized protein	0.67	0.315	0.596	0.789	0.982	1	0.983	0.95	1
	P61960	HUMAN Ubiquitin-fold modifier 1	-0.09	0.61	0.756	0.85	0.997	0.994	0.967	0.939	1
	Q5VWC4	HUMAN 26S proteasome non-ATPase regulatory subunit 4	1.258	0.539	0.294	0.058	0.909	0.994	0.999	0.998	1
	Q9Y4X5	HUMAN E3 ubiquitin-protein ligase ARIH1	1.17	0.616	0.564	0.424	0.925	0.993	0.982	0.988	1
O14818	I3LVJ7	PIG Uncharacterized protein (Fragment) PSMA7	0.261	-0.25	0.881	0.946	0.993	1	0.943	0.918	3
	Q6SEG5	PIG Ubiquitin carboxyl-terminal hydrolase isozyme L1	1.161	0.377	0.407	0.97	0.925	1.006	0.996	0.917	2
P67812	I3LF38	PIG Uncharacterized protein (Fragment) SEC11A	0.399	0.542	0.76	0.791	0.991	0.992	0.967	0.952	2
	Q8WU17	HUMAN E3 ubiquitin-protein ligase RNF139	0.069	0.513	0.738	0.88	0.997	0.998	0.968	0.93	1
F1RNP8	F1RNP8	PIG Uncharacterized protein ANAPC5	1.053	1.407	0.273	-0.28	0.946	0.777	1.001	0.987	1
F1SU85	F1SU85	PIG Uncharacterized protein (Fragment) ANAPC1	0.818	0.636	0.131	-0.07	0.962	0.994	1.002	0.997	1
	O14818	HUMAN Proteasome subunit alpha type-7	0.405	-0.31	0.563	0.798	0.991	1	0.981	0.949	4
	P35750	PIG Calpain-1 catalytic subunit	0.312	0.258	0.474	0.457	0.997	0.999	0.987	0.988	14
F1RG00	F1RG00	PIG Uncharacterized protein (Fragment) PSMA5	1.215	0.329	0.188	0.139	0.92	1.005	1.001	0.996	1
	P60900	HUMAN Proteasome subunit alpha type-6	0.358	0.224	0.152	0.097	0.996	1.002	1.001	0.999	2
	Q9H0M0	HUMAN NEDD4-like E3 ubiquitin-protein ligase WWP1	-0.19	-0.24	0.143	1.325	0.991	1.002	1.002	0.833	1
	A0SEH0	PIG Complement component C6	0.592	0.5	0.728	0.5	0.986	0.999	0.969	0.98	7
	F4ZS20	PIG Ubiquitin-conjugating enzyme E2L 6	0.26	0.025	0.524	0.064	0.993	1	0.985	0.998	2
	P00736	HUMAN Complement C1r subcomponent	-0.56	0.502	0.99	0.49	0.984	0.999	0.924	0.981	1
	A5D9J4	PIG Proteasome subunit beta type	0.54	0.562	0.543	0.473	0.985	0.99	0.981	0.984	1
	Q32YV9	PIG Proteasome 26S subunit non-ATPase 4	0.532	0.871	0.496	0.493	0.986	0.971	0.988	0.98	6
	P61086	HUMAN Ubiquitin-conjugating enzyme E2 K	0.382	-0.46	0.005	0.612	0.994	1.002	0.999	0.966	4

	P19205	PIG Acylamino-acid-releasing enzyme	0.524	0.781	0.548	0.012	0.987	0.985	0.982	1.001	5
	F1ST02	PIG Proteasome subunit beta type	-0.11	-0.65	0.401	0.505	0.996	0.993	0.995	0.978	2
	H0Y4S7	HUMAN RanBP-type and C3HC4-type zinc finger-containing protein 1 (Fragment)	0.357	0.675	0.268	0.268	0.996	0.991	1	0.986	1
Q9HB71	F1S710	PIG Uncharacterized protein CACYBP	-0.4	-0.1	0.506	0.796	0.991	1.002	0.987	0.95	6
O00231	F1RKW8	PIG Uncharacterized protein (Fragment) PSMD11	0.18	-0.12	0.345	0.46	0.989	1.003	0.994	0.986	10
	A1XQU1	PIG Proteasome subunit beta type-7	-0.16	-0.43	0.216	0.423	0.99	1.006	1	0.988	5
	Q863Z0	PIG Proteasome activator complex subunit 2	-0.18	-0.22	0.396	0.824	0.989	1.001	0.995	0.943	7
	O00231	HUMAN 26S proteasome non-ATPase regulatory subunit 11	-0.62	-0.25	0.63	0.82	0.983	1	0.98	0.942	1
P54725	F1SD96	PIG Uncharacterized protein (Fragment) RAD23A	-0.01	0.292	0.624	0.224	1	1	0.978	0.99	5
	A7XUJ6	PIG TNF receptor-associated factor 6	0.197	-0.65	0.55	0.873	0.991	0.992	0.982	0.932	1
P35998	I3LJ30	PIG Uncharacterized protein	0.343	0.073	0.362	0.451	0.998	1.003	0.996	0.986	11
	G3DRF8	PIG Proteasome subunit beta type	0.603	-0.18	0.215	0.457	0.986	1.003	1	0.987	4
Q16401	F1SME6	PIG Uncharacterized protein	0.933	0.621	0.011	-0.15	0.955	0.993	1	0.997	3
	Q9Y4E6	HUMAN WD repeat-containing protein 7	-0.27	-0.01	0.2	-0.12	0.993	0.999	1	0.998	1
	P62197	PIG 26S protease regulatory subunit 8	0.485	0.442	0.246	0.176	0.985	1.005	1.001	0.993	11
Q9BZ11	F1S8C4	PIG Uncharacterized protein ADAM33	0.608	-0.31	-0.29	-0.36	0.986	1	1.001	0.989	1
P51665	I3L6B9	PIG Uncharacterized protein PSMD7	-0.3	0.152	0.553	0.47	0.997	1.004	0.981	0.985	1
	B6VAQ2	PIG Proteasome subunit alpha type 6	0.316	-0.25	0.257	0.417	0.997	1	1.001	0.988	6
O00232	F1RSM2	PIG Uncharacterized protein PSMD12	-0.51	-0.4	0.643	0.445	0.988	1.006	0.98	0.987	3
Q9UNM6	F1RGC9	PIG Uncharacterized protein PSMD13	0.884	0.424	-0.07	-0	0.956	1.006	0.998	1	6
F1SU56	F1SU56	PIG Uncharacterized protein (Fragment) USP54	0.483	1.02	0.179	0.191	0.985	0.934	1	0.992	1
	P63053	PIG Ubiquitin-60S ribosomal protein L40	-0.81	-0.23	0.438	0.706	0.962	1.001	0.992	0.961	9
	I3LQ51	PIG Proteasome subunit beta type	0.294	-0.21	0.161	0.221	0.996	1.001	1.002	0.99	8
	D7RA34	PIG Autophagy related 12-like protein	0.299	-0.26	-0.27	-0.17	0.995	0.999	1	0.995	1
P07357	F1S788	PIG Uncharacterized protein (Fragment) C8A	0.717	0.082	0.21	0.226	0.974	1.002	0.999	0.992	5
	F1SKD5	PIG Ubiquitin carboxyl-terminal hydrolase	0.544	0.602	0.207	-0	0.984	0.992	0.999	1	1
P48556	F1RKI9	PIG Uncharacterized protein PSMD8	-0.12	0.28	0.499	0.317	0.994	0.998	0.987	0.99	4

	P25786	HUMAN Proteasome subunit alpha type-1	-0.37	-0.79	0.17	0.368	0.996	0.985	1.002	0.989	13
	I3LQD3	PIG Calpain-2 catalytic subunit (Fragment)	0.423	0.034	-0.01	0.047	0.99	1.001	0.999	1	1
	P28066	HUMAN Proteasome subunit alpha type-5	1.092	0.072	0.045	0.101	0.939	1.002	1.002	0.998	7
	O97506	PIG Kallikrein	0.582	-0.7	-0.27	0.079	0.984	0.991	1	0.998	4
	Q64L94	PIG Proteasome activator complex subunit 1	-0.21	-0.13	0.337	0.437	0.992	1.004	0.994	0.987	14
F1SHV8	F1SHV8	PIG Uncharacterized protein UBE3C	1.512	0.711	-0.5	-0.75	0.828	0.992	0.988	0.958	1
P55072	F1SIH8	PIG Uncharacterized protein LOC100516776	-0.11	-0.27	0.246	0.408	0.995	0.999	1.002	0.988	1
	P25787	HUMAN Proteasome subunit alpha type-2	0.104	-0.34	0.035	0.142	0.996	1.007	1	0.997	3
	P60468	HUMAN Protein transport protein Sec61 subunit beta	0.273	-0.62	-0.23	0.16	0.994	0.993	1.001	0.998	1
	P63279	HUMAN SUMO-conjugating enzyme UBC9	-0.33	-0.34	0.195	0.421	0.997	1.006	1	0.988	2
	F1SLS7	PIG Autophagy-related protein 3	0.035	-0.17	0.115	0.251	0.999	1.004	1.002	0.987	3
Q99460	I3LB90	PIG Uncharacterized protein	0.35	-0.1	-0.12	-0.1	0.995	1.002	1.002	0.999	4
	A5D9J0	PIG Proteasome (Prosome, macropain) subunit, beta type, 9 (Fragment)	-0.29	-0.29	-0.33	-0.1	0.995	1	0.995	0.999	1
	P03974	PIG Transitional endoplasmic reticulum ATPase	-0.7	-0.7	0.377	0.628	0.977	0.991	0.994	0.965	30
	O43184	HUMAN Disintegrin and metalloproteinase domain-containing protein 12	-1.59	-0.31	0.08	0.674	0.783	1	0.998	0.963	1
Q15008	I3LS80	PIG Uncharacterized protein LOC100739334	-0.34	-0.43	-0.04	0.251	0.999	1.007	1.002	0.987	2
F1SM56	F1SM56	PIG Uncharacterized protein SEC11C	0.632	0.274	-0.27	-0.14	0.983	0.998	1.001	0.996	1
	P62191	HUMAN 26S protease regulatory subunit 4	0.185	-0.35	-0.04	0.159	0.989	1.007	1.001	0.998	9
	Q99460	HUMAN 26S proteasome non-ATPase regulatory subunit 1	-0.13	1.048	-0.2	-0.48	0.993	0.926	1.001	0.984	1
A0AVT1	F1RVE8	PIG Uncharacterized protein UBA6	-0.43	-0.48	-0.35	-0.81	0.99	1.002	0.995	0.947	1
	K9J6M4	PIG E3 ubiquitin-protein ligase UBR4	0.654	0.081	-0.43	-0.3	0.984	1.002	0.995	0.989	2
Q15008	F1SGF1	PIG Uncharacterized protein PSMD6	-0.47	-0.16	0.106	0.237	0.987	1.004	1	0.99	3
O00487	F1RPQ3	PIG Uncharacterized protein PSMD14	-0.15	-0.48	-0.13	0.059	0.994	1.001	1.002	0.997	2
	H0YBB4	HUMAN E3 ubiquitin-protein ligase UBR5 (Fragment)	-0.42	-0.51	-0.24	-0.56	0.989	0.998	1.001	0.975	2
	O15484	HUMAN Calpain-5	0.635	0.211	-0.18	-0.2	0.985	1.001	1.002	0.991	1
	I3L945	PIG Ubiquitin carboxyl-terminal hydrolase (Fragment)	-0.19	-0.22	-0.03	-0.03	0.99	1.001	1	1	5
	Q9Y3C8	HUMAN Ubiquitin-fold modifier-conjugating enzyme 1	0.169	-0.33	0.027	0.144	0.99	1.004	1	0.997	1

	K9IVF7	PIG X-prolyl aminopeptidase (Aminopeptidase P) 1, soluble	-0.01	-0.33	-0.3	-0.37	1	1.005	0.997	0.989	6
	Q5T4S7	HUMAN E3 ubiquitin-protein ligase UBR4	-0.41	1.103	0.947	0.54	0.991	0.902	0.932	0.973	1
	P04574	PIG Calpain small subunit 1	-0.55	-0.61	-0.01	0.312	0.985	0.994	0.998	0.988	9
	P25789	HUMAN Proteasome subunit alpha type-4	-0.15	-0.62	-0.22	0.135	0.993	0.994	1.002	0.998	1
Q8TBC4	F1SFQ0	PIG Uncharacterized protein UBA3	0.372	-0.74	-0.52	-0.11	0.996	0.99	0.986	1	1
P54725	F1SP32	PIG Uncharacterized protein RAD23B	-0.47	-0.21	0.288	0.839	0.989	1.001	1.002	0.939	10
	Q06AB3	PIG Ubiquitin carboxyl-terminal hydrolase isozyme L3	0.31	-0.24	-0.29	0.168	0.996	1.003	1.002	0.996	1
Q16401	I3L9N5	PIG Uncharacterized protein LOC100626266	-0.35	-0.46	-0.21	-0.11	0.995	1.001	0.999	1	2
I3LHM5	I3LHM5	PIG Uncharacterized protein USP53	0.019	0.326	0.036	-0.69	0.999	1.003	1	0.963	2
	P59510	HUMAN A disintegrin and metalloproteinase with thrombospondin motifs 20	0.446	0.106	-0.51	-0.54	0.986	1.002	0.985	0.974	1
	Q06AT3	PIG Ubiquitin carboxyl-terminal hydrolase isozyme L5	0.406	-0.07	-0.73	-0.79	0.991	1.002	0.97	0.951	1
	P68036	HUMAN Ubiquitin-conjugating enzyme E2 L3	0.058	-0.53	-0.01	0.328	0.997	0.997	0.999	0.991	4
	F1RHF0	PIG Ubiquitin carboxyl-terminal hydrolase isozyme L3 (Fragment)	-0.77	-0.46	-0.53	-0.19	0.968	1.004	0.985	0.992	2
	P49459	HUMAN Ubiquitin-conjugating enzyme E2 A	-0.49	-0.39	0.041	0.434	0.986	1.004	1.001	0.987	1
	F1SAF6	PIG Ubiquitin carboxyl-terminal hydrolase	-0.39	-0.35	-0.52	-0.51	0.992	1.006	0.985	0.977	1
	F1SLU6	PIG Ubiquitin carboxyl-terminal hydrolase	-0.93	-1.13	-0.23	0.176	0.958	0.897	1.001	0.993	14
	P43367	PIG Calpain-2 catalytic subunit (Fragment)	-0.28	-0.17	-0.27	0.217	0.994	1.004	1.001	0.99	9
	P37111	PIG Aminoacylase-1	-0.05	-0.86	-0.76	-0.43	0.998	0.974	0.967	0.987	8
Q9NVA1	I3L662	PIG Uncharacterized protein LOC100622542	0.269	0.33	-0.76	-0.91	0.993	1.006	0.967	0.921	1
I3LG40	I3LG40	PIG Uncharacterized protein (Fragment) SENP7	0.142	-0.8	-0.83	-0.45	0.994	0.985	0.954	0.987	1
	F1SSL6	PIG Proteasome subunit alpha type	-0.54	-0.53	-0.31	-0.2	0.984	0.997	0.996	0.991	11
O75150	F1RG77	PIG Uncharacterized protein RNF40	-0.56	-0.97	-0.76	-0.32	0.983	0.944	0.967	0.99	1
F1SB05	F1SB05	PIG Uncharacterized protein ADAM30	-0.72	-0.66	-0.22	0.187	0.975	0.99	1.001	0.994	1
	Q9ULZ3	HUMAN Apoptosis-associated speck-like protein containing a CARD	-0.25	-0.12	-0.42	-0.8	0.994	1.003	0.996	0.949	1
	Q8NDL9	HUMAN Cytosolic carboxypeptidase-like protein 5	-0.08	-0.58	-0.47	0.06	0.996	0.991	0.987	0.998	1
	F1RYL9	PIG Ubiquitin carboxyl-terminal hydrolase	0.657	0.407	-0.53	-1.41	0.983	1.006	0.985	0.823	1
Q5XPI4	F1SPR6	PIG Uncharacterized protein RNF123	-0.69	-0.28	-0.14	-0.31	0.977	1	1.002	0.988	5

	Q29096	PIG Proteasome subunit C9-like protein (Fragment)	-1.62	-1.06	-0.08	0.248	0.773	0.925	0.998	0.988	5
	Q9H0E7	HUMAN Ubiquitin carboxyl-terminal hydrolase 44	-0.26	-0.28	-0.73	-0.5	0.993	0.999	0.969	0.979	1
P51965	I3LQC1	PIG Uncharacterized protein (Fragment) UBE2E1	-0.92	-0.98	-0.66	-0.5	0.956	0.945	0.978	0.979	1
Q969Q1	I3L5M6	PIG Uncharacterized protein TRIM63	-1.57	-1.65	-1.14	-0.55	0.795	0.65	0.897	0.974	1
	I3LHE4	PIG Coronin	-0.46	-1.41	-1.36	-0.96	0.987	0.779	0.818	0.92	1
	H9TUB4	PIG UME3A	-1.35	-0.79	-0.55	-0.5	0.89	0.984	0.981	0.98	1
	Q06AA9	PIG Ubiquitin-conjugating enzyme E2 D2	-0.95	-1.17	-0.37	-0.08	0.955	0.884	0.994	0.999	2
Q13404	I3L6T2	PIG Uncharacterized protein (Fragment) UBE2V2	-0.96	-0.75	-0.3	-0.27	0.953	0.989	0.996	0.987	4
F1RM03	F1RM03	PIG Uncharacterized protein SAE1	-0.38	-0.33	-0.53	-0.44	0.994	1.006	0.985	0.986	1
O75439	F1SKM0	PIG Uncharacterized protein UQCRC1	-0.51	-0.18	-0.89	-1.42	0.987	1.003	0.942	0.82	22
Q04323	I3LUD5	PIG Uncharacterized protein UBXN1	-1.2	-0.76	-0.73	-0.27	0.926	0.986	0.97	0.987	3
	Q6ZT12	HUMAN E3 ubiquitin-protein ligase UBR3	-0.49	-0.79	-1.18	-1.55	0.984	0.984	0.879	0.8	1
	Q8NFA0	HUMAN Ubiquitin carboxyl-terminal hydrolase 32	-1.34	0.337	0.633	-0.78	0.894	1.006	0.98	0.949	2
	Q9Y4W6	HUMAN AFG3-like protein 2	-0.5	-1.24	-1.26	-0.9	0.987	0.859	0.852	0.925	1
Q96K76	F1S6W7	PIG Uncharacterized protein (Fragment) USP47	-0.35	-0.85	-1.03	-0.92	0.996	0.974	0.915	0.922	1
	Q9P275	HUMAN Ubiquitin carboxyl-terminal hydrolase 36	-1.33	-0.52	-1.64	-1.21	0.897	0.998	0.695	0.869	1
Q92890	F1RK61	PIG Uncharacterized protein UFD1L	-0.07	-0.63	-1.18	-1.55	0.997	0.992	0.878	0.799	1
P61960	F1RS33	PIG Uncharacterized protein (Fragment) LOC100522509	-0.54	-0.62	-0.83	-0.62	0.985	0.993	0.954	0.965	1
Q8NDL9	I3LU97	PIG Uncharacterized protein AGBL5	0.048	0.228	-1.73	-1.59	0.998	1.001	0.666	0.801	2
	P10323	HUMAN Acrosin	-0.87	-0.24	-0.98	-0.41	0.958	1.001	0.924	0.988	1
Q9Y4W6	I3LLQ8	PIG Uncharacterized protein (Fragment) AFG3L2	-0.5	-1.52	-1.12	-0.98	0.987	0.715	0.905	0.918	6
	Q13404	HUMAN Ubiquitin-conjugating enzyme E2 variant 1	-1.43	-2.13	-0.94	-0.51	0.858	0.382	0.932	0.977	2
Q9GZZ9	F1SNT0	PIG Uncharacterized protein (Fragment) UBA5	-1.55	-1.42	-0.82	-0.42	0.809	0.772	0.958	0.988	1
	P22314	HUMAN Ubiquitin-like modifier-activating enzyme 1	-0.71	-0.7	-1.1	-0.42	0.976	0.992	0.906	0.988	1
F1SDM3	F1SDM3	PIG Uncharacterized protein (Fragment) ADAM12	-0.2	0.541	-1.95	-2.29	0.992	0.993	0.545	0.532	1
	H9BR48	PIG Thrombospondin type 1 motif disintegrin-like and metallopeptidase	-1.51	-0.97	-1.5	-1.5	0.828	0.944	0.75	0.811	1
Q8NHY2	F1S705	PIG Uncharacterized protein RFWD2	-2.42	-2.26	-1.56	-0.72	0.356	0.322	0.726	0.963	1

O00762	F1SC78	PIG Uncharacterized protein UBE2C	-1.6	-1.23	-1.08	-1.29	0.78	0.862	0.908	0.844	1
	P12821	HUMAN Angiotensin-converting enzyme	-1.91	-1.96	-1.64	-1.16	0.623	0.475	0.694	0.87	1
F1RTR7	F1RTR7	PIG Uncharacterized protein YOD1	-1.69	-3.61	-1.87	-1.26	0.751	0.016	0.583	0.85	1
	Q2TAM5	HUMAN RELA protein	-1.14	-1.48	-1.18	-0.46	0.933	0.729	0.878	0.986	1
	H0YCB4	HUMAN Transcription factor p65 (Fragment)	-1.13	-1.83	-2.34	-1.88	0.932	0.543	0.348	0.697	1
Q14669	I3LK74	PIG Uncharacterized protein	-2.18	-1.89	-1.59	-1.32	0.48	0.515	0.715	0.835	1
	Q7Z569	HUMAN BRCA1-associated protein	-1.27	-2.64	-2.15	-1.81	0.91	0.17	0.449	0.735	1

Supplementary Table S4. Protein expression changes in the functionally annotated categories within cluster d.

Human equivalent Ac. No.	Accession No.	Mitochondrial complexes	Zq (log2 FC)				FDR				No. peptides
		Protein	120	1	4	7	120	1	4	7	
-	Q29307	PIG ATPase inhibitor, mitochondrial	-3.77	-3.11	-3.79	-3.16	0.02	0.06	0.02	0.22	7
-	Q35914	PIG ATP synthase protein 8	0.208	-1.69	-5.05	-4.95	0.99	0.63	0.00	0.00	1
-	I3LER5	PIG Cytochrome c oxidase subunit 4 isoform 1, mitochondrial	-3.4	-1.84	-2.42	-3.06	0.05	0.54	0.31	0.23	3
-	Q5S3G4	PIG Cytochrome c oxidase subunit 5B, mitochondrial	-2.29	-0.99	-2.02	-2.71	0.42	0.94	0.51	0.38	9
-	P13618	PIG ATP synthase-coupling factor 6, mitochondrial	-1.04	0.279	-1.77	-2.24	0.95	1.00	0.64	0.55	11
-	C9JJT5	HUMAN Protein ATP5J2-PTCD1	-2.83	-1.77	-2.03	-2.17	0.17	0.58	0.51	0.59	1
P14854	F1RLH7	PIG Uncharacterized protein COX6B	-1.7	-1.13	-2.28	-2.2	0.75	0.90	0.39	0.58	5
P20674	F1SJ34	PIG Uncharacterized protein COX5A	-1.01	-0.18	-1.51	-2.39	0.96	1.00	0.75	0.51	10
-	P14927	HUMAN Cytochrome b-c1 complex subunit 7	-1.24	-0.89	-1.2	-1.87	0.91	0.97	0.87	0.70	4
-	A9LGH7	PIG ATP synthase protein 8	-0.45	-2.74	-1.96	-1.49	0.99	0.14	0.54	0.81	1
O95299	F1SIS9	PIG Uncharacterized protein NDUFA10	-1.23	-0.77	-1.76	-2.13	0.91	0.99	0.65	0.60	13
-	P24964	PIG Cytochrome b	-1.91	-1.7	-2.35	-2.34	0.62	0.62	0.34	0.53	5
O75964	F1SAK6	PIG Uncharacterized protein ATP5L	-1.68	-1.05	-2.03	-2.17	0.75	0.93	0.51	0.59	6
Q16718	F1SLY2	PIG Uncharacterized protein NDUFA5	-1.29	-0.52	-1.83	-2.23	0.91	1.00	0.61	0.56	4
-	Q2EN81	PIG ATP synthase subunit O, mitochondrial	-1.26	-0.65	-1.77	-2.06	0.91	0.99	0.65	0.64	14
-	P06576	HUMAN ATP synthase subunit beta, mitochondrial	-0.71	-0.81	-1.77	-2.75	0.98	0.98	0.65	0.38	1
P51970	F1SLR1	PIG Uncharacterized protein NDUFA8	-1.32	-0.68	-1.58	-1.93	0.90	0.99	0.72	0.68	6
O14949	F1RI18	PIG Uncharacterized protein UQCRQ	-2.08	-1.64	-1.6	-1.79	0.53	0.66	0.71	0.73	6
P49821	F1RVN1	PIG Uncharacterized protein NDUFV1	-1.15	-0.8	-1.44	-1.73	0.93	0.99	0.78	0.75	17

-	A5GFX4	PIG ATP synthase, H+ transporting, mitochondrial F1 complex, epsilon subunit	-1.62	-1.41	-2	-1.93	0.77	0.77	0.52	0.68	2
O43920	F1SV23	PIG Uncharacterized protein NDUFS5	-1.62	-1.09	-1.24	-1.45	0.77	0.91	0.86	0.82	4
-	F1S6Q7	PIG ATP synthase subunit delta, mitochondrial	-0.07	0.634	-1.57	-2.08	1.00	0.99	0.72	0.63	2
-	F1RNZ1	PIG Cytochrome b-c1 complex subunit Rieske, mitochondrial	-1.31	-0.66	-1.58	-2.27	0.90	0.99	0.72	0.55	14
-	O00217	HUMAN NADH dehydrogenase [ubiquinone] iron-sulfur protein 8, mitochondrial	-0.38	-0.37	-1.58	-2.2	0.99	1.01	0.72	0.58	7
-	F1SJP6	PIG NADH dehydrogenase [ubiquinone] 1 alpha subcomplex subunit 6 (Fragment)	-0.72	-0.73	-1.51	-2.17	0.97	0.99	0.75	0.60	7
-	F1STY1	PIG NADH dehydrogenase [ubiquinone] 1 subunit C2	-1.97	-1.05	-1.35	-1.87	0.59	0.93	0.82	0.71	6
-	K7GMN1	PIG Uncharacterized protein COX7B	-1.89	-1.68	-1.71	-1.74	0.63	0.64	0.67	0.75	2
O75489	F1SIF2	PIG Uncharacterized protein NDUFS3	-1.01	-0.6	-1.35	-1.75	0.96	0.99	0.82	0.74	13
-	F1SD73	PIG Uncharacterized protein NDUFB1	-1.11	-0.57	-1.51	-1.64	0.94	0.99	0.75	0.78	2
O75380	F1S031	PIG Uncharacterized protein NDUFS6	0.341	0.698	-0.93	-1.53	1.00	0.99	0.93	0.80	5
-	A1XQT2	PIG Cytochrome c oxidase subunit 6C	-1.81	-0.94	-1.58	-1.92	0.69	0.96	0.72	0.68	13
-	O43920	HUMAN NADH dehydrogenase [ubiquinone] iron-sulfur protein 5	-1.62	-0.99	-0.91	-1.12	0.77	0.94	0.94	0.88	1
-	Q007T0	PIG Succinate dehydrogenase [ubiquinone] iron-sulfur subunit, mitochondrial	-0.59	-0.36	-1.44	-2.01	0.98	1.01	0.78	0.66	12
-	P80021	PIG ATP synthase subunit alpha, mitochondrial	-0.41	-0.24	-1.76	-1.97	0.99	1.00	0.65	0.67	46
-	Q9MYT8	PIG ATP synthase subunit e, mitochondrial	-0.68	-0.52	-1.17	-1.59	0.98	1.00	0.88	0.80	6
-	F1SGC6	PIG Uncharacterized protein NDUFB5	-1.12	-0.79	-1.24	-1.46	0.93	0.98	0.86	0.82	8
-	P14854	HUMAN Cytochrome c oxidase subunit 6B1	-0.68	-0.06	-1.23	-1.69	0.98	1.00	0.86	0.76	1
Q99766	F1SHX8	PIG Uncharacterized protein (Fragment) ATP5S	-0.58	-0.87	-1.36	-1.26	0.98	0.97	0.82	0.85	4
-	F1S3W0	PIG Cytochrome b-c1 complex subunit 6	0.326	1.427	-0.82	-1.83	1.00	0.76	0.95	0.73	4
-	Q94PW4	PIG ATP synthase protein 8	-0.46	-0.11	-1.05	-1.59	0.99	1.00	0.91	0.80	1
-	Q29259	PIG NADH dehydrogenase [ubiquinone] 1 beta subcomplex subunit 6	-0.79	-0.43	-1	-1.56	0.96	1.01	0.92	0.80	3
O95168	I3LPW0	PIG Uncharacterized protein NDUFB4	-0.25	-0.37	-1.59	-1.8	0.99	1.01	0.72	0.73	6
Q9UI09	F1SQP4	PIG Uncharacterized protein (Fragment) NDUFA12	-0.9	-1.14	-1.26	-1.16	0.96	0.89	0.85	0.87	9
-	K7GLT8	PIG ATP synthase subunit beta	-0.97	-0.53	-1.54	-1.51	0.96	1.00	0.73	0.81	3
P28331	F1SHD7	PIG Uncharacterized protein (Fragment) NDUFS1	-0.39	-0.31	-1.17	-1.49	0.99	1.00	0.88	0.81	33
-	P22695	HUMAN Cytochrome b-c1 complex subunit 2, mitochondrial	-0.79	-0.11	-1.14	-1.92	0.96	1.00	0.90	0.69	2

-	F1RRC9	PIG Uncharacterized protein NDUFC1	-1.17	-0.92	-0.94	-1.19	0.92	0.96	0.93	0.87	1
P24539	F1SBN7	PIG Uncharacterized protein ATP5F1	-1.05	-0.51	-1.53	-1.64	0.95	1.00	0.74	0.78	15
-	I3LDC3	PIG Uncharacterized protein NDUFB10	0.132	0.083	-0.98	-1.62	0.99	1.00	0.92	0.79	8
-	F1RWV4	PIG Uncharacterized protein NDUFB11	-0.64	-0.4	-0.97	-1.75	0.98	1.00	0.93	0.74	5
P19404	F1SM98	PIG Uncharacterized protein NDUFV2	0.433	0.377	-0.88	-1.57	0.99	1.01	0.94	0.80	12
Q9Y6M9	F1RRP9	PIG Uncharacterized protein NDUFB9	-0.91	-0.56	-1.25	-1.54	0.96	0.99	0.85	0.80	6
-	P36542	HUMAN ATP synthase subunit gamma, mitochondrial	-0.3	-0.45	-1.4	-1.58	1.00	1.00	0.80	0.80	2
-	Q95283	PIG Cytochrome c oxidase subunit 4 isoform 1, mitochondrial (Fragment)	-1.02	-0.48	-1.14	-1.63	0.95	1.00	0.90	0.78	10
-	Q0QEM6	PIG ATP synthase subunit beta (Fragment)	0.153	0.159	-1.28	-1.95	0.99	1.00	0.85	0.67	37
-	D0VWV4	PIG Succinate dehydrogenase cytochrome b560 subunit, mitochondrial	0.037	-0.48	-1.54	-1.99	1.00	1.00	0.73	0.67	1
-	I3LRR4	PIG Uncharacterized protein (Fragment) NDUFV3	-0.82	-0.19	-0.81	-0.92	0.96	1.00	0.96	0.92	14
-	I3LTV6	PIG Cytochrome c oxidase subunit 6A, mitochondrial	0.416	0.014	1.396	-2.11	0.99	1.00	0.80	0.60	1
-	B2R4A2	HUMAN Cytochrome b-c1 complex subunit 7	-0.31	-0.06	-1.17	-1.23	1.00	1.00	0.88	0.86	2
-	G8JE99	HUMAN Cytochrome c oxidase subunit 2	-0.1	0.188	-0.96	-1.27	1.00	1.00	0.93	0.85	1
O75439	F1SKM0	PIG Uncharacterized protein UQCRC1	-0.51	-0.18	-0.89	-1.42	0.99	1.00	0.94	0.82	22
-	Q8SPJ9	PIG Cytochrome c oxidase subunit 7A1, mitochondrial	-0.31	-0.44	-1.38	-1.93	1.00	1.00	0.80	0.68	2
-	P28331	HUMAN NADH-ubiquinone oxidoreductase 75 kDa subunit, mitochondrial	-0.44	-0.21	-0.48	-0.2	0.99	1.00	0.99	0.99	1
O75306	F1S1A8	PIG Uncharacterized protein NDUFS2	0.646	0.119	-0.91	-1.43	0.98	1.00	0.94	0.82	16
-	Q0QF01	PIG Succinate dehydrogenase [ubiquinone] flavoprotein subunit, mitochondrial	-0.63	-0.52	-1.17	-1.28	0.98	1.00	0.88	0.85	22
O95178	F1SRQ0	PIG Uncharacterized protein (Fragment) NDUFB2	-0.05	-0.5	-1.11	-1.42	1.00	1.00	0.90	0.82	1
-	P56556	HUMAN NADH dehydrogenase [ubiquinone] 1 alpha subcomplex subunit 6	-0.32	-0.71	-0.77	-0.77	1.00	0.99	0.97	0.95	1
-	Q95339	PIG ATP synthase subunit f, mitochondrial	0.032	0.193	-0.96	-1.7	1.00	1.00	0.93	0.75	2
-	A5GZW8	PIG Succinate dehydrogenase [ubiquinone] cytochrome b small subunit	-0.67	-0.77	-0.94	-1.02	0.98	0.99	0.93	0.91	3
-	Q29235	PIG ATP synthase beta chain, mitochondrial (Fragment)	1.094	0.679	-0.94	-1.34	0.94	0.99	0.93	0.83	1
-	O75489	HUMAN NADH dehydrogenase [ubiquinone] iron-sulfur protein 3, mitochondrial	0.019	-0.64	-0.76	-1.05	1.00	0.99	0.97	0.91	1
Q9P0J0	F1S6Q1	PIG Uncharacterized protein NDUFA13	-0.18	-0.15	-0.95	-1.3	0.99	1.00	0.93	0.84	11
-	P24539	HUMAN ATP synthase F(0) complex subunit B1, mitochondrial	-0.46	-0.42	-0.24	-0.02	0.99	1.01	1.00	1.00	1

-	I3LGM4	PIG Uncharacterized protein NDUFA11	0.178	-0.23	-0.95	-1.4	0.99	1.00	0.93	0.82	2
-	F1S4V0	PIG Uncharacterized protein (Fragment) COX7A2L	-0.41	-0.37	-0.47	-0.6	0.99	1.01	0.99	0.97	2
-	P13073	HUMAN Cytochrome c oxidase subunit 4 isoform 1, mitochondrial	-0.31	-0.68	-0.17	0.525	1.00	0.99	1.00	0.98	1
-	P50667	PIG Cytochrome c oxidase subunit 2	0.393	0.566	-0.87	-1.45	0.99	0.99	0.94	0.82	11
-	P36542-2	HUMAN Isoform Heart of ATP synthase subunit gamma, mitochondrial	0.781	0.799	-0.88	-0.9	0.97	0.99	0.94	0.92	1
-	O79876	PIG Cytochrome c oxidase subunit 1	1.18	-0.28	-0.58	-0.77	0.92	1.00	0.98	0.95	2
-	P25705	HUMAN ATP synthase subunit alpha, mitochondrial	1.071	0.642	-0.34	-0.79	0.94	0.99	0.99	0.95	1
-	F1SI50	PIG Uncharacterized protein NDUFB3	-0	0.426	-0.68	-1.29	1.00	1.01	0.97	0.84	3
-	Q35915	PIG ATP synthase subunit a	-0.58	0.287	-0.45	-0.54	0.98	1.00	0.99	0.97	1
P17568	F1SCH1	PIG Uncharacterized protein (Fragment) NDUFB7	0.476	0.445	-0.49	-0.21	0.99	1.00	0.99	0.99	3
-	Q69GF7	PIG Cytochrome c oxidase subunit 2	0.254	0.909	0.034	-1.24	0.99	0.96	1.00	0.85	1
-	F1RJI7	PIG Cytochrome c oxidase subunit 6A, mitochondrial	0.666	0.227	0.262	0.023	0.98	1.00	1.00	1.00	1
-	Q2EN79	PIG Ubiquinol-cytochrome c reductase complex	0.736	0.193	-0.22	-0.87	0.97	1.00	1.00	0.93	2
-	H0YI37	HUMAN ATP synthase subunit beta, mitochondrial (Fragment)	0.52	0.072	0.166	-0.07	0.99	1.00	1.00	1.00	1
-	D6R613	HUMAN ATP synthase protein 8	1.819	0.197	-0.01	0.128	0.69	1.00	1.00	1.00	1
-	F1RQI1	PIG Uncharacterized protein COX7A2	0.61	0.48	0.584	0.123	0.99	1.00	0.98	1.00	2
-	Q9UII2	HUMAN ATPase inhibitor, mitochondrial	6.597	3.521	-1.98	-2.31	0.00	0.02	0.53	0.53	1
-	O95167	HUMAN NADH dehydrogenase [ubiquinone] 1 alpha subcomplex subunit 3	0.252	-0.05	0.916	1.396	0.99	1.00	0.93	0.82	1
-	F8W0P7	HUMAN ATP synthase subunit beta, mitochondrial (Fragment)	0.75	0.431	0.347	0.55	0.97	1.01	0.99	0.97	1
-	I3LS54	PIG Succinate dehydrogenase [ubiquinone] flavoprotein subunit, mitochondrial	1.095	2.439	-0.22	-0.34	0.94	0.24	1.00	0.99	1
-	F1SRG2	PIG NADH dehydrogenase [ubiquinone] 1 alpha subcomplex subunit 6	0.602	2.52	0.506	1.265	0.99	0.22	0.99	0.85	1

Human equivalent Ac. No.	Accession No.	TCA cycle	Zq (log2 FC)				FDR				No. peptides
		**Protein**	**120**	**1**	**4**	**7**	**120**	**1**	**4**	**7**	
-	P11708	PIG Malate dehydrogenase, cytoplasmic	-3.11	-4.13	-2.89	-2.18	0.10	0.00	0.15	0.60	20
-	P33198	PIG Isocitrate dehydrogenase [NADP], mitochondrial (Fragment)	-1.49	-2.81	-3.18	-2.57	0.83	0.12	0.08	0.44	29

-	P16276	PIG Aconitate hydratase, mitochondrial	-1.75	-2.2	-2.63	-2.42	0.72	0.35	0.23	0.50	41
-	P09622	HUMAN Dihydrolipoyl dehydrogenase, mitochondrial	-3.46	-3.34	-2.4	-2.08	0.04	0.03	0.32	0.63	1
-	P00346	PIG Malate dehydrogenase, mitochondrial	-1.38	-2.24	-2.86	-2.38	0.88	0.33	0.16	0.51	18
-	P56471	PIG Isocitrate dehydrogenase [NAD] subunit alpha, mitochondrial (Fragments)	-0.72	-2.11	-2.65	-2.19	0.97	0.39	0.23	0.58	3
-	F1RKU0	PIG Isocitrate dehydrogenase [NAD] subunit alpha, mitochondrial	-1.24	-2.49	-2.43	-2.17	0.91	0.23	0.31	0.59	12
-	O19069	PIG Succinyl-CoA ligase [ADP/GDP-forming] subunit alpha, mitochondrial	-1.71	-2.54	-2.2	-1.56	0.74	0.21	0.43	0.80	11
-	O97580	PIG Succinyl-CoA ligase [ADP-forming] subunit beta, mitochondrial (Fragment)	-1.33	-2.45	-2.33	-1.98	0.90	0.24	0.36	0.67	16
-	P00889	PIG Citrate synthase, mitochondrial	-1.93	-1.11	-2.39	-2.18	0.62	0.90	0.32	0.59	16
P11177	F1SGH5	PIG Uncharacterized protein PDHB	-1.21	-2.26	-2.36	-2.21	0.92	0.32	0.34	0.57	17
-	P53590	PIG Succinyl-CoA ligase [GDP-forming] subunit beta, mitochondrial (Fragment)	-0.91	-1.89	-2.13	-1.77	0.96	0.51	0.46	0.74	21
-	I3LPP1	PIG Fumarate hydratase, mitochondrial	-0.89	-0.92	-1.93	-2.12	0.96	0.96	0.55	0.59	2
-	Q9N0F1	PIG Dihydrolipoyllysine-residue succinyltransferase	-0.93	-0.5	-1.65	-1.93	0.96	1.00	0.69	0.69	16
-	F1RK10	PIG Succinyl-CoA ligase [ADP-forming] subunit beta, mitochondrial	0.153	-2.13	-2.23	-2.03	0.99	0.38	0.41	0.65	2
-	P10173	PIG Fumarate hydratase, mitochondrial	-1.25	-0.76	-1.66	-1.74	0.91	0.99	0.69	0.75	27
-	Q1G1K7	PIG Mitochondrial NAD+isocitrate dehydrogenase 3 beta variant 2	-0.8	-1.79	-1.86	-1.61	0.96	0.57	0.59	0.80	18
-	P09623	PIG Dihydrolipoyl dehydrogenase, mitochondrial	-0.73	-0.46	-1.64	-1.79	0.97	1.00	0.70	0.74	15
-	P11177	HUMAN Pyruvate dehydrogenase E1 component subunit beta, mitochondrial	-1.66	-1.82	-1.46	-1.15	0.76	0.55	0.76	0.87	1
Q9NX18	F1RKQ9	PIG Uncharacterized protein LOC100519294	-1.2	-1.16	-1.07	-0.92	0.92	0.89	0.91	0.92	3
-	P48735	HUMAN Isocitrate dehydrogenase [NADP], mitochondrial	0.032	-0.89	-1.5	-1.28	1.00	0.97	0.75	0.85	2
-	Q007T0	PIG Succinate dehydrogenase [ubiquinone] iron-sulfur subunit, mitochondrial	-0.59	-0.36	-1.44	-2.01	0.98	1.01	0.78	0.66	12
-	K9IVI1	PIG 2-oxoglutarate dehydrogenase, mitochondrial	-0.81	-0.68	-1.26	-1.29	0.96	0.99	0.85	0.84	39
-	F1S297	PIG Isocitrate dehydrogenase [NAD] subunit gamma, mitochondrial	-0.05	-1.14	-1.06	-0.93	1.00	0.90	0.91	0.92	9
-	D0VWV4	PIG Succinate dehydrogenase cytochrome b560 subunit, mitochondrial	0.037	-0.48	-1.54	-1.99	1.00	1.00	0.73	0.67	1
-	C6KE31	PIG Transporter	-0.06	-0.32	-0.97	-1.02	1.00	1.00	0.93	0.91	1
-	P42174	PIG Glutamate dehydrogenase 1, mitochondrial (Fragments)	-0.76	-0.83	-0.52	-0.05	0.97	0.98	0.99	1.00	1
-	Q0QF01	PIG Succinate dehydrogenase [ubiquinone] flavoprotein subunit, mitochondrial	-0.63	-0.52	-1.17	-1.28	0.98	1.00	0.88	0.85	22
-	H0YIC4	HUMAN Citrate synthase (Fragment)	0.299	0.577	-0.58	-1.59	1.00	0.99	0.98	0.80	1

Human equivalent Ac. No.	Accession No.	Protein	Zq (log2 FC) 120	1	4	7	FDR 120	1	4	7	No. peptides
-	A5GZW8	PIG Succinate dehydrogenase [ubiquinone] cytochrome b small subunit	-0.67	-0.77	-0.94	-1.02	0.98	0.99	0.93	0.91	3
-	P40926	HUMAN Malate dehydrogenase, mitochondrial	0.246	-0.01	-0.94	-0.93	0.99	1.00	0.93	0.92	2
P35232	F1RWF5	PIG Uncharacterized protein	-0.13	0.27	-0.82	-1.03	0.99	1.00	0.96	0.91	12
-	O43837	HUMAN Isocitrate dehydrogenase [NAD] subunit beta, mitochondrial	0.84	0.458	-0.66	-1.02	0.96	1.00	0.98	0.91	1
-	F1SEN2	PIG Glutamate dehydrogenase 1, mitochondrial	-0.05	-0.08	-0.17	-0.18	1.00	1.00	1.00	0.99	19
-	I3LDC7	PIG Isocitrate dehydrogenase [NADP] (Fragment)	0.041	0.329	0.062	0.279	1.00	1.01	1.00	0.99	11
-	F1SAF0	PIG Dihydrolipoyl dehydrogenase	0.96	1.192	0.15	-0.43	0.95	0.88	1.00	0.99	1
O14950	I3LBF7	PIG Uncharacterized protein (Fragment) LOC733637	0.699	1.052	1.506	1.795	0.98	0.93	0.75	0.73	2

Fatty acid beta-oxidation

Human equivalent Ac. No.	Accession No.	Protein	Zq (log2 FC) 120	1	4	7	FDR 120	1	4	7	No. peptides
-	P24752	HUMAN Acetyl-CoA acetyltransferase, mitochondrial	-4.03	-3.15	-3.37	-2.75	0.01	0.06	0.05	0.38	1
P30084	F1SAC1	PIG Uncharacterized protein ECHS1	-2.33	-3.15	-2.66	-2.26	0.40	0.06	0.22	0.54	14
-	P00348	PIG Hydroxyacyl-coenzyme A dehydrogenase, mitochondrial	-1.73	-1.89	-2.59	-2.17	0.73	0.52	0.24	0.59	24
-	D0G0B3	PIG Acetyl-Coenzyme A acyltransferase 2	-1.19	-1.6	-2.2	-2.25	0.93	0.67	0.43	0.54	19
-	P40939	HUMAN Trifunctional enzyme subunit alpha, mitochondrial	-1.11	-2.92	-2.09	-2.92	0.93	0.09	0.48	0.30	2
-	K7GM59	PIG Uncharacterized protein (Fragment) ABCD1	-2.35	-2.22	-1.35	-0.71	0.39	0.35	0.82	0.96	1
-	P42765	HUMAN 3-ketoacyl-CoA thiolase, mitochondrial	-1.07	-1.13	-2.22	-2.58	0.94	0.90	0.42	0.43	2
P24752	I3LP02	PIG Uncharacterized protein ACAT1	-2.02	-1.53	-2.09	-1.88	0.58	0.71	0.47	0.70	20
-	P79273	PIG Short-chain specific acyl-CoA dehydrogenase, mitochondrial	-1.12	-1.93	-1.8	-1.67	0.93	0.49	0.63	0.77	10
Q99714	K7GND4	PIG Uncharacterized protein (Fragment) HSD17B10	-1.1	-1.53	-1.69	-1.62	0.94	0.71	0.68	0.79	3
P45954	F1SED0	PIG Uncharacterized protein (Fragment) ACADSB	-1.24	-2.15	-1.95	-1.67	0.91	0.38	0.54	0.77	12
P49748	I3LLE3	PIG Uncharacterized protein (Fragment) LOC100520636	-1.03	-1.84	-1.91	-1.72	0.95	0.54	0.56	0.75	27
Q13011	I3LJJ4	PIG Uncharacterized protein ECH1	-0.8	-0.39	-1.32	-1.61	0.96	1.00	0.83	0.79	6
P55084	F1SDN2	PIG Uncharacterized protein HADHB	-0.82	-1.21	-1.63	-1.47	0.96	0.87	0.70	0.81	33
-	I3LAI1	PIG Uncharacterized protein (Fragment) ECHS1	-0.63	-1.35	-1.44	-1.55	0.98	0.81	0.78	0.80	1

-	K7GRM6	PIG Uncharacterized protein (Fragment) HSD17B10	-0.16	-1.27	-1.57	-1.38	0.99	0.85	0.72	0.83	1
-	Q29554	PIG Trifunctional enzyme subunit alpha, mitochondrial	-0.71	-1.21	-1.84	-1.45	0.98	0.87	0.60	0.82	41
-	P79274	PIG Long-chain specific acyl-CoA dehydrogenase, mitochondrial	-0.41	-0.57	-1.39	-1.45	0.99	0.99	0.80	0.82	22
Q6JQN1	I3LK34	PIG Uncharacterized protein LOC100625244	-0.02	-0.06	-0.98	-1.1	1.00	1.00	0.93	0.89	4
-	P41367	PIG Medium-chain specific acyl-CoA dehydrogenase, mitochondrial	-0.03	-0.65	-1.59	-1.27	1.00	0.99	0.71	0.85	15
-	Q3Y5G5	PIG Peroxisomal enoyl coenzyme A hydratase 1	-0.59	-0.24	-0.71	-0.9	0.99	1.00	0.97	0.93	6
-	Q8HY46	PIG Carnitine O-palmitoyltransferase 1, muscle isoform	0.05	-1.04	-1.23	-1.46	1.00	0.93	0.86	0.82	18
P23786	F1S764	PIG Uncharacterized protein CPT2	-0.01	-0.83	-0.94	-0.83	1.00	0.98	0.93	0.94	9
-	P55931	PIG Electron transfer flavoprotein-ubiquinone oxidoreductase, mitochondrial	-0.54	-0.41	-0.96	-1.21	0.99	1.01	0.93	0.87	19
-	B2ZF49	PIG Hydroxyacyl-coenzyme A dehydrogenase	0.22	-0.94	-1.26	-1.92	0.99	0.95	0.85	0.68	1
-	A7YB26	PIG Peroxisomal enoyl coenzyme A hydratase 1 (Fragment)	0.658	0.008	-0.78	-0.92	0.98	1.00	0.96	0.92	1
-	Q16836	HUMAN Hydroxyacyl-coenzyme A dehydrogenase, mitochondrial	0.489	-0.35	-0.87	-1.17	0.98	1.01	0.94	0.87	1
Q9H845	I3LUR5	PIG Uncharacterized protein (Fragment) ACAD9	0.224	-0.61	-1.02	-0.85	0.99	0.99	0.92	0.94	7
Q6JQN1	I3LDT4	PIG Uncharacterized protein	-0.14	-0.36	-0.01	-0.34	0.99	1.01	1.00	0.99	2
-	F1RL48	PIG Uncharacterized protein ACAD10	0.835	0.675	0.211	-0.88	0.96	0.99	1.00	0.93	1
-	F1RUP0	PIG Uncharacterized protein (Fragment) ECHDC3	0.165	-0.79	0.041	-0.37	0.99	0.98	1.00	0.99	1
Q99714	K7GMY3	PIG Uncharacterized protein (Fragment) HSD17B10	0.159	-0.64	-0.41	-0.12	0.99	0.99	1.00	1.00	3
-	Q28956	PIG 17beta-estradiol dehydrogenase	0.459	-0.07	0.136	-0.06	0.99	1.00	1.00	1.00	6
P51659	I3LEF8	PIG Uncharacterized protein HSD17B4	0.632	-0.02	-0.06	0.015	0.98	1.00	1.00	1.00	9
-	I3LJ48	PIG Uncharacterized protein (Fragment) EHHADH	0.38	0.745	0.319	-0.18	0.99	0.99	1.00	0.99	1
-	P23786	HUMAN Carnitine O-palmitoyltransferase 2, mitochondrial	0.525	0.869	0.213	-0.1	0.99	0.97	1.00	1.00	1
-	P55084	HUMAN Trifunctional enzyme subunit beta, mitochondrial	1.044	0.97	0.559	-0.02	0.95	0.94	0.98	1.00	1

Muscle contraction

**Human equivalent Ac. No.**	**Accession No.**	**Protein**	**Zq (log2 FC)**				**FDR**				**No. peptides**
			**120**	**1**	**4**	**7**	**120**	**1**	**4**	**7**	
-	F2Z5B6	PIG Tropomyosin alpha-1 chain	-2.26	-0.07	-2.84	-3.22	0.44	1.00	0.17	0.21	5

-	Q6ZRF8	HUMAN RING finger protein 207	-1.93	-2.57	-3.3	-2.5	0.62	0.19	0.06	0.46	1
P45379	I3LS66	PIG Uncharacterized protein TNNT2	-1.73	-0.96	-2.62	-2.76	0.73	0.94	0.23	0.38	23
-	D2SQP3	PIG Cysteine and glycine-rich protein 3	-3.27	-3.44	-2.14	-1.18	0.07	0.03	0.45	0.87	5
P12814	F1RHL9	PIG Uncharacterized protein ACTN2	-2.02	-3.39	-2.64	-2.17	0.57	0.03	0.23	0.59	55
-	P26678	HUMAN Cardiac phospholamban	-2.44	-2.49	-2.21	-1.44	0.35	0.22	0.43	0.82	1
Q8N335	I3LLU0	PIG Uncharacterized protein GPD1L	-2.49	-2.77	-2.24	-1.71	0.33	0.13	0.41	0.75	15
-	P07951	HUMAN Tropomyosin beta chain	-0.59	0.06	-2.64	-2.81	0.99	1.00	0.23	0.36	2
Q14324	F1SID7	PIG Uncharacterized protein MYBPC3	-2.79	-3.8	-2.37	-1.93	0.19	0.01	0.33	0.68	71
-	A4GR69	PIG Telethonin	-1.93	-2.19	-1.89	-1.8	0.62	0.36	0.57	0.74	6
-	Q99959	HUMAN Plakophilin-2	-1.99	-3.02	-1.98	-1.43	0.58	0.08	0.53	0.82	1
-	F1RIN7	PIG Uncharacterized protein HRC	-0.67	-0.42	-1.91	-1.66	0.98	1.01	0.56	0.78	10
-	P11607	PIG Sarcoplasmic/endoplasmic reticulum calcium ATPase 2	-1.51	-2.3	-1.98	-1.83	0.83	0.30	0.53	0.72	46
-	F1SNW4	PIG MYL3	-0.24	0.62	-1.84	-2.5	0.99	0.99	0.60	0.46	17
-	P04075	HUMAN Fructose-bisphosphate aldolase A	-1.91	-2.87	-1.65	-1.09	0.62	0.11	0.69	0.89	16
-	F1SAW8	PIG Calsequestrin (Fragment)	-1.32	-0.75	-1.71	-1.97	0.90	0.99	0.67	0.67	6
-	P67936-2	HUMAN Isoform 2 of Tropomyosin alpha-4 chain	-1.32	-0.3	-1.4	-1.91	0.90	1.00	0.80	0.69	1
Q9UI47	F1SUJ6	PIG Uncharacterized protein CTNNA3	-1.25	-1.75	-1.92	-1.55	0.91	0.59	0.55	0.80	1
-	Q03135	HUMAN Caveolin-1	-0.66	-1.44	-1.89	-1.92	0.98	0.75	0.57	0.69	1
-	Q14896	HUMAN Myosin-binding protein C, cardiac-type	-2.2	-1.23	-0.95	-1.01	0.46	0.86	0.93	0.91	1
P15924	F1RW75	PIG Uncharacterized protein DSP	-1.55	-2.08	-1.37	-1.1	0.81	0.40	0.81	0.89	75
O75380	F1S031	PIG Uncharacterized protein NDUFS6	0.341	0.698	-0.93	-1.53	1.00	0.99	0.93	0.80	5
-	Q14BN4-8	HUMAN Isoform 8 of Sarcolemmal membrane-associated protein	-1.53	-1.34	-1.2	-0.94	0.82	0.81	0.87	0.92	2
-	I3LVH8	PIG Uncharacterized protein (Fragment) DSC2	-1.15	-1.06	-1.18	-1.15	0.93	0.92	0.88	0.87	3
-	P04178	PIG Superoxide dismutase [Cu-Zn]	-1.37	-0.64	-0.45	-0.25	0.88	0.99	0.99	0.99	5
-	P54296	HUMAN Myomesin-2	-1.34	-1.73	-1.41	-1.5	0.89	0.61	0.80	0.81	6
-	A1XQT6	PIG MLC1f	0.263	-1.54	-1.4	-1.83	0.99	0.71	0.80	0.73	13

Q99959	F1SGD2	PIG Uncharacterized protein PKP2	-1.03	-1.36	-1.1	-0.97	0.95	0.80	0.91	0.92	18
-	O14958	HUMAN Calsequestrin-2	-1.11	-0.53	-1.57	-1.85	0.94	1.00	0.72	0.72	1
Q13424	F1S4Z9	PIG Uncharacterized protein SNTA1	-0.85	-1.48	-1.3	-1.34	0.96	0.73	0.84	0.83	7
P52179	F1SM75	PIG Uncharacterized protein MYOM1	-1.4	-1.97	-1.42	-1.38	0.87	0.47	0.78	0.83	86
-	Q96MG2	HUMAN Junctional sarcoplasmic reticulum protein 1	-0.72	-0.84	-0.9	-0.57	0.97	0.98	0.94	0.97	1
-	Q9UI47	HUMAN Catenin alpha-3	-0.93	-0.81	-0.63	-0.7	0.96	0.98	0.98	0.96	3
P28289	I3LJ88	PIG Uncharacterized protein TMOD1	-0.56	-0.7	-1	-1.21	0.98	0.99	0.92	0.87	19
-	P16960	PIG Ryanodine receptor 1	-0.85	-1.02	-0.7	-0.83	0.96	0.93	0.97	0.94	2
-	Q00872	HUMAN Myosin-binding protein C, slow-type	-1.29	-1.72	-1.15	-0.67	0.91	0.61	0.89	0.96	2
-	P01160	HUMAN Natriuretic peptides A	-1.37	-1.54	-0.79	-0.51	0.88	0.71	0.96	0.98	1
P21817	F1RHM3	PIG Uncharacterized protein (Fragment) RYR2	-0.44	-0.58	-0.94	-0.9	0.99	0.99	0.93	0.92	21
-	Q14BN4	HUMAN Sarcolemmal membrane-associated protein	-1.06	-0.45	-0.72	-0.92	0.95	1.00	0.97	0.92	19
-	P08590	HUMAN Myosin light chain 3	1.056	1.008	-1.09	-1.45	0.95	0.94	0.91	0.82	1
-	P32418	HUMAN Sodium/calcium exchanger 1	-0.71	-0.86	-0.94	-0.68	0.98	0.97	0.93	0.96	11
-	Q9UKX3	HUMAN Myosin-13	-0.84	-0.72	-0.91	-1.07	0.96	0.99	0.94	0.90	5
-	Q9BX66	HUMAN Sorbin and SH3 domain-containing protein 1	-0.54	-1.25	-0.37	0.032	0.98	0.85	1.00	1.00	1
Q8WZ42	I3L8Q0	PIG Uncharacterized protein	-1.43	-1.76	-1	-0.75	0.86	0.58	0.92	0.96	87
-	Q8WZ42	HUMAN Titin	-1.29	-1.81	-1.07	-0.84	0.91	0.56	0.91	0.94	575
-	P12883	HUMAN Myosin-7	-0.64	-0.46	-0.36	-0.66	0.98	1.00	1.00	0.96	4
-	Q9BUA6	HUMAN Myosin regulatory light chain 10	0.621	0.212	-0.62	-0.95	0.99	1.00	0.98	0.92	1
Q86TC9	I3LIB5	PIG Uncharacterized protein MYPN	-0.01	-0.83	-0.79	-0.25	1.00	0.98	0.96	0.99	3
-	O95180	HUMAN Voltage-dependent T-type calcium channel subunit alpha-1H	-0.82	-1.81	-0.83	-0.89	0.96	0.56	0.96	0.93	1
-	P15924	HUMAN Desmoplakin	1.216	-0.12	-0.84	-0.82	0.92	1.00	0.95	0.94	2
-	Q59H18	HUMAN Serine/threonine-protein kinase TNNI3K	-0.3	-0.38	-0.52	0.263	1.00	1.01	0.99	0.99	1
Q01484	F1S146	PIG Uncharacterized protein ANK2	-0.54	-0.5	-0.24	-0.45	0.98	1.00	1.00	0.99	6
P12829	F1RRT2	PIG Uncharacterized protein (Fragment) MYL4	-0.14	0.267	-0.89	-1.08	0.99	1.00	0.94	0.89	8
Q6P5Q4	F1SLY4	PIG Uncharacterized protein LMOD2	0.426	0.1	-0.36	-0.61	0.99	1.00	0.99	0.97	7

-	Q01449	HUMAN Myosin regulatory light chain 2, atrial isoform	-0.03	-0.57	-0.49	-0.9	1.00	0.99	0.99	0.93	1
-	P11055	HUMAN Myosin-3	0.637	-0.16	-0.7	-0.7	0.98	1.00	0.97	0.96	5
-	Q86U42	HUMAN Polyadenylate-binding protein 2	-0.46	-0.55	-0.2	0.356	0.99	0.99	1.00	0.99	2
-	Q9UKX2	HUMAN Myosin-2	0.286	0.479	-0.35	-0.6	1.00	1.00	1.00	0.97	1
-	P09493-6	HUMAN Isoform 6 of Tropomyosin alpha-1 chain	-0.55	-0.11	-0.37	0.668	0.98	1.00	0.99	0.96	4
-	P13535	HUMAN Myosin-8	1.309	1.185	0.196	-0.67	0.90	0.88	1.00	0.96	5
-	F1S9D6	PIG Myosin-7	2.4	0.366	-1.01	-0.95	0.36	1.01	0.92	0.92	2
Q8WUF5	I3L801	PIG Uncharacterized protein (Fragment) PPP1R13L	0.789	-0.09	-0.4	-0.75	0.96	1.00	0.99	0.96	1
-	Q14126	HUMAN Desmoglein-2	-1.21	-0.93	0.541	1.265	0.92	0.96	0.98	0.85	1
-	P17661	HUMAN Desmin	-0.05	-0.34	0.693	0.35	1.00	1.01	0.97	0.99	1
-	P50402	HUMAN Emerin	-0.05	0.447	0.439	0.354	1.00	1.00	0.99	0.99	2
-	P35749	HUMAN Myosin-11	-0.75	-0.52	-0.19	0.7	0.97	1.00	1.00	0.96	6
-	P20929	HUMAN Nebulin	-0.79	-0.06	0.206	0.434	0.96	1.00	1.00	0.99	3
-	P29536	HUMAN Leiomodin-1	1.404	1.552	-0.28	-0.09	0.87	0.70	1.00	1.00	1
-	O00168	HUMAN Phospholemman	0.735	0.899	-0.2	-0.29	0.97	0.97	1.00	0.99	1
-	P13533	HUMAN Myosin-6	1.199	-0.79	-0.69	-1.17	0.93	0.98	0.97	0.87	8
-	P08133	HUMAN Annexin A6	0.779	0.511	0.159	-0.57	0.97	1.00	1.00	0.97	1
-	F1RIM2	PIG Uncharacterized protein RNF207	-0.12	0.612	0.239	-0.27	0.99	0.99	1.00	0.99	1
Q05682	I3L7S4	PIG Uncharacterized protein CALD1	-0.35	-0.06	0.877	2.315	1.00	1.00	0.94	0.53	28
-	Q9N0Y9	PIG Ubiquitous tropomodulin U-Tmod	0.151	0.584	0.426	0.471	0.99	0.99	1.00	0.98	10
-	Q9Y623	HUMAN Myosin-4	0.8	0.771	-0.22	-0.43	0.96	0.98	1.00	0.99	4
-	Q9NZR1	HUMAN Tropomodulin-2	0.407	0.508	0.287	-0.34	0.99	1.00	1.00	0.99	1
-	P09493-2	HUMAN Isoform 2 of Tropomyosin alpha-1 chain	0.357	0.587	0.5	1.27	1.00	0.99	0.99	0.85	3
-	Q05682-2	HUMAN Isoform 2 of Caldesmon	1.392	0.786	0.447	0.017	0.87	0.98	0.99	1.00	1
-	F1S0V3	PIG Annexin (Fragment)	0.339	0.395	0.242	0.585	1.00	1.00	1.00	0.97	31
-	Q14247	HUMAN Src substrate cortactin	0.674	0.544	0.89	0.95	0.98	0.99	0.94	0.92	3
-	Q13698	HUMAN Voltage-dependent L-type calcium channel subunit alpha-1S	-0.51	-0.34	0.988	2.721	0.99	1.01	0.92	0.38	1

-	Q9Y490	HUMAN Talin-1	0.357	0.423	0.962	0.789	1.00	1.01	0.93	0.95	1
-	I3L650	PIG Uncharacterized protein CALD1	1.553	0.941	-0.05	0.269	0.81	0.95	1.00	0.99	1
-	O60662	HUMAN Kelch-like protein 41	0.918	0.59	1.097	0.772	0.96	0.99	0.90	0.95	1
-	Q15746	HUMAN Myosin light chain kinase, smooth muscle	0.795	0.521	0.43	1.174	0.96	1.00	0.99	0.87	2
-	Q9UHP9	HUMAN Small muscular protein	1.57	0.699	0.702	0.563	0.79	0.99	0.97	0.97	1
-	P09493-5	HUMAN Isoform 5 of Tropomyosin alpha-1 chain	0.995	2.156	1.163	0.929	0.96	0.37	0.88	0.92	1
-	P06753-2	HUMAN Isoform 2 of Tropomyosin alpha-3 chain	-0.32	1.143	1.883	2.269	1.00	0.89	0.58	0.55	2
-	P67936	HUMAN Tropomyosin alpha-4 chain	0.268	0.854	0.852	2.109	0.99	0.97	0.95	0.60	7
-	P60660	HUMAN Myosin light polypeptide 6	0.605	1.409	1.727	2.636	0.99	0.78	0.66	0.41	8
-	P01042	HUMAN Kininogen-1	3.166	2.613	0.966	0.818	0.09	0.18	0.93	0.94	1
-	P08670	HUMAN Vimentin	1.094	1.74	2.384	3.078	0.94	0.60	0.33	0.24	1

Human equivalent Ac. No.	Accession No.	Fatty acid biosynthesis<br>Protein	Zq (log2 FC)<br>120	1	4	7	FDR<br>120	1	4	7	No. peptides
-	Q6B339	PIG Acyl coenzyme A synthetase long-chain 1 (Fragment)	-2.37	-2.5	-1.96	-1.89	0.38	0.22	0.54	0.70	22
-	O00763	HUMAN Acetyl-CoA carboxylase 2	-1.72	-1.76	-1.77	-1.11	0.74	0.58	0.65	0.88	2
-	Q4L235	HUMAN Acyl-CoA synthetase family member 4	-1.1	-2.15	-1.22	-0.85	0.94	0.38	0.86	0.94	1
-	Q4G176	HUMAN Acyl-CoA synthetase family member 3, mitochondrial	-0.66	-1.49	-1.36	-0.78	0.98	0.73	0.82	0.95	1
-	F1S3I9	PIG Uncharacterized protein ACOT4	-0.75	-0.56	-0.91	-0.33	0.97	0.99	0.94	0.99	1
-	D2D0E6	PIG Malonyl-CoA decarboxylase	-1.05	-0.74	-0.99	-1.24	0.95	0.99	0.92	0.86	2
Q4G176	I3LK72	PIG Uncharacterized protein (Fragment) ACSF3	-0.05	-0.7	-0.97	-0.82	1.00	0.99	0.93	0.95	5
Q9BV79	F1STL4	PIG Uncharacterized protein MECR	-0.52	-0.52	-0.55	-0.46	0.99	1.00	0.98	0.99	3
-	Q08AH1	HUMAN Acyl-coenzyme A synthetase ACSM1, mitochondrial	-0.44	-0.23	-0.6	-1.15	0.99	1.00	0.98	0.87	1
-	P09917	HUMAN Arachidonate 5-lipoxygenase	0.322	0.326	-0.37	-0.91	1.00	1.00	0.99	0.92	1
-	H7C2V0	HUMAN Fatty acid desaturase 1 (Fragment)	0.771	0.677	0.025	-0.62	0.97	0.99	1.00	0.96	2
-	O75342	HUMAN Arachidonate 12-lipoxygenase, 12R-type	-1.09	0.097	1.09	0.495	0.94	1.00	0.90	0.98	1

-	Q9XT00	PIG Estradiol 17-beta-dehydrogenase 8	1.351	0.468	-0.03	-0.81	0.89	1.00	1.00	0.95	2

Metabolism of amino acids

Human equivalent Ac. No.	Accession No.	Protein	Zq (log2 FC) 120	1	4	7	FDR 120	1	4	7	No. peptides
-	P00503	PIG Aspartate aminotransferase, cytoplasmic	-2.06	-3.05	-2.38	-1.86	0.55	0.07	0.33	0.71	24
-	P00506	PIG Aspartate aminotransferase, mitochondrial	-0.77	-0.77	-2.04	-2.28	0.97	0.98	0.50	0.54	26
-	Q96N76	HUMAN Urocanate hydratase	-2.12	-1.55	-1.92	-2.12	0.51	0.71	0.56	0.59	1
P51649	F1RUE3	PIG Uncharacterized protein ALDH5A1	-1.07	-1.58	-1.97	-1.73	0.95	0.69	0.53	0.75	8
-	P31641	HUMAN Sodium- and chloride-dependent taurine transporter	-1.88	-1.88	-1.35	-0.86	0.64	0.51	0.82	0.94	1
P31937	F1SHU0	PIG Uncharacterized protein (Fragment) LOC100516841	-0.21	-0.72	-1.32	-1.25	0.99	0.99	0.83	0.86	2
P31937	I3LUZ8	PIG Uncharacterized protein LOC100516656	-0.38	-0.73	-1.56	-1.45	0.99	0.99	0.73	0.82	3
-	B1PK12	PIG Branched chain keto acid dehydrogenase E1 alpha polypeptide	-0.09	-0.64	-1.3	-1.3	1.00	0.99	0.84	0.84	6
-	Q29582	PIG Pyruvate kinase M2 (Fragment)	-1.1	-2.04	-1.57	-0.98	0.94	0.43	0.72	0.92	1
-	P54886	HUMAN Delta-1-pyrroline-5-carboxylate synthase	-0.56	-0.66	-0.9	-1.39	0.98	0.99	0.94	0.83	1
-	Q28969	PIG Nitric oxide synthase, endothelial	-0.73	-0.82	-1.37	-1.07	0.97	0.98	0.81	0.90	1
-	F1SHL9	PIG Pyruvate kinase (Fragment)	-1.71	-2.45	-0.99	-0.3	0.74	0.24	0.92	0.99	31
-	O95865	HUMAN N(G),N(G)-dimethylarginine dimethylaminohydrolase 2	-0.14	-0.93	-1.06	-0.59	0.99	0.96	0.91	0.97	5
-	Q7YS30	PIG F1F0-ATP synthase complex g subunit-like protein (Fragment)	-0.81	-0.42	-1.01	-0.87	0.96	1.01	0.92	0.93	2
-	P42174	PIG Glutamate dehydrogenase 1, mitochondrial (Fragments)	-0.76	-0.83	-0.52	-0.05	0.97	0.98	0.99	1.00	1
-	P34896	HUMAN Serine hydroxymethyltransferase, cytosolic	-0.68	-0.83	-0.47	-0.17	0.98	0.98	0.99	1.00	1
-	Q29558	PIG NADP-dependent malic enzyme (Fragment)	0.02	-0.38	-0.7	-0.32	1.00	1.01	0.97	0.99	1
Q86WA6	F1RWY7	PIG Uncharacterized protein BPHL	0.251	-0.46	-0.78	-0.52	0.99	1.00	0.97	0.98	4
O94760	F1SEX0	PIG Uncharacterized protein (Fragment) DDAH1	-0.76	-0.27	0.321	0.011	0.97	1.00	1.00	1.00	4
Q9HCC0	I3LQU8	PIG Uncharacterized protein	-0.3	-0.18	-0.69	-0.41	1.00	1.00	0.97	0.99	3
-	F1SEN2	PIG Glutamate dehydrogenase 1, mitochondrial	-0.05	-0.08	-0.17	-0.18	1.00	1.00	1.00	0.99	19
-	P23434	HUMAN Glycine cleavage system H protein, mitochondrial	0.494	0.142	-0.55	-0.36	0.99	1.00	0.98	0.99	1

-	I3LKS6	PIG Dihydropteridine reductase	-0.11	-0.21	-0.03	0.667	1.00	1.00	1.00	0.96	1
-	P00505	HUMAN Aspartate aminotransferase, mitochondrial	0.939	0.272	-0.37	-0.43	0.96	1.00	1.00	0.99	1
-	F1RPN9	PIG Malic enzyme	0.25	0.153	-0.14	-0.16	0.99	1.00	1.00	1.00	3
-	F1SPI3	PIG Transporter (Fragment)	0.697	0.271	-0.42	-0.34	0.98	1.00	1.00	0.99	1
P48637	F1S4X9	PIG Uncharacterized protein GSS	0.518	-0.3	0.151	0.555	0.99	1.00	1.00	0.98	2
-	P31937	HUMAN 3-hydroxyisobutyrate dehydrogenase, mitochondrial	1.121	0.512	-0.05	0.007	0.93	1.00	1.00	1.00	2
-	Q8MJ30	PIG Dihydropteridine reductase	0.943	0.234	-0.03	-0.2	0.96	1.00	1.00	0.99	8
P12955	I3LR43	PIG Uncharacterized protein PEPD	0.926	-0.18	0.146	0.443	0.96	1.00	1.00	0.99	4
P12955	F1RNW4	PIG Uncharacterized protein LOC100739263	-0.58	-0.35	0.744	0.805	0.98	1.01	0.97	0.95	5
-	P14618	HUMAN Pyruvate kinase PKM	0.476	-0.11	0.24	0.775	0.99	1.00	1.00	0.95	2
-	Q9UBK8	HUMAN Methionine synthase reductase	0.675	0.887	0.805	0.52	0.98	0.97	0.96	0.98	1
-	P32322	HUMAN Pyrroline-5-carboxylate reductase 1, mitochondrial	0.366	0.201	0.766	1.649	1.00	1.00	0.97	0.78	2
Q00610	I3LGD4	PIG Uncharacterized protein	0.517	0.124	0.627	1.008	0.99	1.00	0.98	0.91	22
Q9HCC0	F1SK15	PIG Uncharacterized protein LOC100518224	1.27	0.579	0.927	-0.17	0.91	0.99	0.93	1.00	2
-	F1SM93	PIG 6-pyruvoyl tetrahydrobiopterin synthase	0.283	1.021	0.309	-0.41	0.99	0.93	1.00	0.99	1
-	Q95JC8	PIG Arginase-1	0.744	1.054	0.839	0.165	0.97	0.93	0.95	1.00	2
-	P12955	HUMAN Xaa-Pro dipeptidase	1.066	1.071	1.061	1.397	0.95	0.92	0.91	0.82	1
-	P50440	HUMAN Glycine amidinotransferase, mitochondrial	1.325	0.561	0.836	0.736	0.90	0.99	0.95	0.96	1
-	D0G0C6	PIG Asparagine synthetase	1.429	1.114	1.251	1.567	0.86	0.90	0.85	0.80	3

Human equivalent Ac. No.	Accession No.	Mitochondrial ribosome<br>Protein	Zq (log2 FC) 120	1	4	7	FDR 120	1	4	7	No. peptides
	F1RYS1	PIG Uncharacterized protein (Fragment) MRPL1	-2.20	-1.15	-2.09	-2.50	0.46	0.89	0.47	0.47	1
P82909	I3LQ99	PIG Uncharacterized protein MRPS36	-1.19	-0.35	-1.44	-1.79	0.93	1.01	0.78	0.74	5
	F1RK59	PIG Uncharacterized protein MRPL40	-0.35	-0.67	-1.56	-1.78	1.00	0.99	0.73	0.74	3
	F1S8G3	PIG Uncharacterized protein MRPS33	-0.86	-0.72	-1.08	-0.75	0.96	0.99	0.91	0.96	1

	P52815	HUMAN 39S ribosomal protein L12, mitochondrial	-0.26	0.27	-0.89	-0.96	0.99	1.00	0.94	0.92	4
Q8N983	F1S8U4	PIG Uncharacterized protein MRPL43	-0.20	-0.45	-0.91	-0.69	0.99	1.00	0.94	0.96	3
	F1S9B7	PIG Uncharacterized protein MRPL52	0.30	-0.30	-0.69	-0.70	1.00	1.00	0.97	0.96	1
	F1S8A5	PIG Uncharacterized protein MRPS26	0.54	0.28	-0.42	-0.20	0.99	1.00	1.00	0.99	3
	Q9Y2Q9	HUMAN 28S ribosomal protein S28, mitochondrial	0.71	0.64	0.11	-0.12	0.97	0.99	1.00	1.00	1
	F1SG95	PIG Uncharacterized protein MRPS35	0.52	-0.06	-0.13	0.14	0.99	1.00	1.00	1.00	1
	I3LSY1	PIG Uncharacterized protein MRPL12	0.37	0.28	-0.13	0.24	1.00	1.00	1.00	0.99	1
	F1RU54	PIG Uncharacterized protein MRPL11	1.03	0.91	-0.33	-0.11	0.95	0.96	1.00	1.00	2
	F1SL55	PIG Uncharacterized protein MRPS22	1.89	2.44	1.51	1.39	0.63	0.24	0.75	0.83	1

Supplementary Table S5. Protein expression changes in the functionally annotated categories within cluster a.

Human equivalent Ac. No.	Accession No.	ATPase activity regulation<br>Protein	Zq (log2 FC) 120	1	4	7	FDR 120	1	4	7	No. peptides
-	I3LJX7	PIG Uncharacterized protein TOR1AIP2	3.94	5.98	1.53	-0.67	0.01	0.00	0.78	1.03	1
-	Q06AA3	PIG Regucalcin	3.5	4.2	2.69	-1.06	0.03	0.00	0.15	1.00	1
-	P09493-6	HUMAN Isoform 6 of Tropomyosin alpha-1 chain	2.83	2.51	2.25	-1.17	0.13	0.23	0.35	0.97	1
-	Q29307	PIG ATPase inhibitor, mitochondrial	1.49	0.26	0.45	1.72	0.76	1.01	1.01	0.76	6
-	F1S9B6	PIG Uncharacterized protein (Fragment) OXA1L	0.57	0.85	1.62	-0.07	1.00	0.97	0.74	1.00	1
-	P60660	HUMAN Myosin light polypeptide 6	3.09	1.15	0.32	-1.94	0.07	0.91	1.00	0.64	4
P12829	F1RRT2	PIG Uncharacterized protein (Fragment) MYL4	2.15	-0.39	0.44	0.25	0.40	1.00	1.01	1.01	3
-	A5X497	PIG Cardiac troponin I	0.04	0.73	0.95	0.4	1.00	0.97	0.96	1.02	22
P45379	I3LS66	PIG Uncharacterized protein TNNT2	1.04	1.15	0.41	-0.55	0.94	0.91	1.00	1.02	24
-	Q8MKF2	PIG Beta-tropomyosin (Fragment)	0.12	1.27	-0.52	0.94	1.01	0.88	1.01	1.02	1
-	P14632	PIG Lactotransferrin	0.71	0.86	-0.05	0.19	1.00	0.97	1.00	1.01	2
-	P09493-2	HUMAN Isoform 2 of Tropomyosin alpha-1 chain	1.09	0.98	0.29	-0.78	0.93	0.95	1.00	1.03	1
-	Q5JTV8	HUMAN Torsin-1A-interacting protein 1	0.4	0.64	0.52	-0.21	1.00	0.98	1.01	1.00	1
-	P09493-5	HUMAN Isoform 5 of Tropomyosin alpha-1 chain	2.71	1.09	-1.02	-1.75	0.17	0.93	0.95	0.74	1
O95433	F1SE06	PIG Uncharacterized protein LOC100626607	0.3	0.3	-0.01	0.31	1.00	1.00	1.00	1.02	4
Q01449	F1SSF9	PIG Uncharacterized protein MYL7	2.41	-0	-0.7	-1.81	0.30	1.00	0.99	0.73	3
-	J3KN66	HUMAN Torsin-1A-interacting protein 1	-0.31	0.44	-0.7	0.4	1.00	0.99	0.99	1.02	1
-	F1S698	PIG Uncharacterized protein TOR1AIP1	0.29	0.41	-0.91	-0.21	1.00	1.00	0.96	1.00	2
-	O95433	HUMAN Activator of 90 kDa heat shock protein ATPase homolog 1	-0.53	0.32	-0.2	-0.11	1.00	1.00	1.01	1.00	1
-	Q8TCG4	HUMAN TPMsk1 (Fragment)	2.86	1.59	-1.11	-4.03	0.12	0.73	0.93	0.00	1
-	Q719I0	HUMAN Activator of 90 kDa heat shock protein ATPase homolog 2	-0.12	0.06	-0.78	-0.29	1.01	1.00	0.98	1.02	1
-	Q92736	HUMAN Ryanodine receptor 2	-0.62	-0.68	-0.45	0.42	1.00	0.98	1.01	1.02	5
Q9UBS4	F1SFJ8	PIG Uncharacterized protein DNAJB11	0.37	0.17	-0.94	-1.07	1.00	1.00	0.96	1.00	1
-	P26678	HUMAN Cardiac phospholamban	0.33	-0.37	-0.45	-1.03	1.00	1.00	1.00	1.02	1
-	F1SNW4	PIG MYL3	3.9	1.3	-1.11	-5.74	0.01	0.87	0.93	0.00	13

-	P07951	HUMAN Tropomyosin beta chain	2.96	1.67	-1.21	-5.41	0.10	0.70	0.90	0.00	2
-	P42639	PIG Tropomyosin alpha-1 chain	3.72	1.99	-0.71	-7.39	0.01	0.50	0.99	0.00	35
-	Q01449	HUMAN Myosin regulatory light chain 2, atrial isoform	0.62	-0.37	-0.72	-2.22	1.00	1.00	0.99	0.45	1
-	A1X899	PIG Beta-tropomyosin	3.24	1.07	-1.27	-6.1	0.05	0.94	0.88	0.00	11
-	P05027	PIG Sodium/potassium-transporting ATPase subunit beta-1	-1.33	-0.91	-1.14	-0.2	0.84	0.96	0.92	1.01	7
Q92736	F1RHM3	PIG Uncharacterized protein (Fragment) RYR2	-0.66	-1.15	-1.27	-0.55	1.01	0.91	0.88	1.02	72
-	P63317	PIG Troponin C, slow skeletal and cardiac muscles	2.27	0.37	-1.6	-7.09	0.35	1.00	0.74	0.00	9
-	A1XQT6	PIG MLC1f	2.11	-1.65	-2.71	-5.26	0.42	0.70	0.15	0.00	5
-	F2Z5B6	PIG Tropomyosin alpha-1 chain	2.48	1.02	-2.03	-9.77	0.27	0.95	0.48	0.00	5
-	A5X5T5	PIG Cardiac troponin I	-3.87	-13.2	-12.9	-6.75	0.01	0.00	0.00	0.00	2

Human equivalent Ac. No.	Accession No.	Thin filament<br>Protein	Zq 120	Zq 1	Zq 4	Zq 7	FDR 120	FDR 1	FDR 4	FDR 7	No. peptides
	P09493-6	HUMAN Isoform 6 of Tropomyosin alpha-1 chain	2.83	2.51	2.25	-1.17	0.13	0.23	0.35	0.97	1
	Q9N250	PIG Titin (Fragment)	1.64	1.79	2.48	1.89	0.69	0.61	0.23	0.67	6
	P42639	PIG Tropomyosin alpha-1 chain	3.72	1.99	-0.71	-7.39	0.01	0.50	0.99	0.00	35
	K7EQW8	HUMAN Tropomyosin alpha-4 chain (Fragment)	3.28	1.65	-0.09	-3.08	0.05	0.70	1.00	0.07	1
	Q9N251	PIG Titin (Fragment)	0.67	1.03	2.9	1	1.01	0.95	0.10	1.02	7
	A1XQV4	PIG Tropomyosin alpha-3 chain	3.23	2.26	-1.06	-5.65	0.05	0.36	0.94	0.00	3
	Q29117	PIG Titin (Fragment)	0.3	0.88	2.34	2.49	1.00	0.97	0.30	0.27	21
	P07951	HUMAN Tropomyosin beta chain	2.96	1.67	-1.21	-5.41	0.10	0.70	0.90	0.00	2
	Q8TCG4	HUMAN TPMsk1 (Fragment)	2.86	1.59	-1.11	-4.03	0.12	0.73	0.93	0.00	1
	A1X899	PIG Beta-tropomyosin	3.24	1.07	-1.27	-6.1	0.05	0.94	0.88	0.00	11
	P67936	HUMAN Tropomyosin alpha-4 chain	1.48	1.18	0.13	-0.27	0.77	0.91	1.00	1.01	1
	P09493-5	HUMAN Isoform 5 of Tropomyosin alpha-1 chain	2.71	1.09	-1.02	-1.75	0.17	0.93	0.95	0.74	1
P45379	I3LS66	PIG Uncharacterized protein TNNT2	1.04	1.15	0.41	-0.55	0.94	0.91	1.00	1.02	24
	Q75ZZ6	PIG Troponin T, slow skeletal muscle	0.66	0.93	0.93	0.52	1.01	0.96	0.96	1.03	2
	Q8WZ42-6	HUMAN Isoform 6 of Titin	0.65	0.55	1.28	1.23	1.00	0.99	0.88	0.96	1
	B3VFA9	PIG Troponin I skeletal slow-twitch protein	2.04	0.75	-0.41	-0.66	0.46	0.97	1.00	1.03	3
	P09493-2	HUMAN Isoform 2 of Tropomyosin alpha-1 chain	1.09	0.98	0.29	-0.78	0.93	0.95	1.00	1.03	1
	Q8WZ42	HUMAN Titin	0.1	0.49	1.69	1.86	1.01	1.00	0.70	0.69	758

	A5X497	PIG Cardiac troponin I	0.04	0.73	0.95	0.4	1.00	0.97	0.96	1.02	22
	P02587	PIG Troponin C, skeletal muscle	1.57	-0.21	0.32	-1.55	0.72	1.00	1.00	0.85	1
	F2Z5B6	PIG Tropomyosin alpha-1 chain	2.48	1.02	-2.03	-9.77	0.27	0.95	0.48	0.00	5
	Q4JH15	PIG Troponin I	0.92	0.65	-0.11	-0.6	0.98	0.98	1.00	1.03	1
Q6P5Q4	F1SLY4	PIG Uncharacterized protein LMOD2	0.09	1.82	-0.65	-0.35	1.00	0.60	1.00	1.01	11
	P63317	PIG Troponin C, slow skeletal and cardiac muscles	2.27	0.37	-1.6	-7.09	0.35	1.00	0.74	0.00	9
	Q8MKF2	PIG Beta-tropomyosin (Fragment)	0.12	1.27	-0.52	0.94	1.01	0.88	1.01	1.02	1
	P79309	PIG Tropomyosin TM30-pl (Fragment)	0.87	0.58	-1.01	-1.73	0.99	0.99	0.96	0.75	1
Q8WZ42	I3L8Q0	PIG Uncharacterized protein	-0.43	-0.18	0.87	1.42	1.00	1.00	0.97	0.90	111
	Q8MKF3	PIG Beta-tropomyosin (Fragment)	1.92	-0.08	-1.8	-4.56	0.53	1.00	0.64	0.00	4
	Q9N0Y9	PIG Ubiquitous tropomodulin U-Tmod	0.57	-0.09	-0.78	0.17	1.00	1.00	0.98	1.00	1
	G0W2Z1	PIG Troponin T type 2 (Cardiac) (Fragment)	-0.22	0.18	-0.37	0.76	1.00	1.00	1.00	1.03	3
	G8IFA6	PIG Mitochondrial NADH dehydrogenase Fe-S protein 4	-0.09	-0.17	-0.39	0.81	1.00	1.00	1.00	1.03	1
P28289	I3LJ88	PIG Uncharacterized protein TMOD1	0.14	0.22	-1.13	-1.35	1.01	1.00	0.92	0.91	18
	O97771	PIG Titin (Fragment)	-1.86	-1.94	0.29	2.35	0.57	0.53	1.00	0.35	12
	P68137	PIG Actin, alpha skeletal muscle	-1.55	-1.73	-0.75	-0.71	0.73	0.65	0.99	1.03	38
	P29536	HUMAN Leiomodin-1	-1.6	-1.69	-1.36	-1.56	0.70	0.68	0.84	0.84	1
	A5X5T5	PIG Cardiac troponin I	-3.87	-13.2	-12.9	-6.75	0.01	0.00	0.00	0.00	2

Thin filament components (troponin-tropomyosin complex)

Human equivalent Ac. No.	Accession No.	Protein	Zq 120	Zq 1	Zq 4	Zq 7	FDR 120	FDR 1	FDR 4	FDR 7	No. peptides
	P42639	PIG Tropomyosin alpha-1 chain	3.72	1.99	-0.71	-7.39	0.01	0.50	0.99	0.00	35
	K7EQW8	HUMAN Tropomyosin alpha-4 chain (Fragment)	3.28	1.65	-0.09	-3.08	0.05	0.70	1.00	0.07	1
P45379	I3LS66	PIG Uncharacterized protein TNNT2	1.04	1.15	0.41	-0.55	0.94	0.91	1.00	1.02	24
	A5X497	PIG Cardiac troponin I	0.04	0.73	0.95	0.4	1.00	0.97	0.96	1.02	22
P28289	I3LJ88	PIG Uncharacterized protein TMOD1	0.14	0.22	-1.13	-1.35	1.01	1.00	0.92	0.91	18
	Q8TCG4	HUMAN TPMsk1 (Fragment)	2.86	1.59	-1.11	-4.03	0.12	0.73	0.93	0.00	1
	P09493-6	HUMAN Isoform 6 of Tropomyosin alpha-1 chain	2.83	2.51	2.25	-1.17	0.13	0.23	0.35	0.97	1
	P09493-5	HUMAN Isoform 5 of Tropomyosin alpha-1 chain	2.71	1.09	-1.02	-1.75	0.17	0.93	0.95	0.74	1
	A1X899	PIG Beta-tropomyosin	3.24	1.07	-1.27	-6.1	0.05	0.94	0.88	0.00	11
	P63317	PIG Troponin C, slow skeletal and cardiac muscles	2.27	0.37	-1.6	-7.09	0.35	1.00	0.74	0.00	9

	F2Z5B6	PIG Tropomyosin alpha-1 chain	2.48	1.02	-2.03	-9.77	0.27	0.95	0.48	0.00	5
	Q8MKF3	PIG Beta-tropomyosin (Fragment)	1.92	-0.08	-1.8	-4.56	0.53	1.00	0.64	0.00	4
	P02587	PIG Troponin C, skeletal muscle	1.57	-0.21	0.32	-1.55	0.72	1.00	1.00	0.85	1
	P67936	HUMAN Tropomyosin alpha-4 chain	1.48	1.18	0.13	-0.27	0.77	0.91	1.00	1.01	1
	P09493-2	HUMAN Isoform 2 of Tropomyosin alpha-1 chain	1.09	0.98	0.29	-0.78	0.93	0.95	1.00	1.03	1
	A1XQV4	PIG Tropomyosin alpha-3 chain	3.23	2.26	-1.06	-5.65	0.05	0.36	0.94	0.00	3
	Q4JH15	PIG Troponin I	0.92	0.65	-0.11	-0.6	0.98	0.98	1.00	1.03	1
	P79309	PIG Tropomyosin TM30-pl (Fragment)	0.87	0.58	-1.01	-1.73	0.99	0.99	0.96	0.75	1
	B3VFA9	PIG Troponin I skeletal slow-twitch protein	2.04	0.75	-0.41	-0.66	0.46	0.97	1.00	1.03	3
	Q9N0Y9	PIG Ubiquitous tropomodulin U-Tmod	0.57	-0.09	-0.78	0.17	1.00	1.00	0.98	1.00	1
	G0W2Z1	PIG Troponin T type 2 (Cardiac) (Fragment)	-0.22	0.18	-0.37	0.76	1.00	1.00	1.00	1.03	3
	Q8MKF2	PIG Beta-tropomyosin (Fragment)	0.12	1.27	-0.52	0.94	1.01	0.88	1.01	1.02	1
	P07951	HUMAN Tropomyosin beta chain	2.96	1.67	-1.21	-5.41	0.10	0.70	0.90	0.00	2
	Q75ZZ6	PIG Troponin T, slow skeletal muscle	0.66	0.93	0.93	0.52	1.01	0.96	0.96	1.03	2

Human equivalent Ac. No.	Accession No.	Mitochondrial ribosome<br>Protein	Zq				FDR				No. peptides
			120	1	4	7	120	1	4	7	
Q8N983-4	F1S8U4	PIG Uncharacterized protein MRPL43	4.04	3.69	0.31	1	0.00	0.01	1.00	1.02	2
I3LSY1	I3LSY1	PIG Uncharacterized protein MRPL12	1.42	2.36	2.29	1.48	0.80	0.31	0.32	0.88	1
-	P52815	HUMAN 39S ribosomal protein L12, mitochondrial	3.31	1.27	1.65	-0.3	0.04	0.87	0.71	1.01	1
-	P82933	HUMAN 28S ribosomal protein S9, mitochondrial	0.29	0.62	3.34	1.28	1.00	0.99	0.03	0.94	1
-	P82914	HUMAN 28S ribosomal protein S15, mitochondrial	2.61	0.82	0.72	0.86	0.21	0.97	0.99	1.03	2
Q9Y3B7	F1RU54	PIG Uncharacterized protein MRPL11	1.65	1.81	0.6	-0.43	0.68	0.60	1.01	1.02	3
P82673	F1SG95	PIG Uncharacterized protein MRPS35	0.63	1.66	0.17	1.09	1.00	0.70	1.00	0.99	1
-	F1S9B7	PIG Uncharacterized protein MRPL52	0.55	0.08	1.13	0.94	1.00	1.00	0.92	1.02	1
Q96EL2	F1ST79	PIG Uncharacterized protein MRPS24	0.51	1.12	0.15	-0.77	1.00	0.92	1.00	1.03	1
-	F1SL55	PIG Uncharacterized protein MRPS22	0.57	0.35	-0.28	0.17	1.00	1.00	1.00	1.01	1
-	Q767K8	PIG 28S ribosomal protein S18b, mitochondrial	1.61	0.24	0.69	-1.87	0.70	1.00	0.99	0.68	1
-	F1RK59	PIG Uncharacterized protein MRPL40	1.51	0.95	-0.67	-1.2	0.75	0.95	1.00	0.97	3
-	F1S8A5	PIG Uncharacterized protein MRPS26	0.11	-0.95	0.45	0.31	1.01	0.96	1.01	1.02	2
-	F1SRT1	PIG Uncharacterized protein MRPS11	-0.47	0.01	-0.92	-0.08	1.00	1.00	0.96	1.00	1

P82909	I3LQ99	PIG Uncharacterized protein MRPS36	-0.44	-1.17	-0.96	0.36	1.00	0.91	0.96	1.01	5
-	F1RWJ0	PIG Uncharacterized protein MRPL10	-0.81	-0.03	-0.68	-0.81	0.99	1.00	1.00	1.03	1
-	F1S8G3	PIG Uncharacterized protein MRPS33	-1.18	-1.32	-1.4	-0.29	0.90	0.86	0.83	1.02	1

Human equivalent Ac. No.	Accession No.	Fibrillar collagen<br>Protein	Zq<br>120	1	4	7	FDR<br>120	1	4	7	No. peptides
P08123	F1SFA7	PIG Uncharacterized protein COL1A2	1.88	4.26	1.31	1.83	0.55	0.00	0.86	0.70	8
P02452	I3LJX2	PIG Uncharacterized protein	2.3	2.53	1.24	2.27	0.34	0.22	0.90	0.41	2
P02452	I3LUM2	PIG Uncharacterized protein	1.82	2.35	1.88	1.74	0.59	0.31	0.58	0.75	4
-	P08123	HUMAN Collagen alpha-2(I) chain	1.57	3.48	1.45	1.27	0.72	0.02	0.81	0.95	2
-	P02461	HUMAN Collagen alpha-1(III) chain	0.42	1.75	0.47	1.27	1.00	0.63	1.01	0.95	2
P02461	F1RYI8	PIG Uncharacterized protein COL3A1	0.7	0.8	0.67	1.6	1.00	0.97	1.00	0.82	2
-	P02452	HUMAN Collagen alpha-1(I) chain	0.41	1.82	-0.15	1.27	1.00	0.60	1.00	0.95	1
-	P12107	HUMAN Collagen alpha-1(XI) chain	0.38	-0.15	1.26	1.76	1.00	1.00	0.88	0.74	1
-	Q59IP2	PIG Procollagen alpha 2(V)	1.46	0.43	-0.05	-0.09	0.77	0.99	1.00	1.01	3
P02458	I3LSV6	PIG Uncharacterized protein (Fragment) COL2A1	0.4	0.32	-0.04	0.73	1.00	1.00	1.00	1.03	1
Q8IZC6	I3LDG8	PIG Uncharacterized protein	0.93	0.15	-0.07	0.13	0.97	0.99	1.00	1.00	1
P20908	F1S021	PIG Uncharacterized protein (Fragment) COL5A1	-0.67	0.14	0.03	0.76	1.01	0.99	1.00	1.03	2
-	Q59IP1	PIG Procollagen alpha 3(V)	-0.67	-0.69	-0.87	-0.44	1.01	0.98	0.97	1.02	2
P12107	F1S571	PIG Uncharacterized protein (Fragment) COL11A1	-4.09	-4.15	-1.94	2.38	0.00	0.00	0.54	0.33	1

Human equivalent Ac. No.	Accession No.	Proteolysis<br>Protein	Zq<br>120	1	4	7	FDR<br>120	1	4	7	No. peptides
-	Q10713	HUMAN Mitochondrial-processing peptidase subunit alpha	5.37	7.17	6.2	-2.38	0.00	0.00	0.00	0.33	1
-	Q99542	HUMAN Matrix metalloproteinase-19	6.47	4.98	0.94	0.43	0.00	0.00	0.96	1.02	1
-	K9J6J4	PIG Caspase-7 isoform alpha	2.08	1.3	1.39	1.11	0.44	0.87	0.83	0.99	1
-	Q6Q781	PIG Calpastatin	1.41	1.84	0.1	0.96	0.81	0.58	1.00	1.03	5
-	P12675	PIG Calpastatin	1.69	1.56	0.61	0.27	0.67	0.75	1.01	1.01	22
-	H0Y6I4	HUMAN Matrix metalloproteinase-23, soluble form (Fragment)	0.17	2	1.05	0.24	1.01	0.50	0.94	1.01	1
Q9NY33	I3LGH6	PIG Uncharacterized protein	1.47	1.82	0.65	-0.54	0.77	0.60	1.00	1.02	3

-	Q19AZ8	PIG Prothrombin	0.55	0.14	1.09	1.45	1.00	0.99	0.93	0.88	3
-	B5L0Y2	PIG Calpastatin	0.5	1.61	0.1	0.77	1.00	0.72	1.00	1.03	1
Q99538	F1SD70	PIG Uncharacterized protein LGMN	2.08	2.15	-0.18	-1.36	0.44	0.41	1.00	0.91	1
-	P49923	PIG Lipoprotein lipase	1.03	0.79	0.65	0.18	0.94	0.97	1.00	1.00	1
-	K9IVF7	PIG X-prolyl aminopeptidase (Aminopeptidase P) 1, soluble	1.11	1.92	0.26	-0.68	0.92	0.54	1.00	1.02	6
-	Q9GLP0	PIG Integrin beta-1	0.72	1.14	0.1	0.45	1.00	0.91	1.00	1.02	8
Q8TBC4	F1SFQ0	PIG Uncharacterized protein UBA3	1.16	0.89	0.88	-0.78	0.90	0.97	0.97	1.03	2
-	Q8N2S1	HUMAN Latent-transforming growth factor beta-binding protein 4	-0.34	0.48	0.52	1.46	1.00	0.99	1.01	0.88	1
Q13049	F1SMI2	PIG Uncharacterized protein LOC100737405	0.76	0.65	-0.14	0.86	1.01	0.98	1.00	1.03	1
-	I3LD20	PIG Dystroglycan	-0.26	0.14	0.19	1.53	1.00	1.00	1.01	0.86	8
-	F1RX50	PIG Uncharacterized protein (Fragment) PITRM1	-0.22	-0.49	0.63	1.52	1.00	1.00	1.01	0.86	1
P53634	F1STR1	PIG Uncharacterized protein CTSC	0.37	0.66	0.73	-0.64	0.99	0.98	0.99	1.03	3
Q12797	I3LGF7	PIG Uncharacterized protein (Fragment) LOC100525988	0.26	-0.31	-0.23	1.3	1.00	1.01	1.00	0.94	3
-	F1RZR2	PIG Uncharacterized protein BAK1	1.17	-0.31	-0.47	0.5	0.90	1.01	1.01	1.03	1
-	G9FSR3	PIG Non-muscle myosin heavy chain II A (Fragment)	0.43	0.18	-0.46	0.58	1.00	1.00	1.01	1.03	18
-	H9TUB4	PIG UME3A	0.73	0.57	-0.93	0.2	1.00	0.99	0.96	1.01	2
-	Q16186	HUMAN Proteasomal ubiquitin receptor ADRM1	0.11	0.33	-0.23	-0.06	1.01	1.00	1.00	1.00	1
-	Q29243	PIG Dystroglycan (Fragment)	1.38	1.13	-1.48	-1.03	0.82	0.91	0.79	1.01	1
-	P00795	PIG Cathepsin D	0.28	0.02	-0.25	-0.21	1.00	1.00	1.00	1.00	8
-	I3L812	PIG Uncharacterized protein TPP1	0.1	0.58	0.18	-1.32	1.00	0.99	1.00	0.93	1
-	K9IVP5	PIG N-myosin-9	0.01	-0.43	-0.84	0.6	1.00	0.99	0.97	1.03	9
-	O43464	HUMAN Serine protease HTRA2, mitochondrial	0.53	0.67	-0.65	-1.33	1.00	0.98	1.00	0.93	1
-	B0FWK4	PIG Signal peptidase complex subunit 1	0.3	-1.01	-0.61	-0.11	1.00	0.95	1.01	1.00	1
-	F1RZL2	PIG Uncharacterized protein HTRA4	-0.37	-0.85	-0.78	0.25	0.99	0.97	0.98	1.01	1
-	P07093	HUMAN Glia-derived nexin	-0.75	-0.39	-0.88	0.22	1.01	1.00	0.97	1.01	1
Q92542	F1RJY0	PIG Uncharacterized protein NCSTN	-0.47	-1.11	0.93	-1.22	1.00	0.92	0.96	0.96	1
-	P43686	HUMAN 26S protease regulatory subunit 6B	-0.46	-0.74	-0.65	-0.53	1.00	0.97	1.00	1.03	3
-	G9BWQ1	PIG V-akt murine thymoma viral oncogene-like 1	0.1	-1.01	-1.43	-0.32	1.00	0.95	0.82	1.02	2
Q15011	I3LET0	PIG Uncharacterized protein (Fragment) NEDD8	-0.93	0.38	-7.88	0.02	0.97	1.00	0.00	1.00	1
-	O14672	HUMAN Disintegrin and metalloproteinase domain-containing protein 10	-1.16	-3.33	-4.63	-0.62	0.90	0.03	0.00	1.03	1

Supplementary Table S6. Protein expression changes in the functionally annotated categories within cluster b.

Human equivalent Ac. No.	Accession No.	Mitochondrial carriers<br>Protein	Zq 120	Zq 1	Zq 4	Zq 7	FDR 120	FDR 1	FDR 4	FDR 7	No. peptides
Q00325	F1SQT3	PIG Uncharacterized protein (Fragment) SLC25A3 [PiC]	-2.97	-2.23	-1.68	-0.35	0.10	0.38	0.71	1.01	21
-	Q6QRN9	PIG ADP/ATP translocase 3 [ANT3]	-2.32	-2.26	-1.06	0.38	0.33	0.36	0.94	1.01	15
-	O75746	HUMAN Calcium-binding mitochondrial carrier protein Aralar1	-1.53	-1.58	-1.36	-0.15	0.74	0.74	0.84	1.00	1
-	M3VK21	PIG Solute carrier family 25 (Aspartate/glutamate carrier), member 12 [Aralar 1]	-1.27	-1.36	-1.48	-0.46	0.87	0.84	0.80	1.02	5
P05141	F2Z565	PIG Uncharacterized protein SLC25A5 [ANT2]	-2.39	-0.91	-0.56	0.11	0.30	0.97	1.01	1.00	5
Q9UJS0	F1SF87	PIG Uncharacterized protein (Fragment) SLC25A13 [Aralar2]	-1.09	-0.96	-1.11	-0.51	0.93	0.95	0.93	1.03	10
-	F1RLI6	PIG Solute carrier family 25, member 46	0.17	-0.6	-1.37	-1.3	1.01	0.99	0.84	0.94	4
-	F1SUU2	PIG Uncharacterized protein SLC25A34	0.16	-0.18	-1.27	-0.43	1.01	1.00	0.88	1.02	1
P12235	F1RZQ6	PIG Uncharacterized protein SLC25A4 [ANT1]	-2.19	-1.62	2.13	0.22	0.39	0.72	0.41	1.01	4
P53007	F1RK74	PIG Uncharacterized protein SLC25A1	-0.09	-0.03	-0.01	0.53	1.01	1.00	1.00	1.03	2
-	O43772	HUMAN Mitochondrial carnitine/acylcarnitine carrier protein	0.1	-0.62	0.32	0.27	1.00	0.99	1.00	1.01	1
-	M3VH80	PIG Solute carrier family 25 (Aspartate/glutamate carrier), member 13 tv2	-1.17	1.34	-2.15	2.9	0.90	0.85	0.40	0.11	1
O43772	F1SKK1	PIG Uncharacterized protein SLC25A20 [CAC]	-0.62	-0.28	1.13	2.36	1.00	1.00	0.92	0.34	6
P19367	F1SUF2	PIG Uncharacterized protein (Fragment) HK1	-0.8	-0.71	-0.4	0.63	1.00	0.97	1.00	1.03	22
-	Q9MZ16	PIG Voltage-dependent anion-selective channel protein 1	-1.48	-1.01	-0.92	-0.85	0.77	0.95	0.96	1.03	16

Human equivalent Ac. No.	Accession No.	Fatty acid B-oxidation<br>Protein	Zq 120	Zq 1	Zq 4	Zq 7	FDR 120	FDR 1	FDR 4	FDR 7	No. peptides
-	P40939	HUMAN Trifunctional enzyme subunit alpha, mitochondrial	-2.03	-4.35	-1.46	-7.15	0.47	0.00	0.81	0.00	1
-	B2ZF49	PIG Hydroxyacyl-coenzyme A dehydrogenase	-1.13	-4.02	-2.00	-4.45	0.92	0.00	0.51	0.00	1
-	Q29554	PIG Trifunctional enzyme subunit alpha, mitochondrial	-1.54	-0.15	-0.17	1.08	0.73	0.99	1.00	1.00	43
-	P09110	HUMAN 3-ketoacyl-CoA thiolase, peroxisomal	-2.20	-2.96	-3.13	-0.91	0.38	0.09	0.05	1.03	1
P49748	I3LLE3	PIG Uncharacterized protein (Fragment) LOC100520636	-1.30	-1.05	-0.15	0.85	0.85	0.94	1.00	1.03	32
-	P11310	HUMAN Medium-chain specific acyl-CoA dehydrogenase, mitochondrial	-1.26	-1.85	-1.51	-0.77	0.88	0.58	0.79	1.03	1

-	Q9H845	HUMAN Acyl-CoA dehydrogenase family member 9, mitochondrial	-1.16	-1.38	-1.21	-1.23	0.90	0.84	0.90	0.96	1
-	K7GM59	PIG Uncharacterized protein (Fragment) ABCD1	-2.33	-1.51	-0.04	-1.08	0.33	0.77	1.00	1.00	1
P55084	F1SDN2	PIG Uncharacterized protein HADHB	-1.23	-0.54	-0.22	0.59	0.89	0.99	1.00	1.03	31
-	P00348	PIG Hydroxyacyl-coenzyme A dehydrogenase, mitochondrial	-2.10	-1.46	-1.72	-0.54	0.42	0.79	0.69	1.03	27
-	P24752	HUMAN Acetyl-CoA acetyltransferase, mitochondrial	-1.15	-1.31	-1.57	-0.30	0.90	0.86	0.75	1.01	1
-	P79274	PIG Long-chain specific acyl-CoA dehydrogenase, mitochondrial	-0.78	-1.12	1.09	-0.17	1.00	0.92	0.93	1.00	23
-	A7YB26	PIG Peroxisomal enoyl coenzyme A hydratase 1 (Fragment)	-0.63	-1.33	-1.17	-0.78	1.00	0.86	0.92	1.03	1
-	D0G0B3	PIG Acetyl-Coenzyme A acyltransferase 2	-0.53	-0.02	0.63	1.23	1.00	1.00	1.01	0.96	21
P24752	I3LP02	PIG Uncharacterized protein ACAT1	-2.27	-1.11	-0.82	-0.53	0.35	0.92	0.97	1.03	20
-	Q99714	HUMAN 3-hydroxyacyl-CoA dehydrogenase type-2	-1.24	-1.68	0.23	-0.91	0.88	0.68	1.00	1.03	1
-	F1RL48	PIG Uncharacterized protein ACAD10	-0.04	-0.28	-1.37	-1.85	1.00	1.00	0.84	0.70	1
-	P55931	PIG Electron transfer flavoprotein-ubiquinone oxidoreductase, mitochondrial	-1.21	-1.01	-0.75	-0.11	0.89	0.95	0.99	1.00	20
-	Q8HY46	PIG Carnitine O-palmitoyltransferase 1, muscle isoform	-0.54	-0.41	-1.08	-0.36	1.00	1.00	0.93	1.01	19
-	P41367	PIG Medium-chain specific acyl-CoA dehydrogenase, mitochondrial	-0.37	0.88	0.52	-0.33	0.99	0.97	1.01	1.02	16
P23786	F1S764	PIG Uncharacterized protein CPT2	-0.33	-0.66	0.35	-0.31	1.00	0.98	1.00	1.01	14
I3LJ48	I3LJ48	PIG Uncharacterized protein (Fragment) EHHADH	-0.05	0.24	-1.21	-1.51	1.00	1.00	0.90	0.87	1
P30084	F1SAC1	PIG Uncharacterized protein ECHS1	-1.97	-2.35	-2.50	-2.86	0.50	0.31	0.23	0.12	13
P45954	F1SED0	PIG Uncharacterized protein (Fragment) ACADSB	-0.18	0.22	-0.76	-1.45	1.01	1.00	0.98	0.88	12
-	P79273	PIG Short-chain specific acyl-CoA dehydrogenase, mitochondrial	-0.53	-0.89	-0.86	-0.78	1.00	0.97	0.97	1.03	11
-	K7GRM6	PIG Uncharacterized protein (Fragment) HSD17B10	0.15	0.19	-0.58	-1.65	1.01	1.00	1.01	0.80	1
Q9H845	I3LUR5	PIG Uncharacterized protein (Fragment) ACAD9	0.00	-0.37	0.28	-0.26	1.00	1.00	1.00	1.01	10
P51659	I3LEF8	PIG Uncharacterized protein HSD17B4	-0.29	-0.09	-0.63	-0.20	1.00	1.00	1.01	1.01	7
Q6JQN1	I3LK34	PIG Uncharacterized protein LOC100625244	0.01	-0.35	0.00	-1.05	1.00	1.00	1.00	1.00	6
Q13011	I3LJJ4	PIG Uncharacterized protein ECH1	-0.63	-0.91	-1.05	-0.85	1.01	0.97	0.94	1.03	5
-	Q28956	PIG 17beta-estradiol dehydrogenase	1.09	1.59	-0.13	-0.19	0.93	0.73	1.00	1.01	5
Q6JQN1	I3LDT4	PIG Uncharacterized protein	0.29	-0.46	0.14	-1.10	1.00	0.99	1.00	0.99	4
-	Q3Y5G5	PIG Peroxisomal enoyl coenzyme A hydratase 1	-1.81	-1.33	-0.53	-0.25	0.60	0.86	1.01	1.01	3
Q99714	K7GND4	PIG Uncharacterized protein (Fragment) HSD17B10	-0.81	-0.53	-0.36	-1.28	1.00	1.00	1.00	0.95	3
Q99714	K7GMY3	PIG Uncharacterized protein (Fragment) HSD17B10	0.71	-0.16	0.16	-0.25	1.00	1.00	1.00	1.01	3
-	P45954	HUMAN Short/branched chain specific acyl-CoA dehydrogenase, mitochondrial	-0.41	0.53	-0.70	0.06	1.00	1.00	0.99	1.00	1
-	P42765	HUMAN 3-ketoacyl-CoA thiolase, mitochondrial	-0.94	-1.29	-1.17	-0.99	0.97	0.87	0.92	1.02	2
-	I3LAI1	PIG Uncharacterized protein (Fragment) ECHS1	-0.95	-0.63	-1.62	-0.72	0.96	0.99	0.74	1.03	2

-	O43772	HUMAN Mitochondrial carnitine/acylcarnitine carrier protein	0.10	-0.62	0.32	0.27	1.00	0.99	1.00	1.01	1
-	Q16698	HUMAN 2,4-dienoyl-CoA reductase, mitochondrial	-0.84	-1.31	0.30	-1.96	0.99	0.86	1.00	0.63	2
-	Q16836	HUMAN Hydroxyacyl-coenzyme A dehydrogenase, mitochondrial	-0.18	-0.42	-1.09	-0.53	1.01	1.00	0.93	1.03	2
-	H9BYW3	PIG Acyl-coenzyme A oxidase	1.04	0.89	-0.62	-0.52	0.94	0.97	1.01	1.03	1
-	P28330	HUMAN Long-chain specific acyl-CoA dehydrogenase, mitochondrial	-0.50	-1.12	1.14	-0.71	1.00	0.92	0.92	1.03	2
-	F1RUP0	PIG Uncharacterized protein (Fragment) ECHDC3	-0.72	-0.05	0.33	0.08	1.00	1.00	1.00	1.00	2

Thick filament

Human equivalent Ac. No.	Accession No.	Protein	Zq 120	Zq 1	Zq 4	Zq 7	FDR 120	FDR 1	FDR 4	FDR 7	No. peptides
-	Q9UKX2	HUMAN Myosin-2	1.76	-3.1	-4.51	-4.85	0.62	0.06	0.00	0.00	1
-	Q14896	HUMAN Myosin-binding protein C, cardiac-type	-2.14	-1.94	-1.68	-1.18	0.40	0.53	0.71	0.98	2
-	P79293	PIG Myosin-7	-2.48	-2.28	-1.39	-0.13	0.26	0.35	0.83	1.00	229
P11055	F1SS61	PIG Uncharacterized protein MYH3	-1.91	-1.35	-1.79	-0.66	0.53	0.85	0.65	1.03	10
Q14324	F1SID7	PIG Uncharacterized protein MYBPC3	-2.07	-2.26	-1.2	0.01	0.44	0.36	0.90	1.00	84
-	P12883	HUMAN Myosin-7	-1.91	-0.56	-1.7	-0.94	0.54	0.99	0.69	1.02	3
Q96K31	F1RR11	PIG Uncharacterized protein LOC100517937	-1.59	-1.43	-1.23	-0.38	0.71	0.81	0.90	1.01	1
P52179	F1SM75	PIG Uncharacterized protein MYOM1	-1.32	-1.62	-1.17	-0.03	0.84	0.72	0.91	1.00	99
-	Q9TV61	PIG Myosin-1	-1.67	-1.59	-1	0.42	0.68	0.73	0.96	1.02	70
-	P11055	HUMAN Myosin-3	-0.89	-1.25	-0.8	-0.74	0.99	0.88	0.98	1.03	6
-	Q9TV63	PIG Myosin-2	-0.97	-0.67	-0.11	0.02	0.96	0.98	1.00	1.00	15
Q9UPQ3	I3LTX4	PIG Uncharacterized protein	0.04	-0.15	0.23	-0.1	1.00	0.99	1.00	1.00	1
-	F1S9D6	PIG Myosin-7	1.65	1.03	-0.67	-0.16	0.68	0.95	1.00	1.00	2
Q9NU22	F1RY21	PIG Uncharacterized protein	0.23	0.77	0.26	0.72	1.00	0.97	1.00	1.03	1
-	E7EX84	HUMAN Myosin-2 (Fragment)	0.76	0.73	1	1.93	1.01	0.97	0.96	0.65	1
-	P12882	HUMAN Myosin-1	6.28	1.36	2.98	3.93	0.00	0.84	0.08	0.00	1

Accessory contractile p.

Human equivalent Ac. No.	Accession No.	Protein	Zq 120	Zq 1	Zq 4	Zq 7	FDR 120	FDR 1	FDR 4	FDR 7	No. peptides
-	Q14896	HUMAN Myosin-binding protein C, cardiac-type	-2.14	-1.94	-1.68	-1.18	0.40	0.53	0.71	0.98	2
P98160	F1SU03	PIG Uncharacterized protein HSPG2	-0.79	-1.86	-1.9	-1.28	1.00	0.57	0.57	0.94	16

-	P98160	HUMAN Basement membrane-specific heparan sulfate proteoglycan core protein	-0.98	-2.05	-1.69	-1.07	0.96	0.47	0.69	1.00	7
P54296	F1RVL5	PIG Uncharacterized protein (Fragment) MYOM2	-1.78	-2.16	-1.68	-0.08	0.61	0.41	0.70	1.00	63
Q14324	F1SID7	PIG Uncharacterized protein MYBPC3	-2.07	-2.26	-1.2	0.01	0.44	0.36	0.90	1.00	84
-	P54296	HUMAN Myomesin-2	-1.2	-2.59	-0.59	-0.59	0.89	0.20	1.01	1.03	3
F1SJL0	F1SJL0	PIG Uncharacterized protein IGDCC3	-1.87	-1.75	-1.6	0.35	0.56	0.63	0.74	1.01	1
P52179	F1SM75	PIG Uncharacterized protein MYOM1	-1.32	-1.62	-1.17	-0.03	0.84	0.72	0.91	1.00	99
-	G8FUN4	PIG Myomesin family member 3 (Fragment)	-0.97	-1.46	-0.98	-0.03	0.97	0.79	0.96	1.00	5
P98160	I3LLD8	PIG Uncharacterized protein (Fragment) LOC100626701	-0.12	-0.41	-1.6	-1.15	1.01	1.00	0.74	0.98	8
I3LB92	I3LB92	PIG Uncharacterized protein (Fragment) MYOM3	-1.35	-1.46	-1.02	0.56	0.83	0.79	0.95	1.03	14
-	F1RH19	PIG Uncharacterized protein MYBPC2	-0.7	-1.26	-0.84	-0.19	1.00	0.87	0.97	1.00	1
P98160	I3LAA4	PIG Uncharacterized protein	-0.61	-1.42	-0.42	-0.06	1.00	0.81	1.00	1.01	4
Q86TC9	I3LIB5	PIG Uncharacterized protein MYPN	-0.48	-0.89	-0.62	-0.51	1.00	0.97	1.01	1.03	10
O60229	I3LAJ5	PIG Uncharacterized protein (Fragment)	-1.33	-0.65	-0.51	0.09	0.84	0.98	1.00	1.01	1
P54296	I3LES8	PIG Uncharacterized protein (Fragment)	-1.21	-1.16	-1.04	1.03	0.89	0.90	0.95	1.01	6
-	Q5VTT5	HUMAN Myomesin-3	-0.83	-0.77	-0.74	0.03	0.99	0.97	0.99	1.00	18
P98160	F1SU00	PIG Uncharacterized protein (Fragment)	0.16	-0.49	-1	-0.75	1.01	1.00	0.96	1.03	2
F1RZ06	F1RZ06	PIG Uncharacterized protein (Fragment) MXRA5	-0.94	-0.72	0.17	-0.46	0.96	0.97	1.00	1.02	2
Q8IZU9	F1S6H0	PIG Uncharacterized protein KIRREL3	-0.8	-0.1	-0.62	0.24	1.00	1.00	1.01	1.01	1
-	P43121	HUMAN Cell surface glycoprotein MUC18	-1.08	0.22	-0.54	0.64	0.93	1.00	1.01	1.03	1
-	Q96JA1	HUMAN Leucine-rich repeats and immunoglobulin-like domains protein 1	-0	0.07	-0.41	-0.32	1.00	1.00	1.00	1.02	1
-	O60229	HUMAN Kalirin	1.01	1.54	-0.53	-1.68	0.95	0.75	1.01	0.78	1
-	Q15746	HUMAN Myosin light chain kinase, smooth muscle	1.13	0.38	-0.15	-0.64	0.92	1.00	1.00	1.03	1
-	F1SFR8	PIG Uncharacterized protein LRIG1	0.86	0.28	0.34	-0.32	0.99	1.00	1.00	1.02	1
Q9UBF9	F1RH92	PIG Uncharacterized protein MYOT	-0.39	0.7	0.69	0.98	1.00	0.97	0.99	1.02	16

Mitochondrial complexes

Human equivalent Ac. No.	Accession No.	Protein	Zq 120	Zq 1	Zq 4	Zq 7	FDR 120	FDR 1	FDR 4	FDR 7	No. peptides
-	Q35914	PIG ATP synthase protein 8	-0.47	-3.69	-12.3	-12.8	1.00	0.01	0.00	0.00	1
O75964	F1SAK6	PIG Uncharacterized protein ATP5L	-2.3	-2.97	-2.79	-1.77	0.34	0.09	0.13	0.74	6
-	I3LER5	PIG Cytochrome c oxidase subunit 4 isoform 1, mitochondrial	-3.51	-3.64	-1.88	0.18	0.02	0.01	0.58	1.00	4
O75489	F1SIF2	PIG Uncharacterized protein NDUFS3	-1.05	-1.79	-2.38	-2.73	0.94	0.62	0.28	0.16	14

-	A5GFX4	PIG ATP synthase, H+ transporting, mitochondrial F1 complex, epsilon subunit	-2.72	-3.42	-1.4	-0.29	0.16	0.03	0.83	1.02	2
-	P0C7P4	HUMAN Putative cytochrome b-c1 complex subunit Rieske-like protein 1	0.01	-1.47	-4.08	-2.22	1.00	0.79	0.00	0.45	1
-	F1SGC6	PIG Uncharacterized protein NDUFB5	-1.89	-1.85	-1.73	-1.07	0.55	0.58	0.68	1.00	9
-	G8JE99	HUMAN Cytochrome c oxidase subunit 2	-0.66	-1.12	-2.64	-1.73	1.01	0.92	0.17	0.75	1
-	K7GMN1	PIG Uncharacterized protein COX7B	-2.75	-2.59	-2.19	1.53	0.16	0.20	0.38	0.86	2
-	F1RQI1	PIG Uncharacterized protein COX7A2	-1.67	-1.27	-2.2	-0.75	0.68	0.87	0.38	1.03	2
O95299	F1SIS9	PIG Uncharacterized protein NDUFA10	-1.1	-0.78	-1.65	-2.02	0.92	0.97	0.71	0.58	14
-	F1SJP6	PIG NADH dehydrogenase [ubiquinone] 1 alpha subcomplex subunit 6 (Fragment)	-1.24	-0.74	-2.09	-1.23	0.88	0.97	0.44	0.96	7
O95168	I3LPW0	PIG Uncharacterized protein NDUFB4	-1.16	-0.9	-1.78	-1.4	0.90	0.97	0.65	0.90	7
-	Q5S3G4	PIG Cytochrome c oxidase subunit 5B, mitochondrial	-0.79	-2.51	-1.66	-0.26	1.00	0.23	0.71	1.01	9
O14949	F1RI18	PIG Uncharacterized protein UQCRQ	-2.22	-1.65	-1.22	-0.09	0.37	0.70	0.90	1.00	7
-	D0VWV4	PIG Succinate dehydrogenase cytochrome b560 subunit, mitochondrial	-2.37	-1.57	-1.55	0.35	0.31	0.75	0.76	1.01	1
-	F1RNZ1	PIG Cytochrome b-c1 complex subunit Rieske, mitochondrial	-1.57	-1.63	-1.36	-0.41	0.72	0.72	0.84	1.02	15
-	P22695	HUMAN Cytochrome b-c1 complex subunit 2, mitochondrial	-0.98	-0.66	-1.26	-1.94	0.96	0.98	0.89	0.64	2
P24539	F1SBN7	PIG Uncharacterized protein ATP5F1	-2.06	-1.71	-1.38	0.33	0.45	0.66	0.84	1.02	14
-	F1STY1	PIG NADH dehydrogenase [ubiquinone] 1 subunit C2	-1.91	-1.54	-1.31	-0.03	0.53	0.76	0.87	1.00	8
P51970	F1SLR1	PIG Uncharacterized protein NDUFA8	-0.91	-1.25	-1.3	-1.12	0.98	0.88	0.88	0.99	6
-	Q2EN81	PIG ATP synthase subunit O, mitochondrial	-1.7	-1.62	-1.22	0.1	0.66	0.72	0.90	1.00	16
-	P24964	PIG Cytochrome b	-2.51	-1.78	-1.16	1.05	0.25	0.62	0.91	1.00	4
-	Q8SPJ9	PIG Cytochrome c oxidase subunit 7A1, mitochondrial	-1.58	-1.58	-1.39	0.2	0.71	0.74	0.83	1.00	2
-	Q0QF01	PIG Succinate dehydrogenase [ubiquinone] flavoprotein subunit, mitochondrial	-1.5	-1.4	-0.94	-0.51	0.76	0.82	0.96	1.03	24
-	Q95283	PIG Cytochrome c oxidase subunit 4 isoform 1, mitochondrial (Fragment)	-1.62	-1.7	-0.92	-0.05	0.70	0.67	0.96	1.00	10
P20674	F1SJ34	PIG Uncharacterized protein COX5A	0.61	-1.08	-2.39	-1.35	1.00	0.93	0.28	0.91	10
P49821	F1RVN1	PIG Uncharacterized protein NDUFV1	-1.18	-0.98	-0.73	-1.31	0.90	0.95	0.99	0.94	16
-	P50667	PIG Cytochrome c oxidase subunit 2	-0.95	-0.96	-1.74	-0.53	0.97	0.96	0.68	1.03	10
-	Q29235	PIG ATP synthase beta chain, mitochondrial (Fragment)	0.14	-0.74	-1.68	-1.88	1.01	0.97	0.70	0.68	2
-	P06576	HUMAN ATP synthase subunit beta, mitochondrial	-0.58	-1	-0.87	-1.63	1.00	0.95	0.97	0.80	1
-	P14927	HUMAN Cytochrome b-c1 complex subunit 7	-1.1	-0.94	-0.82	-1.14	0.93	0.96	0.97	0.98	6
-	Q95339	PIG ATP synthase subunit f, mitochondrial	-0.83	-0.12	-1.79	-1.26	0.99	1.00	0.65	0.95	2
-	A1XQT2	PIG Cytochrome c oxidase subunit 6C	-2.28	-1.78	-0.7	0.82	0.35	0.62	0.99	1.03	12
O75439	F1SKM0	PIG Uncharacterized protein UQCRC1	-0.59	-0.66	-0.97	-1.69	1.00	0.98	0.96	0.77	20
Q16718	F1SLY2	PIG Uncharacterized protein NDUFA5	-0.71	-0.41	-0.89	-1.74	1.00	1.00	0.96	0.75	5

Q9P0J0	F1S6Q1	PIG Uncharacterized protein NDUFA13	-1.07	-0.72	-1.39	-0.54	0.94	0.97	0.83	1.03	13
-	P36542-2	HUMAN Isoform Heart of ATP synthase subunit gamma, mitochondrial	-0.43	0.93	-3.37	-0.72	1.00	0.96	0.03	1.03	1
P14854	F1RLH7	PIG Uncharacterized protein COX6B	-1.23	-1.35	-1.35	0.69	0.89	0.85	0.84	1.03	4
F1SD73	F1SD73	PIG Uncharacterized protein NDUFB1	-1.11	-0.86	-0.92	-0.26	0.92	0.97	0.96	1.01	1
-	A5GZW8	PIG Succinate dehydrogenase [ubiquinone] cytochrome b small subunit	-1.53	-1.02	-0.58	0.23	0.74	0.95	1.01	1.01	2
-	Q29259	PIG NADH dehydrogenase [ubiquinone] 1 beta subcomplex subunit 6	-1.63	-0.95	-0.58	0.4	0.70	0.96	1.01	1.02	3
Q9NX14	F1RWV4	PIG Uncharacterized protein NDUFB11	-1.05	-1.04	-1.29	0.62	0.93	0.95	0.88	1.03	6
-	P14854	HUMAN Cytochrome c oxidase subunit 6B1	-0.63	-0.26	-1.24	-0.53	1.01	1.01	0.90	1.03	1
-	O79876	PIG Cytochrome c oxidase subunit 1	-0.76	-0.85	-0.41	-0.56	1.01	0.97	1.00	1.03	1
Q9UI09	F1SQP4	PIG Uncharacterized protein (Fragment) NDUFA12	-0.74	-0.71	-0.31	-0.54	1.01	0.97	1.00	1.03	11
O75306	F1S1A8	PIG Uncharacterized protein NDUFS2	0.37	0	-1.17	-1.45	0.99	1.00	0.92	0.88	17
Q9Y6M9	F1RRP9	PIG Uncharacterized protein NDUFB9	-0.88	-0.99	-0.01	-0.35	0.99	0.95	1.00	1.01	5
-	I3LDC3	PIG Uncharacterized protein NDUFB10	0.02	0.16	-0.84	-1.49	1.00	1.00	0.97	0.87	8
P28331	F1SHD7	PIG Uncharacterized protein (Fragment) NDUFS1	-0.17	-0.14	-0.77	-0.98	1.01	0.99	0.98	1.02	32
-	O00217	HUMAN NADH dehydrogenase [ubiquinone] iron-sulfur protein 8, mitochondrial	-0.46	-0.05	-0.03	-1.23	1.00	1.00	1.00	0.96	4
-	F1SV23	PIG Uncharacterized protein NDUFS5	0.13	-0.28	-0.84	-0.62	1.01	1.00	0.97	1.03	3
-	F1SI50	PIG Uncharacterized protein NDUFB3	-0.41	-0.48	-0.45	-0.12	1.00	0.99	1.00	1.00	2
-	K7GLT8	PIG ATP synthase subunit beta	-0.72	-1.13	0.83	-0.25	1.00	0.92	0.97	1.01	2
-	I3LGM4	PIG Uncharacterized protein NDUFA11	-0.43	0.08	-0.32	-0.51	1.00	1.00	1.00	1.03	2
O95178	F1SRQ0	PIG Uncharacterized protein (Fragment) NDUFB2	0.1	-0.42	-0.94	0.14	1.01	1.00	0.96	1.00	1
-	Q0QEM6	PIG ATP synthase subunit beta (Fragment)	0.76	0.16	0.39	-1.93	1.01	1.00	1.01	0.65	37
O14548	F1S4V0	PIG Uncharacterized protein (Fragment) COX7A2L	-0.36	-0.21	0	-0.03	0.99	1.00	1.00	1.00	2
-	Q007T0	PIG Succinate dehydrogenase [ubiquinone] iron-sulfur subunit, mitochondrial	-0.28	-0.38	-0.24	0.33	1.00	1.00	1.00	1.02	12
-	P80021	PIG ATP synthase subunit alpha, mitochondrial	-0.58	-0.73	0.89	-0.11	1.00	0.97	0.96	1.00	44
I3LRR4	I3LRR4	PIG Uncharacterized protein (Fragment) NDUFV3	0.56	-0.41	-0.46	-0.11	1.00	0.99	1.01	1.00	13
-	Q69GF7	PIG Cytochrome c oxidase subunit 2	0.53	-0.42	-0.82	0.4	1.00	1.00	0.97	1.02	2
-	Q9MYT8	PIG ATP synthase subunit e, mitochondrial	-0.61	-0.94	-0.84	2.35	1.00	0.96	0.97	0.35	4
P19404	F1SM98	PIG Uncharacterized protein NDUFV2	1.16	0.82	-0.2	-1.8	0.90	0.97	1.01	0.73	12
F1RRC9	F1RRC9	PIG Uncharacterized protein NDUFC1	-0.87	-0.59	0.36	1.14	0.99	0.99	1.00	0.98	2
-	P36542	HUMAN ATP synthase subunit gamma, mitochondrial	-0.33	-0.46	1.42	-0.35	1.00	0.99	0.82	1.01	2
-	B2R4A2	HUMAN Cytochrome b-c1 complex subunit 7	-0.12	0.38	0.37	0.28	1.01	1.00	1.00	1.01	1
-	F8W0P7	HUMAN ATP synthase subunit beta, mitochondrial (Fragment)	1.4	0.82	0.48	-1.77	0.81	0.97	1.01	0.74	2

Q99766	F1SHX8	PIG Uncharacterized protein (Fragment) ATP5S	0.14	0.19	0.43	0.33	1.01	1.00	1.01	1.02	4
-	I3LTV6	PIG Cytochrome c oxidase subunit 6A, mitochondrial	-0	-0.34	2.05	-0.43	1.00	1.00	0.47	1.02	2
-	Q2EN79	PIG Ubiquinol-cytochrome c reductase complex	-0.69	-0.24	1.16	1.09	1.01	1.00	0.92	0.99	1
O75380	F1S031	PIG Uncharacterized protein NDUFS6	1.7	0.99	-1.33	0.01	0.66	0.95	0.86	1.00	6
-	H0YI37	HUMAN ATP synthase subunit beta, mitochondrial (Fragment)	0.28	0.97	0.64	-0.48	1.00	0.95	1.00	1.02	1
-	F1S6Q7	PIG ATP synthase subunit delta, mitochondrial	2.73	0.93	-0.44	-1.69	0.16	0.96	1.00	0.77	3
-	P25705	HUMAN ATP synthase subunit alpha, mitochondrial	1.24	-0.36	0.1	0.56	0.89	1.00	1.00	1.03	2
-	O43920	HUMAN NADH dehydrogenase [ubiquinone] iron-sulfur protein 5	0.33	0.48	0.39	0.73	1.00	0.99	1.00	1.03	1
-	F1S3W0	PIG Cytochrome b-c1 complex subunit 6	3.34	1.88	-0.13	-3.13	0.04	0.56	1.00	0.06	4
-	P13618	PIG ATP synthase-coupling factor 6, mitochondrial	1.9	0	-0.02	0.1	0.54	1.00	1.00	1.00	10
P17568	F1SCH1	PIG Uncharacterized protein (Fragment) NDUFB7	1.58	0.44	0.3	1.15	0.71	0.99	1.00	0.98	3
-	Q29307	PIG ATPase inhibitor, mitochondrial	1.49	0.26	0.45	1.72	0.76	1.01	1.01	0.76	6
-	O95169	HUMAN NADH dehydrogenase [ubiquinone] 1 beta subcomplex subunit 8	1.17	1.86	0.85	0.09	0.90	0.57	0.97	1.01	1
-	Q94PW4	PIG ATP synthase protein 8	-0.67	1.24	2.3	1.85	1.01	0.88	0.32	0.70	2
-	F1SRG2	PIG NADH dehydrogenase [ubiquinone] 1 alpha subcomplex subunit 6	1.86	7.68	8.14	17.9	0.56	0.00	0.00	0.00	1

TCA cycle

Human equivalent Ac. No.	Accession No.	Protein	Zq				FDR				No. peptides
			120	1	4	7	120	1	4	7	
-	O43837	HUMAN Isocitrate dehydrogenase [NAD] subunit beta, mitochondrial	-1.63	-1.34	-2.36	-1.29	0.69	0.85	0.29	0.94	1
-	P00889	PIG Citrate synthase, mitochondrial	-2.33	-2.73	-1.28	-0.26	0.33	0.15	0.88	1.01	17
-	P33198	PIG Isocitrate dehydrogenase [NADP], mitochondrial (Fragment)	-1.34	-1.51	-1.4	-1.06	0.84	0.77	0.83	1.00	29
-	D0VWV4	PIG Succinate dehydrogenase cytochrome b560 subunit, mitochondrial	-2.37	-1.57	-1.55	0.35	0.31	0.75	0.76	1.01	1
-	P00346	PIG Malate dehydrogenase, mitochondrial	-1.64	-1.27	-1.33	-0.81	0.69	0.87	0.86	1.03	18
-	I3LPP1	PIG Fumarate hydratase, mitochondrial	-1.25	-1.59	-0.97	-1.12	0.88	0.74	0.96	0.98	2
-	P16276	PIG Aconitate hydratase, mitochondrial	-2.27	-2.08	-0.76	0.22	0.35	0.46	0.98	1.01	42
-	F1SEN2	PIG Glutamate dehydrogenase 1, mitochondrial	-0.81	-0.96	-1.41	-1.36	1.00	0.95	0.82	0.91	17
-	O19069	PIG Succinyl-CoA ligase [ADP/GDP-forming] subunit alpha, mitochondrial	-1.39	-1.44	-0.94	-0.71	0.82	0.80	0.96	1.03	11
-	Q0QF01	PIG Succinate dehydrogenase [ubiquinone] flavoprotein subunit, mitochondrial	-1.5	-1.4	-0.94	-0.51	0.76	0.82	0.96	1.03	24
-	K9IVI1	PIG 2-oxoglutarate dehydrogenase, mitochondrial	-1.4	-1.47	-0.99	-0.48	0.81	0.79	0.96	1.02	46
-	F1RK10	PIG Succinyl-CoA ligase [ADP-forming] subunit beta, mitochondrial	-0.76	-0.62	-1.47	-0.49	1.01	0.99	0.80	1.02	2
-	O97580	PIG Succinyl-CoA ligase [ADP-forming] subunit beta, mitochondrial (Fragment)	-1.02	-1.13	-0.93	0.08	0.95	0.91	0.96	1.01	17

-	A5GZW8	PIG Succinate dehydrogenase [ubiquinone] cytochrome b small subunit	-1.53	-1.02	-0.58	0.23	0.74	0.95	1.01	1.01	2
-	Q1G1K7	PIG Mitochondrial NAD+isocitrate dehydrogenase 3 beta variant 2	-0.56	-0.77	-0.94	-0.63	1.00	0.97	0.96	1.03	18
-	P09623	PIG Dihydrolipoyl dehydrogenase, mitochondrial	-0.94	-1.06	-0.36	-0.26	0.96	0.94	1.00	1.01	17
-	F1SAF0	PIG Dihydrolipoyl dehydrogenase	0.05	0.19	-1.65	-1.21	1.00	1.00	0.71	0.96	1
P11177	F1SGH5	PIG Uncharacterized protein PDHB	-0.74	-0.1	-0.53	-1.02	1.01	1.00	1.01	1.01	17
P35232	F1RWF5	PIG Uncharacterized protein	-0.79	-0.31	-0.93	-0.2	1.00	1.01	0.96	1.01	12
-	P53590	PIG Succinyl-CoA ligase [GDP-forming] subunit beta, mitochondrial (Fragment)	-0.07	0	-0.23	-1.43	1.00	1.00	1.00	0.89	19
-	P10173	PIG Fumarate hydratase, mitochondrial	-0.39	-0.5	-0.26	-0.45	1.00	1.00	1.00	1.02	26
-	F1S297	PIG Isocitrate dehydrogenase [NAD] subunit gamma, mitochondrial	-0.07	-0.28	-0.51	-0.71	1.00	1.00	1.01	1.03	11
-	H0YIC4	HUMAN Citrate synthase (Fragment)	-0.89	-0.54	-0.49	0.88	0.99	0.99	1.00	1.03	1
-	P48735	HUMAN Isocitrate dehydrogenase [NADP], mitochondrial	0.47	0.22	-0.44	-0.92	1.00	1.00	1.00	1.03	2
-	Q007T0	PIG Succinate dehydrogenase [ubiquinone] iron-sulfur subunit, mitochondrial	-0.28	-0.38	-0.24	0.33	1.00	1.00	1.00	1.02	12
-	F1RKU0	PIG Isocitrate dehydrogenase [NAD] subunit alpha, mitochondrial	-0.22	-0.01	0.33	-0.66	1.00	1.00	1.00	1.03	13
Q9NX18	F1RKQ9	PIG Uncharacterized protein LOC100519294	0.17	0.05	-0.69	0.28	1.01	1.00	0.99	1.01	1
-	P11177	HUMAN Pyruvate dehydrogenase E1 component subunit beta, mitochondrial	0.32	-0.3	0.2	-0.29	1.00	1.00	1.01	1.02	1
-	P56471	PIG Isocitrate dehydrogenase [NAD] subunit alpha, mitochondrial (Fragments)	0.33	0.63	0	-0.81	1.00	0.99	1.00	1.03	3
O14950	I3LBF7	PIG Uncharacterized protein (Fragment) LOC733637	1.46	0.88	-0.54	-0.92	0.78	0.97	1.01	1.03	2
-	P42174	PIG Glutamate dehydrogenase 1, mitochondrial (Fragments)	1.35	0.41	-0.91	0.03	0.83	1.00	0.96	1.00	1
-	P11708	PIG Malate dehydrogenase, cytoplasmic	-0.57	-0.78	2.11	0.24	1.00	0.97	0.43	1.01	24
-	P29803	HUMAN Pyruvate dehydrogenase E1 component subunit alpha	0.64	2.93	-0.65	-0.22	1.00	0.10	1.00	1.00	1
-	Q9N0F1	PIG Dihydrolipoyllysine-residue succinyltransferase	0.68	0.63	2.16	-0.69	1.01	0.99	0.39	1.03	16
-	I3LDC7	PIG Isocitrate dehydrogenase [NADP] (Fragment)	0.63	1.73	0.66	-0.01	1.00	0.64	1.00	1.00	9

Supplementary Table S7. Protein expression changes in the functionally annotated categories within cluster c.

Human equivalent Ac. No.	Accession No.	Acute phase response signaling<br>Protein	Zq 120	Zq 1	Zq 4	Zq 7	FDR 120	FDR 1	FDR 4	FDR 7	No. peptides
	Q8SPS7	PIG Haptoglobin	3.34	4.13	9.47	3.97	0.04	0.00	0.00	0.00	11
	F1SH92	PIG Inter-alpha-trypsin inhibitor heavy chain H4	-0.12	2.85	5.5	10.9	1.01	0.11	0.00	0.00	2
	P79263	PIG Inter-alpha-trypsin inhibitor heavy chain H4	-0.33	2.05	4.04	9.41	1.00	0.47	0.00	0.00	23
	P14477	PIG Fibrinogen beta chain (Fragment)	0.24	1.08	3.32	5.04	1.00	0.93	0.03	0.00	1
	I3LQ17	PIG Uncharacterized protein	1.23	0.42	4.23	2.57	0.89	1.00	0.00	0.24	23
	P00747	HUMAN Plasminogen	2.18	2	2.1	1.78	0.39	0.50	0.44	0.73	1
	P00450	HUMAN Ceruloplasmin	0.74	0.03	4.52	2.55	1.00	1.00	0.00	0.25	1
	P09571	PIG Serotransferrin	2.85	1.51	3.81	-0.61	0.13	0.77	0.01	1.03	28
P02675	I3L651	PIG Uncharacterized protein	-0.75	0.09	3.13	4.68	1.01	1.00	0.05	0.00	20
	I3LQR9	PIG Fibrinogen alpha chain	-1.01	0.06	2.75	5.27	0.95	1.00	0.14	0.00	9
	F1RX36	PIG Fibrinogen alpha chain	-1.36	-0.25	2.97	5.7	0.83	1.01	0.08	0.00	4
	I3L818	PIG Uncharacterized protein (Fragment) SERPINF2	1.5	2.06	2.66	0.68	0.76	0.46	0.16	1.03	2
	P50828	PIG Hemopexin	1.73	0.15	3.78	0.56	0.64	1.00	0.01	1.03	8
	K9J6H8	PIG Alpha-2-macroglobulin	0.66	0.44	3.51	0.97	1.00	0.99	0.02	1.02	1
P04196	F1SFI5	PIG Uncharacterized protein HRG	1.84	1.54	1.62	-0.02	0.59	0.75	0.73	1.00	9
P02679	F1RX35	PIG Uncharacterized protein LOC100627396	-1.73	-0.84	2.77	4.61	0.64	0.97	0.13	0.00	15
P02748	F1SMJ6	PIG Uncharacterized protein (Fragment) C9	1.47	0.95	0.32	1.9	0.77	0.95	1.00	0.66	1
	F1STG7	PIG Uncharacterized protein (Fragment) IL1R1	0.86	0.7	4.05	-1.14	0.99	0.98	0.00	0.98	1
	P02751	HUMAN Fibronectin	2.06	1.64	-0.18	0.48	0.45	0.71	1.00	1.02	1
	P62070	HUMAN Ras-related protein R-Ras2	-0.01	1.01	0.64	2.3	1.00	0.95	1.01	0.39	1
	F1SFI7	PIG Alpha-2-HS-glycoprotein (Fragment)	2.03	0.21	1.45	0.16	0.47	1.00	0.81	1.00	1
	F1S1A9	PIG Uncharacterized protein APOA2	0.48	0.03	0.96	2.27	1.00	1.00	0.96	0.41	1
	P14460	PIG Fibrinogen alpha chain (Fragment)	-1.11	-1.03	2.32	3.43	0.92	0.95	0.31	0.02	2
	H9LBP0	PIG Serpin peptidase inhibitor clade D member 1 (Fragment)	2.35	1.11	-0.07	0.05	0.32	0.92	1.00	1.00	2
	Q19AZ8	PIG Prothrombin	0.55	0.14	1.09	1.45	1.00	0.99	0.93	0.88	3

	A0PFK5	PIG Capping protein (Actin filament) muscle Z-line, alpha 1	0.53	0.51	0.91	1.11	1.00	1.00	0.96	0.99	4
	O02668	PIG Inter-alpha-trypsin inhibitor heavy chain H2	0.81	0.56	1.24	0.37	0.99	0.99	0.89	1.01	12
Q02750	I3L5D4	PIG Uncharacterized protein MAP2K2	0.25	0.76	0.93	0.93	1.00	0.97	0.96	1.02	2
	Q0PM28	PIG Pigment epithelium-derived factor	0.73	1.27	0.19	0.17	1.00	0.87	1.01	1.00	4
	P28768	PIG Superoxide dismutase [Mn], mitochondrial (Fragment)	0.21	-0.35	1.62	0.55	1.00	1.00	0.74	1.02	8
	F1SH94	PIG Uncharacterized protein ITIH3	0.68	-0.35	0.69	0.94	1.01	1.00	0.99	1.02	3
P61978	I3LQS0	PIG Uncharacterized protein HNRNPK	0.73	1	1.11	-0.92	1.00	0.95	0.93	1.03	9
	Q2MJK3	PIG GTPase NRas	0.61	1.16	0.54	-0.5	1.00	0.91	1.01	1.03	1
P02749	I3LGN5	PIG Uncharacterized protein (Fragment)	-0.56	-0.02	1.27	0.96	1.00	1.00	0.88	1.03	1
	I3LIZ1	PIG Uncharacterized protein (Fragment) MAPK12	0.73	0.42	0.42	0.03	1.01	1.00	1.00	1.00	1
	F1SA28	PIG Uncharacterized protein ECSIT	0.14	0.94	0.51	-0.06	1.01	0.96	1.00	1.00	1
	P50447	PIG Alpha-1-antitrypsin	0.79	0.1	1.94	-1.4	1.00	1.00	0.54	0.91	6
P01116-2	I3LCQ9	PIG Uncharacterized protein KRAS	-0.01	-0.03	0.37	1	1.00	1.00	1.00	1.02	1
P05546	F1RKY2	PIG Uncharacterized protein SERPIND1	0.32	-0.45	0.41	0.71	1.00	0.99	1.00	1.03	5
P10301	F1RHW4	PIG Uncharacterized protein RRAS	0.24	0.74	0.44	-0.46	1.00	0.97	1.00	1.02	6
	P61978	HUMAN Heterogeneous nuclear ribonucleoprotein K	0.31	0.61	0.09	-0.17	1.00	0.99	1.00	1.00	2
	H0YA55	HUMAN Serum albumin (Fragment)	0.61	-0.26	1.29	-0.81	1.00	1.01	0.88	1.03	1
	P04179	HUMAN Superoxide dismutase [Mn], mitochondrial	0.26	-0.63	0.8	0.29	1.00	0.99	0.98	1.01	1
	P06867	PIG Plasminogen	-1.06	-2.01	1.62	2.11	0.94	0.49	0.74	0.52	7
	P02768	HUMAN Serum albumin	0.65	-1.15	3.52	-2.54	1.01	0.91	0.02	0.25	1
	M3V839	PIG Mitogen-activated protein kinase 3	1.01	0.13	-0.71	0.05	0.95	0.99	0.99	1.00	1
	P05231	HUMAN Interleukin-6	-0.95	-1.14	0.31	2.15	0.97	0.91	1.00	0.49	1
P30519	F1RK58	PIG Uncharacterized protein HMOX2	0.48	0	-0.5	0.21	1.00	1.00	1.00	1.00	3
P45985	F1SS51	PIG Uncharacterized protein (Fragment) MAP2K4	-0.1	-1.73	2.16	-0.26	1.01	0.65	0.39	1.01	1
	P50390	PIG Transthyretin	1.11	-1.6	1.82	-1.32	0.92	0.73	0.63	0.93	4
	P01025	PIG Complement C3	-0.16	-2.19	1.43	0.92	1.01	0.39	0.82	1.02	48
	P08835	PIG Serum albumin	0.2	-1.49	3.17	-1.9	1.00	0.78	0.05	0.66	24
	Q9N1U3	PIG Glucocorticoid receptor	-0.43	-0.27	-0.88	1.2	1.00	1.00	0.97	0.97	1
	M4QER0	PIG Glucocorticoid receptor variant gamma	-0.03	-0.62	-0.54	0.79	1.00	0.99	1.01	1.03	1
P02549	F1RR78	PIG Uncharacterized protein LOC100049693	-0.65	-0.51	-0.49	1.02	1.00	1.00	1.00	1.01	149
	P62993	HUMAN Growth factor receptor-bound protein 2	-0.01	-0.01	-0.29	-0.56	1.00	1.00	1.00	1.03	3
	P29700	PIG Alpha-2-HS-glycoprotein (Fragment)	0.38	-1.04	-0.08	-0.36	0.99	0.95	1.00	1.01	2

	A9QT41	PIG Inhibitor of kappa light polypeptide gene enhancer in B-cells kinase gamma	-0.43	-0.06	-0.84	-0.15	1.00	1.00	0.97	1.00	1
	P02675	HUMAN Fibrinogen beta chain	0.5	-0.68	-0.56	-1.15	1.00	0.98	1.01	0.98	1
	I3LTB8	PIG Uncharacterized protein	-1.55	-2.38	0.46	1	0.73	0.30	1.01	1.02	1
	G9BWQ1	PIG V-akt murine thymoma viral oncogene-like 1	0.1	-1.01	-1.43	-0.32	1.00	0.95	0.82	1.02	2
	A0PFK4	PIG F-actin capping protein subunit beta 2	-1.38	-0.72	0.13	-0.96	0.82	0.97	1.00	1.03	1
	P00451	HUMAN Coagulation factor VIII	-1.62	-1.1	-0.96	0.72	0.70	0.92	0.96	1.03	1
	F1RUN2	PIG Serum albumin	-0.44	-1.3	0.31	-1.63	1.00	0.86	1.00	0.80	1
	F1SB81	PIG Plasminogen	-1.17	-2.07	-1.13	1.02	0.90	0.46	0.92	1.01	1
P02751	F1SS24	PIG Uncharacterized protein FN1	-0.5	-1.59	-1.05	-0.23	1.00	0.73	0.94	1.01	26
	H0YCB4	HUMAN Transcription factor p65 (Fragment)	-0.76	-0.74	-1.14	-1.09	1.01	0.97	0.92	0.99	1
Q9NPH3-5	I3LR80	PIG Uncharacterized protein IL1RAP	-0.7	-0.78	-1.26	-1.02	1.00	0.97	0.89	1.01	1
	I3LVK2	PIG Uncharacterized protein (Fragment) MAPK3	-0.62	-1.08	-1.24	-1.54	1.00	0.93	0.90	0.85	1
	P18648	PIG Apolipoprotein A-I	0.79	-2.36	-1.05	-2.44	1.00	0.31	0.94	0.29	25

Human equivalent Ac. No.	Accession No.	Adhesion of blood cells<br>Protein	Zq<br>120	1	4	7	FDR<br>120	1	4	7	No. peptides
-	I3LQ17	PIG Uncharacterized protein	1.23	0.42	4.23	2.57	0.89	1.00	0.00	0.24	23
-	I3LQR9	PIG Fibrinogen alpha chain	-1.01	0.06	2.75	5.27	0.95	1.00	0.14	0.00	9
-	F1RX36	PIG Fibrinogen alpha chain	-1.36	-0.25	2.97	5.7	0.83	1.01	0.08	0.00	4
-	K7GR72	PIG Uncharacterized protein SLC4A1	1.17	1.96	2.65	1.01	0.90	0.52	0.17	1.01	4
-	P06702	HUMAN Protein S100-A9	0.67	-0.38	0.52	5.49	1.01	1.00	1.01	0.00	1
-	K9J6H8	PIG Alpha-2-macroglobulin	0.66	0.44	3.51	0.97	1.00	0.99	0.02	1.02	1
-	I3LR17	PIG Uncharacterized protein COR1A	1.29	1.82	0.95	1.45	0.86	0.59	0.96	0.88	1
-	Q95242	PIG Platelet endothelial cell adhesion molecule	0.98	1.66	1	0.8	0.96	0.70	0.96	1.03	5
-	P56199	HUMAN Integrin alpha-1	0.67	0.07	1.43	2.18	1.01	1.00	0.82	0.47	1
-	Q06AU2	PIG Ras-related protein Rap-2a	1.45	1.99	1.12	-0.3	0.79	0.50	0.92	1.01	1
-	P27917	PIG Apolipoprotein C-III	0.5	0.09	3.04	0.58	1.00	1.00	0.07	1.02	4
-	P02751	HUMAN Fibronectin	2.06	1.64	-0.18	0.48	0.45	0.71	1.00	1.02	1
-	P14460	PIG Fibrinogen alpha chain (Fragment)	-1.11	-1.03	2.32	3.43	0.92	0.95	0.31	0.02	2
-	A7WLI1	PIG CD81	0.47	1.08	0.33	1.73	1.00	0.93	1.00	0.75	1
-	P20701	HUMAN Integrin alpha-L	0.49	0.5	1.12	1.37	1.00	1.00	0.92	0.91	1

P15311	F1SB42	PIG Uncharacterized protein (Fragment) EZR	-0.04	0.27	1.18	1.87	1.00	1.01	0.91	0.68	11
-	Q19AZ8	PIG Prothrombin	0.55	0.14	1.09	1.45	1.00	0.99	0.93	0.88	3
-	Q13464	HUMAN Rho-associated protein kinase 1	0.61	0.35	2.55	-0.43	1.00	1.00	0.20	1.02	1
-	P18650	PIG Apolipoprotein E	0.62	0.06	1.31	1.02	1.00	1.00	0.87	1.01	4
-	Q9GKE8	PIG Leukocyte surface antigen CD47	1.49	0.45	0.32	0.69	0.76	0.99	1.00	1.03	1
-	P19619	PIG Annexin A1	0.66	0.64	0.58	1.05	1.01	0.98	1.01	1.00	10
-	Q9BDY7	PIG Angiopoietin-2	0.98	0.93	1.1	-0.15	0.96	0.96	0.93	1.00	1
-	F1RTN3	PIG Moesin	0.01	0.68	0.62	1.23	1.00	0.98	1.01	0.96	1
-	Q29030	PIG Kit ligand	-0.32	-0.2	1.7	1.31	1.00	1.00	0.69	0.93	1
-	Q3HUX1	PIG Fatty acid translocase/CD36	-0.62	0.26	1.29	1.51	1.00	1.01	0.88	0.87	9
-	Q9GLP0	PIG Integrin beta-1	0.72	1.14	0.1	0.45	1.00	0.91	1.00	1.02	8
-	A2SW51	PIG Monocyte differentiation antigen CD14	0.93	0.85	0.49	-0.09	0.97	0.97	1.00	1.00	1
-	K7GS51	PIG Uncharacterized protein (Fragment) ITGA1	0.87	0.57	0.42	0.3	0.99	0.99	1.00	1.01	2
-	P30533	HUMAN Alpha-2-macroglobulin receptor-associated protein	0.78	-0.85	1.36	0.7	1.00	0.97	0.84	1.03	1
-	B2ZI35	PIG F11 receptor	-0.1	0.3	1.2	0.58	1.01	1.00	0.90	1.03	1
-	P14632	PIG Lactotransferrin	0.71	0.86	-0.05	0.19	1.00	0.97	1.00	1.01	2
P02749	I3LGN5	PIG Uncharacterized protein (Fragment)	-0.56	-0.02	1.27	0.96	1.00	1.00	0.88	1.03	1
-	Q4A3R3	PIG Deleted in malignant brain tumors 1 protein	-1.06	0.22	1.49	0.96	0.94	1.00	0.79	1.03	1
-	P26042	PIG Moesin	-0.41	-0.03	0.46	1.53	1.00	1.00	1.01	0.86	34
Q96S97	F1RNK3	PIG Uncharacterized protein LOC100519316	0.02	0.9	-0.27	0.37	1.00	0.97	1.00	1.01	3
-	J3KP07	HUMAN Growth arrest-specific protein 6	-0.95	-0.26	1.28	0.76	0.97	1.01	0.88	1.03	1
-	P11215	HUMAN Integrin alpha-M	-0.18	-0.41	0.5	0.9	1.01	0.99	1.00	1.03	1
-	K7ZJP7	PIG IgM heavy chain constant region (Fragment)	0.05	-1.07	2.56	-0.74	1.00	0.94	0.20	1.03	6
-	P05231	HUMAN Interleukin-6	-0.95	-1.14	0.31	2.15	0.97	0.91	1.00	0.49	1
-	P01025	PIG Complement C3	-0.16	-2.19	1.43	0.92	1.01	0.39	0.82	1.02	48
-	F1S0A2	PIG Peptidyl-prolyl cis-trans isomerase	0.02	0.11	-0.33	0.2	1.00	1.00	1.00	1.00	1
-	P16284	HUMAN Platelet endothelial cell adhesion molecule	0.75	-0.71	0.04	-0.16	1.01	0.97	1.00	1.00	1
-	F1RYZ1	PIG Uncharacterized protein CD151	-0.95	-0.24	0.73	0.31	0.96	1.00	0.99	1.02	3
-	O14746	HUMAN Telomerase reverse transcriptase	-0.14	-0.54	0.97	-0.47	1.01	0.99	0.96	1.02	1
-	Q7L5Y9	HUMAN Macrophage erythroblast attacher	0.7	0.82	-1.33	-0.39	1.00	0.97	0.85	1.01	1
-	F1S1G8	PIG Amine oxidase	-0.01	-0.07	0.21	-0.47	1.00	1.00	1.00	1.02	9
-	P15311	HUMAN Ezrin	-0.4	-1.15	-0.09	1.1	1.00	0.91	1.00	1.00	5

P23284	I3LUC8	PIG Uncharacterized protein LOC100154783	-0.56	-0.42	-0.19	0.57	1.00	1.00	1.01	1.03	8
P02549	F1RR78	PIG Uncharacterized protein LOC100049693	-0.65	-0.51	-0.49	1.02	1.00	1.00	1.00	1.01	149
P63000	I3LFI0	PIG Uncharacterized protein (Fragment) RAC1	-0.68	-0.52	-0.07	0.58	1.01	1.00	1.00	1.03	2
-	P43121	HUMAN Cell surface glycoprotein MUC18	-1.08	0.22	-0.54	0.64	0.93	1.00	1.01	1.03	1
-	F1S2E3	PIG Peptidyl-prolyl cis-trans isomerase	-0.69	-0.39	-0.05	0.35	1.01	1.00	1.00	1.01	8
-	K9IVW2	PIG Integrin alpha-X	-0.77	-0.61	0.15	0.29	1.00	0.99	1.00	1.01	1
-	P14287	PIG Osteopontin	3.33	-0.25	0.01	-4.24	0.04	1.00	1.00	0.00	1
-	F1S0J0	PIG Uncharacterized protein CD55	0	-0.3	-1.02	-0.33	1.00	1.00	0.95	1.02	1
Q12846	F1RIR2	PIG Uncharacterized protein STX4	-0.84	-0.62	0.17	-0.59	0.99	0.99	1.00	1.03	1
-	P82460	PIG Thioredoxin	0.16	-0.68	-0.19	-1.46	1.01	0.98	1.01	0.88	3
-	O15230	HUMAN Laminin subunit alpha-5	-1.31	-1.1	0.25	-0.19	0.85	0.93	1.00	1.01	5
-	I3LTB8	PIG Uncharacterized protein	-1.55	-2.38	0.46	1	0.73	0.30	1.01	1.02	1
-	K9J4V8	PIG ICOS ligand	-1.49	-0.71	-0.45	0.02	0.76	0.97	1.01	1.00	1
Q8NCN5	F1S430	PIG Uncharacterized protein (Fragment) LOC100525578	-0.2	-0.22	-1.33	-1.2	1.00	1.00	0.86	0.97	3
-	P08514	HUMAN Integrin alpha-IIb	-1.17	-0.66	-1.4	0.28	0.90	0.98	0.83	1.01	1
P02751	F1SS24	PIG Uncharacterized protein FN1	-0.5	-1.59	-1.05	-0.23	1.00	0.73	0.94	1.01	26
-	H0YCB4	HUMAN Transcription factor p65 (Fragment)	-0.76	-0.74	-1.14	-1.09	1.01	0.97	0.92	0.99	1
P21980	F1SDX6	PIG Uncharacterized protein TGM2	-1.37	-1.62	-1.15	-0.06	0.83	0.72	0.92	1.01	25
-	O46409	PIG Apolipoprotein A-IV	1.82	-0.79	-3.83	-1.77	0.59	0.97	0.01	0.73	6
-	P52552	PIG Peroxiredoxin-2 (Fragment)	0.39	-0.61	-0.77	-3.8	1.00	0.99	0.98	0.01	7
-	Q9GLP2	PIG Vitamin K-dependent protein C	-1.77	-3.09	-3.38	-2.07	0.62	0.07	0.03	0.55	1

Human equivalent Ac. No.	Accession No.	Response to wounding<br>Protein	Zq 120	1	4	7	FDR 120	1	4	7	No. peptides
-	P14477	PIG Fibrinogen beta chain (Fragment)	0.24	1.08	3.32	5.04	1.00	0.93	0.03	0.00	1
-	P00747	HUMAN Plasminogen	2.18	2	2.1	1.78	0.39	0.50	0.44	0.73	1
P02675	I3L651	PIG Uncharacterized protein	-0.75	0.09	3.13	4.68	1.01	1.00	0.05	0.00	20
-	I3LQR9	PIG Fibrinogen alpha chain	-1.01	0.06	2.75	5.27	0.95	1.00	0.14	0.00	9
-	F1RX36	PIG Fibrinogen alpha chain	-1.36	-0.25	2.97	5.7	0.83	1.01	0.08	0.00	4
-	Q1KYT0	PIG Beta-enolase	0.49	0.72	3.12	0.16	1.00	0.97	0.06	1.00	32
-	P02751	HUMAN Fibronectin	2.06	1.64	-0.18	0.48	0.45	0.71	1.00	1.02	1

-	P14460	PIG Fibrinogen alpha chain (Fragment)	-1.11	-1.03	2.32	3.43	0.92	0.95	0.31	0.02	2
Q9UBX5	F1SD87	PIG Uncharacterized protein (Fragment) FBLN5	0.71	0.68	0.87	1.2	1.00	0.98	0.97	0.97	4
-	P37176	PIG Endoglin	1.25	1.99	0.88	-0.77	0.88	0.50	0.97	1.03	2
-	Q19AZ8	PIG Prothrombin	0.55	0.14	1.09	1.45	1.00	0.99	0.93	0.88	3
-	Q9GLP1	PIG Coagulation factor V	0.74	0.56	0.61	0.54	1.01	0.99	1.01	1.03	1
Q14344	F1RV20	PIG Uncharacterized protein GNA13	0.61	0.84	-0.65	-0.12	1.00	0.97	1.00	1.00	1
-	P06867	PIG Plasminogen	-1.06	-2.01	1.62	2.11	0.94	0.49	0.74	0.52	7
Q13310	F1RPL9	PIG Uncharacterized protein LOC100525442	0.62	0.58	-0.51	-0.12	1.00	0.99	1.00	1.00	1
-	I3L8C1	PIG Uncharacterized protein ELK3	1.87	-0.26	-0.92	-0.22	0.56	1.01	0.96	1.01	1
-	Q8MJ14	PIG Glutathione peroxidase 1	-0.03	0.22	-0.26	0.31	1.00	1.00	1.00	1.02	4
P0CB38	F1SV06	PIG Uncharacterized protein PABPC4	-0.21	0.17	0.17	0.06	1.00	1.00	1.00	1.01	12
-	P16293	PIG Coagulation factor IX (Fragment)	0.64	0.34	-0.03	-0.76	1.00	1.00	1.00	1.03	2
O75923	F1SLE7	PIG Uncharacterized protein LOC100522732	0.95	0.88	-0.89	-1.29	0.97	0.97	0.97	0.94	9
-	P20305	PIG Gelsolin (Fragment)	-0.16	-0.77	0.34	-0.21	1.01	0.97	1.00	1.00	19
-	P02675	HUMAN Fibrinogen beta chain	0.5	-0.68	-0.56	-1.15	1.00	0.98	1.01	0.98	1
-	P00451	HUMAN Coagulation factor VIII	-1.62	-1.1	-0.96	0.72	0.70	0.92	0.96	1.03	1
-	Q700R3	PIG Peroxisome proliferative activated receptor delta (Fragment)	-0.83	-0.57	-0.99	-0.59	0.99	0.99	0.96	1.03	1
-	F1SB81	PIG Plasminogen	-1.17	-2.07	-1.13	1.02	0.90	0.46	0.92	1.01	1
P02751	F1SS24	PIG Uncharacterized protein FN1	-0.5	-1.59	-1.05	-0.23	1.00	0.73	0.94	1.01	26
-	K7GQL2	PIG Coagulation factor XIII, A1 polypeptide	-0.11	-0.47	-1.36	-2.81	1.01	0.99	0.84	0.14	1
-	Q9GLP2	PIG Vitamin K-dependent protein C	-1.77	-3.09	-3.38	-2.07	0.62	0.07	0.03	0.55	1

Supplementary Table S8. Protein expression changes in the functionally annotated categories within cluster d.

Renal toxicity biomarker panel

Human equivalent Ac. No.	Accession No.	Protein	Zq 120	Zq 1	Zq 4	Zq 7	FDR 120	FDR 1	FDR 4	FDR 7	No. peptides
-	Q06AA3	PIG Regucalcin	3.5	4.2	2.69	-1.06	0.03	0.00	0.15	1.00	1
-	I3LQR9	PIG Fibrinogen alpha chain	-1.01	0.06	2.75	5.27	0.95	1.00	0.14	0.00	9
-	F1RX36	PIG Fibrinogen alpha chain	-1.36	-0.25	2.97	5.7	0.83	1.01	0.08	0.00	4
-	P80031	PIG Glutathione S-transferase P	1.3	2.52	0.92	-0.91	0.85	0.23	0.96	1.03	6
-	P14460	PIG Fibrinogen alpha chain (Fragment)	-1.11	-1.03	2.32	3.43	0.92	0.95	0.31	0.02	2
-	Q5S1U1	PIG Heat shock protein beta-1	-0.04	0.9	1.39	0.75	1.00	0.97	0.83	1.03	15
-	O02705	PIG Heat shock protein HSP 90-alpha	-1.06	-0.09	0.37	1.28	0.94	1.00	1.00	0.94	40
-	F1SKD6	PIG Uncharacterized protein SLC16A7	-0.8	-0.71	-0.71	1.44	1.00	0.97	0.99	0.88	2
-	P14287	PIG Osteopontin	3.33	-0.25	0.01	-4.24	0.04	1.00	1.00	0.00	1

Acute renal failure panel

Human equivalent Ac. No.	Accession No.	Protein	Zq 120	Zq 1	Zq 4	Zq 7	FDR 120	FDR 1	FDR 4	FDR 7	No. peptides
-	P14477	PIG Fibrinogen beta chain (Fragment)	0.24	1.08	3.32	5.04	1.00	0.93	0.03	0.00	1
P02452	I3LJX2	PIG Uncharacterized protein	2.3	2.53	1.24	2.27	0.34	0.22	0.90	0.41	2
-	I3LQ17	PIG Uncharacterized protein	1.23	0.42	4.23	2.57	0.89	1.00	0.00	0.24	23
P02675	I3L651	PIG Uncharacterized protein	-0.75	0.09	3.13	4.68	1.01	1.00	0.05	0.00	20
P02452	I3LUM2	PIG Uncharacterized protein	1.82	2.35	1.88	1.74	0.59	0.31	0.58	0.75	4
Q01995	F1SJS8	PIG Uncharacterized protein TAGLN	-0.6	0.87	4.5	1.93	1.00	0.97	0.00	0.64	14
-	K9J6H8	PIG Alpha-2-macroglobulin	0.66	0.44	3.51	0.97	1.00	0.99	0.02	1.02	1
-	P00339	PIG L-lactate dehydrogenase A chain	0.68	0.47	2.73	1.89	1.01	0.99	0.15	0.67	14
-	P02461	HUMAN Collagen alpha-1(III) chain	0.42	1.75	0.47	1.27	1.00	0.63	1.01	0.95	2
P02461	F1RYI8	PIG Uncharacterized protein COL3A1	0.7	0.8	0.67	1.6	1.00	0.97	1.00	0.82	2
-	P02452	HUMAN Collagen alpha-1(I) chain	0.41	1.82	-0.15	1.27	1.00	0.60	1.00	0.95	1
-	P19619	PIG Annexin A1	0.66	0.64	0.58	1.05	1.01	0.98	1.01	1.00	10

-	A2SW51	PIG Monocyte differentiation antigen CD14	0.93	0.85	0.49	-0.09	0.97	0.97	1.00	1.00	1
-	Q6PQZ1	PIG Aquaporin-1	0.6	1.36	0.34	1.16	1.00	0.84	1.00	0.98	2
-	P05231	HUMAN Interleukin-6	-0.95	-1.14	0.31	2.15	0.97	0.91	1.00	0.49	1
P07305	I3LNZ2	PIG Uncharacterized protein LOC100510904	-0.56	0.64	1.56	1.76	1.00	0.98	0.76	0.75	5
-	Q99988	HUMAN Growth/differentiation factor 15	1.15	1.2	0.25	-0.4	0.90	0.90	1.00	1.02	1
-	F1S7W0	PIG Uncharacterized protein (Fragment) AKAP12	1.29	1.08	-0.16	-0.2	0.86	0.93	1.00	1.01	3
-	P19620	PIG Annexin A2	0.08	0.35	0.55	0.52	1.00	1.00	1.00	1.03	24
-	B1PK17	PIG Aspartoacylase	0.53	-0.47	-0.19	0.59	1.00	0.99	1.01	1.03	1
-	F1SKD6	PIG Uncharacterized protein SLC16A7	-0.8	-0.71	-0.71	1.44	1.00	0.97	0.99	0.88	2
-	Q2VTP6	PIG Peptidyl-prolyl cis-trans isomerase	-0.14	-0.47	-0.25	0.06	1.01	0.99	1.00	1.00	2
-	P07437	HUMAN Tubulin beta chain	0.99	0.44	-1.16	-1.27	0.96	0.99	0.92	0.95	4
-	P02675	HUMAN Fibrinogen beta chain	0.5	-0.68	-0.56	-1.15	1.00	0.98	1.01	0.98	1
P55042	I3L820	PIG Uncharacterized protein (Fragment) RRAD	-2.3	-0.1	-1.01	-1.24	0.34	1.00	0.96	0.96	2

Human equivalent Ac. No.	Accession No.	**Chromatin organization**<br>Protein	Zq 120	Zq 1	Zq 4	Zq 7	FDR 120	FDR 1	FDR 4	FDR 7	No. peptides
-	P06348	PIG Histone H1t	-0.86	-0.01	1.31	3.09	0.99	1.00	0.87	0.07	4
-	P12682	PIG High mobility group protein B1	-0.73	0.08	0.21	1.01	1.00	1.00	1.00	1.01	6
-	P17741	PIG High mobility group protein B2	0.29	0.89	1.35	0.8	1.00	0.97	0.84	1.03	4
-	P80272	PIG Non-histone chromosomal protein HMG-17	-0.35	-0.84	-0.75	5.93	1.00	0.97	0.99	0.00	4
-	Q8IUE6	HUMAN Histone H2A type 2-B	-0.13	0.09	0.74	0.02	1.01	1.00	0.99	1.00	1
-	Q9H2F5	HUMAN Enhancer of polycomb homolog 1	-1.44	-1.87	-1.47	0.58	0.79	0.57	0.80	1.02	1
-	Q9UQL6	HUMAN Histone deacetylase 5	0.09	-0.33	-0.46	-0.92	1.01	1.01	1.01	1.03	1
P09429	A7E1T5	PIG Putative uncharacterized protein (Fragment)	0.91	0.86	0.27	1.15	0.98	0.97	1.00	0.98	1
-	B6CVL5	PIG High mobility group AT-hook 1 transcript variant 2	0.74	1.32	2.29	4.78	1.00	0.86	0.32	0.00	1
P23284	I3LUC8	PIG Uncharacterized protein LOC100154783	-0.56	-0.42	-0.19	0.57	1.00	1.00	1.01	1.03	8
-	F1S0Z4	PIG Histone H2A (Fragment)	-1.32	-0.8	-0.08	0.65	0.84	0.97	1.00	1.03	2
-	P36968	PIG Phospholipid hydroperoxide glutathione peroxidase, mitochondrial	-1.31	0.47	-1.54	0.76	0.85	0.99	0.77	1.03	4
Q8TDV2	F1SPG1	PIG Uncharacterized protein H1FX	0.01	-0.39	0.89	1.5	1.00	1.00	0.96	0.87	6
P16401	I3L6Q5	PIG Uncharacterized protein HIST1H1B	0.19	0.75	2.31	3.78	1.01	0.97	0.32	0.01	4
-	I3L7T6	PIG Histone H2A	-0.62	-0.08	-0.78	1.85	1.00	1.00	0.98	0.69	1

Human equivalent Ac. No.	Accession No.	Protein	Zq 120	Zq 1	Zq 4	Zq 7	FDR 120	FDR 1	FDR 4	FDR 7	No. peptides
-	I3LAQ1	PIG Histone H2B	-2.19	-0.48	0.76	0.27	0.39	0.99	0.98	1.01	2
-	I3LQZ2	PIG Histone H2A (Fragment)	-1.44	-0.82	-1.41	0.59	0.79	0.97	0.83	1.03	2
-	Q4TTS4	PIG Histone H1.2-like protein	-0.98	-0.95	-0.34	0.95	0.96	0.96	1.00	1.03	4

Flux of Ca2+

Human equivalent Ac. No.	Accession No.	Protein	Zq 120	Zq 1	Zq 4	Zq 7	FDR 120	FDR 1	FDR 4	FDR 7	No. peptides
P98161	F1RFB1	PIG Uncharacterized protein PKD1	1.06	1.76	1.89	0.81	0.94	0.63	0.58	1.03	2
-	Q95242	PIG Platelet endothelial cell adhesion molecule	0.98	1.66	1	0.8	0.96	0.70	0.96	1.03	5
-	Q19AZ8	PIG Prothrombin	0.55	0.14	1.09	1.45	1.00	0.99	0.93	0.88	3
-	P19619	PIG Annexin A1	0.66	0.64	0.58	1.05	1.01	0.98	1.01	1.00	10
-	P62936	PIG Peptidyl-prolyl cis-trans isomerase A	-0.13	0.77	0.61	0.2	1.01	0.97	1.01	1.01	12
-	D2KMN4	PIG Glial cell-line derived neurotrophic factor (Fragment)	-0.89	-0.26	0.52	1.22	0.98	1.01	1.01	0.96	1
-	P01025	PIG Complement C3	-0.16	-2.19	1.43	0.92	1.01	0.39	0.82	1.02	48
-	F1S0A2	PIG Peptidyl-prolyl cis-trans isomerase	0.02	0.11	-0.33	0.2	1.00	1.00	1.00	1.00	1
-	P16284	HUMAN Platelet endothelial cell adhesion molecule	0.75	-0.71	0.04	-0.16	1.01	0.97	1.00	1.00	1
-	M3TYK5	PIG ATPase, Ca++ transporting, plasma membrane 4 tv2	0.08	-0.21	-0.13	-0.2	1.00	1.00	1.00	1.01	3
P23284	I3LUC8	PIG Uncharacterized protein LOC100154783	-0.56	-0.42	-0.19	0.57	1.00	1.00	1.01	1.03	8
P02549	F1RR78	PIG Uncharacterized protein LOC100049693	-0.65	-0.51	-0.49	1.02	1.00	1.00	1.00	1.01	149
Q9UNN5	K7GLR2	PIG Uncharacterized protein (Fragment) FAF1	-0.88	-1	-0.27	1.48	0.99	0.95	1.00	0.88	1
-	F1S2E3	PIG Peptidyl-prolyl cis-trans isomerase	-0.69	-0.39	-0.05	0.35	1.01	1.00	1.00	1.01	8
-	Q92736	HUMAN Ryanodine receptor 2	-0.62	-0.68	-0.45	0.42	1.00	0.98	1.01	1.02	5
-	I3L9F1	PIG Uncharacterized protein (Fragment) BSG	-0.39	-0.06	-0.47	-0.5	0.99	1.00	1.01	1.03	7
-	Q6RVA9	PIG Caveolin-1	-1.46	-0.38	-0.73	1.09	0.77	1.00	0.99	0.99	8
-	P18509	HUMAN Pituitary adenylate cyclase-activating polypeptide	-0.75	-0.73	-0.54	-0.26	1.00	0.97	1.01	1.01	1
-	I3LTB8	PIG Uncharacterized protein	-1.55	-2.38	0.46	1	0.73	0.30	1.01	1.02	1
	O75185	HUMAN Calcium-transporting ATPase type 2C member 2	-1.33	-1	-0.92	-0.37	0.84	0.95	0.96	1.01	1

Ribosome

Human equivalent Ac. No.	Accession No.	Protein	Zq 120	Zq 1	Zq 4	Zq 7	FDR 120	FDR 1	FDR 4	FDR 7	No. peptides
-	F1SUM7	PIG 40S ribosomal protein S3 (Fragment)	0.65	1.19	1.32	1.5	1.01	0.90	0.86	0.87	7

-	P62750	HUMAN 60S ribosomal protein L23a	0.51	0.84	0.84	2.26	1.00	0.97	0.97	0.41	4
-	F2Z5F5	PIG 40S ribosomal protein S8 (Fragment)	-0.03	0.99	1.51	1.86	1.00	0.95	0.79	0.69	5
-	F1SML8	PIG 40S ribosomal protein S6	1.48	0.73	2.57	-0.68	0.77	0.97	0.20	1.03	1
-	Q95281	PIG 60S ribosomal protein L29	0.17	0.42	1.02	2.26	1.01	1.00	0.95	0.41	4
P46783	F1RZ28	PIG Uncharacterized protein RPS10	0.39	0.84	1.27	1.35	1.00	0.97	0.88	0.91	7
P62899	F1SRB7	PIG Uncharacterized protein (Fragment) LOC100624537	0.45	0.89	1.23	0.86	1.00	0.97	0.90	1.03	1
-	Q29198	PIG Ribosomal protein S6 (Fragment)	0.75	0.7	0.49	1.39	1.01	0.97	1.00	0.91	3
-	P62857	HUMAN 40S ribosomal protein S28	0.57	0.64	0.82	0.95	1.00	0.98	0.97	1.02	1
P18621	I3LT81	PIG Uncharacterized protein RPL17	-0.37	0.63	1.09	1.6	1.00	0.99	0.93	0.82	7
P83881	I3LHT5	PIG Uncharacterized protein	-0.23	0.06	1.41	1.68	1.00	1.00	0.82	0.78	1
-	P32969	HUMAN 60S ribosomal protein L9	0.36	0.65	0.9	0.57	0.99	0.98	0.96	1.03	5
-	P67985	PIG 60S ribosomal protein L22	0.03	0.53	0.91	1	1.00	1.00	0.96	1.02	3
-	P49666	PIG 60S ribosomal protein L21 (Fragment)	-0.28	0.66	1.71	0.34	1.00	0.98	0.69	1.01	3
-	Q29223	PIG 60S ribosomal protein L34	1.23	0.01	0.44	0.69	0.89	1.00	1.00	1.03	2
-	P62280	HUMAN 40S ribosomal protein S11	-0.5	0.47	1.11	1.06	1.00	0.99	0.92	1.00	6
-	F1RYZ0	PIG 60S acidic ribosomal protein P2	2.08	1.6	0.03	-1.68	0.44	0.73	1.00	0.78	2
-	Q29375	PIG 60S ribosomal protein L7a (Fragment)	-0.68	0.16	0.77	1.74	1.01	1.00	0.98	0.75	3
-	Q29315	PIG 60S acidic ribosomal protein P2	2.3	1.14	-0.1	-1.48	0.34	0.91	1.00	0.88	3
P62899	F1SH66	PIG Uncharacterized protein LOC100625352	-0.06	-0.07	1.02	0.95	1.00	1.00	0.95	1.03	1
-	I3LSD3	PIG 60S ribosomal protein L13	-0.36	0.22	0.07	1.88	0.99	1.00	1.00	0.68	9
-	I3L6F1	PIG 60S ribosomal protein L18	-0.64	1.31	0.01	1.13	1.00	0.86	1.00	0.98	1
-	B6V8C8	PIG 40S ribosomal protein S3a	0.07	0.19	0.61	0.78	1.00	1.00	1.01	1.03	9
-	F2Z514	PIG 40S ribosomal protein S27 (Fragment)	0.4	0.1	0.12	1.01	1.00	1.00	1.00	1.01	1
P83731	F2Z5Q2	PIG Uncharacterized protein (Fragment) RPL24	-0.39	0.62	0.22	1.18	1.00	0.99	1.00	0.98	4
-	Q29308	PIG 40S ribosomal protein S19 (Fragment)	-0.21	0.65	0.47	0.57	1.00	0.98	1.00	1.03	8
-	P23396	HUMAN 40S ribosomal protein S3	0.51	0.64	-0.03	0.31	1.00	0.98	1.00	1.02	6
-	P39019	HUMAN 40S ribosomal protein S19	0.68	0.81	-0.14	-0.05	1.01	0.97	1.00	1.00	1
-	I3LFL4	PIG 60S ribosomal protein L18 (Fragment)	-0.3	0.23	-0.3	1.62	1.00	1.00	1.00	0.80	2
-	Q5T7N0	HUMAN 60S ribosomal protein L5 (Fragment)	-0.39	0.44	0.02	1.17	1.00	0.99	1.00	0.98	1
-	P62899	HUMAN 60S ribosomal protein L31	-0.43	0.79	-0.38	1.22	1.00	0.97	1.00	0.96	2
-	P61353	HUMAN 60S ribosomal protein L27	-0.1	0.24	1	0.03	1.01	1.00	0.96	1.00	1
-	Q95307	PIG 60S ribosomal protein L13a (Fragment)	-0.41	-0.01	0.84	0.66	1.00	1.00	0.97	1.03	2

-	P46779	HUMAN 60S ribosomal protein L28	-1.02	-0.15	0.76	1.47	0.95	0.99	0.98	0.88	3
-	Q29323	PIG Ribosomal protein S5 (Fragment)	-0.18	-0.14	1.07	0.08	1.01	1.00	0.94	1.00	1
-	Q6QAP7	PIG 40S ribosomal protein S17	0.41	0.83	0.01	-0.44	1.00	0.97	1.00	1.02	3
-	Q29361	PIG 60S ribosomal protein L35	-0.23	0.01	-0.08	0.98	1.00	1.00	1.00	1.03	4
-	Q95L19	PIG Ribosomal protein L19 (Fragment)	-0.36	0.24	-0.01	0.78	0.99	1.00	1.00	1.03	3
-	Q95342	PIG 60S ribosomal protein L18 (Fragment)	-0.07	0.49	-0.73	0.91	1.00	1.00	0.99	1.03	3
-	P62244	HUMAN 40S ribosomal protein S15a	-0.01	0.42	-0.06	0.23	1.00	1.00	1.00	1.01	4
-	P62081	HUMAN 40S ribosomal protein S7	0.07	0.36	0.09	0.04	1.00	1.00	1.00	1.00	2
-	P62279	PIG 40S ribosomal protein S13 (Fragment)	0.06	0.4	0.16	-0.19	1.00	1.00	1.00	1.00	5
-	Q6QAS5	PIG 60S ribosomal protein L12 (Fragment)	-0.44	-0.25	0.65	0.47	1.00	1.00	1.00	1.02	6
-	I3LEX0	PIG 40S ribosomal protein S9 (Fragment)	-0.85	0.6	0.18	0.4	0.99	0.99	1.00	1.02	6
-	Q29195	PIG 60S ribosomal protein L10	-0.77	0.46	-0.09	0.53	1.01	0.99	1.00	1.03	6
-	Q29190	PIG Ribosomal protein S7 (Fragment)	0.18	-0.6	0.6	-0.06	1.01	0.99	1.01	1.01	2
-	P46778	HUMAN 60S ribosomal protein L21	0.01	0.73	0.16	-0.84	1.00	0.97	1.00	1.03	1
-	Q2YGT9	PIG 60S ribosomal protein L6	-0.54	-0.9	0.06	1.44	1.00	0.97	1.00	0.88	11
-	F1SJJ5	PIG 60S ribosomal protein L4	-0.53	-0.44	-0.47	1.49	1.00	0.99	1.01	0.87	7
-	P25398	HUMAN 40S ribosomal protein S12	0.05	0.29	-0.3	-0.04	1.00	1.00	1.00	1.00	2
-	P49171	PIG 40S ribosomal protein S26	-0.71	0.02	0.48	0.13	1.00	1.00	1.01	1.00	2
P62269	F1S4M2	PIG Uncharacterized protein (Fragment) LOC100738304	-0.27	-0.41	0.19	0.35	1.00	1.00	1.01	1.01	2
P83881	F1RT21	PIG Uncharacterized protein	-1.06	-0.11	0.11	0.83	0.94	1.00	1.00	1.03	1
-	P83881	HUMAN 60S ribosomal protein L36a	-1.5	-1.27	0.64	1.86	0.76	0.88	1.00	0.69	1
-	Q4GWZ2	PIG 40S ribosomal protein SA	0.08	0.7	-0.1	-1.02	1.01	0.97	1.00	1.01	5
-	Q29197	PIG 40S ribosomal protein S9 (Fragment)	-0.66	-0.18	0.16	0.34	1.01	1.00	1.00	1.01	7
P62851	F2Z5G8	PIG Uncharacterized protein (Fragment) RPS25	-0.61	-0.02	-0.18	0.37	1.00	1.00	1.00	1.01	6
-	P62888	HUMAN 60S ribosomal protein L30	-0.58	-0.38	0.05	0.46	1.00	1.00	1.00	1.02	4
P02549	F1RR78	PIG Uncharacterized protein LOC100049693	-0.65	-0.51	-0.49	1.02	1.00	1.00	1.00	1.01	149
-	F1RI01	PIG 60S ribosomal protein L13a	-0.36	-0.43	0.11	-0.08	0.99	0.99	1.00	1.01	3
-	P53027	PIG 60S ribosomal protein L10a (Fragment)	-0.86	-0.46	0.33	0.22	0.99	0.99	1.00	1.01	5
-	Q29205	PIG 60S ribosomal protein L11	-0.33	-0.3	-0.47	0.26	1.00	1.00	1.01	1.01	3
-	P46782	HUMAN 40S ribosomal protein S5	-0.75	-0.62	0.25	0.23	1.01	0.99	1.00	1.01	6
-	P62910	HUMAN 60S ribosomal protein L32	-0.01	0.22	-0.52	-0.69	1.00	1.00	1.01	1.03	2
P46776	I3LK51	PIG Uncharacterized protein (Fragment)	0.22	0.31	-0.73	-0.85	1.00	1.01	0.99	1.03	3

-	B7TJ03	PIG Ribosomal protein L26-like 1	-1.13	-0.47	-0	0.54	0.92	0.99	1.00	1.03	5
-	F1S530	PIG 60S ribosomal protein L5	-1.58	-0.81	0.06	1.21	0.71	0.97	1.00	0.96	7
-	I3LVI8	PIG 60S ribosomal protein L7a	-0.81	-0.99	0.03	0.63	1.00	0.95	1.00	1.03	2
-	P62829	HUMAN 60S ribosomal protein L23	-1.14	-0.37	-0.01	0.22	0.91	1.00	1.00	1.01	5
-	P62424	HUMAN 60S ribosomal protein L7a	-1.07	-0.79	-0.05	0.61	0.94	0.97	1.00	1.03	4
-	P62249	HUMAN 40S ribosomal protein S16	-0.27	-0.68	-0.24	-0.17	1.00	0.98	1.00	1.01	2
P62249	F1SP46	PIG Uncharacterized protein	-0.75	-0.8	0.05	0.12	1.01	0.97	1.00	1.00	6
-	Q29214	PIG 60S acidic ribosomal protein P0	0.1	-0.05	-0.56	-0.87	1.00	1.00	1.01	1.03	8
-	F6Q5P0	PIG 40S ribosomal protein S13	-0.1	-0.22	-0.44	-0.7	1.00	1.00	1.01	1.03	2
P46779	I3LSZ6	PIG Uncharacterized protein RPL28	-0.88	-0.2	-0.48	-0.19	0.99	1.00	1.01	1.00	1
-	P62979	HUMAN Ubiquitin-40S ribosomal protein S27a	-0.94	-0.52	-0.21	-0.14	0.97	1.00	1.00	1.00	2
P18124	F1RWI5	PIG Uncharacterized protein	-0.58	-0.48	-1.26	0.49	1.00	0.99	0.89	1.02	9
-	P62841	HUMAN 40S ribosomal protein S15	-1.11	-1.62	1.11	-0.21	0.92	0.72	0.93	1.00	1
P62249	K7GLK5	PIG Uncharacterized protein	-0.69	-0.65	-0.41	-0.18	1.01	0.98	1.00	1.01	4
-	P62269	HUMAN 40S ribosomal protein S18	-1.1	-1.04	-0.37	0.56	0.92	0.95	1.00	1.03	5
-	A1XQU9	PIG 40S ribosomal protein S20	-0.35	-0.44	-0.56	-0.64	1.00	0.99	1.01	1.03	3
-	Q6SA96	PIG 40S ribosomal protein S23	-1.59	-1.13	0.12	0.53	0.71	0.92	1.01	1.03	6
-	Q6QAS9	PIG 60S ribosomal protein L7 (Fragment)	-1.59	-0.92	-0.94	1.3	0.71	0.96	0.96	0.94	3
-	I3LS62	PIG Ribosomal protein L15	-1.79	-1.08	-0.55	0.69	0.61	0.94	1.01	1.03	3
-	P63220	HUMAN 40S ribosomal protein S21	-0.31	-1.51	-1.41	0.16	1.00	0.77	0.82	1.00	1
-	P79324	PIG 60S ribosomal protein L15 (Fragment)	-1.48	-1.82	-0.18	-0.36	0.76	0.59	1.00	1.01	4
-	Q29187	PIG 60S ribosomal protein L4 (Fragment)	-2.14	-1.94	-1.29	1.49	0.40	0.53	0.88	0.87	1
-	A1XQU3	PIG 60S ribosomal protein L14	-1.74	-1	-1.21	0	0.64	0.95	0.90	1.00	4
P62081	I3L7B0	PIG Uncharacterized protein (Fragment)	-0.84	-1.23	-1.04	-1.08	0.99	0.88	0.94	0.99	2
-	P62913	HUMAN 60S ribosomal protein L11	-0.07	-1.17	-2.14	-0.85	1.00	0.91	0.41	1.03	1
-	P63173	HUMAN 60S ribosomal protein L38	-0.97	-0.82	-1.74	-0.84	0.97	0.97	0.68	1.03	2
-	Q6QAQ3	PIG 60S ribosomal protein L23 (Fragment)	-1.8	-1.75	-2.23	-0.89	0.61	0.63	0.36	1.03	1
P05386	F1SIT7	PIG Uncharacterized protein LOC100523874	0.92	-0.48	-2.87	-5.27	0.97	0.99	0.10	0.00	1

Human equivalent Ac. No.	Accession No.	Collagen<br>Protein	Zq 120	Zq 1	Zq 4	Zq 7	FDR 120	FDR 1	FDR 4	FDR 7	No. peptides
P08123	F1SFA7	PIG Uncharacterized protein COL1A2	1.88	4.26	1.31	1.83	0.55	0.00	0.86	0.70	8
P02452	I3LJX2	PIG Uncharacterized protein	2.3	2.53	1.24	2.27	0.34	0.22	0.90	0.41	2
P02452	I3LUM2	PIG Uncharacterized protein	1.82	2.35	1.88	1.74	0.59	0.31	0.58	0.75	4
-	P08123	HUMAN Collagen alpha-2(I) chain	1.57	3.48	1.45	1.27	0.72	0.02	0.81	0.95	2
-	P02461	HUMAN Collagen alpha-1(III) chain	0.42	1.75	0.47	1.27	1.00	0.63	1.01	0.95	2
P02461	F1RYI8	PIG Uncharacterized protein COL3A1	0.7	0.8	0.67	1.6	1.00	0.97	1.00	0.82	2
-	P02452	HUMAN Collagen alpha-1(I) chain	0.41	1.82	-0.15	1.27	1.00	0.60	1.00	0.95	1
-	P12107	HUMAN Collagen alpha-1(XI) chain	0.38	-0.15	1.26	1.76	1.00	1.00	0.88	0.74	1
-	Q02388	HUMAN Collagen alpha-1(VII) chain	-0.46	-0.55	1.25	1.56	1.00	0.99	0.89	0.84	3
-	Q59IP2	PIG Procollagen alpha 2(V)	1.46	0.43	-0.05	-0.09	0.77	0.99	1.00	1.01	3
Q05707	K7GT00	PIG Uncharacterized protein (Fragment) COL14A1	0.69	0.8	-0.06	0.32	1.01	0.97	1.00	1.02	17
P02458	I3LSV6	PIG Uncharacterized protein (Fragment) COL2A1	0.4	0.32	-0.04	0.73	1.00	1.00	1.00	1.03	1
-	H0Y409	HUMAN Collagen alpha-2(IX) chain (Fragment)	0.44	0.28	0.96	-0.31	1.00	1.00	0.96	1.02	1
Q8IZC6	I3LDG8	PIG Uncharacterized protein	0.93	0.15	-0.07	0.13	0.97	0.99	1.00	1.00	1
Q02388	F1SKM1	PIG Uncharacterized protein COL7A1	1.65	0.33	-0.01	-1.48	0.68	1.00	1.00	0.88	1
P20908	F1S021	PIG Uncharacterized protein (Fragment) COL5A1	-0.67	0.14	0.03	0.76	1.01	0.99	1.00	1.03	2
-	F1SSE7	PIG Uncharacterized protein (Fragment) COL15A1	0.96	0.49	-0.1	-1.34	0.97	1.00	1.00	0.92	3
-	Q1T7A9	PIG Type VI collagen alpha-1 chain (Fragment)	0.22	-0.3	-0.69	-0.31	1.00	1.01	0.99	1.02	2
-	P12111	HUMAN Collagen alpha-3(VI) chain	-0.51	-0.68	-1.25	0.72	1.00	0.98	0.89	1.03	1
-	Q59IP1	PIG Procollagen alpha 3(V)	-0.67	-0.69	-0.87	-0.44	1.01	0.98	0.97	1.02	2
-	Q1T7A8	PIG Type VI collagen alpha-1 chain (Fragment)	-0.67	-1.39	-1.32	0.68	1.01	0.83	0.86	1.03	3
-	Q1T7A5	PIG Type VI collagen alpha-3 chain (Fragment)	-1.17	-1.48	-1.2	0.78	0.90	0.79	0.90	1.03	19
Q05707	F1S285	PIG Uncharacterized protein COL14A1	-1.89	-1.22	-0.58	0.38	0.55	0.89	1.01	1.01	4
-	F1RZK4	PIG Uncharacterized protein COL10A1	-1.88	-1	-1.18	0.29	0.56	0.95	0.91	1.02	1
-	E7EX21	HUMAN Collagen alpha-1(XIII) chain	-1.11	-0.52	-1.26	-1.42	0.92	1.00	0.88	0.90	1
P08572	F1RLL9	PIG Uncharacterized protein (Fragment) COL4A2	-0.69	-1.38	-1.9	-0.76	1.01	0.83	0.57	1.03	2
-	P27658	HUMAN Collagen alpha-1(VIII) chain	-1.36	-1.87	-1.09	-0.46	0.83	0.57	0.93	1.02	1
-	P12109	HUMAN Collagen alpha-1(VI) chain	-1.93	-2.12	-1.98	0.85	0.53	0.43	0.52	1.03	5
P39059	I3LIL7	PIG Uncharacterized protein LOC100620394	-0.57	-1.45	-1.49	-1.71	1.00	0.79	0.80	0.77	4

P12109	I3LS72	PIG Uncharacterized protein COL6A1	-2.86	-3.44	-2.19	1.5	0.12	0.03	0.39	0.87	6

Supplementary Table S9. Protein expression changes in the functionally annotated categories within cluster e.

Human equivalent Ac. No.	Accession No.	DNA damage checkpoint regulation<br>Protein	Zq 120	Zq 1	Zq 4	Zq 7	FDR 120	FDR 1	FDR 4	FDR 7	No. peptides
Q92831	I3LRW1	PIG Uncharacterized protein (Fragment) KAT2B	-2.14	-4.48	-1.41	-3.1	0.40	0.00	0.82	0.06	1
-	A0SNV3	PIG Tyrosine 3-monooxygenase/tryptophan 5-monooxygenase activation protein	-1.65	-2.17	-1.4	-1.57	0.68	0.41	0.83	0.84	6
-	P61981	HUMAN 14-3-3 protein gamma	-0.68	-0.97	-0.83	-1.45	1.01	0.95	0.97	0.88	9
-	F1SIF5	PIG Uncharacterized protein PTPMT1	0.43	-0.12	-1.07	-1.43	1.00	1.00	0.94	0.89	1
Q13616	F1SAD7	PIG Uncharacterized protein CUL1	0.03	0.36	-0.86	0.09	1.00	1.00	0.97	1.01	2
P62258	K7GP62	PIG Uncharacterized protein	0.36	-0.08	0.29	-0.58	0.99	1.00	1.00	1.03	2
-	P63208	HUMAN S-phase kinase-associated protein 1	2.3	0.93	-0.17	-2.51	0.34	0.96	1.00	0.26	2
P62258	F2Z4Y1	PIG Uncharacterized protein YWHAH	-0.07	0.79	0.23	-0.22	1.00	0.97	1.00	1.01	12
-	D2IE28	PIG Cyclin B1 transcript variant 1	2.43	0.41	-0.44	-1.54	0.29	0.99	1.01	0.85	1
-	K7EM20	HUMAN 14-3-3 protein epsilon (Fragment)	2.41	-0.03	-0.12	-1.27	0.30	1.00	1.00	0.95	2
P31946	F1SDR7	PIG Uncharacterized protein YWHAB	0.9	0.79	0.58	-0.58	0.98	0.97	1.01	1.03	7
-	P62258	HUMAN 14-3-3 protein epsilon	0.82	0.58	0.53	0.02	0.99	0.99	1.01	1.00	4
-	F1RS45	PIG DNA topoisomerase 2	0.63	2.86	0.18	-0.54	1.00	0.11	1.00	1.03	1

Human equivalent Ac. No.	Accession No.	Response to oxidative stress<br>Protein	Zq 120	Zq 1	Zq 4	Zq 7	FDR 120	FDR 1	FDR 4	FDR 7	No. peptides
-	Q99814	HUMAN Endothelial PAS domain-containing protein 1	-2.22	-2.43	-1.95	-1.94	0.37	0.27	0.54	0.64	1
P30048	F1S418	PIG Uncharacterized protein PRDX3	0.61	-1	-1.22	-4.36	1.00	0.95	0.90	0.00	6
O95168	I3LPW0	PIG Uncharacterized protein NDUFB4	-1.16	-0.9	-1.78	-1.4	0.90	0.97	0.65	0.90	7
-	P52552	PIG Peroxiredoxin-2 (Fragment)	0.39	-0.61	-0.77	-3.8	1.00	0.99	0.98	0.01	7
Q99497	F1RII4	PIG Uncharacterized protein PARK7	-0.48	-0.72	-0.98	-1.67	1.00	0.97	0.96	0.79	8
-	P55931	PIG Electron transfer flavoprotein-ubiquinone oxidoreductase, mitochondrial	-1.21	-1.01	-0.75	-0.11	0.89	0.95	0.99	1.00	20
Q8NCN5	F1S430	PIG Uncharacterized protein (Fragment) LOC100525578	-0.2	-0.22	-1.33	-1.2	1.00	1.00	0.86	0.97	3
-	Q0R678	PIG DJ-1 protein	-0.01	-0.54	-0.43	-1.41	1.00	0.99	1.01	0.90	4

Q9UI09	F1SQP4	PIG Uncharacterized protein (Fragment) NDUFA12	-0.74	-0.71	-0.31	-0.54	1.01	0.97	1.00	1.03	11
O75306	F1S1A8	PIG Uncharacterized protein NDUFS2	0.37	0	-1.17	-1.45	0.99	1.00	0.92	0.88	17
-	P36968	PIG Phospholipid hydroperoxide glutathione peroxidase, mitochondrial	-1.31	0.47	-1.54	0.76	0.85	0.99	0.77	1.03	4
Q06830	F1S3U9	PIG Uncharacterized protein PRDX1	0.37	-0.76	0.46	-1.67	1.00	0.97	1.01	0.78	8
-	A5A791	PIG DNA-repair protein complementing XP-D cells (Fragment)	0.48	0.06	-0.54	-1.52	1.00	1.00	1.01	0.86	1
-	F1SDX0	PIG Uncharacterized protein ERCC6	0.36	0.57	-0.91	-1.18	0.99	0.99	0.96	0.98	1
-	Q9GLW8	PIG Peroxiredoxin 5	-0.15	1.1	-1.9	-0.04	1.01	0.92	0.57	1.00	1
O00151	F1SC51	PIG Uncharacterized protein	0.85	0.98	-0.66	-1.66	0.99	0.95	1.00	0.79	15
-	Q863I2	PIG Serine/threonine-protein kinase OSR1	0.01	0.69	-0.9	-0.3	1.00	0.97	0.96	1.02	8
-	K7GNQ2	PIG Eosinophil peroxidase	1.73	-1.32	-0.37	-0.49	0.65	0.86	1.00	1.02	1
-	P30041	HUMAN Peroxiredoxin-6	1.53	1.31	0.4	-3.28	0.74	0.86	1.00	0.04	1
-	Q9TSX9	PIG Peroxiredoxin-6	-0.45	-0.18	-0.07	0.75	1.00	1.00	1.00	1.03	11
P30519	F1RK58	PIG Uncharacterized protein HMOX2	0.48	0	-0.5	0.21	1.00	1.00	1.00	1.00	3
-	Q8MJ14	PIG Glutathione peroxidase 1	-0.03	0.22	-0.26	0.31	1.00	1.00	1.00	1.02	4
-	Q6XQN2	PIG Nicotinate phosphoribosyltransferase-like protein (Fragment)	-0.35	-1.2	2.29	-0.43	0.99	0.90	0.32	1.02	1
-	P49927	PIG Major prion protein	-0.15	-0.39	0.46	0.5	1.01	1.00	1.01	1.03	1
P30086	F1RKG8	PIG Uncharacterized protein PEBP1	-0.21	0.63	1.5	-1.42	1.00	0.99	0.79	0.90	9
P48637	F1S4X9	PIG Uncharacterized protein GSS	-0.37	-0.49	0.94	0.5	0.99	1.00	0.96	1.03	1
-	I3L677	PIG Glucose-6-phosphate 1-dehydrogenase (Fragment)	0.63	-0.36	-0.82	1.34	1.00	1.00	0.97	0.92	2
P35580	F1SSA6	PIG Uncharacterized protein (Fragment) LOC396903	0.24	0.71	0.11	0.44	1.00	0.97	1.00	1.02	36
-	Q9NRD9	HUMAN Dual oxidase 1	-0.1	0.07	0.74	1.03	1.00	1.00	0.99	1.01	1
F1S1E9	F1S1E9	PIG Uncharacterized protein OXR1	-0.72	0.23	0.58	1.67	1.00	1.00	1.01	0.79	1
-	P11493	PIG Serine/threonine-protein phosphatase 2A catalytic subunit beta isoform	1.07	0.6	0.44	0.18	0.93	0.99	1.01	1.01	1
-	F1RHU1	PIG Uncharacterized protein PNKP	1.61	1.9	1.87	0.75	0.70	0.56	0.58	1.03	1
-	P36639	HUMAN 7,8-dihydro-8-oxoguanine triphosphatase	0.67	3.85	2.35	0.13	1.01	0.01	0.30	1.00	1
-	F1SRG2	PIG NADH dehydrogenase [ubiquinone] 1 alpha subcomplex subunit 6	1.86	7.68	8.14	17.9	0.56	0.00	0.00	0.00	1

NAD or NADP as acceptor

Human equivalent Ac. No.	Accession No.	Protein	Zq				FDR				No. peptides
			120	1	4	7	120	1	4	7	
P49419	I3L670	PIG Uncharacterized protein	-1.74	-4.32	-1.49	-0.98	0.64	0.00	0.79	1.02	2
-	D6QST6	PIG 2,4-dienoyl-CoA reductase 1	-1.41	-1.25	-4.88	-0.94	0.80	0.88	0.00	1.02	1

-	P49419	HUMAN Alpha-aminoadipic semialdehyde dehydrogenase	-1.35	-2.81	-2.39	-1.09	0.83	0.13	0.28	0.99	1
P51648	F1SDC7	PIG Uncharacterized protein ALDH3A2	-2.27	-2.03	-2.55	-0.22	0.35	0.48	0.21	1.01	10
P49419	F1RKM1	PIG Uncharacterized protein (Fragment) ALDH7A1	-0.87	-2.56	-1.24	-1.52	0.99	0.21	0.90	0.86	11
P30038	I3LNB4	PIG Uncharacterized protein (Fragment) ALDH4A1	-0.58	-1.21	-1.66	-1.7	1.00	0.89	0.71	0.77	17
-	P30043	HUMAN Flavin reductase (NADPH)	-0.11	0.35	-1.8	-2.82	1.01	1.00	0.64	0.14	1
P51649	F1RUE3	PIG Uncharacterized protein ALDH5A1	-1.29	-0.98	-1.51	-0.56	0.86	0.95	0.79	1.03	11
-	Q16698	HUMAN 2,4-dienoyl-CoA reductase, mitochondrial	-0.84	-1.31	0.3	-1.96	0.99	0.86	1.00	0.63	2
-	Q99714	HUMAN 3-hydroxyacyl-CoA dehydrogenase type-2	-1.24	-1.68	0.23	-0.91	0.88	0.68	1.00	1.03	1
-	F1S232	PIG 4-trimethylaminobutyraldehyde dehydrogenase	-0.61	-1.01	-0.82	-1.13	1.00	0.95	0.97	0.98	10
Q02252	F1S3H1	PIG Uncharacterized protein ALDH6A1	-0.39	-0.65	-1.27	-1.14	1.00	0.98	0.88	0.98	20
-	F1RGV4	PIG Uncharacterized protein DECR2	-0.73	-1.23	-1.3	0.02	1.01	0.88	0.87	1.00	1
-	F1RHN4	PIG Uncharacterized protein TXNRD2	-0.39	-0.03	-1.36	-1.46	1.00	1.00	0.84	0.88	3
Q99714	K7GND4	PIG Uncharacterized protein (Fragment) HSD17B10	-0.81	-0.53	-0.36	-1.28	1.00	1.00	1.00	0.95	3
P11766	F1S0C1	PIG Uncharacterized protein ADH5	-1.11	-0.97	-0.28	-0.5	0.92	0.95	1.00	1.03	7
-	Q9BPX1	HUMAN 17-beta-hydroxysteroid dehydrogenase 14	0.2	-0.58	-1.42	-0.64	1.00	0.99	0.82	1.03	1
-	K7GRM6	PIG Uncharacterized protein (Fragment) HSD17B10	0.15	0.19	-0.58	-1.65	1.01	1.00	1.01	0.80	1
Q9UDR5	F1SLX5	PIG Uncharacterized protein AASS	-0.2	-0.23	-0.97	-0.29	1.01	1.00	0.96	1.01	16
P51659	I3LEF8	PIG Uncharacterized protein HSD17B4	-0.29	-0.09	-0.63	-0.2	1.00	1.00	1.01	1.01	7
-	I3LQH7	PIG Uncharacterized protein BLVRB	0.04	0.83	-0.43	-1.44	1.00	0.97	1.00	0.88	3
-	F1RL81	PIG Uncharacterized protein HSD17B14	-0.11	-0.48	0.26	-0.39	1.00	0.99	1.00	1.01	1
-	Q9XT00	PIG Estradiol 17-beta-dehydrogenase 8	0.57	-0.35	0.19	-0.81	1.00	1.00	1.01	1.03	3
-	D0G6Y0	PIG Hydroxysteroid (17-beta) dehydrogenase 12 (Fragment)	-0.23	-0.06	0.29	-0.4	1.00	1.00	1.00	1.01	1
Q9BRA2	F1RGM3	PIG Uncharacterized protein TXNDC17	0.39	1.14	-0.04	-1.57	1.00	0.91	1.00	0.84	4
-	Q29228	PIG 4-trimethylaminobutyraldehyde dehydrogenase (Fragment)	-0.21	-0.51	0.74	-0.01	1.00	1.00	0.99	1.00	2
Q99714	K7GMY3	PIG Uncharacterized protein (Fragment) HSD17B10	0.71	-0.16	0.16	-0.25	1.00	1.00	1.00	1.01	3
-	Q8MKG1	PIG Hydroxysteroid 11-beta dehydrogenase 2	-0.31	0.22	0.42	1.01	1.00	1.00	1.00	1.01	1
-	F1SG38	PIG Thioredoxin reductase 1, cytoplasmic	0.94	0.89	0.3	-0.48	0.97	0.97	1.00	1.02	3
-	Q9TV69	PIG Trans-1,2-dihydrobenzene-1,2-diol dehydrogenase	0.26	-0.1	1.56	0.24	1.00	1.00	0.75	1.01	8
-	Q3SY69	HUMAN Mitochondrial 10-formyltetrahydrofolate dehydrogenase	-0.85	0.7	0.96	1.38	0.99	0.97	0.96	0.91	1
-	Q28956	PIG 17beta-estradiol dehydrogenase	1.09	1.59	-0.13	-0.19	0.93	0.73	1.00	1.01	5
-	Q9MYY8	PIG Thioredoxin reductase 1, cytoplasmic	1.63	0.9	1.2	0.2	0.70	0.97	0.90	1.00	2

Supplementary Table S10. Comparison of temporal protein changes between remote and ischemic myocardium during the first week of reperfusion.

Uniprot Accession No.	Protein name	Gene ID	ISCHEMIC (Zq) R-120 min	ISCHEMIC (Zq) R- 24 hours	ISCHEMIC (Zq) R-Day 4	ISCHEMIC (Zq) R-Day 7	REMOTE (Zq) R-120 min	REMOTE (Zq) R- 24 hours	REMOTE (Zq) R-Day 4	REMOTE (Zq) R-Day 7	No. Peptides Ischemic tissue	No. Peptides Remote tissue
I3LEK3	PIG Uncharacterized protein (Fragment)	CALML6	10.3	8.5	1.2	2.0	1.6	1.5	1.7	0.0	1	1
P02042	HUMAN Hemoglobin subunit delta	HBD	2.1	8.3	7.6	1.9	0.6	0.3	2.2	-0.3	1	2
F1SA43	PIG Uncharacterized protein	FAM71D	1.8	8.2	7.1	2.3	0.5	0.6	2.1	0.0	1	1
P02067	PIG Hemoglobin subunit beta	HBB	0.7	7.2	6.5	1.9	-0.3	-1.0	3.7	-0.5	11	10
P01965	PIG Hemoglobin subunit alpha	HBA	0.6	7.5	6.4	1.7	0.2	-0.4	4.5	0.5	10	10
P02009	PIG Hemoglobin subunit zeta	NA	0.7	6.3	6.7	2.3	0.1	-0.3	3.9	1.4	1	1
P02008	HUMAN Hemoglobin subunit zeta	HBZ	1.4	5.9	5.8	2.1	3.2	5.4	3.3	3.5	1	1
I3LMI5	PIG Uncharacterized protein	BRPF1	0.9	3.7	5.3	5.2	-0.8	0.7	0.2	-0.5	1	1
B4DJT1	HUMAN Protein DPCD	DPCD	4.2	7.0	2.4	1.2	0.0	0.7	0.1	0.2	1	1
Q96JM4	HUMAN Leucine-rich repeat and IQ domain-containing protein 1	LRRIQ1	0.8	4.7	5.0	4.2	0.2	0.4	0.1	2.2	1	1
Q6P597	HUMAN Kinesin light chain 3	KLC3	1.1	5.8	5.5	1.6	-0.3	-0.1	1.7	-0.4	1	1
P27917	PIG Apolipoprotein C-III	APOC3	2.0	6.1	4.5	1.4	0.5	0.1	3.0	0.6	7	4
Q8SPS7	PIG Haptoglobin	HP	3.1	3.5	4.4	2.8	3.3	4.1	9.5	4.0	14	11
Q14315	HUMAN Filamin-C	FLNC	-0.3	6.2	6.1	1.7	-0.4	-0.7	3.1	1.1	1	2
Q28936	PIG Fibrinogen A-alpha-chain (Fragment)	NA	1.2	3.8	4.9	3.4	-2.1	-0.3	3.0	5.7	5	4
K7ZLA7	PIG IgG heavy chian constant region (Fragment)	IGHG1	4.1	4.1	3.1	2.0	0.6	0.7	5.2	-0.7	9	7
P15175	PIG Cathelin	NA	2.4	5.1	4.1	1.4	2.2	2.8	0.5	5.4	4	2
K7GLE1	PIG Annexin	ANXA1	0.8	4.1	4.5	3.6	-0.8	-0.2	0.3	1.0	1	1
P35054	PIG Transforming growth factor beta receptor type 3	TGFBR3	4.2	5.9	2.7	0.1	3.5	7.9	5.6	-2.3	1	1
I3LAF6	PIG Uncharacterized protein	NA	7.4	2.0	2.6	0.9	-0.4	-1.3	-0.8	1.0	1	3
P01846	PIG Ig lambda chain C region	NA	2.8	3.4	3.5	2.6	-0.5	-0.6	5.5	0.2	3	3
Q10713	HUMAN Mitochondrial-processing peptidase subunit alpha	PMPCA	5.1	6.9	1.2	-1.2	5.4	7.2	6.2	-2.4	1	1
Q9UK32	HUMAN Ribosomal protein S6 kinase alpha-6	RPS6KA6	3.5	5.1	2.7	0.4	4.0	12.2	8.8	-1.0	1	1
F1SS51	PIG Uncharacterized protein (Fragment)	MAP2K4	2.6	3.1	3.1	2.9	-0.1	-1.7	2.2	-0.3	1	1
A5D9N3	PIG Allograft inflammatory factor 1	AIF1	0.7	2.4	4.1	4.5	1.7	1.6	-0.9	-1.1	1	1
Q29549	PIG Clusterin	CLU	2.3	3.8	3.8	1.7	1.2	-0.6	-0.6	-1.5	28	3
F1RX36	PIG Fibrinogen alpha chain	FGA	0.7	3.0	4.3	3.6	-1.4	-0.2	3.0	5.7	4	4
I3L6R8	PIG Uncharacterized protein	FBRS	2.2	2.6	3.4	3.3	1.2	0.2	-0.8	-0.8	1	1
F1RII7	PIG Hemoglobin subunit beta	HBB	0.1	4.7	5.2	1.5	-0.7	-1.2	3.0	0.6	2	2
Q29014	PIG Alpha-1 acid glycoprotein (Fragment)	NA	4.2	3.6	2.0	1.7	5.0	2.8	2.6	-0.1	10	6

P00747	HUMAN Plasminogen	PLG	1.2	4.4	3.8	2.0	2.2	2.0	2.1	1.8	1	1
Q9H898	HUMAN Zinc finger matrin-type protein 4	ZMAT4	0.3	4.1	4.8	2.1	-0.3	-0.5	2.3	1.3	1	1
F1RSY9	PIG Uncharacterized protein	CDC40	1.7	3.8	2.6	3.1	-1.3	-1.2	-1.1	0.9	1	1
Q8IUL9	HUMAN Hemoglobin beta chain variant Hb.Sinai-Bel Air (Fragment)	HBB	1.7	4.9	3.8	0.8	-0.4	-0.8	0.8	-0.6	4	1
I3LAQ0	PIG Uncharacterized protein	IGKC	2.9	3.2	2.9	2.2	0.0	-0.1	5.1	-1.0	5	4
Q9Y6Q2-2	HUMAN Isoform 2 of Stonin-1	STON1	0.8	4.6	4.4	1.3	0.8	1.0	1.7	0.9	1	1
Q9GMA8	PIG Alpha-1-antichymotrypsin 3 (Fragment)	SERPINA3-3	3.1	2.9	2.6	2.3	0.9	0.9	5.6	1.0	3	3
I3LQR9	PIG Fibrinogen alpha chain	FGA	1.3	2.5	3.6	3.5	-1.0	0.1	2.7	5.3	12	9
F1SRE3	PIG Uncharacterized protein	NA	1.0	3.5	4.1	2.4	-0.2	0.2	-0.2	-0.8	2	1
Q6EMK4	HUMAN Vasorin	VASN	0.6	4.7	4.8	0.7	0.4	-0.2	-0.1	-2.7	1	2
Q9BXY5	HUMAN Calcyphosin-2	CAPS2	1.8	5.5	2.4	0.9	0.3	0.4	0.3	-0.5	1	1
I3LK76	PIG Uncharacterized protein (Fragment)	SYT12	2.7	3.8	3.5	0.7	0.5	0.6	1.4	-0.7	1	1
I3LBK9	PIG Uncharacterized protein	DNAJC3	4.0	5.5	2.1	-1.0	2.3	6.9	4.5	-1.8	1	1
K7ZMG0	PIG IgG heavy chian constant region (Fragment)	IGHG6-2	3.0	3.0	2.7	1.9	0.7	-0.4	6.3	1.3	11	10
Q86UD7	HUMAN TBC1 domain family member 26	TBC1D26	3.7	3.1	2.1	1.7	1.0	-0.7	1.2	-3.1	1	1
L8B0S2	PIG IgG heavy chain	IGHG	3.0	2.8	2.8	1.9	0.6	0.5	4.9	0.5	2	1
F1RVE2	PIG Uncharacterized protein	HELQ	3.2	2.8	2.2	2.3	2.3	0.8	1.0	0.5	1	1
I3LN42	PIG Uncharacterized protein (Fragment)	GC	2.9	3.8	2.2	1.4	1.1	-0.8	2.4	-1.1	13	7
P08835	PIG Serum albumin	ALB	3.4	2.9	2.3	1.7	0.2	-1.5	3.2	-1.9	28	24
K7ZRJ9	PIG IgG heavy chian constant region (Fragment)	IGHG5-2	3.0	2.5	2.5	2.3	0.4	0.0	5.4	3.0	4	4
A0SEG9	PIG Complement component C9	NA	2.3	2.9	3.0	2.0	0.5	0.3	0.3	1.3	2	1
K7GL65	PIG Uncharacterized protein	CLEC3B	1.8	3.0	2.4	3.0	0.6	0.6	-0.7	-1.7	1	1
I3L651	PIG Uncharacterized protein	NA	1.3	2.5	3.4	3.1	-0.8	0.1	3.1	4.7	27	20
F1RPL9	PIG Uncharacterized protein	LOC100525442	3.0	2.5	2.3	2.2	0.6	0.6	-0.5	-0.1	1	1
Q14587	HUMAN Zinc finger protein 268	ZNF268	2.4	3.8	3.3	0.5	0.9	-0.3	-0.4	-1.0	2	2
F1RX35	PIG Uncharacterized protein	LOC100627396	0.5	2.1	3.8	3.5	-1.7	-0.8	2.8	4.6	23	15
L8B0W0	PIG IgG heavy chain	IGHG	2.3	2.8	3.0	1.8	-0.3	-0.2	2.4	-0.5	2	1
Q96LA8	HUMAN Protein arginine N-methyltransferase 6	PRMT6	2.9	5.8	1.4	-0.3	1.9	1.1	3.5	2.3	1	1
P48819	PIG Vitronectin	VTN	1.5	2.3	3.5	2.4	-0.6	-1.8	0.1	1.0	11	5
F1SCD0	PIG Uncharacterized protein	SERPINA3-3	2.8	3.4	2.0	1.4	0.2	1.6	3.6	2.0	7	5
O43240	HUMAN Kallikrein-10	KLK10	2.7	2.4	2.4	2.2	-0.7	-2.0	1.1	-2.5	1	1
F1S9C0	PIG Serum amyloid A protein (Fragment)	LOC100525856	0.9	4.7	2.7	1.4	0.4	3.6	5.2	6.5	4	1
A6NHR9	HUMAN Structural maintenance of chromosomes flexible hinge domain-containing protein 1	SMCHD1	4.0	5.6	1.0	-1.1	6.2	16.0	16.2	0.6	1	1
I3LGX0	PIG Uncharacterized protein (Fragment)	PPIL4	3.6	4.1	1.6	0.2	2.4	3.0	-0.6	-0.4	1	1
I3LRQ8	PIG Uncharacterized protein (Fragment)	CCDC77	-1.5	6.0	3.9	1.1	-0.9	-0.9	-0.2	-0.6	1	1
P19619	PIG Annexin A1	ANXA1	0.8	3.2	3.1	2.4	0.7	0.6	0.6	1.1	17	10
Q5JXB2	HUMAN Putative ubiquitin-conjugating enzyme E2 N-like	UBE2NL	2.9	2.4	2.0	2.1	0.4	0.5	3.5	-0.6	1	1
F1RXC2	PIG Uncharacterized protein (Fragment)	CA2	0.9	3.9	3.5	1.1	1.0	0.3	2.1	-0.2	10	4
P60709	HUMAN Actin, cytoplasmic 1	ACTB	0.7	1.3	2.8	4.5	-1.1	-0.8	0.0	1.0	1	1
C3S7K5	PIG Calcium-binding protein A8	S100A8	0.4	4.4	3.3	1.1	0.5	0.6	0.5	1.5	6	1

H7C315	HUMAN Formimidoyltransferase-cyclodeaminase (Fragment)	FTCD	0.8	3.0	4.3	1.1	1.0	0.4	2.8	1.6	1	2
P00450	HUMAN Ceruloplasmin	CP	2.5	1.7	2.3	2.6	0.7	0.0	4.5	2.5	1	1
F1STC2	PIG Uncharacterized protein	LOC100625243	2.6	2.6	2.4	1.5	1.5	1.7	7.5	0.5	2	1
F1SHX3	PIG Uncharacterized protein	RIF1	1.4	2.5	2.8	2.3	0.0	0.4	1.3	1.3	1	1
L8B0V9	PIG IgG heavy chain	IGHG	2.8	2.8	2.4	1.1	0.7	0.5	2.5	-1.1	2	2
L8B0T7	PIG IgG heavy chain	IGHG	3.1	2.5	2.2	1.2	0.8	-0.3	3.4	0.2	6	3
I3LEV5	PIG Uncharacterized protein (Fragment)	NA	-0.4	4.0	4.1	1.3	-0.9	-0.4	3.1	0.4	1	1
I3LTM2	PIG Uncharacterized protein (Fragment)	SYNE2	1.2	4.7	2.3	0.8	-0.2	-0.3	-0.3	1.3	1	1
F1SFI5	PIG Uncharacterized protein	HRG	2.3	3.3	1.9	1.4	1.8	1.5	1.6	0.0	12	9
P18650	PIG Apolipoprotein E	APOE	0.9	3.4	3.2	1.4	0.6	0.1	1.3	1.0	25	4
Q06AU2	PIG Ras-related protein Rap-2a	RAP2A	2.1	2.4	2.4	2.0	1.4	2.0	1.1	-0.3	2	1
Q2EN75	PIG Protein S100-A6	S100A6	0.6	1.2	3.0	4.1	1.6	0.1	1.8	1.2	2	1
H3BPA4	HUMAN Exocyst complex component 3-like protein (Fragment)	EXOC3L1	2.5	2.3	2.4	1.5	0.4	1.3	2.6	0.8	1	1
K7ZJP7	PIG IgM heavy chain constant region (Fragment)	IGHM	1.7	2.3	3.2	1.6	0.1	-1.1	2.6	-0.7	12	6
F1RII5	PIG Uncharacterized protein	LOC100515788	1.3	3.0	3.2	1.1	2.0	1.4	1.7	-0.1	1	1
P79263	PIG Inter-alpha-trypsin inhibitor heavy chain H4	ITIH4	1.1	2.5	1.9	3.0	-0.3	2.1	4.0	9.4	39	23
F1SUD6	PIG Uncharacterized protein (Fragment)	FAM120A	1.6	3.2	2.7	1.0	0.4	0.6	0.2	1.6	3	3
K7ZRK0	PIG IgA heavy chian constant region (Fragment)	IGHA	2.5	2.6	2.2	1.3	0.7	1.1	3.7	0.0	8	4
Q9GMA7	PIG Alpha-1-antichymotrypsin 1 (Fragment)	SERPINA3-1	1.0	2.1	2.5	2.9	-0.5	0.7	3.8	4.5	7	3
Q19AZ8	PIG Prothrombin	F2	2.4	3.0	2.0	1.1	0.5	0.1	1.1	1.5	6	3
B3KRD4	HUMAN cDNA FLJ34067 highly similar to Chromodomain helicase-DNA-binding protein 5	NA	-1.1	9.6	0.7	-0.7	0.9	0.7	-0.5	1.2	1	1
Q9C026	HUMAN E3 ubiquitin-protein ligase TRIM9	TRIM9	-0.7	1.3	3.5	4.3	-0.8	0.6	0.7	1.3	1	1
F1SCD1	PIG Uncharacterized protein (Fragment)	NA	1.0	2.1	2.5	2.7	0.3	2.1	7.1	5.8	2	1
P06867	PIG Plasminogen	PLG	1.1	2.6	3.2	1.4	-1.1	-2.0	1.6	2.1	13	7
Q69DL0	PIG Complement C1q subcomponent subunit A	C1QA	0.9	2.6	3.1	1.7	-0.6	-0.6	-0.1	-0.5	4	1
F1SB81	PIG Plasminogen	PLG	0.2	2.6	2.9	2.4	-1.2	-2.1	-1.1	1.0	2	1
P09571	PIG Serotransferrin	TF	3.2	2.3	1.5	1.2	2.8	1.5	3.8	-0.6	34	28
Q9NYJ7	HUMAN Delta-like protein 3	DLL3	3.1	1.1	1.9	2.0	-0.3	-0.1	-0.4	-0.1	1	1
Q5GA58	PIG Proteasome subunit alpha type 2 (Fragment)	PSMA2	1.5	5.8	0.5	0.3	0.5	0.6	1.0	0.8	1	1
Q8N9B5	HUMAN Junction-mediating and -regulatory protein	JMY	6.8	1.1	-0.1	0.4	7.2	2.3	1.1	1.6	1	1
P14632	PIG Lactotransferrin	LTF	2.0	4.1	2.1	-0.2	0.7	0.9	-0.1	0.2	16	2
K7ZRJ8	PIG IgD heavy chain constant region (Fragment)	IGHD	1.6	2.0	2.8	1.7	0.1	-0.4	2.1	-0.1	2	1
K7GR72	PIG Uncharacterized protein	SLC4A1	0.6	3.5	3.2	0.7	1.2	2.0	2.6	1.0	14	4
I3L818	PIG Uncharacterized protein (Fragment)	SERPINF2	1.3	1.7	3.0	2.0	1.5	2.1	2.7	0.7	4	2
F1RUQ0	PIG Uncharacterized protein	IGJ	1.4	2.0	2.8	1.8	1.3	-0.1	2.8	-1.3	2	1
Q8N4C6	HUMAN Ninein	NIN	2.3	3.6	1.1	1.0	0.6	-0.1	-1.1	-0.7	1	1
P18648	PIG Apolipoprotein A-I	APOA1	2.4	2.6	1.8	1.2	0.8	-2.4	-1.1	-2.4	35	25
A5PF00	PIG B-factor, properdin	BF	2.4	1.4	2.0	2.3	0.3	3.1	4.7	5.1	1	1
F1SSN9	PIG Uncharacterized protein	DLGAP5	0.7	3.6	2.5	1.1	-0.1	-0.1	-1.1	-0.8	1	1
F1RSX9	PIG Uncharacterized protein (Fragment)	SNX25	0.4	1.6	2.5	3.3	0.6	0.5	0.5	1.4	1	1

P68871	HUMAN Hemoglobin subunit beta	HBB	1.2	3.5	2.4	0.7	0.9	0.7	0.4	-1.3	3	2
P50828	PIG Hemopexin	HPX	2.3	2.0	1.9	1.6	1.7	0.2	3.8	0.6	11	8
A5D9P0	PIG Ring finger protein 1	RING1	1.5	1.9	1.6	2.7	-0.3	0.4	0.3	0.5	1	1
I3LGN5	PIG Uncharacterized protein (Fragment)	NA	1.7	2.4	2.2	1.5	-0.6	0.0	1.3	1.0	1	1
P20305	PIG Gelsolin (Fragment)	GSN	0.6	3.0	2.7	1.4	-0.2	-0.8	0.3	-0.2	25	19
F1S4B4	PIG Uncharacterized protein	NA	5.2	2.3	-0.1	0.4	-0.4	-0.5	0.1	-0.5	1	1
F1SFI4	PIG Uncharacterized protein	KNG1	2.0	2.0	2.0	1.7	0.8	1.1	0.2	0.3	12	3
F1RWM0	PIG Uncharacterized protein	ZC2HC1A	3.0	2.6	1.6	0.5	4.5	5.9	3.0	-1.8	1	1
P01025	PIG Complement C3	C3	2.5	1.6	1.9	1.7	-0.2	-2.2	1.4	0.9	83	48
F1RPK9	PIG Uncharacterized protein	CHIC1	0.6	3.1	3.0	1.1	0.8	0.3	2.6	0.6	1	1
F1SH92	PIG Inter-alpha-trypsin inhibitor heavy chain H4	ITIH4	1.1	2.1	1.7	2.7	-0.1	2.9	5.5	10.9	6	2
Q6IPS8	HUMAN UTY protein	UTY	5.9	-0.7	0.9	1.5	5.0	-0.2	2.6	7.2	1	1
P01042	HUMAN Kininogen-1	KNG1	3.2	2.6	1.0	0.8	1.2	1.7	0.3	0.0	1	1
Q9GMA6	PIG Alpha-1-antichymotrypsin 2	SERPINA3-2	2.7	2.0	1.5	1.4	0.6	-0.3	2.9	0.7	21	16
F1SRL9	PIG Uncharacterized protein	LOC100522108	0.2	1.1	2.8	3.5	1.2	2.1	-0.1	0.8	14	4
F1RKI5	PIG Uncharacterized protein	CHSY3	1.9	1.9	1.9	1.8	-1.4	-1.6	-1.1	-0.1	1	1
F1SH94	PIG Uncharacterized protein	ITIH3	1.6	2.2	2.1	1.6	0.7	-0.3	0.7	0.9	14	3
Q9GMD6	PIG Antithrombin III (Fragment)	AT3	1.4	2.1	2.3	1.8	-0.2	-0.9	1.9	1.4	8	4
Q9XT08	PIG Apolipoprotein R (Fragment)	APOR	0.9	2.0	2.8	1.8	0.6	-0.3	1.4	0.3	1	1
P50447	PIG Alpha-1-antitrypsin	SERPINA1	2.6	2.1	1.6	1.2	0.8	0.1	1.9	-1.4	13	6
I3LQ26	PIG Uncharacterized protein	LRRC45	-0.8	3.8	3.6	0.9	-0.3	0.1	3.1	0.4	1	1
I3LQ17	PIG Uncharacterized protein	NA	2.0	1.8	2.1	1.6	1.2	0.4	4.2	2.6	50	23
F1SLX2	PIG Uncharacterized protein	A2M	2.0	2.1	1.9	1.4	0.5	-0.3	3.4	0.4	51	36
F1SCC6	PIG Uncharacterized protein	LOC100153899	1.5	2.1	1.9	1.9	1.1	3.0	5.4	6.9	15	10
P02549	HUMAN Spectrin alpha chain, erythrocytic 1	SPTA1	2.4	3.1	1.7	0.2	-1.2	-0.3	-0.3	1.1	5	1
F1RXC1	PIG Uncharacterized protein	CA1	0.0	3.3	3.1	1.0	-0.7	-1.8	0.2	-0.5	10	7
Q8SPK6	PIG Beta-actin (Fragment)	beta-actin	1.2	1.4	2.3	2.3	1.0	0.6	1.4	0.0	3	3
F1SL55	PIG Uncharacterized protein	MRPS22	1.9	2.4	1.5	1.4	0.6	0.4	-0.3	0.2	1	1
F1RKY2	PIG Uncharacterized protein	SERPIND1	2.4	2.0	1.6	1.2	0.3	-0.5	0.4	0.7	9	5
H0YA55	HUMAN Serum albumin (Fragment)	ALB	2.3	2.0	1.3	1.5	0.6	-0.3	1.3	-0.8	1	1
F1RVZ2	PIG Uncharacterized protein (Fragment)	FBF1	5.7	1.3	0.0	0.2	0.7	-0.1	0.3	-0.1	1	1
F1S1W6	PIG Uncharacterized protein (Fragment)	NOSTRIN	1.6	2.1	1.6	1.8	1.1	4.3	3.2	-0.4	1	1
I3LIE3	PIG Uncharacterized protein (Fragment)	LOC100511457	0.8	1.4	2.2	2.8	1.0	0.3	-1.0	-0.2	1	4
K7GRI0	PIG Uncharacterized protein	BCOR	4.1	2.6	0.4	0.0	5.2	2.4	1.9	0.5	1	1
F1S7K2	PIG Uncharacterized protein	LRG1	2.4	1.3	1.7	1.6	1.2	0.8	1.1	-0.5	4	2
Q29238	PIG Chloride intracellular channel protein 1 (Fragment)	CLIC1	1.7	1.5	1.7	2.1	0.0	1.1	-2.2	-1.3	2	1
F1SCC7	PIG Uncharacterized protein	LOC100156325	1.1	1.9	1.8	2.2	0.8	2.9	5.3	5.1	7	6
F1S7X0	PIG Uncharacterized protein (Fragment)	NA	1.7	2.2	1.7	1.4	-0.2	0.4	0.2	1.1	1	1
Q6Y288	HUMAN Beta-1,3-glucosyltransferase	B3GALTL	1.5	2.3	1.7	1.6	0.8	0.1	2.2	-0.6	1	1
L8AXK3	PIG IgG heavy chain	IGHG	2.3	1.8	1.5	1.4	0.5	-0.3	0.1	0.3	2	2

Q96GE4	HUMAN Centrosomal protein of 95 kDa	CEP95	0.8	2.5	2.8	0.9	2.0	0.5	0.0	-0.1	1	2
L8B0U3	PIG IgG heavy chain	IGHG	1.8	1.8	1.8	1.6	-0.2	0.4	2.8	1.5	3	2
Q8MI72	PIG Complement regulator factor H	FH	0.9	2.3	2.2	1.5	-0.1	-0.1	0.4	0.6	5	1
A2SW51	PIG Monocyte differentiation antigen CD14	CD14	1.6	2.5	1.7	1.2	0.9	0.9	0.5	-0.1	2	1
F1SLB0	PIG Uncharacterized protein	BFSP2	-0.4	2.2	3.7	1.4	-0.3	-1.1	1.9	0.8	1	1
I3LDM5	PIG Uncharacterized protein	EXOC1	2.3	1.9	1.6	1.0	1.1	-0.5	1.1	-0.3	1	1
F1SFI7	PIG Alpha-2-HS-glycoprotein (Fragment)	AHSG	1.9	1.8	1.5	1.7	2.0	0.2	1.4	0.2	1	1
Q9GKQ6	PIG Biglycan (Fragments)	BGN	1.0	1.0	1.6	3.2	1.7	1.6	1.4	-0.3	8	3
F1RZ79	PIG Uncharacterized protein	TBC1D16	1.6	1.4	2.0	1.8	0.3	0.2	0.5	0.5	1	1
F1RVD9	PIG Uncharacterized protein	COQ2	3.2	2.6	0.6	0.3	3.4	4.9	2.1	-0.8	1	1
P80229	PIG Leukocyte elastase inhibitor	SERPINB1	1.1	2.5	2.2	0.9	1.9	1.3	0.9	0.2	16	3
F1RRE1	PIG Uncharacterized protein (Fragment)	CMTM6	1.5	1.7	2.1	1.4	0.7	-0.3	-0.6	0.0	1	1
F1SG75	PIG Uncharacterized protein	SSPN	1.2	1.7	1.3	2.4	0.6	0.8	0.4	-0.4	1	2
G8ENL3	PIG Taf15	TAF15	1.7	0.9	2.2	1.9	2.6	3.6	1.3	0.8	1	1
F1S8B0	PIG Uncharacterized protein	DDRGK1	0.3	2.7	2.6	0.9	0.7	0.5	1.0	1.5	2	1
Q9P219	HUMAN Protein Daple	CCDC88C	1.6	1.8	1.3	1.9	0.6	0.3	0.4	-0.5	1	1
F1SAG1	PIG Uncharacterized protein	TTLL7	2.7	1.6	1.3	1.0	-2.3	-0.7	-0.6	-0.3	2	1
F1RYS0	PIG Uncharacterized protein (Fragment)	FRAS1	1.8	1.6	1.7	1.4	-0.5	-0.5	0.3	-0.6	1	1
I3LQ79	PIG Uncharacterized protein	MVP	1.7	1.5	1.7	1.5	1.7	3.2	2.1	3.8	25	7
I3LTB8	PIG Uncharacterized protein	NA	1.9	1.8	1.7	1.1	-1.6	-2.4	0.5	1.0	2	1
F1S4I1	PIG Uncharacterized protein	NA	1.0	1.5	1.8	2.1	0.0	-0.2	-0.4	-0.3	4	1
P02768	HUMAN Serum albumin	ALB	1.8	2.3	1.8	0.6	0.7	-1.2	3.5	-2.5	4	1
F1RMI9	PIG Uncharacterized protein	MAN2A2	0.0	3.1	2.6	0.7	0.5	-0.2	0.3	-0.3	1	3
K7GQB8	PIG Uncharacterized protein	CP	1.4	1.2	1.8	1.9	0.2	-0.2	4.2	2.9	31	20
I3LUK8	PIG Uncharacterized protein	NA	1.6	2.0	1.8	1.1	0.7	0.1	-0.2	1.0	1	1
P60660	HUMAN Myosin light polypeptide 6	MYL6	0.6	1.4	1.7	2.6	3.1	1.2	0.3	-1.9	8	4
P50390	PIG Transthyretin	TTR	2.2	1.8	1.4	1.0	1.1	-1.6	1.8	-1.3	7	4
F2Z5B1	PIG Leukocyte elastase inhibitor	SERPINB1	1.2	2.3	2.0	0.9	2.0	1.8	0.7	0.0	1	1
F1SPP8	PIG Uncharacterized protein (Fragment)	CKAP4	0.9	1.3	2.0	2.2	0.0	0.2	1.0	0.8	18	6
L8B0W4	PIG IgG heavy chain	IGHG	1.8	2.1	1.7	0.7	0.5	-0.9	1.7	0.4	1	1
F1SVB0	PIG Uncharacterized protein	CAPG	0.9	1.0	2.2	2.2	0.7	0.6	0.8	0.6	9	1
Q00613	HUMAN Heat shock factor protein 1	HSF1	-0.2	3.1	2.8	0.5	1.2	1.1	1.6	0.3	1	1
I3LMU6	PIG Uncharacterized protein (Fragment)	RCN3	0.9	1.2	1.8	2.4	0.7	0.0	-0.7	0.2	8	1
F1SM61	PIG Uncharacterized protein	FBLN1	1.2	1.5	1.5	2.0	0.6	0.0	0.7	-0.7	4	1
F1S1A9	PIG Uncharacterized protein	APOA2	1.2	1.7	1.9	1.5	0.5	0.0	1.0	2.3	3	1
F1SC20	PIG Alpha-1B-glycoprotein	A1BG	1.9	1.6	1.2	1.5	1.3	0.9	1.9	0.3	13	11
F1RK02	PIG Uncharacterized protein (Fragment)	LCP1	0.4	1.2	2.2	2.4	1.6	0.8	-0.3	-0.1	19	4
F1SHB1	PIG Uncharacterized protein	PRPF40B	2.5	1.7	1.3	0.7	2.9	3.1	3.0	2.0	1	1
F1RSJ8	PIG Uncharacterized protein	FTSJ3	2.0	1.7	1.3	1.2	0.4	-0.3	0.3	-0.7	1	1
F1SUG8	PIG Uncharacterized protein	DDX50	1.4	2.3	1.4	1.1	-0.7	-1.8	-1.1	0.0	1	1

F1SJW8	PIG Uncharacterized protein	SERPING1	1.3	1.6	1.8	1.5	-0.1	1.2	0.7	-0.4	1	1
Q29268	PIG Syntaxin-binding protein 2 (Fragment)	STXBP2	1.7	2.0	1.6	0.8	-0.2	0.3	-0.9	0.1	1	1
P16293	PIG Coagulation factor IX (Fragment)	F9	2.0	1.7	1.3	1.1	0.6	0.3	0.0	-0.8	2	2
F1SR81	PIG Uncharacterized protein	STK16	7.3	0.2	-0.6	-0.8	7.5	1.1	0.2	-0.4	1	1
A1Y2K1	PIG Tyrosine-protein kinase Fyn	FYN	2.3	1.2	0.9	1.7	-0.8	0.0	2.0	1.7	1	1
I3LHF5	PIG Uncharacterized protein	KCND1	2.0	1.8	1.3	1.0	-0.9	-1.2	-0.3	-0.7	1	1
I3LS26	PIG Saposin-B-Val	PSAP	-0.1	1.3	2.5	2.3	2.2	0.9	0.3	-1.1	6	2
L8B0T2	PIG IgG heavy chain	IGHG	0.4	2.3	1.9	1.4	-1.0	1.2	5.1	1.2	2	2
K7GQL2	PIG Coagulation factor XIII, A1 polypeptide	F13A1	1.0	1.6	2.2	1.2	-0.1	-0.5	-1.4	-2.8	3	1
Q0PM28	PIG Pigment epithelium-derived factor	NA	1.4	1.8	1.4	1.5	0.7	1.3	0.2	0.2	13	4
Q6DUB7	PIG Stathmin	STMN1	1.2	1.6	1.6	1.7	1.5	0.5	0.4	0.3	6	1
Q9ULI4	HUMAN Kinesin-like protein KIF26A	KIF26A	1.0	1.7	2.0	1.3	-0.4	-1.3	-1.1	-1.4	1	2
Q2TAY7	HUMAN WD40 repeat-containing protein SMU1	SMU1	1.4	1.3	1.3	1.9	1.4	2.6	0.6	0.2	3	1
L8AXL9	PIG IgG heavy chain	IGHG	1.3	1.5	1.9	1.2	-1.2	-2.5	2.4	0.0	4	4
F1SMJ6	PIG Uncharacterized protein (Fragment)	C9	0.8	1.8	2.0	1.4	1.5	1.0	0.3	1.9	7	1
Q68RU1	PIG Ovarian and testicular apolipoprotein N	ApoN	0.8	1.9	2.2	1.0	-0.2	-0.9	-0.4	-0.4	5	2
Q96SA9	HUMAN Anti-streptococcal/anti-myosin immunoglobulin kappa light chain variable region (Fragment)	NA	1.9	2.2	1.1	0.7	1.4	1.9	0.9	-0.9	1	1
F1SQE6	PIG Uncharacterized protein (Fragment)	ARPC4	0.6	1.0	2.0	2.4	-0.2	0.5	0.1	0.5	5	5
P29700	PIG Alpha-2-HS-glycoprotein (Fragment)	AHSG	2.1	1.6	1.1	1.1	0.4	-1.0	-0.1	-0.4	4	2
I3L9M7	PIG Uncharacterized protein	NA	0.5	0.9	1.9	2.6	1.1	1.2	-0.1	-0.6	6	1
I3LN07	PIG Uncharacterized protein (Fragment)	UHRF1BP1L	3.5	0.9	0.5	0.9	6.1	2.2	0.3	-0.2	2	1
I3LI44	PIG Uncharacterized protein (Fragment)	COTL1	0.3	0.9	2.2	2.4	-0.4	0.0	0.1	0.4	8	2
Q29052	PIG Inter-alpha-trypsin inhibitor heavy chain H1	ITIH1	1.6	1.6	1.5	1.1	-0.1	-1.2	0.5	0.8	23	12
P00795	PIG Cathepsin D	CTSD	0.6	0.1	1.9	3.1	0.3	0.0	-0.2	-0.2	12	8
K9IVW2	PIG Integrin alpha-X	ITGAX	1.9	1.9	1.3	0.7	-0.8	-0.6	0.2	0.3	1	1
F1S682	PIG Sulfhydryl oxidase	QSOX1	1.3	1.8	1.6	1.2	0.5	0.5	0.7	1.6	11	2
I3LUE0	PIG Uncharacterized protein (Fragment)	CCDC112	5.8	1.2	-0.7	-0.5	8.6	7.2	-0.1	0.3	1	1
F1SIX7	PIG Uncharacterized protein	LOC100520931	2.0	1.7	1.4	0.7	-0.5	-0.5	-0.9	0.0	2	2
F1SU68	PIG Uncharacterized protein	TTC18	2.1	2.5	0.8	0.4	0.2	0.1	0.0	-0.1	1	1
K7GQ48	PIG Uncharacterized protein	A2M	2.0	1.7	1.3	0.7	1.2	0.3	2.1	-0.1	6	3
F1RQW6	PIG Complement factor B	BF	1.2	1.1	1.6	1.7	-0.6	-1.1	1.0	0.7	23	8
I3LGI9	PIG Uncharacterized protein	GLB1	1.0	1.9	2.2	0.7	0.7	1.2	-0.6	-0.9	1	1
F1RJ93	PIG Uncharacterized protein (Fragment)	TAGLN2	0.4	1.0	1.9	2.4	0.4	1.1	0.1	-0.1	13	7
O02668	PIG Inter-alpha-trypsin inhibitor heavy chain H2	ITIH2	1.8	1.4	1.4	1.1	0.8	0.6	1.2	0.4	23	12
B0LFE9	PIG Complement component 4	NA	1.5	1.4	1.3	1.4	0.5	-0.4	1.6	2.9	38	7
F1SP49	PIG Uncharacterized protein (Fragment)	NA	0.6	1.1	1.8	2.1	-0.6	0.3	0.5	0.3	1	1
K7GPJ3	PIG Uncharacterized protein	THBS1	1.2	1.9	1.3	1.2	0.0	-1.2	0.8	0.9	10	1
F1SH87	PIG Uncharacterized protein (Fragment)	LARP4	1.2	1.3	1.6	1.4	-3.0	-5.6	-5.5	-5.5	1	1
A8U4R4	PIG Transketolase	tkt	0.8	1.0	1.9	2.0	0.7	0.9	0.7	-0.3	21	6
F1RN76	PIG Uncharacterized protein	CD5L	0.7	1.3	2.5	1.1	0.0	-1.0	3.3	0.3	7	1

I3LV88	PIG Uncharacterized protein (Fragment)	NA	4.8	0.5	0.1	0.3	7.8	0.0	1.0	1.5	1	2
Q9Y240	HUMAN C-type lectin domain family 11 member A	CLEC11A	1.0	1.5	1.6	1.6	-2.2	-4.5	-3.9	-4.8	1	1
Q9UPU5	HUMAN Ubiquitin carboxyl-terminal hydrolase 24	USP24	1.7	1.2	1.2	1.5	-2.4	-1.3	-0.2	1.1	1	1
P02543	PIG Vimentin	VIM	0.1	0.6	1.9	3.0	0.4	1.4	0.3	-1.3	50	34
F1SJB5	PIG Annexin	ANXA1	0.5	1.5	1.9	1.7	0.9	1.2	1.5	0.3	1	1
Q719N1	PIG Spastin (Fragment)	SPAST	2.5	3.6	-0.6	0.0	4.2	6.2	0.9	0.1	1	1
Q14568	HUMAN Putative heat shock protein HSP 90-alpha A2	HSP90AA2	-0.1	1.4	2.7	1.5	1.8	4.0	9.8	1.3	1	1
F1SS24	PIG Uncharacterized protein	FN1	0.5	0.7	1.7	2.5	-0.5	-1.6	-1.1	-0.2	63	26
I3LVF2	PIG Uncharacterized protein	PTCH1	0.6	1.3	1.7	1.8	-1.3	-0.6	0.0	0.4	1	1
Q9NUQ9	HUMAN Protein FAM49B	FAM49B	1.5	1.8	1.2	1.0	-1.6	-1.1	-0.3	1.0	3	1
I3LAJ5	PIG Uncharacterized protein (Fragment)	NA	1.5	2.4	0.9	0.6	-1.3	-0.6	-0.5	0.1	2	1
P67985	PIG 60S ribosomal protein L22	RPL22	1.0	1.5	1.7	1.2	0.0	0.5	0.9	1.0	3	3
F1RGI2	PIG Uncharacterized protein	LOC100514845	2.2	1.9	0.8	0.5	0.3	0.6	0.9	-0.8	2	2
Q9TUQ3	PIG Complement component C7	C7	1.7	1.6	1.3	0.9	0.3	2.2	3.4	1.0	7	1
F1SBK5	PIG Uncharacterized protein	ANKEF1	2.4	1.8	0.6	0.5	5.7	3.4	-0.3	0.7	1	1
B9TSP4	PIG Tenascin XB	TNXB	1.7	1.1	1.3	1.3	2.2	2.8	0.3	0.7	1	1
K9IW91	PIG GCN1 general control of amino-acid synthesis 1-like 1	GCN1L1	0.1	1.5	2.6	1.2	-1.2	-1.1	1.2	1.2	2	1
F1SKM1	PIG Uncharacterized protein	COL7A1	0.7	1.6	1.9	1.2	1.6	0.3	0.0	-1.5	3	1
F1S8P4	PIG Uncharacterized protein	AHCTF1	1.9	1.2	0.9	1.3	0.5	0.5	1.7	0.8	2	1
Q9GLP2	PIG Vitamin K-dependent protein C	PROC	1.4	1.2	1.5	1.2	-1.8	-3.1	-3.4	-2.1	7	1
I3LQH7	PIG Uncharacterized protein	BLVRB	0.6	2.4	1.9	0.4	0.0	0.8	-0.4	-1.4	8	3
F1S643	PIG Uncharacterized protein	CFH	1.3	1.3	1.5	1.2	0.8	0.7	3.0	1.4	9	2
I3LGC2	PIG Uncharacterized protein	RRBP1	0.6	0.6	1.8	2.3	2.1	2.0	0.8	0.9	36	2
I3L7A9	PIG Uncharacterized protein	NA	1.3	0.8	1.3	1.8	1.6	1.0	0.5	-0.9	1	1
Q8WMQ3	PIG CD9 antigen	CD9	0.1	1.2	2.1	1.8	-0.7	-0.8	-0.6	0.9	1	1
Q9GLP1	PIG Coagulation factor V	F5	1.1	1.5	1.8	0.9	0.7	0.6	0.6	0.5	9	1
P09493-5	HUMAN Isoform 5 of Tropomyosin alpha-1 chain	TPM1	1.0	2.2	1.2	0.9	2.7	1.1	-1.0	-1.7	1	1
Q19AZ5	PIG Antithrombin protein	NA	1.4	1.5	1.3	1.0	0.8	0.7	1.1	0.2	17	7
O95613	HUMAN Pericentrin	PCNT	-0.1	0.2	3.3	1.8	-0.3	0.8	-0.1	-0.2	1	1
F1SQW8	PIG Uncharacterized protein	ARHGDIB	0.7	1.1	1.7	1.7	1.3	2.0	0.6	-0.3	10	2
I3LU39	PIG Uncharacterized protein (Fragment)	ISG15	1.1	0.5	1.8	1.7	1.9	-0.4	3.8	6.0	3	2
F1RXY7	PIG Uncharacterized protein	TSN	2.7	1.8	0.2	0.4	2.5	1.0	0.9	-0.8	2	1
Q9NRC6	HUMAN Spectrin beta chain, non-erythrocytic 5	SPTBN5	0.6	0.9	2.2	1.5	-1.7	-1.6	-0.7	-0.3	3	3
I3LVS5	PIG Uncharacterized protein (Fragment)	NA	0.9	1.1	1.5	1.7	1.1	-0.1	0.4	0.8	5	2
F1S3G9	PIG Uncharacterized protein	LOC100154911	1.8	1.4	1.0	0.9	-0.5	-0.9	-0.3	-0.4	1	1
F1SRC8	PIG Uncharacterized protein	CLEC3B	1.1	1.8	1.0	1.3	0.4	-0.7	-0.1	-0.7	2	2
F1SQ34	PIG Uncharacterized protein	DTX3L	-1.4	1.8	2.4	2.3	-1.4	2.0	-0.1	0.4	1	1
I3LNI2	PIG Uncharacterized protein (Fragment)	TFG	1.9	1.2	1.1	0.9	3.7	1.6	0.6	-0.3	2	2
Q08094	PIG Calponin-2 (Fragment)	CNN2	0.2	0.6	1.8	2.5	0.9	0.5	0.9	1.0	2	1
F1SLA7	PIG Uncharacterized protein	SRPRB	3.4	1.5	0.1	0.0	0.8	0.7	-1.0	-0.1	1	2

P80171	PIG LIM and SH3 domain protein 1 (Fragment)	LASP1	0.5	1.1	1.2	2.3	0.2	0.1	-0.2	-0.6	1	1
Q99542	HUMAN Matrix metalloproteinase-19	MMP19	3.7	1.1	0.0	0.4	6.5	5.0	0.9	0.4	1	1
F1S902	PIG Uncharacterized protein	COMP	-0.2	0.3	1.8	3.1	-1.2	-0.1	-0.4	0.0	1	1
I3LBF7	PIG Uncharacterized protein (Fragment)	LOC733637	0.7	1.1	1.5	1.8	1.5	0.9	-0.5	-0.9	2	2
I3LS00	PIG Uncharacterized protein	PABPC1	1.2	1.4	1.2	1.2	0.3	0.4	1.2	0.7	8	3
I3LM05	PIG Uncharacterized protein	NA	0.8	1.6	1.3	1.3	-1.1	-0.7	-0.6	-0.4	3	1
H9LBP0	PIG Serpin peptidase inhibitor clade D member 1 (Fragment)	SERPIND1	0.7	1.8	1.9	0.6	2.3	1.1	-0.1	0.1	2	2
I3L9S0	PIG Uncharacterized protein (Fragment)	OBSL1	0.8	1.4	1.6	1.3	-0.7	-1.0	-0.5	-0.2	3	3
K7GRI3	PIG Uncharacterized protein (Fragment)	CP	1.6	0.9	1.2	1.4	0.7	0.2	4.6	2.9	11	5
G8G223	PIG Coronin	coro1a	0.4	1.6	1.8	1.2	1.2	0.1	0.3	-0.9	10	1
F1RJL0	PIG Uncharacterized protein	WBSCR16	4.3	0.9	0.1	-0.2	3.2	0.5	0.2	1.2	1	1
F1SFI6	PIG Uncharacterized protein (Fragment)	FETUB	2.2	1.5	0.8	0.5	1.1	0.3	0.1	0.1	10	4
P61421	HUMAN V-type proton ATPase subunit d 1	ATP6V0D1	1.0	1.5	1.2	1.3	2.0	0.6	0.3	-0.4	3	1
A0SEH3	PIG Complement component C8G	LOC100037955	1.5	1.6	1.2	0.7	3.6	3.0	1.3	1.2	5	1
F1RGK5	PIG Tropomyosin alpha-3 chain	TPM3	1.1	1.4	1.1	1.4	1.8	0.9	-0.1	0.2	6	2
F1SH32	PIG Uncharacterized protein	GOLIM4	0.9	0.7	1.4	2.0	0.4	0.9	1.7	3.3	1	1
F1RQW2	PIG Uncharacterized protein	C4	1.6	1.5	0.9	1.0	1.1	0.2	1.0	1.2	2	2
F1RHI6	PIG Uncharacterized protein	NES	0.3	0.9	1.9	1.8	1.5	1.2	-0.4	-1.1	59	15
P27594	PIG Interferon-induced GTP-binding protein Mx1	MX1	1.2	0.6	1.5	1.7	1.4	-0.8	0.3	0.9	19	3
F1SV88	PIG Uncharacterized protein	LOC100518914	1.0	1.0	1.4	1.5	0.3	0.6	0.4	1.1	2	1
I3LM09	PIG Uncharacterized protein (Fragment)	HCN2	1.3	1.4	1.1	1.2	-0.5	0.2	1.7	1.8	1	1
K7GPW1	PIG Uncharacterized protein	CFI	1.4	1.2	1.3	1.1	0.9	-0.9	1.5	-0.4	4	2
I3LLP5	PIG Uncharacterized protein (Fragment)	GIMAP4	0.3	0.5	2.1	2.0	1.4	1.5	0.9	1.3	8	4
P50454	HUMAN Serpin H1	SERPINH1	0.4	0.4	1.7	2.4	0.0	0.5	-0.1	0.7	10	8
Q860V0	PIG MHC class I antigen	SLA-3	1.2	1.4	0.9	1.5	-1.4	-1.3	-1.0	0.5	1	1
A0PFK4	PIG F-actin capping protein subunit beta 2	CP beta2	0.4	0.5	1.7	2.3	-1.4	-0.7	0.1	-1.0	1	1
F1SRG2	PIG NADH dehydrogenase [ubiquinone] 1 alpha subcomplex subunit 6	NDUFA6	0.6	2.5	0.5	1.3	1.9	7.7	8.1	17.9	1	1
Q9UHQ9	HUMAN NADH-cytochrome b5 reductase 1	CYB5R1	1.0	0.8	1.7	1.4	0.3	0.6	0.9	0.0	2	1
I3LRJ6	PIG Uncharacterized protein	CKAP5	1.0	1.3	1.4	1.2	-2.3	-0.4	1.8	3.9	2	1
F1SUM3	PIG Uncharacterized protein (Fragment)	SERPINH1	-0.4	0.4	2.1	2.8	-0.7	-0.3	0.4	1.5	7	4
O15511	HUMAN Actin-related protein 2/3 complex subunit 5	ARPC5	0.3	0.9	1.9	1.7	-0.1	-1.0	-0.4	-0.5	4	1
P16415	HUMAN Zinc finger protein 823	ZNF823	2.8	2.6	-0.1	-0.5	5.5	2.9	1.3	1.2	1	1
K9J6H8	PIG Alpha-2-macroglobulin	A2M	1.7	2.0	1.3	-0.1	0.7	0.4	3.5	1.0	3	1
Q4KWH8	HUMAN 1-phosphatidylinositol 4,5-bisphosphate phosphodiesterase eta-1	PLCH1	0.3	2.5	2.2	-0.1	-0.1	0.2	-0.8	0.9	1	2
F1RVR2	PIG Uncharacterized protein	DNAH8	1.0	1.2	1.4	1.2	0.3	-0.5	0.3	0.3	1	1
Q9Y3B4	HUMAN Pre-mRNA branch site protein p14	SF3B14	1.5	1.6	1.0	0.7	1.0	0.8	-0.2	-0.5	2	1
F1S3B0	PIG Uncharacterized protein	NA	1.7	1.2	1.1	0.7	-0.1	-0.6	0.0	0.9	1	1
F1SNL7	PIG Uncharacterized protein	CNDP2	0.5	0.5	1.7	2.1	1.7	1.1	0.3	0.2	6	4
F1SCV8	PIG Uncharacterized protein (Fragment)	NA	1.1	1.4	1.3	1.0	1.0	0.7	0.0	-1.0	25	3
Q8WWQ8	HUMAN Stabilin-2	STAB2	0.5	0.8	1.6	1.8	0.2	0.2	2.7	2.5	1	1

F1S924	PIG Uncharacterized protein	CCDC124	0.5	1.9	1.7	0.7	1.6	0.5	0.6	0.9	1	1
P04229	HUMAN HLA class II histocompatibility antigen, DRB1-1 beta chain	HLA-DRB1	1.2	0.8	1.7	1.0	0.3	0.2	0.3	-0.6	1	1
O46409	PIG Apolipoprotein A-IV	APOA4	1.6	2.0	0.9	0.4	1.8	-0.8	-3.8	-1.8	31	6
F1RY21	PIG Uncharacterized protein	NA	1.4	1.8	1.0	0.5	0.2	0.8	0.3	0.7	1	1
Q29545	PIG Inhibitor of carbonic anhydrase	ICA	1.9	1.0	1.0	0.8	1.4	0.4	2.9	-0.8	8	4
I3LG67	PIG Uncharacterized protein	MARCKS	0.2	0.7	1.7	2.1	0.5	-0.2	1.6	0.7	4	3
I3LKZ1	PIG Uncharacterized protein	NA	0.8	1.0	1.5	1.4	0.8	-0.1	-1.2	-0.1	16	3
P62910	HUMAN 60S ribosomal protein L32	RPL32	-0.1	1.3	2.2	1.4	0.0	0.2	-0.5	-0.7	2	2
K7GM22	PIG Uncharacterized protein	NAA10	1.6	0.8	1.3	1.0	0.4	0.9	2.0	1.8	1	1
Q29568	PIG Phosphopyruvate hydratase (Fragment)	NA	1.2	0.9	1.2	1.5	0.9	1.0	-0.4	-0.9	1	1
I3LNA4	PIG Uncharacterized protein	LOC100621383	2.3	0.8	0.7	0.9	0.7	1.5	0.0	-1.8	2	1
O14746	HUMAN Telomerase reverse transcriptase	TERT	1.6	1.3	1.1	0.8	-0.1	-0.5	1.0	-0.5	3	1
I3LDD2	PIG Uncharacterized protein	NOP58	0.5	1.9	1.7	0.6	0.7	1.3	-1.4	-1.8	1	1
I3LLD5	PIG Uncharacterized protein (Fragment)	NA	0.8	0.7	1.7	1.4	0.1	0.9	-0.9	-1.0	8	5
I3LK24	PIG Uncharacterized protein	AP2A1	1.3	1.1	1.1	1.2	1.2	0.5	0.2	-0.4	8	5
Q2MJK3	PIG GTPase NRas	NRAS	2.2	2.1	0.3	0.1	0.6	1.2	0.5	-0.5	1	1
F1SGG9	PIG Uncharacterized protein (Fragment)	LOC100737483	3.3	1.5	0.0	-0.2	1.6	0.7	-2.0	1.9	1	1
A1KZ92	HUMAN Peroxidasin-like protein	PXDNL	1.0	1.5	0.7	1.4	-0.5	-2.0	-0.2	-2.4	1	1
P23396	HUMAN 40S ribosomal protein S3	RPS3	0.8	0.5	1.5	1.9	0.5	0.6	0.0	0.3	6	6
Q14241	HUMAN Transcription elongation factor B polypeptide 3	TCEB3	0.0	1.6	2.0	1.0	-0.6	1.0	1.6	1.7	1	1
F1S895	PIG Uncharacterized protein	LOC100624528	0.9	1.1	1.7	0.9	0.4	1.4	0.9	0.2	2	3
Q4QZ00	PIG Elongation factor 1-alpha 1 (Fragment)	EF1A1	-0.7	0.0	2.2	3.1	-0.5	0.3	1.2	1.5	8	9
F2Z5I2	PIG Uncharacterized protein	RBM39	1.4	1.8	1.0	0.5	-0.2	0.3	0.5	0.6	2	2
I3LV13	PIG Uncharacterized protein (Fragment)	PTMA	-0.7	1.7	2.3	1.4	3.9	2.0	0.7	-3.7	5	2
F1SML4	PIG Uncharacterized protein	SND1	1.2	0.7	1.3	1.4	1.4	1.4	1.1	1.0	10	3
F1SPT0	PIG Ubiquitin carboxyl-terminal hydrolase	USP4	2.3	1.1	0.8	0.3	2.5	2.2	2.0	0.9	1	1
Q5NV91	HUMAN V2-19 protein (Fragment)	IGLV3-27	1.0	2.2	1.3	0.1	-0.7	-2.6	2.4	-1.8	1	1
I3LJX7	PIG Uncharacterized protein	TOR1AIP2	2.0	2.2	0.3	0.0	3.9	6.0	1.5	-0.7	1	1
F1SQY2	PIG Uncharacterized protein (Fragment)	DERA	1.6	1.4	1.0	0.5	0.7	1.1	1.2	0.7	1	1
F1SLL2	PIG Uncharacterized protein (Fragment)	IL31RA	1.1	0.7	1.2	1.4	-0.3	0.0	0.6	1.0	1	1
Q29290	PIG Cystatin-B	CSTB	-0.1	0.1	2.0	2.5	0.1	-0.7	-0.9	-2.0	3	1
O60661	HUMAN Alpha myosin heavy chain (Fragment)	NA	2.6	2.4	-0.1	-0.5	-1.2	-1.7	-0.5	-0.6	1	1
K7GMW0	PIG Uncharacterized protein (Fragment)	PTPRC	0.4	1.1	1.3	1.6	-0.8	-0.9	0.2	1.0	3	1
F1SCC9	PIG Uncharacterized protein (Fragment)	NA	1.2	2.1	0.7	0.4	-1.0	0.8	0.4	-2.5	5	4
F2Y8R2	PIG Transmembrane and coiled coil domain protein 3 (Fragment)	Tmcc3	1.3	0.8	1.3	1.0	-1.0	-1.5	-1.7	-1.3	1	1
P62495	HUMAN Eukaryotic peptide chain release factor subunit 1	ETF1	1.2	0.9	1.1	1.2	3.4	1.9	0.7	-0.7	4	1
F1RWT2	PIG Uncharacterized protein	PLS3	0.7	0.9	1.2	1.6	0.4	0.1	0.0	0.3	21	7
A2I7K3	PIG MHC class I antigen	SLA-1	0.6	0.5	1.8	1.4	0.2	1.1	2.9	3.2	1	1
P84157-2	HUMAN Isoform 2 of Matrix-remodeling-associated protein 7	MXRA7	0.8	0.9	1.0	1.7	2.2	1.3	2.6	0.4	1	1
D2K6G0	PIG Thioredoxin reductase 2	NA	1.9	0.7	0.8	1.0	1.9	1.1	0.4	-1.3	1	2

Q7YSG7	PIG Nitric oxide synthase	NA	0.5	1.0	0.9	2.0	-0.7	-0.8	0.8	0.5	1	1
C4NF76	PIG Bone marrow stromal cell antigen 2	BST2	0.7	1.3	1.1	1.3	0.8	0.9	0.5	3.8	2	1
P79309	PIG Tropomyosin TM30-pl (Fragment)	NA	-0.8	0.6	1.5	3.1	0.9	0.6	-1.0	-1.7	3	1
F6PVJ7	PIG Nucleoside diphosphate kinase	NME1	0.9	0.8	1.2	1.5	0.2	0.3	-0.3	-0.5	4	1
F1SUQ3	PIG Uncharacterized protein	EMC1	3.2	1.5	0.1	-0.4	-0.6	-0.5	-0.3	-0.1	1	1
Q06A96	PIG RU2B	NA	2.0	1.0	0.6	0.8	1.7	-0.4	-1.1	-0.6	2	1
A0PFK5	PIG Capping protein (Actin filament) muscle Z-line, alpha 1	CP alpha1	0.6	0.7	1.5	1.5	0.5	0.5	0.9	1.1	6	4
F1SL54	PIG Uncharacterized protein	COPB2	1.0	0.8	1.1	1.4	1.2	2.7	0.8	0.6	9	3
Q6XQN2	PIG Nicotinate phosphoribosyltransferase-like protein (Fragment)	NA	1.3	1.3	1.0	0.7	-0.3	-1.2	2.3	-0.4	2	1
Q8NHP7	HUMAN Exonuclease 3'-5' domain-containing protein 1	EXD1	2.4	1.0	0.5	0.4	2.3	0.1	-0.1	-0.1	1	1
P14477	PIG Fibrinogen beta chain (Fragment)	FGB	-0.2	1.5	1.0	1.9	0.2	1.1	3.3	5.0	1	1
C6K7I1	PIG Importin subunit alpha	NA	0.9	0.9	1.3	1.1	1.0	1.0	-0.3	-0.6	6	2
F1SKN5	PIG Eukaryotic translation initiation factor 3 subunit L	EIF3L	1.2	0.7	1.1	1.3	-0.4	-0.6	-0.1	0.7	6	2
F1SQ36	PIG Uncharacterized protein	PARP14	0.1	0.8	1.8	1.6	-0.5	0.5	3.1	3.3	1	1
Q32M45	HUMAN Anoctamin-4	ANO4	1.9	1.4	0.6	0.3	1.2	1.4	0.8	-1.6	1	1
F1RKA7	PIG Uncharacterized protein (Fragment)	OAS2	1.6	1.5	0.7	0.4	1.7	3.3	0.8	0.2	1	1
P28491	PIG Calreticulin	CALR	-0.6	0.8	1.9	2.2	0.7	0.5	-1.2	-1.6	22	12
Q29308	PIG 40S ribosomal protein S19 (Fragment)	RPS19	0.1	0.6	1.7	1.8	-0.2	0.7	0.5	0.6	9	8
I3LGH6	PIG Uncharacterized protein	NA	1.1	0.9	1.1	1.0	1.5	1.8	0.7	-0.5	10	3
P02675	HUMAN Fibrinogen beta chain	FGB	3.8	0.6	0.0	-0.2	0.5	-0.7	-0.6	-1.1	2	1
Q13885	HUMAN Tubulin beta-2A chain	TUBB2A	0.9	0.4	1.1	1.8	1.5	1.9	-0.3	0.6	2	2
M3UYZ6	PIG ATPase, class VI, type 11A tv2 (Fragment)	ATP11A	0.1	1.0	1.9	1.1	-0.3	-0.6	0.5	0.8	1	1
Q9Y3A6-2	HUMAN Isoform 2 of Transmembrane emp24 domain-containing protein 5	TMED5	1.1	1.1	0.8	1.3	0.0	-0.7	0.6	-1.3	1	1
O60536	HUMAN Antigen NY-CO-43	NA	1.2	0.5	1.2	1.2	1.0	0.2	0.7	0.0	1	1
F1RK53	PIG Coronin	LOC100513346	1.5	1.5	0.6	0.5	3.0	9.1	2.1	-1.1	6	1
P45846	PIG Dermatopontin	DPT	1.0	1.1	0.6	1.4	1.1	0.7	0.1	-0.2	2	1
F1S584	PIG Choline transporter-like protein 2	SLC44A2	3.3	0.6	0.0	0.2	0.5	-0.2	0.7	0.8	1	1
Q2VPJ9-2	HUMAN Isoform 2 of Leucine-rich repeat-containing protein FAM211B	FAM211B	2.2	1.1	0.4	0.4	0.4	0.0	1.1	-0.4	1	1
F1SA35	PIG Uncharacterized protein	LOC100737421	1.0	0.8	1.0	1.4	-0.3	0.8	0.0	-0.2	3	3
F1SNY2	PIG 60S ribosomal protein L3	RPL3	-0.1	0.1	1.7	2.4	-0.3	0.0	0.8	0.6	11	5
P80230	PIG Enhancer of rudimentary homolog (Fragment)	ERH	0.9	0.4	1.3	1.6	0.3	0.2	1.1	0.0	2	2
Q9BSI4	HUMAN TERF1-interacting nuclear factor 2	TINF2	0.0	1.4	2.0	0.7	0.1	0.1	2.3	1.9	1	2
P34935	PIG 78 kDa glucose-regulated protein (Fragment)	HSPA5	-0.2	1.1	1.5	1.8	0.9	0.9	0.0	-0.4	8	8
F1SCY1	PIG Uncharacterized protein (Fragment)	IFIT1	0.9	0.6	1.3	1.3	1.0	0.2	0.2	0.5	7	3
F1SKJ1	PIG Uncharacterized protein	MYH9	0.4	0.6	1.2	2.0	-0.6	-0.4	-0.6	0.3	70	40
K7GLW0	PIG Uncharacterized protein	GK	1.5	1.6	0.9	0.2	-0.4	-0.7	-0.3	-0.7	3	6
I3LUC8	PIG Uncharacterized protein	LOC100154783	0.2	0.5	1.3	2.1	-0.6	-0.4	-0.2	0.6	10	8
Q92734	HUMAN Protein TFG	TFG	0.8	0.9	1.3	1.1	1.5	0.9	0.7	-1.2	3	3
P67936	HUMAN Tropomyosin alpha-4 chain	TPM4	0.3	0.9	0.9	2.1	1.5	1.2	0.1	-0.3	7	1
F1RUW3	PIG Uncharacterized protein	TOMM6	2.2	1.0	0.5	0.4	2.1	2.5	1.6	-0.6	2	1

I3L4N8	HUMAN Actin, cytoplasmic 2, N-terminally processed (Fragment)	ACTG1	1.8	2.3	0.2	-0.2	2.1	3.4	-0.3	-2.0	1	1
A7KZR3	PIG Cytochrome P450 (Fragment)	CYP8A1	1.2	0.4	0.8	1.6	0.2	-0.5	0.1	0.4	3	2
F1SHD6	PIG Uncharacterized protein	EEF1B2	0.1	0.8	1.6	1.5	0.6	1.2	-0.6	-0.6	5	1
P62306	HUMAN Small nuclear ribonucleoprotein F	SNRPF	0.7	0.6	1.5	1.3	0.8	0.4	1.6	1.7	2	2
I3LJ42	PIG Guanine nucleotide-binding protein subunit gamma	GNG2	0.7	1.8	1.4	0.1	-0.2	0.2	2.0	1.9	2	1
Q000H8	PIG Glutathione S-transferase mu 2 (Fragment)	NA	1.9	1.8	0.1	0.2	0.3	0.6	-0.8	-0.7	2	2
I3LPG3	PIG UDP-glucose ceramide glucosyltransferase	UGCG	-0.6	2.2	2.1	0.3	-1.0	-1.0	-0.3	-1.0	1	1
F1RVP8	PIG Uncharacterized protein	TCIRG1	0.8	1.1	0.7	1.4	-1.7	-0.8	-0.7	-1.2	1	1
C3RZ98	PIG Protein arginine methyltransferase 5	PRMT5	1.2	1.3	1.0	0.5	0.3	4.6	0.7	0.2	2	1
Q6Q2J0	PIG Amine oxidase [flavin-containing] A	MAOA	1.9	1.0	0.5	0.6	0.1	0.1	-1.7	-0.7	2	2
I3LK59	PIG Enolase	ENO1	-0.2	0.7	1.6	1.9	-0.7	-0.6	0.2	-0.2	15	9
P62318	HUMAN Small nuclear ribonucleoprotein Sm D3	SNRPD3	0.4	0.9	1.2	1.4	0.0	0.4	-0.4	-0.3	3	2
U5IT50	HUMAN MHC class I antigen (Fragment)	HLA-A	-0.9	2.3	2.9	-0.3	-1.1	-0.8	0.5	1.1	1	1
F1SR76	PIG Uncharacterized protein	DNPEP	1.2	1.2	0.9	0.7	1.7	2.0	1.2	0.6	1	2
F1S3H9	PIG Uncharacterized protein (Fragment)	NA	0.9	1.5	1.4	0.1	-0.9	-0.8	-1.1	-0.1	1	1
O00291	HUMAN Huntingtin-interacting protein 1	HIP1	1.0	1.5	1.1	0.4	0.3	0.5	-1.6	-0.9	1	1
Q8IUE6	HUMAN Histone H2A type 2-B	HIST2H2AB	1.4	1.5	0.7	0.4	-0.1	0.1	0.7	0.0	1	1
P00339	PIG L-lactate dehydrogenase A chain	LDHA	-0.2	0.0	2.0	2.2	0.7	0.5	2.7	1.9	19	14
K7GQP1	PIG Uncharacterized protein	LOC100518277	1.9	1.2	0.8	0.1	1.2	1.0	0.8	-0.8	1	1
G9JM01	PIG Tumor susceptibility 101 protein	NA	2.7	0.6	0.4	0.2	0.5	2.0	-1.4	-0.3	3	1
Q7Z2K5	HUMAN Beta-globin (Fragment)	HBB	1.6	0.9	0.9	0.6	0.9	0.5	2.2	0.9	1	1
I3LN84	PIG Uncharacterized protein	LXN	0.9	1.4	0.7	1.0	1.8	1.2	0.4	-1.7	3	1
I3LQF8	PIG Uncharacterized protein	YBX3	0.3	0.5	1.4	1.8	-0.1	0.2	0.5	2.1	1	1
F1RZ06	PIG Uncharacterized protein (Fragment)	MXRA5	1.1	0.7	0.9	1.3	-0.9	-0.7	0.2	-0.5	11	2
A5A8Y6	PIG 1-acylglycerol-3-phosphate O-acyltransferase 1 (Acetoacetly Coenzyme A thiolase) (Fragment)	AGPAT1	2.0	1.3	0.5	0.1	2.2	2.0	0.0	0.5	1	1
F1RMJ4	PIG Uncharacterized protein	IQGAP1	1.0	0.8	0.9	1.2	1.0	0.2	-1.0	-1.1	11	4
K7GMF6	PIG Uncharacterized protein	CD97	0.8	2.3	-0.2	1.0	0.7	-0.6	3.9	2.9	1	1
F1SAD9	PIG Uncharacterized protein	PDIA4	0.3	0.7	1.4	1.5	0.3	0.5	-0.1	1.0	20	13
E1CAJ6	PIG Protein disulfide isomerase P5	pdi-p5	0.4	0.6	1.2	1.8	0.8	1.7	0.5	-0.1	16	6
A4US67	PIG Paraoxonase	PON1	1.5	1.4	1.0	0.1	1.1	0.1	-1.7	-2.2	6	1
F1S5P2	PIG Uncharacterized protein	SLK	1.4	1.0	0.7	0.8	2.2	0.2	-0.6	1.1	5	4
Q9UHD9	HUMAN Ubiquilin-2	UBQLN2	0.7	1.3	1.2	0.6	0.2	0.2	1.1	0.9	1	1
Q9Y2W3	HUMAN Proton-associated sugar transporter A	SLC45A1	2.9	1.5	-0.2	-0.3	1.9	0.1	-0.8	-0.6	1	2
Q59FH9	HUMAN Zinc finger protein 42 isoform 2 variant (Fragment)	NA	-1.1	4.0	1.2	-0.2	-1.9	0.2	-1.7	-2.1	1	1
K9IVP5	PIG N-myosin-9	MYH9	0.3	0.4	1.2	2.0	0.0	-0.4	-0.8	0.6	14	9
Q29376	PIG Calmodulin (Fragment)	NA	-0.4	1.5	1.3	1.4	1.4	0.4	-1.0	-4.2	2	1
P56941	PIG Niemann-Pick C1 protein	NPC1	3.1	0.9	0.1	-0.2	3.7	-0.3	0.2	-0.2	2	1
B5APU4	PIG Actin-related protein 3-like protein	NA	0.4	0.8	1.3	1.4	-0.1	0.4	-1.0	-0.2	9	5
I3L810	PIG Uncharacterized protein	HNMT	0.3	0.4	1.5	1.7	-0.3	-0.3	-2.2	-1.3	5	1
Q29329	PIG Small nuclear ribonucleoprotein Sm D2 (Fragment)	SNRPD2	0.9	0.7	1.0	1.4	1.1	0.2	0.4	-0.4	2	1

O15083	HUMAN ERC protein 2	ERC2	0.9	0.7	1.1	1.2	-0.5	-0.2	-0.9	-1.1	1	1
I3LGB3	PIG Uncharacterized protein (Fragment)	LSM2	1.5	1.1	1.0	0.2	2.8	4.4	5.3	-0.4	1	1
Q8N715	HUMAN Uncharacterized protein C1orf65	C1orf65	1.1	0.9	1.0	0.9	0.0	-0.1	-0.4	-0.8	2	1
F1RMZ8	PIG Uncharacterized protein	ATP6V1B2	0.7	1.1	1.3	0.7	-0.8	-1.1	-0.7	-1.4	6	1
Q9TTA0	PIG Heat shock protein 47 (Fragment)	NA	-0.2	0.2	1.5	2.3	-0.1	1.2	1.9	0.3	6	4
Q9XSW7	PIG Cyclic nucleotide phosphodiesterase PDE3A	NA	2.7	0.2	0.7	0.2	0.0	-0.2	0.1	1.1	2	7
F1SH96	PIG Uncharacterized protein	NA	1.1	0.8	1.0	0.9	0.5	0.4	0.4	-0.4	3	1
I3L6X7	PIG Uncharacterized protein	SNF8	1.0	1.0	1.0	0.8	0.4	0.1	-0.1	-0.5	1	3
A7VK00	PIG Interferon-induced GTP-binding protein Mx2	MX2	1.3	0.6	0.9	1.0	3.5	1.1	0.6	1.3	10	1
P49720	HUMAN Proteasome subunit beta type-3	PSMB3	0.5	1.2	1.6	0.4	0.2	-0.6	1.3	-0.7	2	1
B6ICW2	PIG SLA-1 histocompatibility antigen, class I	SLA-1a	0.7	0.3	1.7	1.1	1.8	0.8	1.3	1.2	3	1
F1SD70	PIG Uncharacterized protein	LGMN	0.9	0.4	1.0	1.5	2.1	2.2	-0.2	-1.4	1	1
F1SGP8	PIG Uncharacterized protein	RCN1	0.3	0.2	1.0	2.3	1.6	1.5	1.4	1.0	12	1
Q8NBS9	HUMAN Thioredoxin domain-containing protein 5	TXNDC5	0.0	0.8	1.1	1.9	0.3	0.4	0.6	-1.2	6	1
I3LM34	PIG Uncharacterized protein	ADPRHL1	1.9	0.8	0.5	0.5	1.2	1.2	0.9	0.0	2	6
D3K5N7	PIG ELAV (Embryonic lethal, abnormal vision, Drosophila)-like 1	ELAVL1	1.3	0.9	0.7	0.9	-0.2	0.1	0.0	-0.3	4	4
F1SIL5	PIG Uncharacterized protein	VPS13A	1.4	1.2	0.6	0.6	0.9	0.7	0.0	0.2	2	1
A5D9J3	PIG Transporter 1, ATP-binding cassette, sub-family B (MDR/TAP)	TAP1	1.4	1.5	0.8	0.0	1.8	2.7	1.4	-0.2	5	2
F1S666	PIG Uncharacterized protein	NA	1.1	0.9	0.9	0.9	1.5	0.9	0.6	-1.0	12	6
F1S2E5	PIG 40S ribosomal protein S24	RPS24	1.8	0.8	0.4	0.8	0.3	0.2	-0.6	-0.6	4	2
F1RS36	PIG 78 kDa glucose-regulated protein	HSPA5	0.4	0.9	1.2	1.3	1.1	1.2	0.0	-0.8	28	22
P30043	HUMAN Flavin reductase (NADPH)	BLVRB	1.0	1.7	1.1	0.0	-0.1	0.3	-1.8	-2.8	2	1
F1RLT1	PIG Uncharacterized protein (Fragment)	NA	1.1	0.9	0.9	0.9	-0.1	-1.5	-2.2	-1.4	1	1
A6XAC5	PIG Protein phosphatase 2A regulatory subunit B B56 alpha isoform	PPP2R5A	1.6	0.8	0.8	0.6	1.1	1.1	1.1	1.2	1	2
F1SMN1	PIG Uncharacterized protein (Fragment)	CALU	0.8	1.3	0.6	1.0	1.7	0.4	-1.1	-1.7	11	2
G9F6X8	PIG Prolyl 4-hydroxylase beta polypeptide	NA	-0.1	0.4	1.4	2.1	0.5	-0.2	-1.3	-1.9	29	15
F1RW78	PIG Uncharacterized protein	SSR1	0.5	1.0	1.1	1.1	2.4	2.0	-0.1	-0.7	4	1
K7GM18	PIG Uncharacterized protein	MAFF	0.4	0.9	1.2	1.2	0.0	0.2	-0.6	-0.8	1	1
I3LH19	PIG Uncharacterized protein	MUL1	1.7	1.5	0.4	0.0	0.5	-0.7	-2.1	-1.0	1	1
B5APU7	PIG Actin related protein 2/3 complex subunit 2	NA	-0.2	0.6	1.5	1.8	-0.8	-0.1	-0.4	0.1	4	1
F1SIH5	PIG Uncharacterized protein	STOML2	1.6	1.4	0.3	0.4	1.6	1.1	-0.5	-1.7	7	5
Q9H299	HUMAN SH3 domain-binding glutamic acid-rich-like protein 3	SH3BGRL3	0.8	1.1	1.0	0.7	0.9	0.6	0.2	0.3	3	1
Q06AT6	PIG RHOG	NA	0.2	0.2	1.5	1.8	-0.6	-0.2	-0.3	-0.1	5	1
F1S5A8	PIG Uncharacterized protein	DHX15	1.3	0.8	0.9	0.6	-0.1	0.5	0.2	-0.2	3	1
K7GQL8	PIG Uncharacterized protein (Fragment)	CLTC	0.9	0.5	1.0	1.3	0.2	0.1	-0.4	0.1	33	25
O00299	HUMAN Chloride intracellular channel protein 1	CLIC1	-0.2	0.3	1.4	2.2	0.1	0.4	-0.7	0.4	2	2
I3L9V2	PIG Uncharacterized protein	VAT1	0.9	0.5	0.7	1.5	0.7	0.7	-0.3	-0.1	12	5
I3L8F7	PIG Uncharacterized protein	NES	0.3	0.6	1.4	1.3	-0.6	-1.5	-4.6	-3.3	5	1
P08132	PIG Annexin A4	ANXA4	0.4	0.7	1.1	1.5	0.0	-0.6	0.1	-1.1	13	3
F1S433	PIG Uncharacterized protein	LOC100525224	0.6	1.2	1.1	0.8	-0.9	-1.8	-1.2	-1.6	1	6

Q52NJ5	PIG ADP-ribosylation factor-like protein 1	ARL1	0.1	0.8	1.3	1.4	-0.2	0.4	-1.3	-0.4	2	2
F1SHR3	PIG Uncharacterized protein	ATP6V1E1	0.5	0.6	1.1	1.4	1.6	2.7	3.7	0.5	5	1
Q45FY6	PIG Hypoxanthine-guanine phosphoribosyltransferase	HPRT1	0.3	1.7	0.7	0.9	1.3	0.8	1.1	-0.6	3	3
I3L7C9	PIG Uncharacterized protein (Fragment)	BRCA1	1.5	1.1	0.7	0.3	-0.1	-0.4	-2.5	-3.8	1	1
F1RKQ4	PIG Bifunctional ATP-dependent dihydroxyacetone kinase/FAD-AMP lyase (cyclizing)	DAK	1.5	0.7	0.7	0.6	0.9	1.3	1.0	-1.3	8	2
A1XQV4	PIG Tropomyosin alpha-3 chain	TPM3	0.8	1.7	1.1	0.1	3.2	2.3	-1.1	-5.6	8	3
F1STS9	PIG Uncharacterized protein (Fragment)	NA	0.9	0.8	0.8	1.1	0.8	0.6	0.7	1.4	3	1
F1SPF6	PIG Uncharacterized protein	LOC100518399	0.8	1.0	0.8	1.0	-0.9	0.0	-1.2	-1.1	3	1
F1SBA5	PIG Proteasome subunit alpha type	PSMA8	0.3	0.7	1.5	1.1	0.1	0.0	0.7	-0.4	2	3
I3LDT1	PIG Uncharacterized protein	CD3EAP	0.6	0.8	1.1	1.1	1.3	1.4	1.3	1.2	1	1
O77735	PIG Secretory carrier-associated membrane protein 1	SCAMP1	0.7	0.8	1.4	0.7	1.5	1.0	0.5	0.6	1	1
F1S2B6	PIG Biglycan (Fragment)	BGN	-0.1	0.3	1.2	2.2	-0.4	0.4	0.4	0.9	4	2
D3DTX7	HUMAN Collagen, type I, alpha 1, isoform CRA	COL1A1	-0.2	1.2	0.8	1.9	1.8	2.8	0.8	1.4	2	2
Q9H5Z6	HUMAN Protein FAM124B	FAM124B	1.4	2.1	0.2	-0.2	-0.8	-1.9	-1.0	0.2	1	1
I3LG96	PIG Uncharacterized protein (Fragment)	NA	1.3	0.8	0.8	0.8	1.4	1.8	-1.3	-2.1	1	1
A7WLI1	PIG CD81	CD81	1.1	0.9	0.8	0.9	0.5	1.1	0.3	1.7	1	1
F1RIF8	PIG 6-phosphogluconate dehydrogenase, decarboxylating (Fragment)	PGD	0.0	1.0	1.2	1.4	-1.2	-0.6	-1.2	-0.5	7	5
F1RGQ6	PIG Uncharacterized protein (Fragment)	ANKFY1	0.8	0.8	1.1	0.9	0.1	0.4	1.2	2.1	1	2
I3L677	PIG Glucose-6-phosphate 1-dehydrogenase (Fragment)	G6PD	0.2	1.0	1.3	1.1	0.6	-0.4	-0.8	1.3	13	2
P68402	HUMAN Platelet-activating factor acetylhydrolase IB subunit beta	PAFAH1B2	0.7	1.1	1.0	0.7	0.9	1.5	0.0	-1.1	3	2
P10668	PIG Cofilin-1	CFL1	-0.2	0.3	1.5	2.0	0.3	0.2	-0.4	-0.7	8	9
F1S4Z2	PIG Uncharacterized protein	CHMP4B	0.5	1.2	1.2	0.7	1.4	2.3	0.2	-0.3	3	1
F1RMT1	PIG Uncharacterized protein	PVR	1.8	1.5	0.4	-0.2	2.4	0.7	0.1	-0.6	1	1
P63173	HUMAN 60S ribosomal protein L38	RPL38	0.0	0.3	1.2	2.0	-1.0	-0.8	-1.7	-0.8	5	2
F1SA28	PIG Uncharacterized protein	ECSIT	1.0	0.7	1.0	0.9	0.1	0.9	0.5	-0.1	1	1
F1S6G4	PIG Uncharacterized protein (Fragment)	TXNDC12	0.0	1.1	1.1	1.3	-0.7	0.0	0.5	0.9	3	2
F1SST9	PIG Uncharacterized protein	PIKFYVE	1.2	1.3	0.5	0.4	-2.2	-1.1	-2.7	0.2	1	1
A5A767	PIG Adaptor-related protein complex 3, beta-1 subunit	AP3B1	1.4	1.4	0.7	0.1	-0.4	0.5	-0.5	-1.0	1	1
P62244	HUMAN 40S ribosomal protein S15a	RPS15A	0.6	0.3	1.4	1.3	0.0	0.4	-0.1	0.2	4	4
F1SMC0	PIG Adenylyl cyclase-associated protein (Fragment)	CAP1	0.4	0.7	1.1	1.3	0.3	0.6	-0.9	-0.2	12	4
F1RRK9	PIG Uncharacterized protein	LOC100155084	0.9	0.6	0.9	1.1	0.5	1.0	0.8	1.4	6	2
F1SFJ8	PIG Uncharacterized protein	DNAJB11	1.4	0.9	0.7	0.6	0.4	0.2	-0.9	-1.1	4	1
P21333	HUMAN Filamin-A	FLNA	-0.1	0.1	1.2	2.3	0.0	0.3	1.4	1.3	61	40
Q9UP79	HUMAN A disintegrin and metalloproteinase with thrombospondin motifs 8	ADAMTS8	1.0	3.3	0.0	-0.8	-1.5	-1.6	-1.0	-0.7	1	1
F1RIW4	PIG Uncharacterized protein	DDX60	0.8	1.5	0.7	0.5	-0.4	-0.5	-0.3	0.9	1	1
Q29284	PIG Heat shock protein HSP 90-beta (Fragment)	NA	-0.5	0.6	2.0	1.4	-0.4	-0.1	0.6	0.6	2	2
Q9Y468	HUMAN Lethal(3)malignant brain tumor-like protein 1	L3MBTL1	4.7	1.3	-0.9	-1.6	0.0	0.3	0.2	1.1	1	1
F1SK12	PIG Uncharacterized protein	MAP1B	0.7	0.4	0.8	1.6	0.0	0.9	-0.1	0.4	21	2
Q96M60	HUMAN Protein FAM227B	FAM227B	-0.3	2.1	2.0	-0.3	-1.0	-0.3	-0.7	-1.0	1	1
I3L5C7	PIG Uncharacterized protein	NA	0.6	0.6	0.9	1.4	0.8	0.2	0.7	1.6	6	2

Q56P20	PIG ADP-ribosylation factor 4	ARF4	0.2	0.4	1.4	1.5	-0.4	0.3	0.0	0.9	6	5
O15145	HUMAN Actin-related protein 2/3 complex subunit 3	ARPC3	0.4	0.3	1.1	1.7	0.4	0.1	0.3	-0.5	6	2
F1SIP0	PIG Uncharacterized protein	SEPT2	1.1	0.8	0.9	0.7	1.2	1.3	0.2	-0.4	5	4
F1RS37	PIG Uncharacterized protein	POSTN	0.4	0.2	1.0	1.9	-0.3	0.3	0.9	1.5	19	2
F1S0A2	PIG Peptidyl-prolyl cis-trans isomerase	LOC100737887	0.4	0.6	1.1	1.3	0.0	0.1	-0.3	0.2	4	1
I3LP25	PIG Uncharacterized protein	NA	1.8	0.8	0.5	0.4	2.0	1.3	0.6	-0.8	1	1
K9J6K2	PIG Utrophin	UTRN	0.9	0.5	1.1	0.9	-0.4	-0.1	-0.2	0.8	2	2
F1SPG0	PIG Uncharacterized protein (Fragment)	RAB7A	0.7	0.7	1.1	0.9	0.8	1.5	0.1	0.0	7	3
I3LRP1	PIG Uncharacterized protein (Fragment)	NASP	1.0	0.7	1.1	0.7	0.5	0.1	-0.5	0.0	3	1
P01715	HUMAN Ig lambda chain V-IV region Bau	NA	1.1	0.6	1.4	0.4	-0.2	-1.1	0.8	-0.7	1	1
F1SLQ7	PIG Uncharacterized protein	MRRF	1.8	2.0	0.0	-0.3	1.7	3.7	3.1	1.2	1	1
F1RJX8	PIG Uncharacterized protein	COPA	0.6	0.3	1.3	1.2	0.8	1.7	0.7	0.8	17	4
F1STR1	PIG Uncharacterized protein	CTSC	0.0	0.2	1.5	1.8	0.4	0.7	0.7	-0.6	4	3
F1SMW9	PIG Uncharacterized protein	PSMD1	1.1	0.7	0.8	0.8	-0.7	-0.3	-1.2	0.3	6	7
Q07065	HUMAN Cytoskeleton-associated protein 4	CKAP4	0.7	0.4	1.0	1.4	1.0	0.9	-0.3	0.3	7	2
I3L742	PIG Uncharacterized protein	LOC100518219	1.4	1.2	0.8	0.1	1.2	1.1	-0.4	-1.0	3	2
F1RRF0	PIG Oxysterol-binding protein	OSBPL10	0.6	1.0	0.9	0.9	-1.2	0.3	-1.5	-0.5	1	1
F2Z5D4	PIG Uncharacterized protein (Fragment)	SYNCRIP	1.0	0.7	0.8	0.9	0.5	0.8	-0.8	-1.2	12	4
P08134	HUMAN Rho-related GTP-binding protein RhoC	RHOC	0.0	0.1	1.4	2.0	1.1	0.6	0.8	0.8	3	3
I3LDA8	PIG Uncharacterized protein (Fragment)	IQGAP1	0.5	0.5	1.1	1.3	-0.1	-0.6	-1.1	0.2	28	10
F1SD63	PIG Coiled-coil and C2 domain containing 1A	CC2D1A	0.5	1.2	1.6	0.2	3.4	11.4	14.9	2.0	3	1
D2JYW3	PIG F-actin capping protein beta subunit variant I	CAPZB	0.4	0.5	1.3	1.2	0.2	0.6	0.8	0.0	1	1
Q86VQ0	HUMAN Lebercilin	LCA5	-0.6	1.2	1.5	1.2	4.4	3.4	1.5	0.3	3	1
O60662	HUMAN Kelch-like protein 41	KLHL41	0.9	0.6	1.1	0.8	0.0	0.0	0.6	0.4	1	4
I3LPM1	PIG Uncharacterized protein	TMEM70	1.3	1.3	0.5	0.3	0.5	0.5	1.0	0.1	2	2
F1S0Y1	PIG Uncharacterized protein (Fragment)	HMCN2	1.0	0.7	0.7	1.0	-0.2	-0.4	0.0	0.5	2	2
F1SGD7	PIG Uncharacterized protein	NAP1L1	0.7	1.2	0.8	0.7	0.6	0.2	-2.6	-1.7	3	1
Q15814	HUMAN Tubulin-specific chaperone C	TBCC	1.6	1.6	0.5	-0.3	2.1	1.9	0.1	0.9	1	1
P14332	PIG 6-phosphogluconate dehydrogenase, decarboxylating (Fragment)	PGD	0.4	0.7	1.1	1.1	-0.3	-0.9	-0.3	-0.2	7	1
Q9NVJ2	HUMAN ADP-ribosylation factor-like protein 8B	ARL8B	1.5	-0.1	0.6	1.3	-0.4	-0.3	-0.5	-0.2	3	2
I3LK51	PIG Uncharacterized protein (Fragment)	NA	0.6	0.2	1.0	1.5	0.2	0.3	-0.7	-0.9	3	3
F1S1E7	PIG Uncharacterized protein	DPYS	0.1	0.6	1.6	1.1	3.6	0.3	1.7	2.9	1	1
F1RU31	PIG Uncharacterized protein	SART1	2.0	0.2	0.6	0.5	1.3	0.6	0.6	0.5	2	1
Q29305	PIG Disulfide isomerase related protein (ERP72) (Fragment)	NA	0.5	0.5	1.1	1.3	1.2	1.7	0.7	0.5	5	3
Q29384	PIG Proteasome subunit beta type-4 (Fragment)	PSMB4	1.3	0.5	0.8	0.8	-1.6	-1.6	0.4	0.2	2	1
F1S519	PIG Uncharacterized protein (Fragment)	KIF3B	0.9	0.6	0.9	1.0	-0.7	-1.2	-1.6	-1.1	1	2
Q68CN4	HUMAN Putative uncharacterized protein DKFZp686E23209	DKFZp686E23209	0.4	0.9	1.2	0.8	-0.3	-1.6	0.7	-0.6	1	1
P62913	HUMAN 60S ribosomal protein L11	RPL11	0.8	-0.2	0.6	2.0	-0.1	-1.2	-2.1	-0.9	1	1
P61923	HUMAN Coatomer subunit zeta-1	COPZ1	0.6	1.0	0.8	1.0	1.1	0.2	0.2	0.1	5	1
F1RHE6	PIG Uncharacterized protein	KCTD12	0.6	0.5	1.0	1.2	1.3	2.3	1.6	-0.9	10	3

F1SSF7	PIG Uncharacterized protein	AEBP1	0.1	0.4	0.7	2.0	0.5	1.5	0.9	1.4	1	1
F1SR70	PIG Uncharacterized protein	LOC100524650	0.2	0.6	1.5	1.0	-0.7	-0.4	1.0	-0.8	2	2
I3L6Q5	PIG Uncharacterized protein	HIST1H1B	-0.3	0.7	1.3	1.6	0.2	0.7	2.3	3.8	4	4
Q59IP1	PIG Procollagen alpha 3(V)	COL5A3	-0.1	0.7	1.0	1.7	-0.7	-0.7	-0.9	-0.4	3	2
K9IWI4	PIG Talin-1	TLN1	-0.2	-0.1	1.5	2.2	0.6	0.4	0.6	1.8	2	2
F1SNE9	PIG Coatomer subunit gamma	COPG2	-0.3	0.6	1.1	2.0	-0.6	0.2	-1.3	-1.8	1	1
A7KZR6	PIG Cytochrome P450 (Fragment)	CYP27A1	0.8	1.3	0.9	0.3	1.4	2.0	0.2	-0.3	1	1
I3LPP4	PIG Uncharacterized protein (Fragment)	CSE1L	0.8	0.5	0.8	1.2	-1.7	-2.3	-1.7	-1.2	3	1
K7GSG9	PIG Uncharacterized protein	CXCL17	0.1	0.9	1.4	0.7	0.3	-0.3	-0.4	-1.4	1	1
F1RLI6	PIG Solute carrier family 25, member 46	SLC25A46	1.8	0.8	0.6	0.0	0.2	-0.6	-1.4	-1.3	4	4
F1SNC2	PIG Guanine nucleotide-binding protein subunit gamma	GNG10	0.0	0.5	1.4	1.3	0.4	-0.2	0.3	0.7	1	1
Q764M5	PIG Signal transducer and activator of transcription 1	STAT1	1.1	0.3	0.8	1.0	1.3	0.6	-0.1	0.4	14	12
Q06AA3	PIG Regucalcin	RGN	2.7	0.9	0.0	-0.3	3.5	4.2	2.7	-1.1	1	1
F1SQ11	PIG Uncharacterized protein (Fragment)	EEA1	0.8	0.7	0.8	1.0	1.3	1.2	2.3	1.9	11	1
F1S2Q3	PIG Uncharacterized protein	TMED10	0.8	0.7	0.8	0.8	-0.7	-0.9	-0.6	-0.1	6	3
M3V7X9	PIG Lectin, galactoside-binding, soluble, 3 binding protein	LGALS3BP	1.1	0.1	0.8	1.2	1.1	1.4	1.1	1.0	6	2
P08758	HUMAN Annexin A5	ANXA5	-0.4	0.9	1.2	1.6	-0.7	0.1	0.4	0.7	3	3
F1RY58	PIG Uncharacterized protein	NDUFAF6	0.6	0.4	0.8	1.5	-0.1	-0.2	-0.4	-1.1	1	2
F1RLL9	PIG Uncharacterized protein (Fragment)	COL4A2	0.3	0.5	1.0	1.4	-0.7	-1.4	-1.9	-0.8	4	2
Q9NWQ4-4	HUMAN Isoform 4 of G patch domain-containing protein 2-like	GPATCH2L	2.1	1.1	0.1	-0.2	1.0	0.9	-0.2	-0.8	1	1
I3LI24	PIG Guanine nucleotide-binding protein subunit beta-2-like 1	GNB2L1	0.3	0.0	1.3	1.7	0.0	0.1	-0.1	0.7	8	7
P19620	PIG Annexin A2	ANXA2	-0.1	0.4	1.2	1.8	0.1	0.4	0.6	0.5	32	24
O00469	HUMAN Procollagen-lysine,2-oxoglutarate 5-dioxygenase 2	PLOD2	0.6	0.4	0.9	1.4	-1.1	-1.7	-0.6	-0.9	1	1
Q6NSI1	HUMAN Putative ankyrin repeat domain-containing protein 26-like protein	ANKRD26P1	-0.3	0.1	1.3	2.0	-0.2	-0.2	-0.5	-1.1	1	1
F1SDR7	PIG Uncharacterized protein	YWHAB	0.5	0.4	1.0	1.3	0.9	0.8	0.6	-0.6	8	7
F1RK00	PIG Uncharacterized protein	ZC3H13	1.3	1.2	0.4	0.2	-0.1	-0.2	0.0	0.9	1	1
B1PK15	PIG Beta-hexosaminidase	NA	0.5	0.6	0.8	1.3	0.6	1.1	0.6	-0.7	5	1
P55735	HUMAN Protein SEC13 homolog	SEC13	0.5	0.2	0.9	1.6	0.7	0.5	0.5	0.0	3	1
F1RWY9	PIG Uncharacterized protein	PSMG4	1.1	1.2	0.7	0.1	1.6	1.3	-0.3	-0.6	1	1
Q96QK1	HUMAN Vacuolar protein sorting-associated protein 35	VPS35	0.8	0.6	0.7	1.0	1.5	3.3	0.1	0.7	2	2
Q6P0N0	HUMAN Mis18-binding protein 1	MIS18BP1	-0.2	1.2	1.4	0.7	-2.5	-2.7	0.3	0.8	1	1
Q29099	PIG Polypyrimidine tract-binding protein 1	PTBP1	-0.2	0.5	1.2	1.6	-0.4	1.3	0.4	0.4	6	2
P55060	HUMAN Exportin-2	CSE1L	0.9	1.0	0.6	0.6	0.2	0.2	0.5	0.7	4	3
F1RGA9	PIG Coronin (Fragment)	CORO1C	0.5	0.2	1.0	1.4	-1.5	-1.0	-0.7	-0.1	7	3
F1SFS5	PIG Uncharacterized protein (Fragment)	TENC1	0.7	0.6	1.2	0.6	-0.6	0.1	-0.4	-0.1	1	1
F1SI65	PIG Uncharacterized protein	NA	0.1	0.6	0.9	1.5	-2.6	-2.2	-2.2	-1.0	2	1
F1SD97	PIG Uncharacterized protein	FARSA	1.8	0.4	0.3	0.6	2.6	1.2	0.8	0.7	3	2
F1SGC8	PIG Uncharacterized protein (Fragment)	ACTL6A	1.7	1.2	0.2	0.0	1.1	0.7	0.8	1.1	1	1
E7EI20	PIG Rho GDP dissociation inhibitor alpha	ARHGDIA	0.1	0.1	1.2	1.7	-0.1	0.0	-0.2	-0.9	11	7
F2Z5H8	PIG Uncharacterized protein (Fragment)	BTF3	0.7	0.6	0.9	0.8	1.1	1.2	1.7	0.5	6	1

F1S340	PIG Uncharacterized protein	SRI	0.5	1.2	0.8	0.6	1.2	0.2	0.6	0.2	4	1
F1RRD3	PIG Uncharacterized protein	NAA15	-0.5	1.3	1.5	0.7	-0.1	0.1	-0.6	-0.3	2	2
F1S754	PIG Signal recognition particle receptor subunit alpha	SRPR	1.6	0.4	0.7	0.3	0.0	-1.3	0.4	2.1	3	1
L8B0W9	PIG IgG heavy chain	IGHG	1.4	1.0	0.5	0.1	0.6	-0.2	2.2	-0.2	4	1
Q9UK96	HUMAN F-box only protein 10	FBXO10	1.3	1.1	0.6	0.0	0.3	-0.2	1.1	1.1	1	1
Q29021	PIG Apolipoprotein B (Fragment)	APOB	1.1	0.6	0.9	0.5	0.5	-0.3	1.1	0.4	12	3
F1RGP1	PIG Uncharacterized protein	MYBBP1A	0.8	0.5	0.7	1.1	0.6	0.7	0.8	1.2	8	2
Q14247	HUMAN Src substrate cortactin	CTTN	0.7	0.5	0.9	0.9	0.8	-0.9	-0.4	-0.7	3	1
Q92900	HUMAN Regulator of nonsense transcripts 1	UPF1	0.9	0.8	0.8	0.6	-0.9	-0.6	0.2	1.2	2	1
Q562Z4	HUMAN Actin-like protein (Fragment)	ACT	0.9	0.8	0.5	0.8	-0.7	-0.7	-0.7	-0.6	3	2
F1RLD2	PIG Uncharacterized protein	SEMA6A	0.6	0.5	0.8	1.2	-0.8	-0.8	-0.4	0.2	1	1
Q6PEW1	HUMAN Zinc finger CCHC domain-containing protein 12	ZCCHC12	2.7	2.0	-0.4	-1.2	3.9	5.5	-0.1	-1.2	1	1
I3LT08	PIG Uncharacterized protein	LOC100737048	0.7	0.2	1.0	1.1	1.7	1.1	-0.6	-0.4	4	3
P68104	HUMAN Elongation factor 1-alpha 1	EEF1A1	-1.1	-0.1	1.9	2.4	-1.4	-0.3	2.6	3.3	1	1
F1RR44	PIG Uncharacterized protein	PPP2R4	1.3	0.4	0.6	0.8	0.1	0.0	-0.1	0.5	3	2
P00761	PIG Trypsin	NA	1.1	1.3	0.6	0.0	0.6	2.3	2.4	3.3	6	7
O14979	HUMAN Heterogeneous nuclear ribonucleoprotein D-like	HNRNPDL	0.6	0.4	0.9	1.1	0.7	1.0	1.0	-1.1	2	2
Q5HYI7	HUMAN Metaxin-3	MTX3	0.3	0.9	1.4	0.3	0.5	0.6	1.1	0.5	1	1
F1RQJ4	PIG Uncharacterized protein	LOC100737246	1.1	0.8	0.7	0.5	-0.8	-0.7	-1.6	-0.6	6	1
I3LHI0	PIG Uncharacterized protein	KRT23	0.8	0.9	0.4	0.9	0.0	-1.1	3.0	7.7	1	1
P37176	PIG Endoglin	ENG	1.1	1.1	0.6	0.1	1.2	2.0	0.9	-0.8	3	2
P50552	HUMAN Vasodilator-stimulated phosphoprotein	VASP	0.8	0.9	0.7	0.7	0.6	0.4	1.6	0.7	3	1
F1SFF6	PIG Uncharacterized protein	TMX1	1.1	0.6	0.7	0.6	0.4	0.5	-0.5	1.2	4	4
P02751	HUMAN Fibronectin	FN1	0.9	0.4	0.8	1.0	2.1	1.6	-0.2	0.5	5	1
G9FSR3	PIG Non-muscle myosin heavy chain II A (Fragment)	NA	0.2	0.3	1.0	1.5	0.4	0.2	-0.5	0.6	27	18
F1S4D7	PIG Uncharacterized protein	GBP1	0.8	0.9	0.7	0.5	-0.1	0.6	-0.3	-0.4	4	3
F1RFY1	PIG Profilin	PFN1	-0.2	0.2	1.5	1.5	-0.8	-0.1	0.3	0.4	9	8
F1S935	PIG Uncharacterized protein	RPL18A	0.2	0.8	0.9	1.1	-0.9	-0.2	0.1	-0.1	2	2
F1S775	PIG Uncharacterized protein	STT3A	0.3	0.5	1.1	1.2	-0.6	-0.1	-0.1	0.4	7	2
P83916	HUMAN Chromobox protein homolog 1	CBX1	0.7	1.4	1.0	-0.1	1.2	0.0	-1.0	-1.9	3	1
P07477	HUMAN Trypsin-1	PRSS1	0.8	0.9	0.7	0.6	-0.7	0.4	1.4	2.0	1	1
F1S0P3	PIG C-type natriuretic peptide	CNP	0.6	0.7	0.8	0.9	0.6	0.9	-0.2	-0.4	2	1
F1SUW3	PIG Uncharacterized protein	EFHD2	0.0	0.4	1.2	1.4	0.5	1.3	0.3	0.3	10	2
D2WKD8	PIG Sodium/potassium-transporting ATPase subunit alpha-2	ATP1A2	1.0	0.9	0.7	0.4	-0.5	0.8	0.1	-0.1	1	1
F1RQ06	PIG Peptidyl-prolyl cis-trans isomerase	FKBP2	0.4	0.8	1.1	0.7	-0.1	0.1	0.1	-0.4	3	2
O95831	HUMAN Apoptosis-inducing factor 1, mitochondrial	AIFM1	2.0	1.2	0.2	-0.4	-0.9	-0.1	-0.5	0.7	1	1
F1S365	PIG Uroporphyrinogen decarboxylase	UROD	0.0	0.5	1.2	1.3	-0.2	0.1	-0.1	-0.1	1	1
F1S0A3	PIG Uncharacterized protein (Fragment)	SNX1	1.6	0.7	0.2	0.4	0.4	-0.3	-0.6	-0.5	3	1
O75330	HUMAN Hyaluronan mediated motility receptor	HMMR	1.9	1.3	-0.1	-0.2	3.0	1.3	-0.1	0.0	2	1
I3LDY4	PIG Uncharacterized protein (Fragment)	VARS	1.4	0.5	0.4	0.7	0.5	0.7	0.1	-0.7	2	1

I3LE55	PIG Uncharacterized protein (Fragment)	NA	1.0	0.9	0.9	0.1	-0.3	-0.9	1.0	1.1	1	2
I3LGP6	PIG Uncharacterized protein (Fragment)	TSTA3	1.5	0.8	0.3	0.4	0.9	0.6	-0.2	0.4	1	2
I3LVP6	PIG Uncharacterized protein (Fragment)	ANKLE2	1.1	1.0	0.6	0.3	-0.3	-1.0	-0.5	-0.2	1	1
Q9GL01	PIG Dolichyl-diphosphooligosaccharide--protein glycosyltransferase subunit 2	RPN2	1.0	0.3	0.7	1.0	0.7	0.4	0.2	0.3	11	8
F1SGS6	PIG Uncharacterized protein	CAPRIN1	0.3	0.4	1.1	1.2	0.7	-0.1	-1.0	-0.8	4	1
Q8TF72	HUMAN Protein Shroom3	SHROOM3	-0.5	1.4	1.1	0.9	-1.5	-0.7	-0.2	-0.4	1	1
P52552	PIG Peroxiredoxin-2 (Fragment)	PRDX2	-0.8	1.3	1.8	0.6	0.4	-0.6	-0.8	-3.8	8	7
F1RW32	PIG Uncharacterized protein	SPARCL1	0.6	0.4	0.7	1.2	1.1	1.5	0.7	0.0	1	1
Q15746	HUMAN Myosin light chain kinase, smooth muscle	MYLK	0.8	0.5	0.4	1.2	1.1	0.4	-0.2	-0.6	2	1
F1SJJ5	PIG 60S ribosomal protein L4	RPL4	-0.1	0.2	1.2	1.6	-0.5	-0.4	-0.5	1.5	11	7
Q4GWZ2	PIG 40S ribosomal protein SA	RPSA	0.3	0.2	1.2	1.3	0.1	0.7	-0.1	-1.0	8	5
F1RZ70	PIG Uncharacterized protein	SEC14L1	0.0	1.2	1.4	0.3	0.7	-0.9	0.3	0.8	1	1
Q8N2S1	HUMAN Latent-transforming growth factor beta-binding protein 4	LTBP4	1.5	1.9	-0.1	-0.4	-0.3	0.5	0.5	1.5	1	1
F1SII0	PIG Uncharacterized protein	DNAJB5	-0.1	0.3	1.6	1.2	-1.6	-1.1	-0.9	0.2	1	1
F1REW9	PIG Uncharacterized protein	DENR	0.9	0.4	0.6	1.0	2.9	2.7	1.2	2.2	3	1
I3LER3	PIG Uncharacterized protein	LOC100620173	0.9	0.3	0.6	1.2	0.7	1.1	0.5	-0.1	1	1
L7PBE6	PIG T-complex protein 1 subunit epsilon	CCT5	-0.2	0.2	1.4	1.4	0.1	0.5	0.6	1.3	4	2
A6M929	PIG Eukaryotic translation initiation factor 4A isoform 2	EIF4A2	0.2	0.3	1.1	1.3	-0.1	0.2	-0.4	-0.3	13	11
Q06AU6	PIG Ras-related protein Rab-5A	RAB5A	1.2	0.5	0.6	0.6	-0.1	0.9	0.1	0.5	5	3
F1RUN2	PIG Serum albumin	ALB	1.3	0.6	0.5	0.5	-0.4	-1.3	0.3	-1.6	2	1
Q05193	HUMAN Dynamin-1	DNM1	1.1	1.0	0.5	0.3	0.0	0.7	-0.4	-0.5	1	1
P50570	HUMAN Dynamin-2	DNM2	0.3	0.6	0.8	1.1	0.0	0.9	0.7	0.4	1	1
I3LHM3	PIG Uncharacterized protein (Fragment)	TOP1	0.1	0.6	1.0	1.3	1.1	0.2	-0.4	0.5	3	1
F1RYI8	PIG Uncharacterized protein	COL3A1	0.0	0.0	1.0	1.8	0.7	0.8	0.7	1.6	7	2
F1SMM3	PIG Uncharacterized protein	RANBP6	3.0	0.2	0.3	-0.7	0.8	0.4	0.6	-0.6	1	2
P30041	HUMAN Peroxiredoxin-6	PRDX6	0.9	1.6	0.5	-0.1	1.5	1.3	0.4	-3.3	1	1
F1SPF9	PIG Uncharacterized protein (Fragment)	COPG1	0.6	0.5	0.7	1.0	-0.2	0.6	0.7	-0.1	6	3
F1S4R5	PIG Uncharacterized protein (Fragment)	SRSF7	1.2	0.4	0.6	0.6	0.1	0.4	-0.2	-0.1	5	5
I3LN21	PIG Uncharacterized protein (Fragment)	SEPHS1	1.9	1.0	-0.1	0.0	1.5	1.5	0.6	0.2	2	1
P16469	PIG Arachidonate 15-lipoxygenase	ALOX15	1.3	1.1	0.6	-0.2	0.7	1.0	-0.4	-0.6	4	1
P62312	HUMAN U6 snRNA-associated Sm-like protein LSm6	LSM6	0.2	0.7	1.0	0.9	0.1	0.3	0.6	0.2	3	2
Q5PXT2	PIG LIM and cysteine-rich domains protein 1	LMCD1	1.1	0.5	0.4	0.7	1.6	1.2	0.4	-0.2	4	3
M3TYC8	PIG DEAD (Asp-Glu-Ala-Asp) box helicase 1	DDX1	0.9	-0.1	1.1	1.0	0.2	0.1	0.1	-0.7	2	2
Q5K4G2	PIG Lysosome-associated membrane glycoprotein 1	LAMP-1	0.2	0.7	1.0	1.0	0.9	1.0	-0.1	-0.2	3	2
F1SIT7	PIG Uncharacterized protein	LOC100523874	-0.2	0.1	1.5	1.4	0.9	-0.5	-2.9	-5.3	1	1
F1SBS1	PIG Uncharacterized protein (Fragment)	CSDE1	1.4	0.7	0.5	0.1	0.7	1.1	-0.6	-1.5	4	1
A5GFX7	PIG Cathepsin Z	CTSZ	0.2	-0.4	0.9	2.1	0.3	0.0	-0.2	0.6	4	1
F1S3M9	PIG Uncharacterized protein	EPB41L2	0.7	0.6	0.6	0.8	0.7	0.7	0.2	-1.4	18	11
F2Z5Q2	PIG Uncharacterized protein (Fragment)	RPL24	0.4	0.0	0.7	1.7	-0.4	0.6	0.2	1.2	6	4
M3UZ93	PIG Synaptogyrin	SYNGR2	0.4	0.7	0.9	0.8	-0.2	0.3	1.3	0.8	2	1

F1RHM8	PIG Uncharacterized protein	RANBP1	0.0	0.5	1.2	1.1	2.2	1.2	1.3	-1.1	6	4
Q14905	HUMAN Myosin heavy chain beta-subunit (Fragment)	MYH7	1.5	0.5	0.1	0.7	-1.7	0.5	-2.5	-2.5	1	1
I3LVI8	PIG 60S ribosomal protein L7a	RPL7A	-0.1	0.6	0.9	1.4	-0.8	-1.0	0.0	0.6	3	2
I3LMH4	PIG Uncharacterized protein	EIF4G1	0.3	0.2	1.1	1.3	0.7	0.4	0.1	0.5	16	9
P06702	HUMAN Protein S100-A9	S100A9	0.5	1.2	0.8	0.3	0.7	-0.4	0.5	5.5	2	1
A1XQR9	PIG Small nuclear ribonucleoprotein E	SNRPE	1.2	0.4	0.6	0.6	0.4	-0.1	-1.5	-2.3	2	2
I3L7S4	PIG Uncharacterized protein	CALD1	-0.3	-0.1	0.9	2.3	2.2	2.1	1.9	1.0	28	8
P54289	HUMAN Voltage-dependent calcium channel subunit alpha-2/delta-1	CACNA2D1	0.3	1.2	1.1	0.2	0.2	0.8	-0.3	-1.6	1	1
F1SSF5	PIG Uncharacterized protein	BLVRA	0.4	1.0	1.1	0.3	0.6	0.5	-0.4	0.3	2	1
A2J1M5	HUMAN Rheumatoid factor RF-IP4 (Fragment)	NA	0.7	0.6	0.8	0.7	0.4	-0.9	1.2	1.3	2	1
F1SGS9	PIG Catalase	CAT	0.6	1.1	0.9	0.2	-0.3	-0.4	-0.8	-0.7	3	3
B5APV1	PIG Actin-related protein 2/3 complex subunit 5	NA	0.6	0.6	0.9	0.6	0.4	0.9	-0.5	0.3	2	3
L8AXK8	PIG IgG heavy chain	IGHG	0.7	1.4	0.6	0.1	-0.2	-0.2	1.2	0.3	1	1
M3VJZ7	PIG LIM and SH3 protein 1	LASP1	0.2	0.3	1.0	1.3	1.1	1.1	0.3	-0.6	10	7
F2Z5R5	PIG Uncharacterized protein	PDCD10	1.0	0.2	0.8	0.8	3.8	1.2	0.1	0.8	1	1
Q6IAN0	HUMAN Dehydrogenase/reductase SDR family member 7B	DHRS7B	1.0	1.4	0.4	-0.1	-0.9	0.4	0.0	0.6	1	2
F1S956	PIG Uncharacterized protein (Fragment)	PGLS	0.3	0.9	0.8	0.7	0.9	0.2	0.0	-0.9	4	1
I3LBZ1	PIG Uncharacterized protein (Fragment)	COL18A1	0.4	0.4	0.7	1.3	0.0	-0.3	-1.0	-2.0	6	6
F1SEM9	PIG Uncharacterized protein	SOGA1	1.0	1.4	0.4	0.0	-0.1	-0.5	-0.4	0.0	1	1
F1RX17	PIG Ubiquitin carboxyl-terminal hydrolase (Fragment)	LOC100517440	2.9	0.3	-0.3	-0.1	0.4	0.5	0.0	1.2	2	3
F1RYI3	PIG Uncharacterized protein	CAND1	0.8	0.5	0.7	0.8	0.4	1.1	-0.2	-0.1	11	5
A5A780	PIG Uncharacterized protein	VPS33A	0.6	0.7	0.8	0.6	0.8	1.3	2.0	1.0	2	1
Q7Z403	HUMAN Transmembrane channel-like protein 6	TMC6	1.5	0.5	0.4	0.4	-0.9	-0.5	-0.9	-0.2	2	1
F1SJR7	PIG Uncharacterized protein	TTLL12	0.7	0.4	0.8	0.8	0.7	-0.1	0.3	0.3	8	1
F1RQZ9	PIG Uncharacterized protein	EFTUD2	0.8	0.3	0.8	0.8	0.5	0.3	0.3	0.8	6	3
K7GL95	PIG Uncharacterized protein	MAGOHB	0.6	0.6	0.7	0.8	0.3	1.8	-0.6	-1.0	2	2
I3LLY9	PIG Uncharacterized protein (Fragment)	NA	1.2	0.8	0.4	0.4	-0.3	-1.3	-0.9	0.4	3	2
F1RWH4	PIG Uncharacterized protein (Fragment)	COPZ2	0.5	0.8	0.7	0.8	0.6	0.6	0.0	-0.3	2	1
P09493-2	HUMAN Isoform 2 of Tropomyosin alpha-1 chain	TPM1	0.4	0.6	0.5	1.3	1.1	1.0	0.3	-0.8	3	1
K7GPQ7	PIG Uncharacterized protein (Fragment)	AGT	1.2	0.8	0.4	0.3	0.7	-0.3	-0.8	-0.6	5	1
P29269	PIG Myosin regulatory light polypeptide 9	MYL9	0.1	0.9	0.6	1.0	1.3	0.6	-1.1	-2.0	10	8
B1PK10	PIG Neutrophil cytosolic factor 2	NA	0.7	0.3	1.0	0.8	0.5	0.4	1.1	-0.1	1	1
P53027	PIG 60S ribosomal protein L10a (Fragment)	RPL10A	-0.3	-0.3	1.3	1.9	-0.9	-0.5	0.3	0.2	7	5
Q8MIZ3	PIG m7GpppX diphosphatase	DCPS	0.6	0.8	0.7	0.6	2.7	0.7	1.3	-1.9	5	1
Q6N089	HUMAN Putative uncharacterized protein DKFZp686P15220	DKFZp686P15220	0.5	0.2	1.1	0.9	-0.8	-0.6	1.3	0.4	1	1
Q8N503	HUMAN LOC100049716 protein (Fragment)	LOC100049716	0.4	1.3	0.3	0.6	-1.5	-0.5	1.3	0.1	2	1
F1S021	PIG Uncharacterized protein (Fragment)	COL5A1	-0.3	0.6	1.0	1.4	-0.7	0.1	0.0	0.8	5	2
K9IWF9	PIG Ras GTPase-activating-like protein IQGAP1	IQGAP1	0.6	0.1	0.9	1.1	1.6	0.4	-0.5	0.6	4	2
K9J6J4	PIG Caspase-7 isoform alpha	CASP7	1.1	0.3	0.5	0.8	2.1	1.3	1.4	1.1	3	1
Q29036	PIG Dolichyl-diphosphooligosaccharide--protein glycosyltransferase subunit DAD1	DAD1	0.7	0.5	0.6	0.9	1.7	2.3	1.7	1.8	1	2

F1S9D1	PIG Uncharacterized protein (Fragment)	ACIN1	1.2	1.2	0.5	-0.2	-1.4	-1.2	-1.0	-0.5	1	2
C4MRC5	PIG ADP-ribosylation factor 2	ARF3	0.1	0.4	0.9	1.2	-0.4	0.8	1.0	0.2	7	4
F1S3P8	PIG Uncharacterized protein	STX7	1.2	0.6	0.3	0.6	1.3	1.4	0.5	-0.3	1	2
F1SRV9	PIG Uncharacterized protein	ZYX	0.5	0.3	0.6	1.2	1.7	1.1	0.2	1.0	6	5
F1RNP2	PIG Uncharacterized protein	AZGP1	0.9	0.6	0.8	0.4	2.2	2.3	2.8	1.5	10	3
P81605	HUMAN Dermcidin	DCD	0.4	0.6	0.8	0.9	-0.5	-0.5	-0.2	4.4	1	1
A0FGR8	HUMAN Extended synaptotagmin-2	ESYT2	0.6	0.9	0.6	0.6	0.9	0.7	-0.1	0.1	1	1
F1SAN6	PIG Uncharacterized protein	ILVBL	0.1	0.6	1.0	0.8	-0.6	-0.3	1.0	2.9	1	3
Q9NSG2	HUMAN Uncharacterized protein C1orf112	C1orf112	1.1	0.5	0.8	0.3	0.2	-0.2	-0.7	-2.2	1	1
Q2Q1W2	HUMAN E3 ubiquitin-protein ligase TRIM71	TRIM71	1.2	0.6	0.3	0.6	0.9	1.8	2.0	1.9	1	1
F1S9Y5	PIG Uncharacterized protein (Fragment)	PPP2R5C	0.5	0.9	0.7	0.7	-0.6	1.3	1.4	0.1	1	1
F2Z5C1	PIG Annexin	ANXA5	0.0	0.5	0.8	1.3	0.0	0.9	-0.1	-0.7	20	16
I3LIW3	PIG Uncharacterized protein	EIF2S2	0.2	0.3	0.9	1.2	0.0	-0.2	-0.5	-0.3	2	2
Q6ZP98	HUMAN Putative uncharacterized protein C16orf47	C16orf47	0.7	0.6	0.8	0.5	0.0	0.6	-0.1	0.0	1	1
F1SM79	PIG Uncharacterized protein	PKDREJ	0.8	0.7	0.3	0.8	-0.5	-0.1	0.4	0.0	1	1
F1SJ93	PIG Uncharacterized protein	RCN2	1.1	0.7	0.6	0.2	-0.1	-1.4	-5.1	-2.7	5	1
B6CVD6	PIG Thioredoxin domain-containing 4	TXNDC4	0.6	1.0	0.5	0.6	0.2	0.4	1.0	-0.2	1	1
F1SIY3	PIG Uncharacterized protein	NA	1.2	0.8	0.4	0.3	0.0	0.7	0.0	1.3	2	1
F1SRE0	PIG Uncharacterized protein	XRCC6	-0.3	-0.6	1.0	2.5	1.3	1.3	0.7	-0.9	1	2
F1SJY6	PIG Uncharacterized protein	CTNND1	0.6	1.1	0.6	0.3	0.5	0.3	-0.1	-0.7	5	2
F1RZT0	PIG Uncharacterized protein	TAPBP	0.9	0.7	0.6	0.5	1.4	1.2	-0.1	-0.1	4	3
B8XSJ0	PIG Acylglycerol kinase	AGK	1.3	1.3	-0.1	0.0	0.1	0.3	-0.5	-0.4	2	3
F1SK15	PIG Uncharacterized protein	LOC100518224	1.3	0.6	0.9	-0.2	-0.9	-1.6	-0.2	-0.3	2	6
I3L6Y6	PIG Uncharacterized protein (Fragment)	AP2B1	0.7	0.1	0.8	1.0	0.8	1.3	-0.2	-0.5	13	3
F1SV93	PIG Importin subunit alpha (Fragment)	KPNA6	1.2	0.7	0.4	0.3	1.9	2.1	1.2	0.3	1	1
K7GLR2	PIG Uncharacterized protein (Fragment)	FAF1	0.2	0.8	2.0	-0.4	-0.9	-1.0	-0.3	1.5	1	1
F1RQS5	PIG Uncharacterized protein	CDC5L	0.7	0.7	0.4	0.7	-0.6	0.6	0.2	1.5	2	1
F1RX66	PIG Uncharacterized protein (Fragment)	GSR	1.1	0.8	0.4	0.3	0.1	-0.1	1.0	-0.5	3	2
Q9C0F0	HUMAN Putative Polycomb group protein ASXL3	ASXL3	0.9	1.1	0.3	0.3	-0.3	-0.8	-1.2	-1.6	1	1
P30443	HUMAN HLA class I histocompatibility antigen, A-1 alpha chain	HLA-A	0.9	0.9	0.5	0.4	0.9	1.1	-0.5	-1.1	1	1
Q29381	PIG Dolichyl-diphosphooligosaccharide--protein glycosyltransferase 48 kDa subunit	DDOST	0.4	0.3	0.8	1.0	1.4	0.8	0.0	0.3	6	3
F1S3E0	PIG Uncharacterized protein (Fragment)	LOC100511312	0.4	0.5	0.7	1.0	0.4	0.1	-0.2	0.5	4	2
F1RQB6	PIG Uncharacterized protein (Fragment)	G3BP1	0.4	0.5	0.9	0.8	0.7	1.6	1.0	0.1	6	4
F1SPH1	PIG Uncharacterized protein	LOC100737962	0.4	-0.1	1.0	1.3	0.3	0.3	-0.3	0.9	9	6
P29536	HUMAN Leiomodin-1	LMOD1	1.4	1.6	-0.3	-0.1	-1.6	-1.7	-1.4	-1.6	1	1
P23220	PIG Plasma membrane calcium-transporting ATPase 1	ATP2B1	0.7	0.1	0.6	1.2	-0.2	-0.2	-0.5	-0.5	5	3
F1S9H9	PIG Low molecular weight phosphotyrosine protein phosphatase (Fragment)	ACP1	1.0	0.9	0.4	0.3	-0.1	0.3	-0.5	0.0	3	1
I3L918	PIG Uncharacterized protein	NA	1.1	1.0	0.3	0.2	-0.5	-1.0	0.9	2.5	3	2
Q6LEP1	PIG Ribosomal protein S4 (Fragment)	Rps4	0.2	-0.5	1.1	1.7	0.6	0.9	0.7	0.9	15	12
F1S7P0	PIG Uncharacterized protein	ANKRD24	1.1	1.0	0.3	0.2	1.1	0.2	0.8	-0.6	2	2

F1SAI8	PIG Uncharacterized protein	HYOU1	0.2	0.5	0.9	0.9	1.3	1.3	0.2	-0.1	15	3
Q86SQ0	HUMAN Pleckstrin homology-like domain family B member 2	PHLDB2	0.1	1.5	1.2	-0.3	0.5	0.6	0.2	-0.5	1	1
L8B180	PIG IgG heavy chain	IGHG	0.7	0.8	0.5	0.5	-1.2	-0.9	0.9	0.8	2	1
F1RYT3	PIG Uncharacterized protein	SCARB2	0.0	0.3	0.9	1.4	2.0	2.9	4.1	0.8	6	2
I3L8M1	PIG Uncharacterized protein	FBXO18	3.4	-0.3	-0.4	-0.2	2.9	0.7	1.4	1.4	1	2
F1RI41	PIG Eukaryotic translation initiation factor 3 subunit K	EIF3K	1.0	0.5	0.5	0.6	0.6	-0.1	-0.7	-0.3	2	1
K7GRK7	PIG Uncharacterized protein (Fragment)	NA	1.4	0.6	0.3	0.2	1.5	1.6	-1.0	1.1	2	2
Q6NSJ0	HUMAN Uncharacterized family 31 glucosidase KIAA1161	KIAA1161	0.4	0.5	0.7	1.0	2.7	0.6	0.2	1.1	1	1
I3LG15	PIG Uncharacterized protein (Fragment)	NA	0.3	0.7	1.1	0.4	0.5	1.0	0.6	2.8	2	3
I3L8I5	PIG Uncharacterized protein (Fragment)	EIF6	0.4	0.6	0.7	0.7	-1.7	-1.3	-0.5	0.7	3	1
Q9Y490	HUMAN Talin-1	TLN1	0.4	0.4	1.0	0.8	0.3	0.3	1.0	0.7	1	1
P62279	PIG 40S ribosomal protein S13 (Fragment)	RPS13	0.0	-0.1	1.1	1.4	0.1	0.4	0.2	-0.2	6	5
F1S458	PIG Lysine--tRNA ligase	KARS	0.8	0.3	0.6	0.8	1.4	0.8	0.7	-0.1	11	3
F1SH38	PIG Uncharacterized protein	PTPRB	2.6	0.4	-0.2	-0.3	-0.8	-0.4	0.8	-0.3	2	2
P07437	HUMAN Tubulin beta chain	TUBB	0.1	0.0	0.9	1.6	1.0	0.4	-1.2	-1.3	4	4
F1STE2	PIG Uncharacterized protein (Fragment)	TSGA10	0.5	0.7	0.4	0.9	-1.8	-1.6	-0.9	0.4	1	1
I3L8S3	PIG Uncharacterized protein (Fragment)	CMBL	0.7	0.7	0.7	0.4	0.9	1.2	0.6	0.7	3	2
F1RHU6	PIG Uncharacterized protein	PRMT1	0.9	0.2	0.7	0.7	0.3	-1.1	-1.1	-1.6	5	4
F1STE8	PIG Uncharacterized protein (Fragment)	EIF5B	0.7	0.3	0.8	0.7	-1.2	-0.8	-0.6	2.2	5	3
I3LIX1	PIG 60S ribosomal protein L27	RPL27	-0.4	0.1	1.2	1.6	-1.7	-0.7	0.0	0.7	5	5
Q9NZZ3	HUMAN Charged multivesicular body protein 5	CHMP5	1.6	0.3	0.4	0.2	2.3	1.4	1.0	-0.5	1	1
Q2MJV8	PIG Paralemmin-1	PALM	2.7	0.4	-0.2	-0.3	1.1	0.1	0.5	1.6	1	1
I3LF38	PIG Uncharacterized protein (Fragment)	SEC11A	0.4	0.5	0.8	0.8	-0.2	-0.1	0.1	-0.4	2	1
Q06AS6	PIG GBI2	GBI1	0.5	0.7	0.7	0.6	0.8	1.0	0.7	0.4	5	4
Q767L6	PIG Flotillin-1	FLOT1	0.8	0.6	0.8	0.2	0.5	0.2	-0.2	-1.2	10	5
Q5K4Q3	PIG Workshop cluster 3 antigen	swc3	0.9	1.1	0.7	-0.2	1.7	1.7	0.4	-0.3	1	2
Q08092	PIG Calponin-1	CNN1	0.6	0.1	0.4	1.3	0.2	1.5	3.8	1.4	13	9
I3LJS5	PIG Uncharacterized protein	DCTN4	1.4	0.6	0.2	0.4	0.7	1.6	-0.9	0.9	4	3
F1SSA6	PIG Uncharacterized protein (Fragment)	LOC396903	0.2	0.3	0.6	1.4	0.2	0.7	0.1	0.4	71	36
H7BZ55	HUMAN Putative ciliary rootlet coiled-coil protein-like 3 protein	NA	0.6	0.8	0.4	0.6	0.9	0.9	0.5	0.2	1	1
Q95LE2	PIG Laminin beta-1 chain (Fragment)	NA	0.4	0.8	0.8	0.4	0.5	0.3	0.3	0.4	3	4
P39019	HUMAN 40S ribosomal protein S19	RPS19	0.1	0.6	1.0	0.8	0.7	0.8	-0.1	0.0	1	1
H0Y500	HUMAN Bromodomain and WD repeat-containing protein 1 (Fragment)	BRWD1	1.0	1.1	0.7	-0.3	1.4	3.9	2.7	-0.8	1	1
F1RNK3	PIG Uncharacterized protein	LOC100519316	0.7	0.7	0.5	0.6	0.0	0.9	-0.3	0.4	3	3
F1S596	PIG Uncharacterized protein	PRKCSH	-0.1	0.7	0.8	1.0	3.0	1.2	0.1	-0.2	10	2
F1RY22	PIG Uncharacterized protein	MDN1	0.9	0.5	0.6	0.4	-0.9	-2.2	0.8	0.1	2	1
F2Z560	PIG Uncharacterized protein	RAB5C	0.7	0.7	0.5	0.5	0.5	2.3	-0.8	-0.8	3	2
P29412	PIG Elongation factor 1-beta	EEF1B	-0.7	0.4	1.2	1.5	1.4	1.0	-0.6	-1.6	4	2
Q9Y5U9	HUMAN Immediate early response 3-interacting protein 1	IER3IP1	0.7	0.6	0.6	0.6	0.1	0.0	-0.2	0.7	1	1
I3LVF5	PIG Uncharacterized protein	PA2G4	-0.2	-0.1	1.0	1.7	1.4	0.8	0.6	-0.1	2	2

P61353	HUMAN 60S ribosomal protein L27	RPL27	0.6	0.4	0.7	0.8	-0.1	0.2	1.0	0.0	1	1
F1RWH6	PIG Uncharacterized protein	CDK5RAP3	1.2	1.2	0.0	0.0	0.6	0.2	-0.7	0.7	1	1
Q7Z7M1	HUMAN Probable G-protein coupled receptor 144	GPR144	0.6	0.8	0.6	0.3	-0.1	0.8	-0.2	1.0	1	1
K7GRY0	PIG Uncharacterized protein	UBA1	0.3	0.5	0.8	0.8	0.1	0.3	0.3	0.4	23	19
I3LUM2	PIG Uncharacterized protein	NA	-1.4	1.0	0.7	2.2	1.8	2.3	1.9	1.7	2	4
F1SQ09	PIG Uncharacterized protein	LUM	0.1	0.6	0.4	1.3	1.3	0.7	0.2	-0.9	12	10
F1RXA1	PIG Uncharacterized protein	PAG1	-0.5	0.6	0.9	1.4	0.7	0.0	0.4	0.2	1	1
I3LS04	PIG Uncharacterized protein (Fragment)	NA	-0.4	-0.3	0.9	2.2	-0.3	0.0	0.7	0.0	18	15
P62899	HUMAN 60S ribosomal protein L31	RPL31	0.2	0.4	0.6	1.2	-0.4	0.8	-0.4	1.2	1	2
F1SH66	PIG Uncharacterized protein	LOC100625352	0.1	-0.1	0.7	1.7	-0.1	-0.1	1.0	1.0	1	1
Q32YV9	PIG Proteasome 26S subunit non-ATPase 4	PSMD4	0.5	0.9	0.5	0.5	-0.7	1.3	-1.4	-0.7	6	1
P62424	HUMAN 60S ribosomal protein L7a	RPL7A	-0.4	-0.2	1.3	1.8	-1.1	-0.8	-0.1	0.6	5	4
I3LPS9	PIG Uncharacterized protein (Fragment)	MLF1	0.9	0.9	0.4	0.2	-0.3	-0.7	-0.4	0.1	1	5
Q9BT78	HUMAN COP9 signalosome complex subunit 4	COPS4	1.2	0.6	0.5	0.1	0.9	-0.4	0.1	-1.3	4	3
Q9P1Z9	HUMAN Coiled-coil domain-containing protein 180	CCDC180	1.5	0.2	0.6	0.1	2.5	0.9	-0.4	-0.7	1	1
O60229	HUMAN Kalirin	KALRN	0.4	0.7	1.0	0.3	1.0	1.5	-0.5	-1.7	3	1
P51149	HUMAN Ras-related protein Rab-7a	RAB7A	-0.1	0.4	0.9	1.1	-1.9	-1.2	0.1	-0.3	3	3
I3LPD5	PIG Uncharacterized protein	NA	0.6	0.1	0.7	0.9	0.4	0.9	0.6	0.0	2	1
I3LES1	PIG Uncharacterized protein	NA	0.7	0.3	0.6	0.8	-0.1	0.1	0.3	-0.7	1	1
Q96C19	HUMAN EF-hand domain-containing protein D2	EFHD2	0.1	0.6	0.6	1.1	0.1	0.0	0.6	-0.1	1	1
Q93034	HUMAN Cullin-5	CUL5	0.9	0.6	0.6	0.3	-0.6	-0.9	-1.1	0.1	5	7
F1RYY6	PIG Transaldolase (Fragment)	TALDO1	-0.6	0.3	1.3	1.4	-0.5	-0.4	-0.3	-0.1	9	6
I3LGJ4	PIG Uncharacterized protein	PITPNB	0.7	0.5	0.6	0.6	-1.0	-0.9	-1.5	-0.5	4	1
I3LRK9	PIG Uncharacterized protein	PIGS	2.0	0.5	-0.2	0.1	2.1	1.5	0.4	-0.9	2	1
P79385	PIG Lactadherin	MFGE8	1.4	0.4	0.2	0.3	1.6	1.6	1.5	0.6	3	2
F1SII4	PIG Uncharacterized protein	GARS	0.8	0.3	0.3	1.0	0.3	0.6	-0.7	-0.6	13	3
P14460	PIG Fibrinogen alpha chain (Fragment)	FGA	0.3	1.0	0.5	0.6	-1.1	-1.0	2.3	3.4	2	2
Q2YGT9	PIG 60S ribosomal protein L6	RPL6	-0.1	-0.1	1.0	1.6	-0.5	-0.9	0.1	1.4	14	11
F1SI06	PIG Uncharacterized protein	HDLBP	0.4	0.0	0.7	1.3	-0.1	0.9	0.4	0.4	18	4
F1RZR6	PIG Uncharacterized protein	CUTA	1.1	0.7	0.6	0.0	2.3	3.5	-0.7	-3.7	3	1
F1S1U5	PIG Uncharacterized protein	GORASP2	-0.1	0.3	1.3	0.9	0.7	0.5	5.5	13.0	3	1
F1SHF4	PIG Uncharacterized protein	SCFD1	1.0	0.5	0.3	0.4	1.9	1.2	-0.9	-1.3	6	4
F2Z5F5	PIG 40S ribosomal protein S8 (Fragment)	RPS8	-0.1	0.1	1.0	1.3	0.0	1.0	1.5	1.9	8	5
F1RP96	PIG Uncharacterized protein	CD33	-0.6	0.8	0.5	1.6	0.8	1.8	1.5	0.4	1	1
P61160	HUMAN Actin-related protein 2	ACTR2	0.2	0.4	0.8	1.0	0.6	1.4	1.0	1.0	13	6
P12107	HUMAN Collagen alpha-1(XI) chain	COL11A1	0.5	-0.1	0.8	1.2	0.4	-0.2	1.3	1.8	2	1
F1RKM0	PIG Uncharacterized protein	LMNB1	0.2	0.5	0.7	1.0	0.0	0.1	-0.2	-0.1	33	16
P09651-2	HUMAN Isoform A1-A of Heterogeneous nuclear ribonucleoprotein A1	HNRNPA1	0.0	0.2	0.9	1.2	0.1	1.3	0.2	-1.4	8	8
Q7M3B0	PIG Actin beta (Fragments)	NA	0.2	0.3	0.8	0.9	0.4	0.8	1.2	-0.5	1	1
Q9G1G3	PIG Cytochrome c oxidase subunit 2	COII	1.5	1.5	0.2	-0.9	0.2	-0.2	-0.9	-1.0	2	2

Q29561	PIG UMP-CMP kinase	CMPK1	0.0	0.5	0.8	1.0	-0.4	-0.9	-0.4	-1.0	6	4
F1SDQ5	PIG Uncharacterized protein	EMILIN1	0.7	0.8	0.2	0.6	1.2	0.6	0.6	-0.7	6	1
F1SQ46	PIG Uncharacterized protein	YBX3	0.0	0.7	0.8	0.7	1.5	2.3	1.4	3.4	6	8
Q15907	HUMAN Ras-related protein Rab-11B	RAB11B	1.0	0.7	0.4	0.3	0.9	1.3	0.4	-0.4	1	1
F1SMW7	PIG Uncharacterized protein	SERPINB2	-0.6	0.0	1.2	1.7	-0.2	0.7	1.2	0.5	17	14
Q6Q7J2	PIG Rab GDP dissociation inhibitor beta	GDI2	0.2	0.4	0.7	1.0	-0.4	0.0	-0.5	-0.8	16	12
F1RHJ1	PIG Uncharacterized protein	MRPL24	0.2	-0.3	2.4	-0.1	0.2	-0.1	0.3	-0.2	1	4
I3LQS2	PIG Uncharacterized protein	NT5C	0.2	-0.1	1.0	1.1	0.0	-0.7	0.0	-0.4	1	1
P15981	PIG SLA class II histocompatibility antigen, DQ haplotype D alpha chain	NA	0.2	0.9	0.8	0.3	1.2	1.6	-1.6	-1.9	3	1
P84085	HUMAN ADP-ribosylation factor 5	ARF5	0.0	0.2	0.6	1.6	0.1	0.5	-0.6	-0.4	1	1
B8XSJ1	PIG ER lipid raft-associated 1	ERLIN1	0.9	0.7	0.4	0.3	0.3	-0.2	-0.8	-0.3	3	2
F1SM72	PIG Uncharacterized protein (Fragment)	NCAM1	0.3	0.4	0.8	0.8	0.3	1.0	0.4	0.0	11	6
I3LQK1	PIG Uncharacterized protein	LOC100738707	0.3	0.7	1.1	0.2	-0.3	-0.2	0.5	-0.2	1	2
F1RMG0	PIG Uncharacterized protein	FAM111A	0.5	0.4	0.4	1.0	0.5	0.7	1.9	3.2	2	1
I3LGD4	PIG Uncharacterized protein	NA	0.5	0.1	0.6	1.0	0.1	-0.1	0.2	-0.3	22	13
Q9H341	HUMAN Olfactory receptor 51M1	OR51M1	1.3	1.1	0.4	-0.6	-0.5	-0.9	-1.6	-0.5	1	1
Q15651	HUMAN High mobility group nucleosome-binding domain-containing protein 3	HMGN3	-0.7	1.0	0.8	1.1	-0.1	0.6	0.3	2.6	1	1
E1CAJ5	PIG Glucose regulated protein 58	grp-58	-0.4	0.2	0.9	1.6	0.2	0.2	0.3	0.4	33	29
Q29375	PIG 60S ribosomal protein L7a (Fragment)	RPL7A	-0.1	-0.5	1.1	1.8	-0.7	0.2	0.8	1.7	4	3
F1SS96	PIG Uncharacterized protein	CERS2	1.1	0.7	0.2	0.3	0.3	0.0	-0.1	0.4	1	1
A6NI56	HUMAN Coiled-coil domain-containing protein 154	CCDC154	1.2	0.7	0.3	0.0	0.7	1.1	3.0	0.7	1	2
Q49I35	PIG Galectin-1	LGALS1	-1.0	-0.6	1.3	2.5	1.4	0.3	0.3	-2.5	5	5
F1RZJ9	PIG Uncharacterized protein	TACC1	-0.9	0.1	1.2	1.8	2.3	-1.4	1.5	-1.8	1	1
F1SR90	PIG Selenoprotein S	VIMP	2.3	0.9	-0.4	-0.6	1.1	1.4	3.2	2.4	1	1
F1SN51	PIG Uncharacterized protein	INPP1	0.5	0.6	0.6	0.6	-0.3	1.1	0.5	-0.8	1	1
Q9GKE8	PIG Leukocyte surface antigen CD47	CD47	0.3	0.6	0.9	0.4	1.5	0.4	0.3	0.7	1	1
F1RZ25	PIG Uncharacterized protein	MPP1	0.8	0.1	0.7	0.6	-0.9	-0.6	-0.6	-0.4	3	1
F1SHC0	PIG Uncharacterized protein	PRPH	0.8	1.2	0.5	-0.3	0.4	0.3	1.8	0.9	1	1
Q9TT35	PIG Thyroxine-binding globulin	SERPINA7	0.6	0.4	-0.2	1.5	-1.4	-0.7	-1.4	-1.7	1	1
F1RVC9	PIG Uncharacterized protein (Fragment)	HNRNPD	0.7	0.5	0.3	0.8	1.1	1.3	0.4	-1.2	4	3
I3LTH4	PIG Cytosolic Fe-S cluster assembly factor NUBP1	NUBP1	1.1	0.8	0.3	0.0	0.9	1.7	-0.1	-0.8	1	1
F1SJS8	PIG Uncharacterized protein	TAGLN	-0.7	-0.4	0.8	2.5	-0.6	0.9	4.5	1.9	23	14
F1RYU9	PIG Uncharacterized protein	USO1	0.2	0.4	0.7	0.8	0.9	0.5	0.2	0.2	7	10
F1RS63	PIG Uncharacterized protein	SATB1	-0.1	2.6	0.4	-0.8	-0.1	0.3	-0.1	0.4	1	1
I3LR97	PIG Uncharacterized protein	NA	0.6	0.5	0.8	0.3	0.8	0.7	-1.1	-1.3	1	1
F1SIM4	PIG Uncharacterized protein	ELMO1	0.2	0.3	1.0	0.7	0.4	1.2	-0.4	-0.9	1	1
Q8WU17	HUMAN E3 ubiquitin-protein ligase RNF139	RNF139	0.1	0.5	0.7	0.9	0.3	0.1	1.4	0.1	1	1
F1SB67	PIG Uncharacterized protein	M6P/IGF2R	0.5	0.7	0.6	0.4	0.6	0.8	-1.1	0.8	3	1
F1SPG1	PIG Uncharacterized protein	H1FX	1.3	0.5	0.0	0.4	0.0	-0.4	0.9	1.5	8	6
Q9UKX5	HUMAN Integrin alpha-11	ITGA11	1.7	0.7	0.0	-0.1	1.8	0.8	-0.5	-0.6	1	1

F1SLF6	PIG Uncharacterized protein	CCT7	0.5	0.0	0.7	1.0	-0.1	-0.4	0.1	0.9	12	11
Q7Z4W1	HUMAN L-xylulose reductase	DCXR	1.0	-0.1	0.4	0.9	-0.8	-0.4	-1.1	-2.0	1	2
I3LJC3	PIG Uncharacterized protein (Fragment)	NA	2.1	0.5	-0.1	-0.3	1.5	-0.3	-1.4	-4.2	1	1
F1SH20	PIG Uncharacterized protein	TRHDE	0.5	0.0	0.6	1.0	-0.8	-0.9	-0.9	0.8	1	1
I3LGU2	PIG Uncharacterized protein	CCDC114	0.0	0.8	0.7	0.7	-0.5	-0.4	2.4	3.2	2	1
F1RVD4	PIG Uncharacterized protein	SEC31A	0.1	0.0	0.7	1.4	-1.4	-0.5	-0.3	1.0	14	5
Q29214	PIG 60S acidic ribosomal protein P0	RPLP0	-0.2	-0.1	1.0	1.5	0.1	-0.1	-0.6	-0.9	12	8
P62263	HUMAN 40S ribosomal protein S14	RPS14	0.3	0.2	0.7	1.0	0.1	0.2	-0.2	0.5	6	4
O75628	HUMAN GTP-binding protein REM 1	REM1	1.0	0.2	0.6	0.4	0.4	-0.2	0.0	-1.6	1	1
F1RQU2	PIG Uncharacterized protein	HSPCB	-0.3	0.2	1.1	1.1	-0.3	0.4	0.5	0.9	13	10
A0T4C3	HUMAN Truncated MHC class II antigen (Fragment)	HLA-DRB1	0.7	1.2	0.7	-0.4	1.1	0.7	-0.3	-0.7	1	1
B6ICY1	PIG MHC class I antigen	SLA-2	1.0	0.7	0.3	0.2	0.8	0.1	-0.9	-1.5	2	1
F1S6V4	PIG Uncharacterized protein (Fragment)	EIF4G2	0.3	0.4	0.7	0.8	-0.5	-0.2	-0.1	-0.1	10	3
F1SAJ5	PIG DEAD (Asp-Glu-Ala-Asp) box helicase 6	DDX6	0.4	0.0	0.6	1.1	-0.7	-1.0	-1.1	-0.1	3	4
I3LEX0	PIG 40S ribosomal protein S9 (Fragment)	RPS9	-0.3	-0.6	1.2	1.8	-0.8	0.6	0.2	0.4	5	6
K7GML1	PIG Uncharacterized protein	RBBP7	0.4	0.1	0.8	0.9	-0.1	-0.1	0.3	-1.4	3	1
F1SSX4	PIG Uncharacterized protein (Fragment)	LOC100620549	1.2	0.4	0.3	0.3	0.6	0.0	-0.3	1.3	2	2
F1S4J5	PIG Uncharacterized protein	DAPK1	0.5	0.2	0.6	0.8	0.0	0.4	0.0	-0.3	1	1
P61960	HUMAN Ubiquitin-fold modifier 1	UFM1	-0.1	0.6	0.8	0.8	-0.1	0.0	-0.7	0.3	1	2
Q8MJ14	PIG Glutathione peroxidase 1	GPX1	0.4	0.5	0.6	0.7	0.0	0.2	-0.3	0.3	8	4
F1S2E1	PIG Uncharacterized protein	VMA21	0.4	0.9	0.4	0.3	0.9	0.7	0.7	1.9	1	1
M3TYC1	PIG Eukaryotic translation initiation factor 3 subunit B	EIF3B	0.2	0.4	0.8	0.8	0.6	-0.4	-0.7	-0.5	11	4
F1S939	PIG Uncharacterized protein	MAP1S	0.3	0.3	0.7	0.8	1.2	1.0	0.0	0.0	7	1
F1SR61	PIG Uncharacterized protein	LOC100511787	0.6	0.5	0.6	0.4	0.6	0.6	-0.4	-1.0	1	2
I3LVS6	PIG Uncharacterized protein	LOC100523123	-0.3	0.3	0.4	1.7	-1.0	-0.6	0.1	-0.7	1	1
I3LBB2	PIG Vacuolar protein sorting-associated protein 35 (Fragment)	VPS35	0.5	0.3	0.7	0.7	0.0	0.3	-0.7	-0.5	11	7
K7GLK5	PIG Uncharacterized protein	NA	0.1	0.3	0.6	1.0	-0.7	-0.7	-0.4	-0.2	4	4
B0FWP5	PIG Sirtuin 2	SIRT2	1.1	0.6	0.2	0.2	0.3	0.7	-0.6	-0.8	3	3
P62314	HUMAN Small nuclear ribonucleoprotein Sm D1	SNRPD1	0.2	0.4	0.8	0.7	-0.2	0.1	0.7	-0.3	1	1
P61009	HUMAN Signal peptidase complex subunit 3	SPCS3	0.5	0.8	0.3	0.5	0.8	0.7	-0.3	0.0	3	1
I3LRJ5	PIG Uncharacterized protein (Fragment)	CCDC64B	1.5	0.4	0.1	0.1	0.1	0.6	-0.2	0.1	2	1
I3LLY4	PIG Uncharacterized protein	NQO2	1.0	0.7	0.2	0.1	1.6	1.8	1.7	-0.6	2	2
Q007T2-2	PIG Isoform 1 of Cell division control protein 42 homolog	CDC42	0.2	-0.2	0.7	1.3	-1.5	-1.2	0.4	2.1	3	2
F1RHH8	PIG Uncharacterized protein (Fragment)	PRPF8	-0.3	0.1	0.9	1.3	-0.5	-0.4	-0.5	-0.8	2	2
K7GKY4	PIG Uncharacterized protein	ANK3	1.1	0.3	0.6	0.0	1.4	0.1	-0.1	0.5	2	4
F8W0P7	HUMAN ATP synthase subunit beta, mitochondrial (Fragment)	ATP5B	0.8	0.4	0.3	0.6	1.4	0.8	0.5	-1.8	1	2
I3LFS8	PIG Uncharacterized protein (Fragment)	FLOT2	1.1	0.6	0.3	0.0	1.0	1.1	-0.1	0.0	11	7
F1SUA3	PIG Uncharacterized protein	ZC3H6	1.0	0.9	0.8	-0.6	-0.7	-0.3	-0.1	0.1	1	1
I3LLJ7	PIG Uncharacterized protein	NA	-0.6	0.1	1.0	1.6	-0.6	-0.7	-1.5	0.0	1	1
I3LSH8	PIG Uncharacterized protein (Fragment)	NPTN	-0.2	0.2	1.0	1.0	0.4	0.0	0.2	0.3	2	1

Q8IVT2	HUMAN Mitotic interactor and substrate of PLK1	MISP	1.7	0.4	-0.1	0.1	2.3	1.8	1.7	-0.6	1	1
R9Y4B7	HUMAN NADH-ubiquinone oxidoreductase chain 5	ND5	0.2	-0.2	0.7	1.3	-0.8	-0.8	-0.1	1.3	1	1
P14287	PIG Osteopontin	SPP1	0.0	0.0	0.5	1.5	3.3	-0.3	0.0	-4.2	6	1
F1RST0	PIG Uncharacterized protein	HSPH1	0.8	1.1	0.2	0.0	-0.5	-1.1	0.1	1.0	7	1
B6A7R0	PIG Tubulin alpha 3	NA	-0.7	-0.1	1.2	1.7	-0.7	-1.4	-1.4	-2.4	2	2
F1S8Z6	PIG Uncharacterized protein (Fragment)	LOC100519326	0.5	0.6	0.4	0.5	0.4	0.9	1.4	0.6	1	1
F1RGM3	PIG Uncharacterized protein	TXNDC17	0.2	0.5	0.8	0.6	0.4	1.1	0.0	-1.6	3	4
I3L5J0	PIG Uncharacterized protein	NA	0.9	0.0	0.6	0.4	-0.3	-0.6	-0.3	-0.5	2	1
Q99613	HUMAN Eukaryotic translation initiation factor 3 subunit C	EIF3C	0.6	0.3	0.5	0.7	0.3	0.0	-0.2	-0.2	8	6
P81693	PIG Low molecular weight phosphotyrosine protein phosphatase	ACP1	1.1	0.7	0.3	-0.1	1.5	2.1	0.8	-0.1	2	2
I3L9F5	PIG Uncharacterized protein (Fragment)	PCYOX1	1.0	1.0	0.3	-0.2	0.0	0.8	-0.6	-0.5	7	5
O62839	PIG Catalase	CAT	-0.1	1.2	0.9	0.0	0.3	0.1	0.4	-0.4	18	11
F1S912	PIG Uncharacterized protein	FKBP8	1.3	0.6	0.2	0.0	0.0	-0.6	-0.8	-0.1	3	3
A9YUA9	PIG Alpha-2-macroglobulin receptor-associated protein	NA	-0.5	0.0	1.0	1.5	-0.9	-3.0	-1.0	-1.4	1	1
F1S0J8	PIG Uncharacterized protein	KRT19	0.5	0.7	0.7	0.1	0.3	-0.3	0.2	3.9	4	2
P13535	HUMAN Myosin-8	MYH8	1.3	1.2	0.2	-0.7	1.2	1.0	0.7	1.0	5	2
F1RK74	PIG Uncharacterized protein	SLC25A1	0.7	0.4	0.3	0.5	-0.1	0.0	0.0	0.5	1	2
I3LAT6	PIG Uncharacterized protein	LOC100739149	1.3	0.9	-0.1	0.0	1.0	1.2	1.1	1.4	1	2
F1SD83	PIG Uncharacterized protein	NACC1	1.0	0.4	0.2	0.3	-0.1	0.1	0.8	0.3	1	2
F1RIF3	PIG Uncharacterized protein	FAH	0.6	0.2	0.6	0.6	-0.6	-0.8	0.8	-0.4	4	1
F1SV45	PIG Uncharacterized protein	LOC100525755	0.1	-0.1	0.8	1.1	-0.3	-0.5	-0.3	-0.2	2	3
F1S645	PIG Uncharacterized protein (Fragment)	KCNT2	1.3	1.2	0.0	-0.4	-0.7	0.9	-0.5	1.5	1	1
P35580	HUMAN Myosin-10	MYH10	0.6	0.3	0.3	0.8	0.6	0.2	0.0	0.0	5	4
A6M931	PIG Eukaryotic initiation factor 4A-III	EIF4A3	-0.8	-0.4	1.3	1.9	-1.2	-0.1	0.0	0.2	6	4
F1SE30	PIG Uncharacterized protein	ANK1	0.1	1.0	1.0	0.0	-0.6	-0.6	-0.1	0.2	19	7
F1SDW3	PIG Uncharacterized protein	TIMM23	2.4	0.4	-0.2	-0.6	2.5	2.0	0.2	-0.6	1	1
O14782	HUMAN Kinesin-like protein KIF3C	KIF3C	0.0	0.4	1.0	0.6	-1.1	-1.2	-1.3	-0.6	1	1
Q9H987	HUMAN Synaptopodin 2-like protein	SYNPO2L	1.0	0.5	0.4	0.1	1.0	1.3	-0.1	0.3	1	2
P62280	HUMAN 40S ribosomal protein S11	RPS11	-0.6	-0.4	1.1	1.9	-0.5	0.5	1.1	1.1	7	6
F1RLM4	PIG Uncharacterized protein (Fragment)	DAP3	1.1	0.4	0.5	0.0	0.1	0.8	1.4	-0.8	1	2
B4DCU2	PIG Calmodulin 1 (Fragment)	CALM1	-0.2	0.8	0.4	1.0	2.9	2.3	-0.2	-3.5	10	4
F1SJT1	PIG Uncharacterized protein	EXOG	0.9	0.8	0.0	0.2	1.6	3.0	1.5	-0.8	1	2
A2NW98	HUMAN Rheumatoid factor light chain variable region (Fragment)	NA	0.3	0.5	1.2	0.1	-0.6	-0.5	3.2	0.8	1	1
I3LLS7	PIG Uncharacterized protein (Fragment)	XPO1	0.6	0.6	0.4	0.3	-0.2	-0.8	-1.3	-1.4	10	7
Q9GMB0	PIG Dolichyl-diphosphooligosaccharide--protein glycosyltransferase subunit 1	RPN1	-0.1	0.2	0.8	1.1	-0.4	-0.4	-0.6	0.5	19	15
F1S1V1	PIG Uncharacterized protein	SSB	-0.2	0.0	0.9	1.2	0.0	0.5	0.6	0.3	10	4
P08123	HUMAN Collagen alpha-2(I) chain	COL1A2	-1.1	1.1	0.5	1.5	1.6	3.5	1.4	1.3	3	2
F1S7W0	PIG Uncharacterized protein (Fragment)	AKAP12	0.8	0.3	0.1	0.7	1.3	1.1	-0.2	-0.2	12	3
Q6GWX0	PIG Probable ATP-dependent RNA helicase DDX4	DDX4	0.6	0.0	0.5	0.8	2.2	0.8	-0.7	-0.3	1	1
P61019	HUMAN Ras-related protein Rab-2A	RAB2A	0.8	0.6	0.3	0.2	0.3	0.4	1.0	-0.4	1	1

F1SA98	PIG Uncharacterized protein (Fragment)	YWHAQ	-0.2	0.0	0.7	1.5	-0.3	0.2	1.1	0.2	7	7
F1S5Z3	PIG Uncharacterized protein	SARS	-0.4	0.4	0.8	1.2	0.0	0.3	-0.9	-0.8	7	4
F1SB63	PIG T-complex protein 1 subunit alpha	TCP1	0.4	0.3	0.5	0.8	0.2	0.6	0.3	0.9	19	14
B1A8Z3	PIG Phosphorylase	NA	0.3	0.4	0.6	0.6	0.0	-0.3	-0.3	0.3	20	16
I3LVK2	PIG Uncharacterized protein (Fragment)	MAPK3	0.6	0.5	0.7	0.2	-0.6	-1.1	-1.2	-1.5	2	1
P04163	PIG Protein S100-A10	S100A10	-1.0	0.1	0.9	1.9	-1.2	-0.5	0.5	0.7	1	1
I3LBY0	PIG Uncharacterized protein	ARCN1	-0.1	0.1	0.6	1.3	0.2	0.4	-0.1	-0.2	9	2
F1S085	PIG Methionine aminopeptidase (Fragment)	METAP1D	0.8	0.5	0.2	0.4	-0.3	-1.2	0.1	0.1	1	1
P55083	HUMAN Microfibril-associated glycoprotein 4	MFAP4	-0.5	0.6	0.5	1.3	-1.6	-0.7	0.9	1.6	3	2
K7GMK5	PIG Uncharacterized protein	ILF2	0.2	0.1	0.6	1.0	0.1	-0.5	-2.0	-0.9	7	4
Q8MIR4	PIG Bifunctional coenzyme A synthase	COASY	0.6	0.6	0.7	0.0	0.1	-1.0	-1.1	0.9	1	3
I3LHY0	PIG Uncharacterized protein (Fragment)	NA	0.3	0.9	0.4	0.3	0.4	1.6	0.7	0.1	5	5
P79275	PIG Rho A (Fragment)	NA	0.7	0.2	0.4	0.6	0.7	0.7	-0.1	-0.5	1	1
F1SAF0	PIG Dihydrolipoyl dehydrogenase	DLD	1.0	1.2	0.1	-0.4	0.1	0.2	-1.7	-1.2	1	1
P12277	HUMAN Creatine kinase B-type	CKB	0.4	-0.4	0.1	1.7	2.2	1.8	0.3	0.0	6	4
P23246	HUMAN Splicing factor, proline- and glutamine-rich	SFPQ	0.0	0.0	0.7	1.2	-0.6	-0.3	-0.4	0.0	13	12
P19205	PIG Acylamino-acid-releasing enzyme	APEH	0.5	0.8	0.5	0.0	0.7	0.7	0.8	-0.3	5	8
I3LLE6	PIG Uncharacterized protein	FAM98B	0.8	0.5	0.2	0.4	0.7	0.2	-0.4	-0.8	2	1
F1S3U9	PIG Uncharacterized protein	PRDX1	-0.3	-0.4	1.0	1.6	0.4	-0.8	0.5	-1.7	12	8
F1RF77	PIG Uncharacterized protein (Fragment)	PLOD1	0.8	1.1	-0.1	0.1	0.7	1.3	-0.2	-1.4	1	1
I3LSD1	PIG Sorting nexin (Fragment)	SNX9	0.3	0.5	0.5	0.5	0.8	2.3	0.9	0.4	2	2
F1SJI0	PIG Uncharacterized protein	ESPNL	0.8	0.9	0.1	0.1	0.6	-0.3	0.7	0.1	1	1
I3LPQ3	PIG Uncharacterized protein	KCNJ13	0.2	0.7	0.5	0.5	0.5	0.3	-0.2	-1.1	1	1
O75369	HUMAN Filamin-B	FLNB	0.5	0.2	0.4	0.8	0.2	1.2	0.2	0.3	21	5
K7GQM4	PIG Uncharacterized protein	PRAF2	0.5	0.4	0.5	0.4	0.0	-0.1	0.5	0.6	1	1
Q59G56	HUMAN Two pore segment channel 2 variant (Fragment)	NA	0.5	0.4	0.5	0.4	-0.8	-1.0	0.2	0.2	1	1
P22626	HUMAN Heterogeneous nuclear ribonucleoproteins A2/B1	HNRNPA2B1	-0.2	0.0	0.9	1.2	-1.2	0.5	-0.1	0.1	7	7
I3LLG3	PIG Uncharacterized protein	SUMO3	0.0	0.2	0.6	1.1	0.6	0.0	0.2	-1.3	1	1
I3LVJ7	PIG Uncharacterized protein (Fragment)	PSMA7	0.3	-0.2	0.9	0.9	-0.9	-1.7	-1.0	-1.6	4	4
F1S087	PIG Uncharacterized protein	DYNC1I2	-0.6	-0.5	1.0	1.9	0.0	-0.3	-0.2	1.1	9	6
P55081	HUMAN Microfibrillar-associated protein 1	MFAP1	-0.3	-0.2	0.9	1.5	0.8	1.3	-0.6	-0.5	2	1
Q71V07	HUMAN Signal recognition particle subunit SRP72	NA	-0.3	0.0	0.6	1.6	-0.1	1.3	-1.2	1.9	1	1
F1RRU6	PIG Uncharacterized protein (Fragment)	TLK2	1.1	0.7	0.5	-0.5	-0.2	0.0	-1.0	-0.2	1	1
F1S3D5	PIG Uncharacterized protein	LMAN2	0.1	0.3	0.7	0.8	1.5	1.4	1.0	0.6	6	2
Q9UQ80	HUMAN Proliferation-associated protein 2G4	PA2G4	-0.2	-0.4	0.9	1.5	-1.3	-2.0	-1.7	-1.6	1	1
P62750	HUMAN 60S ribosomal protein L23a	RPL23A	-0.2	-0.1	0.7	1.4	0.5	0.8	0.8	2.3	9	4
K7GT02	PIG Uncharacterized protein	CCDC22	0.5	0.8	0.3	0.3	-0.6	-1.0	-1.7	-1.8	3	1
Q9TVC1	PIG V-type proton ATPase subunit H	ATP6V1H	0.6	0.6	0.4	0.2	1.1	1.5	1.2	0.8	1	2
B9ZSM8	PIG CD90 protein	CD90	-0.1	0.8	0.5	0.6	1.1	1.4	1.2	-0.1	2	1
F1RR13	PIG Uncharacterized protein	FAM83A	-0.5	0.9	1.6	-0.1	-0.9	-1.4	0.6	-0.6	2	1

Q12984	HUMAN Putative uncharacterized protein	NA	2.8	0.7	-0.7	-1.0	0.5	-0.2	0.1	0.3	1	1
F1RQI1	PIG Uncharacterized protein	COX7A2	0.6	0.5	0.6	0.1	-1.7	-1.3	-2.2	-0.8	2	2
F1SAV8	PIG Uncharacterized protein	LOC100518203	0.5	0.3	0.3	0.7	1.1	0.6	1.7	1.1	1	1
Q13765	HUMAN Nascent polypeptide-associated complex subunit alpha	NACA	0.5	0.4	0.6	0.3	1.0	1.2	0.6	0.7	4	4
B2CCY7	PIG Nuclear matrix protein 200	nmp-200	0.1	0.3	0.6	0.9	0.9	0.5	0.8	1.0	2	3
F1SUN0	PIG Uncharacterized protein (Fragment)	ARRB1	0.1	-0.3	0.8	1.2	-1.1	-2.0	-2.3	0.7	1	1
F1SF93	PIG Uncharacterized protein	LOC100523850	0.9	0.7	0.3	-0.1	-0.2	-0.6	0.1	-0.3	1	1
Q0ZHH9	PIG Low density lipoprotein receptor-related protein-associated protein 1 (Fragment)	LREAP1	-0.8	0.1	0.9	1.6	0.1	0.5	0.4	0.7	1	1
I3LEC2	PIG Uncharacterized protein	PCBP1	0.5	0.0	0.4	0.9	1.2	1.7	2.1	0.4	3	4
F1SQN1	PIG T-complex protein 1 subunit delta	CCT4	0.0	0.1	0.7	1.0	0.4	0.4	-0.2	-0.1	16	14
F1SB18	PIG Uncharacterized protein	NA	0.3	0.4	0.3	0.7	0.5	0.0	-0.1	-0.2	4	1
P61457	HUMAN Pterin-4-alpha-carbinolamine dehydratase	PCBD1	1.2	0.3	0.2	0.1	0.8	-0.1	-0.4	-0.6	2	1
I3L7T6	PIG Histone H2A	H2AFX	0.7	0.1	0.3	0.6	-0.6	-0.1	-0.8	1.8	1	1
Q29197	PIG 40S ribosomal protein S9 (Fragment)	RPS9	-0.2	-0.6	1.1	1.5	-0.7	-0.2	0.2	0.3	10	7
F1SGA9	PIG Uncharacterized protein	DNAJC19	1.0	0.5	-0.1	0.3	0.6	1.1	0.1	0.9	1	3
Q6QAS5	PIG 60S ribosomal protein L12 (Fragment)	RPL12	-0.1	-0.3	0.9	1.3	-0.4	-0.3	0.6	0.5	5	6
A5A8V7	PIG Heat shock 70 kDa protein 1-like	HSPA1L	0.1	0.0	0.7	0.9	0.2	1.4	0.3	-0.6	1	1
F1SFZ8	PIG Uncharacterized protein	TLN1	0.0	0.1	0.7	1.0	0.3	0.5	-0.1	0.5	71	50
I3LD13	PIG Uncharacterized protein	LOC100624417	-0.1	0.0	0.8	1.0	-0.5	-0.3	-0.2	-0.3	6	1
F1RUR3	PIG Uncharacterized protein	CCNY	0.9	0.6	0.2	0.1	1.6	1.0	-0.1	-0.5	1	1
B4DFN7	HUMAN cDNA FLJ59676, highly similar to Myosin-18A	NA	1.5	0.9	-0.2	-0.4	-0.5	-0.9	-0.5	-1.0	1	1
F8SIP2	PIG EGF-containing fibulin-like extracellular matrix protein 1	EFEMP1	0.4	0.4	0.4	0.5	1.5	1.8	0.5	-0.5	2	1
F1RJU9	PIG Uncharacterized protein (Fragment)	SEPT8	0.9	0.1	0.2	0.5	-0.1	0.1	-1.2	-1.4	4	3
P13647	HUMAN Keratin, type II cytoskeletal 5	KRT5	1.1	0.3	0.0	0.3	0.0	-0.7	-0.1	7.1	2	2
A5GFX6	PIG Tubulin, beta family 1	TUBB1	0.4	0.5	0.1	0.7	-0.5	0.9	5.3	-0.1	2	1
I3LHB2	PIG Uncharacterized protein	RPA1	1.1	-0.2	0.3	0.5	1.9	1.3	-0.2	-0.9	1	1
Q02833	HUMAN Ras association domain-containing protein 7	RASSF7	1.7	0.4	0.0	-0.4	-0.3	0.1	0.9	0.4	1	1
F1SBE5	PIG Uncharacterized protein (Fragment)	PTGIS	1.1	0.5	0.0	0.1	-1.0	-1.0	-0.5	1.4	3	1
F1S4M2	PIG Uncharacterized protein (Fragment)	LOC100738304	-0.2	0.1	0.7	1.2	-0.3	-0.4	0.2	0.3	6	2
F1SUM7	PIG 40S ribosomal protein S3 (Fragment)	RPS3	-0.1	-0.4	0.8	1.4	0.7	1.2	1.3	1.5	10	7
P62805	HUMAN Histone H4	HIST1H4A	-0.6	0.5	0.7	1.1	-1.8	-0.9	0.4	0.3	10	11
F1SPI1	PIG Uncharacterized protein	TMEM43	0.6	0.3	0.3	0.6	-0.6	-0.9	-0.5	-0.8	4	4
I3LIK6	PIG Uncharacterized protein	NA	-0.3	-0.4	0.7	1.7	-0.8	-0.8	0.4	0.1	9	2
F1SMD7	PIG Uncharacterized protein	STOM	0.2	0.7	0.5	0.3	0.1	-0.4	-0.4	-0.4	5	3
Q8N901	HUMAN cDNA FLJ38626 fis, clone HEART2009599	NA	0.3	0.6	0.5	0.2	-0.2	-0.2	-0.3	-1.3	2	1
P01598	HUMAN Ig kappa chain V-I region EU	NA	0.4	0.7	0.4	0.2	1.9	2.0	2.6	0.4	1	1
Q04446	HUMAN 1,4-alpha-glucan-branching enzyme	GBE1	0.6	0.3	0.4	0.4	0.5	0.2	-0.5	-0.7	1	1
F1RG16	PIG Uncharacterized protein	HNRNPF	-0.2	0.1	0.9	0.9	0.5	0.9	-0.1	-1.1	8	5
F1RKC0	PIG Uncharacterized protein (Fragment)	HIP1	1.4	0.2	-0.1	0.2	0.8	0.9	0.7	0.1	3	4
F6Q5P0	PIG 40S ribosomal protein S13	RPS13	0.1	0.2	0.8	0.6	-0.1	-0.2	-0.4	-0.7	3	2

Q15413	HUMAN Ryanodine receptor 3	RYR3	1.3	1.2	-0.2	-0.6	1.5	1.1	0.2	-1.1	6	2
Q45VM7	HUMAN Mutant desmin	NA	1.6	0.5	0.0	-0.4	3.4	1.7	1.9	0.2	1	1
B3VMR0	PIG 5-aminoimidazole-4-carboxamide ribonucleotide formyltransferase/IMP cyclohydrolase	purH	0.2	0.2	0.7	0.6	1.3	0.3	-0.1	0.5	4	2
A9ED96	PIG CArG-binding factor A	CBF-A	-0.2	0.3	0.9	0.8	1.2	1.7	2.1	-0.4	4	1
F2Z4X6	PIG Uncharacterized protein (Fragment)	TOMM20	1.0	0.3	0.0	0.3	1.0	0.3	-0.2	0.2	1	2
F1SUE4	PIG Uncharacterized protein	ASPN	0.3	0.3	0.0	1.1	-0.4	-0.7	-1.5	-0.8	8	7
I3L9F7	PIG Importin subunit alpha (Fragment)	KPNA1	0.9	0.6	0.2	0.0	0.3	0.5	-0.3	-0.5	1	1
P32969	HUMAN 60S ribosomal protein L9	RPL9	-0.4	-0.2	0.9	1.4	0.4	0.7	0.9	0.6	7	5
F1SRK6	PIG Endoplasmin	HSP90B1	0.2	0.1	1.0	0.4	1.3	1.4	2.7	0.1	1	1
I3LAI3	PIG Uncharacterized protein (Fragment)	NA	0.0	-1.1	0.8	1.9	1.6	0.5	0.8	1.5	1	1
Q9H390	HUMAN PRO2300	NA	0.2	0.3	0.7	0.4	0.1	-0.5	0.6	-0.5	1	1
A5A8V9	PIG Valyl-tRNA synthetase 2	VARS2	0.6	0.2	0.3	0.5	0.1	0.2	0.9	0.4	4	3
I3LIQ9	PIG Uncharacterized protein	NA	0.3	0.1	0.4	0.8	1.3	1.2	0.9	-0.2	7	2
I3LU70	PIG Uncharacterized protein	C1orf170	0.9	0.6	0.2	-0.1	0.1	1.5	-0.3	-1.7	1	2
Q9N0Y9	PIG Ubiquitous tropomodulin U-Tmod	TMOD3	0.2	0.6	0.4	0.5	0.6	-0.1	-0.8	0.2	10	1
F1SGS7	PIG Uncharacterized protein	NAT10	0.5	0.9	0.1	0.2	-0.3	-0.2	6.7	-1.1	1	1
Q702N8	HUMAN Xin actin-binding repeat-containing protein 1	XIRP1	0.8	0.5	0.3	0.1	0.8	1.0	-0.1	0.2	5	2
I3LNN3	PIG Uncharacterized protein	MKI67	0.5	0.4	0.5	0.3	-1.3	-1.7	-0.7	-0.9	1	1
F1S4Y9	PIG Uncharacterized protein	RALY	1.0	0.3	0.0	0.3	0.2	1.0	1.0	1.1	3	1
O75197	HUMAN Low-density lipoprotein receptor-related protein 5	LRP5	0.6	0.9	0.3	-0.1	-0.2	-0.1	-0.3	-0.6	1	1
P62936	PIG Peptidyl-prolyl cis-trans isomerase A	PPIA	-0.8	-0.3	1.0	1.7	-0.1	0.8	0.6	0.2	13	12
I3LMX3	PIG Uncharacterized protein	POLDIP2	0.8	0.1	0.4	0.3	-0.1	-0.5	0.7	-0.5	3	4
Q9GLP0	PIG Integrin beta-1	ITGB1	0.2	0.6	0.4	0.5	0.7	1.1	0.1	0.4	12	8
F1SPG9	PIG Uncharacterized protein	ESYT1	0.4	0.3	0.3	0.6	0.3	0.1	-1.3	-0.3	10	9
F1RZ71	PIG Uncharacterized protein	SEPT9	-0.5	0.0	0.7	1.4	0.5	2.2	-0.1	-0.2	7	1
O75436	HUMAN Vacuolar protein sorting-associated protein 26A	VPS26A	0.9	-0.1	0.1	0.7	0.0	-0.7	-0.7	0.4	1	1
F1S4P0	PIG Uncharacterized protein	STRN	1.1	0.3	0.2	-0.1	1.6	1.4	1.4	0.3	2	1
P31937	HUMAN 3-hydroxyisobutyrate dehydrogenase, mitochondrial	HIBADH	1.1	0.5	0.0	0.0	1.0	1.3	1.1	-0.1	2	2
F1SGJ3	PIG Uncharacterized protein	FLNB	1.0	0.2	0.0	0.4	0.2	-0.5	-0.2	-0.1	7	2
F1SH90	PIG Uncharacterized protein	CACNA1D	1.3	0.5	0.4	-0.5	1.0	0.4	-0.4	-1.4	1	1
Q29198	PIG Ribosomal protein S6 (Fragment)	NA	0.7	0.1	0.2	0.6	0.7	0.7	0.5	1.4	3	3
F1S9S0	PIG Uncharacterized protein (Fragment)	GRIK4	0.6	0.5	0.0	0.5	-0.1	1.3	0.5	0.1	1	1
F1S3J8	PIG Uncharacterized protein	MRPL4	1.0	0.5	0.3	-0.2	0.6	0.9	0.4	-0.8	1	1
Q13045	HUMAN Protein flightless-1 homolog	FLII	0.5	0.3	0.5	0.3	-2.9	-2.5	-0.1	0.1	6	3
Q29300	PIG NADH-ubiquinone oxidoreductase 49 KD subunit (Fragment)	NA	1.1	0.2	0.4	0.0	0.4	0.3	-0.2	-0.7	1	2
K7GMX3	PIG Uncharacterized protein	PDK2	0.9	0.5	0.5	-0.3	0.4	-0.5	-0.3	-0.3	1	1
I3LVF9	PIG Uncharacterized protein	VAMP3	1.2	0.8	-0.1	-0.3	1.9	2.9	0.9	-0.4	1	1
F1STY8	PIG Uncharacterized protein (Fragment)	RSF1	0.2	0.6	0.6	0.1	-1.4	-0.8	0.0	0.4	2	2
F1SPM8	PIG AP2-associated protein kinase 1	AAK1	0.9	0.3	0.1	0.3	0.6	0.1	0.3	1.0	4	3
I3L759	PIG Uncharacterized protein (Fragment)	NA	0.3	0.2	0.4	0.6	0.9	0.3	0.5	0.7	7	3

Q95KR6	PIG Mono (ADP-ribosyl)transferase (Fragment)	ART3	0.8	0.9	-0.1	0.0	-1.5	-2.2	-1.6	-1.5	3	1
F1RMJ9	PIG Oxysterol-binding protein	OSBP	0.4	0.2	0.4	0.5	-2.8	-4.2	-5.6	-4.4	3	1
F1S0V3	PIG Annexin (Fragment)	ANXA6	0.3	0.4	0.2	0.6	-0.5	-0.4	-0.1	0.0	32	26
F1SS95	PIG Uncharacterized protein	SETDB1	1.1	0.6	-0.1	0.0	-0.5	0.1	-2.1	-1.2	1	1
F1S7L8	PIG Uncharacterized protein	SH3GL1	0.9	0.1	0.4	0.2	-1.2	-0.8	-0.4	-0.1	2	1
Q8SPC2	PIG MHC class I antigen	SLA-3	0.1	0.0	0.8	0.6	1.6	0.7	1.0	0.0	5	4
F1RKX9	PIG Uncharacterized protein	CRKL	1.0	0.3	0.2	0.1	1.5	1.0	0.1	-1.1	4	3
F1SKU5	PIG Uncharacterized protein	STRBP	0.2	-0.2	0.3	1.2	-0.5	0.1	0.8	-0.3	3	1
I3LT93	PIG Uncharacterized protein	UROS	1.7	0.7	-0.1	-0.8	0.1	0.2	-1.0	-0.7	1	1
L0R849	HUMAN Enolase	EDARADD	0.9	0.0	0.3	0.4	3.0	1.7	-1.3	-1.8	2	1
Q9GKX6	PIG Aldose 1-epimerase	GALM	0.5	0.0	0.4	0.7	0.5	1.0	-0.3	-0.5	4	3
K7GL83	PIG Uncharacterized protein	ILF3	0.1	0.0	0.6	0.9	0.7	0.9	0.0	0.0	19	11
P31950	PIG Protein S100-A11	S100A11	-0.5	0.0	0.7	1.4	-1.5	-1.4	-0.5	-0.1	1	1
F1RLQ2	PIG Prelamin-A/C	LMNA	1.0	1.0	-0.6	0.2	0.1	1.1	-1.9	-1.0	1	1
Q29195	PIG 60S ribosomal protein L10	RPL10	-0.3	-0.4	0.8	1.4	-0.8	0.5	-0.1	0.5	6	6
O76024	HUMAN Wolframin	WFS1	0.6	0.2	0.9	-0.1	-0.6	-0.8	-1.6	-0.6	1	2
F1SU44	PIG Uncharacterized protein	NA	0.9	0.4	0.2	0.0	-0.1	0.8	-0.8	0.0	1	1
F1S1G8	PIG Amine oxidase	AOC3	0.0	0.5	0.5	0.5	0.0	-0.1	0.2	-0.5	9	9
B5LX40	PIG Adaptor protein phosphotyrosine interaction PH domain and leucine zipper containing 1	APPL1	1.0	0.1	0.2	0.3	-1.5	-0.8	-0.8	-1.2	3	1
I3LDA4	PIG Uncharacterized protein	PXDN	0.4	0.8	0.4	-0.1	0.7	0.5	-1.1	0.3	3	3
I3LM93	PIG Uncharacterized protein (Fragment)	TNPO1	-0.2	0.2	0.6	0.9	-0.6	0.3	-1.3	0.4	9	4
F1SE06	PIG Uncharacterized protein	LOC100626607	0.5	0.5	0.3	0.2	0.3	0.3	0.0	0.3	6	4
F1S891	PIG Small nuclear ribonucleoprotein-associated protein	SNRPB	-0.3	0.0	0.8	1.1	1.1	0.2	-0.6	-0.3	4	1
P27816-5	HUMAN Isoform 5 of Microtubule-associated protein 4	MAP4	1.4	0.0	-0.1	0.2	1.5	1.4	9.0	0.8	1	1
F2Z5J8	PIG Eukaryotic translation initiation factor 2 subunit 1	EIF2S1	0.5	0.0	0.4	0.7	-0.1	-0.8	-0.2	-0.4	11	4
I3LUP6	PIG Uncharacterized protein	LOC100525313	0.2	0.0	0.6	0.7	0.1	0.2	-1.7	0.0	14	5
Q85ZW4	PIG MHC class II antigen	SLA-DRA1	0.7	0.3	0.4	0.2	0.0	0.5	-0.6	-1.7	2	1
Q0VDD8	HUMAN Dynein heavy chain 14, axonemal	DNAH14	0.8	0.8	0.1	-0.1	0.2	0.1	-0.2	0.3	1	1
I3LFQ5	PIG Uncharacterized protein	PFKL	0.4	0.2	0.5	0.5	0.3	-0.2	0.1	0.0	10	9
Q9HC84	HUMAN Mucin-5B	MUC5B	0.0	0.1	0.5	0.9	-1.4	-1.6	-0.5	0.7	1	2
F1S2U6	PIG Uncharacterized protein (Fragment)	LPGAT1	1.0	0.6	-0.1	-0.1	1.2	1.0	0.2	-1.0	1	2
F1S9J8	PIG Uncharacterized protein	LOC100626723	-1.2	0.7	1.4	0.7	0.6	1.2	0.2	-2.7	1	1
B4DNX1	HUMAN cDNA FLJ53752, highly similar to Heat shock 70 kDa protein 1	NA	0.4	0.6	0.8	-0.3	0.4	1.0	0.7	-1.9	1	1
Q8TBY9	HUMAN WD repeat-containing protein 66	WDR66	0.3	0.3	0.6	0.3	0.8	1.1	0.6	0.4	1	1
A8K2X8	HUMAN cDNA FLJ78433, highly similar to Homo sapiens chaperonin containing TCP1, subunit 5 (epsilon) (CCT5)	NA	0.5	0.1	0.3	0.6	0.0	0.0	0.1	0.4	1	1
F1RU54	PIG Uncharacterized protein	MRPL11	1.0	0.9	-0.3	-0.1	1.7	1.8	0.6	-0.4	2	3
P35750	PIG Calpain-1 catalytic subunit	CAPN1	0.3	0.3	0.5	0.5	0.6	0.9	0.6	0.4	14	12
F1SMS8	PIG Uncharacterized protein	LMAN1	0.1	0.2	0.6	0.6	-0.1	-0.5	-0.5	-0.4	8	3
F1RJV5	PIG Uncharacterized protein	PDLIM4	0.5	0.0	0.3	0.7	2.2	0.8	0.3	0.6	3	1

F2Z5U4	PIG Ras-related protein Rab-1A	RAB1A	0.1	-0.4	0.6	1.2	-0.2	0.5	0.9	1.2	14	8
O00410	HUMAN Importin-5	IPO5	-0.4	-0.4	1.0	1.3	0.0	-0.9	-1.2	0.9	2	2
I3LEY2	PIG Annexin	ANXA7	-0.3	0.2	0.9	0.7	-0.4	-0.7	0.4	0.2	9	8
I3LEW5	PIG Uncharacterized protein	PSMD2	0.2	0.0	0.6	0.8	0.2	0.0	0.3	0.1	11	9
F1SRB7	PIG Uncharacterized protein (Fragment)	LOC100624537	-0.3	0.2	0.7	0.9	0.4	0.9	1.2	0.9	1	1
F2Z547	PIG cAMP-dependent protein kinase inhibitor alpha	PKIA	2.6	0.0	-0.9	-0.1	2.1	0.7	-1.7	-1.3	1	1
F1SUX4	PIG Uncharacterized protein (Fragment)	NUMA1	0.5	0.1	0.3	0.6	0.2	0.1	-0.5	0.8	15	8
I3LUR1	PIG Uncharacterized protein	HNRNPUL2	0.7	0.1	0.3	0.4	0.8	2.0	2.0	0.7	5	4
Q06AA4	PIG U1 small nuclear ribonucleoprotein A	SNRPA	0.2	-0.2	0.5	1.0	1.6	1.2	0.1	1.4	3	2
F1SHH7	PIG Uncharacterized protein	API5	0.5	0.3	0.5	0.2	-0.2	0.1	-0.4	0.6	3	2
I3LAB7	PIG Uncharacterized protein (Fragment)	CYP27A1	1.5	0.6	-0.1	-0.4	1.7	1.6	-1.4	-1.2	1	4
F1RGD9	PIG Uncharacterized protein	HARS	0.8	-0.2	0.2	0.7	0.5	0.2	-0.5	1.0	9	1
I3LEA6	PIG Uncharacterized protein	NA	-0.2	0.2	1.0	0.5	-1.0	-1.0	-0.5	0.3	2	1
I3L5M4	PIG Uncharacterized protein	CA14	1.3	0.8	-0.3	-0.3	1.7	0.9	-0.5	-0.6	2	1
Q13564	HUMAN NEDD8-activating enzyme E1 regulatory subunit	NAE1	0.5	0.7	0.7	-0.4	1.6	0.5	-0.1	-0.9	2	2
Q5TZA2	HUMAN Rootletin	CROCC	1.4	0.5	-0.3	-0.2	0.8	-0.8	-0.2	-2.1	1	2
F1RXA7	PIG Uncharacterized protein	LOC100512253	0.3	0.0	0.4	0.8	-0.3	0.2	0.1	0.2	13	9
F8SIP3	PIG Polyribonucleotide nucleotidyltransferase 1	PNPT1	0.3	0.6	0.6	0.1	0.2	0.3	-0.8	-0.6	2	3
I3L5H6	PIG Uncharacterized protein	NA	0.0	0.7	0.2	0.6	-0.5	-0.6	-0.1	0.6	3	3
G8ENL4	PIG FUS	FUS	-0.4	0.0	0.7	1.2	1.2	1.0	1.4	0.9	8	2
I3L813	PIG Proliferating cell nuclear antigen	PCNA	0.2	0.4	0.5	0.4	0.8	-0.3	-0.6	-1.3	3	1
Q9NPG4	HUMAN Protocadherin-12	PCDH12	0.8	0.7	0.1	-0.1	-1.0	-1.4	-0.3	0.5	1	1
F1RJY0	PIG Uncharacterized protein	NCSTN	1.0	0.5	0.1	-0.1	-0.5	-1.1	0.9	-1.2	1	1
F1RM44	PIG Uncharacterized protein (Fragment)	TOMM40	0.5	0.5	0.3	0.1	-0.4	-0.7	-0.9	-0.5	6	4
P62269	HUMAN 40S ribosomal protein S18	RPS18	-0.6	-0.2	0.9	1.4	-1.1	-1.0	-0.4	0.6	7	5
O14818	HUMAN Proteasome subunit alpha type-7	PSMA7	0.4	-0.3	0.6	0.8	-0.2	-0.7	0.8	-0.1	4	4
I3LVG4	PIG GrpE protein homolog	GRPEL1	0.0	0.8	0.1	0.5	2.0	1.3	-1.0	-1.7	4	1
F1SIW8	PIG Uncharacterized protein	NA	0.5	0.0	0.1	0.9	1.4	1.8	4.9	6.0	1	1
F1SSE7	PIG Uncharacterized protein (Fragment)	COL15A1	0.7	0.7	-0.2	0.3	1.0	0.5	-0.1	-1.3	3	3
Q69GF3	PIG NADH-ubiquinone oxidoreductase chain 3	ND3	0.6	0.7	0.5	-0.3	-0.3	1.2	2.3	2.8	1	1
I3LK71	PIG Uncharacterized protein	HNRNPC	-0.3	0.1	0.7	0.9	0.5	1.9	-0.1	-0.6	11	8
F1S8J6	PIG Uncharacterized protein	RAB2B	0.1	0.3	0.4	0.6	0.4	0.6	0.7	0.2	5	5
F1RJK0	PIG Uncharacterized protein	COQ5	1.4	0.3	0.0	-0.3	0.3	-0.6	-0.9	-0.9	6	5
I3LAJ6	PIG Uncharacterized protein	RALA	0.4	0.2	0.5	0.4	0.0	-0.2	0.9	1.2	2	3
I3LPA4	PIG Uncharacterized protein (Fragment)	KIF21B	0.2	0.3	0.7	0.2	-0.5	-0.1	0.4	0.6	1	1
Q19PY3	PIG tRNA-splicing ligase RtcB homolog	RTCB	0.7	-0.1	0.3	0.6	0.8	1.2	-0.1	-0.1	14	9
F1SRQ7	PIG Single-stranded DNA-binding protein	SSBP1	0.3	0.5	0.2	0.4	1.4	1.0	0.4	1.1	6	3
Q96FW1	HUMAN Ubiquitin thioesterase OTUB1	OTUB1	0.2	-0.2	0.5	0.9	-0.4	0.0	0.1	-0.3	5	2
H0YAA0	HUMAN Uncharacterized protein (Fragment)	NA	1.0	0.1	0.1	0.1	0.6	0.3	-1.1	-0.3	1	1
F1SME6	PIG Uncharacterized protein	NA	0.9	0.6	0.0	-0.2	0.6	0.1	-0.7	-1.4	3	3

P26042	PIG Moesin	MSN	-0.5	-0.2	0.7	1.4	-0.4	0.0	0.5	1.5	47	34
I3LJK4	PIG Uncharacterized protein	TMEM126A	0.9	0.6	0.1	-0.2	-0.7	-0.9	-1.0	0.7	1	2
F1SG92	PIG Uncharacterized protein	PARL	1.0	0.9	0.1	-0.6	1.3	2.2	-0.8	-1.4	2	1
Q15369	HUMAN Transcription elongation factor B polypeptide 1	TCEB1	0.2	0.3	0.7	0.2	1.2	-0.2	-0.9	-0.4	3	1
Q1T7A9	PIG Type VI collagen alpha-1 chain (Fragment)	COL6A1	0.3	0.2	0.1	0.7	0.2	-0.3	-0.7	-0.3	2	2
Q9NVE4	HUMAN Coiled-coil domain-containing protein 87	CCDC87	0.0	-0.1	0.4	1.1	-0.6	-0.2	0.4	-1.6	1	1
Q6QAQ3	PIG 60S ribosomal protein L23 (Fragment)	NA	0.2	0.4	0.4	0.4	-1.8	-1.8	-2.2	-0.9	1	1
I3LNF2	PIG Uncharacterized protein	DNAH1	0.5	0.6	0.2	0.1	1.0	8.3	0.3	-0.2	1	1
O94875-3	HUMAN Isoform 3 of Sorbin and SH3 domain-containing protein 2	SORBS2	0.2	0.4	0.7	0.0	-0.5	-0.2	-0.3	0.2	1	1
F1RT25	PIG Uncharacterized protein	SNX3	0.0	0.2	0.5	0.8	0.1	0.4	-0.1	0.6	5	2
B4DLU8	HUMAN cDNA FLJ59121, highly similar to Phosphatidylinositol 4-kinase alpha (EC 2.7.1.67)	NA	0.4	-0.2	0.4	0.9	-1.6	-1.3	-0.4	-1.1	1	1
Q5TB80	HUMAN Centrosomal protein of 162 kDa	KIAA1009	-0.1	0.4	0.5	0.6	-0.1	0.7	0.9	0.0	1	1
F1S1K0	PIG Uncharacterized protein	LOC100738633	-1.8	-0.4	1.6	2.0	-2.6	-5.4	-0.2	-2.8	2	2
F1S8N3	PIG Uncharacterized protein	RGS12	-0.5	0.0	1.2	0.7	-0.8	-0.6	-0.5	0.4	1	1
Q95319	PIG Beta-actin, cytoplasmic (Fragment)	NA	0.1	0.1	0.2	1.0	-0.8	0.5	0.3	-0.3	5	5
K7GT08	PIG Uncharacterized protein (Fragment)	LOC100525679	0.1	-0.4	0.4	1.3	0.2	0.0	-0.3	-1.4	1	1
I3LR32	PIG Uncharacterized protein	CCT5	-0.1	-0.3	0.7	1.1	-0.2	0.2	0.4	0.8	15	7
Q99988	HUMAN Growth/differentiation factor 15	GDF15	1.5	0.6	-0.4	-0.3	1.2	1.2	0.2	-0.4	1	1
I3LMJ1	PIG Uncharacterized protein	LOC100624587	-1.3	0.1	1.0	1.5	2.0	1.3	0.4	5.0	2	2
I3LL97	PIG Uncharacterized protein	CSRP1	-0.1	-0.1	0.6	1.0	-0.7	2.7	3.5	2.0	5	2
F1SS99	PIG Uncharacterized protein	PRUNE	0.3	0.7	0.4	0.0	-0.1	0.3	-0.2	-0.7	1	2
Q9MZ46	PIG Crocalbin-like protein (Fragment)	NA	-0.8	0.5	0.4	1.2	2.3	0.8	1.0	-0.6	10	5
A7TX80	PIG COP9 constitutive photomorphogenic-like subunit 5	COPS5	0.2	0.1	0.5	0.6	0.3	0.8	0.6	1.0	3	3
Q9BXP5	HUMAN Serrate RNA effector molecule homolog	SRRT	0.7	0.6	0.0	0.1	-0.5	0.0	-0.3	0.2	2	1
B6CVD7	PIG ERO1-like protein alpha	ERO1L	0.4	1.0	0.0	0.1	0.3	-0.5	-0.2	0.1	4	2
O02705	PIG Heat shock protein HSP 90-alpha	HSP90AA1	-0.5	-0.3	0.9	1.2	-1.1	-0.1	0.4	1.3	44	40
Q9GLV6	PIG Probable ATP-dependent RNA helicase DDX58	DDX58	0.0	0.1	0.5	0.7	0.6	-1.1	0.3	1.4	8	5
P62197	PIG 26S protease regulatory subunit 8	PSMC5	0.5	0.4	0.2	0.2	0.5	1.2	0.5	-0.1	11	10
F1SNQ1	PIG Uncharacterized protein (Fragment)	NA	-0.2	0.1	0.8	0.6	0.0	0.3	0.2	1.5	3	2
P61224	HUMAN Ras-related protein Rap-1b	RAP1B	0.3	0.1	0.3	0.6	-0.4	0.1	0.7	0.8	2	1
Q6IM70	PIG Reticulon	RTN4	0.2	-0.1	0.5	0.7	0.0	0.1	0.1	-0.1	2	2
Q29092	PIG Endoplasmin	HSP90B1	-0.5	0.1	0.7	1.0	-0.7	-0.4	-0.4	0.2	49	39
F1SSW9	PIG Uncharacterized protein (Fragment)	SPTBN5	0.2	0.6	0.4	0.2	-0.6	0.0	0.2	0.6	1	1
Q9Y3D0	HUMAN Mitotic spindle-associated MMXD complex subunit MIP18	FAM96B	0.3	0.7	0.2	0.1	0.3	0.5	-1.3	-1.6	1	1
I3LGD9	PIG Uncharacterized protein	NA	0.8	0.3	-0.1	0.2	0.7	0.2	-0.6	-0.6	6	4
I3LGI3	PIG Uncharacterized protein	FASTK	0.2	0.4	0.5	0.3	-1.7	-1.9	-2.5	-2.4	1	1
I3L6X5	PIG Uncharacterized protein	EFCAB4A	0.4	0.1	0.4	0.4	0.3	0.8	0.4	-0.2	2	2
P58107	HUMAN Epiplakin	EPPK1	0.8	0.3	0.0	0.2	1.8	1.2	1.3	0.9	2	1
P62081	HUMAN 40S ribosomal protein S7	RPS7	-0.2	-0.3	0.6	1.2	0.1	0.4	0.1	0.0	2	2
F1SHL3	PIG Uncharacterized protein	SEC23A	-0.5	-0.1	0.7	1.2	1.1	0.9	0.8	1.1	3	4

Q68DD2	HUMAN Cytosolic phospholipase A2 zeta	PLA2G4F	0.5	1.0	0.2	-0.4	0.0	1.0	1.0	0.2	1	1
F1RTQ6	PIG Histone H2B	HIST1H2BA	-1.1	0.6	0.7	1.2	-0.9	0.5	1.2	1.8	4	4
P28066	HUMAN Proteasome subunit alpha type-5	PSMA5	1.1	0.1	0.0	0.1	1.2	0.9	1.9	-0.4	7	3
F1SNX0	PIG Uncharacterized protein	LRRTM4	-1.3	-1.0	1.3	2.3	-0.5	-0.8	-0.7	0.4	1	1
F1ST81	PIG Uncharacterized protein	DBNL	0.3	0.1	0.3	0.6	-0.3	-0.2	0.4	-0.3	7	1
K9IVV5	PIG Tryptophan--tRNA ligase, cytoplasmic isoform a	WARS	0.2	0.2	0.4	0.5	0.6	0.0	-0.1	-1.1	5	3
F1S9J9	PIG Uncharacterized protein	RAB3GAP2	0.6	0.3	0.2	0.2	-0.9	0.4	1.1	2.0	1	2
Q06A98	PIG Serine/arginine-rich splicing factor 2	SRSF2	0.0	0.0	0.5	0.8	-1.0	-1.3	-1.2	-0.1	2	3
B0FWK4	PIG Signal peptidase complex subunit 1	SPCS1	-0.3	-0.1	0.7	1.0	0.3	-1.0	-0.6	-0.1	1	1
I3LSU1	PIG Uncharacterized protein (Fragment)	NONO	0.4	-0.1	0.5	0.5	0.2	0.2	-0.2	0.2	14	9
F1S9W8	PIG Uncharacterized protein	LOC100523621	0.3	0.3	0.2	0.5	0.4	-0.3	-0.2	0.4	4	1
Q9H8S5	HUMAN Cyclin N-terminal domain-containing protein 2	CNTD2	0.1	0.2	0.5	0.6	4.3	2.0	-2.0	-1.4	1	1
F1S1D0	PIG Uncharacterized protein (Fragment)	NA	0.3	0.5	0.4	0.0	-0.3	0.3	-0.9	-1.3	1	1
P02452	HUMAN Collagen alpha-1(I) chain	COL1A1	-0.8	0.3	0.6	1.1	0.4	1.8	-0.2	1.3	8	1
I3L829	PIG Uncharacterized protein (Fragment)	NPLOC4	1.1	0.4	-0.1	-0.1	0.8	0.7	0.5	-0.2	5	2
M3VH66	PIG Anoctamin	ANO10	0.3	-0.1	0.3	0.8	0.1	0.2	0.3	0.4	1	1
I3LAK4	PIG Pyruvate kinase	NA	0.7	0.3	0.1	0.2	0.8	0.6	1.7	0.7	1	1
F1S325	PIG Hydroxypyruvate isomerase	HYI	1.2	0.6	-0.1	-0.4	1.5	1.4	-0.3	-2.5	6	4
F1SLX0	PIG Uncharacterized protein (Fragment)	M6PR	-0.1	0.3	0.5	0.5	-0.1	0.0	1.2	-0.2	1	2
Q99719-2	HUMAN Isoform 2 of Septin-5	SEPT5	0.1	0.3	0.5	0.4	-0.6	-0.7	-0.6	-0.9	1	1
F1S0L8	PIG Uncharacterized protein	MTDH	0.1	0.1	0.3	0.8	0.9	0.5	1.4	1.1	6	4
Q06AT9	PIG RNA-binding protein 4B	RBM4B	0.7	0.1	0.2	0.3	-0.1	0.1	0.1	0.5	2	2
I3LJ48	PIG Uncharacterized protein (Fragment)	EHHADH	0.4	0.7	0.3	-0.2	0.0	0.2	-1.2	-1.5	1	1
F1RN27	PIG Uncharacterized protein	MPHOSPH8	0.4	0.4	0.4	0.1	0.0	-1.2	0.0	0.3	1	1
Q93052	HUMAN Lipoma-preferred partner	LPP	0.1	-0.2	0.4	1.0	0.6	0.9	1.0	1.0	3	2
D0G0C8	PIG Chaperonin containing TCP1, subunit 2 (Beta)	CCT2	0.4	-0.1	0.4	0.6	0.5	0.3	0.3	0.1	22	14
F1RW31	PIG Uncharacterized protein	NUDT9	-0.1	0.5	0.7	0.1	-0.6	1.4	-0.4	-0.5	4	3
I3LUG5	PIG Uncharacterized protein	TMEM143	0.7	0.2	0.2	0.1	-0.7	-1.5	-1.9	-1.3	2	3
K7GK90	PIG Uncharacterized protein	SSR4	0.6	-0.2	0.2	0.6	1.4	1.3	-0.5	-0.1	2	1
F1S788	PIG Uncharacterized protein (Fragment)	C8A	0.7	0.1	0.2	0.2	1.2	1.8	-0.2	-1.4	5	1
F1RZZ3	PIG Uncharacterized protein	FN3KRP	0.4	0.6	0.1	0.1	0.0	0.3	0.3	-0.2	1	1
F1S4M3	PIG Uncharacterized protein	PCDH7	1.0	0.2	0.2	-0.1	-0.7	0.5	-0.4	0.9	1	2
F1RGC9	PIG Uncharacterized protein	PSMD13	0.9	0.4	-0.1	0.0	0.4	0.2	-0.8	-0.5	6	3
I3LJ30	PIG Uncharacterized protein	NA	0.3	0.1	0.4	0.5	0.4	-0.1	-0.1	0.6	11	4
I3LS96	PIG Uncharacterized protein (Fragment)	NA	-0.2	-0.1	0.7	0.8	-0.5	-0.6	0.7	-0.9	2	1
Q8TDN2	HUMAN Potassium voltage-gated channel subfamily V member 2	KCNV2	2.0	0.0	-0.1	-0.7	-0.5	-0.7	0.5	-0.3	1	1
I3LGT3	PIG Uncharacterized protein (Fragment)	LAD1	0.8	0.4	0.1	-0.1	0.3	2.4	1.2	2.1	1	1
Q53DY5	PIG Histone H1.3-like protein	LOC595122	-0.8	0.4	0.5	1.1	-0.6	0.2	1.2	5.4	3	3
F1S1X9	PIG Uncharacterized protein	TXNL1	0.5	-0.2	0.3	0.6	1.0	1.3	0.7	-1.0	4	4
Q29223	PIG 60S ribosomal protein L34	RPL34	0.3	-0.4	0.4	1.0	1.2	0.0	0.4	0.7	3	2

F2Z5I4	PIG Histone H2A (Fragment)	NA	-0.2	0.2	0.3	0.9	-0.5	-0.2	0.3	1.8	2	3
Q29205	PIG 60S ribosomal protein L11	RPL11	-0.3	-0.5	0.8	1.3	-0.3	-0.3	-0.5	0.3	4	3
Q9P2Q2	HUMAN FERM domain-containing protein 4A	FRMD4A	0.7	0.7	0.1	-0.4	0.3	-0.3	-0.9	0.1	1	1
F1SBP8	PIG Uncharacterized protein	Ssc.21702	1.0	0.7	0.1	-0.5	3.6	1.8	-0.3	0.0	2	1
F1RHC5	PIG Histone H2A	H2AFY	0.0	0.3	0.3	0.6	-0.5	-0.2	-0.4	0.4	8	9
F1RL04	PIG Uncharacterized protein	PPM1F	0.0	0.3	0.5	0.4	0.7	0.4	-0.8	-1.2	3	1
O11780	PIG Transforming growth factor-beta-induced protein ig-h3	TGFBI	-0.2	-0.4	0.4	1.4	0.3	0.2	0.0	1.0	11	6
F1RWI5	PIG Uncharacterized protein	NA	-0.3	-0.7	0.9	1.3	-0.6	-0.5	-1.3	0.5	13	9
I3LDM3	PIG Uncharacterized protein (Fragment)	IPO7	0.5	0.1	0.3	0.4	0.3	0.8	-0.8	-0.7	9	6
Q9UMY4-2	HUMAN Isoform 2 of Sorting nexin-12	SNX12	0.3	-0.3	0.4	0.7	-0.9	-0.4	-0.8	-0.5	3	1
I3LDS3	PIG Uncharacterized protein	KRT10	0.0	1.1	0.0	0.1	0.0	-1.5	1.7	6.4	9	9
O77773	PIG Uncharacterized protein	CACNA2D1	0.7	0.6	0.0	-0.1	0.2	0.0	0.1	-1.0	7	6
F1SIA4	PIG Uncharacterized protein (Fragment)	ARHGAP1	0.1	-0.3	0.5	1.0	0.6	0.3	1.0	1.1	4	1
I3L893	PIG Uncharacterized protein	GDI1	0.2	0.1	0.4	0.5	0.6	0.9	0.2	-0.2	7	3
Q07960	HUMAN Rho GTPase-activating protein 1	ARHGAP1	-0.4	0.1	0.6	0.9	-0.6	-0.5	0.4	1.0	1	1
F1S6B4	PIG Uncharacterized protein	PRELP	0.2	0.5	-0.3	0.8	0.1	1.0	0.2	-0.5	15	9
K9IVQ2	PIG Ribosomal protein S6 kinase	RPS6KA5_tv1	1.2	0.6	-0.2	-0.4	2.1	-0.9	0.1	1.7	1	1
Q8N3K9	HUMAN Cardiomyopathy-associated protein 5	CMYA5	0.3	0.6	0.5	-0.3	-1.3	0.6	-0.4	0.5	1	2
A7WK50	PIG Insulin-regulated aminopeptidase	IRAP	0.1	-0.1	0.5	0.7	0.4	0.2	0.5	0.3	1	1
F1RG57	PIG Uncharacterized protein	C1orf198	0.3	0.1	0.5	0.3	1.4	-0.1	-0.6	0.5	1	1
F1RIP4	PIG Uncharacterized protein	RUVBL2	0.5	0.4	0.2	0.2	-0.5	-0.3	-0.3	0.4	9	2
P50402	HUMAN Emerin	EMD	-0.1	0.4	0.4	0.4	0.0	0.3	0.5	0.4	2	2
Q92817	HUMAN Envoplakin	EVPL	0.9	0.0	0.3	0.0	0.6	0.4	1.2	2.2	1	2
P62841	HUMAN 40S ribosomal protein S15	RPS15	-1.0	0.0	0.8	1.4	-1.1	-1.6	1.1	-0.2	3	1
P50238	HUMAN Cysteine-rich protein 1	CRIP1	0.2	-0.4	0.3	1.1	0.3	0.6	0.3	1.4	1	1
I3L2U4	HUMAN Peroxisomal acyl-coenzyme A oxidase 1	ACOX1	0.6	0.3	0.2	0.2	-0.3	0.3	-0.1	0.3	1	1
F1S0Q5	PIG Uncharacterized protein	SNX31	0.2	0.5	0.2	0.3	-0.2	-2.3	-2.6	-2.9	1	1
A8CLL2	HUMAN Beta myosin heavy chain cardiac isoform (Fragment)	MYH7	1.0	0.3	-0.1	0.0	0.0	-0.4	-1.4	-2.5	1	1
P54136	HUMAN Arginine--tRNA ligase, cytoplasmic	RARS	0.6	0.0	-0.1	0.7	0.4	-0.5	-0.3	-0.8	2	2
F1SGR0	PIG Eukaryotic translation initiation factor 3 subunit M	EIF3M	-0.1	0.1	0.5	0.7	-0.3	-0.4	0.2	0.6	3	1
F1RI39	PIG Uncharacterized protein	LOC100517284	0.1	-0.1	0.3	0.8	-0.3	-0.5	-0.3	-0.1	37	27
Q9NYQ7	HUMAN Cadherin EGF LAG seven-pass G-type receptor 3	CELSR3	1.2	0.3	-0.4	0.0	-0.2	0.3	0.1	0.5	1	1
P62829	HUMAN 60S ribosomal protein L23	RPL23	-0.3	-0.7	0.7	1.5	-1.1	-0.4	0.0	0.2	5	5
I3LIW1	PIG Uncharacterized protein (Fragment)	LANCL2	0.7	0.1	0.2	0.2	1.7	1.0	0.0	0.6	2	1
O00168	HUMAN Phospholemman	FXYD1	0.7	0.9	-0.2	-0.3	1.4	2.1	-0.1	1.8	1	1
Q95307	PIG 60S ribosomal protein L13a (Fragment)	RPL13A	0.1	-0.7	0.6	1.1	-0.4	0.0	0.8	0.7	7	2
Q9BRG1	HUMAN Vacuolar protein-sorting-associated protein 25	VPS25	0.2	-0.2	1.1	0.0	-0.1	-0.6	-1.5	-1.1	3	2
P08779	HUMAN Keratin, type I cytoskeletal 16	KRT16	0.9	0.5	0.2	-0.5	-1.5	-1.2	-1.9	5.8	1	3
F1SD96	PIG Uncharacterized protein (Fragment)	RAD23A	0.0	0.3	0.6	0.2	1.3	0.4	0.2	0.0	5	1
I3LQI3	PIG Uncharacterized protein (Fragment)	LOC100512195	0.5	0.0	0.2	0.5	-0.4	0.0	-0.4	0.7	2	2

F1S8H8	PIG Purine nucleoside phosphorylase (Fragment)	PNP	-0.1	0.8	0.6	-0.1	0.3	-0.8	-1.6	-1.4	5	1
F1SR53	PIG Uncharacterized protein	ITGA5	0.7	0.8	-0.2	-0.3	-0.6	-1.2	-1.0	-1.0	9	9
F1RJH8	PIG Uncharacterized protein	ASL	0.5	1.5	-0.1	-0.8	0.4	0.3	-0.1	0.1	2	2
B8Y4S5	PIG Sarcoglycan delta	NA	0.7	0.5	0.0	-0.2	0.4	0.5	0.2	0.2	3	6
F1S6R1	PIG Uncharacterized protein	CIRBP	0.9	0.9	-0.2	-0.4	0.7	1.9	0.5	-0.2	1	2
F1S0Z4	PIG Histone H2A (Fragment)	H2AFZ	-1.2	0.5	0.9	1.0	-1.3	-0.8	-0.1	0.7	2	2
P40125	PIG Adenylyl cyclase-associated protein 1 (Fragments)	CAP1	-1.4	-0.2	1.0	1.8	-1.8	-0.3	-0.6	0.9	1	1
P28335-2	HUMAN Isoform 2 of 5-hydroxytryptamine receptor 2C	HTR2C	0.9	0.6	-0.1	-0.3	-0.3	0.8	-0.8	-1.8	1	1
I3LGQ4	PIG Small nuclear ribonucleoprotein Sm D2 (Fragment)	SNRPD2	0.3	0.2	0.3	0.3	0.6	0.0	-0.4	0.2	3	3
Q19QT0	PIG Splicing factor, arginine/serine-rich 11	SFRS11	1.0	0.0	0.1	0.1	1.6	1.4	-0.2	-1.2	1	1
F1SR88	PIG Uncharacterized protein	FAM134A	0.8	0.6	0.0	-0.3	2.3	1.5	-1.4	0.0	2	2
P61163	HUMAN Alpha-centractin	ACTR1A	0.5	-0.1	0.3	0.4	1.2	1.7	1.3	0.5	4	3
Q9Y2D5-4	HUMAN Isoform 2 of A-kinase anchor protein 2	AKAP2	0.9	0.3	-0.1	-0.1	1.2	2.2	0.7	-0.9	1	1
I3LMV8	PIG Uncharacterized protein	ABCB8	1.7	0.4	-0.1	-1.0	0.6	0.4	0.5	-0.2	3	7
Q9HAT2	HUMAN Sialate O-acetylesterase	SIAE	0.2	-0.4	0.7	0.6	1.0	0.8	0.6	-1.4	1	1
G3DRF8	PIG Proteasome subunit beta type	PSMB6	0.6	-0.2	0.2	0.5	0.8	0.5	-0.2	-1.6	4	4
F1SR80	PIG Tubulin alpha-1A chain	TUBA1A	-0.3	-0.4	0.8	1.0	-0.2	-1.4	-1.2	-0.3	2	2
F1RTN3	PIG Moesin	MSN	-0.3	-0.4	0.6	1.2	0.0	0.7	0.6	1.2	3	1
F1RFB1	PIG Uncharacterized protein	PKD1	0.0	0.1	0.7	0.3	1.1	1.8	1.9	0.8	3	2
F1RQM2	PIG Phosphoacetylglucosamine mutase	PGM3	0.8	0.0	0.1	0.1	-0.5	-0.8	0.3	0.0	4	2
K7EM20	HUMAN 14-3-3 protein epsilon (Fragment)	YWHAE	0.6	0.2	0.4	0.0	2.4	0.0	-0.1	-1.3	1	2
M3V862	PIG Nuclear transcription factor Y, alpha tv1	CBFB	0.1	-0.3	0.5	0.8	0.1	0.2	-0.2	-0.3	3	1
F1S408	PIG Uncharacterized protein	FAM98A	0.4	0.3	0.3	0.1	-0.4	0.2	0.6	0.6	5	1
P63218	HUMAN Guanine nucleotide-binding protein G(I)/G(S)/G(O) subunit gamma-5	GNG5	0.2	0.0	0.3	0.5	-0.2	0.1	0.3	1.0	1	1
Q96GF1	HUMAN E3 ubiquitin-protein ligase RNF185	RNF185	-0.4	-0.1	0.4	1.1	0.2	2.3	1.6	0.8	1	1
B4DGH2	HUMAN cDNA FLJ56781	NA	0.1	0.2	0.2	0.6	0.4	0.4	0.2	0.1	1	1
F1SSG5	PIG Uncharacterized protein	NANS	0.5	0.1	0.1	0.4	-0.5	0.9	-1.2	-0.1	2	1
I3LUM8	PIG Uncharacterized protein	LOC100626089	0.3	0.3	0.1	0.4	0.0	-0.1	-0.4	-0.4	3	3
F1S1X3	PIG Uncharacterized protein	NARS	0.3	0.1	0.2	0.4	0.2	0.2	-0.8	-0.8	7	3
I3LNT6	PIG Uncharacterized protein	KRT77	0.7	0.7	-0.2	-0.2	0.4	-1.1	3.4	6.3	1	1
F1SFM3	PIG Uncharacterized protein (Fragment)	CHL1	1.2	-0.1	0.1	-0.1	0.5	-0.2	-1.1	-1.2	1	1
P25398	HUMAN 40S ribosomal protein S12	RPS12	0.0	-0.2	0.5	0.7	0.1	0.3	-0.3	0.0	2	2
I3LPU8	PIG Uncharacterized protein	NA	0.0	0.1	0.4	0.5	0.0	0.7	0.6	-0.2	6	2
F1S8W1	PIG Uncharacterized protein	COX15	0.6	0.1	0.2	0.2	-0.8	-0.7	-0.2	-0.9	2	3
K7GPS6	PIG Uncharacterized protein	ARMCX3	0.5	0.2	0.2	0.1	-0.2	0.0	-1.2	-0.2	4	2
Q8SPB2	PIG MHC class II antigen	SLA-DRB	0.5	-0.3	0.5	0.4	0.3	-1.3	-0.9	-2.0	1	1
Q56VC2	PIG Fas (TNF receptor superfamily, member 6)	TNFRSF6	0.0	0.1	0.5	0.4	-0.3	-0.3	-0.5	0.0	1	1
F1SEF7	PIG Serine/threonine-protein phosphatase (Fragment)	PPP1CB	-0.3	-0.4	0.5	1.2	0.0	-0.4	-0.2	-0.9	1	1
Q8MJ37	PIG Ran (Fragment)	NA	-0.7	-0.5	0.9	1.4	-0.9	0.1	0.7	0.6	3	3
F1SB09	PIG Uncharacterized protein	SEC22B	0.4	-0.1	0.1	0.7	-0.4	-0.2	-0.6	0.5	8	4

F1RZ96	PIG Uncharacterized protein	TARSL2	-0.8	0.5	1.1	0.2	-0.9	-0.9	-0.1	1.2	2	3
F1SEN6	PIG Uncharacterized protein	MMRN2	1.4	0.3	-0.4	-0.3	0.8	1.6	-0.4	-0.6	6	1
Q29599	PIG 2'-5'-oligoadenylate synthase 1	OAS1	1.1	0.5	-0.3	-0.2	1.9	0.0	0.4	-1.8	3	1
Q9GLW8	PIG Peroxiredoxin 5	PRDX5	-0.1	0.1	0.4	0.6	-0.1	1.1	-1.9	0.0	6	1
P62249	HUMAN 40S ribosomal protein S16	RPS16	0.0	-0.5	0.5	1.0	-0.3	-0.7	-0.2	-0.2	2	2
P02461	HUMAN Collagen alpha-1(III) chain	COL3A1	-1.3	1.0	0.3	1.0	0.4	1.7	0.5	1.3	2	2
O97788	PIG Fatty acid-binding protein, adipocyte	FABP4	0.2	0.0	-0.2	1.0	-0.6	-0.5	-0.4	-0.1	8	7
I3L7L5	PIG Uncharacterized protein (Fragment)	NA	-0.6	0.2	0.5	0.9	-0.2	0.6	-0.8	-1.0	2	1
F1RP44	PIG Uncharacterized protein (Fragment)	IPO5	-0.3	-0.2	0.7	0.7	-0.2	-0.6	-0.1	-0.2	9	10
K7GSF4	PIG Uncharacterized protein	FMR1	1.2	-1.2	0.6	0.4	1.1	14.5	1.8	0.2	2	1
I3LP11	PIG Uncharacterized protein	KHDRBS1	0.1	-0.2	0.3	0.8	0.9	0.8	0.6	-1.7	2	1
F1RQU9	PIG Uncharacterized protein	LOC100738725	0.3	-0.1	0.0	0.9	0.0	0.7	0.2	1.8	1	1
F1SFA2	PIG Uncharacterized protein	LOC100620687	1.4	0.2	-0.2	-0.5	0.2	-0.5	-0.9	-0.5	1	1
I3LKZ4	PIG Uncharacterized protein	HNRNPR	-0.1	0.1	0.5	0.5	-0.1	-0.4	-0.4	-1.5	10	10
F1RYY0	PIG Uncharacterized protein	FKBP5	-0.1	0.0	0.4	0.7	0.8	1.2	-0.5	-0.3	3	1
F1RKI9	PIG Uncharacterized protein	PSMD8	-0.1	0.3	0.5	0.3	-0.6	-0.7	-0.1	0.4	4	2
A0PA01	PIG Serine protease inhibitor 9	PI-9	0.9	0.5	-0.1	-0.3	2.0	2.8	1.4	-0.8	4	4
Q9XT00	PIG Estradiol 17-beta-dehydrogenase 8	HSD17B8	1.4	0.5	0.0	-0.8	0.6	-0.3	0.2	-0.8	2	3
F1SUT0	PIG Uncharacterized protein	RAB6C	0.0	0.1	0.4	0.5	0.0	0.4	0.9	1.0	5	4
F1SUK2	PIG Uncharacterized protein	PAK1	0.2	0.0	0.4	0.4	1.0	1.5	-0.1	-0.7	1	1
K9IVR9	PIG Importin subunit beta-1	KPNB1	0.2	0.1	0.3	0.5	0.4	0.5	-0.7	-0.4	17	12
F1RPL2	PIG Histone H3	LOC100156557	-1.4	0.6	0.4	1.4	-2.7	-1.4	0.2	0.5	7	7
F1S1J9	PIG Eukaryotic translation initiation factor 3 subunit H	EIF3H	0.1	-0.2	0.4	0.7	-0.6	-0.3	-0.5	0.2	3	5
I3LRP7	PIG Uncharacterized protein	NA	0.5	0.3	0.1	0.0	0.4	0.9	-0.2	-0.9	1	2
Q6QGC0	PIG PDZ and LIM domain protein 3	PDLIM3	0.5	0.3	0.2	0.0	0.1	0.4	0.0	-1.6	6	2
Q8MJ30	PIG Dihydropteridine reductase	QDPR	0.9	0.2	0.0	-0.2	0.4	0.3	-0.6	-0.2	8	8
I3LMM7	PIG Uncharacterized protein	NA	0.2	0.0	0.1	0.6	0.1	0.4	-0.6	0.0	3	3
F1RI01	PIG 60S ribosomal protein L13a	RPL13A	0.0	-0.6	0.5	1.0	-0.4	-0.4	0.1	-0.1	4	3
Q29048	PIG V-type proton ATPase catalytic subunit A	ATP6V1A	0.0	0.1	0.3	0.5	-0.4	-0.3	0.0	0.5	9	4
I3LS60	PIG Uncharacterized protein	CARKD	0.8	0.5	-0.1	-0.3	-0.5	-0.9	-0.5	-1.1	5	4
I3LIL7	PIG Uncharacterized protein	LOC100620394	0.0	0.1	0.1	0.7	-0.6	-1.5	-1.5	-1.7	6	4
I3L9G3	PIG Uncharacterized protein	CHRNB1	0.8	0.5	-0.1	-0.2	0.1	-0.4	4.5	1.9	1	1
F1RRP6	PIG Annexin (Fragment)	ANXA13	-0.2	-0.1	0.5	0.8	-0.3	-0.6	0.0	1.2	1	1
F1RGY5	PIG Uncharacterized protein	LOC100739233	0.1	0.0	0.3	0.5	1.2	0.5	0.5	0.8	4	2
Q9Y623	HUMAN Myosin-4	MYH4	0.8	0.8	-0.2	-0.4	-2.0	1.3	-1.1	-0.1	4	4
F1S4X9	PIG Uncharacterized protein	GSS	0.5	-0.3	0.2	0.6	-0.4	-0.5	0.9	0.5	2	1
O00442	HUMAN RNA 3'-terminal phosphate cyclase	RTCA	-0.1	0.5	-0.1	0.6	0.5	0.6	-0.9	-1.1	2	2
F1SDK3	PIG Uncharacterized protein	MGMT	0.6	0.5	0.0	-0.1	-0.1	0.3	-1.8	-0.9	3	1
M3VH35	PIG Lamin B receptor	LBR	-0.2	0.3	0.4	0.4	0.1	0.9	0.4	0.1	3	1
F1SK49	PIG Uncharacterized protein	PLGRKT	0.8	0.5	-0.1	-0.2	0.2	0.2	-0.6	0.2	5	5

I3LHR4	PIG Uncharacterized protein	LOC100739651	1.0	0.3	-0.2	-0.2	1.3	0.4	-0.4	-2.6	1	1
I3LSS2	PIG Uncharacterized protein (Fragment)	CHMP2A	0.0	0.1	0.2	0.6	1.8	0.3	-0.5	0.7	1	1
K9J6J2	PIG Erythrocyte band 7 integral membrane protein isoform a	STOM_tv1	0.4	0.3	0.1	0.1	0.7	-0.4	-0.8	-0.3	4	2
P00519	HUMAN Tyrosine-protein kinase ABL1	ABL1	0.3	-0.4	0.2	0.8	0.4	0.8	0.4	0.9	1	1
F1RZ28	PIG Uncharacterized protein	RPS10	-1.0	0.0	0.7	1.2	0.4	0.8	1.3	1.4	5	7
Q5T7N0	HUMAN 60S ribosomal protein L5 (Fragment)	RPL5	-1.0	-0.3	0.7	1.5	-0.4	0.4	0.0	1.2	1	1
F1SL58	PIG Uncharacterized protein (Fragment)	LOC100519091	0.3	0.2	0.2	0.3	2.1	2.1	0.6	-1.1	10	6
F1SRM1	PIG Uncharacterized protein (Fragment)	MYO10	0.6	0.4	0.2	-0.3	-0.9	0.1	-1.8	-0.5	2	2
F1RWP3	PIG Uncharacterized protein	CYGB	0.2	0.1	0.0	0.6	0.1	1.0	0.8	0.6	6	1
K7GQ07	PIG Neuropilin-1 isoform a	NRP1	0.1	0.0	0.1	0.7	-0.6	1.5	0.0	1.0	2	1
I3LI96	PIG Uncharacterized protein	NA	1.4	0.0	0.0	-0.4	0.1	-0.3	-1.1	0.2	1	4
F1ST77	PIG Uncharacterized protein	ADCY1	0.8	0.2	-0.1	0.0	0.5	-0.2	0.7	0.0	1	2
F1RP17	PIG T-complex protein 1 subunit gamma	CCT3	0.0	-0.1	0.4	0.6	0.4	0.9	0.5	0.5	12	15
P15982	PIG SLA class II histocompatibility antigen, DQ haplotype C beta chain	NA	0.3	0.1	0.2	0.3	-0.6	-0.2	-2.0	-0.4	3	2
I3L816	PIG Uncharacterized protein	HNRNPH1	0.2	0.3	0.1	0.3	0.8	1.8	0.0	-0.8	2	2
F1S1B0	PIG Uncharacterized protein	TOMM40L	-0.4	0.1	1.0	0.3	-1.1	-0.8	0.7	-0.1	1	1
F1S399	PIG Uncharacterized protein (Fragment)	FAF2	0.6	0.5	0.4	-0.5	0.5	0.7	0.1	-0.1	1	2
Q86V81	HUMAN THO complex subunit 4	ALYREF	1.0	0.0	-0.3	0.2	0.7	0.4	0.0	0.0	4	4
I3LNQ4	PIG Uncharacterized protein	MAP3K2	1.0	0.7	-0.3	-0.5	-0.8	-1.2	-1.5	-1.0	1	1
P79324	PIG 60S ribosomal protein L15 (Fragment)	RPL15	-0.7	-0.5	0.7	1.4	-1.5	-1.8	-0.2	-0.4	6	4
Q9NQC3	HUMAN Reticulon-4	RTN4	0.6	0.1	0.2	0.1	0.6	0.4	-0.9	-1.2	1	1
Q7M2W6	PIG Alpha-crystallin B chain	CRYAB	-0.8	0.1	0.8	0.7	-0.5	0.5	1.2	0.4	13	13
F2Z5R2	PIG Uncharacterized protein (Fragment)	RHEB	0.6	0.3	-0.1	0.1	0.7	0.0	-1.1	-1.0	1	1
P08133	HUMAN Annexin A6	ANXA6	0.8	0.5	0.2	-0.6	0.6	0.8	-0.7	-1.3	1	1
Q14204	HUMAN Cytoplasmic dynein 1 heavy chain 1	DYNC1H1	0.3	0.0	0.1	0.5	-0.4	-0.1	-0.5	0.1	44	45
F1RX16	PIG Uncharacterized protein	DDX3X	-0.2	-0.1	0.5	0.7	0.3	1.2	-0.1	-0.2	11	8
F1STP0	PIG Uncharacterized protein	FAT3	0.5	0.7	-0.2	-0.1	0.2	0.0	0.0	-0.1	1	1
P12309	PIG Glutaredoxin-1	GLRX	-0.7	0.2	0.5	0.9	1.3	2.1	0.6	0.7	4	3
I3L6B9	PIG Uncharacterized protein	PSMD7	-0.3	0.2	0.6	0.5	-1.2	-1.0	-0.5	-0.4	1	1
Q29228	PIG 4-trimethylaminobutyraldehyde dehydrogenase (Fragment)	ALDH9A1	0.2	-0.3	0.5	0.5	-0.2	-0.5	0.7	0.0	2	2
F1RYZ0	PIG 60S acidic ribosomal protein P2	RPLP2	0.1	0.2	0.2	0.3	2.1	1.6	0.0	-1.7	4	2
Q92901	HUMAN 60S ribosomal protein L3-like	RPL3L	-0.3	-0.1	0.6	0.6	0.0	0.4	0.2	0.0	2	2
A5A759	PIG Keratin 2A	KRT2A	1.0	-0.1	0.7	-0.7	0.3	-2.1	2.2	-0.2	2	3
F1RKW8	PIG Uncharacterized protein (Fragment)	PSMD11	0.2	-0.1	0.3	0.5	0.3	0.3	-0.1	-0.9	10	6
Q12769-3	HUMAN Isoform 3 of Nuclear pore complex protein Nup160	NUP160	0.0	-0.4	0.6	0.6	-1.2	-0.2	-0.1	-0.3	1	1
F1RU38	PIG Uncharacterized protein	SF3B2	-0.2	-0.1	0.4	0.7	0.7	-0.2	-1.1	0.3	2	1
B7TJ03	PIG Ribosomal protein L26-like 1	RPL26L1	-0.6	-0.5	0.7	1.2	-1.1	-0.5	0.0	0.5	5	5
P50578	PIG Alcohol dehydrogenase [NADP(+)]	AKR1A1	-0.4	-0.2	0.6	0.8	-0.4	0.1	0.4	-0.2	9	7
Q8TC05	HUMAN Nuclear protein MDM1	MDM1	1.3	0.1	-0.2	-0.3	1.6	1.2	0.7	0.9	1	1
F1S8X9	PIG Uncharacterized protein	HOGA1	1.1	0.3	0.1	-0.6	-0.2	-0.5	-1.1	-1.6	3	4

I3LQI9	PIG Uncharacterized protein (Fragment)	RAP1GDS1	0.3	0.5	0.1	0.0	1.0	1.5	1.2	0.7	1	1
Q8WNY6	PIG Cytoplasmic light-chain dynein	NA	-0.8	-0.5	0.7	1.4	-0.2	0.2	0.3	1.4	1	1
F1SFL1	PIG Uncharacterized protein	ARL8B	0.2	-0.2	0.2	0.7	0.0	0.3	-0.4	-0.1	2	2
F1STR6	PIG Uncharacterized protein	NUDC	-0.5	-0.3	0.7	1.0	-0.3	-0.2	0.3	0.3	13	4
A2TLM1	PIG Ribose-5-phosphate isomerase	RPIA	0.8	0.5	-0.1	-0.4	0.3	0.2	-0.2	-0.7	4	3
P0C671	HUMAN Uncharacterized protein C6orf222	C6orf222	0.2	0.0	0.3	0.3	0.3	-0.3	1.2	-1.3	1	1
P31689	HUMAN DnaJ homolog subfamily A member 1	DNAJA1	0.1	0.1	0.2	0.5	0.5	0.2	-0.8	-0.1	3	1
Q52NJ1	PIG Ras-related protein Rab-11A	RAB11A	-0.1	0.3	0.3	0.3	-0.5	0.0	0.0	0.5	9	8
H7C2V0	HUMAN Fatty acid desaturase 1 (Fragment)	FADS1	0.8	0.7	0.0	-0.6	0.1	-0.3	-1.5	-0.7	2	1
Q99963	HUMAN Endophilin-A3	SH3GL3	0.8	0.7	-0.2	-0.5	0.3	0.4	0.4	0.5	1	1
F1RL48	PIG Uncharacterized protein	ACAD10	0.8	0.7	0.2	-0.9	0.0	-0.3	-1.4	-1.8	1	1
F1SKQ0	PIG Uncharacterized protein (Fragment)	DDX17	0.2	-0.4	0.4	0.6	0.5	0.7	-0.1	-0.1	19	11
Q8MHT7	PIG MHC class I antigen	SLA-1	0.6	-0.2	0.1	0.3	0.4	-0.4	-1.4	-1.7	1	1
F1SUE3	PIG Uncharacterized protein	PPA1	-0.2	-0.1	0.6	0.6	0.7	0.2	-0.4	-1.9	6	1
F1RZQ9	PIG Uncharacterized protein	MNF1	0.6	0.5	0.0	-0.3	0.0	0.7	0.4	0.4	2	2
F1SQS2	PIG Uncharacterized protein	TMPO	0.9	-0.1	0.0	0.1	0.0	0.4	1.0	0.7	2	1
F1RFB4	PIG Uncharacterized protein (Fragment)	SLC9A3R2	1.0	-0.3	0.1	0.1	0.8	0.1	0.0	-0.9	3	2
I3LU76	PIG Uncharacterized protein	CAND2	0.5	0.0	0.1	0.3	0.8	0.4	-0.6	-0.4	5	5
D2SW95	PIG Coatomer subunit beta	COPB1	-0.3	-0.4	0.6	1.0	-0.2	0.3	0.2	0.8	13	4
F1S431	PIG Uncharacterized protein	AARS	0.3	-0.2	0.1	0.6	2.9	0.3	-0.1	0.6	9	1
P60900	HUMAN Proteasome subunit alpha type-6	PSMA6	0.4	0.2	0.2	0.1	1.0	1.0	1.0	-0.8	2	1
F2Z5B4	PIG Uncharacterized protein	HNRNPA3	-0.5	-0.3	0.5	1.0	-0.2	1.0	1.3	0.0	14	10
I3LFL4	PIG 60S ribosomal protein L18 (Fragment)	RPL18	-0.1	-0.5	0.3	1.1	-0.3	0.2	-0.3	1.6	1	2
Q9P278	HUMAN Folliculin-interacting protein 2	FNIP2	2.6	0.2	-0.8	-1.2	-0.7	-0.4	-0.1	0.7	1	1
I3LV17	PIG Uncharacterized protein (Fragment)	RALB	0.5	0.2	0.0	0.1	0.4	0.5	-0.6	-0.5	4	2
Q6QAP7	PIG 40S ribosomal protein S17	RPS17	-0.4	-0.5	0.7	1.1	0.4	0.8	0.0	-0.4	6	3
P79403	PIG Neutral alpha-glucosidase AB	GANAB	-0.1	0.0	0.3	0.7	-0.3	-0.3	-0.1	-0.3	25	18
Q863Z0	PIG Proteasome activator complex subunit 2	PSME2	-0.2	-0.2	0.4	0.8	2.2	2.2	0.6	0.1	7	2
F1S1L1	PIG Uncharacterized protein	TIMM8A	0.7	0.9	-0.4	-0.4	3.5	1.0	0.2	-2.6	2	1
F1SBC8	PIG Uncharacterized protein	EMILIN2	-0.2	0.4	0.4	0.1	0.7	0.7	0.4	0.6	2	2
F1S8L9	PIG Uncharacterized protein	HNRNPU	-0.3	-0.2	0.4	0.9	0.4	0.8	0.4	0.0	20	10
F1SAG9	PIG Uncharacterized protein	MCAM	-0.7	0.0	0.5	1.0	0.1	0.7	0.5	-0.3	1	1
P02554	PIG Tubulin beta chain	NA	-0.5	-0.4	0.5	1.2	0.6	0.7	-0.4	-1.0	24	20
F1S9D6	PIG Myosin-7	MYH7	2.4	0.4	-1.0	-1.0	1.6	1.0	-0.7	-0.2	2	2
I3LRH2	PIG Uncharacterized protein	LOC100519984	-0.4	0.2	0.6	0.4	1.5	0.8	-0.7	-1.6	19	9
F1S710	PIG Uncharacterized protein	CACYBP	-0.4	-0.1	0.5	0.8	0.7	0.8	-0.7	-0.4	6	1
F1SNL1	PIG Cerebellin 2	CBLN2	0.9	0.7	-0.3	-0.5	-0.1	1.1	0.6	-0.7	1	1
I3LCA2	PIG Uncharacterized protein	CCT8	0.0	-0.3	0.4	0.7	0.0	0.1	0.2	0.7	26	18
I3LII3	PIG Uncharacterized protein	EEF2	-0.8	-0.5	0.9	1.3	-1.2	-0.8	-0.2	0.3	29	23
P79381	PIG Epoxide hydrolase 1	EPHX1	0.7	0.4	0.1	-0.4	0.2	-0.1	1.1	0.2	11	14

F1SFA7	PIG Uncharacterized protein	COL1A2	-0.8	0.5	0.0	1.1	1.9	4.3	1.3	1.8	11	8
F1RS45	PIG DNA topoisomerase 2	NA	0.0	-0.2	0.5	0.5	0.6	2.9	0.2	-0.5	4	1
F1SLZ3	PIG Uncharacterized protein (Fragment)	LOC100520244	1.1	1.2	-0.5	-1.0	2.5	0.8	0.3	0.3	3	1
F1SH76	PIG Uncharacterized protein	HMGA2	0.0	0.0	0.3	0.5	1.1	1.6	1.6	1.2	1	2
Q29593	PIG Transaldolase (Fragment)	TALDO1	0.2	-0.2	0.2	0.7	0.6	0.0	-0.8	-0.3	1	1
F1S9S9	PIG Uncharacterized protein	FUBP1	0.3	-0.4	0.3	0.6	1.6	1.2	0.5	0.4	8	3
Q6PGN9	HUMAN Proline/serine-rich coiled-coil protein 1	PSRC1	0.7	0.0	0.1	0.0	-1.7	-1.6	-1.2	-0.1	1	1
Q27HK4	PIG Metaxin-1	MTX1	0.8	0.5	-0.3	-0.2	0.8	1.1	-0.3	-0.1	2	4
I3LSY1	PIG Uncharacterized protein	MRPL12	0.4	0.3	-0.1	0.2	1.4	2.4	2.3	1.5	1	1
Q6S4N2	PIG Heat shock 70 kDa protein 1B	HSPA1B	0.2	0.3	0.1	0.1	0.5	0.9	0.4	-0.4	8	6
Q2YHQ3	PIG Filamin-A (Fragment)	FLNA	-0.8	-0.1	0.6	1.2	-0.9	-0.7	1.6	1.4	9	3
F1RUH7	PIG Uncharacterized protein	TPMT	0.9	-0.1	0.3	-0.3	-0.1	-0.6	-0.9	0.4	1	2
Q96QR8	HUMAN Transcriptional activator protein Pur-beta	PURB	0.9	-0.1	-0.2	0.1	1.7	2.2	3.8	1.7	1	1
Q06190	HUMAN Serine/threonine-protein phosphatase 2A regulatory subunit B'' subunit alpha	PPP2R3A	0.3	0.2	0.4	-0.2	1.7	2.8	6.7	3.3	2	1
F1RNN0	PIG Uncharacterized protein	VPS29	0.2	-0.5	0.3	0.7	-0.5	-0.7	0.0	0.9	5	2
O43684-2	HUMAN Isoform 2 of Mitotic checkpoint protein BUB3	BUB3	0.0	0.0	0.1	0.6	0.7	-0.8	-0.9	-0.6	2	1
B6VAQ2	PIG Proteasome subunit alpha type 6	PSMA6	0.3	-0.3	0.3	0.4	0.9	0.6	1.8	-1.1	6	5
I3LDQ9	PIG Uncharacterized protein	LOC100737818	0.4	0.5	0.2	-0.4	-0.6	-0.8	-0.5	0.9	3	4
F1SLC2	PIG Uncharacterized protein (Fragment)	TOMM22	0.9	0.3	-0.2	-0.3	0.9	0.0	0.0	-0.2	4	4
Q9NP79	HUMAN Vacuolar protein sorting-associated protein VTA1 homolog	VTA1	-0.1	0.1	0.3	0.4	-0.1	-0.5	-0.2	-0.1	1	1
P49666	PIG 60S ribosomal protein L21 (Fragment)	RPL21	-0.7	-0.6	0.7	1.3	-0.3	0.7	1.7	0.3	3	3
P46778	HUMAN 60S ribosomal protein L21	RPL21	-0.1	-0.3	0.3	0.9	0.0	0.7	0.2	-0.8	1	1
F2Z5A8	PIG Structural maintenance of chromosomes protein	SMC1A	0.2	-0.5	0.7	0.3	0.5	-0.3	0.2	0.7	5	3
I3LQ16	PIG Uncharacterized protein	NA	-0.8	0.0	0.6	0.8	-2.0	-1.5	-0.7	0.7	1	1
O60437	HUMAN Periplakin	PPL	0.2	1.2	0.3	-1.0	0.0	0.1	1.7	1.3	2	2
F1S5H7	PIG Uncharacterized protein	NHLRC2	0.8	0.3	0.0	-0.4	0.4	0.3	-0.4	-0.7	5	11
I3L6L6	PIG Uncharacterized protein	AKNA	1.9	-1.1	-0.3	0.3	-0.5	0.0	-0.1	0.9	1	1
F1RXG2	PIG Uncharacterized protein	KRT28	0.1	0.8	-0.2	0.1	0.6	-1.3	1.6	9.3	2	3
I3LDC7	PIG Isocitrate dehydrogenase [NADP] (Fragment)	IDH1	0.0	0.3	0.1	0.3	0.6	1.7	0.7	0.0	11	9
D5K895	PIG Spermatogenesis associated serine-rich 2-like protein	SPATS2L	-0.1	0.6	0.1	0.1	1.0	0.3	-0.4	0.8	2	2
Q9C007	HUMAN Voltage-gated sodium channel alpha subunit splice variant SCN3A-s	SCN3A	1.0	1.3	-0.6	-1.0	1.5	4.6	2.1	-0.3	1	1
Q75N90	HUMAN Fibrillin-3	FBN3	-0.3	-0.3	0.4	0.9	0.9	-0.3	0.7	0.4	1	1
Q04967	PIG Heat shock 70 kDa protein 6	HSPA6	0.0	-0.4	0.3	0.8	0.0	-0.3	-0.4	-1.8	2	4
B8XSJ2	PIG ER lipid raft-associated 2 isoform 2	ERLIN2	0.5	0.2	0.0	0.0	0.1	0.3	-0.7	0.1	5	4
I3LFQ9	PIG Uncharacterized protein	LOC100522249	0.0	0.0	0.6	0.1	-1.5	-0.8	-0.2	-0.4	1	1
Q16186	HUMAN Proteasomal ubiquitin receptor ADRM1	ADRM1	-0.3	-0.4	0.5	0.8	0.1	0.3	-0.2	-0.1	1	1
F1SPK1	PIG Uncharacterized protein (Fragment)	PLXND1	0.4	0.6	-0.3	-0.1	0.1	0.7	-0.1	1.3	1	1
H0YI37	HUMAN ATP synthase subunit beta, mitochondrial (Fragment)	ATP5B	0.5	0.1	0.2	-0.1	0.3	1.0	0.6	-0.5	1	1
I3LB68	PIG Uncharacterized protein	DAZAP1	0.6	-0.2	0.2	0.0	-1.1	-1.5	-2.0	-1.4	2	2
Q19PY1	PIG Phosphorylase (Fragment)	PYGM	0.6	0.1	-0.2	0.1	0.3	1.3	3.0	2.1	1	1

Q29387	PIG Elongation factor 1-gamma (Fragment)	EEF1G	-1.2	-0.7	1.0	1.5	-1.3	-0.7	0.3	0.3	18	17
I3LKU0	PIG Uncharacterized protein	RAC2	-0.5	0.1	0.5	0.6	-1.3	-0.9	-0.5	0.3	4	2
F2Z558	PIG Uncharacterized protein (Fragment)	YWHAZ	-0.5	-0.7	0.6	1.4	-0.6	-0.4	-0.3	-0.4	2	2
F1SSN5	PIG Uncharacterized protein	KTN1	0.2	0.0	0.1	0.4	0.5	0.7	-0.2	0.8	16	11
F1RKF2	PIG Nitric oxide synthase	NOS1	-1.1	1.3	-1.2	1.7	2.7	-1.0	9.9	3.1	1	1
F1SUB2	PIG Uncharacterized protein (Fragment)	SGPL1	0.1	0.0	0.3	0.3	0.1	0.8	0.1	-0.3	2	1
P35527	HUMAN Keratin, type I cytoskeletal 9	KRT9	1.0	0.0	-0.1	-0.2	2.2	3.5	6.9	4.1	17	15
F1SD87	PIG Uncharacterized protein (Fragment)	FBLN5	0.3	0.3	0.0	0.0	0.7	0.7	0.9	1.2	6	4
I3LI20	PIG Uncharacterized protein	ABCD3	0.4	-0.2	0.1	0.3	3.2	1.4	1.2	1.0	1	2
I3LJT9	PIG Protein phosphatase methylesterase 1 (Fragment)	PPME1	-0.2	-0.2	1.2	-0.2	-0.3	-0.5	-0.4	-0.3	3	5
F1RQ85	PIG Uncharacterized protein	NA	0.3	0.1	0.1	0.1	0.1	-0.9	-1.0	-2.5	1	1
Q9UQ90	HUMAN Paraplegin	SPG7	0.5	0.4	-0.1	-0.1	0.0	-0.4	-1.3	0.3	2	2
F1SUD8	PIG Uncharacterized protein (Fragment)	C9orf89	1.5	0.5	-0.4	-0.9	0.4	0.3	-0.6	-1.2	1	1
Q15019	HUMAN Septin-2	SEPT2	0.0	-0.3	0.0	0.9	0.6	0.5	-0.3	-1.0	1	1
P17661	HUMAN Desmin	DES	-0.1	-0.3	0.7	0.3	0.2	0.2	-0.3	-2.2	1	1
Q9BUB5	HUMAN MAP kinase-interacting serine/threonine-protein kinase 1	MKNK1	-0.6	0.0	0.3	1.0	3.0	1.8	2.3	0.1	1	1
I3LMU5	PIG Uncharacterized protein	NA	0.1	0.0	0.1	0.4	-0.1	0.1	2.8	0.4	6	2
F1SCY0	PIG Uncharacterized protein (Fragment)	IFIT5	-0.1	-0.1	0.2	0.6	7.5	5.9	1.2	2.5	2	1
F1RPM0	PIG Uncharacterized protein (Fragment)	LOC100525092	0.0	0.1	0.1	0.5	0.1	0.2	0.9	0.3	1	1
I3LCP8	PIG Uncharacterized protein	HMSD	0.7	0.1	0.0	-0.2	-0.4	-1.4	-1.4	0.2	1	1
I3LCA1	PIG 6-phosphofructokinase, muscle type	PFKM	0.2	0.0	0.4	0.0	-0.9	-1.5	-0.8	-0.1	1	3
Q29315	PIG 60S acidic ribosomal protein P2	RPLP2	0.4	0.5	0.0	-0.1	2.3	1.1	-0.1	-1.5	3	3
O97763	PIG Epididymal secretory protein E1	NPC2	-0.4	-0.6	0.6	1.0	-1.0	-0.2	0.0	-0.3	1	2
Q92545	HUMAN Transmembrane protein 131	TMEM131	0.7	0.2	-0.1	-0.2	-0.6	0.6	-0.8	-1.0	1	1
I3LTY2	PIG Uncharacterized protein (Fragment)	DOCK9	0.3	0.3	0.1	-0.1	-0.5	-0.6	-1.3	0.2	2	1
I3LSD3	PIG 60S ribosomal protein L13	RPL13	-0.7	-0.6	0.6	1.3	-0.4	0.2	0.1	1.9	9	9
B4E031	HUMAN cDNA FLJ54213, moderately similar to Programmed cell death protein 7	NA	1.1	0.5	-0.5	-0.5	5.3	1.9	0.4	-0.5	1	1
F1RNW4	PIG Uncharacterized protein	LOC100739263	-0.6	-0.3	0.7	0.8	0.8	1.9	0.0	-0.1	5	3
F1RJM2	PIG Endoplasmic reticulum resident protein 29 (Fragment)	ERP29	0.7	0.2	-0.1	-0.2	0.2	0.6	-0.5	0.0	6	3
I3LS62	PIG Ribosomal protein L15	NA	-0.7	-1.0	0.8	1.6	-1.8	-1.1	-0.5	0.7	3	3
Q29550	PIG Liver carboxylesterase	NA	0.7	1.0	-0.3	-0.8	0.6	1.4	0.5	-1.8	2	2
F1SIK9	PIG Uncharacterized protein	SEPT7	0.0	-0.2	0.1	0.6	-0.3	0.1	-0.2	-0.6	9	4
P13645	HUMAN Keratin, type I cytoskeletal 10	KRT10	0.2	0.9	-0.3	-0.2	0.1	-1.9	0.4	4.2	15	12
P04114	HUMAN Apolipoprotein B-100	APOB	0.6	-0.1	-0.2	0.2	2.3	1.9	0.9	-1.1	6	1
F1S4G6	PIG Uncharacterized protein	TMEM33	0.0	0.1	0.3	0.2	-0.6	-0.7	0.6	0.3	1	1
Q58FF7	HUMAN Putative heat shock protein HSP 90-beta-3	HSP90AB3P	-1.4	-0.7	1.3	1.4	-0.1	1.6	-0.1	1.5	1	1
C5I4T6	PIG Phosphoenolpyruvate carboxykinase 2	PCK2	0.3	-0.4	0.1	0.7	-2.0	-1.1	-1.5	0.1	3	1
P23229	HUMAN Integrin alpha-6	ITGA6	0.5	0.6	0.1	-0.6	-1.2	-0.7	0.2	0.6	1	1
F1SCS2	PIG Uncharacterized protein	MYCN	-0.6	-0.1	0.5	0.7	-0.6	-0.1	1.7	-0.2	1	1
O75342	HUMAN Arachidonate 12-lipoxygenase, 12R-type	ALOX12B	-1.1	0.1	1.1	0.5	0.3	0.2	2.5	1.8	1	1

F1RX74	PIG Uncharacterized protein (Fragment)	HNRNPA2B1	-0.5	-0.6	0.6	1.1	-0.1	1.3	0.6	0.4	12	11
F1S848	PIG Uncharacterized protein	NT5C2	-0.1	0.0	0.2	0.5	-0.5	-0.3	-0.4	0.0	2	2
F1RIE2	PIG Uncharacterized protein	DDB1	0.4	-0.2	0.1	0.3	-0.4	-0.1	0.1	-1.4	10	11
F1RPH6	PIG Uncharacterized protein (Fragment)	MAGT1	-1.2	-0.2	0.5	1.4	-0.5	-0.7	-0.6	0.6	2	2
P25705	HUMAN ATP synthase subunit alpha, mitochondrial	ATP5A1	1.1	0.6	-0.3	-0.8	1.2	-0.4	0.1	0.6	1	2
I3LEF8	PIG Uncharacterized protein	HSD17B4	0.6	0.0	-0.1	0.0	-0.3	-0.1	-0.6	-0.2	9	7
P62136	HUMAN Serine/threonine-protein phosphatase PP1-alpha catalytic subunit	PPP1CA	-0.2	-0.2	0.4	0.6	0.0	0.3	-0.2	-0.3	2	2
F1SGC2	PIG Uncharacterized protein	CCDC39	0.3	-0.2	0.0	0.5	0.7	0.5	-0.4	0.2	1	1
F1RKI0	PIG Uncharacterized protein (Fragment)	RAPGEF6	0.6	-0.2	-0.2	0.3	0.2	-0.2	-1.1	-3.1	1	1
F1RR06	PIG Glycylpeptide N-tetradecanoyltransferase	NMT1	0.2	-0.3	0.2	0.3	-1.0	-0.4	-1.1	0.4	3	4
Q8MJ31	PIG Hepatocyte growth factor activator (Fragment)	NA	-0.2	0.5	-0.4	0.6	2.5	4.7	1.7	1.9	1	1
F1SG10	PIG Uncharacterized protein	PRPS2	-0.4	-0.2	0.3	0.8	0.3	1.5	0.4	0.0	2	1
H3BQD4	HUMAN Ribosome biogenesis protein TSR3 homolog (Fragment)	TSR3	0.2	0.1	0.6	-0.3	0.6	0.0	0.1	0.6	1	1
P04264	HUMAN Keratin, type II cytoskeletal 1	KRT1	0.2	0.7	-0.2	-0.2	1.0	-0.2	5.1	9.1	20	25
G0WLG4	PIG RING finger protein 3	murf3	1.1	-0.1	-0.4	-0.1	-0.3	0.0	-0.5	0.1	4	1
P61086	HUMAN Ubiquitin-conjugating enzyme E2 K	UBE2K	0.4	-0.5	0.0	0.6	0.7	3.0	-0.2	-0.8	4	1
P09874	HUMAN Poly [ADP-ribose] polymerase 1	PARP1	0.5	-0.1	0.1	0.1	0.5	0.9	1.0	0.7	1	1
Q95274	PIG Thymosin beta-4	TMSB4	-1.2	0.1	0.7	0.9	2.3	-0.2	-0.9	-1.4	2	1
K9IVN9	PIG Thyroid hormone receptor-associated protein 3	THRAP3	-1.0	-1.3	0.8	1.9	-2.0	-1.0	-0.8	1.4	1	3
P02550	PIG Tubulin alpha-1A chain	TUBA1A	-0.5	-0.8	0.6	1.3	0.0	0.0	-0.3	-0.5	21	16
F1SGG3	PIG Uncharacterized protein	KRT1	0.5	0.5	-0.4	-0.1	0.7	-0.4	5.4	8.4	7	7
F1SP81	PIG Uncharacterized protein (Fragment)	PLAA	0.4	-0.3	0.0	0.4	-0.4	-1.1	-0.6	-0.6	4	4
F1SJY2	PIG Uncharacterized protein	LOC100520175	0.6	0.5	-0.1	-0.5	0.8	0.0	-0.5	-0.6	2	3
Q1W674	PIG Hexokinase-2	HK2	0.6	0.5	0.1	-0.6	0.0	-0.1	0.0	0.2	6	6
F1RJE4	PIG Uncharacterized protein	LOC100525876	0.3	-0.1	0.1	0.2	0.6	0.8	0.2	0.0	8	9
Q86SX3	HUMAN Uncharacterized protein C14orf80	C14orf80	0.0	0.2	-0.1	0.4	-1.6	-1.9	-2.0	-1.1	1	1
I3LQH4	PIG Uncharacterized protein (Fragment)	NA	-0.3	-0.8	0.5	1.1	-0.8	-0.3	-0.7	-0.6	1	1
I3LCR2	PIG Uncharacterized protein (Fragment)	NA	0.8	-0.4	-0.1	0.2	1.3	2.1	-0.4	-1.7	1	1
F2Z5G2	PIG Uncharacterized protein	SMC3	-0.5	-0.5	0.5	1.0	-0.8	0.2	-1.6	0.3	3	3
P46782	HUMAN 40S ribosomal protein S5	RPS5	-0.3	-0.3	0.4	0.7	-0.8	-0.6	0.3	0.2	6	6
Q02388	HUMAN Collagen alpha-1(VII) chain	COL7A1	0.5	0.1	0.0	-0.1	-0.5	-0.6	1.2	1.6	4	3
F1RFN9	PIG Fascin	FSCN1	-0.6	-0.3	0.3	1.1	-0.2	0.7	-0.5	-0.6	13	9
B6V8C8	PIG 40S ribosomal protein S3a	RPS3A	-0.8	-0.6	0.7	1.2	0.1	0.2	0.6	0.8	12	9
F1S9K5	PIG Uncharacterized protein	EPRS	-0.1	-0.3	0.3	0.6	-0.2	-0.3	-0.1	0.0	18	12
F1S420	PIG Uncharacterized protein	NA	-0.8	-0.4	0.7	1.1	1.3	2.5	2.2	1.5	5	3
Q9HDC9	HUMAN Adipocyte plasma membrane-associated protein	APMAP	0.7	1.1	-0.6	-0.7	3.0	2.9	-0.2	-3.6	1	1
I3LQD3	PIG Calpain-2 catalytic subunit (Fragment)	CAPN2	0.4	0.0	0.0	0.0	-0.2	-0.5	-1.1	-0.7	1	1
Q96GQ5	HUMAN UPF0420 protein C16orf58	C16orf58	0.6	0.5	0.1	-0.6	-2.0	-4.1	-4.0	-2.6	1	1
F1SP56	PIG Uncharacterized protein	NIPSNAP3A	0.7	0.1	-0.1	-0.1	0.4	0.3	1.5	-0.8	5	6
P28838	HUMAN Cytosol aminopeptidase	LAP3	0.4	-0.1	-0.2	0.4	0.2	0.5	2.3	1.0	4	4

O43681	HUMAN ATPase ASNA1	ASNA1	0.9	0.1	-0.3	-0.2	-0.2	0.0	-1.4	-2.5	6	5
I3LIC8	PIG Uncharacterized protein	RASIP1	0.3	-0.1	-0.1	0.3	-0.8	1.1	0.2	1.5	2	2
F1RHW4	PIG Uncharacterized protein	RRAS	0.3	0.1	0.1	0.1	0.2	0.7	0.4	-0.5	5	6
K7GRN9	PIG Uncharacterized protein	FAM129A	0.0	0.2	-0.1	0.4	-0.3	-1.5	-2.2	-0.7	5	1
F1S8Y2	PIG Uncharacterized protein (Fragment)	MMS19	0.6	0.0	0.1	-0.2	0.9	0.4	-0.5	-1.2	4	3
F1SG95	PIG Uncharacterized protein	MRPS35	0.5	-0.1	-0.1	0.1	0.6	1.7	0.2	1.1	1	1
O18866	PIG Calcium-activated potassium channel subunit alpha-1 (Fragment)	KCNMA1	0.9	0.1	-0.2	-0.3	-1.9	-1.3	-0.8	1.1	1	1
O75161	HUMAN Nephrocystin-4	NPHP4	0.1	-0.2	0.2	0.4	0.4	0.8	-2.5	-1.6	1	2
Q28956	PIG 17beta-estradiol dehydrogenase	NA	0.5	-0.1	0.1	-0.1	1.1	1.6	-0.1	-0.2	6	5
F1SU28	PIG Uncharacterized protein	LIMS1	-0.3	-0.2	0.1	0.8	-0.8	-0.8	-0.4	-0.2	3	2
P49721	HUMAN Proteasome subunit beta type-2	PSMB2	0.0	-0.5	0.3	0.6	0.7	0.2	0.6	-0.4	4	3
I3L688	PIG Uncharacterized protein	NA	-0.1	-0.2	0.4	0.3	-0.6	-1.2	-0.3	0.3	2	2
I3LQ51	PIG Proteasome subunit beta type	PSMB1	0.3	-0.2	0.2	0.2	1.4	0.6	1.7	-0.5	8	6
F1RU33	PIG Uncharacterized protein	LOC100522848	-0.5	0.0	0.3	0.7	0.1	-0.6	-0.6	-0.9	3	2
F1SD98	PIG Uncharacterized protein	TRMT1	-0.1	-0.3	0.2	0.6	0.4	0.8	0.6	0.3	1	1
D0G6Y0	PIG Hydroxysteroid (17-beta) dehydrogenase 12 (Fragment)	HSD17B12	0.4	0.2	0.0	-0.1	-0.2	-0.1	0.3	-0.4	1	1
F1SKJ5	PIG Eukaryotic translation initiation factor 3 subunit D	EIF3D	0.3	0.1	0.1	0.0	1.6	0.3	0.0	0.0	3	2
F1SC07	PIG Uncharacterized protein (Fragment)	DNAH11	0.5	-0.2	0.1	0.1	1.2	0.3	0.6	0.3	4	1
F1SP32	PIG Uncharacterized protein	RAD23B	-0.5	-0.2	0.3	0.8	2.9	2.4	0.8	-0.5	10	5
F1RP39	PIG Uncharacterized protein	IQGAP3	0.1	-0.2	0.1	0.4	-0.1	0.2	0.4	0.2	2	1
Q64L94	PIG Proteasome activator complex subunit 1	PSME1	-0.2	-0.1	0.3	0.4	0.9	0.3	-0.2	-0.7	15	13
K7GT00	PIG Uncharacterized protein (Fragment)	COL14A1	0.1	0.0	-0.1	0.4	0.7	0.8	-0.1	0.3	20	17
F1SK86	PIG Uncharacterized protein	FKBP4	-0.7	0.1	0.5	0.6	-2.2	-5.2	-4.2	1.9	6	1
P57058	HUMAN Hormonally up-regulated neu tumor-associated kinase	HUNK	0.3	0.6	-0.2	-0.3	1.1	1.5	0.8	0.5	1	1
F1ST01	PIG Uncharacterized protein	SELENBP1	0.2	0.6	-0.1	-0.3	0.9	1.3	1.2	0.4	13	13
F1SDX9	PIG Peroxiredoxin-2	PRDX2	-0.4	0.0	0.6	0.2	0.2	-0.1	0.5	-0.2	1	1
Q29386	PIG Keratin type II cytoskeletal 8 (Fragment)	NA	0.6	0.2	0.2	-0.6	-0.1	-1.4	1.1	5.6	1	1
F1SBT6	PIG Uncharacterized protein	KHSRP	-0.2	-0.1	0.1	0.6	2.0	2.5	2.3	0.4	14	7
F1RLB6	PIG Uncharacterized protein	NAPA	0.2	0.2	0.1	-0.1	0.6	0.0	-1.0	0.2	6	3
F1S9Y8	PIG Uncharacterized protein (Fragment)	DYNC1H1	0.1	-0.2	0.0	0.5	-0.5	0.1	-1.0	-0.2	20	21
I3LK68	PIG Uncharacterized protein (Fragment)	PFKP	0.0	-0.3	0.3	0.3	1.1	1.2	1.0	0.1	6	5
F1RWX8	PIG Uncharacterized protein	UBA1	-0.8	-0.7	0.5	1.4	-0.5	-0.8	-0.1	0.6	2	1
F1REW5	PIG Uncharacterized protein (Fragment)	CLIP1	0.2	0.4	-0.1	0.0	0.1	-1.0	-0.9	0.0	8	6
F1SQ68	PIG Uncharacterized protein	TCTP	-0.1	-0.2	0.4	0.3	4.5	1.7	0.3	1.8	1	1
F1SJG4	PIG Uncharacterized protein	DMRT2	0.3	-0.3	0.1	0.3	-1.2	-0.6	-0.5	-1.2	1	1
Q8WUM4	HUMAN Programmed cell death 6-interacting protein	PDCD6IP	-0.5	-0.2	0.5	0.5	-0.2	-0.5	-0.2	-1.3	3	2
Q9HCS4	HUMAN Transcription factor 7-like 1	TCF7L1	0.1	0.1	-0.2	0.5	-1.1	0.3	0.2	0.1	1	1
F1RY01	PIG Uncharacterized protein	NDUFAF4	0.4	0.7	-0.1	-0.6	1.4	0.5	0.1	0.4	2	1
F1SAD7	PIG Uncharacterized protein	CUL1	0.7	0.0	-0.2	-0.1	0.0	0.4	-0.9	0.1	1	2
F1RJL2	PIG Uncharacterized protein	GTF2I	-0.1	-0.1	0.1	0.5	0.5	1.6	-0.2	-1.7	2	1

F1SLG9	PIG Uncharacterized protein (Fragment)	MOB1A	-0.2	-0.5	0.5	0.5	0.8	0.8	1.1	-0.3	1	1
F1SS19	PIG Uncharacterized protein	XRCC5	-0.6	-0.6	0.5	1.2	-0.1	-0.3	1.7	2.3	1	1
B0LY44	PIG Mitochondrial ornithine aminotransferase	NA	0.5	-0.1	0.0	-0.1	0.1	-0.5	-0.7	-0.5	1	1
I3LNA5	PIG Uncharacterized protein	TMEM151B	-0.3	-0.3	0.2	0.7	0.4	1.6	1.9	0.3	1	1
F1S1G0	PIG Eukaryotic translation initiation factor 3 subunit E	EIF3E	-0.5	-0.3	0.5	0.7	-1.0	-0.5	0.7	0.4	5	2
F1S2J1	PIG Uncharacterized protein	FAM65A	-0.3	-0.7	0.4	0.9	-0.3	-0.5	-0.2	0.2	1	1
F1SGI7	PIG Uncharacterized protein (Fragment)	KRT75	0.6	0.4	-0.3	-0.3	-0.1	-1.6	-1.3	9.9	3	2
I3LU86	PIG Uncharacterized protein	MTUS2	0.1	1.1	-0.2	-0.7	-0.3	-1.0	-1.8	-2.3	1	1
F1SFE6	PIG Uncharacterized protein	PSMC6	-0.2	-0.1	0.3	0.3	0.6	1.1	-0.1	-0.9	7	3
F1STM4	PIG Elongation factor 1-alpha	Ssc.41914	-1.4	-0.9	1.2	1.5	-1.4	-0.8	0.1	0.4	8	8
B8Q0B4	PIG Popeye domain containing protein 2 variant 2	NA	0.7	-0.3	-0.1	0.0	2.0	4.4	0.2	0.3	2	1
K9IVK1	PIG Hexose-6-phosphate dehydrogenase (Glucose 1-dehydrogenase)	H6PD	-0.1	0.0	0.4	0.1	-1.2	-0.9	-0.9	-1.1	3	1
F1RM25	PIG Serine/threonine-protein phosphatase (Fragment)	PPP5C	-0.6	0.1	0.5	0.4	0.1	0.7	-0.2	-0.1	2	3
I3LPB5	PIG Creatine kinase B-type (Fragment)	CKB	0.1	-0.9	0.1	1.0	0.8	0.2	0.3	1.0	9	3
F2Z4Z8	PIG Uncharacterized protein (Fragment)	LOC100620305	-0.2	-0.4	0.3	0.6	0.1	0.7	0.0	-0.9	2	2
Q0VD83	HUMAN Apolipoprotein B receptor	APOBR	0.6	0.5	-0.3	-0.5	-1.7	-1.3	-1.4	-0.8	1	1
F1S459	PIG Uncharacterized protein	TERF2IP	0.2	0.0	0.0	0.1	-0.1	-0.2	0.0	-0.1	1	1
Q07717	PIG Beta-2-microglobulin	B2M	-0.3	-0.4	0.4	0.7	0.4	-0.3	0.6	-0.5	3	2
I3LKS6	PIG Dihydropteridine reductase	QDPR	-0.1	-0.2	0.0	0.7	-0.2	0.6	-0.5	0.2	1	1
I3LJ87	PIG Uncharacterized protein	RPS2E	-0.6	-0.9	0.6	1.3	-1.0	-0.4	-0.4	0.3	10	9
P31942	HUMAN Heterogeneous nuclear ribonucleoprotein H3	HNRNPH3	0.5	0.1	-0.2	-0.1	-0.1	0.2	-0.2	-0.5	1	1
F2Z5S1	PIG Uncharacterized protein (Fragment)	RAB6B	0.0	0.1	0.0	0.2	-0.5	0.8	0.2	0.4	4	3
I3L9T6	PIG Uncharacterized protein	LOC100739576	-0.3	0.2	-0.2	0.7	0.5	1.2	0.6	0.2	11	9
F1RQR4	PIG Uncharacterized protein (Fragment)	EHD1	0.4	0.0	0.0	-0.2	0.1	0.2	-0.3	-0.2	14	9
B8XSJ9	PIG Monoglyceride lipase	MGLL	-0.2	-0.5	0.4	0.5	-0.6	-0.8	-1.0	-0.6	3	3
F1SE70	PIG Uncharacterized protein	SGCB	0.8	0.2	-0.1	-0.6	0.0	-0.2	0.2	0.2	5	5
Q6IAA8	HUMAN Ragulator complex protein LAMTOR1	LAMTOR1	0.4	0.2	-0.2	-0.2	0.1	-1.0	-1.4	-0.7	1	1
F1SFQ4	PIG Uncharacterized protein	PCBP2	-0.6	-0.7	0.6	1.0	0.5	0.9	0.3	1.1	6	7
Q29190	PIG Ribosomal protein S7 (Fragment)	NA	-0.3	-0.6	0.2	1.0	0.2	-0.6	0.6	-0.1	2	2
Q95342	PIG 60S ribosomal protein L18 (Fragment)	RPL18	-0.2	-0.6	0.3	0.8	-0.1	0.5	-0.7	0.9	4	3
I3LFI0	PIG Uncharacterized protein (Fragment)	RAC1	0.3	-0.2	0.0	0.2	-0.7	-0.5	-0.1	0.6	3	2
A7TX81	PIG COP9 signalosome complex subunit 6	COPS6	-0.1	-0.1	0.1	0.3	-2.1	-1.6	0.6	-1.1	3	1
I3LHZ6	PIG Uncharacterized protein	DHX9	0.1	-0.2	0.1	0.3	-0.3	-0.1	-0.7	-0.4	17	12
B6VAP9	PIG APEX nuclease 1	APEX1	0.5	0.0	-0.2	0.0	0.5	0.7	2.1	2.2	1	1
F1RI15	PIG Uncharacterized protein	HSPA4	-0.1	-0.3	0.2	0.5	0.0	0.1	0.0	0.6	21	19
F1STD5	PIG Uncharacterized protein (Fragment)	ACTR1B	0.0	0.2	0.0	0.1	0.3	0.5	0.0	0.0	2	4
F1SIH8	PIG Uncharacterized protein	LOC100516776	-0.1	-0.3	0.2	0.4	0.2	0.4	0.6	0.0	1	1
Q5HYK3	HUMAN 2-methoxy-6-polyprenyl-1,4-benzoquinol methylase, mitochondrial	COQ5	-0.5	0.3	0.4	0.1	0.2	0.6	-1.1	-1.8	1	1
P61006	HUMAN Ras-related protein Rab-8A	RAB8A	-0.2	0.9	0.3	-0.8	0.8	1.5	0.5	0.4	1	1
Q2VL90	PIG Scavenger receptor cysteine-rich type 1 protein M130	CD163	-0.2	-0.3	0.3	0.6	-0.3	1.4	3.1	2.0	4	1

F1RMM0	PIG Uncharacterized protein	PRKCDBP	-0.3	-0.2	0.0	0.7	0.0	-0.4	-0.2	0.9	3	2
P48729	HUMAN Casein kinase I isoform alpha	CSNK1A1	-0.8	-0.4	0.3	1.2	-1.0	-0.7	-0.9	-0.1	1	1
Q9NYU1	HUMAN UDP-glucose:glycoprotein glucosyltransferase 2	UGGT2	3.4	-0.2	-1.6	-1.3	3.7	0.8	-0.9	-0.5	1	1
F1RTK4	PIG Uncharacterized protein (Fragment)	CDK8	-1.6	-0.1	0.8	1.1	-1.8	0.6	-0.3	0.7	1	1
Q9Y2D5	HUMAN A-kinase anchor protein 2	AKAP2	0.3	0.1	-0.3	0.1	0.5	1.4	0.6	-1.4	6	4
F1RRY6	PIG Uncharacterized protein (Fragment)	PTGES2	1.1	0.3	-0.5	-0.7	0.9	0.7	-0.2	-1.1	6	6
I3LCE6	PIG Uncharacterized protein	BAZ1B	-0.3	-0.3	0.2	0.7	-0.6	-0.4	0.6	1.5	1	2
P12814	HUMAN Alpha-actinin-1	ACTN1	-1.0	-1.1	-0.1	2.5	-1.0	-1.0	-1.0	-0.8	4	5
F1SA60	PIG Uncharacterized protein (Fragment)	SPTB	-0.2	0.4	0.4	-0.4	-0.1	0.1	-0.1	0.6	44	56
F1S409	PIG Uncharacterized protein	SEC23IP	0.4	0.8	-0.3	-0.7	0.1	0.5	-0.1	0.3	3	2
F1SDY2	PIG Uncharacterized protein (Fragment)	NA	-0.3	0.7	0.7	-0.9	-1.1	-0.5	0.7	0.9	1	1
F1RUU2	PIG Uncharacterized protein	MRPS10	0.4	0.3	-0.3	-0.2	1.7	2.0	-0.2	-0.4	1	1
F1S069	PIG Uncharacterized protein	PDK1	0.9	0.5	-0.5	-0.6	2.0	1.7	-0.1	-0.4	3	3
F1S4A6	PIG Uncharacterized protein (Fragment)	SYNJ2BP	-0.2	0.3	0.1	0.1	-0.5	-0.4	-1.1	-0.6	2	1
Q863I2	PIG Serine/threonine-protein kinase OSR1	OXSR1	0.2	-0.1	0.1	0.0	0.0	0.7	-0.9	-0.3	7	8
P10775	PIG Ribonuclease inhibitor	RNH1	0.1	-0.8	0.3	0.7	0.0	1.5	0.6	0.3	4	1
I3LAQ3	PIG Phosphatidate cytidylyltransferase	CDS2	0.6	-0.2	0.1	-0.3	0.6	0.6	-0.7	0.7	1	2
F1SLS7	PIG Autophagy-related protein 3	ATG3	0.0	-0.2	0.1	0.3	-0.6	-0.5	-1.0	-1.6	3	1
I3LC46	PIG Uncharacterized protein	HTATSF1	0.1	-0.1	0.1	0.1	1.3	2.3	0.2	-0.4	2	3
I3LPY1	PIG Uncharacterized protein (Fragment)	TNS1	0.4	0.0	-0.2	0.0	-0.4	1.1	-0.2	-0.4	5	6
G9M4N3	PIG Toll interacting protein	Tollip	0.3	-0.4	0.1	0.2	1.1	0.7	0.6	-1.3	2	3
F1SCH1	PIG Uncharacterized protein (Fragment)	NDUFB7	0.5	0.4	-0.5	-0.2	1.6	0.4	0.3	1.2	3	3
F1SFF4	PIG Uncharacterized protein	C14orf166	-0.2	-0.1	0.2	0.4	0.7	-0.3	-1.9	-2.1	7	3
I3LB23	PIG Uncharacterized protein (Fragment)	TMX4	1.1	-0.5	-0.2	-0.2	0.3	-0.4	0.3	-0.3	2	2
F1RPU0	PIG Uncharacterized protein	MGPD	0.5	0.1	-0.3	-0.1	0.4	0.3	1.1	-0.2	4	3
Q6PQZ1	PIG Aquaporin-1	AQP1	0.2	0.1	-0.1	0.0	0.6	1.4	0.3	1.2	2	2
F1S8A5	PIG Uncharacterized protein	MRPS26	0.5	0.3	-0.4	-0.2	0.1	-0.9	0.5	0.3	3	2
F1S0C8	PIG Uncharacterized protein	RRAGD	0.0	-0.3	0.0	0.5	0.9	-0.9	-0.2	0.3	1	1
Q767L0	PIG ATP-binding cassette sub-family F member 1	ABCF1	-0.2	-0.3	0.2	0.5	-0.2	0.1	-0.2	1.4	3	2
P49171	PIG 40S ribosomal protein S26	RPS26	-0.3	-0.5	0.2	0.7	-0.7	0.0	0.5	0.1	2	2
F1RQW4	PIG Uncharacterized protein	SKIV2L	-0.4	-0.6	0.4	0.7	0.8	1.2	-0.3	-1.0	1	1
K7GNV3	PIG Uncharacterized protein	LOC100514935	-0.6	0.1	0.3	0.4	-1.6	-1.5	-1.3	-0.3	2	1
L0R890	HUMAN Alternative protein LGI2	LGI2	0.5	-0.1	-0.3	0.0	0.0	-0.1	-0.8	-0.4	1	1
P81271	PIG Myosin-11 (Fragments)	MYH11	-0.3	-0.3	0.0	0.8	-0.4	0.2	0.9	0.5	1	1
F1SN59	PIG Uncharacterized protein (Fragment)	NA	-0.3	-0.2	0.0	0.6	-1.2	-1.2	-2.6	-0.7	7	3
Q9BXJ9	HUMAN N-alpha-acetyltransferase 15, NatA auxiliary subunit	NAA15	0.5	0.8	-0.5	-0.6	0.8	0.6	0.1	-0.7	1	1
Q95L19	PIG Ribosomal protein L19 (Fragment)	NA	-0.4	-0.6	0.5	0.7	-0.4	0.2	0.0	0.8	5	3
I3LR88	PIG Uncharacterized protein (Fragment)	LMO7	-0.3	-0.2	0.3	0.3	0.4	0.1	-0.5	-0.5	1	3
F1SFH7	PIG Uncharacterized protein	LOC100738983	0.4	-0.3	-0.2	0.4	0.8	1.5	0.6	-0.1	4	5
F1RSM2	PIG Uncharacterized protein	PSMD12	-0.5	-0.4	0.6	0.4	1.5	0.7	-0.8	-0.1	3	5

F1RZA6	PIG Uncharacterized protein (Fragment)	RNF213	0.4	-0.3	0.0	0.1	0.0	0.6	1.5	0.4	1	2
I3LGP0	PIG Uncharacterized protein (Fragment)	NA	0.4	-0.1	-0.3	0.1	-0.4	1.4	0.8	-1.0	2	1
Q31072	PIG MHC class II antigen	LA-DRB-d	-0.5	0.0	0.4	0.3	2.0	2.7	1.0	0.8	4	1
P80031	PIG Glutathione S-transferase P	GSTP1	0.3	0.2	-0.2	-0.1	1.3	2.5	0.9	-0.9	6	6
F1S1G7	PIG Amine oxidase	AOC2	0.5	0.0	-0.2	-0.1	-0.4	-0.5	0.9	0.6	3	1
P02538	HUMAN Keratin, type II cytoskeletal 6A	KRT6A	1.0	0.0	-0.1	-0.6	-1.6	-2.8	-3.2	11.6	1	3
Q14097	HUMAN CYP2B protein	CYP2B	-0.9	-0.1	0.3	0.9	0.2	0.0	-0.3	-1.8	1	1
I3LLG4	PIG Uncharacterized protein	DNAJA3	0.5	0.6	-0.1	-0.9	0.2	1.1	-0.2	0.1	3	3
F1SUF3	PIG Uncharacterized protein (Fragment)	HKDC1	0.6	0.3	-0.2	-0.6	0.2	0.6	0.3	0.2	1	2
Q27HS3	PIG Vascular smooth muscle alpha-actin (Fragment)	NA	0.1	-0.2	-0.1	0.4	0.3	0.4	0.6	1.5	1	1
I3LAE9	PIG Uncharacterized protein	LOC100523801	0.1	-0.1	0.0	0.1	-0.3	0.7	0.5	0.0	8	2
F1SCJ1	PIG Uncharacterized protein	TRAPPC5	-0.3	-0.5	0.1	0.9	-0.6	-0.2	-0.1	0.0	1	1
I3LIK0	PIG Uncharacterized protein (Fragment)	RPL35A	-0.7	-1.2	0.7	1.3	-0.7	-1.3	-0.3	-0.3	1	1
I3LPR6	PIG Uncharacterized protein	NA	0.2	-0.3	0.0	0.3	-1.6	-1.7	-1.7	-0.9	1	1
F1ST02	PIG Proteasome subunit beta type	PSMB4	-0.1	-0.6	0.4	0.5	-0.1	-0.6	1.7	1.1	2	2
I3LRW1	PIG Uncharacterized protein (Fragment)	KAT2B	-1.1	0.4	0.5	0.4	-2.1	-4.5	-1.4	-3.1	1	1
F1SQM6	PIG Uncharacterized protein	LOC100739488	0.5	-0.1	-0.1	-0.1	-1.0	-1.1	-1.2	-0.3	3	2
I3LC07	PIG Uncharacterized protein	RAB18	-0.1	-0.1	0.3	0.0	0.3	1.5	0.9	0.7	6	6
F1SGB0	PIG Uncharacterized protein	FXR1	0.2	0.2	0.0	-0.2	-0.2	-0.5	-0.5	-0.2	1	1
Q59IP2	PIG Procollagen alpha 2(V)	COL5A2	-0.1	-0.4	0.1	0.6	1.5	0.4	0.0	-0.1	3	3
B8LFE3	PIG Cardiomyopathy associated 1	CMYA1	0.1	-0.3	0.2	0.1	2.4	2.9	0.3	0.8	2	2
F2Z5G8	PIG Uncharacterized protein (Fragment)	RPS25	-0.5	-0.7	0.3	1.0	-0.6	0.0	-0.2	0.4	7	6
F1SLE7	PIG Uncharacterized protein	LOC100522732	0.2	0.3	-0.1	-0.2	0.9	0.9	-0.9	-1.3	7	9
I3L652	PIG Uncharacterized protein	NA	0.5	0.6	-0.2	-0.8	0.5	0.1	-1.1	-0.9	3	4
I3L8P8	PIG Uncharacterized protein (Fragment)	TACC2	0.8	0.2	-0.4	-0.5	-1.6	-1.8	0.3	2.4	1	1
F1RZT2	PIG Uncharacterized protein	PFDN6	0.2	0.6	-0.2	-0.5	0.2	0.5	-0.1	-0.6	1	1
P63053	PIG Ubiquitin-60S ribosomal protein L40	UBA52	-0.8	-0.2	0.4	0.7	0.2	0.8	1.1	0.9	9	8
Q13825	HUMAN Methylglutaconyl-CoA hydratase, mitochondrial	AUH	0.6	0.7	-0.4	-0.8	-0.5	-2.0	-1.7	-1.6	1	2
I3LKJ6	PIG Uncharacterized protein	GFM1	0.1	0.0	0.0	0.0	0.1	0.2	1.6	0.2	2	3
F1SKZ8	PIG Uncharacterized protein	LOC100523641	-0.3	-0.4	0.2	0.6	-0.9	-0.8	-0.9	0.9	1	3
P83686	PIG NADH-cytochrome b5 reductase 3 (Fragment)	CYB5R3	0.1	-0.3	-0.2	0.5	-0.7	-0.4	-1.0	0.8	13	11
Q96HS1	HUMAN Serine/threonine-protein phosphatase PGAM5, mitochondrial	PGAM5	0.5	0.3	-0.3	-0.4	-0.5	-1.3	-1.2	-0.8	2	1
Q29361	PIG 60S ribosomal protein L35	RPL35	-1.2	-0.8	0.6	1.5	-0.2	0.0	-0.1	1.0	6	4
F1RPN9	PIG Malic enzyme	ME2	0.3	0.2	-0.1	-0.2	0.3	0.2	-1.0	-0.7	3	4
I3LIL4	PIG Uncharacterized protein (Fragment)	MYO1C	0.1	-0.2	0.0	0.2	0.1	0.3	-0.4	-0.7	25	26
A5A791	PIG DNA-repair protein complementing XP-D cells (Fragment)	ERCC2	0.7	0.2	-0.3	-0.4	0.5	0.1	-0.5	-1.5	1	1
F1S7Y2	PIG Uncharacterized protein	ABLIM2	0.5	-0.3	-0.2	0.0	-0.1	0.0	-0.6	0.4	1	1
P82460	PIG Thioredoxin	TXN	-1.2	-0.2	0.6	0.9	0.2	-0.7	-0.2	-1.5	4	3
Q8TC99	HUMAN Fibronectin type III domain-containing protein 8	FNDC8	0.6	0.0	-0.1	-0.4	-0.8	-1.5	-1.6	-1.2	1	1
I3LKY3	PIG Uncharacterized protein (Fragment)	NA	-0.2	-0.4	0.6	0.1	-0.4	-0.7	-0.9	-1.6	3	3

F1RL02	PIG Uncharacterized protein (Fragment)	MAPK1	-0.6	-0.4	0.4	0.7	-0.7	-0.8	-0.6	-0.5	3	1
H7BZJ3	HUMAN Thioredoxin (Fragment)	PDIA3	0.3	0.0	-0.2	-0.1	0.8	0.7	0.4	0.5	1	1
F1SG21	PIG Uncharacterized protein (Fragment)	SHROOM2	0.1	0.1	0.3	-0.4	2.6	3.4	4.3	2.0	2	1
P11142	HUMAN Heat shock cognate 71 kDa protein	HSPA8	0.1	0.1	-0.1	0.0	0.3	0.6	-0.4	-0.4	3	2
F1RMZ4	PIG Uncharacterized protein (Fragment)	LOC100153673	-0.4	0.3	0.0	0.2	1.0	0.8	0.0	0.3	1	1
Q5SSJ5	HUMAN Heterochromatin protein 1-binding protein 3	HP1BP3	-0.2	0.0	0.1	0.1	-0.5	-0.8	-0.7	-0.2	2	2
K7GNJ7	PIG Uncharacterized protein	HUWE1	0.6	0.0	-0.2	-0.3	-1.0	0.2	-1.2	0.4	4	5
B2ZFN7	PIG Actin (Fragment)	NA	0.0	0.2	0.2	-0.3	0.2	-0.4	-0.6	-1.0	1	1
I3LL04	PIG Uncharacterized protein	FAM35A	0.5	-0.2	-0.1	-0.2	1.4	3.1	4.2	0.9	1	1
K7GR37	PIG Uncharacterized protein	SEPT6	-0.2	-0.5	0.1	0.6	0.5	0.5	0.1	0.7	5	2
P68400	HUMAN Casein kinase II subunit alpha	CSNK2A1	-0.1	-0.1	0.1	0.1	-0.4	-0.4	-0.6	-1.2	7	4
F1RIU3	PIG Uncharacterized protein	CCT6A	-0.3	-0.3	0.1	0.6	-0.2	0.1	0.3	0.4	13	10
Q13707	HUMAN ACTA2 protein (Fragment)	ACTA2	0.3	0.2	-0.3	-0.2	0.3	0.0	-0.4	-1.1	5	6
K7GMF7	PIG Uncharacterized protein	ILF2	-0.3	-0.5	0.1	0.7	-0.1	0.4	-0.2	0.2	1	1
F1RPW8	PIG Uncharacterized protein	ASRGL1	0.5	-0.4	0.0	0.0	0.5	1.2	1.3	0.7	6	2
F1RXE5	PIG Uncharacterized protein	LOC100524521	0.4	0.2	0.2	-0.7	0.2	0.0	-0.3	4.7	1	1
A1XQU1	PIG Proteasome subunit beta type-7	PSMB7	-0.2	-0.4	0.2	0.4	0.4	0.2	1.7	0.7	5	4
P07942	HUMAN Laminin subunit beta-1	LAMB1	0.4	-0.5	-0.4	0.6	-0.5	0.2	0.7	1.6	1	2
A1Z624	PIG Dynein cytoplasmic heavy polypeptide-like protein (Fragment)	NA	0.0	0.0	-0.3	0.3	1.4	1.9	1.3	0.7	3	2
F2Z546	PIG Ribosomal protein L19 (Fragment)	RPL19	-0.2	-0.6	0.2	0.7	0.1	-0.5	-0.4	0.5	1	1
Q1T7A5	PIG Type VI collagen alpha-3 chain (Fragment)	COL6A3	-0.8	-0.5	0.1	1.2	-1.2	-1.5	-1.2	0.8	17	19
F1SQR8	PIG Leukotriene A-4 hydrolase	LTA4H	-0.2	0.0	0.1	0.2	-0.4	-1.0	-0.7	-0.1	14	12
F1SL16	PIG Uncharacterized protein	PCNP	-0.7	-0.1	0.4	0.5	0.5	0.4	-0.8	1.5	4	3
I3LB90	PIG Uncharacterized protein	NA	0.4	-0.1	-0.1	-0.1	0.2	0.0	-1.6	0.0	4	3
P06733	HUMAN Alpha-enolase	ENO1	-1.5	-0.5	0.7	1.3	0.0	0.2	-0.1	-0.5	2	2
O43683	HUMAN Mitotic checkpoint serine/threonine-protein kinase BUB1	BUB1	-0.2	0.7	-0.1	-0.4	-0.8	-0.5	-0.1	-0.2	2	1
I3L9T3	PIG Uncharacterized protein	LOC100738263	0.3	0.2	-0.3	-0.2	0.4	0.0	-0.2	-0.2	1	2
I3LF84	PIG Uncharacterized protein	RAP1A	-0.6	-0.5	0.3	0.8	-0.2	0.0	0.3	0.2	6	5
F1RVA0	PIG Histone H3 (Fragment)	H3F3B	-0.9	-0.2	0.0	1.1	-1.1	-0.6	0.4	-0.8	1	1
F1RK45	PIG Uncharacterized protein	TRAP1	-0.1	0.1	0.1	-0.1	0.5	0.1	-0.4	-0.7	7	9
Q9Y3C8	HUMAN Ubiquitin-fold modifier-conjugating enzyme 1	UFC1	0.2	-0.3	0.0	0.1	0.0	-0.2	5.3	-0.6	1	1
F1RL38	PIG Uncharacterized protein	DDT	0.6	-0.1	-0.4	-0.1	1.4	1.1	0.6	-0.9	2	3
I3LE39	PIG Histone H2B (Fragment)	HIST1H2BB	0.1	0.2	-0.3	0.0	0.3	0.0	0.1	-1.1	7	5
Q29243	PIG Dystroglycan (Fragment)	DAG1	-0.4	0.6	-0.1	-0.2	1.4	1.1	-1.5	-1.0	6	1
K7GSE0	PIG Uncharacterized protein (Fragment)	LETM1	0.2	0.3	-0.3	-0.2	0.4	-0.1	-0.6	0.2	18	18
Q9UDY4	HUMAN DnaJ homolog subfamily B member 4	DNAJB4	0.2	-0.3	-0.2	0.3	1.2	1.2	1.1	2.8	1	1
K9J6M4	PIG E3 ubiquitin-protein ligase UBR4	UBR4	0.7	0.1	-0.4	-0.3	-0.4	0.2	1.2	0.8	2	2
F1SHW1	PIG Uncharacterized protein (Fragment)	ESYT2	0.4	-0.3	-0.1	0.1	0.6	0.5	-0.1	0.4	5	8
F1S827	PIG Uncharacterized protein	SERBP1	-0.6	-0.5	0.3	0.8	0.4	1.1	1.3	0.8	14	8
I3LA71	PIG Uncharacterized protein	GSE1	0.5	0.3	-0.2	-0.7	-0.4	-0.7	0.4	0.3	1	1

Q52NJ6	PIG Ras-related protein Rab-14	RAB14	0.1	0.1	-0.1	-0.1	0.7	1.3	0.5	0.7	5	3
F1SJR5	PIG Uncharacterized protein	PACSIN2	0.0	-0.3	-0.1	0.5	0.0	0.0	-0.1	0.0	4	1
F1SS01	PIG Uncharacterized protein (Fragment)	ARPC2	-0.9	-0.6	0.5	1.0	-0.5	-0.7	-0.1	-0.1	11	3
M3VH45	PIG Annexin	ANXA6	0.0	0.5	-0.4	-0.1	0.5	1.0	-0.8	-1.4	4	2
F1SBT2	PIG ATP-dependent Clp protease proteolytic subunit	CLPP	0.7	0.0	-0.4	-0.4	-0.3	-1.0	0.0	-0.3	4	4
P13693	HUMAN Translationally-controlled tumor protein	TPT1	-1.0	-0.3	0.5	0.8	-0.9	0.5	-0.8	-0.9	7	2
F1RZM1	PIG Uncharacterized protein	NA	0.1	0.0	0.0	-0.1	0.0	-0.3	0.5	0.7	1	1
Q8SPC6	PIG MHC class I antigen	SLA-1	0.6	0.4	-0.2	-0.8	0.5	0.0	0.4	0.1	1	1
I3LFZ8	PIG Uncharacterized protein (Fragment)	RAB5B	0.6	0.4	-0.3	-0.7	1.0	1.5	1.5	-0.1	3	5
F1SM07	PIG Uncharacterized protein	COPS8	0.6	0.1	-0.3	-0.4	0.8	4.0	-0.7	-1.5	3	2
P27816	HUMAN Microtubule-associated protein 4	MAP4	-0.2	0.5	-0.3	0.0	1.3	1.1	0.3	-0.7	3	2
I3L8N0	PIG Uncharacterized protein (Fragment)	DEPDC7	0.1	0.0	-0.1	0.0	-1.1	0.0	-0.3	0.2	2	2
F1S593	PIG Uncharacterized protein (Fragment)	DNM2	-0.1	-0.2	0.1	0.1	1.1	0.7	1.5	0.4	3	4
F1S166	PIG Uncharacterized protein	UNC45B	0.5	-0.5	-0.1	0.1	0.6	0.3	0.0	-0.4	10	7
P62993	HUMAN Growth factor receptor-bound protein 2	GRB2	-0.4	-0.6	0.3	0.7	0.0	0.0	-0.3	-0.6	3	3
F1RPX1	PIG Uncharacterized protein	MTA2	-0.4	-0.8	0.2	1.0	-0.1	-0.2	-0.2	0.5	1	1
I3LMP2	PIG Uncharacterized protein (Fragment)	ABHD10	0.0	0.3	-0.2	-0.1	-0.9	-0.6	0.4	0.1	2	3
Q69GF7	PIG Cytochrome c oxidase subunit 2	COX2	0.3	0.9	0.0	-1.2	0.5	-0.4	-0.8	0.4	1	2
F1SPN5	PIG Uncharacterized protein	ADCY6	0.4	0.4	-0.1	-0.8	0.5	-0.4	-3.0	-2.8	1	4
Q5VW36	HUMAN Focadhesin	FOCAD	0.5	-0.1	0.0	-0.5	-0.4	-0.4	-1.0	-0.9	1	1
D2KQJ6	PIG MHC class II antigen (Fragment)	SLA-DRB1	0.2	0.2	-0.4	0.0	1.9	1.1	0.9	1.6	1	1
Q6ZUJ4	HUMAN Uncharacterized protein C3orf62	C3orf62	0.1	0.1	-0.2	-0.1	0.1	0.5	0.0	0.1	1	1
Q00577	HUMAN Transcriptional activator protein Pur-alpha	PURA	0.0	0.2	0.0	-0.2	0.9	0.9	0.3	-0.5	8	7
F1SCT9	PIG Uncharacterized protein	RDH14	0.6	-0.6	-0.1	0.1	0.0	-0.4	0.6	0.7	4	4
F1SNT8	PIG Uncharacterized protein	DCTN1	0.2	-0.3	-0.1	0.1	0.6	0.3	-0.8	-0.7	14	10
Q3SY69	HUMAN Mitochondrial 10-formyltetrahydrofolate dehydrogenase	ALDH1L2	-0.1	0.5	-0.1	-0.4	-0.8	0.7	1.0	1.4	1	1
Q06AB3	PIG Ubiquitin carboxyl-terminal hydrolase isozyme L3	UCHL3	0.3	-0.2	-0.3	0.2	-0.2	-0.2	-1.4	-2.1	1	1
P63279	HUMAN SUMO-conjugating enzyme UBC9	UBE2I	-0.3	-0.3	0.2	0.4	-0.3	-0.1	-0.2	0.6	2	2
P62191	HUMAN 26S protease regulatory subunit 4	PSMC1	0.2	-0.3	0.0	0.2	-0.2	-0.4	-0.7	-0.2	9	4
F1SUE1	PIG Uncharacterized protein	NA	-0.3	0.1	-0.5	0.7	0.1	0.6	1.2	1.6	1	1
K7GS51	PIG Uncharacterized protein (Fragment)	ITGA1	0.8	0.1	-0.4	-0.6	0.9	0.6	0.4	0.3	2	2
P25787	HUMAN Proteasome subunit alpha type-2	PSMA2	0.1	-0.3	0.0	0.1	0.4	0.0	0.4	-0.3	3	3
P07101	HUMAN Tyrosine 3-monooxygenase	TH	-0.4	1.0	-0.4	-0.3	8.0	7.8	0.7	0.6	1	1
F1RX51	PIG Uncharacterized protein	Ssc.3881	0.4	-0.2	-0.2	-0.1	-0.2	-0.3	-1.2	-1.2	2	2
P67776	PIG Serine/threonine-protein phosphatase 2A catalytic subunit alpha isoform	PPP2CA	0.2	-0.3	0.0	0.1	-0.3	-0.2	-0.3	-0.7	6	6
Q13813	HUMAN Spectrin alpha chain, non-erythrocytic 1	SPTAN1	0.3	-0.1	0.1	-0.3	0.3	1.0	-0.4	-1.0	2	3
F1SMZ9	PIG Uncharacterized protein (Fragment)	SF3B1	-0.4	0.0	0.0	0.2	-0.6	-1.2	-1.2	0.4	8	3
B2DCZ9	PIG Rho guanine nucleotide exchange factor 2	ARHGEF2	-0.3	-0.3	-0.1	0.6	-1.2	-0.5	-1.2	-3.1	3	1
I3LSZ6	PIG Uncharacterized protein	RPL28	-0.7	-0.2	0.3	0.6	-0.9	-0.2	-0.5	-0.2	1	1
Q9H361	HUMAN Polyadenylate-binding protein 3	PABPC3	-1.0	-0.1	0.7	0.3	-0.6	-0.4	0.6	1.1	2	1

P35346	HUMAN Somatostatin receptor type 5	SSTR5	0.0	-0.4	0.1	0.2	1.0	2.0	-0.1	-0.1	1	1
K7GLE8	PIG Uncharacterized protein (Fragment)	ALCAM	0.9	0.4	-0.4	-0.9	-0.6	-0.9	-1.4	-2.0	5	3
F1S4Y8	PIG Uncharacterized protein	LOC100621044	-0.1	-0.4	0.2	0.2	-0.3	0.7	1.9	0.7	5	1
F1S1R4	PIG SH3 domain-binding glutamic acid-rich-like protein (Fragment)	SH3BGRL	-0.1	-0.1	0.0	0.0	-0.6	0.1	0.0	-0.5	5	1
O95834	HUMAN Echinoderm microtubule-associated protein-like 2	EML2	0.2	-0.1	0.2	-0.4	0.3	0.1	0.8	-0.4	3	3
F1RT87	PIG Uncharacterized protein	NA	-0.2	-0.7	0.1	0.7	0.2	0.6	0.1	0.6	2	3
Q06A99	PIG SFRS4	SRSF4	-0.2	-0.5	0.3	0.3	-0.3	-0.6	-0.5	0.3	2	1
P60508	HUMAN HERV-FRD	ERVFRD-1	0.4	-0.6	-0.7	0.8	0.6	-0.1	1.5	0.6	1	1
F1SG38	PIG Thioredoxin reductase 1, cytoplasmic	TXNRD1	0.2	0.2	-0.1	-0.4	0.9	0.9	0.3	-0.5	2	3
F1SDP3	PIG Uncharacterized protein	OAT	-0.3	-0.3	0.2	0.3	-0.1	-0.5	-0.1	-1.3	5	3
P31948	HUMAN Stress-induced-phosphoprotein 1	STIP1	-0.4	-0.3	0.1	0.5	1.3	1.6	0.8	-0.2	7	3
Q9N1F5	PIG Glutathione S-transferase omega-1	GSTO1	-0.7	-0.2	0.5	0.3	-1.7	0.2	-0.1	-0.2	8	5
I3LC25	PIG Uncharacterized protein	LMNB2	0.0	0.0	-0.2	0.0	0.6	0.9	-0.3	0.3	14	12
O43464	HUMAN Serine protease HTRA2, mitochondrial	HTRA2	0.3	0.0	-0.3	-0.2	0.5	0.7	-0.6	-1.3	2	1
B9TRW9	PIG Guanine nucleotide-binding protein subunit gamma	GNG12	0.4	-0.7	-0.2	0.4	0.4	0.5	-0.9	-0.5	2	1
F1S530	PIG 60S ribosomal protein L5	RPL5	-1.1	-0.5	0.6	0.9	-1.6	-0.8	0.1	1.2	7	7
P68036	HUMAN Ubiquitin-conjugating enzyme E2 L3	UBE2L3	0.1	-0.5	0.0	0.3	0.8	1.4	0.8	0.7	4	3
F1SS60	PIG Uncharacterized protein	SCO1	0.6	0.0	-0.5	-0.3	-0.4	0.2	-0.4	1.2	3	1
P35908	HUMAN Keratin, type II cytoskeletal 2 epidermal	KRT2	0.0	0.0	-0.2	0.0	0.6	-1.2	2.8	-0.1	10	15
P34930	PIG Heat shock 70 kDa protein 1A	HSPA1A	-0.7	0.2	0.1	0.3	0.1	0.5	0.5	-0.4	24	21
F1RHJ2	PIG Uncharacterized protein	HDGF	-0.3	0.1	0.0	0.1	1.7	2.1	0.0	-0.3	8	2
Q9HCB6	HUMAN Spondin-1	SPON1	-0.4	-0.8	0.0	1.0	-0.3	0.1	-0.3	1.1	6	3
F1RUP2	PIG Uncharacterized protein	GRSF1	0.0	0.4	0.2	-0.7	0.3	-0.2	-0.8	-1.4	1	1
F1SAS3	PIG Uncharacterized protein	LOC100157249	-0.2	0.4	0.1	-0.4	0.2	-0.1	0.1	0.0	9	9
P79303	PIG UTP--glucose-1-phosphate uridylyltransferase	UGP2	-0.2	-0.5	0.1	0.4	1.5	1.1	0.9	1.6	13	3
Q6ZRH3	HUMAN Uncharacterized protein FLJ46360	NA	-0.2	-0.2	0.0	0.3	-0.3	-1.2	-0.7	0.3	1	1
Q2EN79	PIG Ubiquinol-cytochrome c reductase complex	UQCR10	0.7	0.2	-0.2	-0.9	-0.7	-0.2	1.2	1.1	2	1
Q6PUJ7	HUMAN Prohibitin	NA	-0.3	-0.3	0.0	0.4	0.4	0.1	-2.4	-0.9	1	1
F1S0D8	PIG Uncharacterized protein (Fragment)	UBXN4	0.7	-0.3	-0.3	-0.3	0.9	0.4	0.2	1.0	1	2
G9HRP0	HUMAN MHC class I antigen (Fragment)	HLA-A	0.1	-0.8	0.2	0.3	-0.3	-0.6	-1.0	-0.1	1	1
Q8TCU4	HUMAN Alstrom syndrome protein 1	ALMS1	0.0	-0.1	-0.1	-0.1	-2.7	-0.5	0.0	0.1	2	1
Q95242	PIG Platelet endothelial cell adhesion molecule	PECAM1	0.1	0.0	-0.3	0.1	1.0	1.7	1.0	0.8	4	5
I3L6F6	PIG Uncharacterized protein	LOC100624306	-0.2	-0.6	0.4	0.2	-0.5	-0.1	1.9	2.3	1	1
Q9UKX2	HUMAN Myosin-2	MYH2	0.3	0.5	-0.4	-0.6	1.8	-3.1	-4.5	-4.8	1	1
E7EAX3	PIG IFITM3	IFITM3	1.1	0.2	-0.7	-0.7	0.7	0.1	1.3	-0.5	1	1
F1S108	PIG Uncharacterized protein	CISD2	-0.3	-0.1	0.2	0.0	0.0	0.1	0.2	0.7	4	3
P43686	HUMAN 26S protease regulatory subunit 6B	PSMC4	-0.1	-0.5	0.1	0.4	-0.5	-0.7	-0.6	-0.5	7	3
I3LAG7	PIG Uncharacterized protein (Fragment)	NA	-0.4	0.1	0.5	-0.5	-0.1	-0.3	1.2	0.6	2	1
P28839	PIG Cytosol aminopeptidase (Fragment)	LAP3	-0.7	-0.2	0.2	0.5	-0.9	-0.6	1.6	-0.7	2	2
P36542-2	HUMAN Isoform Heart of ATP synthase subunit gamma, mitochondrial	ATP5C1	0.8	0.8	-0.9	-0.9	-0.4	0.9	-3.4	-0.7	1	1

Q29578	PIG 44k ATPase of 70k heat-shock cognate protein (Fragment)	NA	-0.3	-0.4	0.1	0.4	0.3	1.1	0.4	0.5	2	2
Q9P2P6	HUMAN StAR-related lipid transfer protein 9	STARD9	0.6	0.3	-0.7	-0.5	0.3	0.0	1.5	0.0	3	1
F1SCH3	PIG Uncharacterized protein	DDX39A	-0.6	-0.8	0.2	1.0	0.0	-0.1	-0.5	-0.6	8	6
F1RX84	PIG Uncharacterized protein	PROSC	-0.1	-0.4	0.0	0.3	-0.4	-0.7	-0.9	-0.6	4	1
K7GQ66	PIG Uncharacterized protein	RPL8	-1.1	-0.8	0.5	1.2	-1.3	-1.0	-2.0	-0.4	3	1
F1SKI5	PIG Inosine-5'-monophosphate dehydrogenase	IMPDH2	-0.2	-0.3	0.0	0.3	0.1	0.4	0.7	0.6	8	2
I3L7F5	PIG Uncharacterized protein (Fragment)	NA	0.6	-0.4	-0.3	-0.1	0.3	-0.1	-0.1	-0.1	1	1
F1SKI6	PIG Uncharacterized protein	NDUFAF3	0.4	-0.1	-0.4	-0.1	0.0	-0.2	1.2	0.1	1	2
F1S1C3	PIG Uncharacterized protein	CA4	0.0	0.5	-0.3	-0.4	-0.1	-0.2	0.3	0.7	4	4
F1RUZ6	PIG Uncharacterized protein	AIP	-0.4	-0.1	-0.1	0.3	1.4	2.4	-0.1	-0.1	2	1
F1RT21	PIG Uncharacterized protein	NA	-0.3	0.0	-0.4	0.6	-1.1	-0.1	0.1	0.8	2	1
F1RQ90	PIG Uncharacterized protein	RBMX	-0.3	-0.5	0.0	0.7	-0.4	0.1	0.6	1.2	10	7
I3LG37	PIG Uncharacterized protein	LOC100738159	-0.2	0.1	0.3	-0.4	1.9	1.2	0.3	1.2	2	2
P50748	HUMAN Kinetochore-associated protein 1	KNTC1	-0.3	-0.4	0.0	0.4	-1.5	-0.8	2.1	1.9	1	1
I3LN25	PIG Uncharacterized protein	COL15A1	-0.4	-0.3	-0.1	0.7	0.7	0.5	-1.4	-0.7	3	4
B2R8G3	HUMAN Hedgehog protein	NA	0.3	0.5	-0.1	-1.0	-0.8	-0.5	-0.6	0.4	1	1
Q9NNZ3	HUMAN DnaJ homolog subfamily C member 4	DNAJC4	-0.2	-0.4	0.2	0.2	-1.2	-1.0	-0.2	-0.8	1	1
F1SV59	PIG Uncharacterized protein (Fragment)	ADPRHL2	0.3	0.1	-0.2	-0.5	1.0	1.0	0.8	-1.0	3	2
P00636	PIG Fructose-1,6-bisphosphatase 1	FBP1	0.8	-0.1	-0.5	-0.5	0.4	0.8	2.0	1.6	2	1
Q95281	PIG 60S ribosomal protein L29	RPL29	-1.1	-0.9	0.3	1.4	0.2	0.4	1.0	2.3	3	4
I3LNE5	PIG Uncharacterized protein	SNRNP70	-0.2	-0.4	0.0	0.4	-0.5	-0.3	-0.5	-0.6	2	1
F1S0V2	PIG Annexin	LOC100515934	-0.3	-0.3	-0.2	0.5	-0.9	-0.3	-0.2	-0.7	5	4
F1RPY3	PIG Reticulon	RTN3	-0.6	0.0	0.5	-0.1	-0.7	-1.8	0.4	0.5	1	1
I3LRQ0	PIG Uncharacterized protein	NA	0.7	0.9	-0.7	-1.1	2.1	3.1	1.0	-0.6	4	4
F1RNR7	PIG Uncharacterized protein	LOC100525255	-0.4	-0.2	0.1	0.3	-0.2	-0.3	0.2	0.3	5	1
I3LKQ1	PIG Guanine nucleotide-binding protein subunit gamma	GNG7	0.6	-0.1	-0.6	-0.3	0.2	0.7	-0.2	0.0	1	1
I3LCC2	PIG Uncharacterized protein	LOC100516610	-0.6	-0.3	0.3	0.4	0.5	0.6	0.4	1.1	2	2
F1RKW7	PIG Uncharacterized protein	ZNF207	-0.1	-0.1	-0.2	0.1	-0.1	-0.1	-1.0	-0.9	3	1
I3LC21	PIG Uncharacterized protein	APC2	0.5	-0.4	-0.5	0.1	0.6	1.1	3.8	1.7	1	1
I3LHS1	PIG Uncharacterized protein (Fragment)	LOC100624058	0.6	-0.2	-0.1	-0.6	0.1	0.3	-0.5	-0.1	4	5
Q684M6	PIG CDC37 cell division cycle 37 protein	CDC37	-0.1	-0.8	0.2	0.4	0.2	-0.4	-0.2	0.1	8	8
I3LAW0	PIG Uncharacterized protein	C9orf64	-0.2	-0.3	0.3	0.0	-1.4	-1.2	-0.7	0.3	2	1
I3LFJ5	PIG Uncharacterized protein	SUGP2	0.5	0.6	-0.3	-1.1	-1.3	-0.9	-0.1	0.8	1	1
Q9MYY8	PIG Thioredoxin reductase 1, cytoplasmic	TXNRD1	0.2	-0.2	-0.3	0.0	1.6	0.9	1.2	0.2	2	2
I3LDA5	PIG Uncharacterized protein (Fragment)	EHD4	0.2	-0.1	-0.3	-0.1	1.8	2.6	1.2	0.6	12	11
I3LTV6	PIG Cytochrome c oxidase subunit 6A, mitochondrial	COX6A2	0.4	0.0	1.4	-2.1	0.0	-0.3	2.1	-0.4	1	2
F1SKI0	PIG Myosin-11 (Fragment)	MYH11	-0.1	-0.7	0.0	0.5	0.1	0.3	0.5	-0.2	3	3
F1S8S9	PIG Uncharacterized protein	GBF1	0.3	0.1	-0.5	-0.1	-0.5	-0.6	0.0	1.4	2	1
F1SGF1	PIG Uncharacterized protein	PSMD6	-0.5	-0.2	0.1	0.2	-0.1	-0.1	0.2	0.4	3	6
F1S2I8	PIG Uncharacterized protein (Fragment)	TPPP3	-0.4	-0.4	-0.1	0.6	0.5	-0.2	-0.8	-0.8	4	1

A6NJI9	HUMAN Leucine-rich repeat-containing protein 72	LRRC72	-0.3	-0.2	0.1	0.1	-0.5	-1.4	-0.4	0.0	1	1
Q2EN77	PIG Microsomal glutathione S-transferase 3	MGST3	0.6	0.1	-0.2	-0.8	-0.1	0.4	-0.3	-0.3	1	1
Q7L1Q6	HUMAN Basic leucine zipper and W2 domain-containing protein 1	BZW1	-0.6	-0.7	0.2	0.9	-0.5	-0.7	-0.8	-0.7	4	1
F1SMM0	PIG Uncharacterized protein	ERMP1	-0.1	-0.2	0.0	0.0	0.5	-0.6	-0.8	-0.2	5	6
F1RRD7	PIG Uncharacterized protein	CLASP2	0.3	-0.9	0.0	0.3	0.3	-0.7	-0.2	1.4	3	3
I3LIY9	PIG Uncharacterized protein (Fragment)	CCDC180	0.2	0.1	-0.4	-0.2	-1.0	-1.1	0.4	0.1	1	1
Q9NPH2	HUMAN Inositol-3-phosphate synthase 1	ISYNA1	0.0	0.1	-0.4	0.0	0.3	0.0	-0.9	-1.4	1	1
F1RP05	PIG Uncharacterized protein	DNAJA2	-0.3	0.2	-0.1	-0.1	0.0	0.8	0.5	-0.2	7	6
I3LHM5	PIG Uncharacterized protein	USP53	0.0	0.3	0.0	-0.7	-1.0	-1.5	-0.7	0.3	2	1
F1RV20	PIG Uncharacterized protein	GNA13	0.8	0.1	-0.5	-0.7	0.6	0.8	-0.6	-0.1	1	1
F1SMY1	PIG Uncharacterized protein	TMX3	-0.4	-0.4	0.1	0.4	-0.2	-0.5	-1.9	-0.5	7	3
I3LPF1	PIG Uncharacterized protein	LOC100621148	-0.8	-0.5	0.5	0.5	0.0	0.0	-1.5	0.0	1	1
I3LH91	PIG Uncharacterized protein	PALLD	0.0	-0.9	-0.1	0.7	0.1	0.0	0.2	-0.5	3	3
F1RRK8	PIG Uncharacterized protein	ABCC10	-0.2	0.4	0.0	-0.5	0.4	-0.2	0.5	-1.0	1	1
Q6ZMN7	HUMAN PDZ domain-containing RING finger protein 4	PDZRN4	-0.1	-0.2	-0.2	0.2	0.4	0.8	1.2	0.6	1	1
F1STQ1	PIG Uncharacterized protein (Fragment)	SYTL1	1.3	-0.5	-0.7	-0.4	-0.8	-0.6	-0.5	-0.6	2	1
F1S4Y6	PIG Uncharacterized protein	DYNLRB1	-0.3	-0.2	-0.1	0.2	0.9	0.5	0.2	-0.9	2	2
F1RUX1	PIG Coronin	CORO1B	0.2	-0.6	-0.2	0.3	-0.2	0.1	-0.7	-0.9	9	4
F1RWC5	PIG Uncharacterized protein (Fragment)	KIF5B	-0.3	-0.4	0.0	0.3	0.6	0.6	-0.5	0.3	17	12
K7GM47	PIG Uncharacterized protein	MECP2	-0.3	-0.3	-0.2	0.4	1.8	2.1	1.1	2.7	5	7
Q14126	HUMAN Desmoglein-2	DSG2	-1.2	-0.9	0.5	1.3	-1.1	-0.9	0.3	0.2	1	1
F1S0U6	PIG Uncharacterized protein	SETX	1.0	0.8	-1.0	-1.2	-1.1	-0.5	-0.5	1.5	1	1
A4USB5	PIG Cystatin B	NA	0.3	0.3	-0.2	-0.7	-0.6	-1.0	-1.0	-0.2	1	1
Q96MR6	HUMAN WD repeat-containing protein 65	WDR65	1.0	0.2	-0.7	-0.9	0.4	0.5	0.7	0.8	2	1
F1RYK8	PIG Uncharacterized protein (Fragment)	LOC100524000	0.6	0.4	-0.2	-1.1	0.8	1.0	0.1	-1.2	3	2
F1RN28	PIG Uncharacterized protein	PSPC1	0.3	-0.3	-0.3	-0.1	0.1	0.5	-0.6	-1.5	6	4
Q96RQ3	HUMAN Methylcrotonoyl-CoA carboxylase subunit alpha, mitochondrial	MCCC1	0.5	-0.1	-0.4	-0.4	-0.1	-0.5	0.1	0.1	1	1
P09493-6	HUMAN Isoform 6 of Tropomyosin alpha-1 chain	TPM1	-0.5	-0.1	-0.4	0.7	2.8	2.5	2.2	-1.2	4	1
I3LTN6	PIG Uncharacterized protein	PPM1G	-0.3	0.1	0.1	-0.3	0.2	0.6	0.0	1.0	1	1
F1SFF3	PIG Uncharacterized protein (Fragment)	NID2	-0.1	-0.2	-0.2	0.1	0.7	0.2	-1.2	-2.0	26	24
M3VK13	PIG Eukaryotic translation initiation factor 5 tv1	EIF5	-0.4	-0.3	0.1	0.3	0.4	0.3	0.6	0.0	5	1
Q96PU8	HUMAN Protein quaking	QKI	-0.2	0.3	0.1	-0.6	-1.3	-1.0	-0.8	0.7	1	1
F1RRD6	PIG Uncharacterized protein (Fragment)	PDCD6IP	-0.2	-0.4	-0.1	0.4	0.9	1.1	0.3	0.5	20	9
O43837	HUMAN Isocitrate dehydrogenase [NAD] subunit beta, mitochondrial	IDH3B	0.8	0.5	-0.7	-1.0	-1.6	-1.3	-2.4	-1.3	1	1
F1RRW8	PIG Uncharacterized protein	DNM1	0.2	-0.4	-0.3	0.0	0.7	0.0	0.6	-0.4	1	2
F1S3I3	PIG Eukaryotic translation initiation factor 3 subunit G	EIF3G	0.2	-0.9	0.0	0.3	1.6	1.8	0.8	0.4	7	2
M3TYW5	PIG Glutamyl-prolyl-tRNA synthetase	QARS	0.1	-0.8	-0.1	0.4	0.7	1.0	0.1	-0.2	7	5
F1SH25	PIG Uncharacterized protein	RAB21	0.3	-0.5	-0.2	0.0	0.3	0.3	0.6	0.9	2	2
P17560	PIG N-acylglucosamine 2-epimerase	RENBP	0.0	0.2	-0.4	-0.3	0.3	-0.5	0.8	-0.6	1	1
F2VR55	PIG BCL2-associated athanogene 2 (Fragment)	BAG2	0.4	-0.3	-0.2	-0.3	0.3	1.3	1.7	0.8	1	1

F1S2E2	PIG Annexin	ANXA11	-0.1	0.1	-0.1	-0.2	-0.1	0.1	0.4	-0.4	16	14
P03974	PIG Transitional endoplasmic reticulum ATPase	VCP	-0.7	-0.7	0.4	0.6	0.4	1.1	0.8	-0.5	30	22
Q9H0Q0	HUMAN Protein FAM49A	FAM49A	0.5	-0.6	-0.3	0.0	0.5	0.3	0.1	0.0	2	1
P02587	PIG Troponin C, skeletal muscle	TNNC2	-0.7	0.1	-0.1	0.3	1.6	-0.2	0.3	-1.6	1	1
Q15542	HUMAN Transcription initiation factor TFIID subunit 5	TAF5	0.3	0.0	-0.3	-0.4	0.5	0.3	0.1	-0.6	1	1
B2R8Y4	HUMAN cDNA, FLJ94117, highly similar to Homo sapiens actinin, alpha 3 (ACTN3), mRNA	NA	0.5	-0.1	-0.4	-0.5	0.0	0.0	0.4	1.6	1	1
F1SFE3	PIG Uncharacterized protein (Fragment)	LOC100154173	0.5	-0.3	-0.5	-0.1	-0.9	-0.6	-0.9	-0.5	7	6
F1SSX0	PIG Uncharacterized protein	LOC100153771	-0.2	-0.5	-0.1	0.4	0.1	0.2	1.3	1.9	4	5
Q462R2	PIG Integrin beta-1-binding protein 2	ITGB1BP2	-0.4	-0.3	0.2	0.1	0.2	0.5	0.3	-1.5	1	1
F1SAE9	PIG Uncharacterized protein (Fragment)	LAMB1	-0.2	-0.2	-0.1	0.1	-0.8	-0.8	-0.8	0.1	28	23
F1SRB9	PIG Uncharacterized protein	ST13	-0.4	-0.6	0.1	0.4	0.1	0.5	1.1	0.6	10	7
I3LLZ8	PIG Uncharacterized protein (Fragment)	WDR19	0.2	-0.6	-0.1	0.2	-0.2	-1.2	-2.1	-0.9	3	1
A1Z1Q3	HUMAN O-acetyl-ADP-ribose deacetylase MACROD2	MACROD2	-0.5	-0.2	0.6	-0.3	-0.7	-1.5	-0.2	-0.5	1	1
P02540	PIG Desmin	DES	-0.8	-0.5	0.5	0.4	-0.3	-0.3	-0.1	-2.5	51	48
Q5S1U1	PIG Heat shock protein beta-1	HSPB1	-1.0	-0.3	0.3	0.6	0.0	0.9	1.4	0.8	12	15
F1RMA3	PIG Uncharacterized protein	KIAA1967	0.3	-0.6	-0.2	0.1	0.3	0.5	0.8	0.3	5	3
F1RJI1	PIG Uncharacterized protein	MLEC	-0.5	-0.4	0.0	0.4	-0.5	-1.4	0.4	0.4	9	7
F1SLY4	PIG Uncharacterized protein	LMOD2	0.4	0.1	-0.4	-0.6	0.1	1.8	-0.6	-0.3	8	11
I3LSV6	PIG Uncharacterized protein (Fragment)	COL2A1	-0.7	-0.2	-0.1	0.6	0.4	0.3	0.0	0.7	2	1
F1S8G6	PIG Uncharacterized protein	SGTA	-0.8	-0.3	0.2	0.5	0.5	0.4	0.1	-0.9	7	2
I3L801	PIG Uncharacterized protein (Fragment)	PPP1R13L	0.8	-0.1	-0.4	-0.7	1.0	-0.1	-0.6	-0.6	1	2
B7Z2V6	HUMAN cDNA FLJ53889, highly similar to Vacuolar ATP synthase catalytic subunit A	NA	0.4	0.1	-0.5	-0.4	-0.4	0.3	0.3	1.5	1	1
O79876	PIG Cytochrome c oxidase subunit 1	MT-CO1	1.2	-0.3	-0.6	-0.8	-0.8	-0.9	-0.4	-0.6	2	1
F1RFI8	PIG Uncharacterized protein	EWSR1	-1.0	-0.6	0.3	0.8	1.0	1.2	2.3	1.6	3	1
K7GN86	PIG Uncharacterized protein	DLG1	-0.9	-0.2	0.6	0.1	0.8	1.9	1.8	-0.1	1	1
F2Z543	PIG Uncharacterized protein	LOC100524707	-0.1	0.3	-0.1	-0.5	0.2	0.3	-1.0	-0.8	3	2
Q9BW92	HUMAN Threonine--tRNA ligase, mitochondrial	TARS2	0.5	-0.3	-0.6	-0.1	-0.1	-1.0	-0.4	-0.3	1	1
I3LGJ5	PIG Uncharacterized protein	TNKS1BP1	0.0	0.0	0.0	-0.4	0.5	0.8	-1.4	-0.8	1	1
I3L945	PIG Ubiquitin carboxyl-terminal hydrolase (Fragment)	LOC100625317	-0.2	-0.2	0.0	0.0	-0.9	0.0	0.9	-0.1	5	2
F1SVA2	PIG Uncharacterized protein	TINAGL1	0.0	0.0	-0.3	-0.1	-0.2	-0.8	-0.9	-1.2	6	4
F1SN05	PIG Uncharacterized protein (Fragment)	EIF3J	-0.2	-0.5	0.1	0.1	2.0	1.2	0.4	-0.5	4	3
F1RIS6	PIG Uncharacterized protein (Fragment)	MMAB	0.8	-0.3	-0.4	-0.5	0.1	-0.3	-0.3	-0.6	1	1
F1RZM4	PIG Uncharacterized protein (Fragment)	LAMA4	0.0	0.1	-0.3	-0.3	-0.8	-1.3	-1.8	-0.8	23	23
F1SNX3	PIG Uncharacterized protein	LOC100525516	-0.1	-0.2	-0.4	0.2	0.2	0.6	0.5	-0.2	1	1
F1SIF5	PIG Uncharacterized protein	PTPMT1	-0.9	0.2	0.6	-0.4	0.4	-0.1	-1.1	-1.4	1	1
P63241	HUMAN Eukaryotic translation initiation factor 5A-1	EIF5A	-1.2	-0.8	0.6	0.9	0.2	0.6	-1.5	-1.7	7	5
F1SEN2	PIG Glutamate dehydrogenase 1, mitochondrial	GLUD1	-0.1	-0.1	-0.2	-0.2	-0.8	-1.0	-1.4	-1.4	19	17
F1RUK8	PIG Rab GDP dissociation inhibitor beta	GDI2	-0.4	-0.6	0.0	0.5	0.0	-0.3	-0.7	-0.4	2	1
I3LK73	PIG Uncharacterized protein	OTOG	-0.4	-0.3	0.0	0.2	-0.8	-0.5	0.4	1.3	1	2
I3LT90	PIG Uncharacterized protein	MCCC1	0.3	-0.2	-0.1	-0.5	0.3	0.8	-0.4	0.3	3	8

F1RT73	PIG Uncharacterized protein (Fragment)	NA	0.7	0.3	-0.6	-0.8	0.8	0.8	0.1	-0.2	1	1
I3L8P7	PIG Uncharacterized protein	LOC100738149	-0.1	-0.5	-0.1	0.1	0.0	-0.4	0.4	0.0	1	1
I3LAR5	PIG Uncharacterized protein	NACA	-0.2	-0.1	-0.1	-0.1	-0.1	-0.1	0.1	1.2	14	21
I3LH05	PIG Uncharacterized protein	CUX2	-0.1	0.3	-0.1	-0.6	-0.8	0.0	0.1	0.3	1	1
I3LQ32	PIG Uncharacterized protein (Fragment)	CGI-51	0.3	0.0	-0.5	-0.3	-0.4	-0.5	-1.0	-1.4	1	2
I3LH70	PIG Uncharacterized protein	NA	-0.5	-0.3	-0.2	0.5	-0.5	-0.7	0.0	-0.3	5	3
F1RWB7	PIG Uncharacterized protein (Fragment)	NA	-1.3	-1.0	0.5	1.3	0.0	1.9	0.9	4.9	3	2
P43367	PIG Calpain-2 catalytic subunit (Fragment)	CAPN2	-0.3	-0.2	-0.3	0.2	-0.4	0.2	-0.5	-0.7	9	6
Q29235	PIG ATP synthase beta chain, mitochondrial (Fragment)	NA	1.1	0.7	-0.9	-1.3	0.1	-0.7	-1.7	-1.9	1	2
I3L812	PIG Uncharacterized protein	TPP1	-0.2	-0.4	-0.1	0.2	0.1	0.6	0.2	-1.3	1	1
I3L9I9	PIG Uncharacterized protein	RAD51AP2	-0.7	-0.2	0.1	0.4	0.1	-0.2	0.8	0.0	1	1
Q4Z8N7	PIG Plasma platelet-activating factor acetylhydrolase	PAF-AH	-0.2	0.2	-0.1	-0.4	0.2	0.8	2.0	2.0	2	1
I3LQS0	PIG Uncharacterized protein	HNRNPK	-1.0	-0.6	0.3	0.8	0.7	1.0	1.1	-0.9	11	9
I3LBS8	PIG Uncharacterized protein	SYNRG	-0.1	-0.4	-0.3	0.2	0.8	0.1	2.4	3.2	1	1
I3LBN9	PIG Uncharacterized protein (Fragment)	TRA2B	-0.1	-0.3	-0.1	0.1	-0.4	-0.3	0.3	-0.1	5	4
P49923	PIG Lipoprotein lipase	LPL	0.3	0.3	-0.4	-0.8	1.0	0.8	0.6	0.2	1	1
A2A312	HUMAN Forkhead box protein P4 (Fragment)	FOXP4	0.7	0.5	-0.6	-1.2	0.5	0.2	0.3	-1.6	1	1
A6NEL2	HUMAN Ankyrin repeat domain-containing protein SOWAHB	SOWAHB	0.4	0.4	-0.5	-0.8	-0.6	0.3	1.2	1.7	2	1
O60333-3	HUMAN Isoform 3 of Kinesin-like protein KIF1B	KIF1B	-0.1	-0.5	0.1	0.0	-0.7	-1.5	-1.9	-0.2	1	1
I3LPY8	PIG Uncharacterized protein (Fragment)	PANK4	0.2	0.5	-0.5	-0.7	-1.5	-1.9	-2.6	-1.6	2	5
F1ST03	PIG Uncharacterized protein	POGZ	0.0	0.3	-0.3	-0.6	0.5	0.6	0.3	0.4	1	1
Q9Y2I9	HUMAN TBC1 domain family member 30	TBC1D30	0.6	-0.1	-0.4	-0.6	-0.2	-0.6	0.0	-0.2	1	1
F1S2T5	PIG Uncharacterized protein	LTBP2	-1.0	-0.8	0.2	1.0	-0.1	1.8	0.9	3.0	1	1
I3L7Q4	PIG Uncharacterized protein	PRPF6	0.9	0.1	-0.8	-0.7	0.1	2.7	5.3	-0.8	2	1
A6NMZ7	HUMAN Collagen alpha-6(VI) chain	COL6A6	-0.1	0.1	-0.4	-0.1	-1.2	-0.2	-0.5	0.0	6	2
I3LS80	PIG Uncharacterized protein	LOC100739334	-0.3	-0.4	0.0	0.3	-1.6	-1.1	-1.3	-0.3	2	2
F1S260	PIG Uncharacterized protein	LOC100739276	0.6	0.1	-0.6	-0.8	-0.2	-0.6	-0.2	0.2	1	1
F1RTY6	PIG Uncharacterized protein	PPID	-0.7	-0.6	0.1	0.7	1.0	1.2	0.6	0.8	6	3
I3L5D4	PIG Uncharacterized protein	MAP2K2	-0.2	-0.4	0.0	0.2	0.3	0.8	0.9	0.9	4	2
P54098	HUMAN DNA polymerase subunit gamma-1	POLG	0.1	0.0	-0.3	-0.4	-0.6	-0.4	-0.2	0.3	1	1
P15924	HUMAN Desmoplakin	DSP	1.2	-0.1	-0.8	-0.8	-1.7	-1.8	-1.5	-1.0	2	4
F1S393	PIG Uncharacterized protein	CYB5B	0.3	-0.2	-0.5	-0.1	0.8	0.9	1.7	0.8	1	1
F1SBW4	PIG Protein quaking (Fragment)	QKI	0.0	-1.2	0.1	0.6	-0.3	-1.1	-0.4	0.2	4	2
Q9H8H3	HUMAN Methyltransferase-like protein 7A	METTL7A	-0.3	0.0	-0.2	-0.1	-1.4	-1.6	-1.5	0.3	1	1
P62701	HUMAN 40S ribosomal protein S4, X isoform	RPS4X	-1.5	-1.3	0.7	1.5	-0.5	-0.2	0.2	0.8	3	3
F1RZR2	PIG Uncharacterized protein	BAK1	0.1	0.3	-0.3	-0.6	1.2	-0.3	-0.5	0.5	2	1
F1RIE0	PIG Uncharacterized protein	MESDC2	0.0	-0.4	-0.1	0.0	0.9	0.2	0.2	-0.8	3	2
F1RIB4	PIG Uncharacterized protein (Fragment)	LOC100154801	0.0	-0.2	-0.2	-0.3	-0.1	-0.5	-0.7	-0.3	2	2
F1RPY1	PIG Uncharacterized protein (Fragment)	ATL3	-0.8	-0.6	-0.1	0.9	-0.4	-0.1	1.7	2.3	2	1
F1S880	PIG Uncharacterized protein	NSFL1C	-0.1	-0.5	0.0	0.0	0.6	0.4	-0.2	-0.7	11	6

I3LMT3	PIG Uncharacterized protein (Fragment)	RBM14	-0.4	-0.4	0.0	0.2	-1.0	-0.5	0.6	0.6	3	1
I3LLD8	PIG Uncharacterized protein (Fragment)	LOC100626701	0.1	-0.1	-0.3	-0.3	-0.1	-0.4	-1.6	-1.1	9	8
F1S2F4	PIG Uncharacterized protein	COMTD1	-0.2	0.1	-0.4	-0.2	-1.6	-0.8	0.1	0.1	1	2
F1RT88	PIG Uncharacterized protein	CHD7	-0.8	-0.7	1.2	-0.2	0.4	1.0	0.4	0.2	1	1
I3LT81	PIG Uncharacterized protein	RPL17	-1.2	-1.1	0.2	1.4	-0.4	0.6	1.1	1.6	6	7
Q9BXS0	HUMAN Collagen alpha-1(XXV) chain	COL25A1	-0.5	0.2	-0.5	0.2	0.1	1.6	4.8	0.0	1	1
P25786	HUMAN Proteasome subunit alpha type-1	PSMA1	-0.4	-0.8	0.2	0.4	0.9	-0.2	0.3	-0.3	13	5
P79384	PIG Propionyl-CoA carboxylase beta chain, mitochondrial	PCCB	0.4	0.3	-0.5	-0.9	-0.2	-0.2	-0.4	0.0	13	16
Q9H254	HUMAN Spectrin beta chain, non-erythrocytic 4	SPTBN4	0.0	0.0	-0.1	-0.6	-2.0	-1.7	-0.7	0.7	1	2
Q6QAS9	PIG 60S ribosomal protein L7 (Fragment)	NA	-1.3	-1.3	0.5	1.4	-1.6	-0.9	-0.9	1.3	3	3
F1SJR2	PIG Uncharacterized protein	XIRP1	-0.8	-0.2	0.0	0.3	1.6	2.0	0.5	0.2	52	44
I3LTR9	PIG Uncharacterized protein	LOC100739583	0.0	-0.1	-0.3	-0.2	1.1	1.2	1.4	-0.4	7	6
Q3Y5G4	PIG Nebulin-related anchoring protein	NRAP	-0.3	-0.4	0.1	0.1	0.1	0.4	-0.7	-1.2	8	12
F1S0B9	PIG Uncharacterized protein	EIF4E	-1.3	-0.9	0.6	0.9	0.0	1.5	1.4	0.9	3	1
F1RJT3	PIG Uncharacterized protein (Fragment)	DPYSL3	-0.2	-0.6	-0.2	0.3	0.5	0.9	0.5	-0.2	19	14
I3L7B0	PIG Uncharacterized protein (Fragment)	NA	-1.0	-0.6	0.2	0.8	-0.8	-1.2	-1.0	-1.1	2	2
P09917	HUMAN Arachidonate 5-lipoxygenase	ALOX5	0.3	0.3	-0.4	-0.9	-0.8	-1.0	-1.6	0.0	1	1
Q864R5	PIG Mitochondrial Rho GTPase 2	RHOT2	-0.2	-0.3	-0.2	0.1	0.4	0.3	-0.4	-0.4	1	1
I3LE98	PIG Uncharacterized protein	NFU1	-0.3	-0.2	0.1	-0.3	0.2	-0.3	0.0	1.3	2	1
F1S9Z4	PIG Uncharacterized protein	NA	0.8	0.2	-0.7	-0.9	1.9	2.0	1.9	1.7	1	1
Q9UKU7	HUMAN Isobutyryl-CoA dehydrogenase, mitochondrial	ACAD8	0.3	-0.2	-0.4	-0.4	0.0	-0.7	-1.0	-1.6	2	2
F1SRB0	PIG Uncharacterized protein	ZNF501	-0.9	-0.9	0.4	0.7	0.3	1.8	-0.9	0.0	1	1
A9QT41	PIG Inhibitor of kappa light polypeptide gene enhancer in B-cells kinase gamma	IKBKG	0.1	0.0	-0.2	-0.5	-0.4	-0.1	-0.8	-0.2	2	1
F1RZS9	PIG Uncharacterized protein	ZBTB22	-0.8	0.1	0.3	-0.3	0.6	1.2	1.9	-0.4	1	1
I3LSZ4	PIG Uncharacterized protein	LOC100518118	-1.0	-0.8	0.6	0.5	-0.4	-0.1	-0.4	-0.8	1	1
F1RR62	PIG Uncharacterized protein	CCBL1	0.6	-0.1	-0.3	-0.8	0.1	-0.9	-0.6	-0.3	1	2
Q9NZJ0	HUMAN Denticleless protein homolog	DTL	0.0	-0.1	-0.3	-0.2	1.1	1.5	-0.1	0.8	1	1
I3LFC4	PIG Uncharacterized protein (Fragment)	AIMP1	-0.1	-0.6	-0.2	0.3	1.0	0.3	0.1	-0.6	7	2
I3LS73	PIG Uncharacterized protein	TIMM13	0.7	0.3	-0.7	-1.0	1.2	1.3	0.0	-0.5	4	3
Q28960	PIG Carbonyl reductase [NADPH] 1	CBR1	0.7	-0.5	-0.4	-0.5	1.1	0.8	-0.2	-0.7	1	1
F1SGJ4	PIG Uncharacterized protein	ABHD6	0.5	-1.0	-0.1	0.0	2.0	1.2	0.5	1.5	2	1
Q8HZV3	PIG Transferrin receptor protein 1	TFRC	0.1	-0.1	-0.3	-0.4	1.0	0.0	0.5	0.8	16	15
Q6UXZ4	HUMAN Netrin receptor UNC5D	UNC5D	0.2	0.0	-0.4	-0.6	-1.3	-0.8	-1.0	-0.5	1	1
D0G6X4	PIG Farnesyl diphosphate synthase	FDPS	-0.1	-0.1	-0.2	-0.3	-2.1	-1.8	-1.3	0.4	4	4
I3LVM9	PIG Uncharacterized protein (Fragment)	LOC100623403	0.9	-0.3	-0.8	-0.4	-0.7	-1.6	0.0	0.5	1	1
K7GLA7	PIG Pyruvate dehydrogenase E1 component subunit alpha, somatic form, mitochondrial	PDHA1	0.6	1.4	-1.1	-1.6	1.0	1.6	1.8	-1.0	1	1
F2Z4Y1	PIG Uncharacterized protein	YWHAH	-0.5	-0.9	0.1	0.6	-0.1	0.8	0.2	-0.2	13	12
Q5BLZ2	PIG Perilipin-3	PLIN3	-0.4	-0.4	-0.2	0.2	0.8	0.8	1.3	1.0	3	3
I3LJM0	PIG Uncharacterized protein (Fragment)	NA	-0.9	0.0	-0.1	0.4	3.0	1.5	0.3	-2.1	3	1
Q9BXB4	HUMAN Oxysterol-binding protein-related protein 11	OSBPL11	0.6	-0.1	-0.6	-0.6	0.0	-0.3	-0.4	-1.6	1	1

F1SB16	PIG Uncharacterized protein	COPS3	-0.1	-0.4	-0.3	0.1	-0.2	-0.2	-0.1	1.8	4	3
F1RJ74	PIG Uncharacterized protein	SUGT1	-0.5	-0.6	0.1	0.2	1.1	1.1	-0.2	-0.7	4	1
F1SMP1	PIG Uncharacterized protein	LOC100155846	0.7	-0.4	-0.2	-0.8	0.0	-0.6	-0.8	-0.7	1	1
P50750-2	HUMAN Isoform 2 of Cyclin-dependent kinase 9	CDK9	-2.2	-0.8	0.3	2.0	-0.1	1.7	1.0	1.9	2	2
F1SEX0	PIG Uncharacterized protein (Fragment)	DDAH1	-0.8	-0.3	0.3	0.0	0.3	0.2	0.4	0.1	4	2
F1RPQ3	PIG Uncharacterized protein	PSMD14	-0.1	-0.5	-0.1	0.1	-0.3	-0.4	-0.3	-0.8	2	1
Q9BPX1	HUMAN 17-beta-hydroxysteroid dehydrogenase 14	HSD17B14	0.1	-0.3	-0.4	-0.1	0.2	-0.6	-1.4	-0.6	1	1
Q6ZSR9	HUMAN Uncharacterized protein FLJ45252	NA	0.2	0.1	-0.7	-0.4	1.7	1.5	0.0	-0.7	1	1
I3LUR7	PIG Uncharacterized protein	COL6A3	-0.8	-0.9	-0.3	1.3	-1.4	-2.1	-1.9	1.6	88	73
K7GKE2	PIG Uncharacterized protein	MCAM	-0.1	0.1	-0.4	-0.4	-0.1	0.6	0.9	-0.4	8	5
Q29221	PIG F-actin-capping protein subunit alpha-2	CAPZA2	0.0	-0.4	-0.2	-0.1	-0.3	0.3	0.1	-0.3	12	10
F1S971	PIG Uncharacterized protein	CEP170B	0.2	-0.2	-0.2	-0.4	-0.8	-0.2	-0.2	-0.2	3	1
Q8TEU7-2	HUMAN Isoform 2 of Rap guanine nucleotide exchange factor 6	RAPGEF6	-0.3	-0.6	-0.1	0.3	0.5	-1.1	0.5	0.2	1	1
Q52I78	PIG Nicotinamide phosphoribosyltransferase	NAMPT	0.1	-0.5	-0.2	-0.1	1.5	1.2	0.5	0.9	3	2
I3LU72	PIG Uncharacterized protein (Fragment)	LOC100513223	-1.1	-0.1	0.2	0.4	0.1	-0.4	0.0	1.3	1	1
P09497	HUMAN Clathrin light chain B	CLTB	-0.6	-0.2	-0.3	0.4	0.6	0.1	-0.5	-0.6	1	1
F1SKR2	PIG Uncharacterized protein (Fragment)	LOC100153863	-2.3	1.0	1.2	-0.7	-1.8	-1.8	0.4	0.3	1	1
F1SIE0	PIG Uncharacterized protein	MTCH2	0.1	0.2	-0.4	-0.7	-1.1	-1.5	-0.8	-0.2	7	8
P11047	HUMAN Laminin subunit gamma-1	LAMC1	-0.3	-0.4	-0.2	0.1	-0.6	-1.8	-2.0	1.1	2	1
F1RYA4	PIG Uncharacterized protein	NAP1L4	-0.2	-0.2	0.1	-0.4	0.8	0.5	-0.9	-1.9	4	3
F1SID4	PIG Uncharacterized protein (Fragment)	PSMC3	-0.6	-0.1	-0.2	0.2	-1.0	-1.1	-1.5	-1.5	10	3
Q9BUA6	HUMAN Myosin regulatory light chain 10	MYL10	0.6	0.2	-0.6	-0.9	-1.0	-1.6	-3.0	-3.5	1	1
Q3Y4D7	PIG Adiponectin (Fragment)	NA	0.0	0.2	-0.4	-0.5	0.3	0.3	2.1	-0.4	7	6
F1RTY3	PIG Uncharacterized protein	CDNF	0.7	-0.2	-0.4	-0.8	2.8	4.5	1.8	-0.5	1	1
F1RW98	PIG Uncharacterized protein	LOC100626145	-0.3	-0.1	-0.3	0.0	-0.4	-0.2	-0.8	-0.5	5	5
Q3ZDQ5	PIG Caveolin-3	CAV3	-0.3	0.7	-0.3	-0.8	-0.3	0.8	1.2	2.4	2	2
F1RX64	PIG Uncharacterized protein	RBPMS	0.2	-0.5	-0.3	-0.2	1.6	1.5	0.5	0.2	4	3
E3UV40	PIG Extracellular signal-regulated kinase-2	ERK2	-0.4	-1.0	0.1	0.5	-0.2	-0.6	0.9	0.9	1	1
F1SMG1	PIG Uncharacterized protein (Fragment)	MRPS30	0.0	0.1	-0.2	-0.6	0.3	0.2	-0.2	0.2	1	1
I3LVR9	PIG Uncharacterized protein	CMPK2	0.0	-0.3	-0.3	-0.2	0.4	0.5	1.9	1.2	3	2
Q8N1G4	HUMAN Leucine-rich repeat-containing protein 47	LRRC47	-0.1	-0.3	-0.2	-0.1	0.1	-0.4	0.3	-0.6	1	1
B7STY5	PIG Chloride intracellular channel 5	CLIC5	0.0	-0.3	-0.1	-0.2	-0.2	0.0	-0.6	-1.7	5	4
F1SSG6	PIG Uncharacterized protein (Fragment)	ANP32B	-0.3	-0.5	0.1	0.0	-0.5	-1.1	-1.0	-1.0	4	1
F1RVY7	PIG Uncharacterized protein (Fragment)	GALK1	0.1	-0.7	-0.2	0.0	1.2	0.8	0.8	-0.3	7	7
I3L5J9	PIG Uncharacterized protein	YTHDF2	-0.5	-0.8	0.1	0.6	0.2	-0.2	0.3	-0.1	3	1
P26374	HUMAN Rab proteins geranylgeranyltransferase component A 2	CHML	0.4	0.0	-0.6	-0.6	-1.1	-0.1	-0.1	0.0	1	1
Q9H0E2	HUMAN Toll-interacting protein	TOLLIP	-0.3	-0.9	0.0	0.4	-1.1	0.3	-0.2	0.2	1	1
Q16543	HUMAN Hsp90 co-chaperone Cdc37	CDC37	-0.4	0.0	0.0	-0.4	-0.8	-0.6	-0.2	0.4	1	1
I3LTK1	PIG Uncharacterized protein	FXN	0.2	0.4	-0.4	-0.9	0.9	1.3	0.7	-1.3	2	1
F1SQD9	PIG Uncharacterized protein	CRELD1	-0.7	-0.9	0.5	0.3	0.1	0.3	0.6	0.5	1	1

F1S1X4	PIG Ferrochelatase (Fragment)	FECH	0.7	-0.3	-0.8	-0.4	-0.4	-0.3	-0.2	-0.1	5	5
P35749	HUMAN Myosin-11	MYH11	-0.8	-0.5	-0.2	0.7	-0.1	-0.1	1.4	1.1	6	4
I3LET0	PIG Uncharacterized protein (Fragment)	NEDD8	0.0	-0.3	-0.3	-0.2	-0.9	0.4	-7.9	0.0	2	1
Q9BV73	HUMAN Centrosome-associated protein CEP250	CEP250	1.1	-0.5	-0.6	-0.8	0.7	-0.9	-0.3	-0.7	2	1
Q1HL06	PIG 6-phosphofructokinase (Fragment)	PFMK	-1.0	-0.9	0.1	1.0	0.0	-1.1	0.8	0.7	4	3
I3LJT1	PIG Uncharacterized protein	NA	-0.2	-0.2	-0.3	-0.1	0.2	-0.1	-1.4	-1.6	4	3
F1RQI6	PIG Uncharacterized protein (Fragment)	FILIP1	0.5	-0.2	-0.5	-0.5	-0.2	0.6	0.2	0.2	1	3
I3LUM9	PIG Uncharacterized protein	LOC100525970	-0.4	-0.4	-0.1	0.2	-0.5	-0.1	-0.4	-0.9	8	3
F2Z5W4	PIG Uncharacterized protein (Fragment)	LOC100737162	-0.8	-0.7	0.1	0.6	-0.3	-0.6	-0.2	-0.4	1	1
D7RA29	PIG Microtubule-associated protein 1 light chain 3 beta	MAP1LC3B	-0.7	-0.8	0.1	0.7	-0.5	1.2	1.0	-0.1	1	1
I3LFK0	PIG Uncharacterized protein	BCS1L	0.3	-0.1	-0.5	-0.5	-1.5	-1.9	-1.1	-0.4	4	1
B0LY42	PIG CD147	NA	0.4	0.2	-0.6	-0.7	0.5	0.1	-0.4	0.2	3	3
F1SU00	PIG Uncharacterized protein (Fragment)	NA	-0.3	-0.8	-0.3	0.6	0.2	-0.5	-1.0	-0.7	2	2
Q3ZD69	PIG Prelamin-A/C	LMNA	-0.6	-0.5	-0.3	0.5	-0.1	0.4	0.4	0.0	55	46
F1S8P9	PIG Adducin 1 (Alpha)	ADD1	-0.2	-0.3	-0.2	-0.1	-0.1	0.0	0.0	0.0	8	8
Q9Y696	HUMAN Chloride intracellular channel protein 4	CLIC4	-0.1	-0.5	-0.4	0.3	0.7	1.8	0.7	-0.1	4	4
Q29090	PIG Serine/threonine-protein phosphatase 2A 55 kDa regulatory subunit B alpha isoform (Fragment)	PPP2R2A	-0.2	-0.5	-0.2	0.1	0.4	0.0	-0.1	-0.3	8	4
O00189	HUMAN AP-4 complex subunit mu-1	AP4M1	-0.3	-0.3	-0.3	0.1	-8.8	-13.8	-15.4	-17.1	1	1
F1RZV1	PIG Uncharacterized protein	LOC100522554	0.3	-0.3	-0.5	-0.3	1.1	1.1	0.6	-0.6	5	2
O75208	HUMAN Ubiquinone biosynthesis protein COQ9, mitochondrial	COQ9	-0.2	0.1	-0.4	-0.3	0.1	-0.9	-1.5	0.5	2	1
F1RKH4	PIG Uncharacterized protein	HSPB8	-0.2	0.0	-0.4	-0.2	0.8	0.7	1.0	-0.6	6	3
F1SNC6	PIG Uncharacterized protein (Fragment)	KIAA0368	-0.3	-0.6	-0.1	0.2	-1.5	-1.3	-1.1	-2.3	2	1
F1SA22	PIG Uncharacterized protein	ZFYVE26	-0.2	0.3	-0.4	-0.5	2.0	0.6	-0.9	-1.2	1	1
A0SNV3	PIG Tyrosine 3-monooxygenase/tryptophan 5-monooxygenase activation protein zeta polypeptide (Fragment)	NA	-0.9	-0.9	0.2	0.7	-1.7	-2.2	-1.4	-1.6	7	6
I3LDT4	PIG Uncharacterized protein	NA	-0.1	-0.4	0.0	-0.3	0.3	-0.5	0.1	-1.1	2	4
I3LK09	PIG Uncharacterized protein	MRPS27	0.7	-0.8	-0.6	-0.2	0.1	0.1	-0.8	-0.3	1	3
P04574	PIG Calpain small subunit 1	CAPNS1	-0.5	-0.6	0.0	0.3	0.6	0.8	0.3	-0.8	9	6
A7UIU7	PIG ATP citrate lyase	ACL	-0.7	-0.3	0.0	0.2	0.6	-0.3	-1.0	0.4	6	2
Q9UMS0	HUMAN NFU1 iron-sulfur cluster scaffold homolog, mitochondrial	NFU1	0.5	-0.4	-0.5	-0.4	2.1	1.5	-0.4	-1.8	2	1
Q86U42	HUMAN Polyadenylate-binding protein 2	PABPN1	-0.5	-0.6	-0.2	0.4	0.5	-0.4	-0.3	-0.3	2	1
H9LB34	PIG Protein phosphatase 1B magnesium-dependent beta isoform (Fragment)	PPM1B	-0.1	-0.4	-0.1	-0.2	0.4	0.5	-0.6	-1.3	2	3
Q9Y512	HUMAN Sorting and assembly machinery component 50 homolog	SAMM50	-0.1	-0.1	-0.2	-0.5	-0.1	-0.2	0.6	0.7	4	5
F1SGG2	PIG Uncharacterized protein	KRT8	-0.5	-0.4	0.0	0.1	0.6	0.1	1.6	4.4	5	2
F1S2Z4	PIG Uncharacterized protein	LOC100525900	-0.1	-0.1	-0.4	-0.3	-0.3	-0.2	-0.4	-0.6	15	15
P25789	HUMAN Proteasome subunit alpha type-4	PSMA4	-0.2	-0.6	-0.2	0.1	0.6	0.1	-0.1	-0.2	1	1
Q6NSX1	HUMAN Coiled-coil domain-containing protein 70	CCDC70	-0.4	-0.1	-0.5	0.1	-0.3	1.0	0.4	-0.3	1	1
Q9NVI7	HUMAN ATPase family AAA domain-containing protein 3A	ATAD3A	-0.4	0.0	-0.2	-0.3	0.0	0.5	0.7	1.2	1	1
Q710C4	PIG Adenosylhomocysteinase	AHCY	-0.5	-1.0	0.1	0.6	0.2	-0.3	1.3	-0.2	10	10
F1SPT5	PIG Uncharacterized protein	LAMB2	0.0	-0.2	-0.3	-0.4	-0.5	-1.0	-1.8	-0.9	25	25

F1SIX3	PIG UTP--glucose-1-phosphate uridylyltransferase (Fragment)	UGP2	-0.4	-0.4	-0.3	0.3	-0.7	0.0	-0.7	-0.4	1	1
Q95N02	PIG Cysteinyl leukotriene receptor 1	CYSLTR1	0.1	0.3	-0.5	-0.8	0.4	0.4	0.5	0.1	1	1
Q9BUH8	HUMAN Brain-enriched guanylate kinase-associated protein	BEGAIN	0.8	0.2	-0.8	-1.2	-0.3	0.3	-1.2	-1.3	2	1
Q5Y2C6	PIG Transcription factor TZP (Fragment)	NA	-0.8	-1.0	0.1	0.8	-0.8	-1.6	0.5	1.6	1	1
I3LCW3	PIG Uncharacterized protein	SRRM2	0.1	-0.4	-0.3	-0.3	0.8	1.4	1.6	2.8	4	1
Q6SA96	PIG 40S ribosomal protein S23	RPS23	-1.3	-1.0	0.2	1.3	-1.6	-1.1	0.1	0.5	6	6
F1RPT3	PIG Uncharacterized protein (Fragment)	TANC1	0.1	-0.1	-0.3	-0.6	-0.8	0.9	0.1	0.9	1	2
F1SFJ7	PIG Uncharacterized protein	LYRM5	0.1	0.2	-0.4	-0.8	-1.3	-1.2	-1.2	-0.9	1	2
F4MHC4	HUMAN Ubiquitously transcribed tetratricopeptide repeat protein Y-linked transcript variant 214	UTY	0.5	0.6	-0.9	-1.1	0.3	0.6	0.6	-0.8	1	1
O79881	PIG NADH-ubiquinone oxidoreductase chain 4	MT-ND4	0.0	0.2	-0.5	-0.6	-0.3	0.6	-1.0	-0.5	5	5
F1S3W0	PIG Cytochrome b-c1 complex subunit 6	UQCRH	0.3	1.4	-0.8	-1.8	3.3	1.9	-0.1	-3.1	4	4
I3LHK5	PIG Uncharacterized protein	LOC100620619	0.0	0.0	-0.6	-0.4	1.4	2.8	1.3	-0.8	1	1
O19066	PIG Ovarian sterol carrier protein 2 (Fragment)	SCP2	-0.6	-0.3	-0.1	0.2	0.1	0.0	-0.6	0.6	4	3
Q562R1	HUMAN Beta-actin-like protein 2	ACTBL2	0.5	0.0	-0.6	-0.8	-0.7	-0.8	-1.3	-0.2	5	3
F5H1B2	HUMAN tRNA pseudouridine synthase A, mitochondrial (Fragment)	PUS1	0.2	0.4	-0.3	-1.2	-0.3	-0.6	-1.3	-0.7	1	1
I3L5B9	PIG Uncharacterized protein	LOC100624781	0.0	-0.4	-0.2	-0.3	0.1	-0.7	-0.5	-0.6	1	5
F1RK18	PIG Uncharacterized protein	CAB39L	-0.7	-0.3	0.0	0.0	0.6	0.3	-0.6	-1.2	2	1
P11055	HUMAN Myosin-3	MYH3	0.6	-0.2	-0.7	-0.7	-0.9	-1.2	-0.8	-0.7	5	6
P49756	HUMAN RNA-binding protein 25	RBM25	-0.5	-0.1	-0.3	-0.1	0.5	2.2	0.7	-1.7	3	1
Q96RL7	HUMAN Vacuolar protein sorting-associated protein 13A	VPS13A	-1.3	0.1	0.5	-0.2	0.1	-0.3	1.0	-0.2	1	1
I3LEB7	PIG Uncharacterized protein (Fragment)	PODN	-0.7	-0.7	-0.2	0.7	-1.0	2.7	-1.6	-0.4	1	2
I3LB11	PIG Uncharacterized protein	CPNE8	0.0	-0.4	-0.5	-0.2	0.2	0.2	-0.4	-0.8	1	1
F1S232	PIG 4-trimethylaminobutyraldehyde dehydrogenase	ALDH9A1	-0.4	-0.6	-0.1	0.1	-0.6	-1.0	-0.8	-1.1	11	10
F1RUP0	PIG Uncharacterized protein (Fragment)	ECHDC3	0.2	-0.8	0.0	-0.4	-0.7	0.0	0.3	0.1	1	2
F1S3J0	PIG Uncharacterized protein	ACOT6	0.7	0.3	-0.7	-1.3	-0.3	-0.3	0.6	-0.7	3	3
F1RXD5	PIG Uncharacterized protein	CPNE3	-0.3	-0.5	-0.2	0.0	0.1	-0.1	0.2	0.4	4	5
I3LHC8	PIG Uncharacterized protein	LOC100522692	0.2	-0.3	-0.2	-0.6	-0.2	-0.6	-0.5	-1.3	4	6
Q9GLE9	PIG Heat shock 90kD protein 1, beta (Fragment)	HSPCB	0.2	-1.9	0.1	0.6	-0.5	0.6	-0.1	-0.8	2	3
P13489	HUMAN Ribonuclease inhibitor	RNH1	-0.3	-0.7	-0.1	0.1	1.3	0.9	0.2	0.1	1	1
P51888	HUMAN Prolargin	PRELP	-0.2	-0.6	-0.3	0.1	-0.1	0.8	0.4	0.5	1	1
P63220	HUMAN 40S ribosomal protein S21	RPS21	0.1	0.0	-0.4	-0.7	-0.3	-1.5	-1.4	0.2	1	1
I3L8K2	PIG Uncharacterized protein	NA	-0.4	-0.1	-0.1	-0.4	-0.6	0.0	-0.6	-0.7	4	9
Q8NFS9	HUMAN N-acetyllactosaminide beta-1,6-N-acetylglucosaminyl-transferase, isoform C	GCNT2	-0.9	-1.0	-0.3	1.2	-0.9	-1.2	-0.6	0.5	1	1
K7GMI6	PIG Uncharacterized protein	TBKBP1	0.7	0.2	-0.8	-1.0	-0.1	0.4	0.0	0.1	1	1
F1SR21	PIG Uncharacterized protein	LOC100516708	0.2	-0.6	-0.4	-0.2	0.8	-0.7	-1.5	-0.1	2	2
F1RLE5	PIG Uncharacterized protein	TMED7	-0.6	0.1	-0.2	-0.3	0.5	-0.7	-0.3	0.6	3	1
F1S1F3	PIG Uncharacterized protein	COA3	0.1	0.2	-0.4	-0.9	-0.6	-0.5	-0.9	-0.1	3	3
F1RF44	PIG Uncharacterized protein	LOC100739542	0.6	0.2	-0.8	-1.0	-0.9	-0.8	-1.7	-1.3	2	3
F1SFQ0	PIG Uncharacterized protein	UBA3	0.4	-0.7	-0.5	-0.1	1.2	0.9	0.9	-0.8	1	2
P14543	HUMAN Nidogen-1	NID1	-0.1	-0.5	-0.4	0.0	-0.1	-1.2	0.2	0.1	4	3

F1RSH0	PIG Uncharacterized protein	MRPL15	0.5	-0.3	-0.5	-0.7	1.6	1.8	-0.3	-0.3	2	3
F1RIJ5	PIG Uncharacterized protein (Fragment)	DNAJC11	-0.1	-0.3	-0.3	-0.3	0.4	-0.7	-0.4	0.0	3	4
F1SHX0	PIG Uncharacterized protein (Fragment)	NEB	-1.0	-0.3	0.3	0.0	-0.8	-0.8	-1.1	-0.6	1	1
F1SIQ5	PIG Uncharacterized protein	GPC1	-0.5	-0.3	0.2	-0.5	-0.9	-0.9	-0.8	-1.2	3	1
O75417	HUMAN DNA polymerase theta	POLQ	0.4	0.3	-0.9	-0.8	2.2	2.2	-0.6	-2.9	2	2
F1SAM8	PIG Uncharacterized protein (Fragment)	CDC42BPB	0.2	-0.2	-0.5	-0.5	0.4	-0.6	-1.2	-0.9	1	1
L7UVK8	PIG MHC class I alpha chain (Fragment)	SLA-2	-0.1	0.0	-0.4	-0.5	-0.2	-1.2	-0.8	-1.3	1	1
B8XY19	PIG Acyl-CoA synthetase short-chain family member 2	ACSS2	0.6	-0.5	-0.5	-0.6	-0.3	0.1	-1.3	-1.2	5	6
K9IVF7	PIG X-prolyl aminopeptidase (Aminopeptidase P) 1, soluble	XPNPEP1	0.0	-0.3	-0.3	-0.4	1.1	1.9	0.3	-0.7	7	6
K7GMY3	PIG Uncharacterized protein (Fragment)	HSD17B10	0.2	-0.6	-0.4	-0.1	0.7	-0.2	0.2	-0.2	3	3
F1SV22	PIG Uncharacterized protein	MACF1	-0.6	-1.1	0.0	0.7	-1.3	-1.3	-1.6	0.0	9	5
I3LCG5	PIG Uncharacterized protein	PLBD1	-0.7	-0.2	0.1	-0.2	0.6	0.6	0.9	0.5	1	1
I3L567	PIG Uncharacterized protein	NA	-0.7	0.0	-0.4	0.1	-0.5	-0.6	0.1	-0.2	1	1
I3LJE2	PIG Uncharacterized protein	DPYSL2	-0.2	-0.5	-0.4	0.1	0.5	0.6	0.9	-0.6	19	16
B9V4E8	PIG ADP-ribosylation factor 2	ARF2	-0.6	-0.4	-0.2	0.2	0.0	0.7	-0.3	-0.8	2	2
Q6YN16	HUMAN Hydroxysteroid dehydrogenase-like protein 2	HSDL2	0.7	-0.5	-0.8	-0.5	0.1	0.2	0.6	1.0	1	1
A7YB26	PIG Peroxisomal enoyl coenzyme A hydratase 1 (Fragment)	ECH1	0.7	0.0	-0.8	-0.9	-0.6	-1.3	-1.2	-0.8	1	1
I3LDB1	PIG Uncharacterized protein (Fragment)	NA	0.0	-0.2	-0.3	-0.5	-0.8	-0.9	-0.1	-0.5	1	1
Q8WNQ7	PIG N-acetylgalactosamine-6-sulfatase	GALNS	-0.2	-0.4	-0.5	0.0	-0.5	-1.1	0.9	-0.7	3	1
F1SU03	PIG Uncharacterized protein	HSPG2	-0.1	-0.6	-0.3	0.0	-0.8	-1.9	-1.9	-1.3	19	16
F1S3V0	PIG Uncharacterized protein	MAST2	1.0	0.3	-1.0	-1.4	-1.1	-2.2	-2.6	-0.7	2	2
F1RNX2	PIG Programmed cell death protein 5	PDCD5	-0.1	-0.4	-0.2	-0.2	1.4	1.4	-0.4	-1.7	4	5
B3KS36	HUMAN cDNA FLJ35376 highly similar to Homo sapiens ribosomal protein L3 (RPL3)	NA	-0.3	-0.4	-0.4	0.0	1.0	2.5	0.5	0.3	1	1
I3LHT9	PIG Uncharacterized protein	STX12	-0.2	0.0	-0.4	-0.4	1.0	0.8	0.1	-0.1	3	1
F1SSR1	PIG Uncharacterized protein	C15orf52	0.3	-1.2	-0.1	0.0	0.8	1.0	1.8	1.9	1	1
F1RI49	PIG Uncharacterized protein (Fragment)	AP5Z1	0.5	0.0	-0.7	-0.8	-0.2	-0.8	-0.1	0.6	1	1
D2KPI8	PIG Adenylosuccinate lyase	ADSL	-0.2	-0.6	0.0	-0.2	-0.9	-1.5	-0.6	-0.4	2	3
I3LLT5	PIG Uncharacterized protein	SUOX	0.2	-0.8	-0.4	-0.1	1.2	0.5	-0.1	-0.1	2	2
D4PEM8	PIG N-Myc downstream regulated gene 2	NDRG2	0.4	-0.1	-0.7	-0.7	0.4	0.8	-0.2	-1.2	1	1
I3L662	PIG Uncharacterized protein	LOC100622542	0.3	0.3	-0.8	-0.9	0.2	-0.3	-1.4	-1.3	1	1
I3LUL5	PIG Uncharacterized protein (Fragment)	LOC100624689	-1.4	-0.5	-0.1	0.8	-0.9	-0.8	-0.8	1.5	6	4
F1RRF2	PIG Uncharacterized protein	STT3B	-0.9	-0.9	0.0	0.7	-1.0	-1.1	0.5	0.1	2	2
I3LTZ5	PIG Uncharacterized protein	LOC100624938	-0.4	-0.4	-0.1	-0.1	-0.9	-0.9	-1.4	-0.6	1	1
Q8IX12	HUMAN Cell division cycle and apoptosis regulator protein 1	CCAR1	-2.5	-1.5	1.2	1.7	-2.1	-2.0	0.7	1.8	2	2
M3TYK5	PIG ATPase, Ca++ transporting, plasma membrane 4 tv2	ATP2B4	-0.4	-0.6	-0.5	0.4	0.1	-0.2	-0.1	-0.2	5	3
P23588	HUMAN Eukaryotic translation initiation factor 4B	EIF4B	-1.0	-0.3	0.0	0.2	1.8	1.8	0.9	1.0	3	2
I3LDU9	PIG Uncharacterized protein	SNTB2	-0.6	0.2	-0.7	-0.1	-0.3	-0.6	-0.9	-0.9	3	3
I3LDQ1	PIG Uncharacterized protein	NA	-0.5	-0.8	-0.2	0.4	0.8	-0.2	-0.6	0.2	4	5
Q96DH6	HUMAN RNA-binding protein Musashi homolog 2	MSI2	-0.1	0.1	-0.6	-0.5	1.0	0.7	-0.8	0.2	1	1
Q86VF7	HUMAN Nebulin-related-anchoring protein	NRAP	-0.7	-0.3	0.0	-0.1	0.0	0.7	-0.3	0.8	3	2

K7GMH0	PIG Uncharacterized protein (Fragment)	MYH6	1.9	-0.6	-1.2	-1.2	3.9	0.9	-0.4	2.0	13	12
F1SAH1	PIG Uncharacterized protein	NLRX1	0.3	0.0	-0.6	-0.8	-0.2	-1.2	-1.2	-1.7	3	10
F1S8M9	PIG Uncharacterized protein	LRPAP1	-1.0	-0.2	0.0	0.0	-0.5	-0.9	-0.9	-0.6	7	7
P00172	PIG Cytochrome b5	CYB5A	-0.7	0.1	-0.1	-0.4	0.0	0.4	-0.6	-1.1	5	5
Q9Y625	HUMAN Glypican-6	GPC6	-0.1	-0.2	-0.4	-0.3	-0.7	-1.1	-1.1	-1.0	1	1
F1SVD5	PIG Uncharacterized protein (Fragment)	SMYD1	-0.2	-0.6	-0.5	0.1	-0.5	-0.9	0.4	1.2	1	1
F1S361	PIG Uncharacterized protein	DSTYK	-0.4	-0.2	-0.2	-0.3	0.2	-0.1	2.0	0.2	2	1
F1S9C9	PIG Proteasome subunit beta type	LOC100155139	-0.3	-1.1	-0.4	0.7	0.4	-0.5	0.8	0.1	4	6
A5A774	PIG Hermansky-Pudlak syndrome 5 protein	HPS5	0.1	0.6	-1.0	-0.7	0.0	-0.6	-0.6	-0.1	1	1
I3LTV9	PIG Uncharacterized protein	LOC100623269	0.4	-0.8	-0.4	-0.3	-1.1	-1.0	-0.8	1.2	2	1
F1SN53	PIG Uncharacterized protein	C2orf88	0.3	-0.3	-0.6	-0.6	-0.7	-1.0	-1.9	-0.7	4	3
I3LEG0	PIG Uncharacterized protein (Fragment)	FARP1	-0.3	-1.0	-0.2	0.3	-1.5	-1.6	-1.4	0.2	2	1
Q8N612	HUMAN FTS and Hook-interacting protein	FAM160A2	0.4	-0.3	-0.7	-0.6	-0.7	0.2	0.7	0.2	1	1
O02840	PIG Cadherin-5	CDH5	-0.5	-0.1	-0.3	-0.2	-1.5	-1.1	-1.7	-1.5	4	1
F1SF87	PIG Uncharacterized protein (Fragment)	SLC25A13	-0.2	-0.2	-0.4	-0.4	-1.1	-1.0	-1.1	-0.5	10	10
K7GPJ4	PIG Uncharacterized protein	ACOT9	0.2	-0.1	-0.5	-0.7	0.4	0.1	-0.7	-0.2	14	15
I3L9N5	PIG Uncharacterized protein	LOC100626266	-0.4	-0.5	-0.2	-0.1	1.0	0.8	-0.7	0.3	2	1
Q15185	HUMAN Prostaglandin E synthase 3	PTGES3	-0.9	-0.9	0.2	0.5	-0.3	-0.1	-1.5	-1.7	2	1
I3LVS3	PIG Uncharacterized protein	LOC100627810	-0.1	-0.4	-0.4	-0.3	0.1	0.7	-0.2	0.2	2	3
A1XQU9	PIG 40S ribosomal protein S20	RPS20	-0.6	-0.9	-0.1	0.4	-0.3	-0.4	-0.6	-0.6	3	3
P62258	HUMAN 14-3-3 protein epsilon	YWHAE	0.2	-0.8	-0.5	-0.1	0.8	0.6	0.5	0.0	5	4
I3LDH3	PIG Uncharacterized protein	PARP1	-0.3	-0.6	-0.3	0.0	0.6	0.6	0.5	1.3	8	7
Q29175	PIG Zinc transporter SLC39A7 (Fragments)	SLC39A7	-1.2	0.0	-0.2	0.2	-0.7	0.0	-0.4	0.8	1	2
I3LSP1	PIG Methionine aminopeptidase 2	METAP2	-0.9	-0.9	0.0	0.7	0.0	-1.1	-0.2	1.3	1	1
F1RHG1	PIG Clustered mitochondria protein homolog	CLUH	0.0	-0.6	-0.5	-0.1	0.8	0.1	0.0	-0.7	2	8
F1SGV7	PIG Uncharacterized protein	TTC3	0.0	-0.1	-0.5	-0.5	-1.1	-1.8	-1.7	0.0	1	1
I3LFQ0	PIG Uncharacterized protein	HNRNPH3	-0.4	-0.5	-0.4	0.1	0.0	0.9	-0.1	-0.2	3	3
Q4G0P3	HUMAN Hydrocephalus-inducing protein homolog	HYDIN	-0.1	-0.5	-0.3	-0.3	-3.8	-4.2	-1.9	-0.5	1	1
I3L9B6	PIG PDZ and LIM domain protein 3	PDLIM3	-0.4	-0.6	-0.3	0.1	0.1	-0.2	0.2	-0.4	1	1
I3LT97	PIG Uncharacterized protein	LOC100627253	-1.1	-0.9	0.2	0.5	-0.5	0.3	0.6	0.6	2	1
B8Q0B3	PIG Popeye domain containing protein 2 variant 1	NA	0.5	0.0	-0.6	-1.0	0.3	0.4	-0.3	-0.5	3	3
Q13126	HUMAN S-methyl-5'-thioadenosine phosphorylase	MTAP	0.4	-0.5	-0.6	-0.5	-1.4	-2.1	-1.4	-3.5	1	1
F1RW03	PIG Uncharacterized protein	MRPL38	-0.1	-0.3	-0.3	-0.5	0.4	0.7	0.1	0.3	1	1
P58546	HUMAN Myotrophin	MTPN	-1.2	-0.5	0.1	0.5	0.8	0.1	-0.7	0.2	1	1
Q7Z3B3	HUMAN KAT8 regulatory NSL complex subunit 1	KANSL1	0.2	0.0	-0.4	-1.1	1.5	1.9	1.0	0.6	1	1
F1STC7	PIG Uncharacterized protein (Fragment)	CNNM3	-2.2	-0.7	0.8	1.0	-0.5	-0.9	-0.5	-0.4	1	1
A6N8P5	PIG Multiprotein bridging factor 1	MBF1	-0.9	-0.6	0.1	0.3	0.2	0.2	-1.3	0.6	1	1
F1SGG6	PIG Uncharacterized protein (Fragment)	KRT5	-0.4	-0.2	-0.1	-0.4	-1.2	-1.9	-2.7	6.2	1	1
F1RFC8	PIG Uncharacterized protein	MTFP1	0.5	0.3	-0.7	-1.3	1.2	1.3	0.0	-0.4	4	2
F1RLK1	PIG Olfactory receptor	LOC100517239	-0.3	-0.7	-0.3	0.2	-0.9	-0.8	0.0	-0.1	1	1

A2SZV5	PIG Tax1 binding protein 3 (Fragment)	NA	-0.2	-0.6	-0.4	0.0	-0.5	-0.4	-0.5	-1.4	3	3
F1SDJ8	PIG Uncharacterized protein	GLRX3	-1.0	-0.2	0.1	0.0	-0.7	-0.7	0.2	-0.8	4	3
Q13683	HUMAN Integrin alpha-7	ITGA7	-0.3	-0.6	-0.2	-0.1	-0.8	-0.9	-0.6	-0.7	5	5
F1RR89	PIG Uncharacterized protein (Fragment)	RARS	-0.8	-0.8	0.0	0.5	0.5	1.1	1.5	0.8	6	2
F1SU06	PIG Uncharacterized protein	HP1BP3	-0.5	-0.1	-0.4	-0.2	-0.9	0.1	0.6	0.7	9	9
F1SHR2	PIG Uncharacterized protein	BCL2L13	0.3	-0.4	-0.6	-0.6	1.3	1.0	0.3	0.4	4	5
Q15149	HUMAN Plectin	PLEC	-0.8	-0.9	0.1	0.4	-0.9	-1.5	-1.3	-0.4	18	19
F1RVA3	PIG Uncharacterized protein (Fragment)	LOC100623668	-0.3	0.0	-0.5	-0.4	-0.4	0.1	-0.4	0.2	5	6
F1S441	PIG Uncharacterized protein	CANX	-1.5	-0.4	0.2	0.6	-1.4	-0.7	-0.6	0.9	21	22
I3LCI1	PIG Uncharacterized protein	TRIP6	-0.4	-0.8	-0.4	0.4	-0.2	0.2	0.1	-0.2	1	1
Q6XGY2	PIG 2,4-dienoyl-CoA reductase (Fragment)	DECR	0.4	-0.3	-0.6	-0.7	0.3	0.4	-1.2	-0.2	1	1
Q29236	PIG T-complex protein 1 subunit zeta (Fragment)	CCT6	-0.8	-0.2	0.0	-0.2	0.2	0.0	0.2	-0.1	2	1
Q96A33	HUMAN Coiled-coil domain-containing protein 47	CCDC47	-0.9	-0.3	-0.1	0.1	-0.8	-0.6	-0.9	0.1	1	1
P68366	HUMAN Tubulin alpha-4A chain	TUBA4A	0.1	0.2	-0.4	-1.0	1.2	0.3	-2.9	-1.9	2	2
F1SBY5	PIG Uncharacterized protein (Fragment)	MLLT4	-0.6	-0.7	-0.5	0.6	-1.4	1.6	-1.5	0.4	2	3
Q15404	HUMAN Ras suppressor protein 1	RSU1	-0.6	-0.8	-0.3	0.5	-1.0	-0.2	-0.2	0.1	2	3
F1RY11	PIG Uncharacterized protein	CCDC93	0.1	-0.1	-0.6	-0.6	0.9	2.0	-0.8	-0.6	2	2
Q8WXB1	HUMAN Protein N-lysine methyltransferase METTL21A	METTL21A	-0.5	-0.6	0.1	-0.2	-1.4	-1.6	-0.5	-0.4	1	1
I3LAQ1	PIG Histone H2B	HIST2H2BF	-1.8	-1.1	0.3	1.5	-2.2	-0.5	0.8	0.3	2	2
I3LRE5	PIG Uncharacterized protein	NA	0.1	-0.7	-0.3	-0.2	0.5	1.0	-1.5	-0.8	1	1
A6YNL5	PIG Calpain 2	Capn2	-0.5	-0.7	-0.3	0.3	-1.2	-0.6	-0.2	0.8	7	7
Q8NHU6	HUMAN Tudor domain-containing protein 7	TDRD7	0.2	-0.1	-0.6	-0.7	-0.4	-0.7	-0.5	-0.3	1	1
F1SBU5	PIG tRNA-dihydrouridine(47) synthase [NAD(P)(+)]	LOC100513458	0.6	-0.3	-0.8	-0.7	0.1	-0.4	0.2	1.0	1	1
D5KJI2	PIG High mobility group AT-hook protein 1	HMGA1	-0.5	-0.2	-0.3	-0.1	0.6	0.5	0.8	2.5	1	1
Q29323	PIG Ribosomal protein S5 (Fragment)	NA	-1.1	-1.1	0.2	0.7	-0.2	-0.1	1.1	0.1	1	1
I3LG34	PIG Uncharacterized protein	RB1CC1	-0.2	-0.7	-0.4	0.1	0.2	0.0	-1.1	-2.0	1	1
F1SA50	PIG Uncharacterized protein (Fragment)	HNRNPM	-0.7	-0.9	0.0	0.4	0.1	0.7	0.6	0.4	25	16
F1S8S4	PIG Uncharacterized protein (Fragment)	ENAH	-1.7	-1.0	0.3	1.2	-0.1	0.4	2.2	1.4	3	1
F1SP46	PIG Uncharacterized protein	NA	-1.2	-1.1	0.1	0.9	-0.8	-0.8	0.0	0.1	6	6
Q2TAL5	HUMAN Smoothelin-like protein 2	SMTNL2	-0.3	-0.5	-0.5	0.0	-0.7	-0.6	-0.8	0.0	1	1
I3LS35	PIG Uncharacterized protein (Fragment)	LPCAT3	-0.2	-0.2	-0.4	-0.5	1.1	0.2	0.0	0.4	1	1
I3LGF7	PIG Uncharacterized protein (Fragment)	LOC100525988	0.6	-0.6	-0.7	-0.6	0.3	-0.3	-0.2	1.3	3	3
A5GHK3	PIG Prefoldin 4	PFDN4	0.2	-0.3	-0.7	-0.6	1.0	-0.2	-1.8	-0.2	3	2
F1RGJ2	PIG Uncharacterized protein	CTNNA1	-0.5	-0.8	-0.1	0.2	0.1	0.5	0.4	1.5	20	20
F1RPK1	PIG Uncharacterized protein	LOC100738677	0.4	0.1	-0.8	-0.9	0.4	1.6	1.3	3.6	1	1
I3L621	PIG Uncharacterized protein (Fragment)	AGAP3	-0.1	0.1	-0.5	-0.7	-1.2	-0.5	0.1	1.1	1	1
P06348	PIG Histone H1t	HIST1H1T	-1.6	-0.4	0.0	0.7	-0.9	0.0	1.3	3.1	3	4
Q6RJV7	HUMAN Alpha-1,4-galactosyltransferase	A4GALT	-0.5	-0.6	-0.2	0.1	-1.4	-0.5	-0.5	-0.2	1	1
Q29187	PIG 60S ribosomal protein L4 (Fragment)	RPL4	-2.2	-1.5	0.3	2.1	-2.1	-1.9	-1.3	1.5	1	1
I3LM66	PIG Uncharacterized protein	PPA2	-0.2	-0.3	-0.4	-0.4	-0.1	-1.0	0.5	-0.8	2	3

F1RK32	PIG Uncharacterized protein (Fragment)	CCDC70	-0.3	0.5	-1.1	-0.4	0.8	0.5	4.4	1.6	1	1
P56975	HUMAN Pro-neuregulin-3, membrane-bound isoform	NRG3	-0.5	-0.9	0.3	-0.1	0.4	1.6	1.5	0.8	1	1
F1RGX2	PIG Uncharacterized protein	LOC100738032	0.5	-0.1	-0.7	-1.0	0.4	0.0	-0.4	-0.1	1	1
I3LGC3	PIG Uncharacterized protein (Fragment)	HAGH	0.1	-0.2	-0.4	-0.8	1.6	1.1	0.5	-1.1	3	2
P98160	HUMAN Basement membrane-specific heparan sulfate proteoglycan core protein	HSPG2	-0.6	-0.7	-0.3	0.2	-1.0	-2.1	-1.7	-1.1	12	7
I3LTA9	PIG Uncharacterized protein	LOC100739628	-0.8	-0.7	0.0	0.1	0.5	0.7	0.1	0.3	1	1
F1SKK1	PIG Uncharacterized protein	SLC25A20	0.1	0.0	-0.8	-0.7	-0.6	-0.3	1.1	2.4	4	6
H0YIC4	HUMAN Citrate synthase (Fragment)	CS	0.3	0.6	-0.6	-1.6	-0.9	-0.5	-0.5	0.9	1	1
I3LJU7	PIG Uncharacterized protein	HSPB7	-1.3	-0.3	0.2	0.2	1.1	0.5	0.2	-0.7	5	6
F1SJG9	PIG Uncharacterized protein	LOC100152831	0.6	-0.4	-0.6	-0.9	-1.5	-1.8	-1.7	-0.5	1	2
Q07955	HUMAN Serine/arginine-rich splicing factor 1	SRSF1	-0.5	-0.6	-0.2	-0.1	0.4	-0.1	-0.9	0.2	6	3
I3LQQ8	PIG Uncharacterized protein	CSF3R	0.2	-0.3	-0.6	-0.6	-0.2	-0.2	-0.3	0.4	1	1
I3L6A4	PIG Uncharacterized protein	NA	-0.3	-0.6	-0.6	0.2	0.3	0.2	1.0	-0.2	1	2
F1RZA8	PIG Uncharacterized protein	LOC100525071	-0.2	-0.4	-0.4	-0.4	-0.1	0.8	1.8	2.2	1	1
I3L693	PIG Uncharacterized protein	HNRNPR	-1.7	-0.4	0.3	0.6	-1.5	-0.1	0.8	0.1	1	2
F1RZH8	PIG Uncharacterized protein	KIAA1715	0.1	-0.9	-0.5	0.0	1.7	-0.2	-0.3	0.7	1	4
F1RG17	PIG Nucleoside diphosphate kinase	NME3	-0.1	-0.6	-0.4	-0.2	0.3	0.1	-0.2	-0.5	2	2
F1S526	PIG Uncharacterized protein	LOC100739177	0.1	-0.8	-0.4	-0.3	-0.4	-0.5	0.1	-0.1	2	1
Q7LBR1	HUMAN Charged multivesicular body protein 1b	CHMP1B	-1.0	-0.5	-0.1	0.3	-0.5	-0.5	0.5	1.3	1	1
F1RIR2	PIG Uncharacterized protein	STX4	-0.8	-0.2	-0.2	-0.1	-0.8	-0.6	0.2	-0.6	2	1
Q9NTG7	HUMAN NAD-dependent protein deacetylase sirtuin-3, mitochondrial	SIRT3	-0.6	-0.6	-0.1	-0.1	-0.1	0.0	0.8	0.6	1	1
F1RX93	PIG Uncharacterized protein	MIEN1	-0.9	-1.1	-0.1	0.9	-0.1	-0.3	0.0	0.1	2	1
I3LD43	PIG Uncharacterized protein	LOC100627689	-0.8	-0.4	-0.3	0.2	0.1	0.4	2.0	1.1	12	11
F1S5K0	PIG Uncharacterized protein	LOC100511191	-0.3	-0.9	-0.2	0.1	-0.3	0.4	-0.5	0.3	1	1
Q96T21	HUMAN Selenocysteine insertion sequence-binding protein 2	SECISBP2	0.3	0.2	-0.7	-1.1	-0.1	-0.8	-1.5	-1.1	1	1
F1RVA1	PIG Uncharacterized protein	EEF1E1	0.2	-0.6	-0.5	-0.5	0.2	0.0	-0.8	-1.5	1	1
I3L687	PIG Uncharacterized protein	HNRNPH2	-1.1	-0.9	0.1	0.5	0.4	1.3	0.5	0.1	5	2
F1SUU2	PIG Uncharacterized protein	SLC25A34	-2.1	-1.6	0.5	1.9	0.2	-0.2	-1.3	-0.4	1	1
F1RZ02	PIG Uncharacterized protein (Fragment)	AP2A2	0.0	-0.6	-0.5	-0.3	-0.1	-0.2	-0.5	-0.3	3	1
P50667	PIG Cytochrome c oxidase subunit 2	MT-CO2	0.4	0.6	-0.9	-1.4	-1.0	-1.0	-1.7	-0.5	11	10
Q29068	PIG T-complex protein 1 subunit gamma (Fragment)	CCT3	-0.5	-0.8	-0.4	0.2	-0.3	-0.4	0.2	1.0	2	2
F1RW43	PIG Uncharacterized protein	PLP2	-0.7	-0.6	-0.4	0.3	-0.9	-1.7	-0.4	0.6	1	1
I3LD01	PIG Uncharacterized protein	FIS1	-0.1	-0.3	-0.5	-0.4	-1.0	-1.1	-1.3	-0.8	3	5
F1RN91	PIG Uncharacterized protein (Fragment)	NA	-0.5	-0.3	-0.4	-0.1	-0.6	-0.6	-0.5	0.2	2	9
I3L675	PIG Uncharacterized protein (Fragment)	MYH15	0.9	-0.5	-0.8	-1.0	0.7	0.4	0.3	1.1	2	2
Q5TEC6	HUMAN Histone H3	HIST2H3PS2	-0.9	-0.1	-0.7	0.4	-2.0	-1.3	-0.4	1.1	1	1
Q29558	PIG NADP-dependent malic enzyme (Fragment)	ME1	0.0	-0.4	-0.7	-0.3	-0.4	-0.4	0.1	0.4	1	1
F1S9B7	PIG Uncharacterized protein	MRPL52	0.3	-0.3	-0.7	-0.7	0.6	0.1	1.1	0.9	1	1
Q8WUF5	HUMAN RelA-associated inhibitor	PPP1R13L	0.9	-0.7	-0.8	-0.8	2.1	1.2	-1.9	-1.3	2	1
A8D737	PIG T-cadherin	NA	-0.3	0.2	-0.6	-0.7	0.7	0.4	0.2	0.4	13	8

F1S7J6	PIG Uncharacterized protein	SAFB	-0.9	-0.8	-0.1	0.5	-1.4	0.2	0.0	1.2	5	4
F1SMW0	PIG Uncharacterized protein (Fragment)	ZCCHC2	-0.8	-0.1	-0.4	-0.1	-0.5	-0.6	1.1	0.9	1	1
Q9TUP1	PIG HnRNP A2/B1 protein (Fragment)	HNRNPA2/B1	-0.3	-0.6	-0.4	-0.1	-0.1	0.0	-0.9	-1.0	1	1
I3LM03	PIG Carbonyl reductase [NADPH] 1	CBR1	0.2	-0.3	-0.7	-0.6	-0.4	0.4	-0.9	-0.5	3	2
I3L7R0	PIG Uncharacterized protein	EHD3	-0.4	-0.4	-0.3	-0.3	0.4	0.2	0.8	1.1	7	5
F1SIC0	PIG Uncharacterized protein	PACSIN3	0.2	-0.5	-0.5	-0.6	0.2	0.2	-0.2	0.3	1	1
P10632	HUMAN Cytochrome P450 2C8	CYP2C8	-0.3	-0.6	-0.3	-0.3	-1.1	0.0	0.2	0.4	1	1
Q13554	HUMAN Calcium/calmodulin-dependent protein kinase type II subunit beta	CAMK2B	0.1	-0.5	-0.2	-0.7	-0.7	0.3	0.1	-0.5	2	3
F1S031	PIG Uncharacterized protein	NDUFS6	0.3	0.7	-0.9	-1.5	1.7	1.0	-1.3	0.0	6	6
P62745	HUMAN Rho-related GTP-binding protein RhoB	RHOB	-0.1	-0.5	-0.6	-0.3	-0.8	-1.2	-0.3	-0.5	1	1
A0PFK7	PIG F-actin-capping protein subunit beta	CAPZB	-0.3	-0.9	-0.2	0.0	-0.7	-0.9	-0.6	-0.9	11	5
F1RPJ8	PIG Uncharacterized protein	ABCB7	-0.3	-0.6	-0.6	0.0	1.0	0.6	0.4	-0.7	2	4
F1RMV5	PIG Uncharacterized protein (Fragment)	ETHE1	-0.2	-0.5	-0.4	-0.3	-0.4	0.1	1.4	1.6	1	1
A4D2F6	HUMAN Similar to Splicing factor, arginine/serine-rich, 46kD	LOC392896	-0.1	-0.7	-0.4	-0.3	0.0	1.0	1.1	0.9	1	1
F1SPR6	PIG Uncharacterized protein	RNF123	-0.7	-0.3	-0.1	-0.3	-0.8	-0.2	0.3	0.9	5	4
F1RYZ1	PIG Uncharacterized protein	CD151	-1.4	-0.5	-0.1	0.5	-1.0	-0.2	0.7	0.3	1	3
K9IVQ6	PIG Plectin (Fragment)	PLEC	-0.8	-0.9	0.0	0.3	-0.8	-1.3	-1.1	-0.1	187	201
A5GFU0	PIG GNAS complex locus	GNAS	-0.2	-0.4	-0.4	-0.4	0.8	1.5	0.6	0.2	3	3
P13533	HUMAN Myosin-6	MYH6	1.2	-0.8	-0.7	-1.2	1.9	0.9	-1.6	1.2	10	9
F1SG97	PIG Uncharacterized protein (Fragment)	KLHL42	-0.1	-1.0	0.8	-1.2	0.1	0.4	0.0	-1.9	1	1
Q8TCU6	HUMAN Phosphatidylinositol 3,4,5-trisphosphate-dependent Rac exchanger 1 protein	PREX1	-0.3	-0.1	-0.1	-1.0	-0.3	-0.3	-0.8	-1.5	1	1
I3L9C8	PIG Uncharacterized protein	ILK	-0.4	-0.7	-0.3	0.0	-0.2	0.0	-0.2	-0.8	14	12
F1RWD9	PIG Uncharacterized protein	SVIL	-0.4	-0.8	-0.3	0.0	-0.4	-0.3	-0.5	0.4	8	11
K9IVP8	PIG Obg-like ATPase 1 isoform 2	OLA1	-0.9	-0.8	0.0	0.2	-0.1	-0.3	0.0	-0.3	7	5
Q14525	HUMAN Keratin, type I cuticular Ha3-II	KRT33B	0.3	0.1	-0.9	-1.0	-0.4	-1.5	-2.5	-1.5	1	1
I3LLU8	PIG Uncharacterized protein	SBK2	-0.2	-0.6	-0.4	-0.3	-0.5	-0.2	-0.4	0.1	1	1
Q86TM3	HUMAN Probable ATP-dependent RNA helicase DDX53	DDX53	-0.5	-0.3	-0.1	-0.6	-1.3	-1.4	-0.6	-0.1	1	1
F1S0C1	PIG Uncharacterized protein	ADH5	-0.2	-0.8	-0.5	0.0	-1.1	-1.0	-0.3	-0.5	6	7
I3LPH5	PIG Uncharacterized protein	G3BP2	-0.8	-0.8	0.1	0.0	0.2	1.9	0.7	1.3	3	1
P12532	HUMAN Creatine kinase U-type, mitochondrial	CKMT1A	0.3	-0.4	-0.7	-0.7	0.5	-0.1	-0.6	-0.8	2	2
P0C221	HUMAN Coiled-coil domain-containing protein 175	CCDC175	-0.3	-0.2	-0.5	-0.5	-1.9	-2.8	-1.9	-2.4	1	1
M3VHA5	PIG SEC16-like A	SEC16A	-0.3	-0.2	-0.5	-0.5	0.3	0.5	0.9	1.1	1	1
F1SCX7	PIG Uncharacterized protein	KIF20B	-0.1	-0.1	-0.3	-0.9	2.2	1.7	1.1	-0.3	1	1
Q9HC98	HUMAN Serine/threonine-protein kinase Nek6	NEK6	-0.8	-0.4	0.3	-0.5	-0.8	0.8	5.9	0.2	1	1
Q52NJ3	PIG GTP-binding protein SAR1a	SAR1A	-1.0	-0.9	0.0	0.5	-1.1	-0.8	-1.0	-1.0	6	5
F1RGI7	PIG Uncharacterized protein	MATR3	-0.8	-0.6	-0.1	0.1	-0.6	0.5	0.4	0.6	13	10
F1RWJ6	PIG Uncharacterized protein	LOC414413	-0.3	-0.7	-0.3	-0.1	-0.3	-0.2	-0.3	0.9	14	10
F1S5M9	PIG Uncharacterized protein	ADD3	0.1	-0.7	-0.7	-0.2	0.1	0.4	-0.3	-0.2	6	6
Q6PJ56	HUMAN TRA@ protein	TRA@	0.4	-0.3	-0.6	-1.1	0.1	-0.2	0.5	-0.4	1	1
I3LN66	PIG Uncharacterized protein (Fragment)	NRBF2	0.3	-0.1	-0.7	-0.9	-0.9	-1.1	-0.6	0.3	2	1

F1RWY7	PIG Uncharacterized protein	BPHL	0.3	-0.5	-0.8	-0.5	1.4	1.4	1.5	0.5	4	4
F1SNX8	PIG Uncharacterized protein	SUN2	-0.6	-0.5	-0.3	-0.2	0.2	0.5	-0.5	0.3	7	12
F1SHF6	PIG Uncharacterized protein	HECTD1	-0.1	-0.1	-0.8	-0.5	-0.6	0.4	-0.1	0.9	2	4
P05383	PIG cAMP-dependent protein kinase catalytic subunit beta	PRKACB	-0.1	-0.7	-0.5	-0.3	-0.2	-1.2	-0.1	-0.4	6	3
Q5UES3	PIG Heat shock 60 kDa protein 1 (Fragment)	NA	0.4	0.3	-1.0	-1.1	0.8	0.8	-0.4	-1.5	5	4
Q9BQC3	HUMAN Diphthamide biosynthesis protein 2	DPH2	-0.3	-0.5	-0.3	-0.4	-0.7	-0.5	-0.3	0.8	1	1
K7GPE1	PIG Aurora kinase B (Fragment)	AIM1	1.0	-0.3	-1.2	-1.0	-0.2	-0.3	-0.6	-1.5	1	1
F1S8K5	PIG Uncharacterized protein	SUPT16H	-0.1	-0.9	-0.3	-0.2	-1.5	-1.4	-0.9	-1.7	2	1
I3LFV4	PIG Uncharacterized protein	YBX1	-1.8	-1.8	0.6	1.6	1.2	2.2	1.3	4.2	7	7
F1SF12	PIG Uncharacterized protein (Fragment)	COBL	-0.3	-0.3	-0.5	-0.4	2.4	0.1	-1.0	2.2	2	3
D2D0E0	PIG Choline/ethanolamine kinase	CHKB	-0.7	-0.1	-0.2	-0.5	0.1	0.4	0.7	0.8	2	2
F1STU9	PIG Uncharacterized protein (Fragment)	PCF11	-0.1	-0.5	-0.6	-0.4	-1.2	-1.3	-1.0	0.0	1	1
F1S2A8	PIG Uncharacterized protein	BCAP31	-1.2	-0.8	0.0	0.5	-0.2	-0.5	-0.1	1.7	13	12
P04350	HUMAN Tubulin beta-4A chain	TUBB4A	-0.3	-0.6	-0.4	-0.2	0.3	0.4	-0.3	-0.9	2	2
F1SUS1	PIG Uncharacterized protein	CROCC	-0.2	-0.1	-0.5	-0.8	-2.2	-2.5	-1.5	-2.1	4	3
F1S6Z6	PIG Uncharacterized protein	TXLNB	-0.1	-1.1	-0.5	0.2	0.7	-1.2	0.2	-0.8	5	1
F1RZL4	PIG Uncharacterized protein	ADAM-9	0.8	0.3	-1.8	-0.9	-0.8	-1.0	2.3	1.5	1	1
P62857	HUMAN 40S ribosomal protein S28	RPS28	-0.5	-0.9	-0.2	0.0	0.6	0.6	0.8	0.9	2	1
F1SN95	PIG Uncharacterized protein	NUP155	-1.0	-0.6	-0.2	0.2	0.2	0.2	1.0	0.2	2	1
F1SI50	PIG Uncharacterized protein	NDUFB3	0.0	0.4	-0.7	-1.3	-0.4	-0.5	-0.4	-0.1	3	2
F1S2K3	PIG Uncharacterized protein	LOC100520245	-0.6	-0.3	-0.4	-0.3	0.6	1.3	1.2	-1.3	3	2
O95671	HUMAN N-acetylserotonin O-methyltransferase-like protein	ASMTL	-0.4	-0.4	-0.5	-0.3	0.4	0.2	0.1	0.2	1	1
F1S5F4	PIG Uncharacterized protein	NEK7	-0.5	-0.8	-0.2	-0.1	0.2	0.3	0.0	0.1	2	2
Q8NBN7	HUMAN Retinol dehydrogenase 13	RDH13	-0.7	-0.7	-0.1	-0.1	-0.1	-0.2	-0.2	0.3	2	2
I3LNG8	PIG Uncharacterized protein	LOC100623923	-0.7	-0.9	-0.2	0.3	0.7	0.7	1.7	0.5	13	13
F1RUJ0	PIG Adenylyl cyclase-associated protein (Fragment)	CAP2	-0.2	-0.4	-0.2	-0.7	-0.6	0.2	-0.5	0.3	3	4
I3LCX2	PIG Uncharacterized protein (Fragment)	NA	-1.3	-0.8	-0.2	0.8	0.1	0.7	0.7	-0.3	11	7
Q8NDB2	HUMAN B-cell scaffold protein with ankyrin repeats	BANK1	0.5	-0.1	-0.7	-1.2	1.6	3.1	0.0	0.8	1	1
F1SUR6	PIG Uncharacterized protein	C2CD3	-0.4	-0.1	-0.6	-0.5	0.3	0.0	0.4	0.1	1	1
P04175	PIG NADPH--cytochrome P450 reductase	POR	-0.9	-0.1	-0.4	-0.1	-0.9	-1.0	-1.3	-1.3	4	1
A5GFN8	PIG RNA-binding region (RNP1, RRM) containing 1	RNPC1	-0.1	-0.8	-0.6	-0.1	0.3	-0.3	0.2	0.0	1	1
Q6UN15	HUMAN Pre-mRNA 3'-end-processing factor FIP1	FIP1L1	-0.6	-1.2	-0.1	0.4	-0.7	0.4	0.9	3.6	1	1
I3LQU8	PIG Uncharacterized protein	NA	-0.3	-0.2	-0.7	-0.4	-0.6	-0.9	-1.0	-0.6	3	4
F1S1A8	PIG Uncharacterized protein	NDUFS2	0.6	0.1	-0.9	-1.4	0.4	0.0	-1.2	-1.4	16	17
F1RHN4	PIG Uncharacterized protein	TXNRD2	-0.4	-0.2	-0.4	-0.5	-0.4	0.0	-1.4	-1.5	2	3
F1SSL6	PIG Proteasome subunit alpha type	LOC100154408	-0.5	-0.5	-0.3	-0.2	1.0	0.3	1.6	-0.4	11	7
M3V839	PIG Mitogen-activated protein kinase 3	MAPK3	-0.2	-0.9	-0.6	0.2	1.0	0.1	-0.7	0.0	3	1
K9IW88	PIG Methionine aminopeptidase 2	METAP2	-1.2	-1.0	0.0	0.7	1.0	-0.4	0.4	2.1	3	1
L8E8Q4	HUMAN Alternative protein FAM178A	FAM178A	-0.4	0.0	-0.6	-0.6	0.2	0.3	1.0	-0.1	1	1
Q9ULZ3	HUMAN Apoptosis-associated speck-like protein containing a CARD	PYCARD	-0.3	-0.1	-0.4	-0.8	-1.5	-0.9	-0.7	-0.5	1	1

Q9NQG5	HUMAN Regulation of nuclear pre-mRNA domain-containing protein 1B	RPRD1B	-0.1	-0.1	-0.6	-0.8	-3.2	-2.0	-2.4	0.0	2	1
F1SC98	PIG Uncharacterized protein (Fragment)	IDE	-0.5	-0.5	-0.5	-0.1	-0.1	1.6	0.3	0.3	4	2
F1SJ04	PIG Uncharacterized protein (Fragment)	LOC100515478	-0.1	0.1	-0.5	-1.1	4.6	2.1	-1.2	-0.7	6	1
F2Z521	PIG Platelet-activating factor acetylhydrolase IB subunit alpha	PAFAH1B1	-0.4	-0.7	-0.4	-0.1	0.5	-0.2	0.4	0.1	6	3
P54612	PIG Serine/threonine-protein phosphatase 2A 65 kDa regulatory subunit A alpha isoform	PPP2R1A	-0.4	-0.9	-0.3	0.0	0.0	0.1	-0.8	-0.4	17	15
Q865U8	PIG Ankyrin repeat domain-containing protein 1	ANKRD1	-0.7	0.0	-0.4	-0.5	-0.4	-0.2	-0.8	-2.1	11	10
F1RGP8	PIG Uncharacterized protein (Fragment)	PBXIP1	-0.7	-0.3	-0.3	-0.3	0.1	0.1	-0.4	-1.0	5	5
I3LJQ8	PIG Uncharacterized protein (Fragment)	NA	-1.4	-1.0	-0.2	1.0	-2.3	-1.8	-1.5	-0.9	2	3
I3LCI0	PIG Uncharacterized protein	NA	-0.5	0.1	-0.3	-0.8	-0.5	-0.4	1.0	0.9	3	2
F1RGY4	PIG Uncharacterized protein	LYST	-0.5	-0.8	0.0	-0.3	-0.2	0.6	1.4	0.1	1	2
O02854	PIG Glycerol-3-phosphate dehydrogenase (Fragment)	mGPD	0.1	-0.3	-0.7	-0.7	-0.2	0.2	-1.0	-0.9	4	4
A7XXV9	PIG Sirtuin 5	NA	0.4	-0.3	-0.9	-0.8	0.2	-0.3	-0.6	-1.1	4	6
F1SSX9	PIG Uncharacterized protein	SEMA6C	0.1	-0.4	-0.6	-0.7	-0.6	-0.8	-0.6	-0.4	1	1
I3LC00	PIG Uncharacterized protein	LOC100524144	-1.5	-1.2	0.2	0.9	-0.5	-0.6	-1.0	-0.6	4	3
K9IVU3	PIG AP-1 complex subunit gamma-1	AP1G1	-0.6	-0.5	-0.6	0.0	-1.6	-1.0	-0.8	-0.5	1	1
F1SM98	PIG Uncharacterized protein	NDUFV2	0.4	0.4	-0.9	-1.6	1.2	0.8	-0.2	-1.8	13	12
A9CQL8	PIG Prostamide/prostaglandin F synthase	FAM213B	-0.4	-0.5	-0.4	-0.3	-0.1	1.0	0.2	0.4	1	1
F1SI13	PIG Peptidyl-prolyl cis-trans isomerase (Fragment)	FKBP3	-0.9	-0.5	-0.2	-0.1	0.3	0.9	-0.1	-0.8	5	5
F1S9W6	PIG Uncharacterized protein (Fragment)	EPS15L1	-0.4	-1.3	-0.4	0.5	0.7	1.4	0.8	1.5	2	1
I3L751	PIG Uncharacterized protein	REXO4	-0.2	0.2	-0.9	-0.8	-1.1	-1.0	0.0	0.0	1	1
F1SLU8	PIG Uncharacterized protein	GNB3	-0.8	-1.2	0.0	0.3	-1.4	-0.8	-0.6	0.4	2	1
Q4QTK1	PIG Programmed death ligand 1	PDL1	-0.8	-0.3	-0.3	-0.2	-1.1	-0.8	-0.9	-0.5	1	1
P80895	PIG Protein-L-isoaspartate(D-aspartate) O-methyltransferase	PCMT1	-0.2	-0.5	-0.5	-0.5	0.4	0.1	-0.1	-1.0	10	10
P18077	HUMAN 60S ribosomal protein L35a	RPL35A	-1.0	-1.4	0.0	0.7	-0.4	-0.6	-0.8	0.1	4	4
F1RS49	PIG Uncharacterized protein	ABCE1	-0.6	-0.9	-0.4	0.2	2.4	2.1	0.2	0.0	9	3
P23687	PIG Prolyl endopeptidase	PREP	-0.3	-0.8	-0.5	0.0	-0.4	-0.2	-0.9	-0.9	10	11
F1S3X5	PIG Uncharacterized protein (Fragment)	NA	-0.9	-0.7	-0.2	0.1	-0.6	0.0	0.1	-0.4	1	1
P61026	HUMAN Ras-related protein Rab-10	RAB10	-0.6	-1.0	-0.4	0.3	-1.0	-0.4	0.5	2.2	4	3
I3LA48	PIG Transcription elongation factor SPT5	SUPT5H	-0.2	-0.3	-0.6	-0.7	1.0	0.7	0.1	-1.8	2	1
F1RM03	PIG Uncharacterized protein	SAE1	-0.4	-0.3	-0.5	-0.4	-1.6	-2.1	-4.6	-4.1	1	1
F1S2Q5	PIG Uncharacterized protein	ZC2HC1C	-0.3	-0.1	-0.8	-0.5	-0.2	-0.6	-0.6	0.1	1	1
P29797	PIG Guanine nucleotide-binding protein G(s) subunit alpha	GNAS	-0.7	-0.6	-0.4	-0.1	-0.5	0.4	0.3	0.2	5	6
F1SJU4	PIG Uncharacterized protein (Fragment)	PARVB	-0.6	-0.6	-0.3	-0.3	0.9	1.2	1.3	-0.1	3	2
P79382	PIG Microsomal glutathione S-transferase 1	MGST1	0.0	-0.7	-0.6	-0.4	-0.1	0.7	-0.4	-0.6	1	1
K9J4S7	PIG Kinesin-1 heavy chain	KIF5B	-0.8	-1.1	-0.4	0.6	-1.7	-1.5	-1.4	0.6	1	2
F1S663	PIG Uncharacterized protein	LAMC1	-0.5	-0.6	-0.5	-0.2	-0.7	-1.2	-1.0	-0.2	30	31
P62070	HUMAN Ras-related protein R-Ras2	RRAS2	-0.5	0.1	-0.5	-0.8	0.0	1.0	0.6	2.3	1	1
F1SMN5	PIG Uncharacterized protein	FLNC	-1.6	-1.0	0.4	0.4	-1.1	-0.5	0.5	0.5	119	117
I3LB94	PIG Uncharacterized protein	DTNA	0.3	-0.4	-0.6	-1.1	0.6	0.9	0.2	-0.2	1	3
I3LLW3	PIG Uncharacterized protein	NA	0.3	-0.2	-0.6	-1.2	-0.9	-0.7	-1.5	-0.9	6	10

K7GL60	PIG Uncharacterized protein	EIF1AX	-1.2	-1.0	0.1	0.4	0.6	0.6	0.9	0.4	2	1
F1RWF5	PIG Uncharacterized protein	NA	-0.1	0.3	-0.8	-1.0	-0.8	-0.3	-0.9	-0.2	12	12
F1SV06	PIG Uncharacterized protein	PABPC4	-0.7	-1.0	-0.1	0.1	-0.2	0.2	0.2	0.1	10	12
F1SB57	PIG Uncharacterized protein	LOC100737606	-0.6	-0.3	-0.6	-0.3	0.4	0.1	-0.6	-0.4	3	2
A1XQS7	PIG MCEE	MCEE	0.1	0.7	-1.0	-1.6	0.7	0.5	1.2	-1.4	3	3
F1S146	PIG Uncharacterized protein	ANK2	-0.5	-0.5	-0.2	-0.4	-0.7	0.4	-0.3	-0.5	6	9
I3L973	PIG Uncharacterized protein (Fragment)	PARVA	-0.6	-0.7	-0.5	0.0	-0.2	-0.8	-0.8	0.2	5	3
K7GNM9	PIG Uncharacterized protein	RPL8	-1.1	-1.2	0.1	0.5	-0.9	-0.5	0.0	0.7	2	3
Q96RW7	HUMAN Hemicentin-1	HMCN1	-0.4	-0.3	-0.7	-0.4	1.3	0.2	0.4	0.7	1	1
I3LKT2	PIG Uncharacterized protein	LOC100625222	-0.5	-1.1	-0.4	0.2	-1.1	-1.1	-0.6	0.7	1	2
I3LI48	PIG Uncharacterized protein (Fragment)	NA	-0.3	-0.3	-0.7	-0.5	-0.8	-0.4	0.1	1.0	1	1
I3LDK5	PIG Uncharacterized protein	NA	0.1	0.6	-1.1	-1.4	-0.2	-0.3	-0.2	0.0	1	1
Q92614	HUMAN Unconventional myosin-XVIIIa	MYO18A	0.1	-0.5	-0.6	-0.7	-0.3	0.2	-1.2	-1.1	1	2
F1RWL7	PIG Uncharacterized protein	LOC100157053	0.4	0.1	-0.8	-1.4	-0.4	-0.3	-0.3	-0.2	7	6
F1SI09	PIG Uncharacterized protein	PPP1R7	0.0	-0.6	-0.7	-0.4	1.3	0.9	1.3	-0.1	9	3
P62826	HUMAN GTP-binding nuclear protein Ran	RAN	-1.4	-0.7	0.1	0.2	-0.6	-0.5	-0.1	0.4	3	3
K9IVR7	PIG WD repeat-containing protein 1	WDR1	-0.8	-1.3	-0.1	0.4	-0.5	-0.8	0.1	-0.5	21	18
Q9UI43	HUMAN Putative ribosomal RNA methyltransferase 2	FTSJ2	0.1	-0.9	-0.6	-0.4	0.0	0.5	0.1	0.8	1	1
F1S4P6	PIG Eukaryotic translation initiation factor 3 subunit A	EIF3A	-1.2	-0.9	-0.1	0.4	-1.0	-0.6	0.3	0.5	23	16
I3L990	PIG Uncharacterized protein (Fragment)	NA	0.0	-0.3	-0.6	-0.9	0.6	0.7	-0.1	-1.2	3	2
Q9P0L0	HUMAN Vesicle-associated membrane protein-associated protein A	VAPA	-0.5	-1.0	-0.4	0.1	-1.2	-1.4	-0.1	-0.1	2	3
P17741	PIG High mobility group protein B2	HMGB2	-0.5	-0.3	-0.4	-0.5	0.3	0.9	1.3	0.8	2	4
F1SAF6	PIG Ubiquitin carboxyl-terminal hydrolase	LOC100520041	-0.4	-0.4	-0.5	-0.5	-0.2	0.3	-0.6	1.0	1	1
I3LT85	PIG Uncharacterized protein	RCC1	0.2	-0.6	-0.7	-0.7	1.4	1.3	1.5	0.4	1	1
F1RP22	PIG Uncharacterized protein	NA	0.0	-0.4	-1.2	-0.2	0.7	0.0	-1.0	-0.8	2	4
F1RN06	PIG Tyrosine-protein kinase transmembrane receptor (Fragment)	ROR2	0.4	-0.5	-0.9	-0.8	-0.2	0.4	-0.3	0.1	1	1
Q02038	PIG Neurolysin, mitochondrial	NLN	0.4	-0.4	-0.8	-1.0	0.8	1.4	0.0	0.2	1	2
I3LKN7	PIG Uncharacterized protein	ACBD3	-0.8	-0.5	-0.2	-0.3	0.9	-0.1	-0.1	0.8	2	1
Q9H2F5	HUMAN Enhancer of polycomb homolog 1	EPC1	-1.8	-1.7	-0.4	2.1	-1.4	-1.9	-1.5	0.6	1	1
F1SGY1	PIG Anoctamin (Fragment)	ANO6	-0.8	-0.9	-0.2	0.2	-1.0	0.2	-0.4	0.3	2	1
F1SA77	PIG Uncharacterized protein	CHD2	0.6	-0.8	-0.9	-0.6	0.0	0.8	-0.6	1.0	1	1
C3VML1	PIG Claudin	CLDN5	-1.0	-0.3	-0.5	-0.1	-1.1	0.1	-1.3	-1.1	1	1
P61981	HUMAN 14-3-3 protein gamma	YWHAG	-0.9	-1.2	-0.1	0.4	-0.7	-1.0	-0.8	-1.5	8	9
B9UJD6	PIG C1q and tumor necrosis factor related protein 3 isoform b	C1QTNF3	-0.9	-0.6	-0.1	-0.2	0.1	-0.2	0.3	-0.5	1	1
Q6QAR9	PIG Puromycin sensitive aminopeptidase (Fragment)	NA	-0.5	-1.0	-0.3	0.0	-0.8	-0.3	-0.2	0.2	10	10
F1SQK1	PIG Uncharacterized protein	NA	-0.7	-0.9	-0.2	0.0	1.1	-0.5	-0.7	-0.6	18	19
D7EZJ4	PIG Immunity-related GTPase family cinema protein	IRGC	0.1	-0.3	-0.8	-0.8	0.8	0.3	-0.6	-1.0	1	1
F1SP54	PIG Uncharacterized protein	NIPSNAP3B	0.1	-0.3	-0.6	-1.0	-0.6	-0.9	1.2	0.1	9	10
O00429	HUMAN Dynamin-1-like protein	DNM1L	0.6	-0.8	-0.8	-0.8	0.1	0.2	-0.7	-0.2	2	3
K7GQA9	PIG Uncharacterized protein (Fragment)	GSTZ1	0.8	-0.1	-1.1	-1.4	-1.3	-1.4	-1.5	-0.6	2	5

F1SQN5	PIG Uncharacterized protein	EIF1AX	-0.2	-0.5	-0.7	-0.4	0.4	0.6	0.9	1.6	3	1
Q9Y2U5	HUMAN Mitogen-activated protein kinase kinase kinase 2	MAP3K2	-1.4	-1.5	0.1	0.9	0.3	0.2	1.7	0.7	1	1
I3LV38	PIG Uncharacterized protein (Fragment)	MSRB3	-0.4	-0.8	-0.5	-0.2	-0.3	-1.6	-0.9	-1.2	1	1
Q86YV0	HUMAN RAS protein activator like-3	RASAL3	-0.6	-0.4	-0.4	-0.4	-1.7	-0.6	-1.0	-1.3	2	1
F1S4V0	PIG Uncharacterized protein (Fragment)	COX7A2L	-0.4	-0.4	-0.5	-0.6	-0.4	-0.2	0.0	0.0	2	2
F1RWX0	PIG Uncharacterized protein (Fragment)	NA	-0.6	-0.9	-0.2	-0.1	-0.5	-0.9	0.0	1.0	2	1
F1RRT2	PIG Uncharacterized protein (Fragment)	MYL4	-0.1	0.3	-0.9	-1.1	2.2	-0.4	0.4	0.2	9	3
P24043	HUMAN Laminin subunit alpha-2	LAMA2	-0.5	-0.4	-0.6	-0.4	-0.4	-0.2	-0.6	0.2	13	17
K7GRF2	PIG Uncharacterized protein	RPL8	-1.2	-1.2	-0.1	0.7	-1.1	-0.8	0.2	1.1	2	3
I3LR17	PIG Uncharacterized protein	CORO1A	-0.5	-1.1	-0.9	0.6	1.3	1.8	1.0	1.4	1	1
F1S0L1	PIG Uncharacterized protein	KRT14	0.0	-1.4	-0.4	0.0	-0.7	-1.6	-0.4	5.0	4	4
Q562W2	HUMAN Actin-like protein (Fragment)	ACT	0.8	-0.3	-0.9	-1.4	1.6	0.2	-1.1	0.7	1	2
P52815	HUMAN 39S ribosomal protein L12, mitochondrial	MRPL12	-0.3	0.3	-0.9	-1.0	3.3	1.3	1.7	-0.3	4	1
Q16775	HUMAN Hydroxyacylglutathione hydrolase, mitochondrial	HAGH	-0.4	-0.8	-0.5	-0.2	-1.0	-0.2	1.0	-0.7	1	1
M3VK09	PIG PHD finger protein 3	PHF3	-0.3	-0.7	-0.6	-0.3	-1.0	-1.3	-0.3	0.4	1	1
I3LS72	PIG Uncharacterized protein	COL6A1	-1.5	-1.4	-0.4	1.5	-2.9	-3.4	-2.2	1.5	10	6
F1SHC3	PIG Uncharacterized protein (Fragment)	KMT2D	-1.3	0.4	-0.6	-0.3	1.4	0.9	1.1	2.1	1	1
F2Z5Q6	PIG 40S ribosomal protein S6 (Fragment)	RPS6	-0.5	-1.0	-0.5	0.2	0.2	-0.1	-0.4	1.4	1	1
B7Z1U0	HUMAN Metal transporter CNNM4	CNNM4	-0.4	-0.4	-0.5	-0.6	-0.3	0.5	0.9	2.4	1	1
K9J4R8	PIG Protein S100-A13	S100A13	-0.2	-0.8	-0.6	-0.3	0.8	-1.4	-1.2	-0.5	3	2
O79874	PIG NADH-ubiquinone oxidoreductase chain 1	MT-ND1	0.1	-0.1	-0.8	-1.0	-0.6	-0.4	-1.4	-1.6	2	3
F1RQF6	PIG Uncharacterized protein (Fragment)	CLINT1	-0.7	-0.4	-0.6	-0.1	0.9	0.1	-0.4	0.2	4	1
Q767M3	PIG Valine--tRNA ligase, mitochondrial	VARS2	-0.5	-0.5	-0.3	-0.6	-0.1	-0.1	1.2	0.4	3	1
I3LIB5	PIG Uncharacterized protein	MYPN	0.0	-0.8	-0.8	-0.3	-0.5	-0.9	-0.6	-0.5	3	10
Q63HR2	HUMAN Tensin-like C1 domain-containing phosphatase	TENC1	-0.2	-0.4	-0.6	-0.6	-0.5	-0.3	0.3	-0.5	1	2
Q53HH3	HUMAN General transcription factor IIH, polypeptide 4, 52kDa variant (Fragment)	NA	0.2	0.5	-0.9	-1.7	0.3	-0.2	-1.7	0.5	1	1
Q14195-2	HUMAN Isoform LCRMP-4 of Dihydropyrimidinase-related protein 3	DPYSL3	-0.9	-1.2	-0.3	0.5	0.1	-0.5	0.7	-0.1	4	4
F1RZC6	PIG Uncharacterized protein (Fragment)	CCDC141	0.3	-0.7	-0.8	-0.6	0.0	0.9	-0.3	0.0	6	12
Q16836	HUMAN Hydroxyacyl-coenzyme A dehydrogenase, mitochondrial	HADH	0.5	-0.4	-0.9	-1.2	-0.2	-0.4	-1.1	-0.5	1	2
F1RH94	PIG Uncharacterized protein (Fragment)	HNRNPA0	-0.5	-0.8	-0.3	-0.2	0.6	0.5	-0.6	-1.2	3	2
I3LEV7	PIG Uncharacterized protein	SH3BGR	-0.1	-0.7	-0.5	-0.7	0.2	-1.0	-0.4	-0.7	2	3
Q9UK54	HUMAN Hemoglobin beta subunit variant (Fragment)	HBB	0.8	-0.3	-1.1	-1.4	-0.5	-0.7	0.1	-1.4	1	1
A8MXV4	HUMAN Nucleoside diphosphate-linked moiety X motif 19, mitochondrial	NUDT19	0.1	-0.4	-0.6	-1.0	1.2	0.3	1.6	-0.3	1	1
F1RFX9	PIG Uncharacterized protein	OMC	0.0	0.0	-0.8	-1.1	-1.5	-1.2	-1.5	-0.5	13	11
Q99028	PIG Catechol O-methyltransferase (Fragment)	COMT	-0.5	-0.5	-0.6	-0.4	0.6	0.8	-1.1	-2.0	6	4
I3LE16	PIG Uncharacterized protein (Fragment)	PGAM5	0.3	-0.5	-0.8	-0.9	0.8	0.6	0.6	-0.3	3	5
F1RXW5	PIG Uncharacterized protein	TMEM177	-0.1	0.3	-1.0	-1.2	-0.3	-0.6	0.0	0.0	2	4
P12109	HUMAN Collagen alpha-1(VI) chain	COL6A1	-1.9	-1.2	-0.3	1.4	-1.9	-2.1	-2.0	0.8	6	5
Q8NF91	HUMAN Nesprin-1	SYNE1	-1.5	-0.8	0.1	0.3	-0.2	-0.2	-0.9	0.0	1	1
I3LHT5	PIG Uncharacterized protein	NA	-0.9	-0.9	-0.4	0.3	-0.2	0.1	1.4	1.7	1	1

I3LUB8	PIG Uncharacterized protein	KIF5C	0.0	-0.3	-0.8	-0.8	3.4	3.7	2.8	-0.9	1	1
I3LLM9	PIG Uncharacterized protein	LOC100523003	0.1	-0.6	-0.7	-0.7	-0.1	0.4	0.4	-0.9	4	4
P61978	HUMAN Heterogeneous nuclear ribonucleoprotein K	HNRNPK	-0.6	-0.7	-0.6	0.0	0.3	0.6	0.1	-0.2	4	2
F1SSV2	PIG Uncharacterized protein	CHP1	-1.4	-1.2	0.2	0.5	-0.4	-0.8	-1.0	-1.5	1	1
Q29245	PIG Clathrin coat assembly protein AP50 (Fragment)	NA	-0.6	-0.7	-0.6	0.0	-1.7	-0.7	-0.4	0.8	1	1
Q86YE8	HUMAN Zinc finger protein 573	ZNF573	0.0	-0.1	-0.6	-1.2	-0.3	-0.8	-0.9	-2.1	1	1
F1RG31	PIG Uncharacterized protein (Fragment)	LOC100520831	0.7	-1.0	-1.1	-0.5	-0.1	0.3	-0.2	-0.4	1	1
Q9GLL4	PIG Glycine cleavage system T-protein (Fragment)	NA	-0.3	-0.4	-0.9	-0.3	-1.6	-1.0	-0.9	0.7	1	1
Q9HCE1	HUMAN Putative helicase MOV-10	MOV10	-0.9	-0.3	-0.5	-0.2	-0.5	-0.3	-1.4	0.4	2	1
O18887	PIG K-Cl cotransporter	SLC12A4	-0.8	-0.2	-0.5	-0.4	-1.3	-0.7	-0.9	0.6	1	2
F1RND9	PIG Uncharacterized protein	MNDA	-1.4	-0.5	0.1	-0.2	0.0	0.5	0.0	44.2	2	1
B6DZ39	PIG APPL2	appl2	-0.5	-1.1	-0.3	-0.1	-0.4	-0.2	0.7	1.8	2	2
Q2MJV9	PIG Palmdelphin	PALMD	-0.3	-0.4	-0.7	-0.5	2.2	2.9	-0.5	0.4	9	14
I3L614	PIG Uncharacterized protein	NA	0.1	0.5	-0.8	-1.7	0.1	-0.1	-1.7	-1.4	7	6
I3LV12	PIG Uncharacterized protein (Fragment)	LOC100623243	0.0	-0.7	-1.1	-0.1	1.9	-0.2	0.9	0.8	1	1
F1SMQ5	PIG Uncharacterized protein	STRIP2	-1.0	-1.1	-0.5	0.6	-0.7	-0.8	0.3	0.4	1	2
F1S0D9	PIG Uncharacterized protein	DARS	-0.8	-1.1	-0.2	0.1	-1.0	-1.0	0.0	-0.2	8	6
I3LD72	PIG Uncharacterized protein	EHD2	-0.3	-0.5	-0.8	-0.4	-0.8	-0.5	-1.1	-0.2	19	17
Q01449	HUMAN Myosin regulatory light chain 2, atrial isoform	MYL7	0.0	-0.6	-0.5	-0.9	0.6	-0.4	-0.7	-2.2	1	1
F1RVS8	PIG Uncharacterized protein	MTCH1	-0.7	-0.9	-0.5	0.1	0.1	-0.1	-0.1	0.1	2	2
I3LJW4	PIG Uncharacterized protein (Fragment)	ACO1	-0.6	-0.9	-0.4	-0.1	-0.5	0.1	0.0	0.1	16	21
I3LIC2	PIG Uncharacterized protein	LRPPRC	0.0	-0.6	-0.7	-0.6	0.1	-0.2	-0.9	-0.4	22	31
I3LC09	PIG Uncharacterized protein (Fragment)	TIMM50	0.0	-0.2	-0.8	-0.9	-0.7	-0.4	-0.1	0.4	3	9
Q9HBI1	HUMAN Beta-parvin	PARVB	-0.2	-1.0	-0.6	-0.3	0.0	-0.8	-1.3	-0.5	1	1
G3CKJ2	PIG Glyceraldehyde-3-phosphate dehydrogenase (Fragment)	GAPDH	-0.3	-0.8	-0.5	-0.4	-0.2	-0.6	-0.2	0.0	5	2
F1SA70	PIG Uncharacterized protein	HSPA2	-1.1	-1.0	-0.1	0.3	-0.1	0.5	0.3	-1.1	11	9
P05024	PIG Sodium/potassium-transporting ATPase subunit alpha-1	ATP1A1	-0.7	-0.3	-0.5	-0.5	-0.9	-0.4	-0.8	0.0	39	42
F1S9Q3	PIG Uncharacterized protein	NA	-1.2	-1.1	0.0	0.3	-0.7	-0.1	0.1	-0.7	22	21
I3LJX2	PIG Uncharacterized protein	NA	-0.8	-1.2	-0.5	0.5	2.3	2.5	1.2	2.3	2	2
Q5TAH2	HUMAN Sodium/hydrogen exchanger 11	SLC9C2	-0.1	-0.6	-0.6	-0.7	2.4	4.9	4.2	0.8	1	1
F1S1Q7	PIG Uncharacterized protein (Fragment)	LOC100153260	0.0	-0.6	-0.7	-0.7	-0.7	-1.8	-1.2	-0.2	10	13
I3L9I6	PIG Uncharacterized protein	BCAM	-0.1	-0.3	-0.8	-0.7	-0.1	-0.3	-1.1	-1.1	19	16
I3LAA4	PIG Uncharacterized protein	NA	-0.8	-1.2	-0.3	0.2	-0.6	-1.4	-0.4	-0.1	5	4
F1SA96	PIG Uncharacterized protein	IAH1	-0.5	-0.7	-0.5	-0.2	0.2	-1.1	0.2	-0.7	4	5
F1SMB6	PIG Uncharacterized protein	HSPB2-C11orf52	0.0	-0.7	-0.9	-0.6	1.7	2.0	0.9	-1.3	6	5
F1SUG4	PIG Uncharacterized protein	TCEB2	-0.8	-0.9	-0.3	0.0	-0.1	-0.9	-1.2	-1.9	6	2
F1RFT1	PIG Uncharacterized protein	LOC100513927	-0.6	-0.9	-0.3	-0.3	-0.1	-0.5	-0.8	-1.7	4	4
F1RN43	PIG Uncharacterized protein	CUL4A	-0.8	-1.0	-0.4	0.2	0.0	-0.2	-0.6	0.6	1	2
F1STL4	PIG Uncharacterized protein	MECR	-0.5	-0.5	-0.6	-0.5	-0.7	-0.9	-0.5	0.2	3	4
A4FU69	HUMAN EF-hand calcium-binding domain-containing protein 5	EFCAB5	-0.8	-1.2	-0.3	0.2	0.0	0.3	1.4	0.8	1	2

P61081	HUMAN NEDD8-conjugating enzyme Ubc12	UBE2M	-0.6	-1.3	-0.3	0.1	0.1	0.3	0.6	0.2	3	3
F1S398	PIG Uncharacterized protein	CLTB	-0.5	-0.7	-0.6	-0.2	1.7	0.8	0.1	-0.7	5	2
Q9H9J1	HUMAN HCG1782272	hCG_1782272	-0.8	-0.8	-0.3	-0.2	-1.0	-1.8	-0.8	-0.5	1	1
F1SJI2	PIG Uncharacterized protein (Fragment)	LOC100517377	-0.7	-0.5	-0.6	-0.2	0.3	-0.3	-0.8	-0.1	4	2
I3LCS2	PIG Uncharacterized protein (Fragment)	NA	-0.4	-0.6	-0.5	-0.5	1.3	1.3	1.9	0.3	3	1
Q2L969	PIG Metaxin-2	MTX2	-0.6	-0.8	-0.4	-0.2	0.1	-0.2	0.2	0.2	1	2
F1RKW4	PIG Mitochondrial Rho GTPase (Fragment)	RHOT1	-0.2	-0.9	-0.6	-0.4	0.8	-0.5	-1.1	-0.5	2	1
F1RYS9	PIG Uncharacterized protein	FAM47E-STBD1	-0.5	-1.3	-0.4	0.1	0.1	0.4	0.5	1.3	3	6
K7GKK5	PIG Uncharacterized protein (Fragment)	UBE2N	-1.0	-0.9	-0.3	0.1	-0.6	0.1	0.2	0.0	7	4
I3L6D7	PIG Uncharacterized protein	DSG2	0.6	-0.8	-1.1	-0.7	0.4	0.5	-0.6	0.0	6	9
F1S4Y0	PIG Uncharacterized protein	ACSS2	-0.6	-0.9	0.0	-0.5	-0.8	-1.2	-1.0	-0.3	1	1
P36956	HUMAN Sterol regulatory element-binding protein 1	SREBF1	-0.3	-0.6	-0.4	-0.8	0.7	2.4	1.3	-0.1	1	1
P36968	PIG Phospholipid hydroperoxide glutathione peroxidase, mitochondrial	GPX4	-0.1	-0.5	-0.7	-0.8	-1.3	0.5	-1.5	0.8	3	4
F1SMX5	PIG Uncharacterized protein	CAB39	-0.5	-0.6	-0.5	-0.5	0.0	-1.3	-2.7	-1.8	2	1
P37111	PIG Aminoacylase-1	ACY1	0.0	-0.9	-0.8	-0.4	1.0	1.1	1.9	0.1	8	8
P62888	HUMAN 60S ribosomal protein L30	RPL30	-0.8	-1.0	-0.3	0.1	-0.6	-0.4	0.0	0.5	4	4
Q0MVN8	PIG Quinone oxidoreductase	CRYZ	-0.7	-0.8	-0.4	-0.3	-0.2	0.0	0.6	0.3	2	2
F1S5R6	PIG Uncharacterized protein	NA	-1.0	-1.3	0.4	-0.3	-0.5	-0.7	-0.6	0.0	4	6
O77591	PIG Inositol monophosphatase 1	IMPA1	0.0	-0.7	-0.8	-0.6	1.6	1.0	1.5	-0.6	7	9
I3LG73	PIG Uncharacterized protein (Fragment)	RSL1D1	-1.6	-1.0	-0.1	0.6	0.2	2.1	0.9	1.0	4	4
Q9H4I8	HUMAN Serine hydrolase-like protein 2	SERHL2	-0.7	0.2	-0.7	-0.8	-0.5	-0.5	-1.5	-0.4	1	1
A1XQU3	PIG 60S ribosomal protein L14	RPL14	-1.6	-1.1	0.0	0.6	-1.7	-1.0	-1.2	0.0	4	4
Q9BQP7	HUMAN Mitochondrial genome maintenance exonuclease 1	MGME1	-0.7	0.0	-0.5	-0.9	-0.1	0.2	-0.5	-0.4	1	1
Q06AK6	PIG Tuftelin-interacting protein 11	TFIP11	1.3	-2.0	-1.0	-0.5	3.3	0.7	2.3	1.8	1	1
I3LC47	PIG Uncharacterized protein	NA	-0.1	-0.6	-0.7	-0.6	-0.7	-1.0	-1.0	-0.1	5	5
F1SUF2	PIG Uncharacterized protein (Fragment)	HK1	-0.5	-0.3	-0.6	-0.7	-0.8	-0.7	-0.4	0.6	23	22
I3LQ84	PIG Uncharacterized protein	NA	-1.4	-1.2	-0.5	1.0	-1.3	-1.8	-1.5	1.0	21	24
I3LCK2	PIG Uncharacterized protein	LOC100622515	-0.1	-0.9	-0.6	-0.4	0.3	-0.2	0.7	0.8	1	1
Q9N250	PIG Titin (Fragment)	TTN	0.1	-0.2	-1.1	-0.9	1.6	1.8	2.5	1.9	4	6
Q9NXZ1	HUMAN Sarcoma antigen 1	SAGE1	-0.2	-0.7	-0.7	-0.6	2.1	1.4	0.0	-0.5	1	1
P12883	HUMAN Myosin-7	MYH7	-0.6	-0.5	-0.4	-0.7	-1.9	-0.6	-1.7	-0.9	5	3
F1SLU6	PIG Ubiquitin carboxyl-terminal hydrolase	USP5	-0.9	-1.1	-0.2	0.2	-0.5	0.0	-0.7	0.2	14	7
F1RJC7	PIG Uncharacterized protein (Fragment)	MIB2	-1.0	-0.2	-0.5	-0.4	0.9	1.6	1.0	2.2	1	2
Q9NXB0	HUMAN Meckel syndrome type 1 protein	MKS1	0.1	-0.5	-0.9	-0.8	-1.0	-0.5	-1.8	-0.8	1	1
I3L820	PIG Uncharacterized protein (Fragment)	RRAD	-0.3	-0.8	-0.1	-0.9	-2.3	-0.1	-1.0	-1.2	2	2
F1SSS1	PIG Uncharacterized protein	LANCL1	-0.1	-1.0	-0.6	-0.4	-0.1	0.0	0.4	-0.6	4	4
K7GKS8	PIG Uncharacterized protein	PTCD3	0.3	-1.1	-1.1	-0.2	-0.1	-0.9	0.0	-1.3	1	3
F1RLQ9	PIG Uncharacterized protein (Fragment)	NA	-0.3	-0.6	-0.6	-0.6	-0.2	0.0	-2.4	0.3	3	3
Q9BX66	HUMAN Sorbin and SH3 domain-containing protein 1	SORBS1	-0.5	-1.3	-0.4	0.0	0.1	0.4	0.2	0.6	1	1
Q03001-3	HUMAN Isoform 3 of Dystonin	DST	-0.1	-0.8	-0.6	-0.6	1.9	0.1	1.0	0.0	2	3

K9IWG6	PIG Spectrin beta chain, brain 1 (Fragment)	SPTBN1	-0.4	-0.7	-0.7	-0.3	-0.3	-0.2	-0.3	0.3	109	115
I3L9F1	PIG Uncharacterized protein (Fragment)	BSG	-0.6	0.3	-0.6	-1.3	-0.4	-0.1	-0.5	-0.5	6	7
Q9H993	HUMAN UPF0364 protein C6orf211	C6orf211	-0.2	-0.6	-0.7	-0.7	0.9	0.6	-1.2	-2.6	1	2
G8JE99	HUMAN Cytochrome c oxidase subunit 2	COX2	-0.1	0.2	-1.0	-1.3	-0.7	-1.1	-2.6	-1.7	1	1
P34896	HUMAN Serine hydroxymethyltransferase, cytosolic	SHMT1	-0.7	-0.8	-0.5	-0.2	0.0	1.5	1.6	-0.4	1	1
F1RZ82	PIG Uncharacterized protein (Fragment)	LOC100526132	-1.2	-0.5	-0.4	-0.1	1.6	0.2	0.4	0.0	3	3
F1SQL0	PIG Uncharacterized protein (Fragment)	PNPT1	-0.4	-0.7	-0.5	-0.5	0.5	1.1	1.7	1.5	2	1
P26044	PIG Radixin	RDX	-1.1	-1.2	-0.3	0.5	-0.1	0.3	1.0	1.3	11	8
F1SHS4	PIG Uncharacterized protein	ERC1	-0.6	-1.4	-0.5	0.3	0.3	0.1	1.3	1.2	1	1
I3LK34	PIG Uncharacterized protein	LOC100625244	0.0	-0.1	-1.0	-1.1	0.0	-0.3	0.0	-1.1	4	6
I3LF22	PIG Integrin beta	ITGB5	-0.8	-0.6	-0.4	-0.3	-0.2	0.3	0.0	-0.9	1	1
P42174	PIG Glutamate dehydrogenase 1, mitochondrial (Fragments)	GLUD1	-0.8	-0.8	-0.5	-0.1	1.4	0.4	-0.9	0.0	1	1
Q9BTT0	HUMAN Acidic leucine-rich nuclear phosphoprotein 32 family member E	ANP32E	-2.0	-1.2	0.1	0.9	2.5	1.9	-0.5	-1.2	2	1
F1SBM1	PIG Uncharacterized protein	MCM8	-0.3	1.0	-1.5	-1.3	3.0	2.3	0.3	-3.6	1	1
K7GP62	PIG Uncharacterized protein	NA	-0.4	-1.0	-0.6	-0.2	0.4	-0.1	0.3	-0.6	5	2
Q2EN74	PIG Epidermal fatty acid-binding protein	FABP5	-1.1	-1.1	-0.7	0.8	-0.9	-0.6	-0.7	-0.1	7	5
I3LIB6	PIG Uncharacterized protein (Fragment)	LOC100156903	-0.1	-0.6	-0.8	-0.7	-1.0	-0.2	-0.5	-0.4	1	1
F2Z5K3	PIG Uncharacterized protein	RAP1A	-0.7	-0.5	-0.5	-0.6	1.7	-0.2	-0.9	-0.2	1	3
Q6IQ22	HUMAN Ras-related protein Rab-12	RAB12	-0.3	-0.5	-0.5	-0.9	0.2	1.5	1.0	0.8	2	1
F1SEQ3	PIG Uncharacterized protein	GHITM	-0.7	-0.6	-0.3	-0.6	-0.8	-0.5	-0.8	-0.2	4	4
F1S5J3	PIG Uncharacterized protein (Fragment)	NRAP	-1.2	-0.9	-0.1	0.0	-0.4	-0.1	0.0	-0.4	25	28
F1S597	PIG Uncharacterized protein	CCDC151	0.1	-0.3	-1.0	-0.9	0.1	-0.2	-0.8	-0.7	1	1
Q9Y4G6	HUMAN Talin-2	TLN2	0.1	-0.9	-0.8	-0.7	-0.3	-0.5	-0.4	-0.4	15	23
F1S798	PIG Uncharacterized protein	LOC100522817	-0.4	-0.2	-0.7	-1.0	1.2	1.0	-1.0	-0.8	1	1
Q8MJG6	PIG ATP synthase gamma subunit 1 (Fragment)	NA	0.3	-0.1	-1.2	-1.2	-0.6	-0.8	1.3	1.3	4	8
A0ZVR1	PIG Mitochondrial antiviral signaling protein	Mavs	-0.3	-0.4	-0.7	-0.8	0.3	1.1	1.2	1.6	7	10
A1XQR7	PIG Protein QIL1	QIL1	-0.3	0.0	-0.8	-1.1	-0.7	-1.1	-1.2	0.0	4	6
F1SK45	PIG Uncharacterized protein	AK3	-0.3	-0.2	-0.8	-0.9	0.1	0.1	-0.3	0.0	10	14
P83881	HUMAN 60S ribosomal protein L36a	RPL36A	-1.8	-2.1	-0.3	2.0	-1.5	-1.3	0.6	1.9	1	1
F1RP25	PIG Uncharacterized protein (Fragment)	CLYBL	0.0	-0.7	-0.9	-0.6	0.2	-0.2	-1.2	-0.3	3	2
I3LQK3	PIG 40S ribosomal protein S19	RPS19	-1.1	-0.7	0.2	-0.6	-1.4	-0.5	-0.2	0.3	2	2
F1SB42	PIG Uncharacterized protein (Fragment)	EZR	-1.1	-0.8	-0.2	-0.1	0.0	0.3	1.2	1.9	13	11
I3LLX3	PIG Uncharacterized protein	CAMSAP2	0.5	-0.6	-0.9	-1.2	-0.3	0.4	0.6	0.3	1	1
P23471	HUMAN Receptor-type tyrosine-protein phosphatase zeta	PTPRZ1	0.0	-0.1	-1.0	-1.1	-2.4	-1.6	-1.0	0.9	2	1
F1S192	PIG Uncharacterized protein	PFDN2	-0.3	-0.3	-0.8	-0.8	2.1	3.5	0.8	-1.0	2	1
F1RRS3	PIG Uncharacterized protein (Fragment)	NSF	-0.4	-0.9	-0.6	-0.3	-0.1	-0.1	-0.4	-0.4	6	2
F1S4X7	PIG Uncharacterized protein	MYH7B	0.3	-0.3	-1.0	-1.2	0.5	0.5	-0.8	-0.2	11	9
Q9XSD9	PIG Decorin	DCN	-1.7	-0.5	-0.5	0.5	-0.8	-0.8	-0.6	-0.1	14	11
F1S8U4	PIG Uncharacterized protein	MRPL43	-0.2	-0.5	-0.9	-0.7	4.0	3.7	0.3	1.0	3	2
I3LUR5	PIG Uncharacterized protein (Fragment)	ACAD9	0.2	-0.6	-1.0	-0.8	0.0	-0.4	0.3	-0.3	7	10

F1SMQ3	PIG Uncharacterized protein	SMO	-0.7	-0.1	-0.8	-0.6	-0.5	-0.3	0.2	0.4	1	1
A2RU54	HUMAN Homeobox protein HMX2	HMX2	-0.7	-0.6	-0.6	-0.5	-2.1	-1.7	-1.4	0.0	1	1
F1S5Y7	PIG Uncharacterized protein	CLCC1	-0.9	-0.7	-0.4	-0.3	-1.2	-0.1	-0.6	0.4	3	4
I3LGX8	PIG Chordin	CHRD	-0.1	-0.4	-0.7	-1.1	0.2	0.1	0.0	0.0	1	1
I3LAQ6	PIG Uncharacterized protein (Fragment)	ABHD14B	-0.4	-1.0	-0.8	-0.1	-0.3	-0.9	0.5	0.2	2	2
I3L6T2	PIG Uncharacterized protein (Fragment)	UBE2V2	-1.0	-0.7	-0.3	-0.3	-0.4	-0.6	0.1	-0.4	4	2
F1SR64	PIG Uncharacterized protein	MRPL44	0.2	-0.7	-0.8	-1.0	0.2	0.1	0.7	-2.0	1	1
F1RHN2	PIG Uncharacterized protein	ARVCF	0.1	-0.6	-0.8	-0.9	0.2	-0.5	-0.3	-0.9	8	13
Q9NX40	HUMAN OCIA domain-containing protein 1	OCIAD1	0.0	-0.1	-1.0	-1.2	-0.7	-1.5	-3.7	-3.2	1	1
F1RQH7	PIG Uncharacterized protein (Fragment)	LIMS2	-1.6	-1.6	-0.1	1.0	-0.6	-0.8	0.8	0.3	1	2
Q9NQT8	HUMAN Kinesin-like protein KIF13B	KIF13B	0.1	-0.6	-1.0	-0.7	-0.4	0.6	0.3	0.8	1	1
M3VK21	PIG Solute carrier family 25 (Aspartate/glutamate carrier), member 12	SLC25A12	-0.3	-0.4	-0.7	-0.9	-1.3	-1.4	-1.5	-0.5	4	5
F1S086	PIG Uncharacterized protein (Fragment)	SLC25A12	0.1	-0.2	-0.9	-1.3	-1.1	-1.2	-1.6	-0.3	15	16
F1RPK0	PIG Histone H2A	LOC100154508	-1.4	-1.0	-0.3	0.4	-1.6	-1.0	-0.4	-0.1	2	2
P14866	HUMAN Heterogeneous nuclear ribonucleoprotein L	HNRNPL	-1.0	-1.2	-0.4	0.2	-0.9	-0.2	0.3	0.0	5	5
F1S9V9	PIG Uncharacterized protein (Fragment)	TMEM38A	0.4	-1.0	-1.1	-0.6	-1.8	-0.9	-0.9	1.4	1	1
F1RY43	PIG Uncharacterized protein	PDP1	0.0	-0.6	-0.9	-0.9	1.2	0.3	1.0	-0.2	4	4
F1RUN1	PIG Uncharacterized protein (Fragment)	USP6NL	-0.7	-0.3	-0.7	-0.6	-1.5	-1.1	-0.7	0.2	1	1
I3LVQ8	PIG Uncharacterized protein (Fragment)	APCDD1	-1.4	0.3	-0.1	-1.2	-2.7	-1.6	-2.1	0.5	2	1
F1SC09	PIG Uncharacterized protein	ABCB5	-0.4	-1.1	-0.5	-0.3	-0.1	-0.1	1.1	1.7	2	1
I3LBZ5	PIG Uncharacterized protein	SCARF2	-1.1	-1.3	-0.2	0.3	0.1	-1.0	-0.4	-1.0	1	1
F1RV77	PIG Uncharacterized protein (Fragment)	RANBP9	0.0	-0.7	-0.8	-0.8	-0.3	0.2	-0.5	-1.2	1	1
K7GML0	PIG Uncharacterized protein	ADHFE1	0.5	-0.4	-1.1	-1.3	0.9	0.0	0.6	-1.5	2	4
I3LGX5	PIG Collagen, type IV, alpha 3 (Goodpasture antigen) binding protein tv2	COL4A3BP	-1.4	-1.2	-0.2	0.4	-1.2	-0.8	-1.2	-0.1	1	1
F1S415	PIG Uncharacterized protein	BAG3	-1.3	-0.3	-0.3	-0.4	2.2	1.8	0.3	0.4	17	11
I3LIL5	PIG Uncharacterized protein	HMGCL	-0.1	-0.5	-0.9	-0.8	0.2	0.7	0.0	0.5	6	6
I3LVC1	PIG Uncharacterized protein (Fragment)	NA	-1.9	-1.5	-0.1	1.2	-1.7	-1.0	-0.6	-0.4	1	1
Q8NCM8	HUMAN Cytoplasmic dynein 2 heavy chain 1	DYNC2H1	-0.2	-0.8	-0.8	-0.6	-0.7	-1.8	0.3	0.1	1	2
Q92886	HUMAN Neurogenin-1	NEUROG1	-0.2	-0.4	-0.9	-0.9	-0.5	-0.9	-0.6	-0.5	1	1
F1S4P7	PIG Uncharacterized protein	NDUFAF7	-0.1	-0.8	-0.7	-0.8	-0.4	-0.2	-1.6	-1.1	1	6
Q2VTP6	PIG Peptidyl-prolyl cis-trans isomerase	LOC654323	-0.7	-0.7	-0.5	-0.4	-0.1	-0.5	-0.3	0.1	2	2
B2ZPK1	PIG Cytochrome b reductase 1	CYBRD1	-1.3	-1.0	-0.3	0.3	1.9	1.6	2.5	3.6	1	2
F2WE98	HUMAN NADH-ubiquinone oxidoreductase chain 4	ND4	0.1	-0.2	-1.0	-1.3	-0.1	-0.1	-1.6	-1.2	1	2
F1S8C1	PIG Uncharacterized protein	HSPA12B	-0.8	-1.0	-0.8	0.1	0.2	-0.8	-0.8	-0.5	1	3
F1RIY7	PIG Uncharacterized protein	NA	-0.6	-1.2	-0.6	0.1	0.7	1.3	0.8	-0.8	1	4
F1S9T0	PIG Uncharacterized protein (Fragment)	DNAJB4	-0.3	-0.7	-0.7	-0.7	-0.4	0.5	0.1	-0.3	8	6
F1S5S1	PIG Uncharacterized protein	IBA57	0.1	0.4	-1.3	-1.6	0.6	0.4	0.3	-1.1	1	4
F1SPD6	PIG Uncharacterized protein (Fragment)	FBXO40	-0.3	-0.9	-0.6	-0.6	0.3	0.1	0.6	-0.6	1	2
Q13948	HUMAN Protein CASP	CUX1	-0.7	-0.9	-0.5	-0.3	1.3	1.0	0.1	0.8	1	1
I3LDC3	PIG Uncharacterized protein	NDUFB10	0.1	0.1	-1.0	-1.6	0.0	0.2	-0.8	-1.5	8	8

Q9UMD9	HUMAN Collagen alpha-1(XVII) chain	COL17A1	-1.3	-0.2	0.2	-1.0	0.4	0.4	0.8	0.1	1	1
I3L6F1	PIG 60S ribosomal protein L18	RPL18	-1.5	-1.9	-0.1	1.1	-0.6	1.3	0.0	1.1	1	1
P53603	PIG Formimidoyltransferase-cyclodeaminase	FTCD	0.0	-0.8	-0.6	-1.0	-0.8	-1.4	-1.1	0.0	1	1
I3LGM4	PIG Uncharacterized protein	NDUFA11	0.2	-0.2	-1.0	-1.4	-0.4	0.1	-0.3	-0.5	2	2
I3LVP5	PIG Uncharacterized protein	PPP1R12A	-0.9	-1.4	-0.4	0.4	-0.5	-0.2	0.1	-0.1	4	2
F1SB03	PIG Uncharacterized protein	SRCIN1	0.1	-0.2	-1.1	-1.3	0.4	1.3	-1.1	-2.7	2	2
D3DTL4	HUMAN Enolase	ENO3	-0.2	-1.0	-0.8	-0.5	1.7	1.0	0.8	-0.6	1	1
Q6PLK3	PIG Amine oxidase [flavin-containing] B	MAOB	-0.2	-0.5	-0.9	-0.8	-0.3	-0.9	0.0	1.2	7	10
F1S2G3	PIG Uncharacterized protein (Fragment)	TBCA	-1.9	-1.1	0.1	0.4	0.3	0.3	0.3	0.2	3	1
Q08AH1	HUMAN Acyl-coenzyme A synthetase ACSM1, mitochondrial	ACSM1	-0.4	-0.2	-0.6	-1.1	-1.1	-1.0	-0.2	0.3	1	2
I3LNE1	PIG Uncharacterized protein (Fragment)	LRRFIP2	-0.8	-0.9	-0.5	-0.2	0.6	0.9	0.3	0.1	3	5
F1S2G4	PIG Uncharacterized protein	KAT6B	-0.8	-1.1	-0.4	-0.1	-0.8	0.4	-0.5	0.0	1	1
Q09666	HUMAN Neuroblast differentiation-associated protein AHNAK	AHNAK	-1.2	-0.6	-0.6	0.0	0.9	2.4	2.2	2.0	42	32
F1RXE6	PIG Uncharacterized protein	SMARCE1	0.1	-0.8	-0.9	-0.8	1.6	1.7	0.4	0.2	1	1
P17152	HUMAN Transmembrane protein 11, mitochondrial	TMEM11	-0.3	-0.8	-0.8	-0.6	-0.5	-0.2	-0.8	-0.4	1	2
Q95339	PIG ATP synthase subunit f, mitochondrial	ATP5J2	0.0	0.2	-1.0	-1.7	-0.8	-0.1	-1.8	-1.3	2	2
F1SI15	PIG Uncharacterized protein	PRPF39	-1.3	-0.9	-0.4	0.1	-2.3	-2.1	-0.8	-1.8	1	1
A8K482	HUMAN Aspartate aminotransferase	NA	-1.1	0.2	-0.1	-1.5	-1.1	-1.6	-1.0	-0.8	2	2
F1S4N5	PIG Uncharacterized protein	SFXN4	-0.2	-0.6	-0.9	-0.8	0.2	-0.1	-0.6	1.2	3	3
I3LQN4	PIG Uncharacterized protein	PHB2	-0.4	-0.5	-0.7	-0.8	-1.4	-1.0	-1.0	0.3	13	15
Q3Y5G5	PIG Peroxisomal enoyl coenzyme A hydratase 1	ECH1	-0.6	-0.2	-0.7	-0.9	-1.8	-1.3	-0.5	-0.3	6	3
F1S1F4	PIG Uncharacterized protein	WNK4	-0.5	-0.9	-0.6	-0.4	-1.2	-1.4	-1.3	-0.9	4	2
Q95J99	PIG Laminin beta 2 chain (Fragment)	NA	-0.7	-0.6	-0.5	-0.6	0.7	-1.0	-1.2	-0.5	1	1
Q7YS91	PIG Protein TBRG4	TBRG4	-0.5	-0.7	-0.6	-0.6	1.1	0.0	0.5	0.4	2	2
F1STT2	PIG Uncharacterized protein	SYTL2	-1.2	-0.7	-0.2	-0.3	-0.5	2.5	0.2	0.0	1	1
F1S8P1	PIG Uncharacterized protein	SCCPDH	0.1	-1.0	-0.9	-0.7	-0.2	-0.6	-1.3	-1.2	5	4
F1S738	PIG Uncharacterized protein (Fragment)	ADGB	-0.4	-0.8	-0.5	-0.7	-0.6	-0.7	0.3	0.6	1	3
F1SCS1	PIG Uncharacterized protein	DDX1	-1.0	-1.0	-0.5	0.1	-0.1	0.2	-0.4	-0.2	6	6
F1RPD2	PIG Uncharacterized protein	LOC100620271	-0.2	0.3	-0.8	-1.8	-0.7	-0.2	-1.3	-1.5	11	11
F1RGE3	PIG NADH dehydrogenase [ubiquinone] 1 alpha subcomplex subunit 2	LOC100520950	0.1	0.1	-1.0	-1.6	-0.7	-0.7	-1.4	0.6	1	1
Q16281	HUMAN Cyclic nucleotide-gated cation channel alpha-3	CNGA3	-0.4	-1.5	-0.5	0.0	0.3	0.2	-0.2	1.4	1	1
F1SEQ4	PIG Uncharacterized protein (Fragment)	NRG3	-0.5	-0.8	-0.6	-0.6	-0.1	-1.1	-0.5	-1.2	1	2
F1ST39	PIG Uncharacterized protein	FXR2	-0.9	-1.1	-0.4	-0.1	1.2	0.7	0.5	0.4	2	1
Q96A19	HUMAN Coiled-coil domain-containing protein 102A	CCDC102A	-0.3	-1.0	-0.8	-0.3	1.4	0.3	0.9	1.9	1	1
F1RFA5	PIG Uncharacterized protein (Fragment)	PGP	-0.3	-0.9	-0.7	-0.6	0.5	1.3	0.6	0.5	4	5
F1RJ25	PIG Fructose-bisphosphate aldolase	ALDOC	-0.2	-1.2	-0.6	-0.4	0.9	1.1	0.9	1.5	8	10
I3LJT3	PIG Adenylyl cyclase-associated protein (Fragment)	NA	-0.2	-1.0	-0.6	-0.7	-0.5	-0.1	-0.1	-0.8	2	3
F1SJC2	PIG Uncharacterized protein	PPP1R3A	-0.7	-1.1	-0.3	-0.3	-0.3	0.6	0.4	0.1	1	1
Q14195	HUMAN Dihydropyrimidinase-related protein 3	DPYSL3	-0.9	-1.3	-0.5	0.2	0.1	0.2	0.7	0.3	7	5
O46560	PIG Pyridoxal kinase	PDXK	-1.1	-0.7	-0.3	-0.4	1.4	2.5	0.3	0.6	2	2

Q14103	HUMAN Heterogeneous nuclear ribonucleoprotein D0	HNRNPD	-1.3	-0.9	-0.3	0.0	0.3	0.5	0.0	-0.5	5	3
Q9TV77	PIG 130 kDa regulatory subunit of myosin phosphatase (Fragment)	NA	-0.6	-1.3	-0.6	0.0	-0.2	-1.0	0.0	1.0	3	1
Q29096	PIG Proteasome subunit C9-like protein (Fragment)	NA	-1.6	-1.1	-0.1	0.2	-0.5	-0.5	1.0	-0.4	5	4
P10323	HUMAN Acrosin	ACR	-0.9	-0.2	-1.0	-0.4	-0.4	0.1	1.8	0.7	1	1
F1S156	PIG Uncharacterized protein	SYNPO2	0.1	-0.8	-1.0	-0.8	0.6	0.5	0.5	0.7	4	11
F1RSI2	PIG Uncharacterized protein	RP1	-0.6	-0.3	-0.9	-0.7	0.8	0.2	-0.2	0.2	1	1
Q6P4F7	HUMAN Rho GTPase-activating protein 11A	ARHGAP11A	-0.6	-0.6	-0.6	-0.7	-0.5	0.0	-0.1	0.7	3	1
Q8WXI4	HUMAN Acyl-coenzyme A thioesterase 11	ACOT11	-0.1	-0.6	-0.9	-0.9	-0.1	-0.5	-1.2	-0.2	1	1
I3LNF1	PIG Uncharacterized protein	FAM208B	-0.3	-0.1	-1.1	-1.1	0.1	0.1	0.2	0.4	2	1
Q9Y2J2	HUMAN Band 4.1-like protein 3	EPB41L3	-0.7	-0.6	-0.3	-1.0	1.3	0.7	0.5	0.4	3	4
Q8TAB5	HUMAN UPF0500 protein C1orf216	C1orf216	-0.6	-1.3	-0.5	-0.2	0.0	-0.2	1.0	1.2	1	1
I3LK72	PIG Uncharacterized protein (Fragment)	ACSF3	0.0	-0.7	-1.0	-0.8	-1.0	-0.8	-0.8	-0.9	5	12
Q197W4	PIG Cyclin-dependent kinase 5	CDK5	-0.9	-1.0	-0.6	0.0	-1.3	-0.1	-1.6	-0.3	1	1
F1S2Y2	PIG Uncharacterized protein	PROX1	-0.4	-0.8	-0.7	-0.5	0.9	0.7	0.4	-0.3	1	1
Q86VS8	HUMAN Protein Hook homolog 3	HOOK3	-0.7	-0.4	-0.5	-0.9	-0.8	-1.5	-2.1	-1.3	4	4
A6NM43	HUMAN Putative T-complex protein 1 subunit theta-like 1	CCT8L1P	0.2	-0.3	-1.0	-1.3	0.1	-0.6	0.6	-0.9	1	1
F1S285	PIG Uncharacterized protein	COL14A1	-1.3	-1.1	-0.8	0.7	-1.9	-1.2	-0.6	0.4	3	4
Q9GLG4	PIG Secretogranin-1	CHGB	-1.9	-0.3	-0.2	-0.1	1.0	5.2	-0.2	0.5	1	1
I3LJU1	PIG Uncharacterized protein (Fragment)	SURF4	-0.7	-0.4	-1.0	-0.6	-0.3	2.9	1.1	0.8	2	1
I3L7Z2	PIG Uncharacterized protein (Fragment)	PRSS46	-0.7	-1.5	-0.4	0.1	0.0	0.3	-0.4	0.4	1	1
H0YEI7	HUMAN Eukaryotic translation initiation factor 4 gamma 2 (Fragment)	EIF4G2	0.1	-1.2	-0.8	-0.6	1.0	2.1	0.9	-0.5	1	1
F1S3I9	PIG Uncharacterized protein	ACOT4	-0.7	-0.6	-0.9	-0.3	-1.3	-0.8	-0.6	0.1	1	2
Q96JG9	HUMAN Zinc finger protein 469	ZNF469	-0.9	-0.3	-0.9	-0.4	-0.4	-1.5	0.1	1.6	1	1
F1S618	PIG Uncharacterized protein (Fragment)	NA	0.6	-1.2	-1.1	-0.9	-0.8	0.5	-0.5	0.8	1	1
Q9NPL8	HUMAN Complex I assembly factor TIMMDC1, mitochondrial	TIMMDC1	-0.2	-0.9	-0.7	-0.7	-0.2	0.4	0.5	1.2	1	1
F1RP34	PIG NAD(P)H-hydrate epimerase	APOA1BP	-0.4	-0.8	-0.9	-0.5	0.7	-0.1	0.6	0.8	1	1
Q00722	HUMAN 1-phosphatidylinositol 4,5-bisphosphate phosphodiesterase beta-2	PLCB2	1.5	-1.1	-1.7	-1.3	3.2	-3.2	-4.9	-3.7	1	1
F1SHR6	PIG Uncharacterized protein (Fragment)	NA	0.0	-0.5	-1.1	-1.1	-0.2	-0.4	-1.4	-1.8	2	2
Q06AA9	PIG Ubiquitin-conjugating enzyme E2 D2	UBE2D2	-1.0	-1.2	-0.4	-0.1	0.6	0.5	-0.4	0.3	2	2
F1SE83	PIG Uncharacterized protein	OCIAD1	-0.4	-0.8	-0.8	-0.6	0.8	0.6	0.1	0.1	4	4
F1S6Q1	PIG Uncharacterized protein	NDUFA13	-0.2	-0.2	-1.0	-1.3	-1.1	-0.7	-1.4	-0.5	11	13
F1S6K2	PIG Uncharacterized protein	JPH3	-0.9	-0.8	-0.4	-0.5	-1.1	-0.6	-0.7	-0.1	1	1
Q9MZ16	PIG Voltage-dependent anion-selective channel protein 1	VDAC1	-0.6	-0.1	-0.7	-1.1	-1.5	-1.0	-0.9	-0.9	16	16
F1SFE2	PIG Uncharacterized protein	NA	-1.2	-0.7	-0.3	-0.3	-0.9	-0.5	1.1	1.4	2	2
F1RP45	PIG Uncharacterized protein (Fragment)	CISD1	-1.3	-0.5	-0.6	-0.1	0.1	0.3	0.4	0.6	1	1
P24540	PIG Acylphosphatase-1	ACYP1	-0.8	-0.9	-0.5	-0.4	-0.9	-0.9	-0.9	-1.8	2	2
Q9BDE7	PIG Activated leukocyte cell adhesion molecule (Fragment)	ALCAM	-0.9	-0.5	-0.6	-0.7	-1.0	-1.4	-1.6	-1.3	3	2
K9J6L1	PIG Solute carrier family 12 member 7	SLC12A7	-0.9	-0.8	-0.5	-0.4	-1.3	-0.5	0.4	0.7	4	2
Q3HUX1	PIG Fatty acid translocase/CD36	CD36	-0.7	-0.2	-0.8	-1.0	-0.6	0.3	1.3	1.5	8	9
F1RJJ3	PIG Uncharacterized protein	SBDS	-0.5	-0.9	-0.7	-0.4	0.1	1.0	-0.1	0.8	5	4

F1RH41	PIG Uncharacterized protein (Fragment)	LOC100524748	-0.8	-0.1	-1.0	-0.7	1.3	0.3	-0.6	-0.2	2	6
I3LAB4	PIG Uncharacterized protein	ATPAF2	-0.1	-0.8	-1.0	-0.7	0.7	0.0	-0.7	-0.3	1	1
F1S9I9	PIG MOSC domain-containing protein 2, mitochondrial	MARC2	-0.2	-0.8	-0.8	-0.7	-0.4	-0.1	0.2	0.6	5	4
F1RW08	PIG Signal recognition particle subunit SRP68	SRP68	-2.4	-1.7	0.2	1.3	-1.5	-0.7	-0.6	1.2	2	1
F1S764	PIG Uncharacterized protein	CPT2	0.0	-0.8	-0.9	-0.8	-0.3	-0.7	0.3	-0.3	9	14
F1RPV1	PIG Uncharacterized protein (Fragment)	TIPRL	-0.7	-1.3	-0.3	-0.3	-1.4	-1.5	-1.7	-1.0	4	3
I3LSM2	PIG Uncharacterized protein	A4GNT	-0.3	-1.3	-0.8	-0.2	0.4	0.6	0.5	-0.3	1	1
M9TGS8	PIG Mitochondrial delta3,delta2-dienoyl-CoA isomerase	Eci1	0.2	-0.2	-1.3	-1.3	-0.5	-1.0	-1.3	0.0	4	5
P47788	PIG Thimet oligopeptidase	THOP1	-1.0	-1.0	-0.4	-0.3	-1.7	-1.2	-0.8	-0.4	3	1
P63208	HUMAN S-phase kinase-associated protein 1	SKP1	-0.9	-0.9	-0.6	-0.2	2.3	0.9	-0.2	-2.5	5	2
Q9BXJ8-2	HUMAN Isoform 2 of Transmembrane protein 120A	TMEM120A	-0.9	-1.2	-0.7	0.1	-0.3	0.4	0.2	-0.5	1	2
Q9N251	PIG Titin (Fragment)	TTN	-0.3	-1.0	-0.6	-0.7	0.7	1.0	2.9	1.0	6	7
Q2EHH7	PIG Serine/threonine-protein phosphatase	NA	-0.9	-1.3	-0.5	0.0	-0.5	-0.1	-1.0	-1.0	3	3
Q7M2Y3	PIG Nonhistone chromosomal protein HMG-14 (Fragment)	NA	-1.5	-1.3	-0.6	0.8	0.2	0.5	-0.1	2.5	2	2
F1RI72	PIG Uncharacterized protein	GOLGA3	-1.6	-1.2	-0.4	0.5	0.6	-0.5	0.0	1.7	2	1
Q96NG3	HUMAN Tetratricopeptide repeat protein 25	TTC25	-0.6	-1.2	-0.5	-0.4	-0.8	-0.4	-0.8	0.3	1	1
Q09138	PIG 5'-AMP-activated protein kinase subunit gamma-1	PRKAG1	-0.2	-0.8	-0.8	-0.8	-0.6	-0.6	-0.4	0.0	3	2
F1RF21	PIG Uncharacterized protein	ANKRD34B	-1.0	-0.9	-0.4	-0.4	0.8	0.3	0.6	0.5	1	1
F1RYS7	PIG Uncharacterized protein (Fragment)	SHROOM3	-0.4	-0.6	-0.6	-1.1	-1.8	-2.2	-0.6	-0.6	2	2
I3LQZ2	PIG Histone H2A (Fragment)	H2AFY2	-1.5	-0.8	0.1	-0.5	-1.4	-0.8	-1.4	0.6	1	2
Q5D144	PIG Transcription factor A, mitochondrial	TFAM	-0.3	-0.7	-0.8	-0.9	-0.1	-0.9	-0.7	0.3	9	7
Q9Y5U8	HUMAN Mitochondrial pyruvate carrier 1	MPC1	-0.6	-1.2	-0.5	-0.4	-1.7	-1.5	0.2	0.7	3	4
F1RIA7	PIG Uncharacterized protein	TMEM109	-0.8	-0.7	-0.7	-0.5	-1.6	-1.1	-0.4	1.6	4	4
I3LIU0	PIG Uncharacterized protein (Fragment)	NA	-1.2	-1.0	-0.5	0.0	-0.1	1.8	1.7	2.1	6	4
Q96JM2	HUMAN Zinc finger protein 462	ZNF462	-0.3	-0.5	-0.9	-1.0	0.8	-0.6	-2.4	-1.9	1	2
F1SUP1	PIG Uncharacterized protein	AKR7A2	-1.2	-1.1	-0.3	-0.1	-0.6	-1.0	-0.5	1.0	5	4
D5FFT2	PIG Cardiomyopathy-associated 4 (Fragment)	NA	-0.6	-0.4	-0.8	-0.9	0.5	1.6	1.9	-0.6	1	1
F1SDZ8	PIG Uncharacterized protein	SEL1L	-0.6	0.0	-1.0	-1.0	1.3	1.6	-0.2	-0.9	2	1
F1RMC9	PIG Uncharacterized protein	NEK5	-0.2	-1.0	-0.7	-0.9	2.4	0.5	1.8	0.3	2	1
F1RT67	PIG Inositol monophosphatase 3	IMPAD1	0.2	-1.4	-0.9	-0.6	-0.7	-1.0	-0.9	0.9	2	1
D6MJK1	HUMAN MHC class I antigen (Fragment)	HLA-C	-1.7	0.4	-0.5	-0.9	-0.7	-0.1	0.2	0.5	1	1
P04178	PIG Superoxide dismutase [Cu-Zn]	SOD1	-1.4	-0.6	-0.5	-0.3	2.5	0.8	0.0	-0.9	5	5
I3LF34	PIG Uncharacterized protein (Fragment)	TRDMT1	-0.7	-0.4	-1.1	-0.5	-1.8	-2.4	-4.3	-0.9	1	1
F1SNT1	PIG Uncharacterized protein	ACAD11	-0.2	-1.0	-0.9	-0.6	-1.3	-1.8	-1.2	-1.2	1	1
F1SMZ6	PIG Uncharacterized protein	HSPE1	-0.3	0.0	-1.4	-1.0	0.6	-0.3	1.1	1.3	8	7
Q28948	PIG 5'-AMP-activated protein kinase catalytic subunit alpha-2	PRKAA2	0.0	-0.2	-0.9	-1.6	-2.6	-0.3	0.7	1.5	1	2
K7GNY9	PIG Uncharacterized protein	VBP1	-0.8	-1.2	-0.6	-0.1	0.8	0.7	-0.4	-0.3	4	1
I3L997	PIG Uncharacterized protein	NA	-0.2	-0.8	-1.0	-0.7	0.3	-0.5	-1.8	-0.8	3	2
F1SN52	PIG Uncharacterized protein	HIBCH	-0.8	-0.6	-0.7	-0.7	0.1	-0.6	-1.0	-1.1	2	2
O95865	HUMAN N(G),N(G)-dimethylarginine dimethylaminohydrolase 2	DDAH2	-0.1	-0.9	-1.1	-0.6	0.8	0.4	0.0	0.2	5	5

F1SLX5	PIG Uncharacterized protein	AASS	-0.1	-0.7	-1.0	-1.0	-0.2	-0.2	-1.0	-0.3	3	16
F1SLQ3	PIG Uncharacterized protein (Fragment)	NA	-0.1	0.1	-1.1	-1.7	0.1	0.8	-0.1	1.2	5	8
I3LLY8	PIG Uncharacterized protein	KRT79	-0.6	-0.4	-0.8	-0.9	-1.1	-2.2	0.0	6.9	4	4
I3LRR4	PIG Uncharacterized protein (Fragment)	NDUFV3	-0.8	-0.2	-0.8	-0.9	0.6	-0.4	-0.5	-0.1	14	13
F1SMT0	PIG Uncharacterized protein (Fragment)	EFHD1	-0.2	-0.1	-1.0	-1.5	0.9	1.0	0.0	0.6	2	1
F1SSF9	PIG Uncharacterized protein	MYL7	0.5	-0.4	-1.3	-1.6	2.4	0.0	-0.7	-1.8	3	3
I3LAC5	PIG Uncharacterized protein (Fragment)	NA	-1.3	-1.0	-0.4	0.0	-0.5	-0.7	0.4	1.8	1	1
I3LJ13	PIG Uncharacterized protein	CLPB	-1.0	-0.3	-0.7	-0.7	-1.6	-2.0	-0.7	-0.5	1	1
A9LM01	PIG Progesterone receptor membrane component 2	PGRMC2	-0.9	-0.9	-0.5	-0.5	-0.3	0.4	0.6	0.9	8	7
F1SAJ9	PIG Uncharacterized protein (Fragment)	MAPRE2	-1.0	-1.2	-0.6	0.0	-1.6	-0.9	-1.5	-2.2	4	2
I3LLI8	PIG Uncharacterized protein (Fragment)	YWHAE	-0.9	-1.2	-0.5	-0.1	-0.5	-0.5	0.1	-0.9	12	9
B2R4A2	HUMAN Cytochrome b-c1 complex subunit 7	NA	-0.3	-0.1	-1.2	-1.2	-0.1	0.4	0.4	0.3	2	1
Q96PZ0	HUMAN Pseudouridylate synthase 7 homolog	PUS7	-0.5	-1.1	-0.8	-0.3	-0.8	0.5	-0.5	-1.2	1	1
F1SDX0	PIG Uncharacterized protein	ERCC6	-0.1	0.0	-1.3	-1.4	0.4	0.6	-0.9	-1.2	1	1
I3LA45	PIG Uncharacterized protein	LOC100625120	-0.7	-0.6	-0.8	-0.6	-0.1	-0.3	0.7	0.0	1	1
F1RZA9	PIG Uncharacterized protein	AZI1	0.7	-0.1	-1.6	-1.8	-1.5	-1.4	-1.4	-0.3	1	2
Q6ZMR3	HUMAN L-lactate dehydrogenase A-like 6A	LDHAL6A	-1.0	-0.8	-0.4	-0.6	0.6	-0.3	0.9	0.7	1	1
Q8WNV7	PIG Dehydrogenase/reductase SDR family member 4	DHRS4	-0.2	-0.4	-1.2	-1.0	-0.5	0.4	1.5	1.5	10	12
F1SPZ9	PIG Uncharacterized protein	APOO	-0.7	-0.5	-0.8	-0.7	-0.9	-0.8	-0.7	-0.1	6	6
B2MUB6	PIG Small calcium-binding mitochondrial carrier 1	SCAMC-1	-0.5	-0.7	-0.8	-0.8	-2.0	-3.1	-0.7	0.1	1	1
F1STS4	PIG Uncharacterized protein (Fragment)	ME3	-0.8	-0.4	-0.6	-1.0	-0.9	-0.9	-0.8	-0.7	5	10
Q95250	PIG Membrane-associated progesterone receptor component 1	PGRMC1	-0.8	-0.6	-0.9	-0.5	-0.5	0.2	-0.4	0.6	9	6
F1RNI5	PIG Uncharacterized protein	LOC100520312	0.5	-0.5	-1.1	-1.6	-0.8	-0.5	-1.5	-0.9	2	3
F1S4V8	PIG Uncharacterized protein	CPNE1	-0.6	-0.8	-0.8	-0.6	0.5	0.6	-1.2	-1.2	1	1
F1RQ84	PIG Uncharacterized protein (Fragment)	TN-X	-0.8	-1.1	-0.7	-0.2	-1.3	-0.8	0.2	-0.3	1	3
P0C267	PIG Coiled-coil domain-containing protein 127	CCDC127	-0.6	-0.9	-0.8	-0.6	1.4	1.4	0.6	0.0	1	1
F1RKJ9	PIG Uncharacterized protein	ISOC1	-0.3	-0.9	-1.0	-0.6	-0.5	-0.7	-0.1	-0.6	5	5
F1SSL9	PIG Uncharacterized protein	C14orf105	-0.5	-1.2	-0.8	-0.3	-0.5	-0.4	1.2	3.5	1	1
Q8N1F7	HUMAN Nuclear pore complex protein Nup93	NUP93	-0.1	-0.9	-1.0	-0.8	-0.1	-0.7	-0.5	-0.2	2	1
F1RHJ9	PIG Uncharacterized protein	GLOD4	-0.9	-0.8	-0.7	-0.5	-0.2	0.3	0.4	-0.4	9	8
Q9BWH6	HUMAN RNA polymerase II-associated protein 1	RPAP1	-0.8	-1.7	-0.5	0.2	-0.5	0.8	-0.2	0.6	1	1
F1RMR5	PIG Uncharacterized protein	ISOC2	-0.6	-0.5	-0.7	-1.1	0.7	-0.1	-0.5	-1.0	1	1
F1RYM4	PIG Uncharacterized protein	ACSS3	-0.4	-0.5	-1.1	-0.8	-0.2	0.6	-2.7	0.3	7	11
A0P8Z5	PIG BRCA1-A complex subunit RAP80	UIMC1	-0.3	-0.3	-1.1	-1.2	0.9	0.0	0.2	0.6	1	1
P49927	PIG Major prion protein	PRNP	-0.5	-0.6	-0.9	-0.8	-0.1	-0.4	0.5	0.5	1	1
F1RHM3	PIG Uncharacterized protein (Fragment)	RYR2	-0.4	-0.6	-0.9	-0.9	-0.7	-1.2	-1.3	-0.6	21	72
P0C2C3	PIG MOSC domain-containing protein 2, mitochondrial (Fragments)	MARC2	-0.4	-1.2	-0.9	-0.4	0.1	0.2	0.7	1.7	1	1
I3L8T3	PIG tRNA (guanine(37)-N1)-methyltransferase	TRMT5	-0.3	0.1	-1.0	-1.6	-1.2	-1.6	-1.2	-1.4	1	1
I3L5D5	PIG Uncharacterized protein	AHNAK	-1.5	-0.9	-0.7	0.2	0.5	2.1	1.9	2.1	65	61
O94826	HUMAN Mitochondrial import receptor subunit TOM70	TOMM70A	-0.8	-1.2	-0.7	-0.2	-1.2	-1.0	-1.0	0.7	7	10

R4JU57	PIG LSm14A	LSm14A	-0.6	-0.4	-1.0	-0.8	-0.8	-1.1	-0.5	-0.4	2	2
G5EA44	HUMAN Chromosome 12 open reading frame 43, isoform CRA	C12orf43	-0.6	-0.9	-0.7	-0.7	0.5	1.1	0.4	0.1	1	1
F1S227	PIG Uncharacterized protein	PTRH2	-1.0	-0.7	-0.7	-0.4	0.2	0.1	-1.1	-0.4	1	2
I3LHV8	PIG Uncharacterized protein (Fragment)	NA	-0.4	-0.8	-0.8	-0.8	1.3	1.8	0.8	0.1	2	1
F1RJM8	PIG Uncharacterized protein (Fragment)	ABHD11	-0.1	-0.5	-1.2	-1.1	0.1	-0.1	0.0	-0.5	5	5
F1SM39	PIG Uncharacterized protein	ALPK2	-0.5	-0.5	-1.0	-0.9	-0.9	-0.4	0.3	-0.1	1	2
F1S7V6	PIG Uncharacterized protein	PPP1R14C	0.0	-1.3	-0.9	-0.6	3.0	1.7	3.3	4.2	1	1
F1SSG1	PIG Uncharacterized protein	YKT6	-1.8	-2.1	0.0	0.9	0.8	1.0	0.4	0.5	2	1
O15078	HUMAN Centrosomal protein of 290 kDa	CEP290	-1.2	-1.3	-0.8	0.4	0.6	0.2	0.5	-1.6	1	1
F1SQG5	PIG Uncharacterized protein	COPS2	-0.4	-1.1	-0.8	-0.5	0.7	0.2	-1.1	-1.8	4	3
Q0QEM6	PIG ATP synthase subunit beta (Fragment)	ATP5B	0.2	0.2	-1.3	-2.0	0.8	0.2	0.4	-1.9	38	37
P07802	PIG cAMP-dependent protein kinase type I-alpha regulatory subunit	PRKAR1A	-0.6	-0.8	-0.8	-0.7	-0.1	0.5	-0.1	-0.4	15	11
F1RK50	PIG Uncharacterized protein (Fragment)	LOC100513918	-0.6	-0.2	-1.2	-0.9	-0.3	0.0	-1.1	0.1	1	3
F1RU84	PIG Uncharacterized protein	C6orf136	-0.8	-1.0	-0.6	-0.6	-1.2	-0.4	0.3	-0.5	2	1
F1SCK4	PIG Uncharacterized protein	NA	-0.7	-0.8	-1.1	-0.4	0.3	0.0	-0.6	0.4	1	1
F1S7T0	PIG Uncharacterized protein	MYOC	-0.7	-0.7	-0.8	-0.8	-1.7	-0.1	-2.2	0.0	1	1
I3LFI3	PIG Uncharacterized protein	NDRG2	0.1	-0.6	-1.2	-1.2	0.7	-0.2	-0.8	-0.5	6	6
F1RF63	PIG Uncharacterized protein	LOC100524945	-0.1	-1.3	-0.7	-0.8	-0.5	-0.9	-0.6	-0.6	1	1
F1SPF3	PIG Uncharacterized protein	MCM2	-1.8	2.1	-1.6	-1.7	-0.7	-0.4	-0.9	-0.9	1	1
I3LUD5	PIG Uncharacterized protein	UBXN1	-1.2	-0.8	-0.7	-0.3	0.2	-0.2	-1.3	-3.5	3	1
A7E1T5	PIG Putative uncharacterized protein (Fragment)	NA	-1.3	-1.3	-0.3	-0.1	0.9	0.9	0.3	1.1	3	1
Q9H867-2	HUMAN Isoform 2 of Protein-lysine methyltransferase METTL21D	VCPKMT	-0.5	-0.2	-0.8	-1.5	-2.0	-0.6	-0.2	0.6	1	1
Q5RLR3	PIG Heat shock 10kDa protein 1 (Fragment)	NA	-0.5	0.1	-1.4	-1.2	1.4	0.8	0.7	0.6	4	3
P15311	HUMAN Ezrin	EZR	-1.6	-1.7	-0.2	0.5	-0.4	-1.1	-0.1	1.1	4	5
P55268	HUMAN Laminin subunit beta-2	LAMB2	-0.8	-0.8	-0.7	-0.6	-2.0	-2.4	-1.8	-0.7	5	5
K7EQW8	HUMAN Tropomyosin alpha-4 chain (Fragment)	TPM4	0.3	0.8	-1.8	-2.4	3.3	1.6	-0.1	-3.1	2	1
I3LFC2	PIG Uncharacterized protein	DUT	-0.6	-1.1	-0.6	-0.7	-1.3	-0.7	-0.4	0.4	1	1
Q8WX94	HUMAN NACHT, LRR and PYD domains-containing protein 7	NLRP7	0.0	-0.4	-1.1	-1.5	-1.5	-1.5	-0.8	1.1	1	1
F1SKM0	PIG Uncharacterized protein	UQCRC1	-0.5	-0.2	-0.9	-1.4	-0.6	-0.7	-1.0	-1.7	22	20
F1S300	PIG Uncharacterized protein	TPR	-0.7	-1.0	-0.5	-0.8	1.3	1.2	0.1	0.8	2	2
M3VH65	PIG Mitochondrial mitofusin 1	MFN1	-0.6	-0.9	-1.0	-0.5	-0.3	0.6	-0.2	-1.2	1	1
P46779	HUMAN 60S ribosomal protein L28	RPL28	-1.3	-1.9	-0.4	0.6	-1.0	-0.1	0.8	1.5	3	3
F2Z514	PIG 40S ribosomal protein S27 (Fragment)	RPS27	-1.0	-0.9	-0.7	-0.4	0.4	0.1	0.1	1.0	3	1
A1XQS2	PIG Mitochondrial import receptor subunit TOM7 homolog	TOMM7	-0.8	-1.1	-0.6	-0.4	0.0	-0.4	-0.2	1.0	1	1
I3LHQ3	PIG Uncharacterized protein (Fragment)	NA	-0.9	-0.4	-0.8	-0.9	-0.4	0.0	0.1	-1.0	1	1
F1SDT0	PIG Uncharacterized protein (Fragment)	LOC100155350	-0.1	-0.8	-1.1	-1.0	-0.5	-1.0	-1.1	0.7	3	4
F1S847	PIG Uncharacterized protein	INA	-0.9	-1.2	-0.5	-0.5	-1.0	-0.9	2.4	-0.8	1	1
F1S6G5	PIG Uncharacterized protein	BTF3L4	-1.0	-1.2	-0.7	-0.1	-0.1	0.1	0.3	-0.4	1	1
Q7JFN4	PIG Calcium/calmodulin-dependent protein kinase II isoform gamma-B	CAMK2G	-0.7	-0.8	-0.9	-0.5	-0.1	0.7	-0.8	-0.9	3	6
Q8TES7	HUMAN Fas-binding factor 1	FBF1	-0.3	-0.6	-1.0	-1.2	-1.2	-0.8	-0.5	0.1	2	1

Q96Q04	HUMAN Serine/threonine-protein kinase LMTK3	LMTK3	-0.9	-1.2	-0.9	-0.1	-0.2	0.1	0.6	1.5	3	1
I3LNP9	PIG Uncharacterized protein (Fragment)	BZW2	-1.3	-1.1	-0.5	-0.1	-1.0	-0.6	-1.7	-0.6	4	4
I3LD20	PIG Dystroglycan	DAG1	-1.0	-0.4	-0.9	-0.8	-0.3	0.1	0.2	1.5	8	8
I3LCX3	PIG Uncharacterized protein	DUSP3	-0.5	-1.1	-0.7	-0.8	0.5	-0.2	-0.4	-0.8	6	6
B3VFA9	PIG Troponin I skeletal slow-twitch protein	TNNI1	-0.5	-0.7	-1.2	-0.8	2.0	0.7	-0.4	-0.7	1	3
B9TX04	HUMAN Mediator complex subunit MED16 variant MED16	MED16	0.6	-0.7	-1.1	-1.8	0.8	0.5	-0.5	-1.7	1	1
Q9UI47	HUMAN Catenin alpha-3	CTNNA3	-0.9	-0.8	-0.6	-0.7	-1.0	-1.1	-0.8	0.0	3	10
F1SRQ0	PIG Uncharacterized protein (Fragment)	NDUFB2	0.0	-0.5	-1.1	-1.4	0.1	-0.4	-0.9	0.1	1	1
F1S6Q7	PIG ATP synthase subunit delta, mitochondrial	ATP5D	-0.1	0.6	-1.6	-2.1	2.7	0.9	-0.4	-1.7	3	3
Q9HAU6	HUMAN Putative apoptosis inhibitor FKSG2	FKSG2	-1.9	-1.2	-0.1	0.2	0.5	1.9	-0.4	-0.6	1	1
I3LP66	PIG Uncharacterized protein	SLC19A1	0.1	-0.6	-1.4	-1.2	0.4	0.1	-0.7	-0.3	1	1
F1SJE6	PIG Uncharacterized protein (Fragment)	LOC100512151	-0.3	-1.1	-1.0	-0.7	-0.2	0.1	1.1	0.4	9	13
Q13813-2	HUMAN Isoform 2 of Spectrin alpha chain, non-erythrocytic 1	SPTAN1	-0.9	-1.0	-0.9	-0.3	-0.3	-0.6	-1.1	1.4	1	1
F1S485	PIG Uncharacterized protein	EPB41L1	-0.8	-0.9	-1.0	-0.4	-0.6	0.7	-0.5	-0.7	1	1
F1SJV0	PIG Uncharacterized protein	BUD13	-1.2	-1.5	-0.5	0.1	0.5	0.8	1.0	0.3	2	1
Q7YS30	PIG F1F0-ATP synthase complex g subunit-like protein (Fragment)	NA	-0.8	-0.4	-1.0	-0.9	-0.8	-0.1	0.4	0.5	2	2
F1SA52	PIG Uncharacterized protein	KANK3	-1.1	-1.0	-0.8	-0.1	-0.6	0.2	-0.4	1.8	2	2
M3TYS2	PIG Nucleoporin like 1 tv2	NUPL1	-0.1	0.2	-1.4	-1.8	-1.8	-1.3	-1.5	-1.0	2	1
Q92833	HUMAN Protein Jumonji	JARID2	-0.1	-0.9	-1.3	-0.9	2.5	-0.5	-1.7	-1.3	2	1
I3L9P1	PIG Uncharacterized protein	CCDC48	-0.8	-1.1	-1.0	-0.2	-0.2	0.0	-0.1	1.5	1	2
B4DI95	HUMAN cDNA FLJ52955, moderately similar to Homo sapiens solute carrier family 25 (SLC25A24)	NA	-0.9	-0.4	-0.9	-1.0	1.2	0.8	-0.5	-0.2	1	2
P55931	PIG Electron transfer flavoprotein-ubiquinone oxidoreductase, mitochondrial	ETFDH	-0.5	-0.4	-1.0	-1.2	-1.2	-1.0	-0.7	-0.1	19	20
I3LR51	PIG Uncharacterized protein	FKBP3	-0.8	-1.5	-0.7	-0.1	0.1	0.3	-0.1	0.2	3	3
Q96T83	HUMAN Sodium/hydrogen exchanger 7	SLC9A7	0.4	-1.8	-1.0	-0.7	-2.5	-2.7	-2.5	-1.1	1	1
K7GR70	PIG Uncharacterized protein	LOC100737114	-1.2	-1.3	-0.5	-0.1	-0.5	-0.4	-0.5	0.6	1	1
F1SFY0	PIG Uncharacterized protein	E2F8	-0.8	-0.3	-0.8	-1.2	-1.3	-1.0	-1.2	-0.7	2	1
O79875	PIG NADH-ubiquinone oxidoreductase chain 2	MT-ND2	0.0	0.0	-1.7	-1.5	-1.1	-0.2	-0.8	0.1	2	2
F1SHH0	PIG Uncharacterized protein	NPAS3	-1.7	-0.4	-0.7	-0.4	0.1	-0.8	-0.5	0.4	1	1
Q9P0D3	HUMAN HSPC103 (Fragment)	NA	-0.1	-0.3	-1.4	-1.3	-0.8	-0.5	-0.5	0.3	1	1
F1S3G5	PIG Peptidyl-prolyl cis-trans isomerase NIMA interacting 1	PIN1	-1.1	-0.9	-0.5	-0.7	-0.5	-0.3	0.1	0.2	2	2
Q8N5G0	HUMAN Small integral membrane protein 20	SMIM20	-0.4	-1.0	-1.0	-0.8	0.7	1.2	0.6	0.1	1	1
F1SNR5	PIG Uncharacterized protein (Fragment)	OTUD7A	-1.0	-0.7	-0.8	-0.6	-1.5	-1.0	-0.6	0.5	2	1
Q14BN4	HUMAN Sarcolemmal membrane-associated protein	SLMAP	-1.1	-0.4	-0.7	-0.9	-0.7	-0.2	0.4	1.3	20	22
I3LDG8	PIG Uncharacterized protein	NA	-2.1	-1.1	-0.5	0.7	0.9	0.1	-0.1	0.1	2	1
Q9UNH7	HUMAN Sorting nexin-6	SNX6	-1.3	-0.8	-0.7	-0.3	-0.3	0.3	-0.5	0.0	7	2
K7GSS3	PIG Uncharacterized protein (Fragment)	ANK3	-0.3	-1.1	-1.0	-0.8	0.3	0.2	-0.7	-0.1	13	22
F1SAR5	PIG Uncharacterized protein	GLRX5	-0.3	-0.8	-1.0	-1.1	0.7	0.8	-0.2	-1.7	5	2
F1S9K7	PIG Uncharacterized protein	SLC30A10	-0.4	-0.9	-1.0	-0.8	-0.8	-1.1	-0.9	0.0	1	1
I3LVD1	PIG Uncharacterized protein	LOC100626596	-0.3	-0.8	-1.0	-1.0	0.4	0.6	-0.2	0.6	8	9
F1S6W7	PIG Uncharacterized protein (Fragment)	USP47	-0.4	-0.9	-1.0	-0.9	-0.4	0.2	0.1	0.5	1	1

I3L8Q2	PIG Uncharacterized protein	PHF23	0.2	-0.8	-1.0	-1.6	-0.7	-0.8	-1.9	-0.3	1	1
F1S1E1	PIG Uncharacterized protein	DCAF13	-0.1	-0.8	-0.8	-1.4	-0.9	-1.0	0.0	-0.4	1	1
F1S297	PIG Isocitrate dehydrogenase [NAD] subunit gamma, mitochondrial	IDH3G	-0.1	-1.1	-1.1	-0.9	-0.1	-0.3	-0.5	-0.7	10	11
F1S009	PIG Uncharacterized protein	HEXDC	-0.6	-1.3	-0.8	-0.5	-0.5	0.0	0.6	1.4	1	1
P32418	HUMAN Sodium/calcium exchanger 1	SLC8A1	-0.7	-0.9	-0.9	-0.7	-0.6	-0.4	-0.1	0.3	12	11
Q4VK66	PIG Glycogen synthase 1 (Fragment)	NA	-0.4	-1.1	-0.8	-0.9	-1.7	-1.5	-1.1	-0.5	2	4
P19367	HUMAN Hexokinase-1	HK1	-0.6	-0.6	-0.9	-1.1	-0.9	-0.5	0.0	-0.2	1	1
H9TUB4	PIG UME3A	NA	-1.3	-0.8	-0.6	-0.5	0.7	0.6	-0.9	0.2	1	2
I3LD98	PIG Uncharacterized protein	ANKRD50	-0.5	-0.7	-0.9	-1.1	-0.3	-0.6	-2.0	-0.7	1	1
Q9NZ45	HUMAN CDGSH iron-sulfur domain-containing protein 1	CISD1	-0.7	-0.5	-0.9	-1.1	-1.6	-1.2	-0.5	0.9	1	1
I3LCZ7	PIG Uncharacterized protein (Fragment)	NA	-1.1	-1.1	-1.0	0.1	-0.5	-0.7	-1.1	0.2	1	1
Q94PW4	PIG ATP synthase protein 8	ATPase 8	-0.5	-0.1	-1.1	-1.6	-0.7	1.2	2.3	1.8	2	2
Q864B5	PIG PDJA1 chaperone	DNAJA4	-1.0	-1.1	-0.7	-0.3	-0.3	0.2	-0.3	-0.1	9	7
F1S6V0	PIG Uncharacterized protein	SWAP70	-1.4	-1.4	-0.6	0.1	-0.6	-0.5	-0.1	0.8	5	2
I3LS16	PIG NADH dehydrogenase [ubiquinone] 1 alpha subcomplex subunit 2	LOC100515604	-0.6	-0.1	-1.0	-1.6	-1.0	-0.9	-1.6	0.7	4	5
F1RWN4	PIG 14 kDa phosphohistidine phosphatase	PHPT1	-1.0	-1.0	-0.7	-0.6	1.0	1.7	1.9	1.1	2	2
Q92527	HUMAN Ankyrin repeat domain-containing protein 7	ANKRD7	-0.5	-0.6	-1.4	-0.7	-1.1	-0.2	-0.4	1.5	1	1
Q9UQB3	HUMAN Catenin delta-2	CTNND2	-0.6	-0.8	-1.2	-0.6	1.1	1.9	1.5	0.9	1	1
F1SSI2	PIG Uncharacterized protein	DHRS7	-1.1	-1.1	-0.7	-0.4	-1.5	-1.2	-1.3	-0.3	6	7
P05165	HUMAN Propionyl-CoA carboxylase alpha chain, mitochondrial	PCCA	-0.9	-0.6	-0.7	-1.1	-1.3	-1.5	-1.3	-0.5	8	10
O75335	HUMAN Liprin-alpha-4	PPFIA4	-0.7	-0.7	-0.7	-1.1	0.1	0.6	0.1	-1.5	1	2
F1SBQ5	PIG Solute carrier family 16, member 1 (Monocarboxylic acid transporter 1) tv1	SLC16A1	-1.2	-0.3	-0.9	-0.9	-1.8	-1.0	-1.3	1.3	9	7
Q28945	PIG Gi-alpha-1 protein (Fragment)	NA	0.3	-1.2	-1.2	-1.2	0.1	0.6	0.6	0.3	1	1
F1S8Q4	PIG Uncharacterized protein	ADCK3	0.0	-1.2	-1.2	-0.8	-0.4	-0.7	-1.0	-0.6	11	11
F1RF76	PIG Uncharacterized protein	MFN2	-0.9	-0.9	-0.9	-0.6	-0.7	-0.5	-0.6	-0.4	2	7
B4DR72	HUMAN cDNA FLJ59963, highly similar to Homo sapiens multiple substrate lipid kinase (MULK)	NA	-0.9	-0.7	-0.9	-0.8	-0.3	0.1	0.6	1.6	1	1
Q29101	PIG Gap junction alpha-1 protein	GJA1	-0.8	-0.8	-0.9	-0.8	0.1	0.4	0.9	1.1	2	3
F1S3H7	PIG Uncharacterized protein	LOC100156930	-0.4	-0.8	-1.1	-1.0	0.2	0.8	0.6	1.0	3	3
F1RR64	PIG Uncharacterized protein	ENDOG	0.0	-0.6	-1.2	-1.6	-0.1	-0.1	-0.4	-1.6	4	5
Q1EG61	PIG Mannose-P-dolichol utilization defect 1	LOC733696	-1.1	-0.4	-0.7	-1.1	-0.5	-1.4	-1.5	-0.3	1	1
F1RL81	PIG Uncharacterized protein	HSD17B14	-0.6	-1.4	-0.9	-0.4	-0.1	-0.5	0.3	-0.4	1	1
I3LQ54	PIG Uncharacterized protein	NA	-0.9	-1.2	-1.0	-0.2	-1.0	-0.3	0.4	-0.7	2	5
G9F6X6	PIG Mitochondrial heat shock 60 kDa protein 1	NA	-0.9	-0.3	-1.1	-1.1	-0.7	-0.6	-1.5	-1.4	1	1
I3L9U0	PIG Uncharacterized protein (Fragment)	MYL5	0.4	0.6	-1.6	-2.7	1.8	0.0	-1.6	-5.0	1	1
Q15785	HUMAN Mitochondrial import receptor subunit TOM34	TOMM34	-0.2	-1.2	-1.2	-0.7	-1.2	-0.5	-0.1	-0.1	1	1
F1SS66	PIG Uncharacterized protein	MYH13	-0.6	-0.5	-1.0	-1.2	-1.5	-0.1	-0.8	0.9	4	7
F1SLF0	PIG Uncharacterized protein	SPR	-0.8	-1.0	-0.9	-0.6	0.7	0.7	-0.2	-0.9	8	9
F1S765	PIG Uncharacterized protein	C1orf123	-1.0	-0.9	-0.8	-0.7	-0.9	-0.8	-0.6	-1.4	1	2
F1RK90	PIG Uncharacterized protein	PPL	-0.4	-1.5	-0.8	-0.6	6.2	-0.2	0.6	0.5	2	1
F1RK48	PIG Uncharacterized protein (Fragment)	SRL	-0.3	-0.2	-1.3	-1.6	-0.3	-0.6	-1.5	-0.3	23	21

Q9GJV4	PIG Four and a half LIM domains 1 protein, isoform C	fhl1C	-1.6	-2.1	-0.4	0.8	-2.0	0.5	1.0	0.6	3	4
F1RZU8	PIG Uncharacterized protein (Fragment)	DST	-0.8	0.3	-1.8	-1.1	0.1	0.2	1.5	-0.3	4	4
F1RR78	PIG Uncharacterized protein	LOC100049693	-0.9	-1.1	-0.8	-0.5	-0.7	-0.5	-0.5	1.0	149	149
B1PK12	PIG Branched chain keto acid dehydrogenase E1 alpha polypeptide	NA	-0.1	-0.6	-1.3	-1.3	-0.6	-1.6	-1.8	-2.0	6	5
F1SU36	PIG Uncharacterized protein (Fragment)	SNRNP200	-1.2	-1.3	-0.8	-0.1	-0.6	1.0	0.8	-0.2	1	1
F1SSH3	PIG Uncharacterized protein	NA	-0.2	-1.0	-1.0	-1.1	2.1	2.5	-0.3	-0.3	1	3
F1RFM8	PIG Uncharacterized protein (Fragment)	DNAH10	-0.5	-0.9	-1.0	-1.0	-0.2	-0.1	0.0	0.7	2	2
Q9HBL0	HUMAN Tensin-1	TNS1	-0.6	0.0	-1.2	-1.5	-0.8	-1.2	-1.1	-0.1	1	1
I3LU95	PIG Uncharacterized protein	PDS5A	-1.8	-0.9	-0.5	-0.1	-0.8	-1.0	-0.4	-0.5	1	1
Q6UY01	HUMAN Leucine-rich repeat-containing protein 31	LRRC31	-1.0	-0.9	-0.4	-1.0	-0.9	-1.3	-1.1	-0.5	1	1
F1SHD7	PIG Uncharacterized protein (Fragment)	NDUFS1	-0.4	-0.3	-1.2	-1.5	-0.2	-0.1	-0.8	-1.0	34	32
F1SIV3	PIG Uncharacterized protein	LOC100155298	-0.9	-1.1	-0.6	-0.7	2.8	1.2	-1.9	-4.1	4	2
F1RWX5	PIG Uncharacterized protein (Fragment)	WRNIP1	-1.5	-1.2	-0.2	-0.4	0.3	1.1	-0.4	-0.9	2	2
Q5VTT5	HUMAN Myomesin-3	MYOM3	-0.5	-1.0	-0.9	-1.0	-0.8	-0.8	-0.7	0.0	13	18
P31152	HUMAN Mitogen-activated protein kinase 4	MAPK4	-3.0	-1.5	-0.1	1.2	-1.5	-0.9	-1.2	0.6	1	1
F1RHN3	PIG Catechol O-methyltransferase	COMT	-0.4	-1.5	-0.9	-0.6	0.6	-0.2	0.2	0.4	3	2
Q6Q2C2	PIG Bifunctional epoxide hydrolase 2	EPHX2	-0.8	-1.1	-0.7	-0.8	-0.1	-0.6	-0.2	-0.5	4	4
B6CVL5	PIG High mobility group AT-hook 1 transcript variant 2	HMGA1	-1.3	-0.2	-1.2	-0.7	0.7	1.3	2.3	4.8	2	1
I3LS02	PIG Uncharacterized protein	NA	-1.6	-1.2	-0.7	0.0	-0.5	-0.7	0.2	0.5	2	5
A6NFB4	HUMAN Chorionic somatomammotropin hormone	CSH1	-0.4	-0.2	-1.2	-1.6	-0.8	0.2	-1.0	-1.7	1	1
P16960	PIG Ryanodine receptor 1	RYR1	-0.8	-1.0	-0.7	-0.8	-1.5	-1.4	-1.0	-0.4	2	3
K9IWG9	PIG Tensin-1	TNS1	-0.5	-1.1	-1.1	-0.7	-0.4	0.3	-0.1	0.4	9	8
F1SD78	PIG Uncharacterized protein	MRI1	-1.5	-1.3	-0.6	0.0	-0.6	1.6	0.6	1.1	1	2
A5GZW8	PIG Succinate dehydrogenase [ubiquinone] cytochrome b small subunit, mitochondrial	SDHD	-0.7	-0.8	-0.9	-1.0	-1.5	-1.0	-0.6	0.2	3	2
F1S8G3	PIG Uncharacterized protein	MRPS33	-0.9	-0.7	-1.1	-0.7	-1.2	-1.3	-1.4	-0.3	1	1
F2Z565	PIG Uncharacterized protein	LOC100158185	-1.3	-0.4	-0.7	-1.0	-2.4	-0.9	-0.6	0.1	7	5
F1S2X3	PIG Uncharacterized protein (Fragment)	LOC100512509	-0.5	-0.3	-1.1	-1.5	-0.7	0.0	-2.1	-1.7	5	2
F1RSZ3	PIG Uncharacterized protein	ACSL1	-0.7	-0.8	-0.9	-1.0	0.1	0.5	-0.1	0.8	2	2
F1RK61	PIG Uncharacterized protein	UFD1L	-0.1	-0.6	-1.2	-1.5	-0.2	0.1	-0.5	1.0	1	1
A6NC97	HUMAN Putative protein FAM172B	FAM172BP	-0.9	-1.9	-0.5	-0.2	-0.2	0.1	0.3	0.2	1	1
F1SD90	PIG Uncharacterized protein	SMEK1	-1.1	-1.0	-2.6	1.2	1.2	-2.3	4.8	2.1	1	1
Q8IYY4	HUMAN Zinc finger protein DZIP1L	DZIP1L	-1.0	-1.3	-1.1	-0.1	3.9	0.1	0.8	0.9	2	1
I3LNT5	PIG Uncharacterized protein	NA	-0.8	-0.1	-1.4	-1.2	-0.4	-1.3	-1.4	0.4	1	1
Q01814	HUMAN Plasma membrane calcium-transporting ATPase 2	ATP2B2	-1.8	-1.7	-0.3	0.3	0.8	2.6	1.3	-0.1	1	1
I3LSM7	PIG Uncharacterized protein (Fragment)	IMMT	-0.9	-0.6	-0.9	-1.1	-1.5	-1.3	-1.0	0.5	41	46
O15230	HUMAN Laminin subunit alpha-5	LAMA5	-1.8	-1.4	-0.3	0.0	-1.3	-1.1	0.3	-0.2	3	5
H0YAJ8	HUMAN Rap guanine nucleotide exchange factor 2 (Fragment)	RAPGEF2	-0.5	-1.2	-0.8	-0.9	-1.5	-1.5	-1.0	-1.1	1	1
I3LE12	PIG Uncharacterized protein	HSD17B12	-1.1	-1.3	-0.8	-0.2	-0.8	-1.0	-1.5	0.2	2	3
P81140	PIG Glutaryl-CoA dehydrogenase, mitochondrial (Fragment)	GCDH	-0.3	-0.1	-1.4	-1.5	-0.8	-1.2	-2.4	-1.9	6	5
I3LJ88	PIG Uncharacterized protein	TMOD1	-0.6	-0.7	-1.0	-1.2	0.1	0.2	-1.1	-1.4	19	18

F1SAM3	PIG Uncharacterized protein (Fragment)	CDH2	-0.6	-0.6	-1.1	-1.2	0.5	0.9	-0.3	-0.7	15	14
F1SLF4	PIG Uncharacterized protein	SMYD5	-0.8	-0.9	-1.0	-0.8	1.7	1.9	-1.0	0.2	2	1
A0AV96-2	HUMAN Isoform 2 of RNA-binding protein 47	RBM47	-0.1	-0.8	-1.1	-1.4	-0.6	-0.3	-0.2	0.8	1	1
F1RRH0	PIG Uncharacterized protein	EOMES	-1.0	-1.0	-0.8	-0.6	0.2	0.3	1.7	0.7	1	1
Q9BRK3	HUMAN Matrix-remodeling-associated protein 8	MXRA8	-0.3	-0.2	-1.0	-2.0	-1.5	0.3	0.0	1.6	1	1
F1S006	PIG Uncharacterized protein	FN3K	-0.8	-0.8	-1.1	-0.8	-1.2	-0.6	-0.5	-0.3	1	1
F1RLE1	PIG Uncharacterized protein	COMMD10	0.0	-0.7	-1.4	-1.4	-1.4	-1.5	-1.1	-1.2	1	1
I3LNJ1	PIG Uncharacterized protein (Fragment)	NA	-1.3	-1.0	-0.8	-0.4	0.6	0.0	0.0	-0.6	1	2
F1SHU0	PIG Uncharacterized protein (Fragment)	LOC100516841	-0.2	-0.7	-1.3	-1.2	0.0	0.3	0.1	-1.2	3	4
B4DVU9	HUMAN cDNA FLJ54389, highly similar to Heat shock 70 kDa protein 1	NA	-0.5	-0.6	-1.2	-1.2	-0.1	-0.7	-1.0	0.0	1	1
F1SUR2	PIG Uncharacterized protein	P4HA3	-0.1	-1.2	-1.2	-1.0	-0.2	-0.2	0.1	-0.4	1	1
I3LBV8	PIG Uncharacterized protein (Fragment)	PPFIA3	-0.8	-1.8	-0.8	-0.1	-0.2	-0.6	-0.3	-0.9	2	2
O60432	HUMAN F02569	NA	-0.9	-1.4	-0.5	-0.7	-0.2	0.5	2.7	1.9	1	1
Q09136	PIG 5'-AMP-activated protein kinase catalytic subunit alpha-1 (Fragments)	PRKAA1	-1.3	-1.3	-0.9	-0.1	-1.3	-1.8	-0.3	-0.5	1	2
F1RZP9	PIG Uncharacterized protein (Fragment)	MICAL1	-0.8	-0.9	-0.9	-0.9	0.8	-0.3	0.6	0.2	2	1
F1RGV4	PIG Uncharacterized protein	DECR2	-0.3	-1.2	-1.0	-1.0	-0.7	-1.2	-1.3	0.0	1	1
Q9GJT2	PIG S-formylglutathione hydrolase	ESD	-1.6	-1.4	-0.6	0.1	-1.0	-1.0	-0.3	0.2	6	5
Q14849	HUMAN StAR-related lipid transfer protein 3	STARD3	-0.2	0.2	-1.6	-1.9	-1.0	0.0	-1.1	0.2	1	1
P26234	PIG Vinculin	VCL	-0.9	-1.3	-1.0	-0.4	-0.6	-0.2	0.2	0.7	71	74
I3LDL4	PIG Uncharacterized protein	UNC80	-0.1	-0.7	-1.3	-1.4	1.6	0.6	-0.7	-2.5	1	2
P41367	PIG Medium-chain specific acyl-CoA dehydrogenase, mitochondrial	ACADM	0.0	-0.6	-1.6	-1.3	-0.4	0.9	0.5	-0.3	15	16
Q864Y5	PIG Small heat shock protein B3 (Fragment)	HSPB3	-1.3	-1.0	-0.7	-0.5	0.2	0.2	0.0	-0.7	4	3
F1RR97	PIG Ubiquinone biosynthesis protein COQ4 homolog, mitochondrial	LOC100156776	-0.8	-1.2	-1.0	-0.5	0.1	-0.2	-1.1	-1.0	1	2
Q9UKX3	HUMAN Myosin-13	MYH13	-0.8	-0.7	-0.9	-1.1	-2.1	-2.0	-1.5	-0.2	5	5
F1SBU8	PIG Uncharacterized protein	C19orf70	-0.5	-0.7	-1.1	-1.4	0.1	-0.1	-0.4	-0.3	1	1
I3L660	PIG Profilin (Fragment)	PFN2	-0.7	-1.6	-1.0	-0.3	-0.4	0.1	-0.7	-0.5	4	3
Q07414	HUMAN Tropomyosin (Fragment)	NA	-0.5	-0.8	-1.1	-1.1	0.1	-0.4	0.3	1.1	1	1
F1SU55	PIG Uncharacterized protein	SYNPO2L	-1.0	-1.0	-0.8	-0.8	0.2	0.4	0.5	0.0	16	19
F1RJ58	PIG Uncharacterized protein (Fragment)	NF1	-0.5	-0.7	-1.3	-1.0	-1.5	-0.2	0.2	0.6	1	1
Q92556	HUMAN Engulfment and cell motility protein 1	ELMO1	-1.0	-1.4	-1.1	-0.1	-1.2	-1.1	-0.9	-0.5	1	1
K7GKB6	PIG Uncharacterized protein	BST1	-0.1	-0.7	-1.5	-1.3	-0.1	-0.4	0.7	0.3	1	1
F1SKM5	PIG Uncharacterized protein	CCDC51	-0.5	-0.8	-1.1	-1.2	0.3	0.3	0.0	1.3	1	1
Q0QF01	PIG Succinate dehydrogenase [ubiquinone] flavoprotein subunit, mitochondrial	SDHA	-0.6	-0.5	-1.2	-1.3	-1.5	-1.4	-0.9	-0.5	22	24
Q1T7A8	PIG Type VI collagen alpha-1 chain (Fragment)	COL6A1	-0.9	-1.6	-0.9	-0.1	-0.7	-1.4	-1.3	0.7	2	3
I3LNZ2	PIG Uncharacterized protein	LOC100510904	-0.8	-0.6	-1.3	-0.9	-0.6	0.6	1.6	1.8	4	5
F1SPP5	PIG Uncharacterized protein	SEMA3F	-0.9	-1.7	-0.8	-0.3	-0.5	0.1	0.1	1.7	2	2
P12111	HUMAN Collagen alpha-3(VI) chain	COL6A3	-0.8	-1.4	-1.2	-0.2	-0.5	-0.7	-1.3	0.7	1	1
F1SGT3	PIG Uncharacterized protein	PDHX	-0.5	-0.1	-1.4	-1.6	-0.2	0.8	0.2	-0.9	18	14
I3LFH3	PIG Rho-associated protein kinase	ROCK1	-0.6	-0.7	-1.3	-1.0	49.1	52.4	52.3	49.8	1	1
I3LRD5	PIG Uncharacterized protein	EIF4H	-0.9	-1.5	-0.9	-0.3	0.3	-0.6	-1.1	-0.7	5	6

P49419	HUMAN Alpha-aminoadipic semialdehyde dehydrogenase	ALDH7A1	-0.6	-0.7	-1.3	-1.0	-1.4	-2.8	-2.4	-1.1	1	1
K9J6I8	PIG Dedicator of cytokinesis 2	DOCK2	-0.8	-1.1	-1.1	-0.6	-0.4	-2.0	-1.5	-0.1	1	1
F1RF45	PIG Uncharacterized protein (Fragment)	HOMER1	-0.8	-1.0	-1.0	-0.9	0.0	0.5	1.0	-0.9	2	1
Q9P2D6	HUMAN Protein FAM135A	FAM135A	-1.0	-1.5	-0.8	-0.4	0.3	0.5	2.0	1.5	1	1
B7U2H0	PIG 1-acylglycerol-3-phosphate O-acyltransferase 8	LCLAT1	-0.7	-0.8	-1.1	-1.1	0.3	-0.5	-0.8	0.1	1	4
I3LN50	PIG Uncharacterized protein	EIF2B3	-1.1	-1.5	-1.0	0.0	-0.5	-0.4	-0.9	0.0	1	1
Q96DR7	HUMAN Rho guanine nucleotide exchange factor 26	ARHGEF26	-0.9	0.1	-1.0	-1.9	-0.2	0.2	-0.5	0.7	1	1
Q27HS0	PIG Signaling lymphocytic activation molecule (Fragment)	NA	-1.4	-1.6	-0.5	-0.2	-2.0	-1.5	-1.3	0.1	1	1
Q53LP3	HUMAN Ankyrin repeat domain-containing protein SOWAHC	SOWAHC	-0.5	-0.7	-1.0	-1.5	0.5	0.2	0.1	-1.6	1	1
P48735	HUMAN Isocitrate dehydrogenase [NADP], mitochondrial	IDH2	0.0	-0.9	-1.5	-1.3	0.5	0.2	-0.4	-0.9	2	2
Q13206	HUMAN Probable ATP-dependent RNA helicase DDX10	DDX10	-0.2	-0.7	-1.2	-1.5	0.4	0.2	-0.3	-0.1	1	1
F1SIJ9	PIG Uncharacterized protein (Fragment)	PSAT1	-1.4	-1.8	-0.5	0.0	-1.1	-1.0	-0.4	0.3	1	1
F1SJ30	PIG Mannose-6-phosphate isomerase	MPI	-0.7	-2.1	-0.8	-0.1	1.6	0.5	0.4	-2.1	2	1
I3L953	PIG Uncharacterized protein (Fragment)	OBSCN	-1.7	-1.2	-0.4	-0.4	-0.1	-1.0	0.1	0.8	10	20
F1RIX8	PIG Uncharacterized protein	CBR4	-0.3	-1.1	-1.1	-1.1	0.2	-1.0	1.1	-0.7	6	6
Q8N163	HUMAN Cell cycle and apoptosis regulator protein 2	CCAR2	0.2	-0.7	-1.4	-1.8	-0.7	-1.1	-1.5	-0.4	1	1
F1SK65	PIG Uncharacterized protein (Fragment)	NA	-1.8	-1.9	0.1	0.0	-1.1	-0.4	2.9	0.4	1	2
Q56P28	PIG PRA1 family protein 3	ARL6IP5	-0.9	-0.9	-1.0	-0.9	-0.5	-0.1	-0.8	-0.4	4	6
F1SNF6	PIG Uncharacterized protein	CHCHD3	-0.9	-0.5	-1.1	-1.2	-1.1	-0.6	0.3	0.7	13	15
P14854	HUMAN Cytochrome c oxidase subunit 6B1	COX6B1	-0.7	-0.1	-1.2	-1.7	-0.6	-0.3	-1.2	-0.5	1	1
F1SRF0	PIG Uncharacterized protein	HHATL	-0.5	-0.9	-1.1	-1.1	-0.2	-0.7	-0.3	0.3	8	10
G9BWQ1	PIG V-akt murine thymoma viral oncogene-like 1	AKT1	-0.4	-0.7	-1.2	-1.3	0.1	-1.0	-1.4	-0.3	1	2
F1RS89	PIG Uncharacterized protein	NA	-1.6	-1.9	-0.5	0.3	0.2	0.4	-0.4	-1.2	1	1
P00819	PIG Acylphosphatase-2	ACYP2	-0.9	-1.4	-0.8	-0.6	-0.7	-0.7	-1.4	0.1	3	6
Q8HY46	PIG Carnitine O-palmitoyltransferase 1, muscle isoform	CPT1B	0.0	-1.0	-1.2	-1.5	-0.5	-0.4	-1.1	-0.4	18	19
F1STB2	PIG Uncharacterized protein	HINT2	-0.3	-0.8	-1.5	-1.2	-0.7	-1.0	-0.9	-1.6	6	5
Q15772	HUMAN Striated muscle preferentially expressed protein kinase	SPEG	-1.1	-0.9	-0.8	-0.9	-0.1	-0.8	-1.2	0.5	2	5
F1S8Y5	PIG Uncharacterized protein	PGAM1	-1.0	-1.6	-0.7	-0.3	0.2	0.0	2.1	-0.6	8	8
Q06AU7	PIG Ras-related protein Rab-1B	RAB1B	-1.7	-1.5	-0.7	0.2	-1.1	-0.9	-1.1	0.8	2	2
Q9NVD7	HUMAN Alpha-parvin	PARVA	-1.4	-1.7	-0.8	0.1	-0.9	-0.4	-1.2	-1.1	2	1
F1S3J2	PIG Uncharacterized protein	PNMA1	-0.6	-0.8	-1.2	-1.1	-0.7	-0.2	-0.5	-1.0	1	1
Q7Z442	HUMAN Polycystic kidney disease protein 1-like 2	PKD1L2	-0.3	-0.6	-1.4	-1.5	-0.7	-0.9	-1.2	-0.4	2	1
Q9HBJ7	HUMAN Ubiquitin carboxyl-terminal hydrolase 29	USP29	-0.2	-0.6	-1.1	-1.8	-0.6	-0.4	-0.7	-0.2	1	1
Q9GL80	PIG Actin-related protein 3 (Fragment)	Arp3	-1.0	-1.6	-0.9	-0.2	0.4	0.2	-0.2	0.7	1	1
E7FLX9	PIG A kinase anchor protein 1	AKAP1	-1.0	-1.4	-1.0	-0.3	0.0	-0.2	0.1	0.9	1	3
Q59E87	HUMAN RalA binding protein 1 variant (Fragment)	NA	-0.4	-0.7	-1.1	-1.5	1.9	0.0	-0.8	-3.3	1	1
E9PG25	HUMAN Coiled-coil domain-containing protein 108	CCDC108	-0.3	-0.8	-1.2	-1.4	-1.4	-1.4	-1.0	-1.5	1	1
F1RIA5	PIG Uncharacterized protein	VPS37C	-1.0	-1.6	-0.8	-0.4	0.4	-0.1	0.9	0.7	1	1
F1SRU4	PIG Uncharacterized protein (Fragment)	SNAPC1	-1.4	-1.2	0.0	-1.0	-3.1	-1.7	-1.0	-1.1	1	1
Q86U44	HUMAN N6-adenosine-methyltransferase 70 kDa subunit	METTL3	-1.0	-1.2	-0.5	-1.1	-0.3	-0.4	0.3	0.8	1	1

F1RT90	PIG Uncharacterized protein (Fragment)	NA	-1.2	-0.8	-1.1	-0.7	-0.2	-0.9	-1.6	-2.2	2	1
F1SRR8	PIG Uncharacterized protein (Fragment)	NA	-1.1	-1.0	-0.4	-1.3	-0.3	-0.5	0.8	0.2	1	1
Q86W77	HUMAN ANKS3 protein	ANKS3	-1.0	-1.0	-1.1	-0.7	0.0	-0.2	-0.4	0.8	1	1
P67872	PIG Casein kinase II subunit beta	CSNK2B	-1.8	-1.0	-0.6	-0.4	-0.6	-0.2	0.3	-0.6	2	2
P36542	HUMAN ATP synthase subunit gamma, mitochondrial	ATP5C1	-0.3	-0.4	-1.4	-1.6	-0.3	-0.5	1.4	-0.3	2	2
F1RUW1	PIG Uncharacterized protein (Fragment)	MED20	-0.6	-1.2	-1.0	-1.0	0.3	2.4	2.9	1.1	1	1
O60313	HUMAN Dynamin-like 120 kDa protein, mitochondrial	OPA1	0.5	-1.2	-1.7	-1.3	0.5	0.2	-0.9	-1.2	2	2
Q99807	HUMAN Ubiquinone biosynthesis protein COQ7 homolog	COQ7	-0.8	-0.8	-0.8	-1.3	-0.6	-0.1	-0.2	-0.5	2	2
F1RVM1	PIG Uncharacterized protein	LOC100518202	-0.3	-1.0	-1.5	-1.0	-0.1	0.2	-0.6	-1.4	6	7
K7GMJ5	PIG Uncharacterized protein	ADHFE1	0.0	-0.9	-1.2	-1.7	0.1	-0.9	0.6	-0.7	5	7
F1RWV4	PIG Uncharacterized protein	NDUFB11	-0.6	-0.4	-1.0	-1.8	-1.1	-1.0	-1.3	0.6	5	6
F1RPD4	PIG Uncharacterized protein	LOC100620271	-1.0	0.0	-1.1	-1.6	-1.3	-1.3	-0.7	-1.2	10	8
Q29259	PIG NADH dehydrogenase [ubiquinone] 1 beta subcomplex subunit 6	NDUFB6	-0.8	-0.4	-1.0	-1.6	-1.6	-1.0	-0.6	0.4	4	3
I3LJK0	PIG Uncharacterized protein	NA	-0.6	-1.3	-1.1	-0.8	-1.2	-1.4	-1.3	-0.2	1	1
Q2TSD0	HUMAN Glyceraldehyde-3-phosphate dehydrogenase	NA	-0.6	-1.2	-0.9	-1.0	-0.7	0.3	0.8	0.4	4	1
Q9BQS7	HUMAN Hephaestin	HEPH	-0.6	1.0	-1.9	-2.3	-1.8	-1.5	-1.3	-0.8	1	1
I3L5S5	PIG Uncharacterized protein (Fragment)	ZNF784	-0.6	-1.1	-1.1	-1.1	-2.0	-1.5	-1.6	0.4	1	1
I3LH99	PIG Uncharacterized protein (Fragment)	SLC7A13	-1.5	-1.6	-0.6	-0.1	-0.5	-1.0	0.0	0.0	1	1
I3LKD2	PIG Uncharacterized protein	FAM162A	-0.7	-0.6	-1.2	-1.4	-0.2	-0.3	-0.6	1.1	9	11
I3LRK1	PIG Uncharacterized protein	NA	-0.7	-0.9	-1.1	-1.0	0.0	-0.2	-0.1	-0.2	8	8
D6QST6	PIG 2,4-dienoyl-CoA reductase 1	DECR1	-0.4	-0.7	-1.2	-1.5	-1.4	-1.3	-4.9	-0.9	1	1
P79274	PIG Long-chain specific acyl-CoA dehydrogenase, mitochondrial	ACADL	-0.4	-0.6	-1.4	-1.4	-0.8	-1.1	1.1	-0.2	22	23
K7GQ72	PIG Uncharacterized protein	SYTL5	-0.4	-0.8	-1.2	-1.4	0.1	-0.2	0.9	0.2	1	1
F1SH10	PIG Uncharacterized protein (Fragment)	NCEH1	-0.9	-0.8	-1.0	-1.1	-1.7	-1.9	-1.6	0.0	12	12
F1S1D9	PIG Uncharacterized protein	PSMC3IP	-0.4	-1.6	-1.1	-0.7	-0.4	0.2	0.1	0.8	2	2
Q29117	PIG Titin (Fragment)	NA	-0.5	-1.4	-1.1	-0.9	0.3	0.9	2.3	2.5	16	21
F1RJH2	PIG Short-chain-specific acyl-CoA dehydrogenase, mitochondrial	ACADS	0.1	-1.5	-1.3	-1.2	-0.1	-0.5	-1.6	-0.3	2	1
Q9TV69	PIG Trans-1,2-dihydrobenzene-1,2-diol dehydrogenase	DHDH	-1.1	-1.3	-0.7	-0.8	0.3	-0.1	1.6	0.2	4	8
F1SBH1	PIG D-tyrosyl-tRNA(Tyr) deacylase	DTD1	-1.0	-1.7	-1.0	-0.1	-0.2	0.6	-0.3	0.7	3	3
Q2QLE2	PIG Caveolin-2	CAV2	-1.4	-1.0	-0.9	-0.5	0.1	0.1	0.1	0.5	2	4
Q95334	PIG Glutamyl aminopeptidase	ENPEP	-1.6	-1.3	-0.6	-0.4	-0.4	-0.5	-0.3	-0.5	1	2
F1SHS6	PIG Serine/threonine-protein kinase WNK1	WNK1	-1.0	-1.2	-0.8	-0.9	0.5	0.4	1.6	3.9	1	1
F1S078	PIG Uncharacterized protein	TLN2	-0.4	-1.3	-1.2	-1.0	-0.7	-0.2	-0.4	-0.1	5	8
R4GMP8	HUMAN Serine/arginine-rich-splicing factor 10	SRSF10	-0.7	-0.7	-1.2	-1.3	-0.6	0.2	-0.2	-0.9	1	1
Q9TSX9	PIG Peroxiredoxin-6	PRDX6	-1.4	-1.1	-0.7	-0.6	-0.4	-0.2	-0.1	0.8	15	11
P29376-2	HUMAN Isoform Lambda P1 of Leukocyte tyrosine kinase receptor	LTK	-2.9	-1.2	0.1	0.2	-0.6	-0.9	-2.4	-2.4	1	1
F1S6M7	PIG Uncharacterized protein	LOC100620928	-1.4	-1.7	-0.4	-0.3	0.2	0.7	-0.5	-0.5	2	2
I6ZX61	PIG Glycogen synthase kinase 3 beta	GSK3B	-0.3	-1.2	-1.3	-1.1	0.3	-0.7	-1.2	-0.5	2	3
F1RM62	PIG Uncharacterized protein	HSPB6	-1.3	-1.9	-0.8	0.1	0.2	-1.4	-0.5	-0.8	8	6
Q86TX2	HUMAN Acyl-coenzyme A thioesterase 1	ACOT1	-0.4	-0.4	-1.4	-1.7	-1.4	-1.5	-0.4	0.9	3	3

Q9Y4W6	HUMAN AFG3-like protein 2	AFG3L2	-0.5	-1.2	-1.3	-0.9	1.3	2.6	-0.8	-0.7	1	1
F1RLG5	PIG Uncharacterized protein	REEP5	-1.5	-1.1	-0.7	-0.6	-1.7	-1.6	-0.9	0.7	9	7
B2ZF49	PIG Trifunctional enzyme subunit alpha, mitochondrial	HADHA	0.2	-0.9	-1.3	-1.9	-1.1	-4.0	-2.0	-4.4	1	1
O95425	HUMAN Supervillin	SVIL	-1.5	-1.2	-0.6	-0.6	0.2	0.0	0.0	2.0	1	1
Q5PYH3	PIG GTP-binding protein SAR1b	SAR1B	-0.9	-1.4	-0.8	-0.8	2.0	0.8	-0.3	0.2	4	2
I3LB92	PIG Uncharacterized protein (Fragment)	MYOM3	-0.5	-1.1	-1.1	-1.3	-1.3	-1.5	-1.0	0.6	12	14
Q29551	PIG Succinyl-CoA:3-ketoacid coenzyme A transferase 1, mitochondrial	OXCT1	-0.4	-0.5	-1.6	-1.5	0.0	0.3	-0.7	-1.4	26	27
F1APU6	PIG Toll-like receptor 3 short-type	TLR3	-0.4	-0.4	-1.6	-1.5	-0.5	-0.3	-0.4	-0.1	1	1
P83111	HUMAN Serine beta-lactamase-like protein LACTB, mitochondrial	LACTB	-0.5	-2.0	-0.8	-0.6	-0.5	-0.6	0.9	2.0	1	1
Q8MI68	PIG Methylmalonyl-CoA mutase, mitochondrial	MUT	-0.2	-1.4	-1.4	-1.0	1.3	0.2	-0.1	-0.6	5	7
F1STL9	PIG Uncharacterized protein (Fragment)	KIAA1731	-0.6	-1.2	-1.2	-0.9	0.9	2.4	-0.4	-0.1	1	1
Q9TV36	PIG Fibrillin-1	FBN1	-1.8	-0.9	-1.1	-0.2	-2.4	-2.6	-1.0	1.6	6	8
Q0VF96	HUMAN Cingulin-like protein 1	CGNL1	-0.9	-0.1	-1.2	-1.7	-0.3	0.5	-0.6	0.0	2	1
I3L6F4	PIG Uncharacterized protein	TRIM55	-0.8	-1.5	-1.0	-0.7	0.6	1.0	-0.5	-1.5	6	3
F1SNW4	PIG MYL3	MYL3	-0.2	0.6	-1.8	-2.5	3.9	1.3	-1.1	-5.7	18	13
F1RGW9	PIG Uncharacterized protein (Fragment)	ARID4B	-0.7	-0.7	-1.4	-1.2	-0.6	0.3	0.7	0.6	1	1
P22695	HUMAN Cytochrome b-c1 complex subunit 2, mitochondrial	UQCRC2	-0.8	-0.1	-1.1	-1.9	-1.0	-0.7	-1.3	-1.9	2	2
I3LEN4	PIG Olfactory receptor (Fragment)	LOC100524696	-0.3	-0.7	-1.3	-1.7	-0.5	-0.5	0.2	1.1	1	1
Q9MYT8	PIG ATP synthase subunit e, mitochondrial	ATP5I	-0.7	-0.5	-1.2	-1.6	-0.6	-0.9	-0.8	2.3	6	4
P48539	HUMAN Purkinje cell protein 4	PCP4	-1.2	-0.9	-0.8	-1.2	1.4	0.1	-0.5	-0.7	1	1
D0VWV4	PIG Succinate dehydrogenase cytochrome b560 subunit, mitochondrial	SDHC	0.0	-0.5	-1.5	-2.0	-2.4	-1.6	-1.5	0.3	1	1
Q86UD0	HUMAN Suppressor APC domain-containing protein 2	SAPCD2	-1.0	-0.7	-1.1	-1.2	-0.6	-0.5	0.8	-0.3	1	1
F1SKD6	PIG Uncharacterized protein	SLC16A7	-1.9	-1.6	-0.9	0.4	-0.8	-0.7	-0.7	1.4	1	2
F1SPE5	PIG Uncharacterized protein	LOC100518837	-1.5	-1.4	-0.8	-0.3	-0.2	0.1	0.2	0.1	1	1
F1RXD4	PIG Uncharacterized protein	RMDN1	-0.5	-0.8	-1.5	-1.2	-0.2	-0.3	-0.5	-0.3	5	5
P62072	HUMAN Mitochondrial import inner membrane translocase subunit Tim10	TIMM10	-0.8	-0.6	-1.5	-1.1	-0.2	-1.1	0.1	0.1	1	1
Q14511	HUMAN Enhancer of filamentation 1	NEDD9	-1.2	-1.4	-0.9	-0.5	-0.5	-0.2	0.3	1.0	1	1
Q6ZT12	HUMAN E3 ubiquitin-protein ligase UBR3	UBR3	-0.5	-0.8	-1.2	-1.6	-0.1	0.8	0.7	0.4	1	1
B7TJ04	PIG Mitochondrial diablo	DIABLO	-1.1	-0.9	-1.3	-0.7	-0.9	-0.6	-1.3	0.0	7	6
I3LPW0	PIG Uncharacterized protein	NDUFB4	-0.2	-0.4	-1.6	-1.8	-1.2	-0.9	-1.8	-1.4	6	7
F1SML8	PIG 40S ribosomal protein S6	NA	-1.8	-2.8	-0.7	1.3	1.5	0.7	2.6	-0.7	2	1
Q9GL76	PIG Poliovirus receptor-related protein 1	PVRL1	-0.6	-0.7	-1.4	-1.3	0.4	0.7	-1.0	-1.0	1	1
I3LRP0	PIG Uncharacterized protein (Fragment)	NA	-1.5	-1.6	-0.6	-0.3	-0.1	-0.8	-0.3	0.8	9	12
D2D0E6	PIG Malonyl-CoA decarboxylase	MLYCD	-1.0	-0.7	-1.0	-1.2	-0.9	-0.1	-0.4	-0.3	2	6
Q9UHB7	HUMAN AF4/FMR2 family member 4	AFF4	-0.8	-1.0	-1.2	-1.1	-1.0	-1.1	-1.4	-1.0	1	2
B5LY67	HUMAN Uridine monophosphate synthetase isoform E	UMPS	-1.1	-1.2	-1.1	-0.7	-1.8	-1.5	-1.1	-0.1	1	1
K9IVI1	PIG 2-oxoglutarate dehydrogenase, mitochondrial	OGDH	-0.8	-0.7	-1.3	-1.3	-1.4	-1.5	-1.0	-0.5	40	46
F1RTQ5	PIG Histone H2B	LOC100152878	-3.1	-0.9	-0.6	0.5	-2.9	-1.0	0.9	1.5	2	1
F1SFG7	PIG Uncharacterized protein	OPA1	-0.8	-1.2	-1.1	-0.9	0.3	-0.2	-0.9	0.1	19	16
F1RXT5	PIG Uncharacterized protein	SBF1	-0.9	-1.5	-0.9	-0.7	-0.2	-1.3	-0.7	-1.6	1	1

I3LSI3	PIG Uncharacterized protein	AK2	-0.6	-0.8	-1.2	-1.4	0.0	1.2	0.1	-1.2	9	7
P82125	PIG 1,5-anhydro-D-fructose reductase	AKR1E2	-1.4	-1.7	-0.7	-0.3	-0.9	-0.2	-0.9	-0.5	1	1
I3LT95	PIG Uncharacterized protein	PSMG1	-2.1	-1.6	-0.8	0.5	-0.6	1.4	0.1	0.3	1	1
O15061	HUMAN Synemin	SYNM	-0.4	-1.4	-1.1	-1.2	0.0	-0.3	-0.5	-1.0	7	5
F1SHX8	PIG Uncharacterized protein (Fragment)	ATP5S	-0.6	-0.9	-1.4	-1.3	0.1	0.2	0.4	0.3	4	4
Q8SPJ9	PIG Cytochrome c oxidase subunit 7A1, mitochondrial	COX7A1	-0.3	-0.4	-1.4	-1.9	-1.6	-1.6	-1.4	0.2	2	2
O95714	HUMAN E3 ubiquitin-protein ligase HERC2	HERC2	-0.4	-1.0	-1.5	-1.2	-1.9	-1.0	-0.8	-1.1	1	1
P54819	HUMAN Adenylate kinase 2, mitochondrial	AK2	-0.9	-0.7	-1.2	-1.3	-0.1	0.0	0.8	0.2	2	1
Q6NZI2	HUMAN Polymerase I and transcript release factor	PTRF	-1.2	-0.6	-1.1	-1.2	-0.6	0.3	-0.3	-0.3	15	16
A8TX70	HUMAN Collagen alpha-5(VI) chain	COL6A5	-0.5	-0.7	-1.1	-1.8	-0.4	1.5	0.1	0.5	1	1
I3LHS7	PIG Uncharacterized protein (Fragment)	COQ9	-0.7	-0.4	-1.6	-1.5	-0.1	0.3	-0.6	-1.3	9	12
I3LJF7	PIG Uncharacterized protein (Fragment)	NA	-0.8	-0.7	-1.5	-1.0	-0.8	-0.1	-1.1	-0.6	1	1
F1SBB4	PIG Uncharacterized protein (Fragment)	ANKRD29	-1.8	-1.1	-0.8	-0.4	-2.4	-1.9	-1.6	0.7	1	1
P61586	HUMAN Transforming protein RhoA	RHOA	-3.0	-1.8	-0.3	1.0	-0.9	-0.2	0.0	1.5	2	3
I3LLQ8	PIG Uncharacterized protein (Fragment)	AFG3L2	-0.5	-1.5	-1.1	-1.0	-0.7	-0.5	-1.0	0.3	6	14
I3LJJ4	PIG Uncharacterized protein	ECH1	-0.8	-0.4	-1.3	-1.6	-0.6	-0.9	-1.1	-0.8	6	5
P07093	HUMAN Glia-derived nexin	SERPINE2	-0.7	-1.5	-1.2	-0.7	-0.7	-0.4	-0.9	0.2	1	1
I3LUZ8	PIG Uncharacterized protein	LOC100516656	-0.4	-0.7	-1.6	-1.4	1.0	1.8	0.8	-1.3	3	4
Q6QRN9	PIG ADP/ATP translocase 3	SLC25A6	-1.0	-0.7	-1.3	-1.2	-2.3	-2.3	-1.1	0.4	21	15
F8W754	HUMAN Gamma-synuclein	SNCG	-0.4	-0.9	-1.4	-1.4	1.4	0.5	-0.7	-1.2	1	1
Q5GN48	PIG Dystrophin	DMD	-1.0	-1.3	-1.0	-0.8	-0.8	-0.7	-0.9	0.5	59	92
I3LJ70	PIG Uncharacterized protein	LOC100623035	-0.1	-0.4	-1.5	-2.2	-0.5	0.1	-1.1	-1.2	1	1
C9JDV5	HUMAN Putative uncharacterized protein C12orf77	C12orf77	-1.1	-1.2	-1.0	-1.0	-0.5	0.2	-0.3	0.1	1	1
I3LDR9	PIG Uncharacterized protein (Fragment)	SDPR	-1.2	-0.7	-1.1	-1.2	0.4	0.8	-0.5	-0.4	13	13
F1SKC4	PIG Uncharacterized protein (Fragment)	GYG1	-1.2	-1.3	-1.0	-0.7	2.0	1.0	0.2	-2.0	7	6
I3LHE4	PIG Coronin	NA	-0.5	-1.4	-1.4	-1.0	0.0	0.8	0.2	0.0	1	1
I3LE14	PIG Uncharacterized protein	CCDC58	-1.4	-1.4	-0.9	-0.4	0.5	1.0	0.5	-1.2	2	2
I3LUC9	PIG Uncharacterized protein	NA	-0.5	-0.9	-1.5	-1.2	-0.4	-0.4	-0.2	-0.7	1	1
F1RYK1	PIG Uncharacterized protein	SYT1	-0.9	-1.3	-1.2	-0.8	0.7	-0.2	0.6	0.7	2	2
G8FUN4	PIG Myomesin family member 3 (Fragment)	NA	-0.7	-1.2	-1.0	-1.2	-1.0	-1.5	-1.0	0.0	5	5
F1RKM1	PIG Uncharacterized protein (Fragment)	ALDH7A1	-0.9	-1.3	-1.1	-0.8	-0.9	-2.6	-1.2	-1.5	15	11
C1PIG4	PIG cAMP-dependent protein kinase regulatory subunit type II alpha	PRKAR2A	-0.7	-1.4	-1.2	-1.0	0.2	1.0	0.1	-1.4	10	11
I3LBT0	PIG Uncharacterized protein (Fragment)	NA	0.2	-1.5	-1.5	-1.4	0.3	-0.2	1.1	1.2	1	1
Q8WNW3	PIG Junction plakoglobin	Jup	-0.9	-1.3	-1.2	-0.8	-0.1	-0.1	-0.2	-0.1	13	22
F1RRC9	PIG Uncharacterized protein	NDUFC1	-1.2	-0.9	-0.9	-1.2	-0.9	-0.6	0.4	1.1	1	2
F1SBX0	PIG Uncharacterized protein	PCLO	-0.6	-0.7	-1.6	-1.2	-0.2	-0.2	0.2	0.6	3	3
F1S430	PIG Uncharacterized protein (Fragment)	LOC100525578	-0.4	-0.9	-1.7	-1.3	-0.2	-0.2	-1.3	-1.2	1	3
F1SDX6	PIG Uncharacterized protein	TGM2	-1.6	-1.8	-0.8	0.0	-1.4	-1.6	-1.2	-0.1	26	25
F1S0S9	PIG Uncharacterized protein	PAPD7	-0.7	-1.4	-1.2	-1.0	0.8	0.6	0.1	0.8	1	1
Q8TC94	HUMAN Actin-like protein 9	ACTL9	-1.4	-1.3	-0.9	-0.7	-1.1	0.1	-0.6	-1.6	1	1

F1SLP8	PIG Uncharacterized protein (Fragment)	NA	-0.6	-0.9	-1.2	-1.5	-1.7	-0.4	0.9	-0.3	1	2
F1RNS8	PIG Uncharacterized protein	PDAP1	-2.2	-1.4	-0.5	0.0	2.0	1.3	0.6	2.3	7	4
F1SM86	PIG Uncharacterized protein	EPB41L3	-1.0	-1.4	-1.1	-0.9	-0.3	0.3	-1.4	-1.0	11	13
I3LSI7	PIG Uncharacterized protein	POLR2E	-1.5	-1.6	-0.8	-0.4	-1.0	-0.8	0.4	-0.2	1	1
F1SND0	PIG Uncharacterized protein	SVEP1	-0.1	-1.1	-1.6	-1.5	-0.1	0.3	-1.0	-0.3	1	1
I3LH83	PIG Uncharacterized protein	NA	-1.2	-1.0	-1.2	-0.9	0.6	0.0	-0.1	0.2	1	1
F1RRP9	PIG Uncharacterized protein	NDUFB9	-0.9	-0.6	-1.2	-1.5	-0.9	-1.0	0.0	-0.4	6	5
P68371	HUMAN Tubulin beta-4B chain	TUBB4B	-1.1	-1.7	-0.9	-0.5	-0.4	-0.4	-0.3	-0.5	1	1
K9IW80	PIG Nicotinamide nucleotide transhydrogenase	NNT	-0.8	-0.5	-1.4	-1.5	-1.7	-1.2	-1.2	0.0	14	14
Q5VXU9	HUMAN Uncharacterized protein C9orf84	C9orf84	0.4	-1.3	-1.3	-2.0	-0.7	-1.4	-0.2	1.5	1	1
Q95283	PIG Cytochrome c oxidase subunit 4 isoform 1, mitochondrial (Fragment)	COX4I1	-1.0	-0.5	-1.1	-1.6	-1.6	-1.7	-0.9	0.0	10	10
B7Z6P1	HUMAN cDNA FLJ53662, highly similar to Actin, alpha skeletal muscle	NA	-0.9	-0.7	-1.3	-1.4	3.5	0.3	0.1	-1.1	2	1
F1S3H4	PIG Uncharacterized protein (Fragment)	COQ6	-0.2	-1.0	-1.6	-1.5	-0.1	0.3	-0.2	-0.5	7	7
Q4G176	HUMAN Acyl-CoA synthetase family member 3, mitochondrial	ACSF3	-0.7	-1.5	-1.4	-0.8	-1.3	-0.8	-0.4	-0.5	1	2
D6R9U4	HUMAN Ankyrin-2 (Fragment)	ANK2	-1.0	-0.9	-0.9	-1.5	0.5	0.6	0.8	1.1	1	1
Q8WWI1	HUMAN LIM domain only protein 7	LMO7	-1.4	-1.7	-0.9	-0.3	-1.3	-1.5	-0.6	0.1	5	6
K7GPB0	PIG Superoxide dismutase	SOD2	-0.5	-0.2	-1.7	-1.9	1.3	0.1	1.6	-1.0	3	3
F1S4N2	PIG Uncharacterized protein	CYB5R1	-0.4	-1.2	-1.3	-1.4	-0.8	-1.3	-1.2	-0.6	8	9
Q00688	HUMAN Peptidyl-prolyl cis-trans isomerase FKBP3	FKBP3	-1.7	-1.9	-1.0	0.2	-0.1	0.2	0.5	0.7	2	2
Q0PIT9	PIG NAD(P)H-hydrate epimerase	APOA1BP	-0.7	-1.2	-1.4	-1.1	0.6	-0.1	1.6	0.6	6	6
Q9TV63	PIG Myosin-2	MYH2	-1.2	-0.7	-0.9	-1.5	-1.0	-0.7	-0.1	0.0	10	15
P05027	PIG Sodium/potassium-transporting ATPase subunit beta-1	ATP1B1	-1.6	-0.8	-1.0	-0.9	-1.3	-0.9	-1.1	-0.2	7	7
F1RRK1	PIG Uncharacterized protein	PCNXL3	-1.3	-0.7	-1.0	-1.3	0.3	12.0	0.0	0.2	1	1
F1RIP1	PIG Uncharacterized protein	GYS1	-0.9	-1.9	-0.9	-0.7	-0.4	-0.9	-0.6	-0.6	12	15
F1SKL2	PIG Uncharacterized protein	TST	-0.6	-1.2	-1.2	-1.3	-0.7	-1.1	-0.9	-0.5	7	10
F1RWW4	PIG Uncharacterized protein	PDLIM5	-1.1	-1.3	-1.2	-0.8	-0.5	-0.3	0.3	-0.1	14	17
F1RKQ9	PIG Uncharacterized protein	LOC100519294	-1.2	-1.2	-1.1	-0.9	0.2	0.1	-0.7	0.3	3	1
O95180	HUMAN Voltage-dependent T-type calcium channel subunit alpha-1H	CACNA1H	-0.8	-1.8	-0.8	-0.9	-0.1	-0.4	-0.3	-0.6	1	1
F1S4V5	PIG Uncharacterized protein	ABLIM1	-1.1	-1.3	-1.2	-0.7	-0.5	-0.3	0.7	0.4	19	25
Q29380	PIG Voltage-dependent anion-selective channel protein 3	VDAC3	-0.9	-0.7	-1.3	-1.6	-2.1	-2.0	-1.9	-1.6	10	9
F1S6C3	PIG Uncharacterized protein (Fragment)	NA	-0.6	-0.8	-1.6	-1.4	-1.1	-1.1	-1.1	-1.8	2	3
F1RK59	PIG Uncharacterized protein	MRPL40	-0.4	-0.7	-1.6	-1.8	1.5	1.0	-0.7	-1.2	3	3
F1RFP4	PIG Uncharacterized protein	BRI3BP	-0.4	-0.6	-1.5	-1.8	0.1	1.0	0.3	-0.9	1	1
B9ZVU2	HUMAN Aspartyl aminopeptidase (Fragment)	DNPEP	-0.2	-1.1	-1.5	-1.5	-0.7	-1.0	-0.7	1.1	1	1
K7GRG9	PIG Uncharacterized protein	TNS1	-0.7	-0.6	-1.6	-1.5	0.4	0.4	-0.8	-1.2	1	1
K7GRM6	PIG Uncharacterized protein (Fragment)	HSD17B10	-0.2	-1.3	-1.6	-1.4	0.1	0.2	-0.6	-1.6	1	1
P80021	PIG ATP synthase subunit alpha, mitochondrial	ATP5A1	-0.4	-0.2	-1.8	-2.0	-0.6	-0.7	0.9	-0.1	47	44
P50747	HUMAN Biotin--protein ligase	HLCS	-0.2	-0.6	-1.5	-2.1	-0.1	0.1	-1.6	-3.0	1	1
K7GL03	PIG Uncharacterized protein (Fragment)	NA	-0.6	-1.2	-1.2	-1.3	0.0	0.1	-1.0	-1.3	3	5
Q9H7C9	HUMAN Mth938 domain-containing protein	AAMDC	-1.2	-1.4	-0.7	-1.1	-1.0	-1.0	-0.4	-1.2	1	1

B8Q0B2	PIG Blood vessel epicardial substance	BVES	-0.7	-1.3	-1.3	-1.1	-0.7	-0.6	-0.6	0.4	3	8
F1S9K1	PIG Uncharacterized protein	LOC100739845	-0.7	-1.1	-1.4	-1.2	-0.2	-0.5	-0.5	0.3	5	7
Q007T0	PIG Succinate dehydrogenase [ubiquinone] iron-sulfur subunit, mitochondrial	SDHB	-0.6	-0.4	-1.4	-2.0	-0.3	-0.4	-0.2	0.3	12	12
P04179	HUMAN Superoxide dismutase [Mn], mitochondrial	SOD2	-1.7	-0.7	-1.1	-0.9	0.3	-0.6	0.8	0.3	1	1
I3LSL4	PIG Uncharacterized protein	NA	-1.0	-1.6	-1.0	-0.9	-1.2	-1.6	-0.2	0.5	1	1
I3LBE4	PIG Uncharacterized protein	NA	-0.8	-1.0	-0.8	-1.8	-1.5	-1.4	0.4	0.3	8	7
O60636	HUMAN Tetraspanin-2	TSPAN2	-0.7	-0.1	-1.5	-2.2	-0.1	-0.4	-0.2	-0.1	1	1
F1RTH3	PIG Uncharacterized protein	AIFM1	-0.7	-0.7	-1.4	-1.7	-1.4	-1.3	-1.0	0.2	24	27
F1SGD2	PIG Uncharacterized protein	PKP2	-1.0	-1.4	-1.1	-1.0	0.0	0.5	0.9	0.5	18	28
F1SQP4	PIG Uncharacterized protein (Fragment)	NDUFA12	-0.9	-1.1	-1.3	-1.2	-0.7	-0.7	-0.3	-0.5	9	11
F1RPU8	PIG Uncharacterized protein	MPC2	-0.8	-1.0	-1.3	-1.4	-1.8	-1.8	-1.9	-0.3	4	4
F1S9V1	PIG Uncharacterized protein	PLD4	-1.0	-0.7	-1.3	-1.5	-1.3	-0.5	-0.1	0.3	1	1
F1RS52	PIG Uncharacterized protein	MMAA	-0.9	-1.9	-0.9	-0.8	0.3	0.8	-0.3	-0.7	1	3
F1RGJ3	PIG Stress-70 protein, mitochondrial	HSPA9	-0.7	-1.6	-1.3	-0.9	0.6	1.0	0.7	-0.9	46	34
Q2EN80	PIG NADH dehydrogenase 1 beta subcomplex 6	LOC733605	-1.0	-0.7	-1.3	-1.5	-0.6	-0.7	-0.6	-0.5	2	4
A1XQT6	PIG MLC1f	MYL1	0.3	-1.5	-1.4	-1.8	2.1	-1.6	-2.7	-5.3	13	5
F1SAS2	PIG Uncharacterized protein	ACSS1	-0.2	-1.5	-1.3	-1.5	-0.2	0.4	0.8	1.6	16	19
I3LHA3	PIG Uncharacterized protein	ACAD8	-0.5	-1.1	-1.6	-1.4	0.2	-1.9	-0.5	0.5	3	1
F1SA66	PIG Uncharacterized protein	TIMM44	-0.3	-1.3	-1.6	-1.3	0.0	-0.4	-0.7	-0.1	13	20
F1SL07	PIG Uncharacterized protein	LOC100524622	-0.9	-0.7	-1.4	-1.6	-1.1	-1.3	-1.7	-0.7	18	19
B4DPN6	HUMAN cDNA FLJ51031, highly similar to ATP-dependent RNA helicase DDX1 (EC 3.6.1.-)	NA	-1.1	-1.6	-1.2	-0.7	-0.3	0.2	1.0	0.7	1	1
O00217	HUMAN NADH dehydrogenase [ubiquinone] iron-sulfur protein 8, mitochondrial	NDUFS8	-0.4	-0.4	-1.6	-2.2	-0.5	-0.1	0.0	-1.2	7	4
I3LVH8	PIG Uncharacterized protein (Fragment)	DSC2	-1.2	-1.1	-1.2	-1.1	0.5	0.4	-0.2	-0.1	3	5
G8IFA6	PIG Mitochondrial NADH dehydrogenase Fe-S protein 4	NA	-0.4	-0.2	-1.6	-2.3	-0.1	-0.2	-0.4	0.8	1	1
F1SMG3	PIG Uncharacterized protein (Fragment)	LOC100526144	-0.9	-0.6	-1.5	-1.5	-1.7	-1.7	-1.3	0.8	20	25
K7GLT8	PIG ATP synthase subunit beta	ATP5B	-1.0	-0.5	-1.5	-1.5	-0.7	-1.1	0.8	-0.3	3	2
I3LDM7	PIG Uncharacterized protein	GLO1	-1.1	-1.6	-1.1	-0.7	-0.3	0.1	-0.1	-1.5	8	7
I3LQ34	PIG Uncharacterized protein	TOMM70A	-0.7	-1.6	-1.2	-1.0	-0.4	-0.5	-0.6	0.3	2	2
F1SMX9	PIG Uncharacterized protein	CDH7	-0.6	-1.2	-1.3	-1.4	-2.6	-2.7	-2.3	-1.3	1	1
F1SMZ7	PIG Uncharacterized protein	HSPD1	-1.3	-0.8	-1.2	-1.2	0.0	0.1	-0.3	-1.5	33	29
F1SA62	PIG Uncharacterized protein (Fragment)	LOC100517634	-1.2	-0.5	-1.4	-1.5	-1.1	-1.2	-1.1	-0.6	9	13
I3LKV0	PIG Uncharacterized protein (Fragment)	SLIRP	-1.0	-1.5	-1.1	-0.9	0.1	-0.4	-0.9	-0.4	1	4
P12682	PIG High mobility group protein B1	HMGB1	-2.3	-1.7	-0.4	-0.2	-0.7	0.1	0.2	1.0	9	6
F1RT59	PIG Uncharacterized protein	SGCA	-1.4	-1.5	-1.2	-0.6	0.3	0.5	-0.3	0.7	3	5
I3LQT4	PIG Uncharacterized protein	NA	-1.5	-1.8	-1.0	-0.3	-1.5	-1.2	-0.4	0.1	1	1
F1SDF9	PIG Uncharacterized protein	SV2A	-1.2	-0.6	-1.5	-1.3	-0.8	-0.8	-0.9	0.0	1	2
F1SU52	PIG Uncharacterized protein	LOC100522130	-1.3	-1.1	-1.3	-0.9	-0.6	-1.2	-1.0	-0.7	8	7
F1SGC6	PIG Uncharacterized protein	NDUFB5	-1.1	-0.8	-1.2	-1.5	-1.9	-1.8	-1.7	-1.1	8	9
F1RPD7	PIG Uncharacterized protein	SMTN	-1.6	-1.9	-1.0	-0.1	0.5	1.3	0.5	0.3	2	1
Q6RVA9	PIG Caveolin-1	CAV1	-1.6	-1.1	-1.1	-0.8	-1.5	-0.4	-0.7	1.1	7	8

P09623	PIG Dihydrolipoyl dehydrogenase, mitochondrial	DLD	-0.7	-0.5	-1.6	-1.8	-0.9	-1.1	-0.4	-0.3	16	17
P63317	PIG Troponin C, slow skeletal and cardiac muscles	TNNC1	-0.6	-0.1	-2.0	-1.9	2.3	0.4	-1.6	-7.1	12	9
Q8WXX0	HUMAN Dynein heavy chain 7, axonemal	DNAH7	-0.6	-0.8	-1.5	-1.7	-0.6	-0.1	0.1	0.6	1	1
F1RHI3	PIG Uncharacterized protein	VPS53	-1.8	-1.8	-0.4	-0.6	-0.4	-0.9	-0.7	-0.6	1	1
O43920	HUMAN NADH dehydrogenase [ubiquinone] iron-sulfur protein 5	NDUFS5	-1.6	-1.0	-0.9	-1.1	0.3	0.5	0.4	0.7	1	1
F1RF09	PIG Uncharacterized protein	MSH3	-1.1	0.4	-1.8	-2.2	1.5	0.5	0.2	-0.2	1	1
F1RIN7	PIG Uncharacterized protein	HRC	-0.7	-0.4	-1.9	-1.7	1.5	-0.5	-2.0	0.6	10	12
F1SB53	PIG Uncharacterized protein (Fragment)	PSMC2	-3.9	-1.5	-0.1	0.8	2.9	2.2	0.6	-0.4	2	1
F1SV04	PIG Uncharacterized protein (Fragment)	NT5C1A	-0.9	-1.5	-1.2	-1.0	-0.5	-0.5	-0.4	-1.3	7	10
F1ST31	PIG Uncharacterized protein	LOC100620565	-2.2	-0.8	-0.6	-1.1	-0.8	-0.4	2.4	-0.3	1	1
Q9P275	HUMAN Ubiquitin carboxyl-terminal hydrolase 36	USP36	-1.3	-0.5	-1.6	-1.2	-1.4	-1.3	-1.4	-0.3	1	1
B3TFD9	PIG Poliovirus receptor-related 2 transcript variant delta	PRR2	0.0	-1.0	-1.5	-2.1	0.9	-0.8	-1.5	-2.2	1	1
Q5VUA4	HUMAN Zinc finger protein 318	ZNF318	-1.1	-1.9	-1.3	-0.4	-1.1	-0.2	0.1	0.7	1	1
Q96LB3	HUMAN Intraflagellar transport protein 74 homolog	IFT74	-1.8	-1.1	-1.1	-0.8	-1.5	-1.2	-0.8	-0.9	1	1
F1SIF2	PIG Uncharacterized protein	NDUFS3	-1.0	-0.6	-1.4	-1.7	-1.1	-1.8	-2.4	-2.7	14	14
F1S1B9	PIG Uncharacterized protein	DHRS11	-1.1	-1.6	-1.2	-0.8	-0.5	0.3	0.2	-0.1	6	7
P57679	HUMAN Ellis-van Creveld syndrome protein	EVC	-1.5	-1.1	-1.1	-1.1	-0.9	-0.9	-1.5	-1.2	1	1
I3LR66	PIG Uncharacterized protein (Fragment)	NA	-1.1	-1.2	-1.3	-1.0	-0.3	-0.3	-0.6	-0.3	6	12
F1SBN7	PIG Uncharacterized protein	ATP5F1	-1.0	-0.5	-1.5	-1.6	-2.1	-1.7	-1.4	0.3	15	14
Q9XSE7	PIG Sarcolumenin (Fragment)	CBPG	-1.1	-0.5	-1.4	-1.8	-1.1	-1.8	-2.8	-1.7	9	9
B5L0Y2	PIG Calpastatin	CAST	-1.0	-1.2	-1.5	-1.0	0.5	1.6	0.1	0.8	1	1
F1SNL5	PIG Uncharacterized protein	LOC100622200	-1.0	-0.9	-1.6	-1.2	-0.3	-0.1	0.5	0.6	3	5
P13618	PIG ATP synthase-coupling factor 6, mitochondrial	ATP5J	-1.0	0.3	-1.8	-2.2	1.9	0.0	0.0	0.1	11	10
I3LQ99	PIG Uncharacterized protein	MRPS36	-1.2	-0.4	-1.4	-1.8	-0.4	-1.2	-1.0	0.4	5	5
I3LUE8	PIG Uncharacterized protein (Fragment)	IARS2	-0.7	-1.5	-1.3	-1.2	-0.1	0.0	-0.5	-0.2	7	12
F1RYH2	PIG Uncharacterized protein	NA	-0.9	-1.0	-1.5	-1.4	-2.5	-1.8	-1.9	-1.2	1	1
A9X3T3	PIG Peroxisomal D3,D2-enoyl-CoA isomerase	PECI	-0.8	-1.3	-1.6	-1.0	-0.4	-0.3	-2.0	-1.5	1	1
F1SLG5	PIG Uncharacterized protein	ACTG2	-0.4	-0.7	-1.7	-2.0	-0.6	-0.4	0.2	0.1	4	4
I3L704	PIG Anamorsin	CIAPIN1	-1.1	-2.3	-1.0	-0.4	1.6	0.5	0.6	0.9	2	1
Q7L590	HUMAN Protein MCM10 homolog	MCM10	-1.2	-1.5	-1.3	-0.8	0.4	0.3	0.7	-0.4	1	1
Q6ZMB5	HUMAN Transmembrane protein 184A	TMEM184A	-0.8	-1.2	-1.6	-1.2	-1.3	-0.5	-0.8	-0.7	1	1
P00355	PIG Glyceraldehyde-3-phosphate dehydrogenase	GAPDH	-1.4	-2.1	-0.8	-0.6	-0.8	-1.1	0.0	0.1	31	35
Q17RP2	HUMAN Tigger transposable element-derived protein 6	TIGD6	-1.8	-1.2	-0.4	-1.4	4.6	2.9	-0.6	0.1	1	1
F1S5H3	PIG Uncharacterized protein	VWA2	-0.7	-1.3	-1.3	-1.6	-0.7	-0.9	0.3	0.8	1	1
F1S9S8	PIG Uncharacterized protein	NEXN	-1.9	-1.5	-0.8	-0.6	0.9	0.3	-0.9	-0.6	28	15
Q2EN76	PIG Nucleoside diphosphate kinase B	NME2	-2.5	-2.1	-0.5	0.3	-1.3	-1.4	0.3	-1.5	9	8
F1SD73	PIG Uncharacterized protein	NDUFB1	-1.1	-0.6	-1.5	-1.6	-1.1	-0.9	-0.9	-0.3	2	1
A5GFN3	PIG CCCTC-binding factor (Zinc finger protein)-like	CTCFL	-1.1	-0.5	-1.5	-1.7	-0.7	0.1	0.3	-0.1	1	1
F1S946	PIG Olfactory receptor	LOC100512292	-0.6	-1.3	-1.4	-1.5	-0.4	-0.3	0.4	-0.5	1	1
D2JYW4	PIG F-actin capping protein beta subunit variant II	CAPZB	-0.6	-1.9	-1.3	-1.0	0.1	0.8	0.1	-0.3	3	3

F1SLI3	PIG Microtubule-associated protein	MAP4	-2.0	-1.9	-0.9	-0.1	0.3	0.6	0.3	0.8	6	7
I3LG33	PIG Uncharacterized protein (Fragment)	LOC100627907	-0.8	-1.4	-1.3	-1.4	-0.7	-0.4	-0.4	-0.3	4	6
I3L806	PIG Uncharacterized protein (Fragment)	NA	-1.5	-1.5	-1.2	-0.6	1.0	1.4	0.6	0.7	16	24
K7GR50	PIG Uncharacterized protein (Fragment)	GSTZ1	-0.7	-1.5	-1.3	-1.4	-1.0	-1.9	-2.2	-0.6	1	1
F1S563	PIG Uncharacterized protein	DBT	-0.8	-0.9	-1.6	-1.7	-0.3	-1.2	-1.8	-1.2	5	4
Q5XLD2	PIG Myosin regulatory light chain 2	HUMMLC2B	-0.9	-0.4	-1.5	-2.1	-0.7	-0.5	3.2	0.9	4	2
I3L5M6	PIG Uncharacterized protein	TRIM63	-1.6	-1.7	-1.1	-0.5	-0.3	0.1	-0.7	0.5	1	1
I3LA61	PIG Uncharacterized protein	GNAT1	-0.2	-1.2	-1.8	-1.7	0.2	-0.4	-1.3	-1.1	2	1
F1RFD4	PIG Uncharacterized protein	USMG5	-0.8	-1.2	-1.5	-1.4	-0.5	-0.3	-1.2	-2.0	2	2
I3LD55	PIG Uncharacterized protein (Fragment)	COQ3	-1.3	-1.1	-1.4	-1.1	-1.2	-1.2	-1.4	-1.5	7	7
I3L8Q0	PIG Uncharacterized protein	NA	-1.4	-1.8	-1.0	-0.7	-0.4	-0.2	0.9	1.4	88	111
F1SLB6	PIG Uncharacterized protein (Fragment)	TIMELESS	-1.1	-1.2	-1.8	-0.8	-0.7	0.1	0.4	0.2	1	1
I3LKP0	PIG Uncharacterized protein (Fragment)	SYNM	-1.0	-1.5	-1.3	-1.2	0.0	-0.5	-1.0	-1.1	27	34
Q1W0Y2	PIG Cytochrome c oxidase subunit 7C, mitochondrial	COX7C	-1.2	-1.1	-1.1	-1.6	-2.2	-1.4	-0.7	0.8	2	2
F1S4Z9	PIG Uncharacterized protein	SNTA1	-0.8	-1.5	-1.3	-1.3	0.3	0.8	0.0	-0.3	7	10
I3LQM9	PIG Uncharacterized protein	NA	-1.6	-1.0	-0.8	-1.5	-1.0	-0.1	3.2	0.1	1	1
I3LAI1	PIG Uncharacterized protein (Fragment)	ECHS1	-0.6	-1.3	-1.4	-1.5	-0.9	-0.6	-1.6	-0.7	1	2
F1RQQ8	PIG Phosphorylase	PYGM	-1.0	-1.8	-1.2	-0.9	-1.0	-0.8	-0.2	-0.6	31	38
P12524	HUMAN Protein L-Myc	MYCL	-1.0	0.1	-1.9	-2.3	-0.3	-0.2	0.0	0.3	1	1
Q9TV62	PIG Myosin-4	MYH4	-0.8	-1.1	-1.5	-1.5	-3.2	-2.8	-1.9	0.1	5	3
Q8N7F3	HUMAN CDNA FLJ25696 fis, clone TST04563	NA	-1.3	-1.1	-1.4	-1.2	-2.1	-0.8	-1.5	1.3	1	1
F1RY71	PIG Uncharacterized protein	IGHMBP2	-0.7	-1.0	-1.4	-1.8	0.6	-0.7	1.6	3.2	1	3
F1SKY2	PIG Uncharacterized protein	NIT2	-1.2	-1.4	-1.4	-1.0	-0.1	-0.8	1.0	-1.2	8	9
P35573	HUMAN Glycogen debranching enzyme	AGL	-0.9	-2.1	-1.2	-0.8	-1.5	-2.3	0.0	0.0	2	4
Q9N0F1	PIG Succinyltransferase component of 2-oxoglutarate dehydrogenase complex, mitochondrial	DLST	-0.9	-0.5	-1.6	-1.9	0.7	0.6	2.2	-0.7	16	16
Q13404	HUMAN Ubiquitin-conjugating enzyme E2 variant 1	UBE2V1	-1.4	-2.1	-0.9	-0.5	-0.3	-0.7	-0.6	-0.1	2	2
Q8WZ42	HUMAN Titin	TTN	-1.3	-1.8	-1.1	-0.8	0.1	0.5	1.7	1.9	576	758
F2Z5I9	PIG Destrin (Fragment)	DSTN	-1.4	-1.6	-1.1	-0.9	-0.5	-0.6	0.0	-0.7	3	1
I3LTZ8	PIG Uncharacterized protein	PBLD	-1.2	-0.6	-1.2	-2.0	0.7	0.3	-0.9	-1.5	1	2
Q14BN4-8	HUMAN Isoform 8 of Sarcolemmal membrane-associated protein	SLMAP	-1.5	-1.3	-1.2	-0.9	-0.5	-0.6	0.2	1.4	2	2
Q9H7I8	HUMAN FLJ00096 protein (Fragment)	FLJ00096	-1.4	-0.9	-1.2	-1.5	0.9	2.4	2.0	-0.6	1	1
I3L9N1	PIG Uncharacterized protein	ADPRHL1	-1.4	-1.3	-1.0	-1.3	-0.6	-0.7	-1.2	-0.3	3	6
F1RMD6	PIG Uncharacterized protein	VPS36	-1.3	-1.8	-1.3	-0.6	0.7	1.1	-0.3	0.6	2	3
I3LI87	PIG Uncharacterized protein	LMAN1L	-0.7	-1.4	-1.4	-1.5	-1.4	-1.3	-2.6	-0.5	1	1
F1RZC1	PIG Uncharacterized protein	MYO5C	-0.6	-0.6	-1.8	-2.1	0.1	1.4	0.1	0.1	1	1
Q96EV2	HUMAN RNA-binding protein 33	RBM33	-0.9	-1.3	-1.2	-1.7	-1.7	-2.2	-0.3	0.6	1	1
O14958	HUMAN Calsequestrin-2	CASQ2	-1.1	-0.5	-1.6	-1.8	-0.1	0.1	-0.5	-0.2	1	1
I3LIQ2	PIG Uncharacterized protein	NA	-0.5	-1.0	-1.9	-1.7	-0.3	-1.3	-1.6	-0.7	2	3
S6BGF9	HUMAN IgG L chain	NA	-0.8	-1.5	-1.5	-1.3	0.3	0.3	0.4	0.9	1	1
Q4VK67	PIG Glycogen synthase 1 (Fragment)	NA	-0.9	-0.9	-1.5	-1.7	0.9	1.0	-0.7	-1.4	1	1

Q9P225	HUMAN Dynein heavy chain 2, axonemal	DNAH2	-1.3	-1.0	-1.4	-1.4	0.6	-1.3	-1.4	-2.0	4	1
F1RLG3	PIG Uncharacterized protein (Fragment)	APC	-0.6	-0.3	-1.6	-2.5	2.5	0.5	-1.6	-3.4	1	1
I3L8N7	PIG Uncharacterized protein (Fragment)	NA	-1.3	-1.3	-1.4	-1.1	-1.1	-1.4	-3.3	-4.1	1	1
Q9TV61	PIG Myosin-1	MYH1	-0.8	-1.1	-1.5	-1.7	-1.7	-1.6	-1.0	0.4	75	70
F1RPA3	PIG Uncharacterized protein (Fragment)	GPRC5B	-0.5	-1.7	-1.3	-1.6	0.0	-0.3	1.1	0.2	2	1
F1SJ34	PIG Uncharacterized protein	COX5A	-1.0	-0.2	-1.5	-2.4	0.6	-1.1	-2.4	-1.4	10	10
O94875-10	HUMAN Isoform 10 of Sorbin and SH3 domain-containing protein 2	SORBS2	-1.8	-1.5	-0.8	-1.0	-0.3	0.9	0.0	-0.1	1	1
A9JPD4	PIG Carnitine acetyl transferase	CRAT	-0.6	-1.5	-1.6	-1.3	-1.1	-1.1	-0.4	0.7	15	16
F1RVN1	PIG Uncharacterized protein	NDUFV1	-1.1	-0.8	-1.4	-1.7	-1.2	-1.0	-0.7	-1.3	17	16
F1RNZ0	PIG Uncharacterized protein	PLEKHF1	-3.0	-2.0	-0.4	0.3	-0.4	0.2	1.4	1.6	1	1
F1SJP6	PIG NADH dehydrogenase [ubiquinone] 1 alpha subcomplex subunit 6 (Fragment)	NDUFA6	-0.7	-0.7	-1.5	-2.2	-1.2	-0.7	-2.1	-1.2	7	7
F1SIL0	PIG Uncharacterized protein	CEP78	-1.1	-1.9	-1.2	-0.9	-0.9	-0.4	1.5	1.9	1	1
Q9NRS4	HUMAN Transmembrane protease serine 4	TMPRSS4	-0.1	-1.8	-1.6	-1.5	3.8	0.1	0.0	0.4	1	1
O75460-2	HUMAN Isoform 2 of Serine/threonine-protein kinase/endoribonuclease IRE1	ERN1	-0.7	-1.3	-1.8	-1.3	-1.1	-1.9	-1.4	-1.1	2	1
F1SDN2	PIG Uncharacterized protein	HADHB	-0.8	-1.2	-1.6	-1.5	-1.2	-0.5	-0.2	0.6	34	31
Q9H2U2	HUMAN Inorganic pyrophosphatase 2, mitochondrial	PPA2	-0.8	-1.1	-1.7	-1.6	-1.6	-0.3	-1.1	-1.4	1	1
C9J5M4	HUMAN Protein eva-1 homolog A (Fragment)	EVA1A	-0.8	-2.6	-1.2	-0.6	-0.4	-1.3	-2.0	-1.9	1	1
O43813	HUMAN LanC-like protein 1	LANCL1	-1.7	-1.8	-1.1	-0.7	-2.4	-1.9	-0.8	-1.2	1	1
Q4TTS4	PIG Histone H1.2-like protein	NA	-2.3	-1.0	-1.1	-0.7	-1.0	-0.9	-0.3	1.0	2	4
P08059	PIG Glucose-6-phosphate isomerase	GPI	-1.6	-2.3	-0.9	-0.4	-0.1	-0.3	2.3	1.1	19	19
Q9NWI7	HUMAN CDNA FLJ20825 fis, clone ADSE00160	NA	-1.1	-1.2	-1.3	-1.5	-0.9	-1.2	-0.9	-0.1	1	1
B4DI89	HUMAN TRAF-type zinc finger domain-containing protein 1	TRAFD1	-1.7	-0.5	-1.1	-1.9	-1.1	-1.0	1.1	0.4	1	1
F1S5K7	PIG Protein kinase C (Fragment)	PRKCE	-1.4	-1.5	-1.3	-1.0	-1.8	-1.1	-1.7	-1.2	2	2
S4R305	HUMAN Putative tyrosine-protein phosphatase auxilin (Fragment)	DNAJC6	-1.5	-0.7	-1.4	-1.6	1.3	8.7	0.4	-1.4	1	1
P14927	HUMAN Cytochrome b-c1 complex subunit 7	UQCRB	-1.2	-0.9	-1.2	-1.9	-1.1	-0.9	-0.8	-1.1	4	6
Q29554	PIG Trifunctional enzyme subunit alpha, mitochondrial	HADHA	-0.7	-1.2	-1.8	-1.4	-1.5	-0.1	-0.2	1.1	41	43
F1S557	PIG Uncharacterized protein	AGL	-1.1	-2.1	-1.1	-0.9	-1.1	-1.1	-0.4	-0.5	34	41
P12882	HUMAN Myosin-1	MYH1	-1.1	-0.7	-1.8	-1.7	6.3	1.4	3.0	3.9	3	1
F1SP14	PIG Uncharacterized protein	AMACR	-1.3	-1.5	-1.5	-1.0	-0.8	-1.2	-1.8	-0.3	2	4
P16066	HUMAN Atrial natriuretic peptide receptor 1	NPR1	-0.8	-1.0	-1.4	-2.0	-1.8	-1.0	-1.2	-0.3	1	1
F1SS61	PIG Uncharacterized protein	MYH3	-1.3	-1.2	-1.3	-1.4	-1.9	-1.3	-1.8	-0.7	11	10
D2KMN4	PIG Glial cell-line derived neurotrophic factor (Fragment)	GDNF	-1.5	-1.6	-1.2	-0.9	-0.9	-0.3	0.5	1.2	1	1
F1RGH4	PIG Uncharacterized protein	PROB1	-0.6	-1.5	-1.7	-1.5	-0.5	-0.3	-1.3	-0.4	4	3
F1S4D5	PIG Uncharacterized protein (Fragment)	CCBL2	-0.9	-1.5	-1.5	-1.4	-0.2	-0.4	1.2	0.3	7	6
Q8MHY0	PIG MYL2	MLC-2V	-1.1	0.0	-1.7	-2.5	0.8	-0.9	-2.4	-6.2	26	25
F1SC51	PIG Uncharacterized protein	NA	-1.3	-1.6	-1.5	-0.9	0.8	1.0	-0.7	-1.7	14	15
F1RG85	PIG Uncharacterized protein	MYO18B	-1.0	-1.4	-1.9	-1.1	-0.7	-0.2	-0.4	0.8	2	2
A4Z6H0	PIG Adenylosuccinate synthetase isozyme 1	ADSSL1	-0.5	-1.5	-1.7	-1.6	0.1	0.7	-0.8	-0.1	5	5
K9J4R6	PIG 6-phosphofructokinase	PFKP_tv1	-2.3	-1.8	-0.5	-0.8	-0.3	-0.9	-0.8	-0.5	3	3
Q00839	HUMAN Heterogeneous nuclear ribonucleoprotein U	HNRNPU	-2.8	-2.3	-0.7	0.5	-2.8	-0.6	-0.4	1.2	4	4

O94875-2	HUMAN Isoform 2 of Sorbin and SH3 domain-containing protein 2	SORBS2	-1.8	-1.8	-1.1	-0.6	0.2	0.9	0.0	-0.2	2	3
Q4L235	HUMAN Acyl-CoA synthetase family member 4	AASDH	-1.1	-2.1	-1.2	-0.8	-0.4	-0.7	0.2	0.0	1	1
Q9P2K8	HUMAN Eukaryotic translation initiation factor 2-alpha kinase 4	EIF2AK4	-0.9	-1.1	-1.6	-1.7	-0.4	0.0	-0.1	1.6	1	1
F1SIB9	PIG Uncharacterized protein	ARFGAP2	-1.3	-1.6	-1.3	-1.1	1.2	1.9	2.1	0.3	1	1
I3LDI0	PIG Uncharacterized protein (Fragment)	NA	-1.2	-1.4	-1.5	-1.3	1.6	1.3	0.2	-0.2	6	9
Q0D2Q6	HUMAN Phosphoglycerate mutase 1 (Brain)	PGAM1	-2.0	-2.1	-1.0	-0.2	0.0	0.0	1.1	0.1	1	1
Q14296-3	HUMAN Isoform 3 of Fas-activated serine/threonine kinase	FASTK	-1.5	-1.6	-1.4	-0.9	-0.4	0.0	-0.3	1.4	1	1
I3LLJ2	PIG Uncharacterized protein	NA	-0.8	-1.3	-1.6	-1.6	-0.5	-0.5	-0.7	0.0	1	1
F1SBU7	PIG Lon protease homolog, mitochondrial	LONP1	-1.1	-1.6	-1.4	-1.3	-0.8	-1.2	-0.5	0.0	13	15
Q9TQR6	PIG Phosphoglucomutase 1 (Fragment)	PGM1	-1.0	-1.9	-1.3	-1.1	0.9	0.3	2.5	1.3	9	9
Q92791	HUMAN Synaptonemal complex protein SC65	LEPREL4	-1.3	-1.4	-1.6	-1.1	-1.4	-1.0	-1.3	-1.0	1	1
F1RH92	PIG Uncharacterized protein	MYOT	-2.0	-2.2	-0.9	-0.3	-0.4	0.7	0.7	1.0	18	16
F1RPG1	PIG Uncharacterized protein	NA	-1.6	-1.7	-1.1	-1.0	-1.3	-0.5	0.6	1.5	1	1
Q14896	HUMAN Myosin-binding protein C, cardiac-type	MYBPC3	-2.2	-1.2	-1.0	-1.0	-2.1	-1.9	-1.7	-1.2	1	2
M3VHA4	PIG MICAL C-terminal like protein	MICALCL	-1.3	-0.7	-1.7	-1.7	0.4	0.5	0.7	-0.1	1	2
Q9NQR4	HUMAN Omega-amidase NIT2	NIT2	-1.0	-1.1	-1.8	-1.6	0.2	0.0	1.2	-0.4	1	1
F1SV23	PIG Uncharacterized protein	NDUFS5	-1.6	-1.1	-1.2	-1.4	0.1	-0.3	-0.8	-0.6	4	3
F1RK58	PIG Uncharacterized protein	HMOX2	-1.2	-1.4	-1.6	-1.2	0.5	0.0	-0.5	0.2	3	3
P10173	PIG Fumarate hydratase, mitochondrial	FH	-1.3	-0.8	-1.7	-1.7	-0.4	-0.5	-0.3	-0.4	28	26
I3LIQ8	PIG Uncharacterized protein (Fragment)	WDFY4	-1.3	-1.5	-1.5	-1.0	-2.4	-2.3	-1.6	0.1	1	1
I3LML9	PIG Uncharacterized protein	NA	-1.2	-1.7	-1.3	-1.2	-1.8	-0.8	1.0	-0.6	1	1
F1SRP8	PIG Uncharacterized protein (Fragment)	DENND2A	-1.1	-2.0	-1.4	-0.9	-0.5	-0.7	-0.9	-0.6	1	1
Q9TS51	PIG Myosin heavy chain (Fragment)	NA	-1.2	-1.2	-1.9	-1.1	-0.1	0.6	-0.9	0.3	1	2
P28220	PIG Sorbin and SH3 domain-containing protein 2	SORBS2	-2.1	-2.2	-0.7	-0.4	-0.9	0.5	0.7	0.6	7	7
Q9BYH1	HUMAN Seizure 6-like protein	SEZ6L	-1.4	-1.5	-1.6	-0.9	0.1	-0.7	0.6	0.5	1	1
F1S7K4	PIG Uncharacterized protein	PLIN4	-1.0	-1.7	-1.4	-1.3	0.5	-0.8	-0.4	2.0	18	33
Q29320	PIG Lactate dehydrogenase-B (Fragment)	NA	-1.0	-1.7	-1.5	-1.2	-0.6	-0.7	-0.1	-0.5	2	1
Q8N3V7	HUMAN Synaptopodin	SYNPO	-1.1	-1.6	-1.6	-1.2	-0.8	-0.4	0.0	-0.5	1	2
P36887	PIG cAMP-dependent protein kinase catalytic subunit alpha	PRKACA	-1.4	-2.2	-1.3	-0.6	-1.8	-1.5	-0.6	0.8	10	11
I3LHL4	PIG Uncharacterized protein (Fragment)	NDUFS7	-1.0	-1.1	-1.7	-1.7	-1.4	-1.4	-1.2	-0.1	8	9
Q29577	PIG Creatine kinase U-type, mitochondrial	CKMT1	0.0	-0.4	-2.3	-2.8	1.2	1.1	1.6	0.4	4	5
I3LDR2	PIG Uncharacterized protein (Fragment)	LOC100621514	-1.2	-1.4	-1.4	-1.4	0.0	-0.5	-1.0	-0.6	7	8
F1SHL9	PIG Pyruvate kinase (Fragment)	PKM	-1.7	-2.5	-1.0	-0.3	-1.2	-0.6	0.1	1.4	32	35
Q01167	HUMAN Forkhead box protein K2	FOXK2	-1.1	-1.9	-1.3	-1.2	-0.5	-1.0	-0.1	-0.8	1	1
P61013	PIG Cardiac phospholamban	PLN	-1.1	-1.3	-1.6	-1.5	-1.1	-1.2	-0.6	1.2	5	5
F1SS86	PIG Uncharacterized protein	DHRS7C	-1.2	-1.4	-1.5	-1.3	-1.0	-2.2	-1.8	-0.4	3	12
I3LTC0	PIG Uncharacterized protein (Fragment)	NA	-0.9	-0.8	-1.8	-1.9	-1.6	-1.7	-1.5	0.3	14	15
I3LRH7	PIG Uncharacterized protein	DSC3	-0.9	-2.0	-1.5	-1.1	0.3	-2.5	-2.4	-1.6	1	1
F1SLR1	PIG Uncharacterized protein	NDUFA8	-1.3	-0.7	-1.6	-1.9	-0.9	-1.3	-1.3	-1.1	6	6
F1RWI9	PIG Uncharacterized protein	LRRC46	-1.2	-1.6	-1.5	-1.2	-0.8	-0.8	-1.5	-0.5	1	1

F1RII4	PIG Uncharacterized protein	PARK7	-1.8	-1.7	-1.3	-0.8	-0.5	-0.7	-1.0	-1.7	9	8
P09913	HUMAN Interferon-induced protein with tetratricopeptide repeats 2	IFIT2	0.8	-1.3	-2.2	-2.9	1.2	0.0	-1.4	-2.4	1	1
B1Q0K1	PIG Tubulin polymerization promoting protein p25 alpha	TPPP	-1.8	-1.6	-1.3	-0.9	1.3	2.2	1.7	-1.1	1	1
F1SMB2	PIG Dihydrolipoyllysine-residue acetyltransferase	DLAT	-0.8	-0.8	-1.9	-2.0	-1.0	-1.1	-1.2	-1.2	18	18
Q17RW2	HUMAN Collagen alpha-1(XXIV) chain	COL24A1	-2.1	-2.0	-1.2	-0.3	1.0	0.2	-0.4	1.3	1	2
F1REZ9	PIG Uncharacterized protein	LOC100519366	-1.1	-0.5	-1.7	-2.4	-1.0	-0.8	-1.0	1.6	2	2
F1RSG9	PIG Uncharacterized protein (Fragment)	LOC100152491	-1.3	-1.5	-1.5	-1.2	-0.8	-0.7	-0.5	-0.3	3	3
A5GFS8	PIG Vesicle-associated membrane protein-associated protein B	VAPB	-1.3	-2.1	-1.3	-1.0	-0.7	-0.2	0.0	1.1	10	8
P11216	HUMAN Glycogen phosphorylase, brain form	PYGB	-0.7	-2.0	-1.6	-1.3	-1.5	-0.2	0.5	0.3	5	6
F1RIJ7	PIG Uncharacterized protein	TAS1R1	-1.3	-1.7	-1.5	-1.2	-0.6	0.1	1.5	1.7	1	1
F1S8H5	PIG Uncharacterized protein	APEX1	-1.5	-1.3	-1.5	-1.3	-0.1	-1.2	-0.1	1.1	3	4
F1RT96	PIG Uncharacterized protein	ACSF2	-1.5	-2.0	-1.4	-0.9	-1.2	-2.2	-2.1	-2.3	6	9
F1STL5	PIG Uncharacterized protein	TMEM200B	-1.4	-1.7	-1.3	-1.2	0.1	-0.9	-0.4	0.4	1	1
Q29582	PIG Pyruvate kinase M2 (Fragment)	NA	-1.1	-2.0	-1.6	-1.0	-0.5	-0.3	-0.5	0.7	1	1
P12675	PIG Calpastatin	CAST	-1.8	-1.7	-1.4	-0.8	1.7	1.6	0.6	0.3	20	22
Q9HAU0-2	HUMAN Isoform 2 of Pleckstrin homology domain-containing family A member 5	PLEKHA5	-1.9	-1.1	-1.5	-1.2	-1.9	-1.0	-2.0	-2.5	1	1
Q6ZU56	HUMAN cDNA FLJ43984 fis, clone TESTI4018886, weakly similar to M-protein, striated muscle	NA	-1.2	-0.9	-1.8	-1.8	-1.4	-1.5	1.4	0.9	1	1
P28768	PIG Superoxide dismutase [Mn], mitochondrial (Fragment)	SOD2	-1.4	-0.2	-1.8	-2.3	0.2	-0.4	1.6	0.6	7	8
O00330	HUMAN Pyruvate dehydrogenase protein X component, mitochondrial	PDHX	-0.5	-0.8	-2.0	-2.4	0.9	0.3	0.9	0.3	3	1
Q562R0	HUMAN Actin-like protein (Fragment)	ACT	-0.1	-1.3	-2.0	-2.4	-0.5	-1.1	-0.1	-0.7	1	1
F1SAW8	PIG Calsequestrin (Fragment)	CASQ2	-1.3	-0.7	-1.7	-2.0	-1.2	-1.5	-2.7	-2.8	6	6
Q2EN81	PIG ATP synthase subunit O, mitochondrial	ATP5O	-1.3	-0.7	-1.8	-2.1	-1.7	-1.6	-1.2	0.1	14	16
F1SP27	PIG Uncharacterized protein (Fragment)	IKBKAP	-0.9	-1.9	-1.8	-1.2	0.5	0.3	-0.2	-0.3	1	2
F1SRV4	PIG Uncharacterized protein	GSTK1	-0.8	-1.5	-1.8	-1.6	-0.9	-0.9	-0.5	-0.1	6	7
P05207	PIG cAMP-dependent protein kinase type II-alpha regulatory subunit (Fragment)	PRKAR2A	-1.6	-1.9	-1.3	-1.0	0.2	0.6	-0.1	-1.3	3	3
F1STY7	PIG Uncharacterized protein	AAMDC	-1.0	-1.8	-1.7	-1.3	0.2	-0.1	0.0	-0.8	3	3
Q8WNW4	PIG Beta-catenin	CTNNB1	-1.5	-1.8	-1.4	-1.1	-0.5	-0.6	-0.8	-0.5	19	18
Q9MZ15	PIG Voltage-dependent anion-selective channel protein 2	VDAC2	-1.2	-1.2	-1.7	-1.7	-2.0	-1.7	-1.3	-0.8	9	8
P38432	HUMAN Coilin	COIL	-1.7	-2.1	-1.2	-0.9	-0.2	0.0	1.2	1.7	1	1
Q0R678	PIG DJ-1 protein	PARK7	-1.6	-1.9	-1.4	-0.8	0.0	-0.5	-0.4	-1.4	3	4
F1RNZ1	PIG Cytochrome b-c1 complex subunit Rieske, mitochondrial	UQCRFS1	-1.3	-0.7	-1.6	-2.3	-1.6	-1.6	-1.4	-0.4	14	15
F1RL80	PIG Branched-chain-amino-acid aminotransferase	BCAT2	-0.7	-1.4	-1.9	-1.8	-1.1	-2.6	-2.1	-2.8	11	13
I3LPP1	PIG Fumarate hydratase, mitochondrial	FH	-0.9	-0.9	-1.9	-2.1	-1.2	-1.6	-1.0	-1.1	2	2
P00506	PIG Aspartate aminotransferase, mitochondrial	GOT2	-0.8	-0.8	-2.0	-2.3	-0.5	-1.4	1.6	-1.0	28	28
Q6MZQ5	HUMAN Putative uncharacterized protein DKFZp686F23130	DKFZp686F23130	-1.5	0.0	-2.0	-2.3	0.7	0.6	1.5	2.3	1	1
F1SLY2	PIG Uncharacterized protein	NDUFA5	-1.3	-0.5	-1.8	-2.2	-0.7	-0.4	-0.9	-1.7	4	5
F1SMR3	PIG Uncharacterized protein	PSIP1	-2.6	-1.6	-1.3	-0.4	-0.6	0.7	1.6	3.9	3	1
F1S5Y5	PIG Uncharacterized protein	STXBP3	-1.7	-1.8	-1.6	-0.8	-1.2	-2.0	-0.9	1.4	4	3
I3L873	PIG Uncharacterized protein	RAVER2	-0.7	-0.5	-2.1	-2.5	-2.6	-2.7	-2.7	-1.1	1	1
F1SK84	PIG Uncharacterized protein (Fragment)	DDX11	-2.1	-1.5	-1.3	-1.0	0.6	0.9	1.4	4.0	2	1

F1SIS9	PIG Uncharacterized protein	NDUFA10	-1.2	-0.8	-1.8	-2.1	-1.1	-0.8	-1.6	-2.0	13	14
G0W2Z1	PIG Troponin T type 2 (Cardiac) (Fragment)	TNNT2	-1.4	-0.4	-1.9	-2.2	-0.2	0.2	-0.4	0.8	4	3
F1S4B5	PIG Uncharacterized protein	PLEKHD1	-1.0	-1.6	-1.4	-1.8	-0.8	-0.7	1.6	1.9	1	1
I3L5C0	PIG Uncharacterized protein (Fragment)	VAPA	-2.0	-2.5	-1.1	-0.4	-0.3	0.0	0.5	1.3	3	4
F1S3Y7	PIG Uncharacterized protein (Fragment)	XDH	-1.5	-1.8	-1.4	-1.3	-1.9	-1.7	-0.6	-0.8	1	1
F1SNA8	PIG Uncharacterized protein	HSDL2	-1.0	-1.9	-1.7	-1.3	-1.5	-0.7	-0.8	0.3	11	12
F1S2E3	PIG Peptidyl-prolyl cis-trans isomerase	LOC100152612	-1.0	-1.2	-2.1	-1.7	-0.7	-0.4	0.0	0.4	7	8
F1RWZ4	PIG Uncharacterized protein	ECI2	-0.7	-1.9	-1.9	-1.5	-0.9	-0.9	-1.5	0.3	10	12
K7GND4	PIG Uncharacterized protein (Fragment)	HSD17B10	-1.1	-1.5	-1.7	-1.6	-0.8	-0.5	-0.4	-1.3	3	3
Q75ZZ6	PIG Troponin T, slow skeletal muscle	TNNT1	-1.2	-0.2	-2.1	-2.3	0.7	0.9	0.9	0.5	2	2
O95398	HUMAN Rap guanine nucleotide exchange factor 3	RAPGEF3	-2.0	-1.7	-0.9	-1.3	-0.7	-0.9	-1.4	0.6	1	2
P68137	PIG Actin, alpha skeletal muscle	ACTA1	-1.4	-1.1	-1.7	-1.7	-1.5	-1.7	-0.7	-0.7	37	38
A5X497	PIG Cardiac troponin I	TNNI3	-0.8	-0.5	-2.4	-2.2	0.0	0.7	0.9	0.4	22	22
P31641	HUMAN Sodium- and chloride-dependent taurine transporter	SLC6A6	-1.9	-1.9	-1.3	-0.9	-0.8	-0.1	0.3	0.5	1	1
F1S418	PIG Uncharacterized protein	PRDX3	-1.3	-0.6	-1.9	-2.2	0.6	-1.0	-1.2	-4.4	8	6
Q7RTW8	HUMAN Otoancorin	OTOA	-2.2	-1.5	-1.2	-1.0	-1.5	-0.9	-0.8	0.9	1	1
P07951	HUMAN Tropomyosin beta chain	TPM2	-0.6	0.1	-2.6	-2.8	3.0	1.7	-1.2	-5.4	2	2
A5D8V7	HUMAN Coiled-coil domain-containing protein 151	CCDC151	-1.2	-0.8	-2.0	-2.0	-1.1	-0.7	0.0	0.5	1	2
P54296	HUMAN Myomesin-2	MYOM2	-1.3	-1.7	-1.4	-1.5	-1.2	-2.6	-0.6	-0.6	6	3
Q5G6W0	PIG Cofilin 2 (Fragment)	NA	-2.1	-2.2	-1.1	-0.6	-0.3	-0.3	-0.6	-2.2	2	1
Q9NSD9	HUMAN Phenylalanine--tRNA ligase beta subunit	FARSB	-1.6	-2.0	-1.4	-1.0	0.4	0.6	-0.7	-0.2	1	2
F1S5J1	PIG Uncharacterized protein	LRPPRC	-1.7	-2.2	-1.3	-0.9	-1.5	-1.2	-0.3	0.9	1	1
F1RZQ8	PIG Uncharacterized protein	LEMD2	-1.4	-1.8	-1.5	-1.3	2.2	-0.8	-0.4	-0.1	3	1
I3LLH8	PIG Uncharacterized protein	ECI1	-0.9	-1.4	-2.0	-1.7	-0.8	-0.8	-0.2	0.5	6	6
I3L6X6	PIG Uncharacterized protein (Fragment)	NA	-1.1	-1.9	-1.8	-1.2	-0.3	-0.1	1.6	1.0	3	2
F1SHV9	PIG Uncharacterized protein	NA	-1.5	-2.2	-1.6	-0.7	-0.6	-0.8	-0.3	-0.9	2	2
P06576	HUMAN ATP synthase subunit beta, mitochondrial	ATP5B	-0.7	-0.8	-1.8	-2.7	-0.6	-1.0	-0.9	-1.6	1	1
Q6LBZ1	HUMAN MRNA for apolipoprotein E (apo E) (Fragment)	NA	-0.5	-1.5	-1.9	-2.1	0.0	0.4	-0.9	0.2	2	3
F1SAB6	PIG Acyl carrier protein	NDUFAB1	-1.7	-0.3	-1.9	-2.2	0.6	-1.0	-3.1	-5.9	3	3
Q1G1K7	PIG Mitochondrial NAD+isocitrate dehydrogenase 3 beta variant 2	IDH3B	-0.8	-1.8	-1.9	-1.6	-0.6	-0.8	-0.9	-0.6	18	18
Q9UHR6	HUMAN Zinc finger HIT domain-containing protein 2	ZNHIT2	-1.5	-1.6	-1.4	-1.6	-2.3	-2.3	-1.8	0.2	1	1
F1RHA0	PIG Uncharacterized protein (Fragment)	LOC100738911	-0.6	-1.8	-1.7	-2.0	-0.3	-1.1	-2.2	-2.5	1	1
P51668	HUMAN Ubiquitin-conjugating enzyme E2 D1	UBE2D1	-1.9	-2.0	-1.1	-1.1	0.6	-0.2	-0.3	0.7	1	1
P11177	HUMAN Pyruvate dehydrogenase E1 component subunit beta, mitochondrial	PDHB	-1.7	-1.8	-1.5	-1.1	0.3	-0.3	0.2	-0.3	1	1
Q5VST9	HUMAN Obscurin	OBSCN	-1.9	-2.0	-1.2	-0.9	-0.4	-1.2	-0.5	-0.3	10	10
F1S197	PIG Orexin	PPOX	-1.5	-1.8	-1.5	-1.3	0.3	-0.3	-0.4	0.5	1	2
F1RW75	PIG Uncharacterized protein	DSP	-1.6	-2.1	-1.4	-1.1	-0.7	-0.5	-0.1	0.3	76	115
F1S6H0	PIG Uncharacterized protein	KIRREL3	-1.6	-1.4	-1.5	-1.7	-0.8	-0.1	-0.6	0.2	2	1
F1RXF3	PIG Uncharacterized protein	DECR1	-0.6	-1.9	-1.8	-1.7	-1.0	-1.7	0.1	-1.0	13	14
F1RT47	PIG Uncharacterized protein	LOC100519752	-1.3	-1.8	-1.6	-1.4	-1.3	-2.1	-1.4	-0.6	1	4

P11607-2	PIG Isoform SERCA2A of Sarcoplasmic/endoplasmic reticulum calcium ATPase 2	ATP2A2	-0.5	-1.6	-1.9	-2.1	-0.4	-0.4	-0.6	0.0	1	1
I3LDC1	PIG Succinate dehydrogenase [ubiquinone] iron-sulfur subunit, mitochondrial	SDHB	-1.4	-0.9	-1.8	-2.0	-1.3	-0.7	-0.4	0.9	2	2
F1SM75	PIG Uncharacterized protein	MYOM1	-1.4	-2.0	-1.4	-1.4	-1.3	-1.6	-1.2	0.0	86	99
E5RJM6	HUMAN Ankyrin repeat domain-containing protein 65	ANKRD65	-2.5	-1.3	-1.1	-1.3	-2.6	-1.7	-0.8	2.5	3	1
M4MEX3	PIG NLRC5	NLRC5	-1.7	-1.8	-1.6	-1.2	4.8	13.9	12.1	-1.5	1	1
I3LN63	PIG Uncharacterized protein	MRPL54	-0.8	-1.1	-2.0	-2.3	1.0	-0.6	-2.1	-2.8	1	1
P80276	PIG Aldose reductase	AKR1B1	-1.7	-2.4	-1.1	-0.9	-0.6	0.1	0.2	-0.5	6	8
F1RUE0	PIG Uncharacterized protein	ACOT13	-0.5	-1.4	-2.1	-2.2	-1.3	-1.3	-1.0	-1.0	4	4
Q9H4Q3	HUMAN PR domain zinc finger protein 13	PRDM13	-1.0	-1.6	-1.8	-1.8	-2.8	-2.9	-1.7	-1.1	1	1
F1STY1	PIG NADH dehydrogenase [ubiquinone] 1 subunit C2	NDUFC2	-2.0	-1.0	-1.3	-1.9	-1.9	-1.5	-1.3	0.0	6	8
P80272	PIG Non-histone chromosomal protein HMG-17	HMGN2	-2.9	-0.7	-1.8	-0.8	-0.3	-0.8	-0.7	5.9	3	4
F1RK10	PIG Succinyl-CoA ligase [ADP-forming] subunit beta, mitochondrial	SUCLA2	0.2	-2.1	-2.2	-2.0	-0.8	-0.6	-1.5	-0.5	2	2
A1XQT2	PIG Cytochrome c oxidase subunit 6C	COX6C	-1.8	-0.9	-1.6	-1.9	-2.3	-1.8	-0.7	0.8	13	12
A1X899	PIG Beta-tropomyosin	TPM2	-1.7	-0.6	-2.3	-1.6	3.2	1.1	-1.3	-6.1	19	11
P15918	HUMAN V(D)J recombination-activating protein 1	RAG1	-1.3	-1.0	-1.6	-2.4	-0.8	-0.6	-1.2	-1.9	1	1
F8WD11	HUMAN Mitochondrial fission 1 protein	FIS1	-1.3	-2.1	-1.4	-1.5	-1.8	-2.4	-1.4	-0.7	2	1
Q96HC4	HUMAN PDZ and LIM domain protein 5	PDLIM5	-1.5	-1.3	-1.8	-1.8	-0.6	-0.8	0.1	-0.7	2	5
I3LMS3	PIG Uncharacterized protein (Fragment)	NA	-1.5	-2.3	-1.6	-1.0	0.4	1.1	0.6	1.1	1	3
F1RUE3	PIG Uncharacterized protein	ALDH5A1	-1.1	-1.6	-2.0	-1.7	-1.3	-1.0	-1.5	-0.6	8	11
O00763	HUMAN Acetyl-CoA carboxylase 2	ACACB	-1.7	-1.8	-1.8	-1.1	0.1	0.7	0.6	1.3	2	1
I3LDH5	PIG Uncharacterized protein	AOX1	-1.3	-1.7	-1.5	-1.8	-2.0	-1.4	-0.7	0.8	1	2
F1RSP5	PIG Uncharacterized protein	LOC100524618	-1.5	-1.3	-1.7	-1.9	0.3	-1.2	-0.1	-1.6	8	9
O18934	PIG Calsequestrin-2 (Fragment)	CASQ2	-1.8	-0.9	-1.7	-2.0	-1.7	-1.9	-2.7	-2.7	6	5
Q9H7N4	HUMAN Splicing factor, arginine/serine-rich 19	SCAF1	-1.8	-1.4	-1.7	-1.4	-1.5	-2.1	-1.8	0.6	1	1
O94875	HUMAN Sorbin and SH3 domain-containing protein 2	SORBS2	-1.9	-2.3	-1.4	-0.9	0.2	0.9	0.7	0.0	14	15
F1SQT3	PIG Uncharacterized protein (Fragment)	SLC25A3	-1.7	-1.1	-1.8	-1.8	-3.0	-2.2	-1.7	-0.4	22	21
P29376	HUMAN Leukocyte tyrosine kinase receptor	LTK	-1.4	-2.0	-1.6	-1.3	-0.4	0.0	1.2	2.6	1	1
F1SMF9	PIG Uncharacterized protein	LOC100525437	-1.9	-0.5	-1.5	-2.5	-1.3	-1.0	-1.1	0.1	6	6
I3L670	PIG Uncharacterized protein	NA	-1.5	-2.4	-1.7	-0.9	-1.7	-4.3	-1.5	-1.0	2	2
A6PVY3	HUMAN Protein FAM177B	FAM177B	-2.2	-1.8	-1.5	-1.0	-2.6	-0.6	-0.6	-0.2	1	1
F2Z5H2	PIG Uncharacterized protein (Fragment)	ATAD1	-1.7	-1.9	-1.7	-1.3	-1.1	-1.2	-0.1	-0.1	2	3
Q14160	HUMAN Protein scribble homolog	SCRIB	-1.7	-1.2	-2.0	-1.7	-0.2	-1.7	-0.4	-0.7	1	2
F1RRP8	PIG Uncharacterized protein	TMEM65	-1.7	-1.4	-1.8	-1.6	-0.8	-0.3	-0.9	-0.4	4	5
F1SUJ6	PIG Uncharacterized protein	CTNNA3	-1.2	-1.7	-1.9	-1.6	-0.7	-0.9	-0.4	0.0	1	1
I3LLE3	PIG Uncharacterized protein (Fragment)	LOC100520636	-1.0	-1.8	-1.9	-1.7	-1.3	-1.1	-0.1	0.9	27	32
A8KA84	HUMAN highly similar to Homo sapiens 2'-5'-oligoadenylate synthetase 3, 100kDa (OAS3)	NA	-2.2	-2.2	-1.4	-0.7	-2.2	-2.0	-1.3	0.0	1	1
F1SUT4	PIG Uncharacterized protein	RELT	-1.5	-1.5	-1.8	-1.7	-1.4	-1.0	-1.3	-0.5	1	1
F1SJU8	PIG Uncharacterized protein (Fragment)	PHF21B	-1.6	-1.7	-1.6	-1.6	-0.5	-0.8	0.6	0.1	1	1
P79273	PIG Short-chain specific acyl-CoA dehydrogenase, mitochondrial	ACADS	-1.1	-1.9	-1.8	-1.7	-0.5	-0.9	-0.9	-0.8	10	11
F1RQQ7	PIG Phosphorylase (Fragment)	PYGB	-1.5	-2.3	-1.6	-1.2	-1.0	-0.3	0.1	0.7	44	44

E7EBY5	PIG MACRO domain containing protein 1	MACROD1	-1.4	-2.0	-1.8	-1.4	-0.6	-0.7	-0.8	-1.2	5	4
F1RRL4	PIG Uncharacterized protein	LOC100739861	-2.4	-2.0	-1.7	-0.5	-3.2	-3.2	-2.2	-0.6	1	1
Q6F5E7	HUMAN Protein TXNRD3NB	TXNRD3NB	-1.0	-1.7	-2.1	-1.8	-2.0	-1.5	-0.6	-0.4	1	1
Q7M337	PIG Creatine kinase chain M2 (Fragment)	NA	-2.4	-1.8	-1.4	-1.0	-1.4	-1.8	-1.0	-0.8	1	1
F1SDC7	PIG Uncharacterized protein	ALDH3A2	-2.2	-2.1	-1.4	-0.9	-2.3	-2.0	-2.5	-0.2	9	10
Q2HYU1	PIG Mitochondrial creatine kinase 2	CKMT2	-0.6	-1.0	-2.4	-2.6	1.2	0.7	1.5	0.0	13	14
I3LES8	PIG Uncharacterized protein (Fragment)	NA	-0.8	-1.9	-2.0	-2.0	-1.2	-1.2	-1.0	1.0	5	6
Q8HZQ4	PIG Thioredoxin reductase (Fragment)	TrxR1	-1.9	-1.9	-1.8	-1.0	-1.5	-1.2	-0.6	0.5	1	2
K7GM59	PIG Uncharacterized protein (Fragment)	ABCD1	-2.4	-2.2	-1.4	-0.7	-2.3	-1.5	0.0	-1.1	1	1
O60934	HUMAN Nibrin	NBN	-0.9	-1.0	-2.4	-2.4	-2.5	-2.2	-1.7	-1.6	2	2
F1RKG8	PIG Uncharacterized protein	PEBP1	-2.0	-2.2	-1.4	-1.1	-0.2	0.6	1.5	-1.4	9	9
P53590	PIG Succinyl-CoA ligase [GDP-forming] subunit beta, mitochondrial (Fragment)	SUCLG2	-0.9	-1.9	-2.1	-1.8	-0.1	0.0	-0.2	-1.4	21	19
C3RZA5	PIG SET and MYND domain-containing 1	SMYD1	-1.5	-2.5	-1.5	-1.2	-1.4	-1.8	-0.4	-0.4	10	14
F1SSG2	PIG Uncharacterized protein	TBC1D2	-1.8	-1.3	-1.5	-2.1	-1.8	-2.9	-0.7	1.6	2	1
Q66GS9	HUMAN Centrosomal protein of 135 kDa	CEP135	-1.3	-1.2	-2.2	-2.1	0.5	0.8	0.4	1.1	1	1
I3LU12	PIG Uncharacterized protein	NA	-1.9	-2.1	-1.6	-1.1	-0.2	-0.3	0.4	0.8	1	1
F1S3H1	PIG Uncharacterized protein	ALDH6A1	-0.9	-1.9	-2.0	-1.9	-0.4	-0.6	-1.3	-1.1	21	20
F1SE25	PIG Uncharacterized protein (Fragment)	NA	-1.6	-2.3	-1.7	-1.2	-1.0	-0.3	0.3	0.8	1	2
Q13470	HUMAN Non-receptor tyrosine-protein kinase TNK1	TNK1	-1.4	-2.1	-1.9	-1.4	-0.3	0.2	-0.8	-1.0	1	1
Q9BYX7	HUMAN Putative beta-actin-like protein 3	POTEKP	-1.5	-1.5	-1.7	-2.0	-0.7	-0.1	-0.5	-0.8	3	3
F1SQX8	PIG Uncharacterized protein	NA	-1.3	-2.0	-1.9	-1.6	-1.1	-1.7	-2.1	-1.8	11	15
P24462	HUMAN Cytochrome P450 3A7	CYP3A7	-1.5	-1.1	-1.7	-2.5	-2.0	-2.3	-2.6	-0.7	1	1
F1SK95	PIG Uncharacterized protein	CPOX	-1.9	-1.3	-1.9	-1.7	0.6	1.3	0.3	0.0	1	1
Q6ZN06	HUMAN Zinc finger protein 813	ZNF813	-1.8	-1.3	-2.1	-1.5	0.4	-0.1	-1.6	-0.9	1	1
E9PAV3	HUMAN Nascent polypeptide-associated complex subunit alpha	NACA	-1.4	-1.9	-2.2	-1.4	1.3	-0.2	-0.1	0.9	1	2
I3LV28	PIG Uncharacterized protein	REPIN1	-3.0	-2.2	-0.9	-0.7	-1.8	-0.6	-0.1	0.5	1	1
Q1AG08	PIG Calsarcin 1	NA	-1.9	-1.4	-1.7	-1.8	-0.1	0.5	0.1	-0.6	25	25
F1RRH7	PIG Uncharacterized protein	LOC100157153	-0.8	-1.9	-2.4	-1.7	-1.4	-0.5	-0.9	-0.3	1	1
Q8NCU1-2	HUMAN Isoform 2 of Uncharacterized protein encoded LINC00521	LINC00521	-0.6	-2.4	-2.1	-1.9	-1.6	-1.9	-1.7	-0.6	1	1
Q6Q781	PIG Calpastatin	CAST	-1.1	-1.9	-2.4	-1.5	1.4	1.8	0.1	1.0	2	5
Q9Y281	HUMAN Cofilin-2	CFL2	-1.9	-2.7	-1.4	-0.9	-1.1	-0.9	-0.4	-1.6	4	6
F1RR11	PIG Uncharacterized protein	LOC100517937	-1.6	-1.4	-1.8	-2.1	-1.6	-1.4	-1.2	-0.4	1	1
P13804	HUMAN Electron transfer flavoprotein subunit alpha, mitochondrial	ETFA	-1.5	-2.1	-1.9	-1.4	0.2	0.0	0.3	0.3	2	1
A5YM48	HUMAN MYBPC3 protein	MYBPC3	-1.7	-2.3	-1.6	-1.2	-2.0	-1.9	-0.5	0.4	5	4
F1SAK6	PIG Uncharacterized protein	ATP5L	-1.7	-1.1	-2.0	-2.2	-2.3	-3.0	-2.8	-1.8	6	6
Q9BXT8	HUMAN RING finger protein 17	RNF17	-2.1	-1.5	-1.7	-1.6	-1.0	-0.9	0.2	0.6	1	1
P05413	HUMAN Fatty acid-binding protein, heart	FABP3	-1.3	-2.5	-1.7	-1.5	-0.2	-0.8	-0.8	-3.0	1	2
Q6ZMZ3	HUMAN Nesprin-3	SYNE3	-1.8	-1.6	-1.9	-1.8	-3.1	-3.1	-5.0	-3.2	1	1
A5GFX4	PIG ATP synthase, H+ transporting, mitochondrial F1 complex, epsilon subunit	ATP5E	-1.6	-1.4	-2.0	-1.9	-2.7	-3.4	-1.4	-0.3	2	2
F1S4B8	PIG Uncharacterized protein	EXD2	-1.9	-2.2	-1.7	-1.2	1.8	0.4	1.4	0.1	1	2

P42765	HUMAN 3-ketoacyl-CoA thiolase, mitochondrial	ACAA2	-1.1	-1.1	-2.2	-2.6	-0.9	-1.3	-1.2	-1.0	2	2
F1SED0	PIG Uncharacterized protein (Fragment)	ACADSB	-1.2	-2.2	-2.0	-1.7	-0.2	0.2	-0.8	-1.4	12	12
K7GMN1	PIG Uncharacterized protein	COX7B	-1.9	-1.7	-1.7	-1.7	-2.8	-2.6	-2.2	1.5	2	2
F1RZW0	PIG Uncharacterized protein (Fragment)	NA	-2.0	-1.8	-1.8	-1.5	0.1	0.1	-0.6	-0.4	5	5
F1RVL5	PIG Uncharacterized protein (Fragment)	MYOM2	-1.4	-1.9	-1.9	-1.8	-1.8	-2.2	-1.7	-0.1	53	63
I3LLA0	PIG Uncharacterized protein	RBM25	-2.4	-2.5	-1.3	-0.8	-1.7	-1.1	1.0	1.5	2	1
Q7SIB7	PIG Phosphoglycerate kinase 1	PGK1	-2.6	-2.7	-1.2	-0.6	-0.5	0.2	0.3	-0.1	30	27
B4DEF0	HUMAN Calsequestrin	NA	-1.8	-1.5	-1.6	-2.2	-1.6	-2.3	-2.9	-2.6	1	1
Q9NRP7	HUMAN Serine/threonine-protein kinase 36	STK36	-2.2	-2.4	-1.5	-1.0	-2.8	-1.5	-1.3	-0.4	1	1
Q9UBB4	HUMAN Ataxin-10	ATXN10	-1.1	-1.8	-2.2	-2.1	-2.0	-0.2	-1.1	-1.2	1	1
I3LBC3	PIG Uncharacterized protein	FHOD3	-2.0	-2.6	-1.5	-1.0	-1.3	-1.2	-0.3	-0.1	2	3
Q96FJ2	HUMAN Dynein light chain 2, cytoplasmic	DYNLL2	-1.6	-2.1	-2.0	-1.4	-1.8	-1.5	-0.8	0.2	1	1
F1RFI1	PIG Elongation factor Tu	TUFM	-1.6	-2.4	-1.7	-1.4	-0.8	-0.8	-1.1	-0.6	23	21
F1RI18	PIG Uncharacterized protein	UQCRQ	-2.1	-1.6	-1.6	-1.8	-2.2	-1.7	-1.2	-0.1	6	7
Q6UV40	PIG Fructose-bisphosphate aldolase A (Fragment)	ALDOA	-1.8	-2.7	-1.6	-1.0	-0.3	0.3	0.4	0.9	5	5
Q9UPS8	HUMAN Ankyrin repeat domain-containing protein 26	ANKRD26	-0.9	-1.0	-2.4	-2.9	-1.8	1.7	-1.0	-0.4	1	1
Q7Z3D6	HUMAN UPF0317 protein C14orf159, mitochondrial	C14orf159	-1.1	-2.3	-2.1	-1.6	-2.1	-1.9	-1.7	-0.9	1	1
P61604	HUMAN 10 kDa heat shock protein, mitochondrial	HSPE1	-1.7	-1.9	-1.7	-1.9	-2.4	-6.3	-3.9	-8.6	1	1
F1SEN8	PIG Uncharacterized protein	LDB3	-1.4	-1.9	-2.0	-1.9	-0.4	0.0	-0.3	-0.6	26	28
Q6ZNE1	HUMAN Eukaryotic translation initiation factor 3 subunit A	EIF3A	-1.3	-1.5	-2.1	-2.3	-0.2	0.0	0.3	-0.4	1	1
H0YCB4	HUMAN Transcription factor p65 (Fragment)	RELA	-1.1	-1.8	-2.3	-1.9	-0.8	-0.7	-1.1	-1.1	1	1
F8W6J3	HUMAN Protein C19orf12	C19orf12	-1.9	-1.5	-1.8	-1.9	0.5	-0.1	0.1	-1.2	1	1
Q59EF7	HUMAN Transducin-like enhancer protein 1 variant (Fragment)	NA	-1.7	-0.6	-2.2	-2.7	1.0	0.4	0.5	-0.6	1	1
H9LBU6	PIG Nuclear factor (Erythroid-derived 2)-like 2 (Fragment)	NFE2L2	-0.9	-2.2	-1.9	-2.2	-0.5	-0.8	-0.5	0.1	1	1
F1RTN1	PIG Uncharacterized protein	LAS1L	-0.9	-0.6	-2.7	-3.1	0.9	-0.8	-2.4	-5.0	1	1
F1SEK2	PIG Uncharacterized protein	TTI1	-1.1	-2.1	-2.1	-1.9	0.0	-0.3	0.5	-0.9	1	1
D0G0B3	PIG Acetyl-Coenzyme A acyltransferase 2	ACAA2	-1.2	-1.6	-2.2	-2.2	-0.5	0.0	0.6	1.2	19	21
F1SA09	PIG Uncharacterized protein	ANKRD9	-1.3	-2.3	-1.7	-1.9	-1.6	-3.7	-4.6	-4.3	1	1
I3L560	PIG Uncharacterized protein (Fragment)	KLB	-1.5	-1.5	-2.0	-2.3	0.2	1.0	0.4	1.3	1	1
Q8IUZ0	HUMAN Leucine-rich repeat-containing protein 49	LRRC49	-1.5	-2.0	-1.9	-1.8	-2.5	-2.4	-1.7	-0.5	1	1
I3LCP0	PIG Uncharacterized protein (Fragment)	LOC100519531	-1.6	-2.5	-1.7	-1.5	-1.3	-1.4	-0.9	-0.1	11	12
F1RLH7	PIG Uncharacterized protein	COX6B	-1.7	-1.1	-2.3	-2.2	-1.2	-1.3	-1.4	0.7	5	4
F1SBC3	PIG Uncharacterized protein	GREB1L	-1.2	0.0	-2.7	-3.4	0.4	0.6	-0.2	0.3	1	1
P12026	PIG Acyl-CoA-binding protein	DBI	-2.4	-2.0	-1.6	-1.4	0.7	-0.3	0.7	0.4	4	6
O76041	HUMAN Nebulette	NEBL	-1.4	-1.9	-2.0	-2.0	-1.7	-0.8	-0.1	1.4	2	5
I3L9K5	PIG Uncharacterized protein	NUCKS1	-2.7	-2.3	-1.5	-1.0	3.3	1.4	0.5	4.0	3	3
I3L7T8	PIG Uncharacterized protein	NA	-1.5	-0.8	-2.2	-3.0	-0.6	-0.9	-2.4	-1.8	2	2
Q0MUU2	PIG Small muscular protein	SMPX	-1.7	-1.6	-2.0	-2.2	0.7	0.1	2.7	-0.1	3	1
F1SSR4	PIG Uncharacterized protein	IVD	-1.5	-1.9	-2.1	-1.9	-0.7	-2.1	-2.4	-2.6	16	17
O43653	HUMAN Prostate stem cell antigen	PSCA	-1.9	-1.5	-2.3	-1.8	-1.0	0.1	0.2	0.3	1	1

Q9H2Y7	HUMAN Zinc finger protein 106	ZNF106	-2.3	-2.0	-1.8	-1.4	-0.2	-1.5	0.1	0.4	1	2
I3LIW2	PIG Uncharacterized protein	LTB4R2	-2.0	-1.5	-2.3	-1.7	-0.2	0.1	-0.6	0.6	1	1
Q7Z7L1	HUMAN Schlafen family member 11	SLFN11	-2.1	-2.6	-1.8	-1.0	0.4	-0.3	-0.2	0.5	1	1
I3LP02	PIG Uncharacterized protein	ACAT1	-2.0	-1.5	-2.1	-1.9	-2.3	-1.1	-0.8	-0.5	21	20
P04075	HUMAN Fructose-bisphosphate aldolase A	ALDOA	-1.9	-2.9	-1.7	-1.1	-0.4	0.0	0.8	1.4	16	16
F1SHA2	PIG Uncharacterized protein (Fragment)	GPD1	-1.5	-2.3	-2.1	-1.6	-1.0	-2.1	-2.4	-1.6	3	3
P42639	PIG Tropomyosin alpha-1 chain	TPM1	-2.0	-0.2	-2.6	-2.8	3.7	2.0	-0.7	-7.4	43	35
F1S814	PIG Uncharacterized protein	PGM1	-2.1	-2.5	-1.7	-1.3	-0.2	-0.8	2.1	1.1	15	19
F1RRN7	PIG Uncharacterized protein	MTMR4	-0.9	-2.2	-2.7	-1.8	2.6	7.0	6.5	-0.1	1	1
P79293	PIG Myosin-7	MYH7	-1.7	-1.8	-2.0	-2.1	-2.5	-2.3	-1.4	-0.1	214	229
P00889	PIG Citrate synthase, mitochondrial	CS	-1.9	-1.1	-2.4	-2.2	-2.3	-2.7	-1.3	-0.3	16	17
P11607	PIG Sarcoplasmic/endoplasmic reticulum calcium ATPase 2	ATP2A2	-1.5	-2.3	-2.0	-1.8	-1.7	-1.1	-0.7	1.2	46	51
K7GMB9	PIG Uncharacterized protein	ADHFE1	-1.1	-2.0	-2.2	-2.3	0.2	0.5	1.4	-1.6	3	3
Q96SD1	HUMAN Protein artemis	DCLRE1C	-1.4	-1.4	-2.6	-2.2	0.0	-0.1	-1.5	-0.2	2	1
P56471	PIG Isocitrate dehydrogenase [NAD] subunit alpha, mitochondrial (Fragments)	IDH3A	-0.7	-2.1	-2.6	-2.2	0.3	0.6	0.0	-0.8	3	3
Q9BR39	HUMAN Junctophilin-2	JPH2	-2.0	-2.4	-2.1	-1.3	-0.2	-0.3	-0.7	0.6	1	3
F1RZQ6	PIG Uncharacterized protein	SLC25A4	-1.8	-0.9	-2.3	-2.8	-2.2	-1.6	2.1	0.2	3	3
H7C2X8	HUMAN cAMP-regulated phosphoprotein 21 (Fragment)	ARPP21	-1.6	-1.5	-2.6	-1.9	-0.5	0.4	-1.6	-0.4	1	1
I3LNB4	PIG Uncharacterized protein (Fragment)	ALDH4A1	-1.6	-2.4	-2.0	-1.7	-0.6	-1.2	-1.7	-1.7	15	17
O14777	HUMAN Kinetochore protein NDC80 homolog	NDC80	-1.9	-1.7	-1.7	-2.5	1.2	-1.1	-0.1	-1.8	1	1
F1SC49	PIG Uncharacterized protein (Fragment)	SORBS1	-2.1	-2.3	-1.8	-1.5	0.0	0.5	-0.4	-0.5	21	26
Q15124	HUMAN Phosphoglucomutase-like protein 5	PGM5	-2.0	-2.7	-1.9	-1.3	-1.5	-1.9	-0.4	-0.2	1	1
A4GR69	PIG Telethonin	TCAP	-1.9	-2.2	-1.9	-1.8	-1.4	-2.6	-2.6	-2.5	6	8
F1RVG6	PIG Uncharacterized protein	NEBL	-2.1	-2.3	-1.8	-1.6	-0.6	-0.3	-0.4	0.0	60	72
O95996	HUMAN Adenomatous polyposis coli protein 2	APC2	-1.7	-2.0	-2.1	-2.0	-2.8	-2.6	-1.1	-0.3	2	2
F1SNU8	PIG Uncharacterized protein	NA	-2.1	-1.9	-2.2	-1.6	1.0	-0.3	1.9	0.1	1	1
F1SNF7	PIG Uncharacterized protein (Fragment)	LOC100511293	-2.7	-2.0	-1.9	-1.2	0.3	-0.5	-0.1	0.5	1	1
Q95266	PIG Calcium/calmodulin-dependent protein kinase type II subunit delta	CAMK2D	-2.1	-3.0	-1.7	-1.1	-1.3	-0.9	-0.3	0.0	16	17
Q5TH69	HUMAN Brefeldin A-inhibited guanine nucleotide-exchange protein 3	ARFGEF3	-1.6	-2.7	-1.8	-1.7	-1.1	-1.2	-1.0	0.2	1	1
K7GPJ6	PIG Uncharacterized protein	MRPL3	-1.1	-2.2	-2.2	-2.3	0.7	0.0	-0.5	0.5	1	1
I3LJ05	PIG Uncharacterized protein	C21orf33	-1.7	-1.9	-2.3	-2.0	-0.9	-1.0	0.7	-1.5	10	10
I3L7N1	PIG Uncharacterized protein	LOC100154079	-1.6	-2.2	-2.3	-1.8	-1.3	-1.7	-0.2	0.2	2	2
Q9Y2P5	HUMAN Bile acyl-CoA synthetase	SLC27A5	-1.5	-2.5	-2.1	-1.7	-1.6	-1.9	-1.6	-1.5	1	1
I3LM00	PIG Uncharacterized protein	NA	-1.3	-1.7	-2.3	-2.7	-0.8	0.0	-0.3	-0.3	1	1
I3L814	PIG Uncharacterized protein (Fragment)	ARSE	-0.4	-1.6	-2.7	-3.2	1.0	1.3	0.0	-0.4	1	1
F1STS2	PIG Uncharacterized protein	ARID1A	-1.4	-1.9	-2.1	-2.6	0.1	-0.5	0.0	-0.2	1	1
F1RW07	PIG Uncharacterized protein	NA	-2.5	-2.1	-1.6	-1.7	-1.4	-1.9	0.2	1.9	3	2
O75323	HUMAN Protein NipSnap homolog 2	GBAS	-1.5	-1.8	-2.4	-2.2	-1.5	-2.0	-1.9	-1.4	6	7
O19069	PIG Succinyl-CoA ligase [ADP/GDP-forming] subunit alpha, mitochondrial	SUCLG1	-1.7	-2.5	-2.2	-1.6	-1.4	-1.4	-0.9	-0.7	11	11
F1SHH1	PIG Uncharacterized protein (Fragment)	EGLN3	-2.4	-1.9	-1.9	-1.9	-1.8	-2.1	-1.7	-0.7	1	1

Q5S3G4	PIG Cytochrome c oxidase subunit 5B, mitochondrial	COX5B	-2.3	-1.0	-2.0	-2.7	-0.8	-2.5	-1.7	-0.3	9	9
F1SGH5	PIG Uncharacterized protein	PDHB	-1.2	-2.3	-2.4	-2.2	-0.7	-0.1	-0.5	-1.0	17	17
I3LS66	PIG Uncharacterized protein	TNNT2	-1.7	-1.0	-2.6	-2.8	1.0	1.1	0.4	-0.6	23	24
O97580	PIG Succinyl-CoA ligase [ADP-forming] subunit beta, mitochondrial (Fragment)	SUCLA2	-1.3	-2.4	-2.3	-2.0	-1.0	-1.1	-0.9	0.1	17	17
F1RU20	PIG Uncharacterized protein	MUS81	-1.0	-1.3	-3.0	-2.7	-1.8	-1.1	-1.6	0.0	1	2
I3LNM6	PIG Uncharacterized protein	CEP250	-2.6	-2.1	-2.0	-1.4	0.3	-0.3	0.9	2.2	2	1
Q8IYR0	HUMAN UPF0704 protein C6orf165	C6orf165	-1.7	-2.0	-2.1	-2.3	-1.4	-1.7	-1.0	-0.4	1	1
B5KJG2	PIG Phosphoglycerate mutase 2	PGAM2	-2.1	-2.8	-1.9	-1.3	0.3	0.4	1.9	-0.1	13	13
Q8N386	HUMAN Leucine-rich repeat-containing protein 25	LRRC25	-2.2	-1.5	-2.0	-2.6	-1.4	-2.3	-2.1	-1.9	1	1
A6NFR6-2	HUMAN Isoform 2 of Putative uncharacterized protein C5orf60	C5orf60	-2.5	-2.5	-1.7	-1.5	-0.8	-0.5	-0.8	-0.6	1	1
Q05639	HUMAN Elongation factor 1-alpha 2	EEF1A2	-2.2	-3.1	-1.7	-1.3	-1.4	-1.1	-1.3	-0.4	8	9
P24964	PIG Cytochrome b	MT-CYB	-1.9	-1.7	-2.4	-2.3	-2.5	-1.8	-1.2	1.1	5	4
F1RKU0	PIG Isocitrate dehydrogenase [NAD] subunit alpha, mitochondrial	IDH3A	-1.2	-2.5	-2.4	-2.2	-0.2	0.0	0.3	-0.7	12	13
F1SAY0	PIG Uncharacterized protein (Fragment)	PTGFRN	-2.4	-2.7	-1.9	-1.3	-0.1	0.5	-0.1	1.4	1	1
Q2HYU2	PIG 6-phosphofructokinase, muscle type	PFKM	-2.2	-2.8	-1.9	-1.5	-1.0	-1.5	0.8	0.2	27	34
P00348	PIG Hydroxyacyl-coenzyme A dehydrogenase, mitochondrial	HADH	-1.7	-1.9	-2.6	-2.2	-2.1	-1.5	-1.7	-0.5	25	27
I3LLT8	PIG Uncharacterized protein (Fragment)	NA	-1.7	-2.3	-2.4	-1.9	0.4	-0.2	0.4	0.9	2	1
F2Z5B6	PIG Tropomyosin alpha-1 chain	TPM1	-2.3	-0.1	-2.8	-3.2	2.5	1.0	-2.0	-9.8	5	5
I3L5Y2	PIG Uncharacterized protein	BAHCC1	-2.2	-2.5	-2.0	-1.8	0.1	-2.0	-2.1	-2.3	1	1
F1RTR7	PIG Uncharacterized protein	YOD1	-1.7	-3.6	-1.9	-1.3	-0.6	-0.7	-0.5	0.4	1	1
K7EL89	HUMAN Spermidine synthase (Fragment)	SRM	-2.6	-2.5	-1.6	-1.7	-0.6	0.1	0.4	-0.3	2	2
G3DR80	HUMAN MHC class I antigen (Fragment)	HLA-A	-2.4	-2.9	-1.7	-1.5	-2.1	-1.2	-0.8	0.6	1	1
P26678	HUMAN Cardiac phospholamban	PLN	-2.4	-2.5	-2.2	-1.4	0.3	-0.4	-0.4	-1.0	1	1
Q96RN5	HUMAN Mediator of RNA polymerase II transcription subunit 15	MED15	-1.2	-3.1	-2.5	-1.9	-1.3	-1.9	-1.7	-0.7	1	1
A8K6S3	HUMAN cDNA FLJ77570, highly similar to Homo sapiens pleckstrin (PLEKHC1)	NA	-1.5	-2.2	-2.7	-2.1	-1.0	-1.3	-1.3	-1.3	1	1
A1XQS3	PIG Mitochondrial NDUFA4	NDUFA4	-1.7	-1.6	-2.7	-2.6	-2.5	-1.9	-2.4	0.8	5	5
B4DW52	HUMAN cDNA FLJ55253, highly similar to Actin, cytoplasmic 1	NA	-1.9	-1.9	-2.2	-2.6	-1.4	-1.9	-1.6	-1.3	2	2
Q9UPW5-3	HUMAN Isoform 3 of Cytosolic carboxypeptidase 1	AGTPBP1	-1.7	-2.2	-2.2	-2.5	-0.1	-0.8	-1.7	-0.4	2	1
I3LM75	PIG Uncharacterized protein	LOC100627469	-2.3	-1.7	-2.5	-2.2	-0.9	0.4	0.7	1.3	2	2
Q6B339	PIG Acyl coenzyme A synthetase long-chain 1 (Fragment)	NA	-2.4	-2.5	-2.0	-1.9	-1.7	-1.4	-0.9	0.5	22	26
M3VH80	PIG Solute carrier family 25 (Aspartate/glutamate carrier), member 13 tv2	SLC25A13	-2.2	-2.0	-2.3	-2.2	-1.2	1.3	-2.2	2.9	1	1
Q9HBB8	HUMAN Cadherin-related family member 5	CDHR5	-2.5	-2.0	-2.1	-2.1	-0.9	-0.4	0.6	-0.2	1	1
O75112-4	HUMAN Isoform 4 of LIM domain-binding protein 3	LDB3	-1.6	-1.9	-2.6	-2.6	-1.0	0.3	0.4	0.0	1	2
I3LCN1	PIG Uncharacterized protein	ENO2	-2.1	-2.9	-2.1	-1.7	-0.6	-1.0	-1.2	0.1	3	2
Q9TDR1	PIG NADH-ubiquinone oxidoreductase chain 5	MT-ND5	-2.3	-2.5	-2.2	-1.8	-1.0	-0.7	0.4	0.0	2	6
P00346	PIG Malate dehydrogenase, mitochondrial	MDH2	-1.4	-2.2	-2.9	-2.4	-1.6	-1.3	-1.3	-0.8	18	18
O02773	PIG Mannosyl-oligosaccharide 1,2-alpha-mannosidase IA	MAN1A1	-2.2	-2.2	-2.4	-2.1	-0.1	0.8	-1.3	0.4	1	1
Q6UAQ8	PIG Electron transfer flavoprotein subunit beta	ETFB	-2.1	-2.8	-2.4	-1.7	-1.2	-1.0	-0.4	-0.3	14	15
F1S5B0	PIG Uncharacterized protein	PPP1R12B	-2.1	-2.9	-2.2	-1.7	1.7	0.2	0.4	0.5	7	10
F1SRC5	PIG Aconitate hydratase, mitochondrial (Fragment)	ACO2	-1.2	-2.0	-3.0	-2.7	-0.5	0.1	-0.2	0.5	2	2

P16276	PIG Aconitate hydratase, mitochondrial	ACO2	-1.7	-2.2	-2.6	-2.4	-2.3	-2.1	-0.8	0.2	41	42
P40939	HUMAN Trifunctional enzyme subunit alpha, mitochondrial	HADHA	-1.1	-2.9	-2.1	-2.9	-2.0	-4.3	-1.5	-7.2	2	1
I3LDE6	PIG Uncharacterized protein	ROPN1L	-0.8	-3.0	-2.8	-2.5	-0.5	0.6	0.1	0.7	1	2
F1SCV4	PIG Uncharacterized protein (Fragment)	KNDC1	-2.1	-2.4	-2.6	-2.0	-1.8	-1.8	0.5	-1.4	2	1
F1SDE0	PIG Uncharacterized protein	ANKRD35	-1.6	-2.8	-2.5	-2.2	-0.5	0.2	0.1	0.3	1	1
P14618-2	HUMAN Isoform M1 of Pyruvate kinase PKM	PKM	-2.2	-3.2	-2.1	-1.8	-1.4	-0.9	0.1	1.0	4	4
O95025	HUMAN Semaphorin-3D	SEMA3D	-2.7	-2.6	-1.8	-2.1	-1.8	-2.6	-0.3	1.1	1	2
I3LAP4	PIG Uncharacterized protein	PCDHGA5	-2.4	-2.2	-2.3	-2.3	-3.7	-2.8	-3.3	0.3	1	1
I3LLU0	PIG Uncharacterized protein	GPD1L	-2.5	-2.8	-2.2	-1.7	-1.2	-1.6	-1.2	-0.1	15	14
Q5TFQ5	HUMAN Signal-regulatory protein delta	SIRPD	-2.2	-1.8	-2.5	-2.8	-2.3	-2.1	-1.2	-2.2	1	1
P46013	HUMAN Antigen KI-67	MKI67	-2.1	-2.8	-2.3	-2.0	-4.2	-4.1	-1.7	1.2	2	2
F1SQA5	PIG Uncharacterized protein	HRH1	-2.8	-2.9	-2.1	-1.4	-0.8	-1.4	-0.7	0.0	1	1
K7GSK1	PIG Uncharacterized protein	ART3	-2.0	-2.7	-3.0	-1.6	-0.4	-0.3	0.4	0.3	1	1
P00503	PIG Aspartate aminotransferase, cytoplasmic	GOT1	-2.1	-3.1	-2.4	-1.9	0.6	0.8	2.7	-0.4	25	27
Q1KYT0	PIG Beta-enolase	ENO3	-2.2	-3.1	-2.2	-1.9	0.5	0.7	3.1	0.2	31	32
Q96AX2	HUMAN Ras-related protein Rab-37	RAB37	-1.9	-3.3	-2.5	-1.7	-0.5	-0.2	0.4	0.6	1	1
Q29594	PIG Creatine kinase B-type (Fragment)	CKB	-2.4	-2.7	-2.5	-1.7	-1.6	0.5	-0.6	-0.1	2	1
B4DP05	HUMAN Formin-2	FMN2	-2.4	-2.4	-2.2	-2.4	-3.2	-2.0	-0.6	0.3	1	1
O75112-2	HUMAN Isoform 2 of LIM domain-binding protein 3	LDB3	-2.1	-2.0	-2.7	-2.6	-1.8	-1.0	-1.1	-1.3	3	2
Q8TC20	HUMAN Cancer-associated gene 1 protein	CAGE1	-2.7	-2.0	-2.4	-2.4	-2.0	-2.2	-1.4	-0.4	1	1
F1SJK4	PIG Uncharacterized protein	SLC24A1	-1.9	-3.3	-2.5	-1.7	-1.4	-1.8	-1.1	-0.1	1	2
P00336	PIG L-lactate dehydrogenase B chain	LDHB	-2.4	-3.6	-2.2	-1.3	-0.9	-2.1	1.0	-1.0	21	19
P29804	PIG Pyruvate dehydrogenase E1 component subunit alpha, somatic form, mitochondrial (Fragment)	PDHA1	-1.8	-3.0	-2.6	-2.1	-1.0	-0.7	-0.8	-0.7	22	26
I3L7Q5	PIG Uncharacterized protein	EVI5L	-2.1	-1.7	-2.9	-2.8	-1.0	-2.1	0.8	0.3	1	3
P62895	PIG Cytochrome c	CYCS	-2.2	-1.6	-2.9	-2.8	-0.6	-0.2	0.6	-0.2	14	14
P06732	HUMAN Creatine kinase M-type	CKM	-1.8	-2.7	-2.7	-2.3	0.5	0.1	-0.1	0.1	1	1
O14594	HUMAN Neurocan core protein	NCAN	-2.5	-2.5	-2.1	-2.5	-2.8	-2.8	-1.5	-0.5	2	1
Q96HC4-2	HUMAN Isoform 2 of PDZ and LIM domain protein 5	PDLIM5	-2.5	-2.9	-2.4	-1.9	-0.7	-0.7	0.2	0.6	4	4
I3LNY9	PIG Uncharacterized protein	PIAS4	-2.4	-2.5	-2.6	-2.2	-2.0	-1.3	-0.1	0.4	1	1
P52943	HUMAN Cysteine-rich protein 2	CRIP2	-2.2	-3.1	-2.9	-1.6	-1.3	-0.4	-0.1	0.0	1	1
P23297	HUMAN Protein S100-A1	S100A1	-1.7	-2.4	-3.0	-2.7	-0.6	-2.7	-4.6	-4.7	1	1
Q6RVD5	PIG Fatty acid coenzyme A ligase long-chain 2 (Fragment)	FACL2	-2.3	-2.8	-2.5	-2.4	-1.7	-1.4	-1.2	0.2	1	3
O97771	PIG Titin (Fragment)	NA	-3.3	-3.3	-1.9	-1.4	-1.9	-1.9	0.3	2.3	9	12
F1RZF6	PIG Uncharacterized protein (Fragment)	RFX7	-2.9	-3.1	-2.3	-1.6	-0.3	0.4	1.6	0.7	1	1
Q14192	HUMAN Four and a half LIM domains protein 2	FHL2	-2.0	-3.4	-2.6	-2.1	-1.3	-1.8	-0.9	-1.3	1	1
D2SQP3	PIG Cysteine and glycine-rich protein 3	CSRP3	-3.3	-3.4	-2.1	-1.2	-2.7	-1.9	-1.6	-2.1	6	6
P33198	PIG Isocitrate dehydrogenase [NADP], mitochondrial (Fragment)	IDH2	-1.5	-2.8	-3.2	-2.6	-1.3	-1.5	-1.4	-1.1	31	29
A8MQ23	HUMAN Serine/threonine-protein kinase WNK2	WNK2	-1.9	-3.2	-2.8	-2.2	-2.2	-1.3	-1.4	0.3	1	1
P00571	PIG Adenylate kinase isoenzyme 1	AK1	-2.8	-3.0	-2.4	-2.0	-0.4	0.0	-0.4	-1.4	10	12
F1RHL9	PIG Uncharacterized protein	ACTN2	-2.0	-3.4	-2.6	-2.2	-1.6	-2.1	-1.4	-0.7	56	55

F1RFE2	PIG Uncharacterized protein (Fragment)	NA	-2.8	-2.2	-2.7	-2.6	-3.9	-3.3	-2.9	-0.9	1	1
Q29371	PIG Triosephosphate isomerase	TPI1	-3.0	-3.7	-2.2	-1.4	-0.4	-0.3	1.5	-1.6	13	12
F1SBS3	PIG Uncharacterized protein	SYCP1	-2.3	-2.8	-2.5	-2.6	-2.2	-2.4	-1.6	0.3	1	1
P49454	HUMAN Centromere protein F	CENPF	-1.7	-1.5	-3.8	-3.3	-3.1	-1.9	-1.5	0.7	1	2
F1SAC1	PIG Uncharacterized protein	ECHS1	-2.3	-3.2	-2.7	-2.3	-2.0	-2.4	-2.5	-2.9	15	13
O15381	HUMAN Nuclear valosin-containing protein-like	NVL	-2.6	-2.6	-2.8	-2.5	-1.3	-0.8	0.0	-1.4	1	1
B7ZAI1	HUMAN highly similar to Homo sapiens 2-aminoadipic 6-semialdehyde dehydrogenase (AASDH)	NA	-3.7	-2.1	-2.4	-2.4	-3.8	-2.1	-2.3	0.9	1	1
I3LER5	PIG Cytochrome c oxidase subunit 4 isoform 1, mitochondrial	COX4I1	-3.4	-1.8	-2.4	-3.1	-3.5	-3.6	-1.9	0.2	4	4
I3L8N1	PIG Uncharacterized protein (Fragment)	BDH1	-2.4	-3.2	-2.9	-2.3	-2.7	-2.8	-3.3	-1.0	3	3
Q8MKF3	PIG Beta-tropomyosin (Fragment)	TPM2	-3.2	-1.8	-3.2	-2.7	1.9	-0.1	-1.8	-4.6	5	4
F1SID7	PIG Uncharacterized protein	MYBPC3	-2.8	-3.8	-2.4	-1.9	-2.1	-2.3	-1.2	0.0	72	84
F1SJX1	PIG Uncharacterized protein	ETFA	-2.1	-3.2	-3.2	-2.5	-0.6	-0.7	-0.5	-1.1	10	9
Q6S8J3	HUMAN POTE ankyrin domain family member E	POTEE	-3.0	-3.0	-2.5	-2.6	-2.2	-3.0	-1.6	-0.2	2	3
I3L8C9	PIG Uncharacterized protein	LOC100626836	-3.2	-2.0	-2.9	-3.0	-2.3	-1.2	-3.1	-2.5	1	1
Q35914	PIG ATP synthase protein 8	MT-ATP8	0.2	-1.7	-5.0	-5.0	-0.5	-3.7	-12.3	-12.8	1	1
I3LTF9	PIG Uncharacterized protein (Fragment)	NA	-3.0	-1.7	-3.5	-3.3	-0.7	-0.9	1.0	-1.7	1	1
F1RTZ9	PIG Uncharacterized protein	MCMDC2	-3.3	-4.0	-2.5	-1.8	-2.0	-1.2	0.5	1.3	2	2
D0G7F6	PIG Triosephosphate isomerase	TPI1	-3.6	-3.9	-2.5	-1.8	-0.4	0.1	1.1	-1.2	4	4
O02772	PIG Fatty acid-binding protein, heart	FABP3	-3.3	-4.3	-2.5	-1.9	-0.6	-1.4	-0.5	-2.1	10	12
P11708	PIG Malate dehydrogenase, cytoplasmic	MDH1	-3.1	-4.1	-2.9	-2.2	-0.6	-0.8	2.1	0.2	21	24
K7ZMG1	PIG Putative cysteine-rich protein 2	CRIP2	-3.3	-4.3	-3.0	-1.8	-2.0	-0.8	0.2	0.2	2	2
P24752	HUMAN Acetyl-CoA acetyltransferase, mitochondrial	ACAT1	-4.0	-3.2	-3.4	-2.8	-1.2	-1.3	-1.6	-0.3	1	1
Q9Y6R4	HUMAN Mitogen-activated protein kinase kinase kinase 4	MAP3K4	-3.5	-3.0	-3.9	-3.1	-2.8	-0.9	0.1	0.4	1	1
Q8IY85	HUMAN EF-hand calcium-binding domain-containing protein 13	EFCAB13	-4.2	-3.8	-2.5	-3.0	-1.8	-2.1	-1.6	1.1	1	1
Q29307	PIG ATPase inhibitor, mitochondrial	ATPIF1	-3.8	-3.1	-3.8	-3.2	1.5	0.3	0.5	1.7	7	6
C3W8F3	PIG Perilipin (Fragment)	LSDP5	-4.3	-2.6	-3.2	-4.0	-3.3	-3.6	2.2	1.0	1	1
P02144	HUMAN Myoglobin	MB	-4.4	-4.2	-3.2	-2.4	0.3	0.7	1.8	0.1	4	2
F1SU18	PIG Uncharacterized protein (Fragment)	FHL2	-3.9	-4.8	-3.2	-2.2	-2.6	-2.6	-1.2	-1.1	5	6
Q6YL37	HUMAN Putative uncharacterized protein	NA	-3.5	-4.1	-3.5	-3.1	-0.5	-0.7	2.2	0.1	1	1
I3LKV1	PIG Uncharacterized protein	HMGN4	-4.1	-4.1	-3.5	-2.7	-0.9	2.3	1.3	3.9	1	1
F1SQJ7	PIG Uncharacterized protein (Fragment)	PSME4	-3.9	-4.6	-3.9	-3.4	-0.9	-0.8	0.7	-1.4	1	2
Q5XLD3	PIG Creatine kinase M-type	CKM	-4.1	-4.3	-4.0	-3.6	-1.1	-0.6	-0.6	-0.9	31	30
A5X5T5	PIG Cardiac troponin I	TNNI3	-3.7	-6.8	-4.5	-4.4	-3.9	-13.2	-12.9	-6.8	2	2
P02189	PIG Myoglobin	MB	-6.8	-6.5	-5.0	-3.8	-1.9	-2.0	1.2	-1.4	20	23

Supplementary Table S11. Peptides monitored by PRM analysis.

Protein	Peptide Sequence	Mass [m/z]	Charge state [z]
**MYOM1**	FTC^HGLTTGQSYIFR	596.6226	3
**MYOM1**	NQVPINVHANPGK	463.2529	3
**MYL3**	ALGQNPTQAEVLR	698.8808	2
**MYL3**	DTGTYEDFVEGLR	751.3439	2
**MYL3**	HVLATLGER	498.2853	2
**UQCRC1**	QLLDLAQK	464.7767	2
**COX6B**	GGDVSVC^EWYR	664.2904	2
**ATP5A1**	ELIIGDR	408.2347	2
**ATP5A1**	ILGADTSVDLEETGR	788.3967	2
**ATP5O**	YATALYSAASK	573.2955	2
**ANT2**	DFLAGGVAAAISK	610.3377	2
**ACO2**	IVYGHLDDPANQEIER	934.9605	2
**ACO2**	NAVTQEFGPVPDTAR	801.3995	2
**MDH2**	GC^DVVVIPAGVPR	669.8636	2
**MYH7**	NLQEEISDLTEQLGSSGK	974.4789	2
**MYH7**	VVDSLQTSLDAETR	767.3914	2
**TPM1**	C^AELEEELK	560.7632	2
**TPM1**	SIDDLEDELYAQK	769.8647	2
**GAPDH**	GAAQNIIPASTGAAK	685.3753	2
**GAPDH**	IVSNASC^TTNC^LAPLAK	910.4557	2
**GAPDH**	VPTPNVSVVDLTC^R	778.9087	2
**GAPDH**	LISWYDNEFGYSNR	882.4048	2

^ Symbol next to cysteine amino acid residue stands for a carbamylation modification.

SUPPLEMENTAL REFERENCES

1 Fernandez-Jimenez, R. *et al.* Myocardial edema after ischemia/reperfusion is not stable and follows a bimodal pattern: imaging and histological tissue characterization. *Journal of the American College of Cardiology* **65**, 315-323, doi:10.1016/j.jacc.2014.11.004 (2015).

2 Fernandez-Jimenez, R. *et al.* Pathophysiology Underlying the Bimodal Edema Phenomenon After Myocardial Ischemia/Reperfusion. *J Am Coll Cardiol* **66**, 816-828, doi:10.1016/j.jacc.2015.06.023 (2015).

3 Fernandez-Jimenez, R. *et al.* Fast T2 gradient-spin-echo (T2-GraSE) mapping for myocardial edema quantification: first in vivo validation in a porcine model of ischemia/reperfusion. *Journal of cardiovascular magnetic resonance : official journal of the Society for Cardiovascular Magnetic Resonance* **17**, 92, doi:10.1186/s12968-015-0199-9 (2015).

4 Nowosielski, M. *et al.* Comparison of wall thickening and ejection fraction by cardiovascular magnetic resonance and echocardiography in acute myocardial infarction. *Journal of cardiovascular magnetic resonance : official journal of the Society for Cardiovascular Magnetic Resonance* **11**, 22, doi:10.1186/1532-429X-11-22 (2009).

5 Martinez-Acedo, P. *et al.* A novel strategy for global analysis of the dynamic thiol redox proteome. *Mol Cell Proteomics* **11**, 800-813, doi:10.1074/mcp.M111.016469 (2012).

6 Martinez-Bartolome, S. *et al.* Properties of average score distributions of SEQUEST: the probability ratio method. *Mol Cell Proteomics* **7**, 1135-1145, doi:10.1074/mcp.M700239-MCP200 (2008).

7 Bonzon-Kulichenko, E., Garcia-Marques, F., Trevisan-Herraz, M. & Vazquez, J. Revisiting peptide identification by high-accuracy mass spectrometry: problems

associated with the use of narrow mass precursor windows. *J Proteome Res* **14**, 700-710, doi:10.1021/pr5007284 (2015).

8 Navarro, P. & Vazquez, J. A refined method to calculate false discovery rates for peptide identification using decoy databases. *J Proteome Res* **8**, 1792-1796, doi:10.1021/pr800362h (2009).

9 Navarro, P. *et al.* General statistical framework for quantitative proteomics by stable isotope labeling. *J Proteome Res* **13**, 1234-1247, doi:10.1021/pr4006958 (2014).

10 Cox, J. & Mann, M. in *Nat Biotechnol* Vol. 26 1367-1372 (2008).

11 Cox, J. *et al.* in *J. Proteome Res.* Vol. 10 1794-1805 (2011).

12 Cox, J. *et al.* Accurate proteome-wide label-free quantification by delayed normalization and maximal peptide ratio extraction, termed MaxLFQ. *Mol Cell Proteomics* **13**, 2513-2526, doi:10.1074/mcp.M113.031591 (2014).

13 Schwanhausser, B. *et al.* Global quantification of mammalian gene expression control. *Nature* **473**, 337-342, doi:10.1038/nature10098 (2011).

14 MacLean, B. *et al.* Skyline: an open source document editor for creating and analyzing targeted proteomics experiments. *Bioinformatics* **26**, 966-968, doi:10.1093/bioinformatics/btq054 (2010).

15 Garcia-Marques, F. *et al.* A Novel Systems-Biology Algorithm for the Analysis of Coordinated Protein Responses Using Quantitative Proteomics. *Mol Cell Proteomics* **15**, 1740-1760, doi:10.1074/mcp.M115.055905 (2016).

16 Calvano, S. E. *et al.* A network-based analysis of systemic inflammation in humans. *Nature* **437**, 1032-1037, doi:10.1038/nature03985 (2005).

17 Ficenec, D. *et al.* Computational knowledge integration in biopharmaceutical research. *Brief Bioinform* **4**, 260-278 (2003).

18 Huang da, W. *et al.* Extracting biological meaning from large gene lists with DAVID. *Curr Protoc Bioinformatics* **Chapter 13**, Unit 13 11, doi:10.1002/0471250953.bi1311s27 (2009).
